# Enantioselective isothiourea-catalysed reversible Michael addition of aryl esters to 2-benzylidene malononitriles†

**DOI:** 10.1039/d3sc02101g

**Published:** 2023-06-02

**Authors:** Alastair J. Nimmo, Jacqueline Bitai, Claire M. Young, Calum McLaughlin, Alexandra M. Z. Slawin, David B. Cordes, Andrew D. Smith

**Affiliations:** a EaStCHEM, School of Chemistry, University of St Andrews St Andrews Fife KY16 9ST UK ads10@st-andrews.ac.uk

## Abstract

Catalytic enantioselective transformations usually rely upon optimal enantioselectivity being observed in kinetically controlled reaction processes, with energy differences between diastereoisomeric transition state energies translating to stereoisomeric product ratios. Herein, stereoselectivity resulting from an unusual reversible Michael addition of an aryl ester to 2-benzylidene malononitrile electrophiles using an isothiourea as a Lewis base catalyst is demonstrated. Notably, the basicity of the aryloxide component and reactivity of the isothiourea Lewis base both affect the observed product selectivity, with control studies and crossover experiments indicating the feasibility of a constructive reversible Michael addition from the desired product. When this reversible addition is coupled with a crystallisation-induced diastereomer transformation (CIDT) it allows isolation of products in high yield and stereocontrol (14 examples, up to 95 : 5 dr and 99 : 1 er). Application of this process to gram scale, plus derivatisations to provide further useful products, is demonstrated.

## Abstract 2

An unusual reversible addition of aryl esters to 2-benzylidene malononitriles using an isothiourea Lewis base catalyst is demonstrated with control studies indicating the feasibility of a constructive reversible Michael addition.

## Graphical abstract

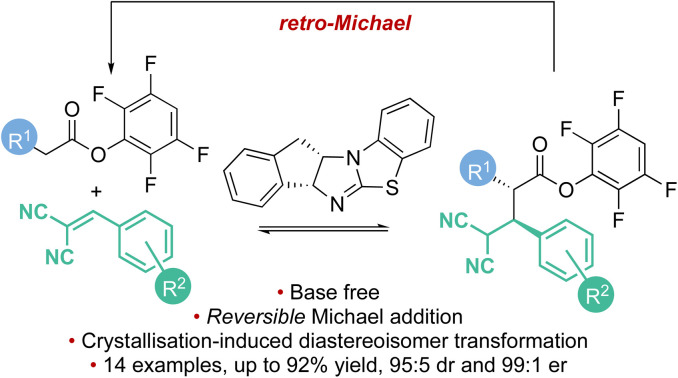

## 1. Introduction

**Scheme 1:**
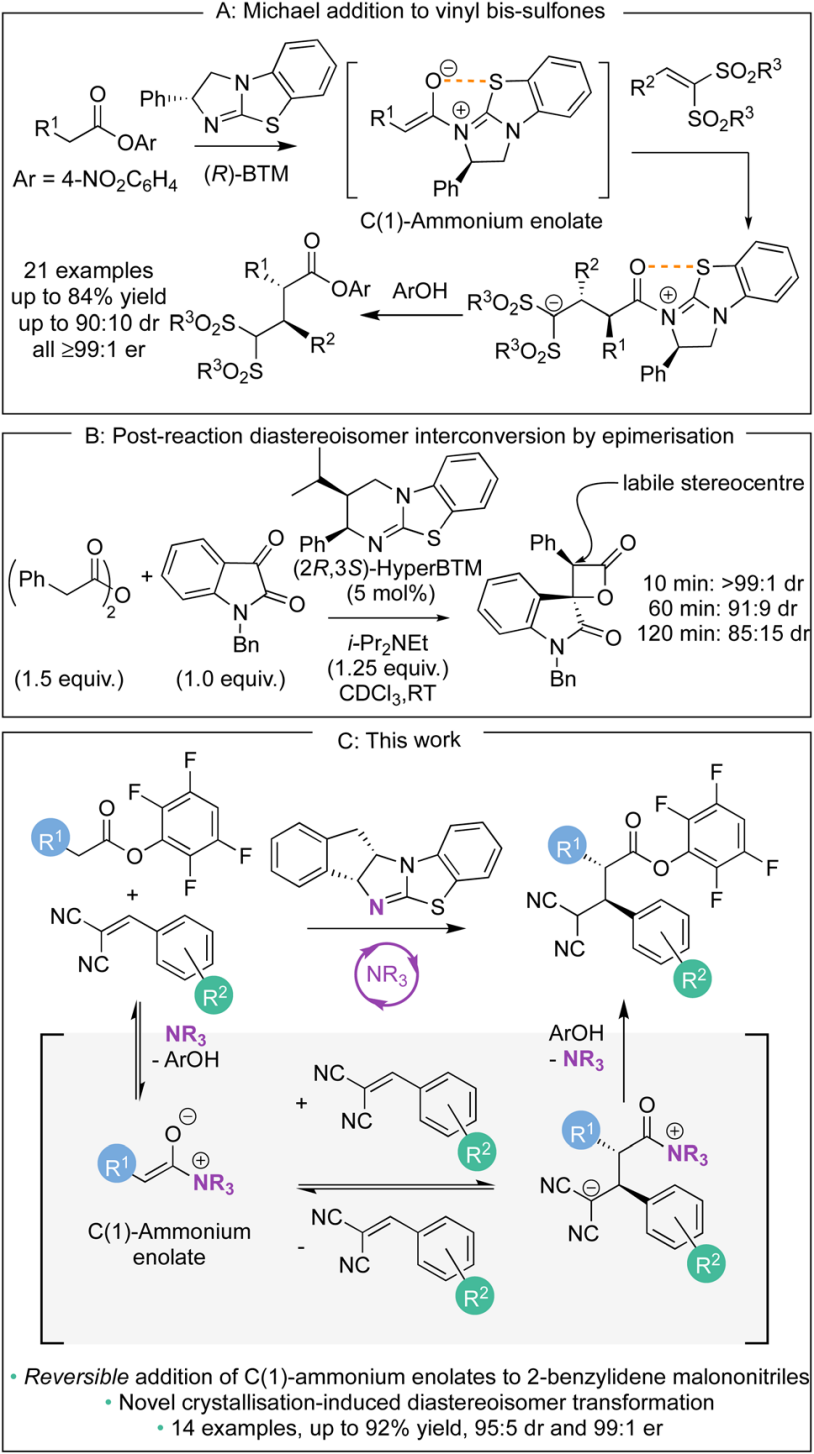
Examples of C(1)-ammonium enolate reactivity in isothiourea catalysis.

Over the last decade, methods for the generation and controlled reactivity of C(1)-ammonium enolates have significantly expanded, with the use of isothioureas alongside cinchona alkaloids pivotal in these advances.^1^ In recent work, the use of electron-deficient aryl esters as C(1)-ammonium enolate precursors in conjunction with isothiourea catalysts has been developed to broaden the electrophiles traditionally compatible with these intermediates (Scheme 1A).^2^ The ability of the aryl ester to acylate the Lewis basic isothiourea, liberating the corresponding aryloxide, that can subsequently act as a nucleophile to turn over the Lewis base catalyst after a constructive enantioselective reaction is key to this strategy.^3^ In these processes the aryloxide is required to fulfil the role of a Brønsted base to generate the C(1)-ammonium enolate as well as a Brønsted acid to protonate the post reaction acyl-ammonium species. The amphoteric aryloxide must therefore possess a delicate balance of p*K*_a_, nucleophilicity, and nucleofugality for a reaction to be successful. This approach has allowed a range of enantioselective processes to be developed, ranging from [2,3]-sigmatropic rearrangements^2*a*–*d*^ to Michael additions,^2*e*^ as well as dual catalytic methods that involve transition metal^2*f*–*m*^ or Brønsted acid co-catalysts.^2*n*^ As a representative example of this approach, in previous work we demonstrated the enantioselective base-free isothiourea-catalysed Michael addition of aryl ester pronucleophiles to vinyl bis-sulfones, generating α-functionalised products containing two contiguous tertiary stereogenic centres in excellent yield and stereoselectivity (all ≥99 : 1 er, Scheme 1A).^2*e*^ The stereoselectivity observed in these processes using C(1)-ammonium enolates is usually considered to rely upon irreversible nucleophilic addition under kinetic control of the reaction, with energy differences between diastereoisomeric transition states translating to stereoisomeric product ratios. In certain circumstances, post reaction equilibration at an acidic position within the product can result in epimerisation, as for example has been observed at the C(3)-position of β-lactones (Scheme 1B).^4^ To the best of our knowledge, stereoselectivity that occurs in a reaction process that involves reversible addition of a C(1)-ammonium enolate to an electrophile generated using isothioureas has not been demonstrated to date. In this manuscript, the expansion of the scope of the base-free enantioselective Michael addition of aryl ester pronucleophiles to include 2-benzylidene malononitrile electrophiles is reported (Scheme 1C). Significantly, judicious choice of aryl ester, solvent, and isothiourea proved crucial for optimal yield and stereoselectivity. Mechanistic investigation demonstrated the ability of both the aryloxide and the isothiourea catalyst to promote retro-Michael addition, a process previously unknown for isothiourea-catalysed Michael additions. In some cases, the reversibility of the Michael addition was harnessed alongside a crystallisation-induced diastereomer transformation (CIDT), giving products with enhanced diastereoselectivity (up to 95 : 5 dr) and with excellent enantioselectivity (up to 99 : 1 er).

## 2. Results and discussion

### 2.1 Initial reactivity, observations and recognition of a reversible Michael addition process

**Scheme 2:**
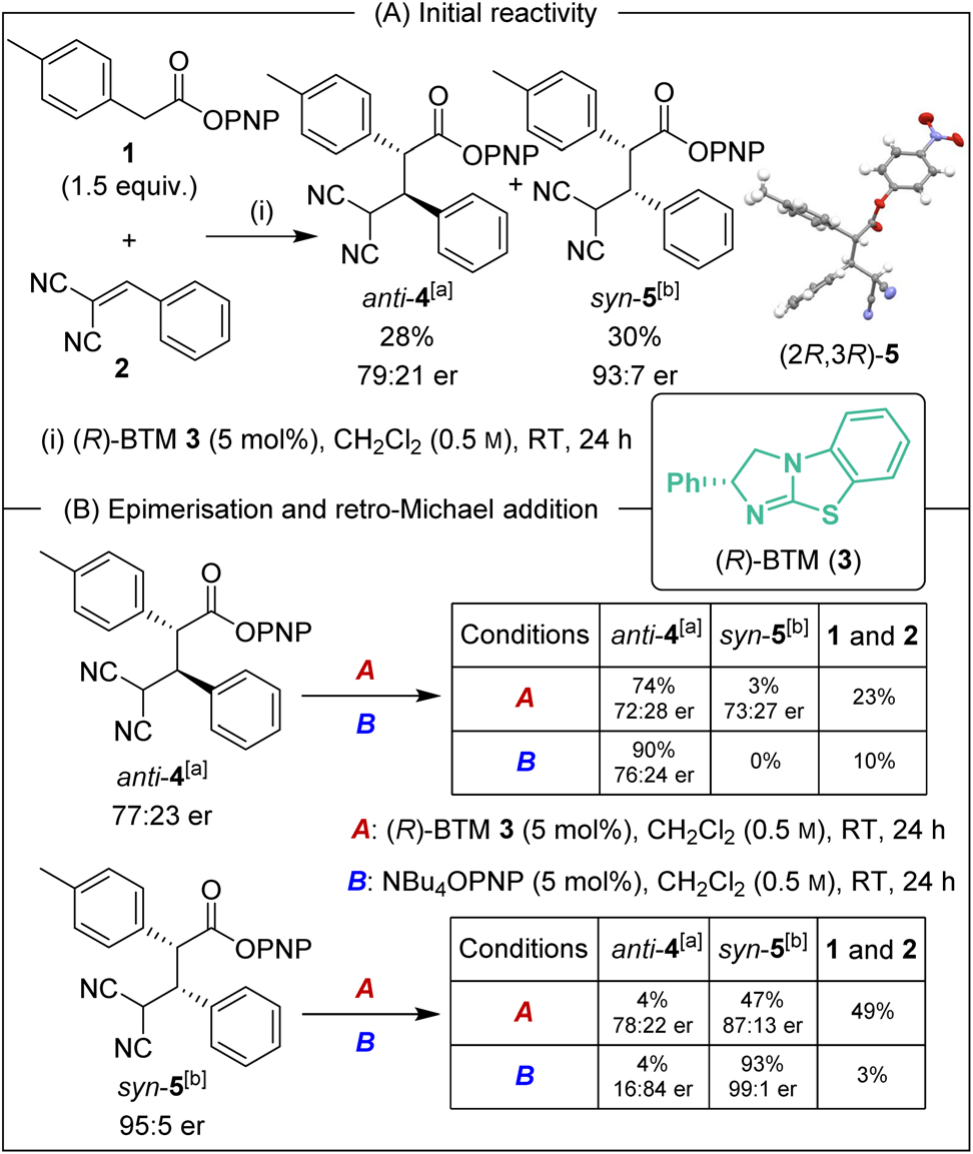
(A) Initial reactivity and (B) evidence of retro-Michael addition and epimerisation. All ers determined by HPLC analysis on a chiral stationary phase. [a] er represents (2*R*,3*S*) : (2*S*,3*R*). [b] er represents (2*R*,3*R*) : (2*S*,3*S*).

Initial investigations began with the reaction of *p*-nitrophenyl (PNP) ester 1 with 2-benzylidene malononitrile 2 and (*R*)-BTM 3 (5 mol%) in CH_2_Cl_2_. The desired Michael addition product was formed in 58% yield as an equal (49 : 51 dr) mixture of diastereoisomers, giving *anti*-4 with moderate enantioselectivity (79 : 21 er) and *syn*-5 with high enantioselectivity (93 : 7 er) (Scheme 2A). The absolute (2*R*,3*R*)-configuration within *syn*-5 was unambiguously established by X-ray analysis.^5^ Initially hypothesising that the difference in enantioselectivity between the diastereoisomeric products may be due to selective *in situ* epimerisation, the separable products *anti*-4 (77 : 23 er, >95 : 5 dr) and *syn*-5 (95 : 5 er, >95 : 5 dr) were treated with both (*R*)-BTM and tetrabutylammonium *p*-nitrophenoxide (Scheme 2B). Interestingly, treatment of both *anti*-4 and *syn*-5 with (*R*)-BTM returned significant equimolar quantities (23% and 49% respectively) of catalysis substrates PNP ester 1 and 2-benzylidene malononitrile 2 (condition A), consistent with retro-Michael addition under these conditions. To date, isothiourea-promoted retro-Michael additions has not been observed, although a related retro-Michael addition to generate benzylidene malononitriles has been reported by Kanger.^6^ Treatment of both *anti*-4 and *syn*-5 with tetrabutylammonium *p*-nitrophenoxide also promoted retro-Michael addition, although to a significantly reduced extent, giving 10% and 3% of PNP ester 1 and 2-benzylidene malononitrile 2 respectively (conditions B). Consistent with our hypothesis, *p*-nitrophenoxide led to epimerisation at C(2) of *syn*-5 but not *anti*-4. Importantly, HPLC analysis showed that epimerisation of (2*R*,3*R*)-*syn*-5 at C(2) produced (2*S*,3*R*)-*anti*-4 that is the enantiomer of (2*R*,3*S*)-*anti*-4 arising from the (*R*)-BTM-catalysed reaction. This is consistent with epimerisation of *syn*-5 contributing to the reduced enantioselectivity observed for *anti*-4 in Scheme 2A.

### 2.2 Importance of aryloxide

Based upon these results, further work considered changing the basicity of the *in situ* generated aryloxide through variation of the aryl ester. It was considered this approach could mitigate both epimerisation and retro-Michael addition both of which are expected to proceed *via* deprotonation at C(2)- and C(4)-respectively within the product (Table 1). While our previous studies have often shown that *p*-nitrophenyl esters deliver optimal yields and stereoselectivities,^2*a*–*c*,2*p*–*r*,7^ Snaddon and Waser have both utilised pentafluorophenyl esters in dual isothiourea/palladium-catalysed α-allylations^2*f*–*j*^ and α-chlorinations^8^ respectively. The steric and electronic effects of the aryloxide leaving group were therefore examined to gain insight into their effects in the model reaction process. The ester of 3,5-bis(trifluoromethyl)phenol 6 (bis-CF_3_, p*K*_a_ 8.26;^9^ compared with *p*-nitrophenol, p*K*_a_ 7.16 (ref. 10)) gave a 48 : 52 mixture of diastereoisomers 10 : 11 with improved enantioselectivity for both diastereoisomers (entry 2, 87 : 13 er_*anti*_, 97 : 3 er_*syn*_). The ester of 2,4,6-trichlorophenol (TCP, p*K*_a_ 5.99)^11^7 was completely unreactive presumably due to steric hindrance (entry 3). Pleasingly, using the esters of 2,3,4,5,6-pentafluorophenol (PFP, p*K*_a_ 5.53)^11^8 (entry 4) and 2,3,5,6-tetrafluorophenol (TeFP, p*K*_a_ 6.00)^11^9 (entry 5) gave the corresponding products 12 : 13 and 14 : 15 with improved diastereo- and enantioselectivity. The TeFP esters were chosen for further optimisation and control studies.

**Table 1:** Variation of aryl ester^a^

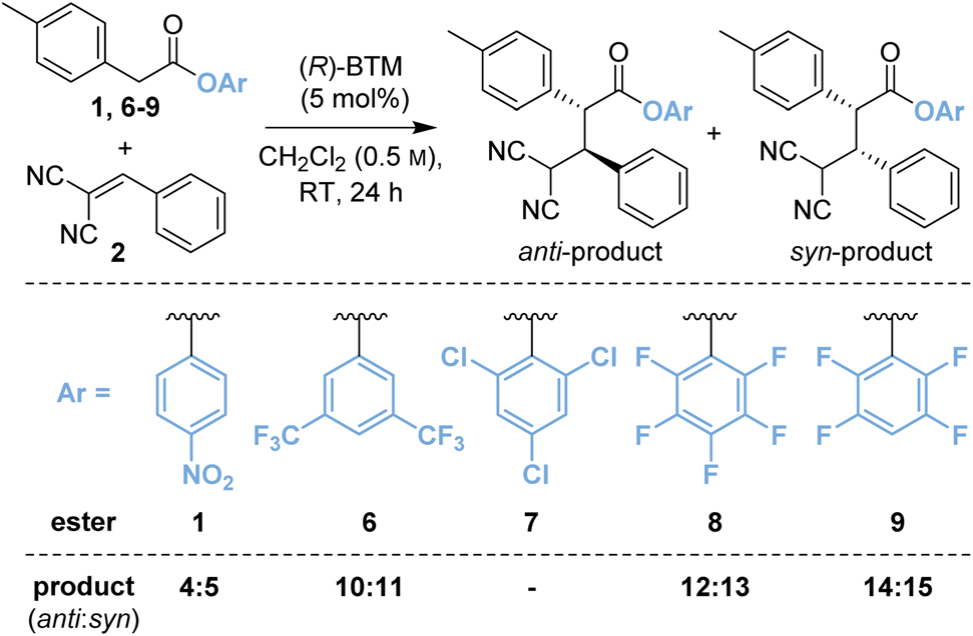						
Entry	Aryl ester	Yield^b^ (%)	Product	dr^c^	er	er
			*anti*- : *syn*-		*anti* d	*syn* d
1	1	58	4 : 5	49 : 51	79 : 21	93 : 7
2	6	48	10 : 11	48 : 52	87 : 13	97 : 3
3	7	0	—	—	—	—
4	8	48	12 : 13	74 : 26	89 : 11	93 : 7
5	9	51	14 : 15	68 : 32	89 : 11	95 : 5

a Reactions performed on 0.5 mmol scale with 1.0 equiv. of 2 and 1.5 equiv. of ester.

b Combined yield of diastereoisomers by ^1^H NMR analysis of the crude reaction mixture using 1,3,5-trimethoxybenzene internal standard.

c Determined by ^1^H NMR analysis of the crude product.

d Determined by HPLC analysis on a chiral stationary phase.

**Scheme 3:**
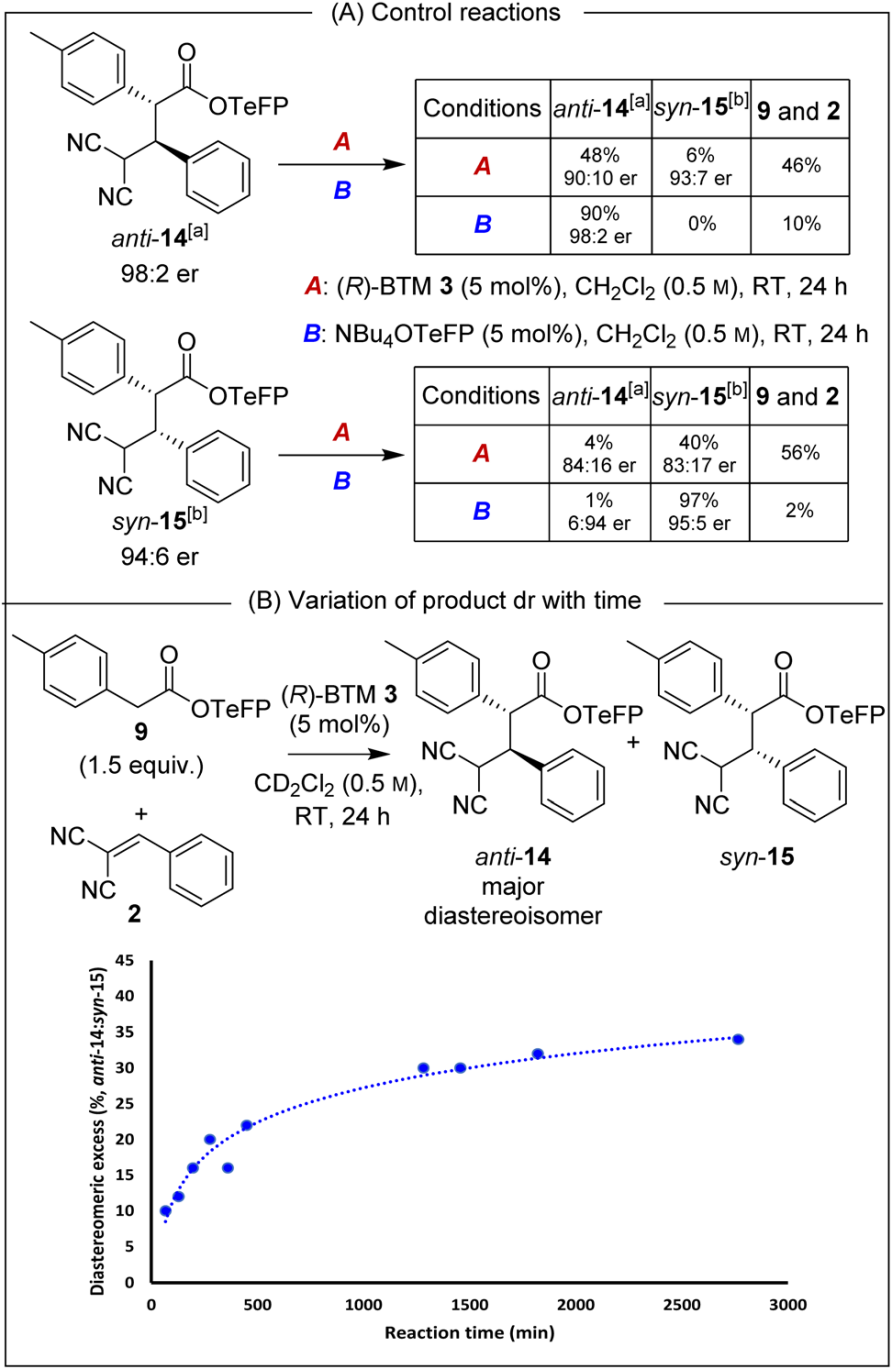
Probing retro-Michael addition and epimerisation in TeFP esters. All ers determined by HPLC analysis on a chiral stationary phase. [a] er represents (2*R*,3*S*) : (2*S*,3*R*). [b] er represents (2*R*,3*R*) : (2*S*,3*S*).

Isolated enantioenriched TeFP products *anti*-14 (>95 : 5 dr, 98 : 2 er) and *syn*-15 (>95 : 5 dr, 94 : 6 er) were treated analogously with (*R*)-BTM and NBu_4_OTeFP (Scheme 3A). Treatment with (*R*)-BTM resulted in increased retro-Michael addition compared to PNP products *anti*-4 and *syn*-5 (46% and 56% *vs.* 23% and 49%). Moreover, treatment of *syn*-15 with 2,3,5,6-tetrafluorophenoxide showed four times less epimerisation than *syn*-5 with *p*-nitrophenoxide (1% *vs.* 4%), consistent with our hypothesis that the basicity of aryloxide was important for both yield and stereoselectivity. To further probe the selectivity observed in the TeFP ester series, the evolution of product diastereoselectivity with time under these reaction conditions was monitored by ^1^H NMR spectroscopic analysis in CD_2_Cl_2_ (Scheme 3B). At low conversions and short reaction times the dr of *anti-*14 : *syn-*15 was moderate (55 : 45 dr) but increased with time (*anti-*14 : *syn-*15 65 : 35 dr after 24 hours), consistent with the retro-Michael control studies. Interestingly, attempted separation of the diastereoisomeric products by chromatographic purification on silica often led to significant variation in isolated product er (ranging from 92 : 8 to 99 : 1 er). Extensive studies indicated this to be due to the phenomenon of self-disproportionation of enantiomers (SDE)^12^ with the er of a given sample not representative of the entire reaction mixture. To ensure that spurious product enantiomeric ratios were not reported during further optimisation of reaction conditions, the products were therefore purposefully isolated as a mixture of diastereoisomers.

### 2.3 Reaction optimisation

The reaction conditions were further optimised using TeFP ester 9 (Table 2). Increasing the reaction concentration to 1.0 m led to improved yield (72%) with similar levels of diastereo- and enantiocontrol (entry 1). Doubling the catalyst loading to 10 mol% improved diastereoselectivity (85 : 15 dr) but reduced enantioselectivity, particularly of syn-15 (83 : 17 er_*syn*_, entry 2). Attempting to improve conversion, the reaction was carried out at 40 °C (entry 3) but this led to decreased yield and stereoselectivity. Variation of the reaction solvent indicated that in both EtOAc and dimethyl carbonate (DMC) the product *anti-*14 : *syn*-15 precipitated from the reaction mixture with an accompanied increase in yield (78% and 76%, entries 4 and 5). Precipitation was also observed in Et_2_O, giving *anti-*14 : *syn*-15 with improved yield and stereoselectivity (entry 6). Increasing the reaction time in Et_2_O to 48 h gave *anti*-14 in quantitative yield (entry 7). Simple filtration of the reaction mixture afforded *anti*-14 as a single diastereoisomer (>95 : 5 dr), indicating the feasibility of a CIDT.^13^ While a range of highly selective CIDT processes have been developed, these processes are generally underutilised as a strategy for enantioselective synthesis.^13*a*^ When demonstrated, CIDT processes often provide routes to a single product diastereoisomer by crystallisation from an equilibrating mixture of isomers. For example, Johnson and co-workers recently harnessed a doubly stereodivergent CIDT process that involved a chiral bifunctional iminophosphorane catalysed enantioselective conjugate addition process between a nitroalkane and a Michael acceptor. This procedure gave γ-nitro-β-ketoamides containing three contiguous stereogenic centres in excellent yield and stereoselectivity (typically >95 : 5 dr, >95 : 5 er) due to catalyst-controlled epimerisation and subsequent CIDT.^14^ In the case described herein, the diastereoisomeric products interconvert through reversible Michael-addition, and to the best of our knowledge is the first CIDT process of its kind, with precipitation of the product beneficial as it can no longer participate in the retro-Michael addition. Building upon these results, various isothiourea catalysts were next screened to improve product enantioselectivity in this protocol. When (*S*)-TM 16 was used the reaction rate significantly decreased, giving only 45% yield after 168 h (entry 8). The use of (2*S*,3*R*)-HyperBTM 17 allowed the reaction time to be reduced to 24 h whilst maintaining the excellent yield and diastereoselectivity, but with reduced 79 : 21 er (entry 9). (*S*)-*i*-Pr-BTM 18 (ref. 15) gave *anti*-14 in 99% yield as a single diastereoisomer with excellent 98 : 2 er, albeit with a reaction time of 96 h (entry 10). Optimal stereocontrol and reduced reaction time was observed using (4b*R*,11a*S*)-fused-BTM 19 (ref. 15) for 24 h, giving *anti*-14 with excellent enantioselectivity (99 : 1 er) with similarly excellent yield and dr (entry 11). Finally, Et_2_O could be substituted for the more industrially preferable^16^ CPME to give the optimised reaction conditions where again a CIDT process was observed. Filtration of the product precipitate directly gave *anti*-14 in 65% yield, >95 : 5 dr and 99 : 1 er, with the filtrate giving a 63 : 37 mixture of *anti-*14 : *syn*-15 (both 95 : 5 er, entry 12). Alternatively, concentration of the reaction mixture, followed by chromatographic purification gave *anti*-14 with 95 : 5 dr in 92% isolated yield with 99 : 1 er (entry 13).

**Table 2:** Optimisation of reaction conditions^a^

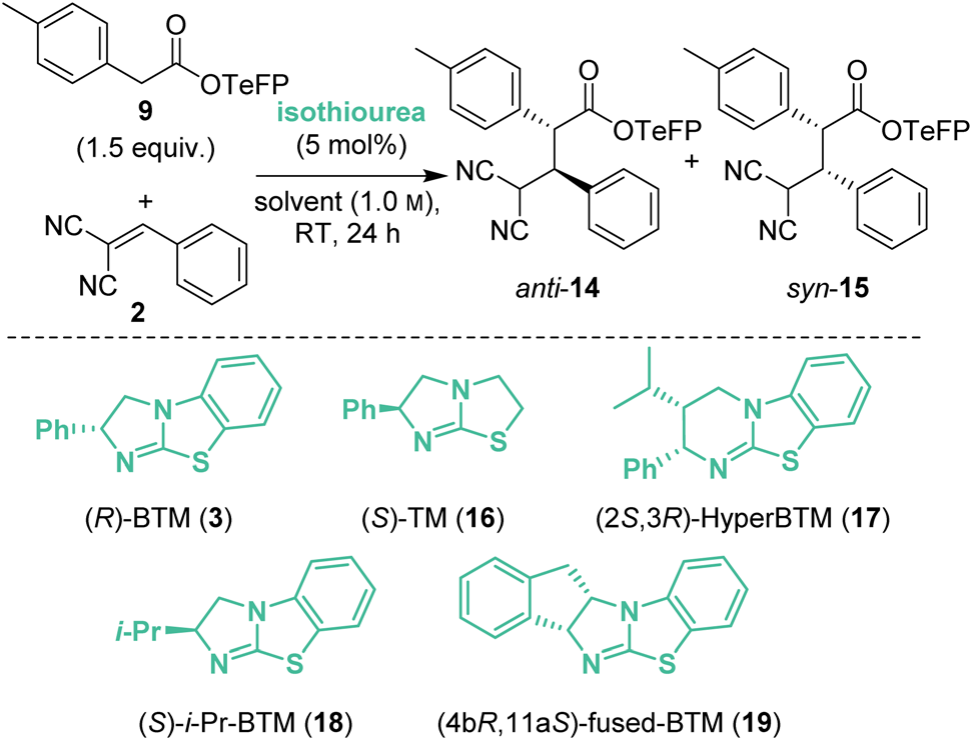						
Entry	Cat.	Solvent	Yield^b^ (%)	dr^c^	er	er
					*anti*-14^d^	*syn*-15^d^
1	3	CH_2_Cl_2_	72	72 : 28	87 : 13	94 : 6
2^e^	3	CH_2_Cl_2_	76	85 : 15	85 : 15	83 : 17
3^f^	3	CH_2_Cl_2_	64	83 : 17	82 : 18	82 : 18
4^g^	3	EtOAc	78	68 : 32	82 : 18	90 : 10
5^g^	3	DMC	76	66 : 34	85 : 15	94 : 6
6^g^	3	Et_2_O	80	75 : 25	89 : 11	92 : 8
7^g^^,^^h^	3	Et_2_O	Quant.	>95 : 5	88 : 12	—
8^g^^,^^i^	16	Et_2_O	45	74 : 26	87 : 13	>99 : 1
9^g^	17	Et_2_O	99	>95 : 5	79 : 21	—
10^g^^,^^j^	18	Et_2_O	99	>95 : 5	98 : 2	—
11^g^	19	Et_2_O	91	95 : 5	99 : 1	—
12^g^	19	CPME	65	>95 : 5	99 : 1	—
13^g^	19	CPME	92	95 : 5	99 : 1	—

a Reactions performed on 0.5 mmol scale with 1.0 equiv. of 2 and 1.5 equiv. of 9.

b Combined yield of diastereoisomers determined by ^1^H NMR analysis of the crude reaction mixture using 1,3,5-trimethoxybenzene internal standard.

c Determined by ^1^H NMR analysis of the crude reaction mixture.

d Determined by HPLC analysis on a chiral stationary phase.

e 10 mol% (*R*)-BTM used.

f Reaction temperature was 40 °C.

g Precipitation of product from reaction mixture.

h 48 h reaction time.

i 168 h reaction time.

j 96 h reaction time.

### 2.4 Scope and limitations of the reversible Michael addition

**Scheme 4:**
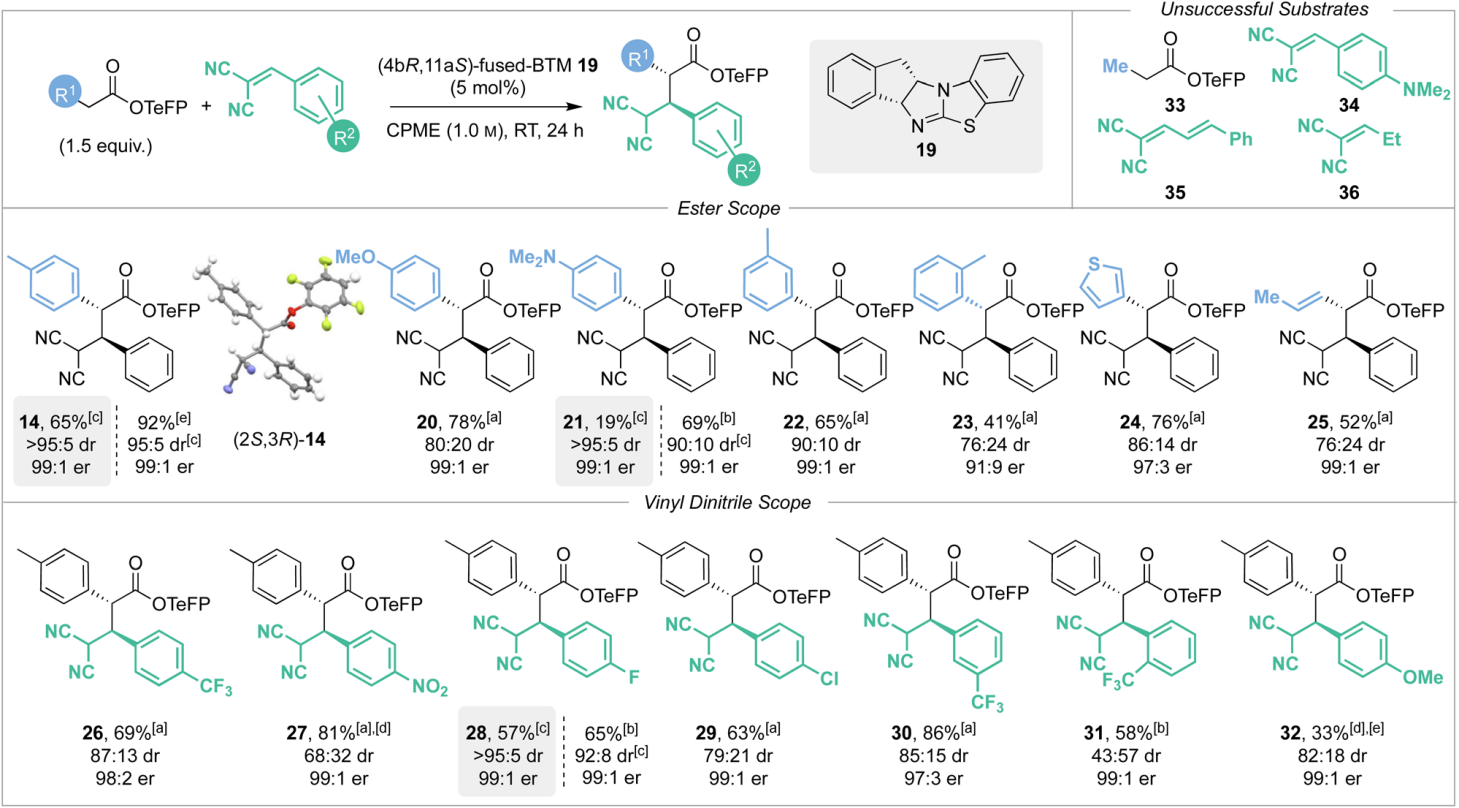
Scope and limitations. Reactions performed on 0.5 mmol scale. dr determined by ^1^H NMR analysis of the crude reaction mixture OR after direct filtration. er determined by HPLC analysis on a chiral stationary phase. [a] Combined yield of separable diastereoisomers. [b] Yield of major diastereoisomer. [c] CIDT process in operation; grey highlighted boxes indicate yield, dr and er isolated after filtration. [d] CH_2_Cl_2_ was solvent. [e] Combined yield of inseparable diastereoisomers.

With the optimised reaction conditions established the scope and limitations of the Michael addition process was investigated using (4b*R*,11a*S*)-fused-BTM 19 (Scheme 4). The relative and absolute configuration of (2*R*,3*S*)-14 was confirmed by single crystal X-ray crystallography, with the configuration within all other products assigned by analogy.^17^ A variety of TeFP esters were synthesised from the corresponding α-aryl, α-alkenyl and α-alkyl substituted carboxylic acids and tested in this protocol. Electron-donating 4-MeOC_6_H_4_ and 4-Me_2_NC_6_H_4_ substituents were well tolerated, giving products 20 and 21 in high yields and with excellent stereoselectivity (78% and 69% respectively, both 99 : 1 er). CIDT of 21 led to moderate isolated yield by direct filtration (19%, >95 : 5 dr, 99 : 1 er), or alternatively concentration of the reaction mixture followed by purification led to improved diastereoselectivity (90 : 10 dr) against that of 20 (80 : 20 dr). 3-MeC_6_H_4_ substitution gave 22 in good yield and excellent stereoselectivity (65%, 90 : 10 dr, 99 : 1 er) without a CIDT process in operation. *Ortho*-substitution was also tolerated, giving product 23 but with reduced yield and enantioselectivity (41%, 91 : 9 er, 76 : 24 dr). Extension to incorporate 3-thiophenyl and prop-1-enyl substituents gave the corresponding products 24 and 25 in good yields (76% and 52%) with excellent enantioselectivity (97 : 3 and 99 : 1 er). Consistent with our previous studies,^2*e*,*n*,2*p*^ a notable limitation of this process showed that an α-alkyl substituent was not tolerated, with Me-substituted TeFP ester 33 proving unreactive and returning only starting material. The scope and limitations with respect to the vinyl dinitrile Michael acceptors was next investigated, with a small selection synthesised by Knoevenagel condensation of malononitrile with the requisite aldehyde. Electron-withdrawing 4-F_3_CC_6_H_4_ and 4-O_2_NC_6_H_4_ substituents gave products 26 and 27 respectively in high yields (69% and 81%) with excellent enantioselectivity (98 : 2 and 99 : 1 er). Halogen containing 4-FC_6_H_4_ and 4-ClC_6_H_4_ substituents were also well tolerated giving products 28 and 29 in good yields (65% and 63%), again with excellent enantioselectivity (both 99 : 1 er). CIDT of product 28 allowed its isolation in good yield by direct filtration (57%, >95 : 5 dr, 99 : 1 er), while alternatively concentration of the reaction mixture followed by purification still gave 28 produced with excellent diastereoselectivity (92 : 8 dr). 3-F_3_CC_6_H_4_ substitution gave product 30 in very high yield (86%) with excellent enantioselectivity (97 : 3 er). 2-F_3_CC_6_H_4_ substitution gave product 31 in reduced 58% yield, presumably due to increased steric hindrance biasing the equilibrium, with the excellent enantioselectivity (99 : 1 er) maintained. Incorporation of an electron-donating 4-MeOC_6_H_4_ substituent led to reduced conversion to product reflecting the assumed reduced electrophilicity of the Michael acceptor containing this conjugating donor substituent, giving product 32 with excellent enantioselectivity (99 : 1 er) but in low 33% yield. Consistent with this observation, incorporation of the stronger electron-donating 4-Me_2_NC_6_H_4_ substituent within 34 was not tolerated, returning only starting material. Attempted replacement of the β-aryl substituent within either cinnamyl 35 or ethyl 36 substituted vinyl dinitrile Michael acceptors again returned only starting material and so represent limitations of this methodology.

### 2.5 Scale-up and derivatisation

**Scheme 5:**
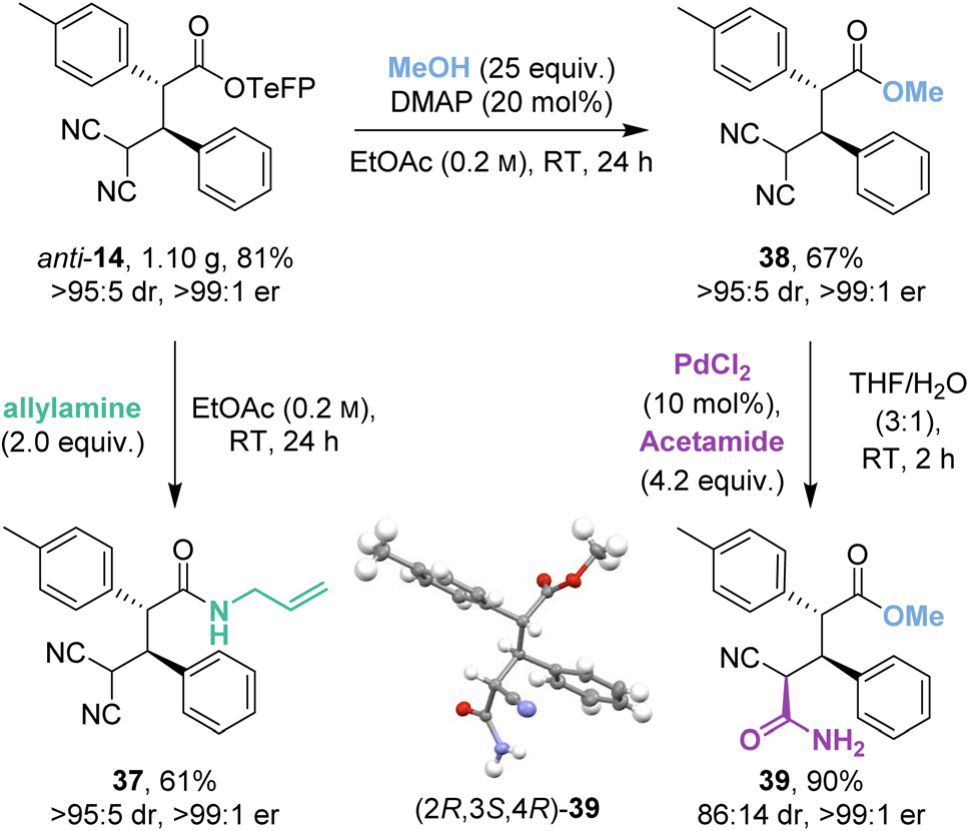
Gram-scale catalytic demonstration and product derivatisations.

The isothiourea-catalysed Michael addition was successfully implemented on gram-scale to give 1.10 g (81%) of *anti*-14 (Scheme 5). Recrystallisation of the crude reaction mixture allowed a chromatography-free preparation of *anti*-14 as a single stereoisomer (>95 : 5 dr, >99 : 1 er). *Anti*-14 was then derivatised to allyl amide 37 and methyl ester 38 in good yields (61% and 67%) and as single stereoisomers (>95 : 5 dr, >99 : 1 er) despite competing retro-Michael addition being observed in both cases. Interestingly, control studies indicated that the derivatised ester and amide products 37 and 38 were stable to retro-Michael addition.

As an alternative derivatisation, desymmetrisation of the geminal dinitriles within 38 by palladium-catalysed hydration gave 39 in excellent yield (90%) with good diastereoselectivity (86 : 14 dr) and without degradation of enantiopurity (>99 : 1 er). The (2*R*,3*S*,4*R*) relative and absolute configuration within the major diastereoisomer 39 was proven by X-ray crystallographic analysis.^18^

### 2.6 Reversible Michael addition control reaction and proposed mechanism

**Scheme 6:**
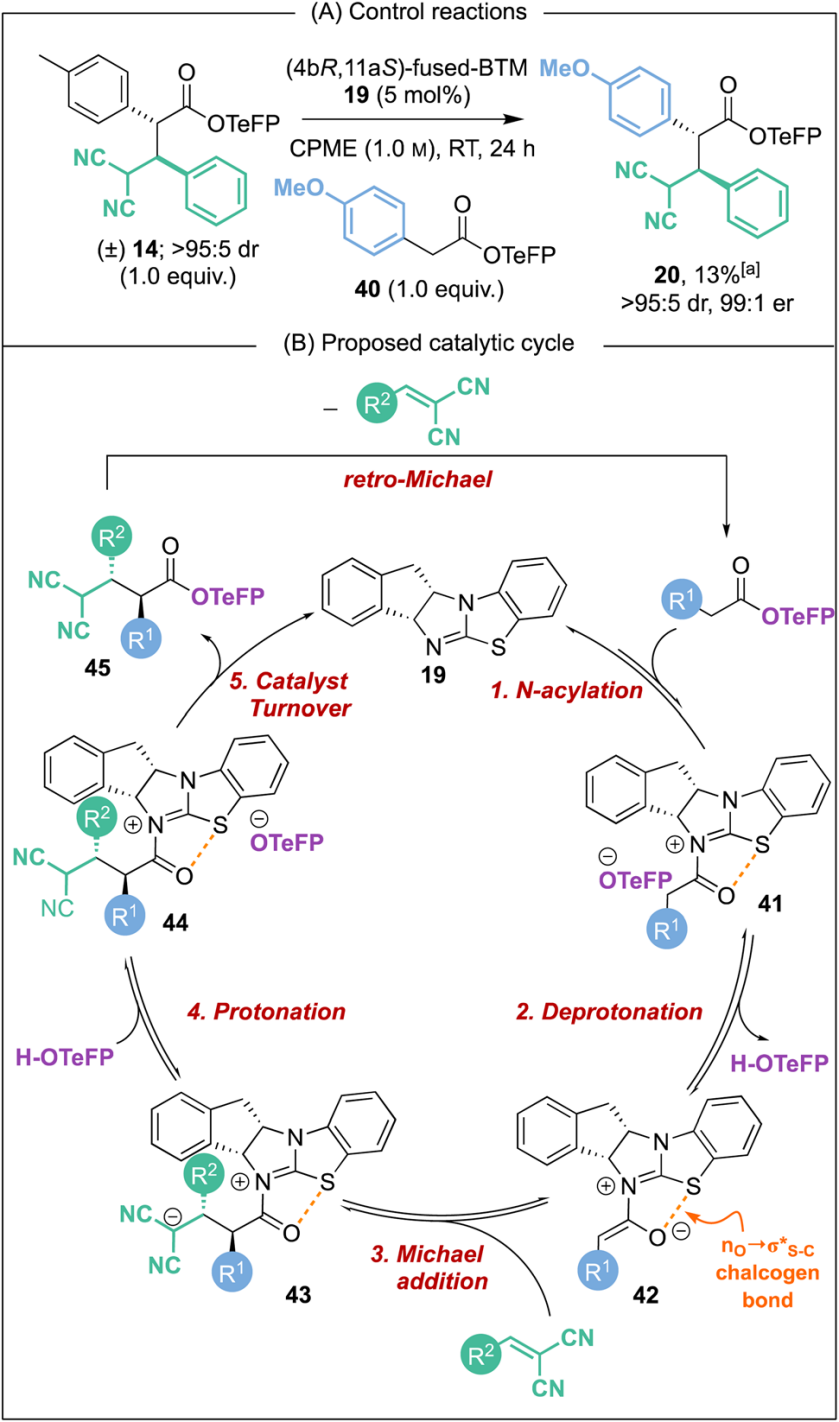
Proposed catalytic cycle. [a] Yield determined by ^1^H NMR analysis of the crude reaction mixture using 1,3,5-trimethoxybenzene internal standard.

Further conclusive evidence of the reversible nature of the Michael addition process was sought. Unambiguous demonstration of the feasibility of this process was observed through treatment of racemic Michael addition product *anti*-14 (>95 : 5 dr) under the standard reaction conditions using the isothiourea (4b*R*,11a*S*)-fused-BTM 19 (5 mol%) in CPME at RT in the presence of 4-MeOC_6_H_4_-substituted TeFP ester 40 (Scheme 6A). After 24 hours, 13% of Michael addition product 20 (>95 : 5 dr, 99 : 1 er) was isolated, indicating that constructive Michael addition of the dinitrile acceptor arising from retro-Michael addition of product 14 was feasible. Based on previous studies^2*e*^ and the observations reported herein, a catalytic cycle for this transformation can be proposed (Scheme 6B). (4b*R*,11a*S*)-Fused-BTM 19 is reversibly acylated by a TeFP ester to form acyl ammonium ion pair 41. Reversible deprotonation by the aryloxide then generates selectively the (*Z*)-C(1)-ammonium enolate 42 which is stabilised by a 1,5-O⋯S chalcogen bonding interaction 
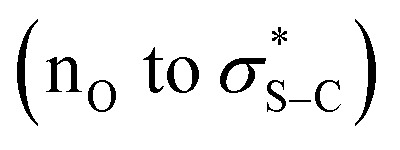
.^19–21^ Michael addition to vinyl dinitrile generates the acyl ammonium intermediate 43 that is subsequently protonated by the 2,3,5,6-tetrafluorophenol to give acyl ammonium ion pair 44. The aryloxide subsequently effects catalyst turnover to afford product 45 with excellent enantioselectivity, with CIDT leading to enhanced diastereoselectivity in specific examples.

Based on this catalytic cycle and control studies, retro-Michael addition could in principle occur from both the acyl ammonium intermediate 43 and the ester product 45 and we currently cannot distinguish unambiguously between both possibilities. Since our studies have demonstrated that aryloxide turnover to give α,α-difunctionalised ester products is irreversible in the presence of an isothiourea,^22^ it seems likely that the isothiourea acts as a Brønsted base to promote retro-Michael addition from product 45.

## 3. Conclusions

In conclusion, the scope and limitations of the base-free enantioselective Michael addition of 2,3,5,6-tetrafluorophenyl esters to 2-benzylidene malononitriles have been demonstrated. Variation of the substitution on both electrophilic and nucleophilic reaction partners was tolerated giving generally good yields and diastereoselectivity with excellent enantioselectivity. Mechanistic investigation determined that retro-Michael addition is promoted by both isothiourea (4b*R*,11a*S*)-fused-BTM 19 and nucleofuge 2,3,5,6-tetrafluorophenoxide. In three examples the reversible nature of the Michael addition was exploited to achieve excellent product diastereoselectivity through a novel CIDT. The reaction can be readily carried out upon a gram scale and derivatised to allow access to a variety of stereodefined products. Further applications of the reversible Michael addition process are currently under investigation in this laboratory.

## Data availability

The research data supporting this publication can be accessed at: A. J. Nimmo, J. Bitai, C. M. Young, A. M. Z. Slawin, D. B. Cordes and A. D. Smith, Data underpinning: “Enantioselective isothiourea-catalysed reversible Michael addition of aryl esters to 2-benzylidene malononitriles”, University of St Andrews Research Portal, 2023, DOI: 10.17630/84e083be-87f4-482e-9f60-c9f08eab44cb.

## Author contributions

AJN carried out all experimental studies in consultation with JB and CMY. CM carried our preliminary work that led to this project. ADS, AJN and CMY wrote the manuscript. AMZS and DBC carried out single crystal X-ray analysis. All authors agreed on the finalised version of the manuscript.

## Conflicts of interest

There are no conflicts to declare.

## Supplementary Material

SC-014-D3SC02101G-s001

SC-014-D3SC02101G-s002
