# Supplementary material for: Enantioselective isothiourea-catalysed reversible Michael addition of aryl esters to 2-benzylidene malononitriles
Source: Chem Sci. 2023 Jun 2;14(27):7537–44. doi: 10.1039/d3sc02101g (PMC10337745; doi:10.1039/d3sc02101g)

Electronic Supplementary Material (ESI) for Chemical Science.
This journal is © The Royal Society of Chemistry 2023

Enantioselective Isothiourea-Catalysed Reversible Michael Addition of Aryl Esters to 2-Benzylidene Malononitriles

Alastair J. Nimmo, Jacqueline Bitai, Claire M. Young, Alexandra M. Z. Slawin, David B. Cordes and Andrew D. Smith*

Supporting Information

Table of Contents

1 General Information ... 3

2 General Procedures ... 5

3 Reaction Optimisation ... 8

4 Unsuccessful Substrates ... 12

5 Mechanistic Studies ... 12

6 Preparation of vinyl dinitriles ... 21

7 Preparation of electron-deficient aryl esters ... 26

8 Ester screen products ... 33

9 Isothiourea catalysis products ... 37

10 Isolation of CIDT products by filtration ... 56

11 Gram scale reaction ... 57

12 Product derivatisations ... 58

13 Single crystal X-ray diffraction data ... 62

14 References ... 66

Appendix I: ^{1}H, $^{19}F\{^{1}H\}$, $^{13}C\{^{1}H\}$, 2D ^{1}H COSY, 2D ^{1}H–^{13}C HSQC and 2D ^{1}H–^{13}C HMBC NMR Spectra ... 67

Appendix II: HPLC traces of novel compounds ... 288

1 General Information

All reagents and solvents were obtained from commercial suppliers and were used without further purification unless otherwise stated. Reactions involving moisture sensitive reagents were carried out in flame-dried glassware under an inert atmosphere (N_2 or Ar) using standard vacuum line techniques. Anhydrous solvents (Et_2O, CH_2Cl_2, THF and toluene) were obtained after passing through an alumina column (Mbraun SPS-800). Petrol is defined as petroleum ether 40–60 °C.

Room temperature (RT) refers to 20–25 °C. Reactions involving heating were performed using DrySyn blocks and a contact thermocouple.

Under reduced pressure refers to the use of either a Büchi Rotavapor R-200 with a Büchi V-491 heating bath and Büchi V-800 vacuum controller, a Büchi Rotavapor R-210 with a Büchi V-491 heating bath and Büchi V-850 vacuum controller, a Heidolph Laborota 4001 with vacuum controller, an IKA RV10 rotary evaporator with a IKA HB10 heating bath and ILMVAC vacuum controller, or an IKA RV10 rotary evaporator with a IKA HB10 heating bath and Vacuubrand CVC3000 vacuum controller. Rotary evaporator condensers are fitted to Julabo FL601 Recirculating Coolers filled with ethylene glycol and set to –5 °C.

Analytical thin layer chromatography (TLC) was performed on pre-coated aluminium plates (Kieselgel 60 F254 silica) and visualisation was achieved using ultraviolet light (254 nm) and/or staining with either aqueous $KMnO_4$ solution, ethanolic phosphomolybdic acid, or ethanolic Vanillin solution followed by heating. Manual column chromatography was performed in glass columns fitted with porosity 3 sintered discs over Kieselgel 60 silica using the solvent system stated.
Melting points were recorded on an Electrothermal 9100 melting point apparatus, (dec) refers to decomposition.

Optical rotations were measured on a Perkin Elmer Precisely/Model-341 polarimeter operating at the sodium D line with a 100 mm path cell at 20 °C. Concentrations (*c*) are stated in g/100 mL.

HPLC analyses were obtained on either a Shimadzu HPLC consisting of a DGU-20A5 degassing unit, LC-20AT liquid chromatography pump, SIL-20AHT autosampler, CMB-20A communications bus module, SPD-M20A diode array detector and a CTO-20A column oven or a Shimadzu HPLC consisting of a DGU-20A5R degassing unit, LC-20AD liquid chromatography pump, SIL-20AHT autosampler, SPD-20A UV/Vis detector and a CTO-20A column oven. Separation was achieved using either a DAICEL CHIRALCEL OD-H column or DAICEL CHIRALPAK AD-H, IA, IB and ID columns using the method stated. HPLC traces of enantiomerically enriched compounds were compared with authentic racemic spectra.

Infrared spectra were recorded on a Shimadzu IRAffinity-1 Fourier transform IR spectrophotometer fitted with a Specac Quest ATR accessory (diamond puck). Spectra were recorded of thin films with characteristic absorption wavenumbers (ν_{max}) reported in cm^{-1}.

^{1}H, $^{13}C\{^{1}H\}$, and $^{19}F\{^{1}H\}$ NMR spectra were acquired on either a Bruker AV400 with a BBFO probe (^{1}H 400 MHz; $^{19}F\{^{1}H\}$ 377 MHz), a Bruker AVII 400 with a BBFO probe (^{1}H 400 MHz; $^{19}F\{^{1}H\}$ 376 MHz), a Bruker AVIII-HD 500 with a SmartProbe BBFO+ probe (^{1}H 500 MHz, $^{19}F\{^{1}H\}$ 470 MHz), a Bruker AVIII 500 with a CryoProbe Prodigy BBO probe (^{1}H 500 MHz, $^{13}C\{^{1}H\}$ 126 MHz), or a Bruker AVIII-HD 700 with a CryoProbe Prodigy TCI probe ($^{13}C\{^{1}H\}$ 176 MHz) in the deuterated solvent stated. All chemical shifts are quoted in parts per million (ppm) relative to the residual solvent peak. All coupling constants, J, are quoted in Hz. Multiplicities are indicated as s (singlet), d (doublet), t (triplet), q (quartet), m (multiplet), and multiples thereof. The abbreviation Ar denotes aromatic. NMR peak assignments were confirmed using 2D ^{1}H correlated spectroscopy (COSY), 2D ^{1}H nuclear Overhauser effect spectroscopy (NOESY), 2D ^{1}H–^{13}C heteronuclear multiple-bond correlation spectroscopy (HMBC), and 2D ^{1}H–^{13}C heteronuclear single quantum coherence (HSQC) where necessary.

Mass spectrometry (*m*/*z*) data were acquired by either electrospray ionisation (ESI), electron impact (EI), or matrix-assisted laser desorption/ionisation with no matrix (MALDI (no matrix)) at either the University of St Andrews Mass Spectrometry Facility or SIRCAMS at University of Edinburgh.

2 General Procedures

2.1 General Procedure A: Preparation of 2-benzylidenemalononitriles

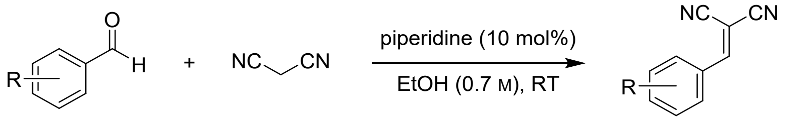

Following the procedure of Guo and co-workers,[1] the requisite aldehyde (1.0 equiv.) was dissolved in EtOH (0.7 M) then malononitrile (1.0 equiv.) followed by piperidine (10 mol%) were added. The reaction mixture was stirred at RT. On completion, pentane:Et_2O (9:1) was added to precipitate the product, which was then collected by filtration under vacuum, washing with pentane:Et_2O (9:1).

2.2 General Procedure B: Preparation of electron-deficient aryl 2-*p*-tolylacetates

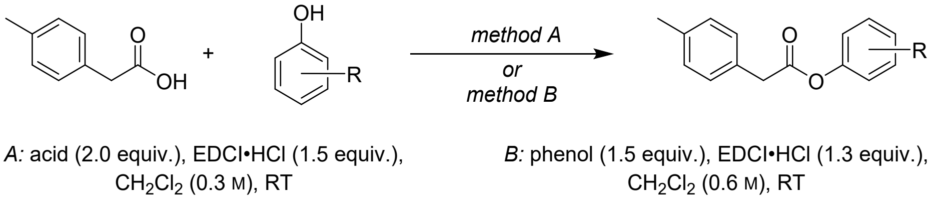

Method A:[2] 2-(*p*-tolyl)acetic acid (2.0 equiv.) and EDCI·HCl (1.5 equiv.) were dissolved in CH_2Cl_2 (0.3 M) with stirring. The appropriate phenol (1.0 equiv.) was added, and the reaction stirred at RT. Upon completion, the reaction mixture was diluted with CH_2Cl_2 and extracted with $NaHCO_3$ (× 2). The organic phase was then dried over $MgSO_4$ and concentrated *in vacuo* to give the crude product which was purified by flash silica column chromatography as specified.

Method B:[3] 2-(*p*-tolyl)acetic acid (1.0 equiv.) and EDCI·HCl (1.3 equiv.) were dissolved in CH_2Cl_2 (0.6 M) with stirring. The appropriate phenol (1.5 equiv.) was added, and the reaction stirred at RT. Upon completion, the reaction mixture was diluted with H_2O and extracted with CH_2Cl_2 (× 3). The organic phase was then dried over $MgSO_4$ and concentrated *in vacuo* to give the crude product which was purified by flash silica column chromatography as specified.

2.3 General Procedure C: Preparation of 2,3,5,6-tetrafluorophenyl esters

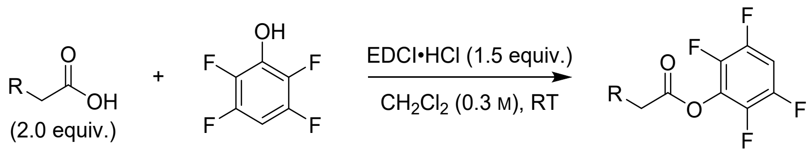

Following the procedure of Smith and co-workers,[2] the appropriate arylacetic acid (2.0 equiv.) and EDCI·HCl (1.5 equiv.) were dissolved in CH_2Cl_2 (0.3 M) with stirring. 2,3,5,6-Tetrafluorophenol (1.0 equiv.) was added, and the reaction stirred at RT. Upon completion, the reaction mixture was diluted with CH_2Cl_2 and extracted with $NaHCO_3$ (× 2). The organic phase was then dried over $MgSO_4$ and concentrated to give the crude product which was purified by flash silica column chromatography as specified.

2.4 General Procedure D: Screening electron-deficient aryl esters

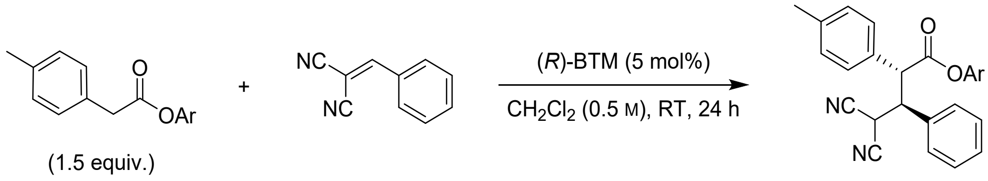

To an oven-dried vial was added the appropriate electron-deficient aryl 2-*p*-tolylacetate (0.75 mmol, 1.5 equiv.), 2-benzylidene malononitrile (78 mg, 0.50 mmol, 1.0 equiv.), and (*R*)-BTM (6.3 mg, 0.025 mmol, 5 mol%). The vial was sealed and purged before CH_2Cl_2 (0.5 M) was added and the reaction stirred at RT for 24 h. The reaction was stopped by removal of the solvent under reduced pressure. The crude product was purified by flash silica column chromatography as specified.

2.5 General Procedure E: Isothiourea-catalysed enantioselective Michael addition

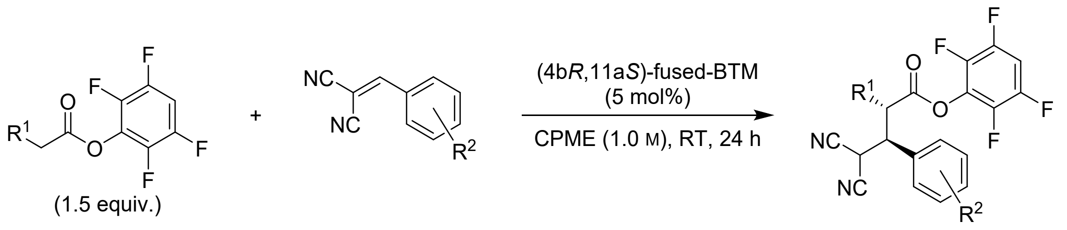

To an oven-dried vial was added the appropriate 2,3,5,6-tetrafluorophenyl ester (0.75 mmol, 1.5 equiv.), vinyl dinitrile (0.50 mmol, 1.0 equiv.), and (4b*R*,11a*S*)-fused-BTM **19** (6.6 mg, 0.025 mmol, 5 mol%). The vial was sealed and purged before CPME (1.0 M) was added and the reaction stirred at RT for 24 h. The reaction was stopped by removal of the solvent under reduced pressure. The crude product was purified by flash silica column chromatography as specified.

3 Reaction Optimisation

All optimisation reactions were performed on a 0.5 mmol scale.

3.1 Screen of electron-deficient aryl esters

Table S1: Influence of electron-deficient aryl ester.

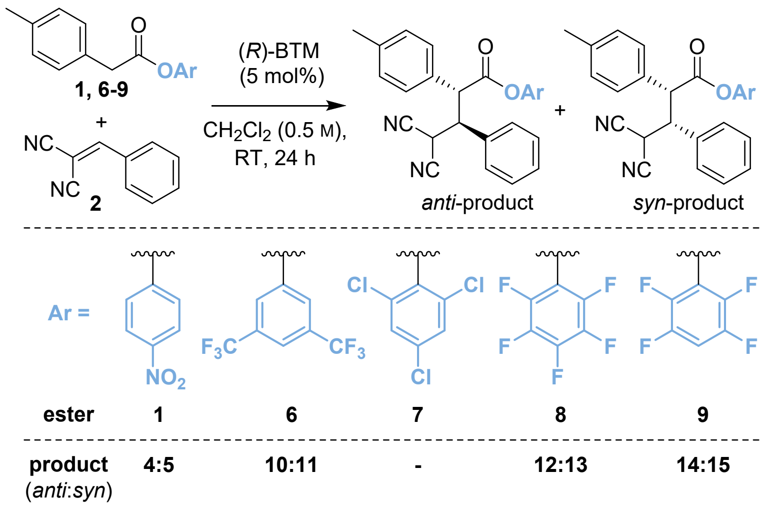

Entry	Ester	dr[a] *anti*:*syn*	er[b] *anti*	er[b] *syn*	Yield[c] (%)
**1**	**1**	49:51	79:21	93:7	58
**2**	**6**	48:52	87:13	97:3	48
**3**	**7**	-	-	-	0
**4**	**8**	74:26	89:11	93:7	48
**5**	**9**	68:32	89:11	95:5	51

[a] Determined by ^{1}H NMR analysis. [b] Determined by chiral HPLC analysis of a mixture of diastereoisomers. [c] Combined isolated yield of diastereoisomers.

3.2 Screen of reaction conditions

Table S2: Optimisation of reaction conditions.

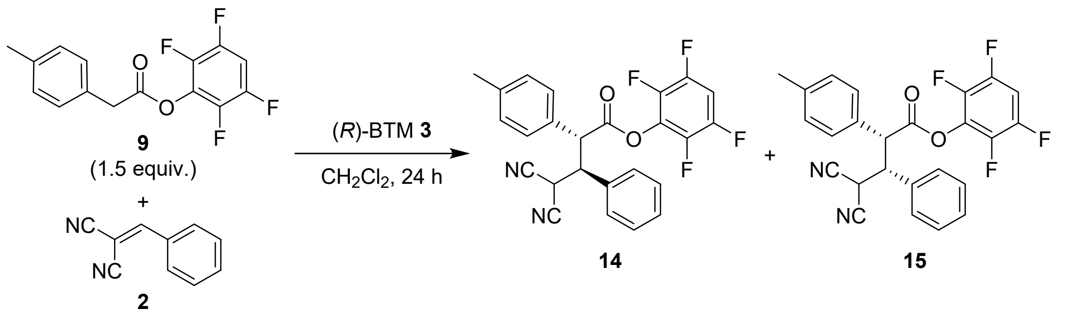

Entry	Catalyst (mol%)	Concentration (M)	Temperature (°C)	dr[a] *anti*:*syn*	er[b] *anti*	er[b] *syn*	Yield[c] (%)
**1**	5	0.5	RT	68:32	89:11	95:5	51
**2**	5	1.0	RT	72:28	87:13	94:6	72
**3**	5	2.0	RT	76:24	89:11	90:10	70
**4**	10	1.0	RT	85:15	85:15	83:17	76
**5**	5	1.0	40	83:17	82:18	82:18	64

[a] Determined by ^1H NMR analysis. [b] Determined by chiral HPLC analysis of a mixture of diastereoisomers. [c] Combined isolated yield of diastereoisomers.

3.3 Screen of solvents

Table S3: Solvent screen.

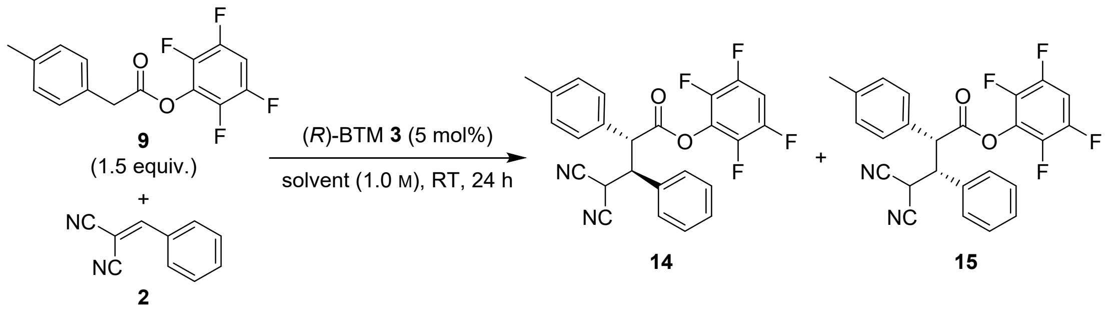

Entry	Solvent	dr[a] *anti*:*syn*	er[b] *anti*	er[b] *syn*	Yield[c] (%)
**1**	CH_2Cl_2	72:28	87:13	94:6	72
**2**	PhMe	74:26	77:23	95:5	67
**3**	THF	52:48	82:18	92:8	69
**4[d]**	Et_2O	75:25	89:11	92:8	80
**5**	1,4-Dioxane	65:35	90:10	95:5	75
**6**	MeOH	-	-	-	0
**7**	MeCN	38:62	47:53	81:19	62
**8**	DMF	26:74	35:65	77:23	23
**9[d]**	EtOAc	68:32	82:18	90:10	78
**10**	$CHCl_3$	77:23	91:9	97:3	54
**11[d]**	TBME	83:17	89:11	90:10	75
**12[d]**	CPME	83:17	90:10	92:8	77
**13**	DME	62:38	78:22	87:13	70
**14**	IPA	69:31	82:18	89:11	70
**15[d]**	DMC	66:34	85:15	94:6	76

[a] Determined by ^1H NMR analysis. [b] Determined by chiral HPLC analysis of a mixture of diastereoisomers. [c] Combined isolated yield of diastereoisomers. [d] Precipitation of products observed.

3.4 Screen of isothiourea catalysts

Table S4: Screening of catalysts with extended reaction time.

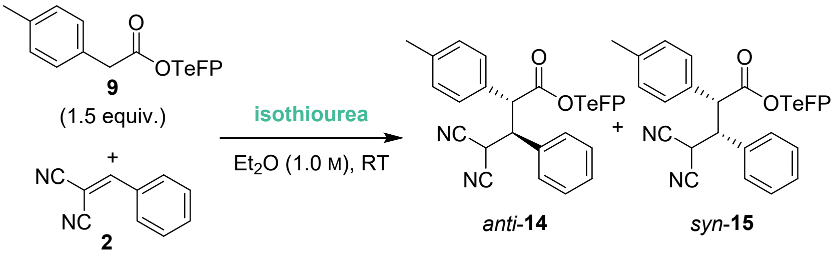

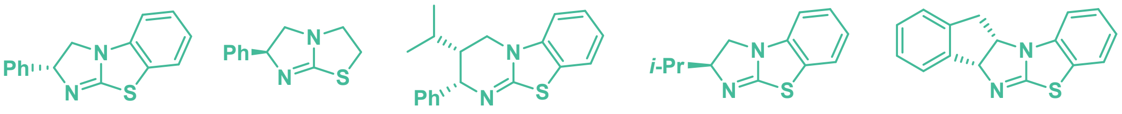

(*R*)-BTM (**3**) (*S*)-TM (**16**) (2*S*,3*R*)-HyperBTM (**17**) (*S*)-*i*-Pr-BTM (**18**) (4b*R*,11a*S*)-fused-BTM (**19**)

Entry	Catalyst (mol%)	Reaction time (h)	dr[a] *anti*:*syn*	er[b] *anti*	er[b] *syn*	Yield[c] (%)
**1**	(*R*)-BTM **3** (5)	48	>95:5	88:12	-	Quant.
**2**	(*R*)-BTM **3** (20)	24	>95:5	87:13	-	86
**3**	(*S*)-TM **16** (20)	168	74:26	87:13[d]	>99:1[d]	45
**4**	(2*S*,3*R*)-HyperBTM **17** (5)	24	>95:5	79:21	-	99
**5**	(*S*)-*i*-Pr-BTM **18** (5)	96	>95:5	98:2[d]	-	99
**6**	(4b*R*,11a*S*)-fused-BTM **19** (5)	24	95:5	99:1	-	91

[a] Determined by ^{1}H NMR analysis. [b] Determined by chiral HPLC analysis of a mixture of diastereoisomers. [c] Combined isolated yield of diastereoisomers. [d] opposite major enantiomer.

4 Unsuccessful Substrates

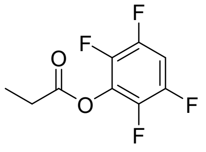

33

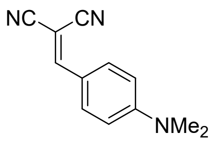

34

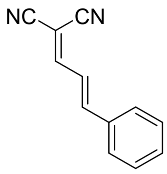

35

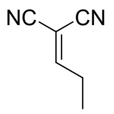

36

2,3,5,6-Tetraflurophenyl ester **33** and vinyl dinitriles **34** and **35** were completely unreactive returning only the starting materials. Vinyl dinitrile **36** reacted poorly giving around 20% conversion in 24 h.

5 Mechanistic Studies

For all reactions the yield was determined by ^{1}H NMR with 1,3,5-trimethoxybenzene as internal standard.

5.1 PNP Ester

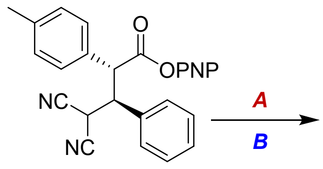

anti-**4**[a]
77:23 er

Conditions	*anti*-**4**[a]	*syn*-**5**[b]	**1** and **2**
A	74%<br>72:28 er	3%<br>73:27 er	23%
B	90%<br>76:24 er	0%	10%

A: (*R*)-BTM **3** (5 mol%), CH_2Cl_2 (0.5 M), RT, 24 h

B: NBu_4OPNP (5 mol%), CH_2Cl_2 (0.5 M), RT, 24 h

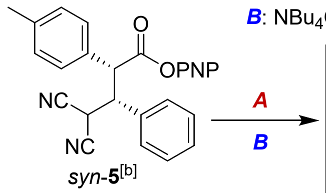

syn-**5**[b]
95:5 er

Conditions	*anti*-**4**[a]	*syn*-**5**[b]	**1** and **2**
A	4%<br>78:22 er	47%<br>87:13 er	49%
B	4%<br>16:84 er	93%<br>99:1 er	3%

A single diastereoisomer of either *anti*-**4** or *syn*-**5** (21.3 mg, 0.05 mmol, 1.0 equiv.) and either (*R*)-BTM (0.6 mg, 0.0025 mmol, 5 mol%) or NBu_4OPNP (1.0 mg, 0.0025 mmol, 5 mol%) were weighed into an oven-dried vial. The vial was capped and purged before addition of CH_2Cl_2 (0.5 M) and the reaction stirred at RT for 24 h.

5.2 TeFP Ester

Tetrabutylammonium 2,3,5,6-tetrafluorophenoxide (S1)

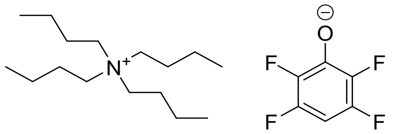

Adapting the procedure of Mayr and co-workers,[4] to a stirred solution of 2,3,5,6-tetrafluorophenol (0.50 g, 3.0 mmol, 1.5 equiv.) in MeOH (0.7 M) was added slowly tetrabutylammonium hydroxide (40% in H_2O) (1.3 mL, 2.0 mmol, 1.0 equiv.). The reaction mixture was stirred at RT for 10 min then the solvent was removed to give the crude product as a colourless solid. The crude product was dissolved in Et_2O then filtered. The filtrate was then concentrated under reduced pressure and triturated with hexane to afford the title compound as a colourless solid (206 mg, 25%). **mp** 47-49 °C; **IR** ν_{max} (film) 3175 (br), 2959 (C-H), 2934, 2874, 1634, 1564, 1504, 1485, 1383, 1271, 1155, 1101, 926, 881; **^{1}H NMR** (400 MHz, MeOD) δ_H: 1.04 (12H, t, *J* 7.3, $NCH_2CH_2CH_2CH_3$), 1.37 – 1.49 (8H, m, $NCH_2CH_2CH_2CH_3$), 1.63 – 1.73 (8H, m, $NCH_2CH_2CH_2CH_3$), 3.21 – 3.29 (8H, m, $NCH_2CH_2CH_2CH_3$), 6.05 (1H, tt, *J* 10.9, 6.9, ArC(4)*H*); ^{19}F{^{1}H} **NMR** (377 MHz, MeOD) δ_F: -168.7 – -168.5 (m), -148.3 – -148.1 (m); **^{13}C{^{1}H} NMR** (126 MHz, MeOD) δ_C: 12.5 ($NCH_2CH_2CH_2CH_3$), 19.3 ($NCH_2CH_2CH_2CH_3$), 23.4 ($NCH_2CH_2CH_2CH_3$), 58.1 ($NCH_2CH_2CH_2CH_3$), 85.6 (t, *J* 24.3, Ar*C*(4)H), 139.7 – 141.9 (m, ArCF), 145.7 – 148.3 (m, ArCF and Ar*C*(1)); **HRMS** (ESI^+) $C_{16}H_{36}N$ $[M]^+$ found 242.2854, requires 242.2842 (+4.8 ppm), (ESI^-) C_6HOF_4 $[M]^-$ found 164.9962, requires 164.9969 (-4.2 ppm).

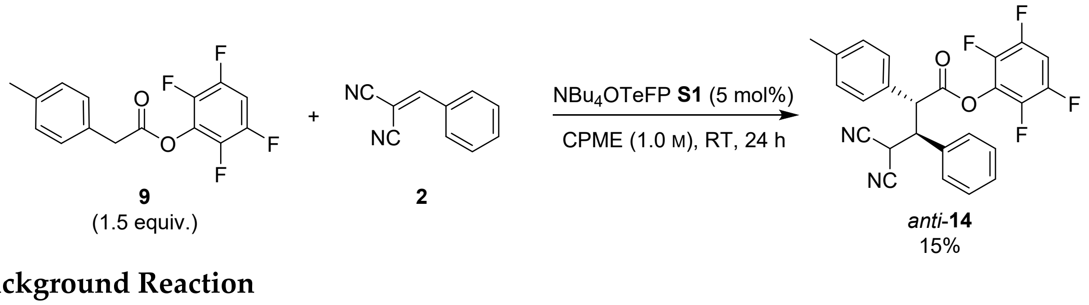

Background Reaction

Adapting General Procedure E by replacing (4b*R*,11a*S*)-fused-BTM **19** with NBu_4OTeFP **S1**, 2,3,5,6-tetrafluorophenyl 2-(p-tolyl)acetate **9** (112 mg, 0.38 mmol, 1.5 equiv.), 2-

benzylidene malononitrile **2** (38.5 mg, 0.25 mmol, 1.0 equiv.), and NBu_4OTeFP **S1** (5.1 mg, 0.013 mmol, 5 mol%) in CPME (1.0 M) gave only *anti*-**14** (15%).

Retro-Michael Reactions

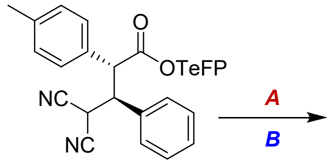

anti-**14**[a]
98:2 er

Conditions	*anti*-**14**[a]	*syn*-**15**[b]	**9** and **2**
A	48%<br>90:10 er	6%<br>93:7 er	46%
B	90%<br>98:2 er	0%	10%

A: (*R*)-BTM **3** (5 mol%), CH_2Cl_2 (0.5 M), RT, 24 h

B: NBu_4OTeFP (5 mol%), CH_2Cl_2 (0.5 M), RT, 24 h

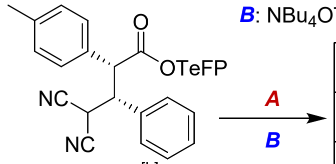

syn-**15**[b]
94:6 er

Conditions	*anti*-**14**[a]	*syn*-**15**[b]	**9** and **2**
A	4%<br>84:16 er	40%<br>83:17 er	56%
B	1%<br>6:94 er	97%<br>95:5 er	2%

A single diastereoisomer of either *anti*-**14** or *syn*-**15** (22.6 mg, 0.05 mmol, 1.0 equiv.) and either (*R*)-BTM **3** (0.6 mg, 0.0025 mmol, 5 mol%) or NBu_4OTeFP **S1** (1.0 mg, 0.0025 mmol, 5 mol%) were weighed into an oven-dried vial. The vial was capped and purged before addition of CH_2Cl_2 (0.5 M) and the reaction stirred at RT for 24 h.

Retro-Michael Reactions in Optimised Conditions

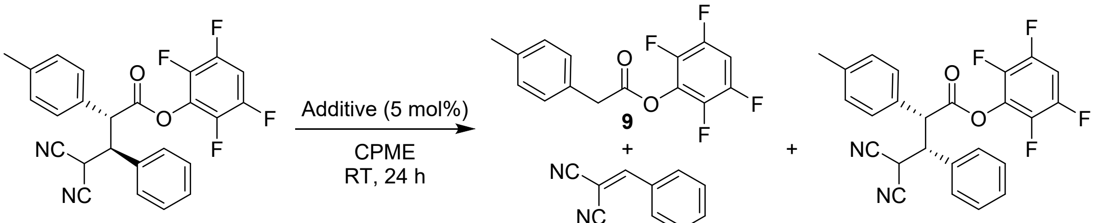

anti-**14**
>99:1 er

syn-**15**

Enantiopure (>99:1 er) *anti*-**14** and either (4b*R*,11a*S*)-fused-BTM **19** or NBu_4OTeFP **S1** were weighed into an oven-dried vial. The vial was sealed and purged before CPME was added and the reaction mixture stirred at RT for 24 h.

In 1.0 M CPME *anti*-**14** was not very soluble, as demonstrated by it precipitating during the catalysis. Under these conditions, without any other additives *anti*-**14** was stable to the retro reaction (Table S5, Entry 1). Adding (4b*R*,11a*S*)-fused-BTM **19** promoted the retro-reaction, but with only slight (6%) conversion to starting materials **9** and **2** (Table S5,

Entry 2). Similarly, adding NBu_4OTeFP **S1** gave 4% starting materials **9** and **2**, with most of *anti*-**14** intact (Table S5, Entry 3).

Table S5: Retro-Michael reaction in CPME (1.0 M)

Entry	(4b*R*,11a*S*)-19 (5 mol%)	NBu_4OTeFP (5 mol%)	*Anti*-14 (%)	*Syn*-15 (%)	9 + 2 (%)
**1**	No	No	100	0	0
**2**	Yes	No	94	0	6
**3**	No	Yes	96	0	4

To account for the relative concentration of *anti*-**14** as it is being formed during the catalysis, the control reactions were also carried out in 0.05 M CPME. Again, without any additives *anti*-**14** was stable to retro reaction (Table S6, Entry 1). Adding (4b*R*,11a*S*)-fused-BTM **19** promoted the retro reaction giving 33% starting materials **9** and **2**. Also, (4b*R*,11a*S*)-fused-BTM **19** catalysed the forward reaction producing 9% *syn*-**15**. The major (2*R*,3*R*) enantiomer of the *syn*-**15** is consistent with the catalysis and not from epimerisation (Table S6, Entry 2). Adding NBu_4OTeFP **S1** gave 24% starting materials **9** and **2**, but no epimerisation to *syn*-**15** was observed (Table S6, Entry 3).

Table S6: Retro-Michael reaction in CPME (0.05 M)

Entry	(4b*R*,11a*S*)-19 (5 mol%)	NBu_4OTeFP (5 mol%)	*Anti*-14 (%)	*Syn*-15 (%)	9 + 2 (%)
**1**	No	No	100	0	0
**2**	Yes	No	58 (99:1 er)	9 (96:4 er)	33
**3**	No	Yes	76	0	24

In conclusion, both (4b*R*,11a*S*)-fused-BTM **19** and NBu_4OTeFP **S1** promoted the retro-Michael reaction. Precipitation of *anti*-**14** from the reaction mixture was beneficial to minimise the retro-Michael reaction.

5.3 ¹H NMR Reaction Monitoring

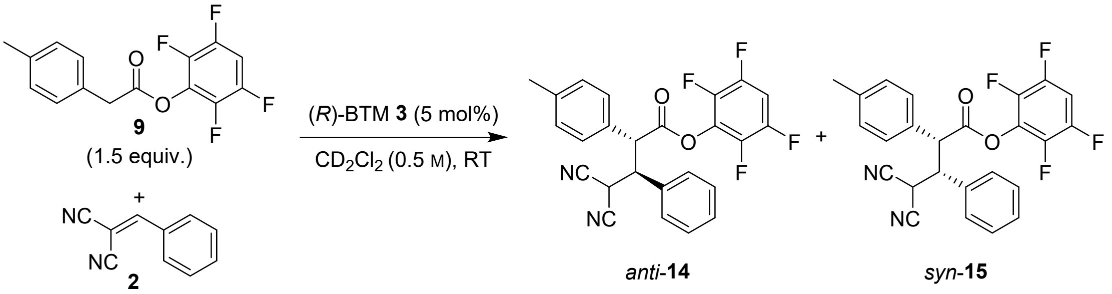

2,3,5,6-tetrafluorophenyl 2-(p-tolyl)acetate **9** (134 mg, 0.45 mmol, 1.5 eq), 2-benzylidene malononitrile **2** (46.3 mg, 0.30 mmol, 1.0 eq), (*R*)-BTM **3** (3.8 mg, 0.015 mmol, 5 mol%) and 1,3,5-trimethoxybenzene (16.8 mg, 0.10 mmol, 0.33 eq) were dissolved in CD_2Cl_2 then transferred into an NMR tube. A ^{1}H NMR spectrum was obtained 10 times over the course of 48 h.

Figure S1: Diastereomeric excess over time.

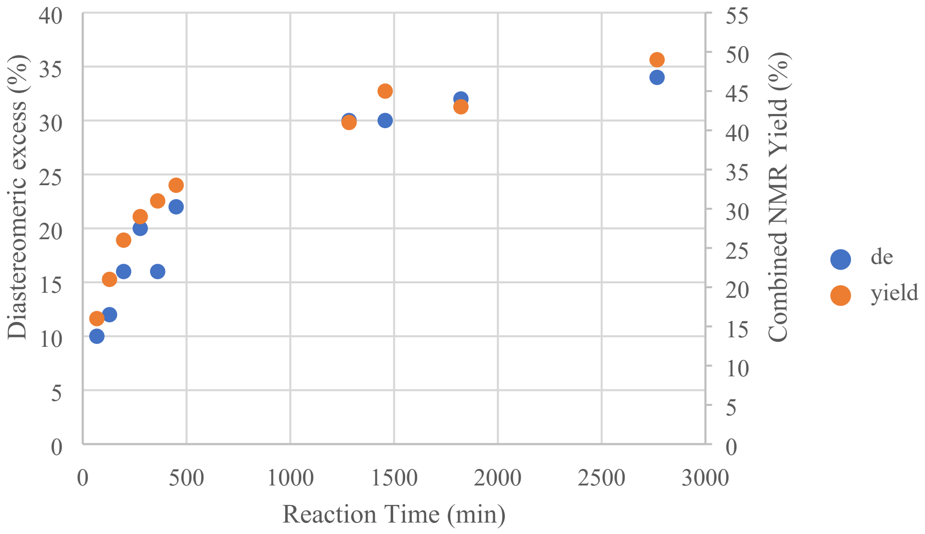

5.4 Probing Transesterification of Catalysis Product

***anti*-2,3,5,6-tetrafluorophenyl 3-phenyl-4,4-bis(phenylsulfonyl)-2-(p-tolyl)butanoate (S2) and *syn*-2,3,5,6-tetrafluorophenyl 3-phenyl-4,4-bis(phenylsulfonyl)-2-(p-tolyl)butanoate (S3)**

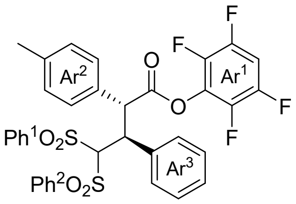

anti-**S2**

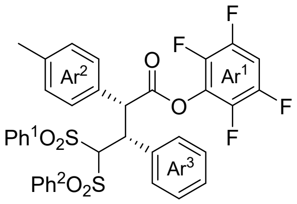

syn-**S3**

2,3,5,6-tetrafluorophenyl 2-(p-tolyl)acetate **9** (224 mg, 0.75 mmol, 1.5 equiv.), (2-phenylethene-1,1-diyldisulfonyl)dibenzene (192 mg, 0.50 mmol, 1.0 equiv.), and (±)-BTM **3** (12.6 mg, 0.05 mmol, 10 mol%) were dissolved in anhydrous CH_2Cl_2 (0.5 M) and the reaction mixture stirred at RT for 24 h. Once complete, the solvent was removed under reduced pressure to give the crude product that was purified by flash silica column chromatography (CH_2Cl_2:hexane 75:25, R_F 0.27) to give the combined *anti* and *syn* diastereoisomers (71:29 dr) (95 mg, 28%) as an inseparable mixture as a colourless solid. **mp** 78–80 °C; **IR** ν_{max} (film) 3069, 1778 (C=O, ester), 1524, 1485, 1449, 1331, 1312, 1159, 1146, 1105, 957, 907, 800; **HRMS** (ESI^+) $C_{35}H_{26}O_6S_2F_4Na$ $[M+Na]^+$ found 705.0981, requires 705.0999 (+2.6 ppm).

Data for *anti*-**S2**: **^{1}H NMR** (500 MHz, $CDCl_3$) δ_H: 2.49 (3H, s, C*H*$_3$), 4.81 (1H, d, *J* 1.5, C(4)*H*), 4.97 (1H, dd, *J* 12.4, 1.5, C(3)*H*), 5.49 (1H, d, *J* 12.4, C(2)*H*), 6.90 (1H, tt, *J* 9.8, 6.8, Ar^{1}C(4)*H*), 7.21 – 7.34 (8H, m, Ar^{2}C(2,3,5,6)*H* and Ph^{1}C(2,6)*H* and Ph^{2}C(2,6)*H*), 7.34 – 7.63 (9H, m, Ar^{3}C(3,4,5)*H* and Ph^{1}C(3,4,5)*H* and Ph^{2}C(3,4,5)*H*), 7.86 – 7.90 (2H, m, Ar^{3}C(2,6)*H*); **^{19}F{^{1}H} NMR** (470 MHz, $CDCl_3$) δ_F: -152.6 – -152.4 (m), -139.1 – -139.0 (m); **^{13}C{^{1}H} NMR** (126 MHz, $CDCl_3$) δ_C: 21.3 (CH_3), 46.1 (*C*(3)H), 54.3 (*C*(2)H), 82.4 (*C*(4)H), 103.3 (t, *J* 22.9, Ar^{1}C(4)H), 128.3 (Ph^2*C*(2,6)H), 128.4 (Ph^1*C*(2,6)H), 128.6 (Ar^{3}C(4)H), 128.7 (Ph^2*C*(3,5)H), 128.9 (Ar^{3}C(3,5)H), 129.0 (Ph^{1}C(3,5)H), 129.1 – 129.4 (m, Ar^{1}C(1)), 129.5 (Ar^{2}C(2,6)H), 130.4 – 130.7 (m, Ar^{2}C(3,5)H and Ar^{3}C(2,6)H), 131.6 (Ar^{2}C(1)), 133.6 (Ar^{3}C(1)), 134.0 (Ph^{2}C(4)H), 134.0 (Ph^1*C*(4)H), 138.6 (Ph^2*C*(1)), 139.4 (Ar^2*C*(4)), 140.1 (Ph^1*C*(1)), 139.2 – 141.5 (m, Ar^{1}CF), 144.6 – 146.9 (m, Ar^{1}CF), 168.1 (*C*(1)).

Data for *syn*-**S3**: ^{1}**H NMR** (500 MHz, $CDCl_3$) (*selected*) δ_H: 2.26 (3H, s, CH_3), 4.78 (1H, dd, *J* 11.9, 3.1, C(3)*H*), 5.45 (1H, d, *J* 11.9, C(2)*H*), 5.86 (1H, d, *J* 3.1, C(4)*H*), 6.98 – 7.10 (5H, m, Ar^{1}C(4)*H* and Ar^{2}C(2,3,5,6)*H*), 7.76 – 7.81 (2H, m, Ar^{3}C(2,6)*H*); ^{19}**F{^{1}H} NMR** (470 MHz, $CDCl_3$) δ_F: -152.5 – -152.3 (m), -138.7 – -138.5 (m); ^{13}**C{^{1}H} NMR** (126 MHz, $CDCl_3$) (*selected*) δ_C: 21.1 (CH_3), 48.0 (*C*(3)H), 54.3 (*C*(2)H), 83.1 (*C*(4)H), 103.6 (t, *J* 22.8, Ar^1*C*(4)H), 128.8 (Ar^{3}C(3,5)H), 129.0 (Ph^{1}C(3,5)H), 129.5 (Ar^{2}C(3,5)H), 133.1 (Ar^{3}C(1)), 134.2 (Ph^{2}C(4)H), 134.2 (Ph^1*C*(4)H), 137.9 (Ar^2*C*(4)), 138.6 (Ph^2*C*(1)), 139.4 – 141.7 (m, Ar^{1}CF), 140.6 (Ph^1*C*(1)), 144.8 – 147.2 (m, Ar^{1}CF), 170.7 (*C*(1)).

anti-4-nitrophenyl 3-phenyl-4,4-bis(phenylsulfonyl)-2-(p-tolyl)butanoate (S4)

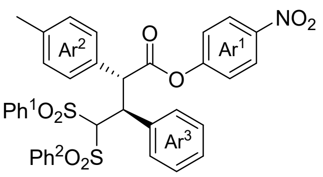

4-nitrophenyl 2-(p-tolyl)acetate **1** (203 mg, 0.75 mmol, 1.5 equiv.), (2-phenylethene-1,1-diyldisulfonyl)dibenzene (192 mg, 0.50 mmol, 1.0 equiv.), and (±)-BTM **3** (12.6 mg, 0.05 mmol, 10 mol%) were dissolved in anhydrous CH_2Cl_2 (0.5 M) and the reaction mixture stirred at RT for 24 h. Once complete, the solvent was removed under reduced pressure to give the crude product (89:11 dr) that was purified by flash silica column chromatography (hexane:EtOAc 85:15 to 50:50, R_F 0.11 at 75:25) to give the title compound (134 mg, 41%) as a colourless solid. **mp** 167–169 °C; **IR** ν_{max} (film) 3063, 1761 (C=O, ester), 1593, 1526, 1447, 1346, 1202, 1159, 1148, 1121, 1080, 860; ^{1}**H NMR** (400 MHz, CD_2Cl_2) δ_H: 2.50 (3H, s, CH_3), 4.74 (1H, d, *J* 1.7, C(4)*H*), 4.81 (1H, dd, *J* 12.4, 1.7, C(3)*H*), 5.38 (1H, d, *J* 12.4, C(2)*H*), 6.67 – 6.73 (2H, m, Ar^{1}C(2,6)*H*), 7.19 – 7.24 (2H, m, Ph^{2}C(2,6)*H*), 7.30 – 7.41 (6H, m, Ar^{2}C(3,5)*H* and Ph^{1}C(3,5)*H* and Ph^{2}C(3,5)*H*), 7.43 – 7.53 (7H, m, Ar^{2}C(2,6)*H* and Ar^{3}C(3,4,5)*H* and Ph^{1}C(2,6)*H*), 7.55 – 7.63 (2H, m, Ph^{1}C(4)*H* and Ph^{2}C(4)*H*), 7.93 – 7.98 (2H, m, Ar^{3}C(2,6)*H*), 8.09 – 8.15 (2H, m, Ar^{1}C(3,5)*H*); ^{13}**C{^{1}H} NMR** (126 MHz, CD_2Cl_2) δ_C: 21.0 (CH_3), 47.2 (*C*(3)H), 54.9 (*C*(2)H), 81.6 (*C*(4)H), 122.1 (Ar^1*C*(2,6)H), 125.0 (Ar^1*C*(3,5)H), 128.1 (Ph^2*C*(2,6)H), 128.2 (Ph^1*C*(2,6)H), 128.5 (Ar^{3}C(3,5)H), 128.6 (Ar^{3}C(4)H), 128.9

(Ph[2]*C*(3,5)H), 129.3 (Ar[2]*C*(2,6)H and Ph[1]*C*(3,5)H), 130.5 (Ar[2]*C*(3,5)H), 131.2 (Ar[3]*C*(2,6)H), 131.7 (Ar[2]*C*(1)), 134.2 (Ph[2]*C*(4)H), 134.2 (Ph[1]*C*(4)H), 134.4 (Ar[3]*C*(1)), 138.3 (Ph[2]*C*(1)), 139.5 (Ar[2]*C*(4)), 140.5 (Ph[1]*C*(1)), 145.4 (Ar[1]*C*(4)), 155.0 (Ar[1]*C*(1)), 169.9 (*C*(1)). **HRMS** (ESI^-) $C_{35}H_{28}O_8S_2N$ $[M-H]^-$ found 654.1247, requires 654.1262 (-2.3 ppm).

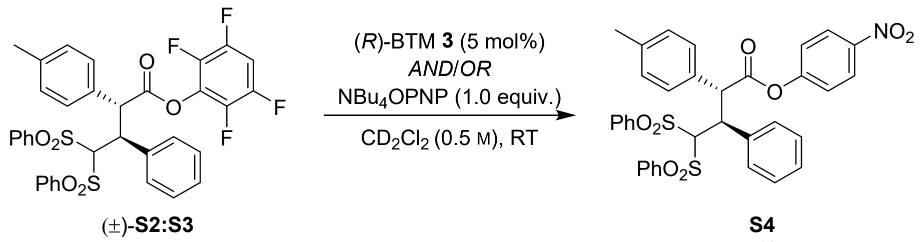

Transesterification Procedure

2,3,5,6-tetrafluorophenyl 3-phenyl-4,4-bis(phenylsulfonyl)-2-(p-tolyl)butanoate **S2:S3** (71:29 dr, 13.7 mg, 0.02 mmol, 1.0 equiv.), 1,3,5-trimethoxybenzene (3.4 mg, 0.02 mmol, 1.0 equiv.), and (*R*)-BTM **3** (0.25 mg, 0.001 mmol, 5 mol%) *AND*/*OR* NBu_4OPNP (7.6 mg, 0.02 mmol, 1.0 equiv.) were dissolved in CD_2Cl_2 then transferred into an NMR tube. A ^1H NMR spectrum was obtained 9 times over the course of 24 h.

No retro-Michael products were observed in any of the reactions. The reaction with only NBu_4OPNP added showed there was no transesterification in the absence of (*R*)-BTM **3**. There was also no transesterification observed when both NBu_4OPNP and (*R*)-BTM **3** were added. These results suggest the final step in the catalysis (catalyst turnover) is irreversible.

5.5 Crossover Through Retro-Michael Reaction

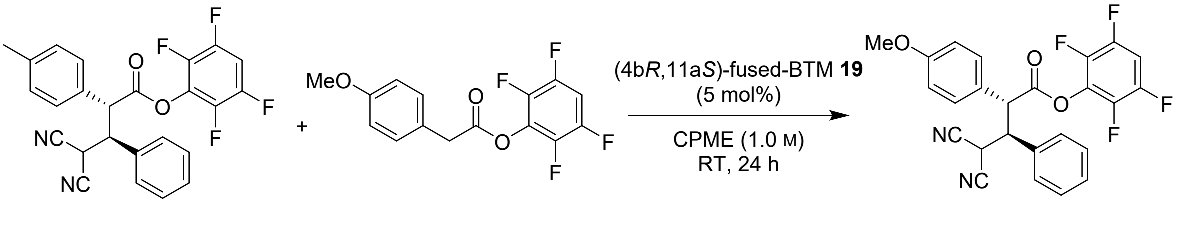

(±)-**14** + **40** → **20**, 13%, 99:1 er

(±)-*anti*-2,3,5,6-tetrafluorophenyl 4,4-dicyano-3-phenyl-2-(*p*-tolyl)butanoate **14** (45.2 mg, 0.1 mmol, 1.0 equiv.), 2,3,5,6-tetrafluorophenyl 2-(4-methoxyphenyl)acetate **40** (31.4 mg, 0.1 mmol, 1.0 equiv.), and (4b*R*,11a*S*)-fused-BTM **19** (1.3 mg, 0.005 mmol, 5 mol%) were dissolved in CPME (1.0 M) and stirred at RT for 24 h. Once complete the solvent was removed under reduced pressure and ^{1}H NMR analysis showed 13% yield of (2*R*,3*S*)-2,3,5,6-tetrafluorophenyl 4,4-dicyano-2-(4-methoxyphenyl)-3-phenylbutanoate **20**. The er was determined to be 99:1 by HPLC analysis on a chiral stationary phase after purification by flash silica column chromatography. For HPLC and chromatography conditions see section 9.

6 Preparation of vinyl dinitriles

2-(4-Fluorobenzylidene)malononitrile (S5)

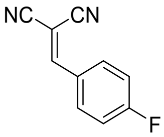

Following General Procedure A, 4-fluorobenzaldehyde (2.48 g, 2.15 mL, 20 mmol), malononitrile (1.32 g, 20 mmol) and piperidine (0.17 g, 0.20 mL, 2 mmol) in EtOH (30 mL) gave the title compound as yellow crystals (2.58 g, 75%). **mp** 118-120 °C {lit[5] 125-126 °C}; **IR** ν_{max} (film) 3038 (C-H), 2230 (C≡N, nitrile), 1599, 1576, 1508, 1416, 1304, 1240, 1161, 1109, 837; **^{1}H NMR** (400 MHz, $CDCl_3$) δ_H: 7.22 – 7.31 (2H, m, ArC(3,5)*H*), 7.77 (1H, s, C(2)*H*), 7.94 – 8.03 (2H, m, ArC(2,6)*H*). **^{19}F{^{1}H} NMR** (376 MHz, $CDCl_3$) δ_F: -100.0 (s); **^{13}C{^{1}H} NMR** (126 MHz, $CDCl_3$) δ_C: 82.4 (d, $^{6}J_{CF}$ 2.7, *C*(1)), 112.5 (CN), 113.6 (CN), 117.2 (d, $^{2}J_{CF}$ 22.5, Ar*C*(3,5)H), 127.4 (d, $^{4}J_{CF}$ 3.4, Ar*C*(1)), 133.5 (d, $^{3}J_{CF}$ 9.5, Ar*C*(2,6)H), 158.4 (*C*(2)H), 166.1 (d, $^{1}J_{CF}$ 260.3, Ar*C*(4)); **HRMS** (EI^{+}) $C_{10}H_5N_2F$ $[M]^{+}$ found 172.0436, requires 172.0431 (+2.9 ppm).

2-(4-Methoxybenzylidene)malononitrile (S6)

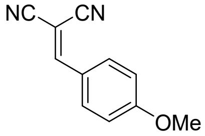

Following General Procedure A, 4-methoxybenzaldehyde (1.36 g, 1.22 mL, 10 mmol), malononitrile (0.66 g, 10 mmol) and piperidine (0.085 g, 0.10 mL, 1 mmol) in EtOH (15 mL) gave the title compound as yellow crystals (1.58 g, 86%). **mp** 110-112 °C {lit[6] 103-107 °C}; **IR** ν_{max} (film) 2220 (C≡N, nitrile), 1605, 1570, 1557, 1512, 1369, 1277, 1180, 1153, 1020, 833; **^{1}H NMR** (400 MHz, $CDCl_3$) δ_H: 3.94 (3H, s, C*H*$_3$), 7.00 – 7.08 (2H, m, ArC(3,5)*H*), 7.68 (1H, s, C(2)*H*), 7.90 – 7.98 (2H, m, ArC(2,6)*H*). **^{13}C{^{1}H} NMR** (126 MHz, $CDCl_3$) δ_C: 55.9 (OCH_3), 78.5 (*C*(1)), 113.4 (CN), 114.5 (CN), 115.2 (Ar*C*(3,5)H), 124.0 (Ar*C*(1)), 133.5 (Ar*C*(2,6)H), 158.9 (*C*(2)H), 164.8 (Ar*C*(4)); **HRMS** (EI^{+}) $C_{11}H_8N_2O$ $[M]^{+}$ found 184.0632, requires 184.0631 (+0.5 ppm).

2-(4-Nitrobenzylidene)malononitrile (S7)

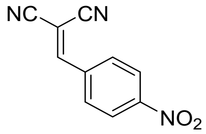

Following General Procedure A, 4-nitrobenzaldehyde (1.51 g, 10 mmol), malononitrile (0.66 g, 10 mmol) and piperidine (0.085 g, 0.10 mL, 1.0 mmol) in EtOH (15 mL) gave the title compound as brown crystals (1.43 g, 72%). **mp** 156-158 °C {lit[7] 160-162 °C}; **IR** ν_{max} (film) 3117 (C-H), 2232 (C≡N, nitrile), 1580, 1522, 1344, 1321, 1304, 1213, 935, 851; **^{1}H NMR** (400 MHz, $CDCl_3$) δ_H: 7.91 (1H, s, C(2)*H*), 8.07 – 8.14 (2H, m, ArC(2,6)*H*), 8.38 – 8.45 (2H, m, ArC(3,5)*H*). **^{13}C{^{1}H} NMR** (126 MHz, $CDCl_3$) δ_C: 87.5 (*C*(1)), 111.6 (*C*N), 112.6 (*C*N), 124.7 (Ar*C*(2,6)H), 131.3 (Ar*C*(3,5)H), 135.8 (Ar*C*(1)), 150.4 (Ar*C*(4)), 156.9 (*C*(2)H); **HRMS** (ESI^-) $C_{10}H_6N_3O_2$ $[M+H]^-$ found 200.0467, requires 200.0466 (+0.5 ppm).

2-(4-Chlorobenzylidene)malononitrile (S8)

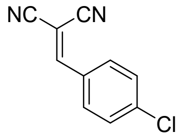

Following General Procedure A, 4-chlorobenzaldehyde (0.70 g, 5.0 mmol), malononitrile (0.33 g, 5.0 mmol) and piperidine (0.043 g, 0.05 mL, 0.50 mmol) in EtOH (10 mL) gave the title compound as white crystals (0.75 g, 80%). **mp** 154-156 °C; **IR** ν_{max} (film) 3034 (C-H), 2228 (C≡N, nitrile), 1585, 1557, 1491, 1406, 1294, 1221, 1096, 935, 829, 814; **^{1}H NMR** (400 MHz, $CDCl_3$) δ_H: 7.51 – 7.59 (2H, m, ArC(3,5)*H*), 7.76 (1H, s, C(2)*H*), 7.84 – 7.92 (2H, m, ArC(2,6)*H*); **^{13}C{^{1}H} NMR** (126 MHz, $CDCl_3$) δ_C: 83.3 (*C*(1)), 112.4 (*C*N), 113.5 (*C*N), 129.3 (Ar*C*(1)), 130.1 (Ar*C*(2,6)H), 131.9 (Ar*C*(3,5)H), 141.2 (Ar*C*(4)), 158.3 (C(2)H); **HRMS** (EI^+) $C_{10}H_5N_2{}^{35}Cl$ $[M]^+$ found 188.0134, requires 188.0136 (-1.1 ppm).

2-(4-(Trifluoromethyl)benzylidene)malononitrile (S9)

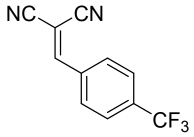

Following General Procedure A, 4-(trifluoromethyl)benzaldehyde (0.87 g, 5.0 mmol), malononitrile (0.33 g, 5.0 mmol) and piperidine (0.043 g, 0.05 mL, 0.5 mmol) in EtOH (10 mL) gave the title compound as white crystals (0.19 g, 17%). The filtrate was concentrated under reduced pressure to give crude product which was purified by flash silica column chromatography (Petrol:CH_2Cl_2 3:1 to 1:1, R_f 0.18 (2:1)) to give the title compound as a colourless solid (0.18 g, 16%). The products were combined to give a total yield of 0.37 g, 33%. **mp** 100-102 °C {lit[7] 107-109 °C }; **IR** ν_{max} (film) 3034 (C-H), 2236 (C≡N, nitrile), 1591, 1566, 1418, 1319, 1167, 1126, 1117, 1070, 1015, 945, 849, 837; **^{1}H NMR** (400 MHz, $CDCl_3$) δ_H: 7.80 – 7.85 (2H, m, ArC(2,6)*H*), 7.87 (1H, s, C*H*), 8.01 – 8.08 (2H, m, ArC(3,5)*H*). **^{19}F{^{1}H} NMR** (376 MHz, $CDCl_3$) δ_F: -63.5 (s); **^{13}C{^{1}H} NMR** (126 MHz, $CDCl_3$) δ_C: 86.0 (*C*(1)), 111.9 (*C*N), 113.0 (*C*N), 123.1 (q, $^1J_{CF}$ 273.0, *C*F$_3$), 126.6 (q, $^3J_{CF}$ 3.8, Ar*C*(3,5)H), 130.8 (Ar*C*(2,6)H), 133.7 (Ar*C*(1)), 135.3 (q, $^2J_{CF}$ 33.3, Ar*C*(4)), 158.1 (*C*(2)H); **HRMS** (ESI^-) $C_{11}H_6N_2F_3$ $[M+H]^-$ found 223.0491, requires 223.0489 (+0.9 ppm).

2-(3-(Trifluoromethyl)benzylidene)malononitrile (S10)

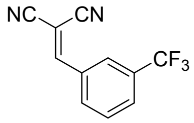

3-(Trifluoromethyl)benzaldehyde (1.34 mL, 10.0 mmol, 1.0 equiv.) was dissolved in EtOH (20 mL) with stirring. Malononitrile (0.66 g, 10.0 mmol, 1.0 equiv.) then piperidine (0.10 mL, 1.0 mmol, 0.1 equiv.) were added and the reaction mixture stirred at RT for 4.5 h. Once complete, the solvent was removed under reduced pressure. The crude product was purified by flash silica column chromatography (Hexane:EtOAc 4:1, R_f 0.44) to give the title compound as a colourless solid (1.58 g, 71%). **mp** 75-77 °C; **IR** ν_{max} (film) 2230 (C≡N, nitrile), 1595, 1325, 1294, 1165, 1123, 1074, 808; **^{1}H NMR** (500 MHz, $CDCl_3$) δ_H: 7.71 – 7.77 (1H, m, ArC(5)*H*), 7.86 – 7.93 (2H, m, C(2)*H* and ArC(4)*H*), 8.09 (1H, s, ArC(2)*H*), 8.18 – 8.24 (1H, m, ArC(6)*H*); **^{19}F{^{1}H} NMR** (376 MHz, $CDCl_3$) δ_F: -63.2 (s); **^{13}C{^{1}H} NMR** (126

MHz, $CDCl_3$) δ_C: 85.3 (*C*(1)), 112.0 (*C*N), 113.1 (*C*N), 123.2 (q, $^1J_{CF}$ 272.8, *C*F_3), 127.7 (q, $^3J_{CF}$ 3.9, Ar*C*(2)H), 130.4 (Ar*C*(5)H), 130.7 (q, $^3J_{CF}$ 3.6, Ar*C*(4)H), 131.4 (Ar*C*(1)), 132.2 (q, $^2J_{CF}$ 33.3, Ar*C*(3)), 132.9 (Ar*C*(6)H), 158.2 (*C*(2)H); **HRMS** ($MALDI\ (no\ matrix)^+$) $C_{11}H_5N_2F_3Na$ $[M + Na]^+$ found 245.0290, requires 245.0297 (-2.9 ppm).

2-(2-(Trifluoromethyl)benzylidene)malononitrile (S11)

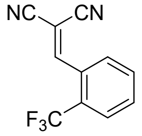

2-(Trifluoromethyl)benzaldehyde (1.32 mL, 10.0 mmol, 1.0 equiv.) was dissolved in EtOH (20 mL) with stirring. Malononitrile (0.66 g, 10.0 mmol, 1.0 equiv.) then piperidine (0.10 mL, 1.0 mmol, 0.1 equiv.) were added and the reaction mixture stirred at RT for 4.5 h. Once complete, the solvent was removed under reduced pressure. The crude product was purified by flash silica column chromatography (Hexane:EtOAc 4:1, R_f 0.42) to give the title compound as a colourless oil (2.00 g, 90%). **IR** ν_{max} (film) 2234 (C≡N, nitrile), 1572, 1315, 1304, 1177, 1109, 1061, 1036, 766; **^{1}H NMR** (400 MHz, $CDCl_3$) δ_H: 7.71 – 7.81 (2H, m, ArC(4,5)*H*), 7.84 – 7.90 (1H, m, ArC(3)*H*), 8.06 – 8.13 (1H, m, ArC(6)*H*), 8.25 (1H, q, $^5J_{HF}$ 1.9, C(2)*H*); **^{19}F{^{1}H} NMR** (376 MHz, $CDCl_3$) δ_F: -58.8 (s); **^{13}C{^{1}H} NMR** (126 MHz, $CDCl_3$) δ_C: 88.4 (*C*(1)), 111.4 (*C*N), 112.6 (*C*N), 123.2 (q, $^1J_{CF}$ 274.1, *C*F_3), 127.0 (q, $^3J_{CF}$ 5.4, Ar*C*(3)H), 128.6 (Ar*C*(1)), 129.6 (q, $^2J_{CF}$ 31.1, Ar*C*(2)), 129.8 (Ar*C*(6)H), 132.8 (Ar*C*(5)H), 133.1 (Ar*C*(4)H), 156.7 (*C*(2)H); **HRMS** ($MALDI\ (no\ matrix)^+$) $C_{11}H_5N_2F_3Na$ $[M + Na]^+$ found 245.0290, requires 245.0297 (-2.9 ppm).

2-(4-(Dimethylamino)benzylidene)malononitrile (34)

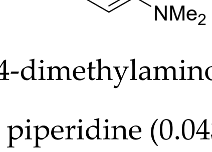

Following General Procedure A, 4-dimethylaminobenzaldehyde (0.75 g, 5.0 mmol), malononitrile (0.33 g, 5.0 mmol) and piperidine (0.043 g, 0.05 mL, 0.50 mmol) in EtOH (10 mL) gave the title compound as orange crystals (0.92 g, 92%). **mp** 170-172 °C {lit[8] 180 °C}; **IR** ν_{max} (film) 2208 (C≡N, nitrile), 1616, 1609, 1558, 1541, 1520, 1456, 1387, 1362, 1194, 1179,

816; **^{1}H NMR** (500 MHz, $CDCl_3$) δ_H: 3.17 (6H, s, C*H*$_3$), 6.72 – 6.77 (2H, m, ArC(3,5)*H*), 7.50 (1H, s, C(2)*H*), 7.81 – 7.87 (2H, m, ArC(2,6)*H*); **^{13}C{^{1}H} NMR** (126 MHz, $CDCl_3$) δ_C: 40.1 ($N(CH_3)_2$), 71.7 (*C*(1)), 111.6 (Ar*C*(3,5)H), 115.0 (*C*N), 116.1 (*C*N), 119.3 (Ar*C*(1)), 133.8 (Ar*C*(2,6)H), 154.3 (Ar*C*(4)), 158.1 (*C*(2)H); **HRMS** (EI^+) $C_{12}H_{11}N_3$ $[M]^+$ found 197.0950, requires 197.0947 (+1.5 ppm).

(*E*)-2-(3-phenylallylidene)malononitrile (35)

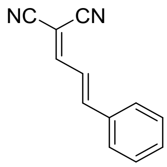

Adapting the procedure of Chen and co-workers,[9] cinnamaldehyde (1.26 mL, 10 mmol, 1.0 equiv.), malononitrile (0.66 g, 10 mmol, 1.0 equiv.) and K_2CO_3 (0.14 g, 1.0 mmol, 0.1 equiv.) were ground together rapidly in a mortar for 5 min. Once complete, the reaction mixture was dissolved in CH_2Cl_2, extracted with H_2O (x2) then dried over $MgSO_4$, filtered, and the solvent removed under reduced pressure to give the crude product as a yellow solid. The crude product was recrystallised from ethanol to give the title compound as yellow crystals (0.84 g, 47%). **mp** 124-126 °C (EtOH) {lit[10] 128 °C}; **IR** ν_{max} (film) 3032 (C-H), 2222 (C≡N, nitrile), 1609, 1576, 1560, 1539, 1491, 1449, 1358, 1321, 1279, 1179, 1152, 976; **^{1}H NMR** (500 MHz, $CDCl_3$) δ_H: 7.24 – 7.35 (2H, m, C(3)*H* and C(4)*H*), 7.44 – 7.54 (3H, m, ArC(3,4,5)*H*), 7.60 – 7.66 (3H, m, ArC(2,6)*H* and C(2)*H*); **^{13}C{^{1}H} NMR** (126 MHz, $CDCl_3$) δ_C: 82.9 (*C*(1)), 111.7 (*C*N), 113.6 (*C*N), 122.3 (*C*(3)H), 129.0 (Ar*C*(2,6)H), 129.4 (Ar*C*(3,5)H), 132.2 (ArC(4)H), 133.9 (Ar*C*(1)), 150.6 (*C*(4)H), 160.2 (*C*(2)H); **HRMS** (EI^+) $C_{12}H_8N_2$ $[M]^+$ found 180.0682, requires 180.0682 (+0.0 ppm).

1-propylidenemalononitrile (36)

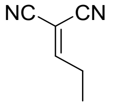

Following the procedure of McQuade and co-workers,[11] to a stirred solution of malononitrile (1.32 g, 20 mmol, 1.0 equiv.) and propionaldehyde (1.44 mL, 20 mmol, 1.0

equiv.) in $CHCl_3$ (1.0 M) was added slowly Al_2O_3 (activated basic, Brockmann I) (2.85 g, 28 mmol, 1.4 equiv.). After addition, the reaction mixture was stirred at RT for 1 h then the Al_2O_3 filtered off, washing with CH_2Cl_2. The filtrate was concentrated under reduced pressure then the residue distilled to give the title compound as a colourless oil (1.48 g, 70%). **^{1}H NMR** (500 MHz, $CDCl_3$) δ_H: 1.22 (3H, t, *J* 7.6, CH_3), 2.63 (2H, dq, *J* 7.6, 7.6, CH_2CH$_3$), 7.35 (1H, t, *J* 7.6, C*H*=C); **^{13}C {^{1}H} NMR** (126 MHz, $CDCl_3$) δ_C: 12.0 (CH$_3$), 26.4 (*C*H$_2$CH$_3$), 89.5 (CH=*C*), 110.4 (*C*N), 112.1 (*C*N), 170.8 (*C*H=C).
Spectroscopic data in accordance with the literature.[11]

7 Preparation of electron-deficient aryl esters

Ester **7** was available in the laboratory, previously prepared following the literature procedure.[2]

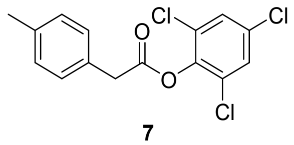

7

4-Nitrophenyl 2-(*p*-tolyl)acetate (1)

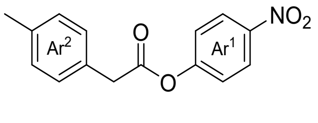

Following General Procedure B, Method B, 2-(*p*-tolyl)acetic acid (5.0 g, 34 mmol, 1.0 equiv.), EDCI·HCl (8.4 g, 44 mmol, 1.3 equiv.) and 4-nitrophenol (7.0 g, 50 mmol, 1.5 equiv.) in CH_2Cl_2 (0.6 M) for 24 h gave crude product which was purified by flash silica column chromatography (CH_2Cl_2, R_f 0.71) to give the title compound as yellow-white crystals (6.9 g, 75%). **mp** 58-60 °C {Lit[2] 60-62 °C}; **^{1}H NMR** (400 MHz, $CDCl_3$) δ_H: 2.39 (3H, s, CH_3), 3.88 (2H, s, C(2)H_2), 7.19 – 7.25 (2H, m, Ar^{2}C(3,5)*H*), 7.25 – 7.32 (4H, m, Ar^{1}C(2,6)*H* and Ar^{2}C(2,6)*H*), 8.23 – 8.30 (2H, m, Ar^{1}C(3,5)*H*).
Spectroscopic data in accordance with literature.[2]

3,5-Bis(trifluoromethyl)phenyl 2-(*p*-tolyl)acetate (6)

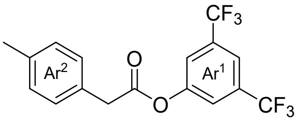

Following General Procedure B, Method A, 2-(*p*-tolyl)acetic acid (1.25 g, 8.4 mmol, 2.0 equiv.), EDCI·HCl (1.20 g, 6.3 mmol, 1.5 equiv.) and 3,5-bis(trifluoromethyl)phenol (0.96 g, 4.2 mmol, 1.0 equiv.) in CH_2Cl_2 (0.3 M) for 54 h gave crude product which was purified by flash silica column chromatography (CH_2Cl_2) to give the title compound as a colourless solid (1.36 g, 89%). **mp** 48-50 °C {Lit[2] 57-58 °C}; **^{1}H NMR** (500 MHz, $CDCl_3$) δ_H: 2.39 (3H, s, C*H*$_3$), 3.89 (2H, s, C(2)*H*$_2$), 7.21 – 7.24 (2H, m, Ar^{2}C(2,6)*H*), 7.27 – 7.30 (2H, m, Ar^{2}C(3,5)*H*), 7.59 (2H, s, Ar^{1}C(2,6)*H*), 7.76 (1H, s, Ar^{1}C(4)*H*); **^{19}F{^{1}H} NMR** (470 MHz, $CDCl_3$) δ_F: -62.9 (s).

Spectroscopic data in accordance with literature.[2]

2,3,4,5,6-Pentafluorophenyl 2-(*p*-tolyl)acetate (8)

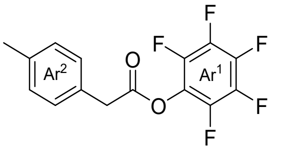

Following General Procedure B, Method B, 2-(*p*-tolyl)acetic acid (1.50 g, 10 mmol, 1.0 equiv.), EDCI·HCl (2.50 g, 13 mmol, 1.3 equiv.) and pentafluorophenol (2.76 g, 15 mmol, 1.5 equiv.) in CH_2Cl_2 (0.6 M) for 72 h gave crude product which was purified by flash silica column chromatography (Petrol:CH_2Cl_2 1:1, R_f 0.71) to give the title compound as yellow-white crystals (1.86 g, 59%). **mp** 28-29 °C {Lit[2] 27-29 °C}; **^{1}H NMR** (500 MHz, $CDCl_3$) δ_H: 2.38 (3H, s, C*H*$_3$), 3.95 (2H, s, C(2)*H*$_2$), 7.19 – 7.23 (2H, m, Ar^{2}C(3,5)*H*), 7.25 – 7.28 (2H, m, Ar^{2}C(2,6)*H*); **^{19}F{^{1}H} NMR** (376 MHz, $CDCl_3$) δ_F: -162.5 – -162.2 (m), -157.9 (t, *J* 21.7), -152.7 – -152.5 (m).

Spectroscopic data in accordance with literature.[2]

2,3,5,6-Tetrafluorophenyl 2-(*p*-tolyl)acetate (9)

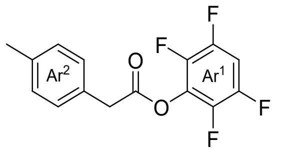

Following General Procedure C, 2-(*p*-tolyl)acetic acid (10.1 g, 67.0 mmol, 2.0 equiv.), EDCI·HCl (9.63 g, 50.3 mmol, 1.5 equiv.) and 2,3,5,6-tetrafluorophenol (5.56 g, 33.5 mmol, 1.0 equiv.) in CH_2Cl_2 (0.3 M) for 240 h gave crude product which was purified by flash silica column chromatography (CH_2Cl_2, R_f 0.75) to give the title compound as a yellow-white solid (9.19 g, 92%). **mp** 28-30 °C {lit[2] 27-29 °C}; **^{1}H NMR** (400 MHz, $CDCl_3$) δ_H: 2.38 (3H, s, CH_3), 3.96 (2H, s, C(2)H_2), 7.01 (1H, tt, *J* 9.9, 7.0, Ar^{1}C(4)*H*), 7.18 – 7.24 (2H, m, Ar^{2}C(2,6)*H*), 7.25 – 7.31 (2H, m, Ar^{2}C(3,5)*H*); **^{19}F{^{1}H} NMR** (376 MHz, $CDCl_3$) δ_F: -152.9 – -152.8 (m), -139.1 – -139.0 (m).
Spectroscopic data in accordance with literature.[2]

2,3,5,6-Tetrafluorophenyl 2-(4-methoxyphenyl)acetate (40)

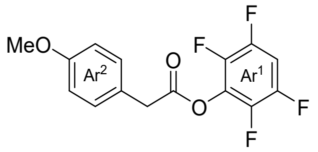

Following General Procedure C, 2-(4-methoxyphenyl)acetic acid (3.32 g, 20 mmol, 2.0 equiv.), EDCI·HCl (2.88 g, 15 mmol, 1.5 equiv.) and 2,3,5,6-tetrafluorophenol (1.66 g, 10 mmol, 1.0 equiv.) in CH_2Cl_2 (0.3 M) for 72 h gave crude product which was purified by flash silica column chromatography (CH_2Cl_2, R_f 0.82) to give the title compound as a colourless solid (2.71 g, 86 %). **mp** 30-32 °C; **IR** ν_{max} (film) 1786 (C=O, ester), 1522, 1514, 1485, 1086; **^{1}H NMR** (500 MHz, $CDCl_3$) δ_H: 3.84 (3H, s, OCH_3), 3.95 (2H, s, C(2)H_2), 6.93 – 6.97 (2H, m, Ar^{2}C(3,5)*H*), 7.01 (1H, tt, *J* 10.0, 7.0, Ar^{1}C(4)*H*), 7.30 – 7.35 (2H, m, Ar^{2}C(2,6)*H*); **^{19}F{^{1}H} NMR** (470 MHz, $CDCl_3$) δ_F: -152.9 – -152.8 (m), -139.1 – -139.0 (m); **^{13}C{^{1}H} NMR** (126 MHz, $CDCl_3$) δ_C: 39.4 (*C*(2)H_2), 55.2 (OCH_3), 103.2 (t, *J* 22.8, Ar^1*C*(4)H), 114.2 (Ar^2*C*(3,5)H), 124.2 (Ar^2*C*(1)), 129.7 (tt, *J* 14.1, 3.9, Ar^{1}C(1)), 130.4 (Ar^{2}C(2,6)H), 140.6 (m, Ar^{1}CF), 146.0 (m, Ar^{1}CF), 159.2 (Ar^2*C*(4)), 167.8 (*C*(1)); **HRMS** (EI^+) $C_{15}H_{10}O_3F_4$ $[M]^+$ found 314.0572, requires 314.0561 (+3.7 ppm).

2,3,5,6-Tetrafluorophenyl 2-(4-(dimethylamino)phenyl)acetate (S12)

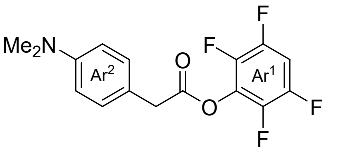

Following General Procedure C, 2-(4-(dimethylamino)phenyl)acetic acid (1.79 g, 10 mmol, 2.0 equiv.), EDCI·HCl (1.44 g, 7.5 mmol, 1.5 equiv.) and 2,3,5,6-tetrafluorophenol (0.83 g, 5.0 mmol, 1.0 equiv.) in CH_2Cl_2 (0.3 M) for 99 h gave crude product which was purified by flash silica column chromatography (Petrol:CH_2Cl_2 1:1, R_f 0.53) to give the title compound as an off-white solid (1.45 g, 88%). **mp** 47-49 °C; **IR** ν_{max} (film) 1782 (C=O, ester), 1522, 1485, 1086; **^{1}H NMR** (400 MHz, $CDCl_3$) δ_H: 2.98 (6H, s, N(CH_3)$_2$), 3.90 (2H, s, C(2)H_2), 6.73 – 6.78 (2H, m, Ar^2C(3,5)*H*), 6.99 (1H, tt, *J* 9.9, 7.0, Ar^1C(4)*H*), 7.22 – 7.27 (2H, m, Ar^2C(2,6)*H*); **^{19}F{^{1}H} NMR** (376 MHz, $CDCl_3$) δ_F: -152.9 – -152.7 (m), -139.3 – -139.1 (m); **^{13}C{^{1}H} NMR** (126 MHz, $CDCl_3$) δ_C: 39.3 (*C*(2)H_2), 40.6 (CH_3), 103.1 (t, *J* 22.9, Ar^1*C*(4)H), 112.7 (Ar^2*C*(3,5)H), 119.6 (Ar^2*C*(1)), 129.8 – 130.1 (m, Ar^1*C*(1) and Ar^2*C*(2,6)H), 140.7 (m, Ar^1CF), 146.0 (m, Ar^1CF), 150.0 (Ar^2*C*(4)), 168.1 (*C*(1)); **HRMS** (EI^+) $C_{16}H_{13}O_2N_1F_4$ $[M]^+$ found 327.0883, requires 327.0877 (+1.7 ppm).

2,3,5,6-Tetrafluorophenyl 2-(thiophen-3-yl)acetate (S13)

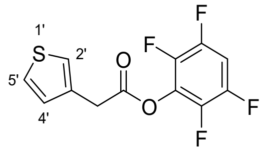

Following General Procedure C, 2-(thiophen-3-yl)acetic acid (1.42 g, 10 mmol, 2.0 equiv.), EDCI·HCl (1.44 g, 7.5 mmol, 1.5 equiv.) and 2,3,5,6-tetrafluorophenol (0.83 g, 5.0 mmol, 1.0 equiv.) in CH_2Cl_2 (0.3 M) for 165 h gave crude product which was purified by flash silica column chromatography (CH_2Cl_2, R_f 0.80) to give the title compound as a yellow oil (1.36 g, 94 %). **IR** ν_{max} (film) 1786 (C=O, ester), 1522, 1485, 1092; **^{1}H NMR** (400 MHz, $CDCl_3$) δ_H: 4.05 (2H, s, C(2)H_2), 7.03 (1H, tt, *J* 9.9, 7.1, Ar^1C(4)*H*), 7.15 (1H, dd, *J* 5.0, 1.3, C(4′)*H*), 7.30 – 7.33 (1H, m, C(2′)*H*), 7.38 (1H, dd, *J* 5.0, 3.0, C(5′)*H*); **^{19}F{^{1}H} NMR** (376 MHz, $CDCl_3$) δ_F: -152.9 – -152.8 (m), -139.0 – -138.8 (m); **^{13}C{^{1}H} NMR** (126 MHz, $CDCl_3$) δ_C: 34.7 (*C*(2)H_2), 103.3 (t, *J* 22.8, Ar^1*C*(4)H), 123.7 (*C*(2′)H), 126.3 (*C*(5′)H), 128.2 (*C*(4′)H), 129.6 (tt, *J* 13.9, 3.7,

Ar^{1}C(1)), 131.6 (C(3)), 140.6 (m, Ar^{1}CF), 146.0 (m, Ar^{1}CF), 167.0 (C(1)); **HRMS** (EI^+) $C_{12}H_{6}$-$O_2F_4{}^{32}S_1$ $[M]^+$ found 290.0009, requires 290.0019 (-3.4 ppm).

2,3,5,6-Tetrafluorophenyl (*E*)-pent-3-enoate (S14)

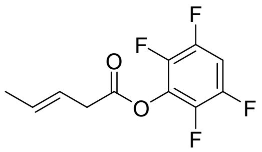

Following General Procedure C, (*E*)-pent-3-enoic acid (1.02 mL, 10 mmol, 2.0 equiv.), EDCI·HCl (1.44 g, 7.5 mmol, 1.5 equiv.) and 2,3,5,6-tetrafluorophenol (0.83 g, 5 mmol, 1.0 equiv.) in CH_2Cl_2 (0.3 M) for 168 h gave crude product which was purified by flash silica column chromatography (CH_2Cl_2, R_f 0.77) to give the title compound as a colourless oil (1.03 g, 83%). **IR** ν_{max} (film) 3086 (C-H), 1790 (C=O, ester), 1522, 1485, 1179, 1086, 955; **^{1}H NMR** (400 MHz, $CDCl_3$) δ_H: 1.74 – 1.80 (3H, m, C(5)H_3), 3.38 – 3.42 (2H, m, C(2)H_2), 5.58 – 5.68 (1H, m, C(4)H), 5.71 – 5.82 (1H, m, C(3)H), 7.02 (1H, tt, *J* 9.9, 7.1, ArC(4)H); **^{19}F{^{1}H} NMR** (376 MHz, $CDCl_3$) δ_F: -153.1 – -153.0 (m), -139.2 – -139.0 (m); **^{13}C{^{1}H} NMR** (126 MHz, $CDCl_3$) δ_C: 18.0 (C(5)H_3), 37.0 (C(2)H_2), 103.2 (t, *J* 22.7, ArC(4)H), 120.7 (C(4)H), 129.5 – 129.9 (m, ArC(1)), 131.2 (C(3)H), 139.4 – 141.8 (m, ArCF), 146.0 (m, ArCF), 168.0 (C(1)); **HRMS** ($MALDI\ (no\ matrix)^+$) $C_{11}H_8O_2F_4Na$ $[M+Na]^+$ found 271.0345, requires 271.0353 (-3.0 ppm).

2,3,5,6-Tetrafluorophenyl 2-(*m*-tolyl)acetate (S15)

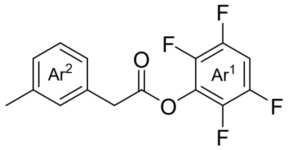

Following General Procedure C, 2-(*m*-tolyl)acetic acid (1.50 g, 10 mmol, 2.0 equiv.), EDCI·HCl (1.44 g, 7.5 mmol, 1.5 equiv.) and 2,3,5,6-tetrafluorophenol (0.83 g, 5 mmol, 1.0 equiv.) in CH_2Cl_2 (0.3 M) for 168 h gave crude product which was purified by flash silica column chromatography (CH_2Cl_2 : hexane 1:1, R_f 0.78) to give the title compound as a colourless oil (1.44 g, 97%). **IR** ν_{max} (film) 3080 (C-H), 1790 (C=O, ester), 1522, 1487, 1179, 1088, 955; **^{1}H NMR** (400 MHz, $CDCl_3$) δ_H: 2.41 (3H, s, CH_3), 3.98 (2H, s, C(2)H_2), 7.01 (1H, tt, *J* 9.9, 7.1, Ar^{1}C(4)H), 7.15 – 7.24 (3H, m, Ar^{2}C(2,4,6)H), 7.28 – 7.33 (1H, m, Ar^{2}C(5)H);

^{19}**F{^{1}H} NMR** (376 MHz, $CDCl_3$) δ_F: -152.9 – -152.8 (m), -139.1 – -139.0 (m); ^{13}**C{^{1}H} NMR** (126 MHz, $CDCl_3$) δ_C: 21.4 (CH_3), 40.2 (*C*(2)H_2), 103.3 (t, *J* 22.8, Ar^1*C*(4)H), 126.3 (Ar^2*C*(6)H), 128.5 (Ar^2*C*(4)H), 128.8 (Ar^2*C*(5)H), 129.5 – 129.9 (m, Ar^1*C*(1)), 130.0 (Ar^2*C*(2)H), 132.1 (Ar^2*C*(1)), 138.6 (Ar^2*C*(3)), 140.6 (m, Ar^{1}CF), 146.0 (m, Ar^{1}CF), 167.6 (*C*(1)); **HRMS** (EI^+) $C_{15}H_{10}O_2F_4$ $[M]^+$ found 298.0606, requires 298.0611 (-1.7 ppm).

2,3,5,6-Tetrafluorophenyl 2-(*o*-tolyl)acetate (S16)

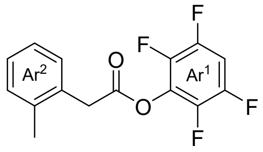

Following General Procedure C, 2-(*o*-tolyl)acetic acid (1.50 g, 10 mmol, 2.0 equiv.), EDCI·HCl (1.44 g, 7.5 mmol, 1.5 equiv.) and 2,3,5,6-tetrafluorophenol (0.83 g, 5 mmol, 1.0 equiv.) in CH_2Cl_2 (0.3 M) for 168 h gave crude product which was purified by flash silica column chromatography (CH_2Cl_2 : hexane 1:1, R_f 0.64) to give the title compound as a colourless solid (1.46 g, 98%). **mp** 54-56 °C; **IR** ν_{max} (film) 3078 (C-H), 1782 (C=O, ester), 1522, 1487, 1179, 1086, 955; ^{1}**H NMR** (400 MHz, $CDCl_3$) δ_H: 2.42 (3H, s, C*H*$_3$), 4.02 (2H, s, C(2)*H*$_2$), 7.01 (1H, tt, *J* 9.9, 7.1, Ar^{1}C(4)*H*), 7.21 – 7.29 (3H, m, Ar^{2}C(3,4,5)*H*), 7.30 – 7.36 (1H, m, Ar^{2}C(6)*H*); ^{19}**F{^{1}H} NMR** (376 MHz, $CDCl_3$) δ_F: -153.0 – -152.9 (m), -139.1 – -139.0 (m); ^{13}**C{^{1}H} NMR** (126 MHz, $CDCl_3$) δ_C: 19.4 (CH_3), 38.3 (*C*(2)H_2), 103.3 (t, *J* 22.7, Ar^1*C*(4)H), 126.4 (Ar^2*C*(5)H), 128.1 (Ar^2*C*(4)H), 129.5 – 129.9 (m, Ar^1*C*(1)), 130.3 (Ar^2*C*(6)H), 130.6 (Ar^2*C*(3)H), 130.8 (Ar^2*C*(1)), 137.0 (Ar^2*C*(2)), 139.5 – 141.8 (m, Ar^{1}CF), 146.0 (m, Ar^{1}CF), 167.4 (*C*(1)); **HRMS** (ESI^+) $C_{15}H_{10}O_2F_4Na$ $[M+Na]^+$ found 321.0512, requires 321.0509 (+0.9 ppm).

2,3,5,6-Tetrafluorophenyl propanoate (33)

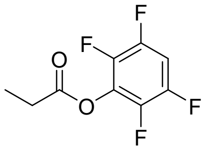

Following General Procedure C, propanoic acid (1.49 mL, 20 mmol, 2.0 equiv.), EDCI·HCl (2.88 g, 15 mmol, 1.5 equiv.) and 2,3,5,6-tetrafluorophenol (1.66 g, 10 mmol, 1.0 equiv.) in CH_2Cl_2 (0.3 M) for 168 h gave crude product which was purified by flash silica column

chromatography (CH_2Cl_2, R_f 0.75) to give the title compound as a colourless oil (1.37 g, 62%). **IR** ν_{max} (film) 3088 (C-H), 1786 (C=O, ester), 1524, 1489, 1179, 1111, 1082, 953; **^{1}H NMR** (400 MHz, $CDCl_3$) δ_H: 1.33 (3H, t, *J* 7.5, CH_2CH_3), 2.73 (2H, q, *J* 7.5, CH_2CH_3), 7.01 (1H, tt, *J* 9.9, 7.1, ArC(4)*H*); **^{19}F{^{1}H} NMR** (376 MHz, $CDCl_3$) δ_F: -153.3 – -153.2 (m), -139.3 – -139.1 (m); **^{13}C{^{1}H} NMR** (126 MHz, $CDCl_3$) δ_C: 8.9 (CH_2CH_3), 26.9 (CH_2CH_3), 103.1 (t, *J* 22.8, ArC(4)H), 129.5 – 131.1 (m, Ar*C*(1)), 139.4 – 141.9 (m, ArCF), 146.0 (m, ArCF), 170.3 (*C*(1)); **HRMS** (ESI^-) $C_9H_5O_2F_4$ $[M-H]^-$ found 221.0232, requires 221.0231 (+0.5 ppm).

8 Ester screen products

(2*R*,3*S*)-4-Nitrophenyl 4,4-dicyano-3-phenyl-2-(*p*-tolyl)butanoate (4) and (2*R*,3*R*)-4-Nitrophenyl 4,4-dicyano-3-phenyl-2-(*p*-tolyl)butanoate (5)

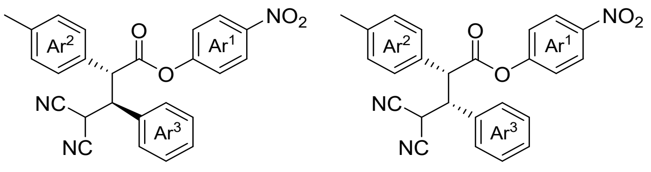

anti-**4** *syn*-**5**

Following General Procedure D, 4-nitrophenyl 2-(p-tolyl)acetate **1** (203 mg, 0.75 mmol, 1.5 eq) gave crude product that was purified by flash silica column chromatography (Petrol:CH_2Cl_2 1:1 to 1:3) to yield the combined *anti* and *syn* diastereoisomers (47:53 dr) (124 mg, 58%) as an inseparable mixture as a colourless solid. **mp** 54-56 °C; $[\alpha]_D^{20}$ -105.9 (*c* 1.4 in $CHCl_3$); **IR** ν_{max} (film) 2922 (C-H), 1755 (C=O, ester), 1526, 1489, 1346, 1204, 1126, 756; **HRMS** (EI^+) $C_{25}H_{19}O_4N_3Na$ $[M+Na]^+$ found 448.1259, requires 448.1268 (-2.0 ppm).

Data for anti-**4**: **Chiral HPLC analysis,** Chiralcel OD-H (97:3 *n*-hexane : IPA, flow rate 1.5 mLmin^{-1}, 211 nm, 40 °C) t_R (2*R*,3*S*) 27.7 min, t_R (2*S*,3*R*) 31.3 min, 79:21 er; **^{1}H NMR** (400 MHz, $CDCl_3$) δ_H: 2.45 (3H, s, C*H*$_3$), 3.75 (1H, d, *J* 4.0, C(4)*H*), 3.97 (1H, dd, *J* 12.2, 4.0, C(3)*H*), 4.57 (1H, d, *J* 12.2, C(2)*H*), 6.69 – 6.74 (2H, m, Ar^{1}C(2,6)*H*), 7.34 – 7.40 (2H, m, Ar^{2}C(3,5)*H*), 7.51 – 7.58 (5H, m, Ar^{2}C(2,6)*H* and Ar^{3}C(3,4,5)*H*), 7.63 – 7.69 (2H, m, Ar^{3}C(2,6)*H*), 8.09 – 8.16 (2H, m, Ar^{1}C(3,5)*H*); **^{13}C{^{1}H} NMR** (126 MHz, $CDCl_3$) δ_C: 21.3 (CH_3), 27.7 (*C*(4)H), 49.4 (*C*(3)H), 53.6 (*C*(2)H), 110.7 (CN), 111.3 (CN), 122.0 (Ar^1*C*(2,6)H), 125.2 (Ar^1*C*(3,5)H), 128.1 (Ar^3*C*(4)H), 128.6 (Ar^3*C*(2,6)H), 129.5 (Ar^3*C*(3,5)H), 129.8 (Ar^2*C*(1)), 129.9 (Ar^2*C*(2,6)H), 131.0 (Ar^2*C*(3,5)H), 134.1 (Ar^3*C*(1)), 140.2 (Ar^{2}C(4)), 145.6 (Ar^{1}C(1)), 154.6 (Ar^1*C*(4)), 168.5 (*C*(1)).

Data for syn-**5**: **Chiral HPLC analysis,** Chiralcel OD-H (97:3 *n*-hexane : IPA, flow rate 1.5 mLmin^{-1}, 211 nm, 40 °C) t_R (2*S*,3*S*) 43.2 min, t_R (2*R*,3*R*) 62.1 min, 93:7 er; **^{1}H NMR** (400 MHz, $CDCl_3$) δ_H: 2.27 (3H, s, C*H*$_3$), 4.01 (1H, dd, *J* 11.4, 5.4, C(3)*H*), 4.53 (1H, d, *J* 11.4, C(2)*H*), 4.72 (1H, d, *J* 5.4, C(4)*H*), 7.03 – 7.10 (4H, m, Ar^{2}C(2,3,5,6)*H*), 7.17 – 7.22 (2H, m, Ar^{1}C(2,6)*H*), 7.26 – 7.34 (5H, m, Ar^3(2,6)*H* and Ar^{3}C(3,4,5)*H*), 8.24 – 8.31 (2H, m, Ar^{1}C(3,5)*H*); **^{13}C{^{1}H} NMR** (126 MHz, $CDCl_3$) δ_C: 21.1 (CH_3), 28.0 (*C*(4)H), 47.8 (*C*(3)H), 53.0 (*C*(2)H), 111.2 (*C*N), 111.5 (*C*N), 122.2 (Ar^1*C*(2,6)H), 125.3 (Ar^1*C*(3,5)H), 128.2 (Ar^{2}C(2,6)H), 128.6 (Ar^{3}C(2,6)H), 129.1 (Ar^{3}C(3,5)H), 129.2 (Ar^{3}C(4)H), 129.9 (Ar^2*C*(3,5)H),

130.6 (Ar^2*C*(1)), 133.6 (Ar^3*C*(1)), 138.6 (Ar^2*C*(4)), 145.8 (Ar^1*C*(1)), 154.7 (Ar^1*C*(4)), 170.5 (*C*(1)).

(2*R*,3*S*)-3,5-Bis(trifluoromethyl)phenyl 4,4-dicyano-3-phenyl-2-(*p*-tolyl)butanoate (10) and (2*R*,3*R*)-3,5-Bis(trifluoromethyl)phenyl 4,4-dicyano-3-phenyl-2-(*p*-tolyl)butanoate (11)

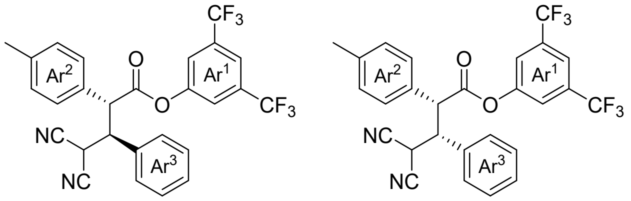

anti-**10** *syn*-**11**

Following General Procedure D, 3,5-bis(trifluoromethyl)phenyl 2-(*p*-tolyl)acetate **6** (272 mg, 0.75 mmol, 1.5 eq) gave crude product that was purified by flash silica column chromatography (Petrol:Acetone 98:2 to 80:20) to yield the combined *anti* and *syn* diastereoisomers (52:48 dr) (125 mg, 48%) as an inseparable mixture as a colourless glass; $[\alpha]_D^{20}$ -73.3 (*c* 1.4 in $CHCl_3$); **IR** ν_{max} (film) 1761 (C=O, ester), 1369, 1279, 1179, 1136; **HRMS** (EI^+) $C_{27}H_{18}O_2N_2F_6Na$ $[M+Na]^+$ found 539.1155, requires 539.1165 (-1.8 ppm).

Data for anti-**10**: **Chiral HPLC analysis,** Chiralcel OD-H (99.5:0.5 *n*-hexane : IPA, flow rate 1.0 mLmin^{-1}, 211 nm, 30 °C) t_R (2*R*,3*S*) 32.6 min, t_R (2*S*,3*R*) 39.8 min, 87:13 er; **^{1}H NMR** (500 MHz, $CDCl_3$) δ_H: 2.46 (3H, s, C*H*$_3$), 3.75 (1H, d, *J* 4.1, C(4)*H*), 3.95 (1H, dd, *J* 12.2, 4.1, C(3)*H*), 4.55 (1H, d, *J* 12.2, C(2)*H*), 7.35 – 7.40 (2H, m, Ar^{2}C(3,5)*H*), 7.50 (2H, s, Ar^{1}C(2,6)*H*), 7.53 – 7.59 (5H, m, Ar^{2}C(2,6)*H* and Ar^{3}C(3,4,5)*H*), 7.64 – 7.70 (2H, m, Ar^{3}C(2,6)*H*), 7.80 (1H, s, Ar^{1}C(4)*H*); **^{19}F{^{1}H} NMR** (470 MHz, $CDCl_3$) δ_F: -69.2 (s); **^{13}C{^{1}H} NMR** (126 MHz, $CDCl_3$) δ_C: 21.2 (*C*H$_3$), 27.6 (*C*(4)H), 49.6 (*C*(3)H), 53.6 (*C*(2)H), 110.7 (CN), 111.5 (CN), 120.2 – 120.4 (m, Ar^1*C*(4)H), 121.9 – 122.2 (m, Ar^1*C*(2,6)H), 122.5 (q, *J* 273, *C*F$_3$), 128.6 (Ar^2*C*(2,6)H), 128.7 (Ar^3*C*(2,6)H), 130.0 (Ar^3*C*(3,5)H), 130.1 (Ar^3*C*(4)H), 130.4 (Ar^2*C*(1)), 131.0 (Ar^2*C*(3,5)H), 132.9 (q, *J* 34.4, Ar^1*C*(3,5)), 134.0 (Ar^3*C*(1)), 140.3 (Ar^2*C*(4)), 150.6 (Ar^1*C*(1)), 168.8 (*C*(1)).

Data for syn-**11**: **Chiral HPLC analysis,** Chiralcel OD-H (99.5:0.5 *n*-hexane : IPA, flow rate 1.0 mLmin^{-1}, 211 nm, 30 °C) t_R (2*S*,3*S*) 24.2 min, t_R (2*R*,3*R*) 26.9 min, 97:3 er; **^{1}H NMR** (500 MHz, $CDCl_3$) (*selected*) δ_H: 2.28 (3H, s, C*H*$_3$), 4.00 (1H, dd, *J* 11.4, 5.4, C(3)*H*), 4.52 (1H, d, *J* 11.4, C(2)*H*), 4.71 (1H, d, *J* 5.4, C(4)*H*), 6.92 (2H, s, Ar^{1}C(2,6)*H*), 7.03 – 7.10 (4H, m,

Ar^{2}C(2,3,5,6)*H*), 7.25 – 7.35 (5H, m, Ar^3(2,3,4,5,6)*H*); **^{19}F{^{1}H} NMR** (470 MHz, CDCl$_3$) δ_F: -63.1 (s); **^{13}C{^{1}H} NMR** (126 MHz, CDCl$_3$) (*selected*) δ_C: 21.1 (CH$_3$), 28.0 (*C*(4)H), 47.9 (*C*(3)H), 53.0 (*C*(2)H), 111.2 (CN), 111.2 (CN), 120.0 – 120.2 (m, Ar^{1}C(4)H), 122.5 (q, *J* 273, CF$_3$), 128.2 (Ar^2*C*(2,6)H), 129.1(Ar^3*C*(3,5)H), 129.2 (Ar^3*C*(4)H), 129.5 (Ar^2*C*(1)), 133.6 (Ar^3*C*(1)), 138.7 (Ar^2*C*(4)), 150.4 (Ar^{1}C(1)), 170.5 (*C*(1)).

(2*R*,3*S*)-Perfluorophenyl 4,4-dicyano-3-phenyl-2-(*p*-tolyl)butanoate (12) and (2*R*,3*R*)-Perfluorophenyl 4,4-dicyano-3-phenyl-2-(*p*-tolyl)butanoate (13)

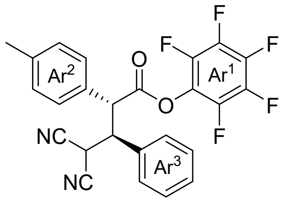

anti-**12**

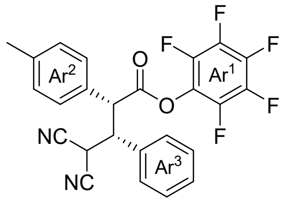

syn-**13**

Following General Procedure D, 2,3,4,5,6-pentafluorophenyl 2-(*p*-tolyl)acetate **8** (237 mg, 0.75 mmol, 1.5 eq) gave crude product that was purified by flash silica column chromatography (Petrol:Acetone 95:5 to 80:20) to yield the combined *anti* and *syn* diastereoisomers (78:22 dr) (149 mg, 63%) as an inseparable mixture as a colourless solid. **mp** 138-140 °C; $[\alpha]_D^{20}$ -68.0 (*c* 2.3 in CHCl$_3$); **IR** ν_{max} (film) 1780 (C=O, ester), 1520, 1109, 997; **HRMS** (EI^+) $C_{25}H_{15}O_2N_2F_5Na$ $[M+Na]^+$ found 493.0942, requires 493.0946 (-0.8 ppm).

*Data for major diastereoisomer anti-***12**: **Chiral HPLC analysis,** Chiralcel OD-H (99.5:0.5 *n*-hexane : IPA, flow rate 1.0 mLmin^{-1}, 211 nm, 30 °C) t_R (2*S*,3*R*) 39.2 min, t_R (2*R*,3*S*) 42.6 min, 89:11 er; **^{1}H NMR** (500 MHz, CDCl$_3$) δ_H: 2.45 (3H, s, C*H*$_3$), 3.73 (1H, d, *J* 3.9, C(4)*H*), 3.97 (1H, dd, *J* 12.2, 3.9, C(3)*H*), 4.67 (1H, d, *J* 12.2, C(2)*H*), 7.34 – 7.38 (2H, m, Ar^{2}C(3,5)*H*), 7.47 – 7.55 (5H, m, Ar^{2}C(2,6)*H* and Ar^{3}C(3,4,5)*H*), 7.60 – 7.64 (2H, m, Ar^{3}C(2,6)*H*); **^{19}F{^{1}H} NMR** (470 MHz, CDCl$_3$) δ_F: -162.1 – -161.9 (m), -157.2 (t, *J* 21.8), -152.5 – -152.4 (m); **^{13}C{^{1}H} NMR** (126 MHz, CDCl$_3$) δ_C: 21.3 (CH$_3$), 27.8 (*C*(4)H), 48.9 (*C*(3)H), 52.8 (*C*(2)H), 110.6 (CN), 111.3 (CN), 124.0 – 124.7 (m, Ar^{1}C(1)), 128.0 (Ar^{3}C(4)H), 128.3 (Ar^{3}C(2,6)H), 129.4 (Ar^{2}C(1)), 129.5 (Ar^{3}C(3,5)H), 129.9 (Ar^{2}C(2,6)H), 131.0 (Ar^{2}C(3,5)H), 133.7 (Ar^3*C*(1)), 136.4 – 138.7 (m, Ar^{1}CF), 138.7 – 140.9 (m, Ar^{1}CF), 139.6 – 141.9 (m, Ar^{1}CF), 140.4 (Ar^{2}C(4)), 166.8 (*C*(1)).

*Data for minor diastereoisomer syn-***13**: **Chiral HPLC analysis,** Chiralcel OD-H (99.5:0.5 *n*-hexane : IPA, flow rate 1.0 mLmin^{-1}, 211 nm, 30 °C) t_R (2*S*,3*S*) 33.2 min, t_R (2*R*,3*R*) 57.5 min,

93:7 er; **^{1}H NMR** (500 MHz, $CDCl_3$) (*selected*) δ_H: 2.27 (3H, s, C*H*$_3$), 4.62 (1H, d, *J* 11.3, C(2)*H*), 7.02 – 7.07 (4H, m, Ar^{2}C(2,3,5,6)*H*), 7.26 – 7.34 (5H, m, Ar^3(2,6)*H* and Ar^{3}C(3,4,5)*H*); **^{19}F{^{1}H} NMR** (470 MHz, $CDCl_3$) δ_F: -161.6 – -161.4 (m), -156.5 (t, *J* 21.6), -152.3 – -152.1 (m); **^{13}C{^{1}H} NMR** (126 MHz, $CDCl_3$) (*selected*) δ_C: 21.1 (*C*H$_3$), 27.8 (*C*(4)H), 47.9 (*C*(3)H), 52.3 (*C*(2)H), 111.0 (*C*N), 111.1 (*C*N), 128.2 (Ar^{2}C(2,6)H), 128.6 (Ar^3*C*(2,6)H), 129.1 (Ar^3*C*(3,5)H), 129.3 (Ar^{3}C(4)H), 129.8 (Ar^2*C*(1)), 129.9 (Ar^{2}C(3,5)H), 133.1 (Ar^{3}C(1)), 138.8 (Ar^{2}C(4)), 169.1 (*C*(1)).

9 Isothiourea catalysis products

(2*R*,3*S*)-2,3,5,6-tetrafluorophenyl 4,4-dicyano-3-phenyl-2-(*p*-tolyl)butanoate (14)

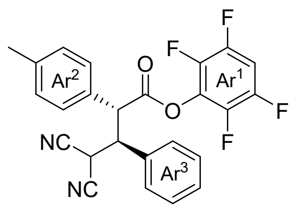

anti-**14**

Following General Procedure E, 2,3,5,6-tetrafluorophenyl 2-(p-tolyl)acetate **9** (224 mg, 0.75 mmol, 1.5 eq), 2-benzylidene malononitrile **2** (78 mg, 0.50 mmol, 1.0 eq), and (4b*R*,11a*S*)-fused-BTM **19** (6.6 mg, 0.025 mmol, 5 mol%) in CPME (1.0 M) gave crude product that was purified by flash silica chromatography (hexane:acetone 90:10 to 80:20, R_F 0.10 at 90:10) to give the combined *anti* and *syn* diastereoisomers (95:5 dr) (208 mg, 92%) as an inseparable mixture. The mixture of diastereoisomers was recrystallized from hexane/CH_2Cl_2 to give the title compound (174 mg, 77%, single major diastereoisomer) as a colourless solid. **mp** 179-181 °C; $[\alpha]_D^{20}$ -64.6 (*c* 2.0 in $CHCl_3$); **Chiral HPLC analysis,** Chiralcel OD-H (99:1 *n*-hexane : IPA, flow rate 1.5 mLmin^{-1}, 211 nm, 30 °C) t_R (2*S*,3*R*) 24.4 min, t_R (2*R*,3*S*) 29.4 min, >99:1 er; **IR** ν_{max} (film) 2907 (C-H), 1788 (C=O, ester), 1524, 1489, 1180, 1117, 1088, 1070, 953; **^{1}H NMR** (400 MHz, $CDCl_3$) δ_H: 2.45 (3H, s, CH_3), 3.75 (1H, d, *J* 4.0, C(4)*H*), 3.99 (1H, dd, *J* 12.2, 4.0, C(3)*H*), 4.69 (1H, d, *J* 12.2, C(2)*H*), 6.93 (1H, tt, *J* 9.9, 7.0 Ar^{1}C(4)*H*), 7.33 – 7.39 (2H, m, Ar^{2}C(3,5)*H*), 7.47 – 7.57 (5H, m, Ar^{2}C(2,6)*H* and Ar^{3}C(3,4,5)*H*), 7.61 – 7.67 (2H, m, Ar^{3}C(2,6)*H*); **^{19}F{^{1}H} NMR** (376 MHz, $CDCl_3$) δ_F: -152.8 – -152.6 (m), -138.8 – -138.6 (m); **^{13}C{^{1}H} NMR** (126 MHz, $CDCl_3$) δ_C: 21.2 (CH_3), 27.8 (*C*(4)H), 48.9 (*C*(3)H), 52.9 (*C*(2)H), 103.5 (t, *J* 22.7, Ar^{1}C(4)H), 110.7 (CN), 111.3 (CN), 128.1 (Ar^3*C*(4)H), 128.3 (Ar^3*C*(2,6)H), 128.6 – 129.1 (Ar^1*C*(1)), 129.5 (Ar^3*C*(3,5)H), 129.9 (Ar^2*C*(2,6)H), 129.9 (Ar^2*C*(1)), 131.0 (Ar^2*C*(3,5)H), 133.7 (Ar^3*C*(1)), 139.0 – 141.4 (m, Ar^{1}CF), 140.3 (Ar^2*C*(4)), 144.6 – 147.0 (m, Ar^{1}CF), 166.7 (*C*(1)). **HRMS** (ESI^+) $C_{25}H_{16}O_2N_2F_4Na$ $[M+Na]^+$ found 475.1023, requires 475.1040 (-3.6 ppm).

(2*R*,3*S*)-2,3,5,6-tetrafluorophenyl 4,4-dicyano-2-(4-methoxyphenyl)-3-phenylbutanoate (20) and (2*R*,3*R*)-2,3,5,6-tetrafluorophenyl 4,4-dicyano-2-(4-methoxyphenyl)-3-phenylbutanoate (S17)

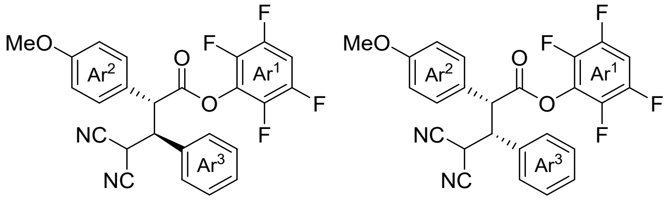

anti-**20** *syn*-**S17**

Following General Procedure E, 2,3,5,6-tetrafluorophenyl 2-(4-methoxyphenyl)acetate **40** (236 mg, 0.75 mmol, 1.5 eq), 2-benzylidenemalononitrile **2** (78 mg, 0.50 mmol, 1.0 eq), and (4b*R*,11a*S*)-fused-BTM **19** (6.6 mg, 0.025 mmol, 5 mol%) in CPME (1.0 M) gave crude product (85:15 dr) that was purified by flash silica column chromatography (hexane:Et_2O 90:10 to 70:30) to give:

Anti-**20** (152 mg, 65%) as a colourless solid. **mp** 52–54 °C; $[\alpha]_D^{20}$ -65.9 (*c* 2.1 in $CHCl_3$); **Chiral HPLC analysis,** Chiralcel OD-H (90:10 *n*-hexane : IPA, flow rate 1.0 $mLmin^{-1}$, 211 nm, 30 °C) t_R (2*S*,3*R*) 19.3 min, t_R (2*R*,3*S*) 22.7 min, 99:1 er; **IR** ν_{max} (film) 2903 (C-H), 1778 (C=O, ester), 1609, 1524, 1512, 1487, 1254, 1179, 1109, 957; **^{1}H NMR** (400 MHz, $CDCl_3$) δ_H: 3.77 (1H, d, *J* 4.0, C(4)*H*), 3.89 (3H, s, OC*H*$_3$), 3.97 (1H, dd, *J* 12.2, 4.0, C(3)*H*), 4.67 (1H, d, *J* 12.2, C(2)*H*), 6.93 (1H, tt, *J* 9.9, 7.0, Ar^1C(4)*H*), 7.03 – 7.10 (2H, m, Ar^2C(3,5)*H*), 7.46 – 7.59 (5H, m, Ar^2C(2,6)*H* and Ar^3C(3,4,5)*H*), 7.60 – 7.66 (2H, m, Ar^3C(2,6)*H*); **^{19}F{^{1}H} NMR** (376 MHz, $CDCl_3$) δ_F: -152.8 – 152.7 (m), -138.8 – -138.6 (m); **^{13}C{^{1}H} NMR** (126 MHz, $CDCl_3$) δ_C: 27.8 (*C*(4)H), 48.9 (*C*(3)H), 52.5 (*C*(2)H), 55.5 (OCH_3), 103.6 (t, *J* 22.7, Ar^1*C*(4)H), 110.7 (CN), 111.4 (CN), 115.6 (Ar^2*C*(3,5)H), 124.2 (Ar^2*C*(1)), 128.3 (Ar^3*C*(2,6)H), 128.7 – 129.1 (m, Ar^1*C*(1)), 129.5 (Ar^3C(3,5)H and Ar^2*C*(2,6)H), 129.9 (Ar^3C(4)H), 133.8 (Ar^3*C*(1)), 139.1 – 141.4 (m, Ar^1CF), 144.7 – 147.0 (m, Ar^1CF), 160.8 (Ar^2*C*(4)), 166.8 (*C*(1)); **HRMS** (ESI^+) $C_{25}H_{16}O_3N_2F_4Na$ $[M + Na]^+$ found 491.1013, requires 491.0989 (+4.9 ppm).

Syn-**S17** (30 mg, 13%) as a colourless glass. $[\alpha]_D^{20}$ -151.2 (*c* 0.7 in $CHCl_3$); **Chiral HPLC analysis,** Chiralcel OD-H (90:10 *n*-hexane : IPA, flow rate 1.0 $mLmin^{-1}$, 211 nm, 30 °C) t_R (2*S*,3*S*) 11.7 min, t_R (2*R*,3*R*) 16.1 min, 96:4 er; **IR** ν_{max} (film) 2918 (C-H), 1771 (C=O, ester), 1526, 1514, 1487, 1256, 1180, 957; **^{1}H NMR** (400 MHz, $CDCl_3$) δ_H: 3.75 (3H, s, OC*H*$_3$), 3.99

(1H, dd, *J* 11.3, 5.2, C(3)*H*), 4.62 (1H, d, *J* 11.3, C(2)*H*), 4.64 (1H, d, *J* 5.2, C(4)*H*), 6.73 – 6.80 (2H, m, Ar[2]C(3,5)*H*), 7.00 – 7.13 (3H, m, Ar[1]C(4)*H* and Ar[2]C(2,6)*H*), 7.26 – 7.36 (5H, m, Ar[3]C(2,3,4,5,6)*H*); **^{19}F{^{1}H} NMR** (376 MHz, $CDCl_3$) δ_F: -152.5 – 152.4 (m), -138.3 – -138.1 (m); **^{13}C{^{1}H} NMR** (126 MHz, $CDCl_3$) δ_C: 27.8 (*C*(4)H), 48.0 (*C*(3)H), 51.9 (*C*(2)H), 55.2 (OCH_3), 103.9 (t, *J* 22.6, Ar[1]*C*(4)H), 111.0 (CN), 111.2 (CN), 114.5 (Ar[2]*C*(3,5)H), 124.8 (Ar[2]*C*(1)), 128.6 (Ar[3]*C*(2,6)H), 128.9 – 129.0 (m, Ar[1]*C*(1)), 129.1 (Ar[2]*C*(2,6)H), 129.3 (Ar[3]*C*(4)H), 129.6 (Ar[3]*C*(3,5)H), 133.1 (Ar[3]*C*(1)), 139.1 – 141.5 (m, Ar[1]CF), 144.8 – 147.2 (m, Ar[1]CF), 159.6 (Ar[2]*C*(4)), 169.0 (*C*(1)); **HRMS** (ESI^+) $C_{25}H_{16}O_3N_2F_4Na$ $[M+Na]^+$ found 491.0999, requires 491.0989 (+2.0 ppm).

(2*R*,3*S*)-2,3,5,6-tetrafluorophenyl 4,4-dicyano-2-(4-(dimethylamino)phenyl)-3-phenylbutanoate (21)

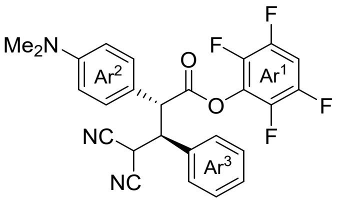

anti-**21**

Following General Procedure E, 2,3,5,6-tetrafluorophenyl 2-(4-dimethylamino)phenyl) acetate **S12** (245 mg, 0.75 mmol, 1.5 eq), 2-benzylidenemalononitrile **2** (78 mg, 0.50 mmol, 1.0 eq), and (4b*R*,11a*S*)-fused-BTM **19** (6.6 mg, 0.025 mmol, 5 mol%) in CPME (0.5 M) gave crude product (90:10 dr) that was purified by flash silica chromatography (hexane:Et_2O 90:10 to 70:30, R_F 0.24 at 70:30) to give the title compound as a colourless solid (166 mg, single diastereoisomer, 69%). **mp** 60–62 °C; $[\alpha]_D^{20}$ -94.0 (*c* 2.0 in $CHCl_3$); **Chiral HPLC analysis,** Chiralcel AD-H (90:10 *n*-hexane : IPA, flow rate 1.0 mLmin^{-1}, 211 nm, 30 °C) t_R (2*R*,3*S*) 11.3 min, t_R (2*S*,3*R*) 14.7 min, 99:1 er; **IR** ν_{max} (film) 2897 (C-H), 1778 (C=O, ester), 1612, 1524, 1487, 1179, 1109, 957; **^{1}H NMR** (400 MHz, $CDCl_3$) δ_H: 3.05 (6H, s, N(C*H*$_3$)$_2$), 3.82 (1H, d, *J* 3.9, C(4)*H*), 3.94 (1H, dd, *J* 12.2, 3.9, C(3)*H*), 4.59 (1H, d, *J* 12.2, C(2)*H*), 6.79 – 6.84 (2H, m, Ar[2]C(3,5)*H*), 6.91 (1H, tt, *J* 9.9, 7.0, Ar[1]C(4)*H*), 7.42 – 7.47 (2H, m, Ar[2]C(2,6)*H*), 7.48 – 7.55 (3H, m, Ar[3]C(3,4,5)*H*), 7.60 – 7.65 (2H, m, Ar[3]C(2,6)*H*); **^{19}F{^{1}H} NMR** (376 MHz, $CDCl_3$) δ_F: -152.7 – 152.5 (m), -139.0 – -138.8 (m); **^{13}C{^{1}H} NMR** (126 MHz, $CDCl_3$) δ_C: 27.8 (*C*(4)H), 40.2 (N(*C*H$_3$)$_2$), 49.1 (*C*(3)H), 52.4 (*C*(2)H), 103.4 (t, *J* 22.8, Ar[1]*C*(4)H), 110.8 (CN),

111.6 (*C*N), 113.2 (Ar[2]*C*(3,5)), 118.9 (Ar[2]*C*(1)), 128.3 (Ar[3]*C*(2,6)), 128.8 – 129.2 (m, Ar[2]*C*(2,6) and Ar[1]*C*(1)), 129.4 (Ar[3]C(3,5)), 129.7 (Ar[3]*C*(4)), 134.0 (Ar[3]*C*(1)), 139.0 – 141.5 (m, Ar[1]CF), 144.6 – 147.0 (m, Ar[1]CF), 151.2 (Ar[2]*C*(4)), 167.0 (*C*(1)); **HRMS** (ESI^+) $C_{26}H_{20}O_2N_3F_4$ $[M+H]^+$ found 482.1485, requires 482.1486 (-0.2 ppm).

(2*R*,3*S*)-2,3,5,6-tetrafluorophenyl 4,4-dicyano-3-phenyl-2-(*m*-tolyl)butanoate (22) and (2*R*,3*R*)- 2,3,5,6-tetrafluorophenyl 4,4-dicyano-3-phenyl-2-(*m*-tolyl)butanoate (S18)

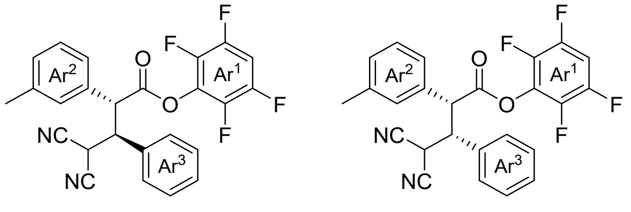

anti-**22** *syn*-**S18**

Following General Procedure E, 2,3,5,6-tetrafluorophenyl 2-(*m*-tolyl)acetate **S15** (224 mg, 0.75 mmol, 1.5 eq), 2-benzylidene malononitrile **2** (78 mg, 0.50 mmol, 1.0 eq), and (4b*R*,11a*S*)-fused-BTM **19** (6.6 mg, 0.025 mmol, 5 mol%) in CPME (1.0 M) gave crude product (90:10 dr) that was purified by flash silica chromatography (hexane:acetone 90:10, R_F 0.13) to give a mixture of diastereoisomers (90:10 dr). The diastereoisomers were then separated by flash silica column chromatography (hexane:Et_2O 90:10 to 80:20) to give:

Anti-**22** (130 mg, 58%) as a colourless solid. **mp** 125–127 °C; $[\alpha]_D^{20}$ -61.7 (*c* 1.2 in $CHCl_3$); **Chiral HPLC analysis,** Chiralpak IA (97:3 *n*-hexane : IPA, flow rate 1.0 mLmin^{-1}, 211 nm, 30 °C) t_R (2*R*,3*S*) 10.1 min, t_R (2*S*,3*R*) 14.6 min, 99:1 er; **IR** ν_{max} (film) 1780 (C=O, ester), 1526, 1487, 1113; **^{1}H NMR** (400 MHz, $CDCl_3$) δ_H: 2.48 (3H, s, C*H*$_3$), 3.74 (1H, d, *J* 3.9, C(4)*H*), 3.99 (1H, dd, *J* 12.2, 3.9, C(3)*H*), 4.68 (1H, d, *J* 12.2, C(2)*H*), 6.93 (1H, tt, *J* 9.9, 7.0, Ar[1]C(4)*H*), 7.31 – 7.37 (1H, m, Ar[2]C(4)*H*), 7.42 – 7.46 (3H, m, Ar[2]C(2,5,6)*H*), 7.49 – 7.56 (3H, m, Ar[3]C(3,4,5)*H*), 7.61 – 7.66 (2H, m, Ar[3]C(2,6)*H*); **^{19}F{^{1}H} NMR** (376 MHz, $CDCl_3$) δ_F: -152.7 – 152.6 (m), -138.8 – -138.6 (m); **^{13}C{^{1}H} NMR** (126 MHz, $CDCl_3$) δ_C: 21.5 (*C*H$_3$), 27.9

(C(4)H), 48.9 (C(3)H), 53.2 (C(2)H), 103.6 (t, *J* 22.7, Ar[1]C(4)H), 110.7 (CN), 111.3 (CN), 125.3 (Ar[2]C(6)H), 128.3 (Ar[3]C(2,6)H), 128.5 – 129.1 (m, Ar[2]C(2)H and Ar[1]C(1)), 129.5 (Ar[3]C(3,5)H), 129.9 (Ar[3]C(4)H), 130.1 (Ar[2]C(5)H), 130.9 (Ar[2]C(4)H), 132.5 (Ar[2]C(1)), 133.7 (Ar[3]C(1)), 139.1 – 141.3 (m, Ar[1]CF), 140.4 (Ar[2]C(3)), 144.6 – 147.0 (m, Ar[1]CF), 166.6 (C(1)); **HRMS** (EI^+) $C_{25}H_{16}O_2N_2F_4$ $[M]^+$ found 452.1133, requires 452.1142 (-2.0 ppm).

Syn-**S18** (15 mg, 7%) as a colourless glass. $[\alpha]_D^{20}$ -116.0 (*c* 0.3 in $CHCl_3$); **Chiral HPLC analysis,** Chiralpak AD-H (97:3 *n*-hexane : IPA, flow rate 1.0 mLmin^{-1}, 211 nm, 30 °C) t_R (2*R*,3*R*) 10.8 min, t_R (2*S*,3*S*) 15.8 min, 91:9 er; **IR** ν_{max} (film) 1771 (C=O, ester), 1526, 1487, 1130; **^{1}H NMR** (400 MHz, $CDCl_3$) δ_H: 2.26 (3H, s, CH_3), 4.01 (1H, dd, *J* 11.3, 5.2, C(3)*H*), 4.62 (1H, d, *J* 11.3, C(2)*H*), 4.68 (1H, d, *J* 5.2, C(4)*H*), 6.94 – 7.16 (5H, m, Ar[2]C(2,4,5,6)*H* and Ar[1]C(4)*H*), 7.26 – 7.36 (5H, m, Ar[3]C(2,3,4,5,6)*H*); **^{19}F{^{1}H} NMR** (376 MHz, $CDCl_3$) δ_F: -152.5 – 152.4 (m), -138.2 – -138.1 (m); **^{13}C{^{1}H} NMR** (126 MHz, $CDCl_3$) δ_C: 21.3 (CH_3), 27.8 (C(4)H), 48.0 (C(3)H), 52.6 (C(2)H), 103.9 (t, *J* 22.7, Ar[1]C(4)H), 111.1 (CN), 111.2 (CN), 125.5 (Ar[2]C(6)H), 128.6 (Ar[3]C(2,6)H), 128.8 – 129.0 (m, Ar[2]C(2)H and Ar[1]C(1)), 129.0 – 129.1 (m, Ar[3]C(3,4,5)H), 129.3 (Ar[2]C(5)H), 129.5 (Ar[2]C(4)H), 132.8 (Ar[2]C(1)), 133.1 (Ar[3]C(1)), 138.9 (Ar[2]C(3)), 139.2 – 141.5 (m, Ar[1]CF), 144.8 – 147.2 (m, Ar[1]CF), 168.9 (C(1)); **HRMS** (EI^+) $C_{25}H_{16}O_2N_2F_4$ $[M]^+$ found 452.1139, requires 452.1142 (-0.7 ppm).

(2*R*,3*S*)-2,3,5,6-tetrafluorophenyl 4,4-dicyano-3-phenyl-2-(*o*-tolyl)butanoate (23) and (2*R*,3*R*)- 2,3,5,6-tetrafluorophenyl 4,4-dicyano-3-phenyl-2-(*o*-tolyl)butanoate (S19)

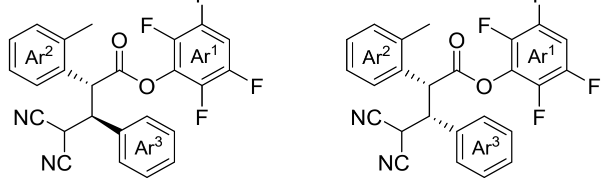

anti-**23** *syn*-**S19**

Following General Procedure E, 2,3,5,6-tetrafluorophenyl 2-(*o*-tolyl)acetate **S16** (224 mg, 0.75 mmol, 1.5 eq), 2-benzylidene malononitrile **2** (78 mg, 0.50 mmol, 1.0 eq), and (4b*R*,11a*S*)-fused-BTM **19** (6.6 mg, 0.025 mmol, 5 mol%) in CPME (1.0 M) gave crude product that was purified by flash silica chromatography (hexane:acetone 90:10, R_F 0.17)

to give a mixture of diastereoisomers (80:20 dr). The diastereoisomers were then separated by flash silica column chromatography (hexane:Et_2O 80:20) to give:

Anti-**23** (72 mg, 32%) as a colourless solid. **mp** 93–95 °C; $[\alpha]_D^{20}$ -38.3 (*c* 1.2 in $CHCl_3$); **Chiral HPLC analysis,** Chiralcel OD-H (99.5:0.5 *n*-hexane : IPA, flow rate 1.0 $mLmin^{-1}$, 211 nm, 30 °C) t_R (2*S*,3*R*) 40.6 min, t_R (2*R*,3*S*) 47.8 min, 91:9 er; **IR** ν_{max} (film) 1780 (C=O, ester), 1526, 1487, 1179, 1103, 957; **^{1}H NMR** (400 MHz, $CDCl_3$) δ_H: 2.75 (3H, s, C*H*$_3$), 3.90 (1H, d, *J* 3.9, C(4)*H*), 4.18 (1H, dd, *J* 12.2, 3.9, C(3)*H*), 5.07 (1H, d, *J* 12.2, C(2)*H*), 6.92 (1H, tt, *J* 9.8, 7.0, Ar^1C(4)*H*), 7.38 – 7.42 (3H, m, Ar^2C(3,4,5)*H*), 7.48 – 7.62 (4H, m, Ar^2C(6)*H* and Ar^3C(3,4,5)*H*), 7.66 – 7.70 (2H, m, Ar^3C(2,6)*H*); **^{19}F{^{1}H} NMR** (376 MHz, $CDCl_3$) δ_F: -152.9 – 152.8 (m), -138.8 – -138.6 (m); **^{13}C{^{1}H} NMR** (126 MHz, $CDCl_3$) δ_C: 19.9 (CH_3), 27.3 (*C*(4)H), 48.0 (*C*(2)H), 48.6 (*C*(3)H), 103.5 (t, *J* 22.8, Ar^1*C*(4)H), 111.2 (*C*N), 111.4 (*C*N), 126.0 (Ar^2*C*(6)H), 127.9 (Ar^2*C*(5)H), 128.4 (Ar^3*C*(2,6)H), 128.6 – 129.1 (m, Ar^1*C*(1)), 129.5 (Ar^3*C*(3,5)H), 129.7 (Ar^3*C*(4)H), 129.9 (Ar^2*C*(4)H), 130.8 (Ar^2*C*(1)), 132.3 (Ar^2*C*(3)H), 133.7 (Ar^3*C*(1)), 138.2 (Ar^2*C*(2)), 139.0 – 141.4 (m, Ar^1*C*F), 144.6 – 146.9 (m, Ar^1*C*F), 166.8 (*C*(1)); **HRMS** (EI^+) $C_{25}H_{16}O_2N_2F_4$ $[M]^+$ found 452.1134, requires 452.1142 (-1.8 ppm).

Syn-**S19** (20 mg, 9%) as a colourless glass. $[\alpha]_D^{20}$ -102.0 (*c* 0.5 in $CHCl_3$); **Chiral HPLC analysis,** Chiralcel OD-H (93:7 *n*-hexane : IPA, flow rate 1.5 $mLmin^{-1}$, 211 nm, 30 °C) t_R (2*S*,3*S*) 7.3 min, t_R (2*R*,3*R*) 11.4 min, 83:17 er; **IR** ν_{max} (film) 2922 (C-H), 1771 (C=O, ester), 1526, 1487, 1456, 1273, 1180, 1132, 1103, 959; **^{1}H NMR** (400 MHz, $CDCl_3$) δ_H: 2.34 (3H, s, C*H*$_3$), 4.09 (1H, dd, *J* 11.6, 5.0, C(3)*H*), 4.79 (1H, d, *J* 5.0, C(4)*H*), 4.93 (1H, d, *J* 11.6, C(2)*H*), 6.99 – 7.09 (2H, m, Ar^2C(3)*H* and Ar^1C(4)*H*), 7.10 – 7.19 (2H, m, Ar^2C(4,5)*H*), 7.24 – 7.33 (6H, m, Ar^2C(6)*H* and Ar^3C(2,3,4,5,6)*H*); **^{19}F{^{1}H} NMR** (376 MHz, $CDCl_3$) δ_F: -152.6 – 152.5 (m), -138.3 – -138.2 (m); **^{13}C{^{1}H} NMR** (126 MHz, $CDCl_3$) δ_C: 19.8 (CH_3), 27.7 (*C*(4)H), 47.8 (*C*(3)H), 48.0 (*C*(2)H), 103.9 (t, *J* 22.7, Ar^1C(4)H), 111.1 (CN), 111.3 (CN), 126.8 (Ar^2*C*(5,6)H), 128.3 (Ar^3*C*(2,6)H), 128.6 (Ar^2*C*(4)H), 128.7 – 129.0 (m, Ar^1*C*(1)), 129.0 (Ar^3*C*(3,5)H), 129.3 (Ar^3*C*(4)H), 131.2 (Ar^2*C*(3)H), 131.8 (Ar^2*C*(1)), 133.3 (Ar^3*C*(1)), 136.7 (Ar^2*C*(2)), 139.1 – 141.5 (m, Ar^1CF), 144.8 – 147.1 (m, Ar^1CF), 169.2 (*C*(1)); **HRMS** (EI^+) $C_{25}H_{16}O_2N_2F_4$ $[M]^+$ found 452.1142, requires 452.1142 (±0.0 ppm).

(2*R*,3*S*)-2,3,5,6-tetrafluorophenyl 4,4-dicyano-3-phenyl-2-(thiophen-3-yl)butanoate (24) and (2*R*,3*R*)- 2,3,5,6-tetrafluorophenyl 4,4-dicyano-3-phenyl-2-(thiophen-3-yl)butanoate (S20)

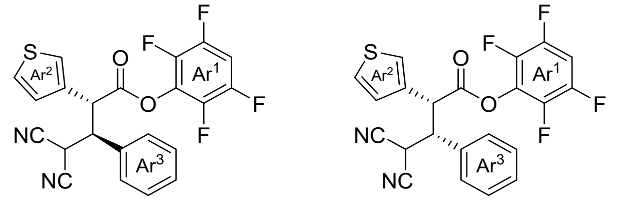

Following General Procedure E, 2,3,5,6-tetrafluorophenyl 2-(thiophen-3-yl)acetate **S13** (218 mg, 0.75 mmol, 1.5 eq), 2-benzylidene malononitrile **2** (78 mg, 0.50 mmol, 1.0 eq), and (4b*R*,11a*S*)-fused-BTM **19** (6.6 mg, 0.025 mmol, 5 mol%) in CPME (1.0 M) gave crude product that was purified by flash silica column chromatography (hexane:acetone 90:10 to 85:15, R_F 0.11 at 90:10) to give a mixture of diastereoisomers (86:14 dr). The diastereoisomers were then separated by flash silica column chromatography (hexane:Et_2O 80:20 to 70:30) to give:

Anti-**24** (149 mg, 67%) as a colourless solid. **mp** 93–95 °C; $[\alpha]_D^{20}$ -54.4 (*c* 1.0 in $CHCl_3$); **Chiral HPLC analysis,** Chiralcel OD-H (97:3 *n*-hexane : IPA, flow rate 1.0 mLmin^{-1}, 211 nm, 30 °C) t_R (2*R*,3*S*) 27.6 min, t_R (2*S*,3*R*) 31.2 min, 97:3 er; **IR** ν_{max} (film) 2918 (C-H), 2156, 1973, 1782 (C=O, ester), 1526, 1487, 1180, 1115, 959; **^{1}H NMR** (400 MHz, $CDCl_3$) δ_H: 3.81 (1H, d, *J* 4.0, C(4)*H*), 3.99 (1H, dd, *J* 12.2, 4.0, C(3)*H*), 4.92 (1H, d, *J* 12.2, C(2)*H*), 6.94 (1H, tt, *J* 9.9, 7.0, Ar^{1}C(4)*H*), 7.35 (1H, dd, *J* 5.1, 1.4, Ar^{2}C(4)*H*), 7.48 – 7.59 (4H, m, Ar^{2}C(5)*H* and Ar^{3}C(3,4,5)*H*), 7.60 – 7.64 (2H, m, Ar^{3}C(2,6)*H*), 7.66 (1H, dd, *J* 3.0, 1.4, Ar^{2}C(2)*H*); **^{19}F{^{1}H} NMR** (376 MHz, $CDCl_3$) δ_F: -152.7 – 152.6 (m), -138.6 – -138.5 (m); **^{13}C{^{1}H} NMR** (126 MHz, $CDCl_3$) δ_C: 28.0 (*C*(4)H), 48.8 (*C*(2)H), 48.9 (*C*(3)H), 103.7 (t, *J* 22.7, Ar^1*C*(4)H), 110.7 (CN), 111.2 (CN), 125.9 (Ar^2*C*(2)H), 126.0 (Ar^2*C*(4)H), 128.2 (Ar^3*C*(2,6)H), 128.5 – 129.0 (m, Ar^1*C*(1)), 129.0 (Ar^2*C*(5)H), 129.6 (Ar^3*C*(3,5)H), 130.0 (Ar^3*C*(4)H), 132.6 (Ar^2*C*(3)), 133.5 (Ar^3*C*(1)), 139.0 – 141.4 (m, Ar^{1}CF), 144.6 – 147.0 (m, Ar^{1}CF), 166.2 (*C*(1)); **HRMS** (ESI^+) $C_{22}H_{13}O_2N_2F_4S$ $[M+H]^+$ found 445.0612, requires 445.0628 (-3.6 ppm).

Syn-**S20** (20 mg, 9%) as a colourless glass. $[\alpha]_D^{20}$ -85.0 (*c* 0.8 in $CHCl_3$); **Chiral HPLC analysis,** Chiralpak IB (98:2 *n*-hexane : IPA, flow rate 1.0 mLmin^{-1}, 211 nm, 30 °C) t_R (2*S*,3*S*) 22.6 min, t_R (2*R*,3*R*) 29.1 min, 87:13 er; **IR** ν_{max} (film) 2920 (C-H), 1773 (C=O, ester), 1526, 1487, 1456, 1179, 1128, 1090, 957; **^{1}H NMR** (500 MHz, $CDCl_3$) δ_H: 4.00 (1H, dd, *J* 10.8, 5.6, C(3)*H*), 4.58 (1H, d, *J* 5.6, C(4)*H*), 4.85 (1H, d, *J* 10.8, C(2)*H*), 6.87 (1H, dd, *J* 5.1, 1.3, Ar^{2}C(4)*H*), 7.07 (1H, tt, *J* 9.8, 7.1, Ar^{1}C(4)*H*), 7.12 (1H, dd, *J* 3.0, 1.3, Ar^{2}C(2)*H*), 7.24 (1H, dd, *J* 5.1, 3.0, Ar^{2}C(5)*H*), 7.27 – 7.32 (2H, m, Ar^{3}C(2,6)*H*), 7.33 – 7.39 (3H, m, Ar^{3}C(3,4,5)*H*); **^{19}F{^{1}H} NMR** (376 MHz, $CDCl_3$) δ_F: -152.5 – 152.4 (m), -138.1 – -137.9 (m); **^{13}C{^{1}H} NMR** (126 MHz, $CDCl_3$) δ_C: 27.7 (*C*(4)H), 48.0 (*C*(3)H), 48.2 (*C*(2)H), 104.0 (t, *J* 22.7, Ar^1*C*(4)H), 110.9 (*C*N), 111.1 (*C*N), 125.0 (Ar^2*C*(2)H), 126.5 (Ar^2*C*(4)H), 127.0 (Ar^2*C*(5)H), 128.5 (Ar^3*C*(2,6)H), 128.7 – 129.1 (m, Ar^1*C*(1)), 129.2 (Ar^3*C*(3,5)H), 129.5 (Ar^3*C*(4)H), 132.3 (Ar^2*C*(3)), 133.1 (Ar^3*C*(1)), 139.2 – 141.5 (m, Ar^{1}CF), 144.8 – 147.2 (m, Ar^{1}CF), 168.2 (*C*(1)); **HRMS** (ESI^+) $C_{22}H_{13}O_2N_2F_4S$ $[M+H]^+$ found 445.0629, requires 445.0628 (+0.2 ppm).

2,3,5,6-tetrafluorophenyl (*S*,*E*)-2-((*S*)-2,2-dicyano-1-phenylethyl)pent-3-enoate (25) and 2,3,5,6-tetrafluorophenyl (*S*,*E*)-2-((*R*)-2,2-dicyano-1-phenylethyl)pent-3-enoate (S21)

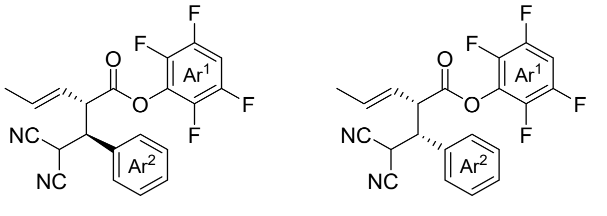

anti-**25** *syn*-**S21**

Following General Procedure E, 2,3,5,6-tetrafluorophenyl (*E*)-pent-3-enoate **S14** (186 mg, 0.75 mmol, 1.5 eq), 2-benzylidenemalononitrile **2** (78 mg, 0.50 mmol, 1.0 eq), and (4b*R*,11a*S*)-fused-BTM **19** (6.6 mg, 0.025 mmol, 5 mol%) in CPME (1.0 M) gave crude product (77:23 dr) that was purified by flash silica column chromatography (hexane:Et_2O 90:10 to 80:20) to give:

Anti-**25** (83 mg, 41%) as a colourless solid. **mp** 84–86 °C; $[\alpha]_D^{20}$ -95.2 (*c* 1.0 in $CHCl_3$); **Chiral HPLC analysis,** Chiralcel OD-H (95:5 *n*-hexane : IPA, flow rate 1.0 mLmin^{-1}, 211 nm, 30 °C) t_R (2*S*,3*S*) 12.3 min, t_R (2*R*,3*R*) 19.9 min, 99:1 er; **IR** ν_{max} (film) 2920 (C-H), 1780 (C=O, ester), 1526, 1487, 1179, 1111, 1065, 959; **^{1}H NMR** (400 MHz, $CDCl_3$) δ_H: 1.92 (3H, dd, *J* 6.6, 1.7, C*H*$_3$), 3.69 (1H, dd, *J* 11.8, 4.0, C(3)*H*), 4.16 (1H, dd, *J* 11.8, 9.6, C(2)*H*), 4.26 (1H, d, *J* 4.0,

C(4)*H*), 5.62 (1H, ddq, *J* 14.9, 9.6, 1.7, C*H*=CHCH$_3$), 6.23 – 6.35 (1H, m, CH=C*H*CH$_3$), 6.96 (1H, tt, *J* 9.9, 7.0, Ar^{1}C(4)*H*), 7.43 – 7.54 (5H, m, Ar^{2}C(2,3,4,5,6)*H*); **^{19}F{^{1}H} NMR** (376 MHz, CDCl$_3$) δ_F: -152.9 – 152.8 (m), -138.8 – -138.6 (m); **^{13}C{^{1}H} NMR** (126 MHz, CDCl$_3$) δ_C: 18.3 (*C*H$_3$), 27.9 (*C*(4)H), 47.2 (*C*(3)H), 52.0 (*C*(2)H), 103.6 (t, *J* 22.7, Ar^1*C*(4)H), 110.8 (*C*N), 111.4 (*C*N), 123.2 (*C*H=CHCH$_3$), 128.2 (Ar^2*C*(2,6)H), 128.7 – 129.0 (m, Ar^1*C*(1)), 129.5 (Ar^2*C*(3,5)H), 129.8 (Ar^{2}C(4)H), 133.5 (Ar^2*C*(1)), 136.9 (CH=*C*HCH$_3$), 139.0 – 141.4 (m, Ar^{1}CF), 144.6 – 147.0 (m, Ar^{1}CF), 166.6 (*C*(1)); **HRMS** (ESI^+) $C_{21}H_{14}O_2N_2F_4Na$ $[M+Na]^+$ found 425.0900, requires 425.0884 (+3.8 ppm).

Syn-**S21** (23 mg, 11%) as a colourless glass. $[\alpha]_D^{20}$ -88.0 (*c* 0.3 in CHCl$_3$); **Chiral HPLC analysis,** Chiralcel OD-H (99:1 *n*-hexane : IPA, flow rate 1.5 mLmin^{-1}, 211 nm, 40 °C) t_R (2*R*,3*S*) 16.4 min, t_R (2*S*,3*R*) 23.3 min, 97:3 er; **IR** ν_{max} (film) 2920 (C-H), 1773 (C=O, ester), 1526, 1487, 1179, 1109, 957; **^{1}H NMR** (400 MHz, CDCl$_3$) δ_H: 1.70 (3H, dd, *J* 6.5, 1.7, C*H*$_3$), 3.82 (1H, dd, *J* 8.9, 7.1, C(3)*H*), 4.12 (1H, dd, *J* 8.9, 8.9, C(2)*H*), 4.33 (1H, d, *J* 7.1, C(4)*H*), 5.36 (1H, ddq, *J* 15.3, 8.9, 1.7, C*H*=CHCH$_3$), 5.82 – 5.94 (1H, m, CH=C*H*CH$_3$), 7.07 (1H, tt, *J* 9.8, 7.1, Ar^{1}C(4)*H*), 7.32 – 7.39 (2H, m, Ar^{2}C(2,6)*H*), 7.41 – 7.51 (3H, m, Ar^{2}C(3,4,5)*H*); **^{19}F{^{1}H} NMR** (376 MHz, CDCl$_3$) δ_F: -152.6 – 152.4 (m), -138.2 – -138.1 (m); **^{13}C{^{1}H} NMR** (126 MHz, CDCl$_3$) δ_C: 18.1 (*C*H$_3$), 27.5 (*C*(4)H), 47.4 (*C*(3)H), 50.1 (*C*(2)H), 103.9 (t, *J* 22.7, Ar^1*C*(4)H), 110.9 (*C*N), 111.3 (*C*N), 122.0 (*C*H=CHCH$_3$), 128.7 (Ar^2*C*(2,6)H), 128.9 – 129.2 (m, Ar^1*C*(1)), 129.3 (Ar^2*C*(3,5)H), 129.6 (Ar^2*C*(4)H), 132.8 (Ar^2*C*(1)), 134.7 (CH=*C*HCH$_3$), 139.2 – 141.6 (m, Ar^{1}CF), 144.8 – 147.2 (m, Ar^{1}CF), 168.1 (*C*(1)); **HRMS** (ESI^+) $C_{21}H_{14}O_2N_2F_4Na$ $[M+Na]^+$ found 425.0881, requires 425.0884 (-0.7 ppm).

(2*R*,3*S*)-2,3,5,6-tetrafluorophenyl 4,4-dicyano-3-(4-(trifluoromethyl)phenyl)-2-(*p*-tolyl)butanoate (26) and (2*R*,3*R*)- 2,3,5,6-tetrafluorophenyl 4,4-dicyano-3-(4-(trifluoromethyl)phenyl)-2-(*p*-tolyl)butanoate (S22)

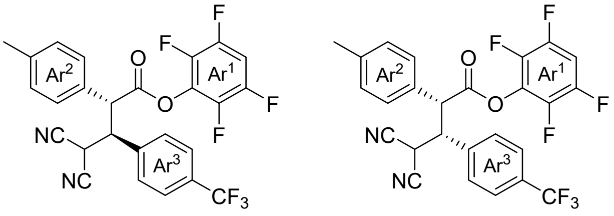

anti-**26** *syn*-**S22**

Following General Procedure E, 2,3,5,6-tetrafluorophenyl 2-(p-tolyl)acetate **9** (224 mg, 0.75 mmol, 1.5 eq), 2-(4-(trifluoromethyl)benzylidene)malononitrile **S9** (111 mg, 0.50 mmol, 1.0 eq), and (4b*R*,11a*S*)-fused-BTM **19** (6.6 mg, 0.025 mmol, 5 mol%) in CPME (1.0 M) gave crude product (85:15 dr) that was purified by flash silica chromatography (hexane:acetone 90:10, R_F 0.21) to give a mixture of diastereoisomers (85:15 dr). The diastereoisomers were then separated by flash silica column chromatography (hexane:EtOAc 95:5 to 90:10) to give:

Anti-**26** (162 mg, 62%) as a colourless solid. **mp** 139–141 °C; $[\alpha]_D^{20}$ -52.8 (*c* 1.3 in $CHCl_3$); **Chiral HPLC analysis,** Chiralcel OD-H (93:7 *n*-hexane : IPA, flow rate 2.0 mLmin^{-1}, 211 nm, 30 °C) t_R (2*S*,3*R*) 7.0 min, t_R (2*R*,3*S*) 12.6 min, 98:2 er; **IR** ν_{max} (film) 1780 (C=O, ester), 1528, 1487, 1327, 1171, 1113, 1070, 957; **^{1}H NMR** (400 MHz, $CDCl_3$) δ_H: 2.46 (3H, s, C*H*$_3$), 3.77 (1H, d, *J* 4.0, C(4)*H*), 4.05 (1H, dd, *J* 12.2, 4.0, C(3)*H*), 4.69 (1H, d, *J* 12.2, C(2)*H*), 6.94 (1H, tt, *J* 9.8, 7.0, Ar^{1}C(4)*H*), 7.35 – 7.41 (2H, m, Ar^{2}C(3,5)*H*), 7.49 – 7.54 (2H, m, Ar^{2}C(2,6)*H*), 7.75 – 7.84 (4H, m, Ar^{3}C(2,3,5,6)*H*); **^{19}F{^{1}H} NMR** (376 MHz, $CDCl_3$) δ_F: -153.1 – 152.9 (m), -138.5 – -138.3 (m), -62.9 (s); **^{13}C NMR** (126 MHz, $CDCl_3$) δ_C: 21.2 (*C*H$_3$), 27.5 (*C*(4)H), 48.7 (*C*(3)H), 52.7 (*C*(2)H), 103.7 (t, *J* 22.7, Ar^1*C*(4)H), 110.3 (CN), 111.0 (CN), 123.7 (q, *J* 272.4, *C*F$_3$), 126.5 (q, *J* 3.7, Ar^{3}C(3,5)), 128.0 (Ar^2*C*(2,6)), 128.5 – 128.8 (m, Ar^1*C*(1)), 128.9 (Ar^3*C*(2,6)), 129.0 (Ar^2*C*(1)), 131.1 (Ar^2*C*(3,5)), 132.1 (q, *J* 32.9 Ar^3*C*(4)), 137.8 (Ar^3*C*(1)), 139.0 – 141.3 (m, Ar^{1}CF), 140.6 (Ar^{2}C(4)), 144.6 – 147.0 (m, Ar^{1}CF), 166.5 (*C*(1)); **HRMS** (ESI^-) $C_{26}H_{14}O_2N_2F_7$ $[M-H]^-$ found 519.0956, requires 519.0949 (+1.4 ppm).

Syn-**S22** (19 mg, 7%) as a colourless glass. $[\alpha]_D^{20}$ -95.3 (*c* 0.9 in $CHCl_3$); **Chiral HPLC analysis,** Chiralcel OD-H (95:5 *n*-hexane : IPA, flow rate 1.0 $mLmin^{-1}$, 211 nm, 30 °C) t_R (2*S*,3*S*) 10.7 min, t_R (2*R*,3*R*) 24.6 min, 87:13 er; **IR** ν_{max} (film) 2924 (C-H), 1771 (C=O, ester), 1528, 1487, 1325, 1179, 1130, 1115, 1070, 959; **^{1}H NMR** (400 MHz, $CDCl_3$) δ_H: 2.28 (3H, s, C*H*$_3$), 4.09 (1H, dd, *J* 11.4, 5.1, C(3)*H*), 4.62 (1H, d, *J* 11.4, C(2)*H*), 4.69 (1H, d, *J* 5.1, C(4)*H*), 7.01 – 7.12 (5H, m, Ar^{2}C(2,3,5,6)*H* and Ar^{1}C(4)*H*), 7.40 – 7.47 (2H, m, Ar^{3}C(2,6)*H*), 7.57 – 7.63 (2H, m, Ar^{3}C(3,5)*H*); **^{19}F{^{1}H} NMR** (376 MHz, $CDCl_3$) δ_F: -152.6 – 152.4 (m), -138.1 – -138.0 (m), -62.9 (s); **^{13}C NMR** (126 MHz, $CDCl_3$) δ_C: 21.1 (*C*H$_3$), 27.6 (*C*(4)H), 47.7 (*C*(3)H), 52.0 (*C*(2)H), 104.0 (t, *J* 22.7, Ar^1*C*(4)H), 110.7 (*C*N), 110.8 (*C*N), 123.6 (q, *J* 272.5, *C*F$_3$), 126.1 (q, *J* 3.7, Ar^{3}C(3,5)), 128.1 (Ar^2*C*(2,6)), 129.1 – 129.4 (m, Ar^3*C*(2,6), Ar^2*C*(1), and Ar^1*C*(1)), 130.1 (Ar^2*C*(3,5)), 131.5 (q, *J* 32.8 Ar^3*C*(4)), 137.1 (Ar^3*C*(1)), 139.1 (Ar^2*C*(4)), 139.2 – 141.4 (m, Ar^{1}CF), 144.8 – 147.1 (m, Ar^{1}CF), 168.7 (*C*(1)); **HRMS** (ESI^+) $C_{26}H_{15}O_2N_2F_7Na$ $[M+Na]^+$ found 543.0908, requires 543.0914 (-1.1 ppm).

(2*R*,3*S*)-2,3,5,6-tetrafluorophenyl 4,4-dicyano-3-(4-nitrophenyl)-2-(*p*-tolyl)butanoate (27) and (2*R*,3*R*)-2,3,5,6-tetrafluorophenyl 4,4-dicyano-3-(4-nitrophenyl)-2-(*p*-tolyl)butanoate (S23)

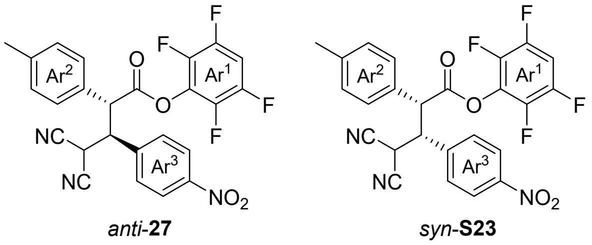

anti-**27** *syn*-**S23**

Following General Procedure E, 2,3,5,6-tetrafluorophenyl 2-(p-tolyl)acetate **9** (224 mg, 0.75 mmol, 1.5 eq), 2-(4-nitrobenzylidene)malononitrile **S7** (100 mg, 0.50 mmol, 1.0 eq), and (4b*R*,11a*S*)-fused-BTM **19** (6.6 mg, 0.025 mmol, 5 mol%) in CH_2Cl_2 (1.0 M) gave crude product (68:32 dr) that was purified by flash silica chromatography (hexane:EtOAc 95:5 to 80:20) to give:

Anti-**27** (147 mg, 59%) as a colourless solid. **mp** 73–75 °C; $[\alpha]_D^{20}$ -42.7 (*c* 1.5 in $CHCl_3$); **Chiral HPLC analysis,** Chiralcel OD-H (93:7 *n*-hexane : IPA, flow rate 1.0 $mLmin^{-1}$, 211 nm, 40 °C) t_R (2*S*,3*R*) 25.3 min, t_R (2*R*,3*S*) 46.2 min, 99:1 er; **IR** ν_{max} (film) 2903 (C-H), 1780 (C=O, ester), 1526, 1487, 1350, 1179, 1113, 957; **^{1}H NMR** (400 MHz, $CDCl_3$) δ_H: 2.46 (3H, s, C*H*$_3$),

3.79 (1H, d, *J* 4.0, C(4)*H*), 4.10 (1H, dd, *J* 12.2, 4.0, C(3)*H*), 4.68 (1H, d, *J* 12.2, C(2)*H*), 6.95 (1H, tt, *J* 9.8, 7.0, $Ar^1C(4)H$), 7.35 – 7.41 (2H, m, $Ar^2C(3,5)H$), 7.46 – 7.54 (2H, m, $Ar^2C(2,6)H$), 7.81 – 7.88 (2H, m, $Ar^3C(2,6)H$), 8.37 – 8.44 (2H, m, $Ar^3C(3,5)H$); **$^{19}F\{^1H\}$ NMR** (376 MHz, $CDCl_3$) δ_F: -153.1 – 152.9 (m), -138.2 – -138.1 (m); **$^{13}C\{^1H\}$ NMR** (126 MHz, $CDCl_3$) δ_C: 21.3 (*C*H_3), 27.3 (*C*(4)), 48.6 (*C*(3)), 52.6 (*C*(2)), 103.9 (t, *J* 22.7, $Ar^1C(4)$), 110.2 (*C*N), 110.8 (*C*N), 124.6 ($Ar^3C(3,5)$), 128.1 ($Ar^2C(2,6)$), 128.6 – 128.8 (m, $Ar^2C(1)$ and $Ar^1C(1)$), 129.6 ($Ar^3C(2,6)$), 131.2 ($Ar^2C(3,5)$), 138.9 – 141.4 (m, Ar^1CF), 140.8 ($Ar^2C(4)$), 140.8 ($Ar^3C(1)$), 144.5 – 147.2 (m, Ar^1CF), 148.8 ($Ar^3C(4)$), 166.5 (*C*(1)). **HRMS** (ESI^+) $C_{25}H_{15}O_4N_3F_4{}^{23}Na$ $[M+Na]^+$ found 520.0894, requires 520.0891 (+0.6 ppm).

Syn-**S23** (54 mg, 22%) as a colourless glass. $[\alpha]_D^{20}$ -160.4 (*c* 1.4 in $CHCl_3$); **Chiral HPLC analysis,** Chiralcel OD-H (93:7 *n*-hexane : IPA, flow rate 1.0 mLmin^{-1}, 211 nm, 40 °C) t_R (2*S*,3*S*) 16.9 min, t_R (2*R*,3*R*) 34.7 min, 99:1 er; **IR** ν_{max} (film) 2926 (C-H), 1771 (C=O, ester), 1526, 1487, 1350, 1180, 1134, 1115, 959; **^1H NMR** (400 MHz, $CDCl_3$) δ_H: 2.28 (3H, s, CH_3), 4.17 (1H, dd, *J* 11.4, 5.2, C(3)*H*), 4.63 (1H, d, *J* 11.4, C(2)*H*), 4.73 (1H, d, *J* 5.2, C(4)*H*), 7.02 – 7.11 (5H, m, $Ar^1C(4)H$ and $Ar^2C(2,3,5,6)H$), 7.48 – 7.53 (2H, m, $Ar^3C(2,6)H$), 8.17 – 8.22 (2H, m, $Ar^3C(3,5)H$); **$^{19}F\{^1H\}$ NMR** (376 MHz, $CDCl_3$) δ_F: -152.6 – 152.4 (m), -138.0 – -137.9 (m); **$^{13}C\{^1H\}$ NMR** (126 MHz, $CDCl_3$) δ_C: 21.1 (*C*H_3), 27.4 (*C*(4)), 47.6 (*C*(3)), 51.9 (*C*(2)), 104.1 (t, *J* 22.7, $Ar^1C(4)$), 110.5 (*C*N), 110.7 (*C*N), 124.3 ($Ar^3C(3,5)$), 128.1 ($Ar^2C(2,6)$), 128.7 – 129.0 (m, $Ar^1C(1)$), 129.1 ($Ar^2C(1)$), 129.9 ($Ar^3C(2,6)$), 130.2 ($Ar^2C(3,5)$), 139.0 – 141.4 (m, Ar^1CF), 139.4 ($Ar^2C(4)$), 140.2 ($Ar^3C(1)$), 144.8 – 147.2 (m, Ar^1CF), 148.3 ($Ar^3C(4)$), 168.5 (*C*(1)). **HRMS** (ESI^+) $C_{25}H_{15}O_4N_3F_4{}^{23}Na$ $[M+Na]^+$ found 520.0890, requires 520.0891 (-0.2 ppm).

(2*R*,3*S*)-2,3,5,6-tetrafluorophenyl 4,4-dicyano-3-(4-fluorophenyl)-2-(*p*-tolyl)butanoate (28)

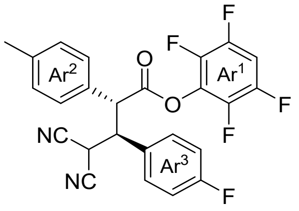

anti-**28**

Following General Procedure E, 2,3,5,6-tetrafluorophenyl 2-(p-tolyl)acetate **9** (224 mg, 0.75 mmol, 1.5 eq), 2-(4-fluorobenzylidene)malononitrile **S5** (86 mg, 0.50 mmol, 1.0 eq), and (4b*R*,11a*S*)-fused-BTM **19** (6.6 mg, 0.025 mmol, 5 mol%) in CPME (1.0 M) gave crude product (95:5 dr) that was purified by flash silica chromatography (hexane:acetone 90:10, R_F 0.31 at 80:20) to give a mixture of diastereoisomers (95:5 dr). The mixture of diastereoisomers was then subjected to flash silica column chromatography (hexane:Et_2O 90:10 to 80:20, R_F 0.18 at 80:20) to afford the title compound (152 mg, 65%, single major diastereoisomer) as a colourless solid. **mp** 165–167 °C; $[\alpha]_D^{20}$ -64.0 (*c* 1.0 in $CHCl_3$); **Chiral HPLC analysis,** Chiralcel OD-H (95:5 *n*-hexane : IPA, flow rate 1.0 mLmin^{-1}, 211 nm, 30 °C) t_R (2*S*,3*R*) 16.4 min, t_R (2*R*,3*S*) 23.4 min, 99:1 er; **IR** ν_{max} (film) 1780 (C=O, ester), 1526, 1514, 1487, 1105, 959; **^{1}H NMR** (500 MHz, $CDCl_3$) δ_H: 2.45 (3H, s, C*H*$_3$), 3.72 (1H, d, *J* 3.9, C(4)*H*), 3.98 (1H, dd, *J* 12.2, 3.9, C(3)*H*), 4.62 (1H, d, *J* 12.2, C(2)*H*), 6.94 (1H, tt, *J* 9.8, 7.0, Ar^{1}C(4)*H*), 7.19 – 7.26 (2H, m, Ar^{3}C(3,5)*H*), 7.33 – 7.39 (2H, m, Ar^{2}C(3,5)*H*), 7.48 – 7.53 (2H, m, Ar^{2}C(2,6)*H*), 7.59 – 7.66 (2H, m, Ar^{3}C(2,6)*H*); **^{19}F{^{1}H} NMR** (470 MHz, $CDCl_3$) δ_F: -152.9 – 152.8 (m), -138.6 – -138.5 (m), -111.0 (s); **^{13}C NMR** (126 MHz, $CDCl_3$) δ_C: 21.3 (CH_3), 27.8 (*C*(4)H), 48.3 (*C*(3)H), 53.0 (*C*(2)H), 103.7 (t, *J* 22.7, Ar^1*C*(4)H), 110.5 (CN), 111.2 (CN), 116.7 (d, *J* 21.9, Ar^{3}C(3,5)H), 128.0 (Ar^2*C*(2,6)H), 128.6 – 129.0 (m, Ar^1*C*(1)), 129.3 (Ar^2*C*(1)), 129.6 (d, *J* 3.3, Ar^3*C*(1)), 130.2 (d, *J* 8.4, Ar^3*C*(2,6)H), 131.0 (Ar^2*C*(3,5)H), 139.0 – 141.3 (m, Ar^{1}CF), 140.4 (Ar^2*C*(4)), 144.6 – 147.0 (m, Ar^{1}CF), 163.5 (d, *J* 249.7, Ar^3*C*(4)), 166.6 (*C*(1)); **HRMS** (EI^+) $C_{25}H_{15}O_2N_2F_5$ $[M]^+$ found 470.1058, requires 470.1048 (+2.1 ppm).

(2*R*,3*S*)-2,3,5,6-tetrafluorophenyl 4,4-dicyano-3-(4-chlorophenyl)-2-(*p*-tolyl)butanoate (29) and (2*R*,3*R*)- 2,3,5,6-tetrafluorophenyl 4,4-dicyano-3-(4-chlorophenyl)-2-(*p*-tolyl)butanoate (S24)

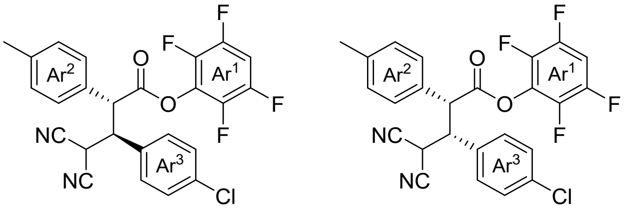

anti-**29** *syn*-**S24**

Following General Procedure E, 2,3,5,6-tetrafluorophenyl 2-(*p*-tolyl)acetate **9** (224 mg, 0.75 mmol, 1.5 eq), 2-(4-chlorobenzylidene)malononitrile **S8** (94 mg, 0.50 mmol, 1.0 eq), and (4b*R*,11a*S*)-fused-BTM **19** (6.6 mg, 0.025 mmol, 5 mol%) in CPME (0.5 M) gave crude product (80:20 dr) that was purified by flash silica chromatography (hexane:acetone 90:10, R_F 0.09) to give a mixture of diastereoisomers (80:20 dr). The diastereoisomers were then separated by flash silica column chromatography (hexane:Et_2O 90:10 to 75:25) to give:

Anti-**29** (125 mg, 51%) as a colourless solid. **mp** 153–155 °C; $[\alpha]_D^{20}$ -44.5 (*c* 1.1 in $CHCl_3$); **Chiral HPLC analysis,** Chiralcel OD-H (95:5 *n*-hexane : IPA, flow rate 1.0 mLmin^{-1}, 211 nm, 30 °C) t_R (2*S*,3*R*) 16.8 min, t_R (2*R*,3*S*) 27.9 min, 99:1 er; **IR** ν_{max} (film) 1782 (C=O, ester), 1526, 1487, 1113; **^{1}H NMR** (400 MHz, $CDCl_3$) δ_H: 2.45 (3H, s, C*H*$_3$), 3.72 (1H, d, *J* 3.9, C(4)*H*), 3.96 (1H, dd, *J* 12.2, 3.9, C(3)*H*), 4.62 (1H, d, *J* 12.2, C(2)*H*), 6.95 (1H, tt, *J* 9.8, 7.0, Ar^{1}C(4)*H*), 7.34 – 7.38 (2H, m, Ar^{2}C(3,5)*H*), 7.47 – 7.54 (4H, m, Ar^{2}C(2,6)*H* and Ar^{3}C(3,5)*H*), 7.55 – 7.60 (2H, m, Ar^{3}C(2,6)*H*); **^{19}F{^{1}H} NMR** (376 MHz, $CDCl_3$) δ_F: -152.8 – 152.7 (m), -138.5 – -138.4 (m); **^{13}C{^{1}H} NMR** (126 MHz, $CDCl_3$) δ_C: 21.3 (CH_3), 27.7 (*C*(4)H), 48.4 (*C*(3)H), 52.8 (*C*(2)H), 103.7 (t, *J* 22.7, Ar^1*C*(4)H), 110.4 (CN), 111.1 (CN), 128.0 (Ar^2*C*(2,6)H), 128.6 – 129.0 (m, Ar^{1}C(1)), 129.2 (Ar^{2}C(1)), 129.6 (Ar^3*C*(2,6)H), 129.8 (Ar^{3}C(3,5)H), 131.0 (Ar^2*C*(3,5)H), 132.2 (Ar^3*C*(1)), 136.0 (Ar^3*C*(4)), 139.0 – 141.3 (m, Ar^{1}CF), 140.5 (Ar^2*C*(4)), 144.6 – 147.0 (m, Ar^{1}CF), 166.6 (*C*(1)); **HRMS** ($MALDI\,(no\ matrix)^+$) $C_{25}H_{15}{}^{35}ClO_2N_2F_4Na$ $[M+Na]^+$ found 509.0635, requires 509.0650 (-2.9 ppm).

Syn-**S24** (28 mg, 12%) as a colourless solid. **mp** 72–74 °C; $[\alpha]_D^{20}$ -155.3 (*c* 0.9 in $CHCl_3$); **Chiral HPLC analysis,** Chiralcel OD-H (93:7 *n*-hexane : IPA, flow rate 1.5 mLmin^{-1}, 211 nm, 30 °C) t_R (2*S*,3*S*) 6.5 min, t_R (2*R*,3*R*) 11.6 min, 96:4 er; **IR** ν_{max} (film) 1769 (C=O, ester),

1526, 1487, 1132, 1111, 1096; **^{1}H NMR** (400 MHz, $CDCl_3$) δ_H: 2.29 (3H, s, CH_3), 4.00 (1H, dd, *J* 11.3, 5.1, C(3)*H*), 4.57 (1H, d, *J* 11.3, C(2)*H*), 4.64 (1H, d, *J* 5.1, C(4)*H*), 7.00 – 7.10 (5H, m, Ar^{2}C(2,3,5,6)*H* and Ar^{1}C(4)*H*), 7.20 – 7.26 (2H, m, Ar^{3}C(2,6)*H*), 7.27 – 7.33 (2H, m, Ar^{3}C(3,5)*H*); **^{19}F{^{1}H} NMR** (376 MHz, $CDCl_3$) δ_F: -152.6 – 152.4 (m), -138.2 – -138.1 (m); **^{13}C{^{1}H} NMR** (176 MHz, $CDCl_3$) δ_C: 21.1 (CH_3), 27.7 (*C*(4)H), 47.4 (*C*(3)H), 52.1 (*C*(2)H), 104.0 (t, *J* 22.7, Ar^{1}C(4)H), 110.8 (CN), 111.0 (CN), 128.2 (Ar^{2}C(2,6)H), 128.8 – 129.1 (m, Ar^1*C*(1)), 129.4 (Ar^3*C*(3,5)H), 129.5 (Ar^2*C*(1)), 130.0 (Ar^2*C*(3,5)H and Ar^3*C*(2,6)H), 131.6 (Ar^3*C*(1)), 135.4 (Ar^3*C*(4)), 139.0 (Ar^2*C*(4)), 139.4 – 141.2 (m, Ar^{1}CF), 145.0 – 146.9 (m, Ar^{1}CF), 168.8 (*C*(1)); **HRMS** ($^{MALDI\ (no\ matrix)^+}$) $C_{25}H_{15}{}^{35}ClO_2N_2F_4Na$ $[M+Na]^+$ found 509.0631, requires 509.0650 (-3.7 ppm).

(2*R*,3*S*)-2,3,5,6-tetrafluorophenyl 4,4-dicyano-2-(*p*-tolyl)-3-(3-(trifluoromethyl)phenyl)butanoate (30) and (2*R*,3*R*)-2,3,5,6-tetrafluorophenyl 4,4-dicyano-2-(*p*-tolyl)-3-(3-(trifluoromethyl)phenyl)butanoate (S25)

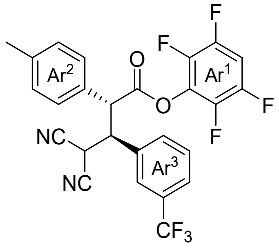

anti-**30**

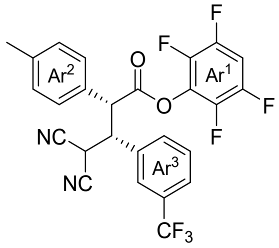

syn-**S25**

Following General Procedure E, 2,3,5,6-tetrafluorophenyl 2-(p-tolyl)acetate **9** (224 mg, 0.75 mmol, 1.5 eq), 2-(3-(trifluoromethyl)benzylidene)malononitrile **S10** (111 mg, 0.50 mmol, 1.0 eq), and (4b*R*,11a*S*)-fused-BTM **19** (6.6 mg, 0.025 mmol, 5 mol%) in CPME (1.0 M) gave crude product that was purified by flash silica column chromatography (hexane:acetone 90:10, R_F 0.10) to give a mixture of diastereoisomers (85:15 dr). The diastereoisomers were then separated by flash silica column chromatography (hexane:Et_2O 90:10 to 75:25) to give:

Anti-**30** (193 mg, 74%) as a colourless solid. **mp** 46–48 °C; $[\alpha]_D^{20}$ -53.0 (*c* 1.0 in $CHCl_3$); **Chiral HPLC analysis,** Chiralcel OD-H (97:3 *n*-hexane : IPA, flow rate 1.0 mLmin^{-1}, 211 nm, 30 °C) t_R (2*S*,3*R*) 25.8 min, t_R (2*R*,3*S*) 31.2 min, 97:3 er; **IR** ν_{max} (film) 2924 (C-H), 1780 (C=O, ester), 1526, 1487, 1329, 1169, 1128, 1076, 959; **^{1}H NMR** (400 MHz, $CDCl_3$) δ_H: 2.46 (3H, s,

CH_3), 3.76 (1H, d, *J* 4.0, C(4)*H*), 4.04 (1H, dd, *J* 12.2, 4.0, C(3)*H*), 4.68 (1H, d, *J* 12.2, C(2)*H*), 6.94 (1H, tt, *J* 9.9, 7.0, Ar^{1}C(4)*H*), 7.34 – 7.41 (2H, m, Ar^{2}C(3,5)*H*), 7.48 – 7.55 (2H, m, Ar^{2}C(2,6)*H*), 7.65 – 7.72 (1H, m, Ar^{3}C(5)*H*), 7.76 – 7.81 (1H, m, Ar^{3}C(4)*H*), 7.82 – 7.87 (1H, m, Ar^{3}C(6)*H*), 7.88 (1H, s, Ar^{3}C(2)*H*); **^{19}F{^{1}H} NMR** (376 MHz, $CDCl_3$) δ_F: -153.1 – -153.0 (m), -138.6 – -138.4 (m), -62.9 (s); **^{13}C{^{1}H} NMR** (126 MHz, $CDCl_3$) δ_C: 21.3 (CH_3), 27.5 (*C*(4)), 48.7 (*C*(3)), 52.7 (*C*(2)), 103.7 (t, *J* 22.8, Ar^1*C*(4)), 110.3 (*C*N), 110.9 (*C*N), 123.6 (q, *J* 272.6, *C*F_3), 125.3 (q, *J* 3.9, Ar^3*C*(2)H), 126.9 (q, *J* 3.6, Ar^3*C*(4)H), 128.0 (Ar^2*C*(2,6)H), 128.5 – 128.9 (m, Ar^1*C*(1)), 129.0 (Ar^2*C*(1)), 130.2 (Ar^3*C*(5)H), 131.1 (Ar^2*C*(3,5)H), 131.7 (Ar^3*C*(6)H), 132.0 (q, *J* 32.8, Ar^3*C*(3)), 134.8 (Ar^3*C*(1)), 139.0 – 141.3 (m, Ar^{1}CF), 140.6 (Ar^2*C*(4)), 144.6 – 147.0 (m, Ar^{1}CF), 166.5 (*C*(1)). **HRMS** (ESI^+) $C_{26}H_{15}O_2N_2F_7Na$ $[M+Na]^+$ found 543.0904, requires 543.0914 (-1.8 ppm).

Syn-**S25** (31 mg, 12%) as a colourless glass. $[\alpha]_D^{20}$ -112.0 (*c* 0.8 in $CHCl_3$); **Chiral HPLC analysis,** Chiralcel OD-H (97:3 *n*-hexane : IPA, flow rate 1.5 mLmin^{-1}, 211 nm, 30 °C) t_R (2*S*,3*S*) 12.3 min, t_R (2*R*,3*R*) 21.0 min, 89:11 er; **IR** ν_{max} (film) 2926 (C-H), 1771 (C=O, ester), 1528, 1487, 1329, 1169, 1130, 1076, 959; **^{1}H NMR** (500 MHz, $CDCl_3$) δ_H: 2.27 (3H, s, CH_3), 4.10 (1H, dd, *J* 11.1, 5.3, C(3)*H*), 4.62 (1H, d, *J* 11.1, C(2)*H*), 4.68 (1H, d, *J* 5.3, C(4)*H*), 6.99 – 7.11 (5H, m, Ar^{1}C(4)*H* and Ar^{2}C(2,3,5,6)*H*), 7.44 – 7.50 (2H, m, Ar^{3}C(2,5)*H*), 7.51 – 7.55 (1H, m, Ar^{3}C(6)*H*), 7.56 – 7.61 (1H, m, Ar^{3}C(4)*H*); **^{19}F{^{1}H} NMR** (376 MHz, $CDCl_3$) δ_F: -152.5 – -152.4 (m), -138.2 – -138.0 (m), -63.0 (s); **^{13}C{^{1}H} NMR** (126 MHz, $CDCl_3$) δ_C: 21.1 (*C*H_3), 27.5 (*C*(4)), 47.8 (*C*(3)), 52.1 (*C*(2)), 104.0 (t, *J* 22.7, Ar^{1}C(4)), 110.7 (*C*N), 110.9 (*C*N), 123.5 (q, *J* 272.5, *C*F_3), 125.8 (q, *J* 4.1, Ar^3*C*(2)H), 126.2 (q, *J* 3.7, Ar^3*C*(4)H), 128.2 (Ar^2*C*(2,6)H), 128.6 – 129.2 (m, Ar^1*C*(1)), 129.3 (Ar^2*C*(1)), 129.7 (Ar^{3}C(5)H), 130.0 (Ar^2*C*(3,5)H), 131.4 (q, *J* 32.8, Ar^{3}C(3)), 132.0 (Ar^{3}C(6)H), 134.2 (Ar^{3}C(1)), 139.1 (Ar^2*C*(4)), 139.1 – 141.4 (m, Ar^{1}CF), 144.7 – 147.2 (m, Ar^1*C*F), 168.6 (*C*(1)). **HRMS** (ESI^+) $C_{26}H_{15}O_2N_2F_7Na$ $[M+Na]^+$ found 543.0905, requires 543.0914 (-1.7 ppm).

(2*R*,3*S*)-2,3,5,6-tetrafluorophenyl 4,4-dicyano-2-(*p*-tolyl)-3-(2-(trifluoromethyl)phenyl)butanoate (31) and (2*R*,3*R*)-2,3,5,6-tetrafluorophenyl 4,4-dicyano-2-(*p*-tolyl)-3-(2-(trifluoromethyl)phenyl)butanoate (S26)

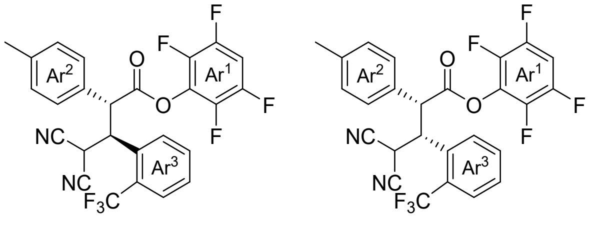

anti-**31** *syn*-**S26**

Following General Procedure E, 2,3,5,6-tetrafluorophenyl 2-(p-tolyl)acetate **9** (224 mg, 0.75 mmol, 1.5 eq), 2-(2-(trifluoromethyl)benzylidene)malononitrile **S11** (111 mg, 0.50 mmol, 1.0 eq), and (4b*R*,11a*S*)-fused-BTM **19** (6.6 mg, 0.025 mmol, 5 mol%) in CPME (1.0 M) gave crude product that was purified by flash silica column chromatography (hexane:acetone 90:10, R_F 0.10) to give the combined *anti* and *syn* diastereoisomers (47:53 dr) (150 mg, 58%) as an inseparable mixture as a colourless solid. **mp** 44–46 °C; $[\alpha]_D^{20}$ -120.0 (*c* 1.0 in $CHCl_3$); **IR** ν_{max} (film) 2318, 1773 (C=O, ester), 1528, 1489, 1312, 1179, 1165, 1113, 1038, 959; **HRMS** (ESI^+) $C_{26}H_{15}O_2N_2F_7Na$ $[M+Na]^+$ found 543.0906, requires 543.0914 (-1.5 ppm).

Data for *anti*-**31**: **Chiral HPLC analysis,** Chiralpak AD-H (99:1 *n*-hexane : IPA, flow rate 1.0 mLmin^{-1}, 211 nm, 30 °C) t_R (2*S*,3*R*) 16.2 min, t_R (2*R*,3*S*) 18.3 min, 99:1 er; **^{1}H NMR** (500 MHz, $CDCl_3$) δ_H: 2.46 (3H, s, C*H*$_3$), 3.90 (1H, d, *J* 3.8, C(4)*H*), 4.51 – 4.59 (1H, m, C(3)*H*), 4.84 (1H, d, *J* 11.8, C(2)*H*), 6.92 (1H, tt, *J* 9.8, 7.0, Ar^{1}C(4)*H*), 7.35 – 7.41 (2H, m, Ar^{2}C(3,5)*H*), 7.58 – 7.65 (3H, m, Ar^{2}C(2,6)*H* and Ar^{3}C(4)*H*), 7.76 – 7.81 (1H, m, Ar^{3}C(5)*H*), 7.84 – 7.89 (1H, m, Ar^{3}C(3)*H*), 8.00 – 8.03 (1H, m, Ar^{3}C(6)*H*); **^{19}F{^{1}H} NMR** (470 MHz, $CDCl_3$) δ_F: -153.2 – -153.1 (m), -138.8 – -138.6 (m), -57.2 (s); **^{13}C{^{1}H} NMR** (126 MHz, $CDCl_3$) δ_C: 21.3 (CH_3), 27.6 (*C*(4)), 43.2 (*C*(3)), 53.5 (*C*(2)), 103.5 (t, *J* 22.8, Ar^1*C*(4)), 110.4 (CN), 110.5 (CN), 124.0 (q, *J* 274.4, *C*F$_3$), 127.3 (Ar^3*C*(6)H), 127.8 (q, *J* 5.8, Ar^3*C*(3)H), 128.5 (Ar^2*C*(2,6)H), 128.5 – 128.8 (m, Ar^1*C*(1)), 128.9 (Ar^2*C*(1)), 129.5 – 130.2 (m, Ar^3*C*(2) and Ar^{3}C(4)H), 130.9 (Ar^{2}C(3,5)H), 133.2 (Ar^{3}C(5)H), 133.8 (Ar^{3}C(1)), 139.0 – 141.4 (m, Ar^{1}CF), 140.7 (Ar^2*C*(4)), 144.6 – 147.1 (m, Ar^{1}CF), 166.1 (*C*(1)).

Data for *syn*-**S26**: **Chiral HPLC analysis,** Enantiomeric ratio could not be determined; **^{1}H NMR** (500 MHz, $CDCl_3$) δ_H: 2.26 (3H, s, CH_3), 4.52 – 4.59 (1H, m, C(3)*H*), 4.76 (1H, d, *J* 11.1, C(2)*H*), 4.81 (1H, d, *J* 5.8, C(4)*H*), 7.00 – 7.10 (5H, m, Ar^{1}C(4)*H* and Ar^{2}C(2,3,5,6)*H*), 7.44 – 7.50 (1H, m, Ar^{3}C(4)*H*), 7.58 – 7.65 (1H, m, Ar^{3}C(3)*H*), 7.67 – 7.74 (1H, m, Ar^{3}C(5)*H*), 7.96 – 8.00 (1H, m, Ar^{3}C(6)*H*); **^{19}F{^{1}H} NMR** (470 MHz, $CDCl_3$) δ_F: -152.4 – -152.3 (m), -138.4 – -138.2 (m), -56.6 (s); **^{13}C{^{1}H} NMR** (126 MHz, $CDCl_3$) δ_C: 21.1 (CH_3), 27.7 (*C*(4)), 41.6 (*C*(3)), 53.1 (*C*(2)), 104.0 (t, *J* 22.7, Ar^{1}C(4)), 110.4 (*C*N), 111.3 (*C*N), 123.5 (q, *J* 274.5, *C*F_3), 127.2 (q, *J* 6.0, Ar^{3}C(3)H), 128.0 (Ar^{3}C(6)H), 128.6 (Ar^{2}C(2,6)H), 128.8 – 129.2 (m, Ar^{1}C(1) and Ar^{2}C(1)), 129.3 – 129.7 (m, Ar^{3}C(2), Ar^{3}C(4)H and Ar^{2}C(3,5)H), 132.7 (Ar^{3}C(5)H), 133.1 (Ar^3*C*(1)), 138.9 (Ar^2*C*(4)), 139.0 – 141.4 (m, Ar^{1}CF), 144.6 – 147.1 (m, Ar^{1}CF), 168.8 (*C*(1)).

(2*R*,3*S*)-2,3,5,6-tetrafluorophenyl 4,4-dicyano-3-(4-methoxyphenyl)-2-(*p*-tolyl)butanoate (32) and (2*R*,3*R*)-2,3,5,6-tetrafluorophenyl 4,4-dicyano-3-(4-methoxyphenyl)-2-(*p*-tolyl)butanoate (S27)

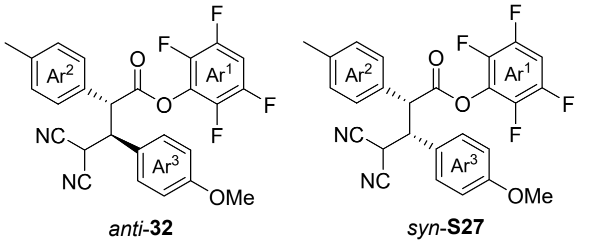

anti-**32** *syn*-**S27**

Following General Procedure E, 2,3,5,6-tetrafluorophenyl 2-(p-tolyl)acetate **9** (224 mg, 0.75 mmol, 1.5 eq), 2-(4-methoxybenzylidene)malononitrile **S6** (92 mg, 0.50 mmol, 1.0 eq), and (4b*R*,11a*S*)-fused-BTM **19** (6.6 mg, 0.025 mmol, 5 mol%) in CH_2Cl_2 (1.0 M) gave crude product (80:20 dr) that was purified by flash silica column chromatography (hexane:Et_2O 90:10 to 80:20, R_F 0.13 at 80:20) to yield the combined *anti* and *syn* diastereoisomers (80:20 dr) (80 mg, 33%) as an inseparable mixture as a colourless solid. **mp** 88–90 °C; $[\alpha]_D^{20}$ -53.6 (*c* 1.1 in $CHCl_3$); **IR** ν_{max} (film) 1780 (C=O, ester), 1612, 1527, 1516, 1487, 1258, 1180, 1109, 957; **HRMS** (EI^+) $C_{26}H_{18}O_3N_2F_4$ $[M]^+$ found 482.1250, requires 482.1248 (+0.4 ppm).

Data for major diastereoisomer *anti*-**32**: **Chiral HPLC analysis,** Chiralcel OD-H (98:2 *n*-hexane : IPA, flow rate 1.5 mLmin^{-1}, 211 nm, 30 °C) t_R (2*S*,3*R*) 15.6 min, t_R (2*R*,3*S*) 19.0 min, 99:1 er; **^{1}H NMR** (400 MHz, $CDCl_3$) δ_H: 2.44 (3H, s, CH_3), 3.70 (1H, d, *J* 3.9, C(4)*H*), 3.87

(3H, s, OCH_3), 3.94 (1H, dd, *J* 12.3, 3.9, C(3)*H*), 4.63 (1H, d, *J* 12.3, C(2)*H*), 6.93 (1H, tt, *J* 9.9, 7.0, $Ar^1C(4)H$), 7.01 – 7.06 (2H, m, $Ar^3C(3,5)H$), 7.32 – 7.38 (2H, m, $Ar^2C(3,5)H$), 7.48 – 7.54 (2H, m, $Ar^2C(2,6)H$), 7.54 – 7.58 (2H, m, $Ar^3C(2,6)H$); **^{19}F{^{1}H} NMR** (470 MHz, $CDCl_3$) δ_F: -152.6 – 152.5 (m), -138.8 – -138.7 (m); **^{13}C{^{1}H} NMR** (126 MHz, $CDCl_3$) δ_C: 21.3 (*C*H_3), 28.1 (*C*(4)H), 48.3 (*C*(3)H), 53.0 (*C*(2)H), 55.4 (OCH_3), 103.5 (t, *J* 22.8, Ar^1*C*(4)H), 110.8 (*C*N), 111.5 (*C*N), 114.9 (Ar^3C(3,5)H), 125.5 (Ar^3*C*(1)), 128.0 (Ar^2*C*(2,6)H), 128.7 – 129.1 (m, Ar^1*C*(1)), 129.5 (Ar^3*C*(2,6)H), 129.8 (Ar^2*C*(1)), 130.9 (Ar^2*C*(3,5)H), 139.1 – 141.3 (m, Ar^1CF), 140.2 (Ar^2*C*(4)), 144.6 – 147.0 (m, Ar^1CF), 160.6 (Ar^3*C*(4)), 166.7 (*C*(1));

Data for minor diastereoisomer *syn*-**S27**: **Chiral HPLC analysis,** Chiralcel OD-H (98:2 *n*-hexane : IPA, flow rate 1.5 mLmin^{-1}, 211 nm, 30 °C) t_R (2*S*,3*S*) 12.2 min, t_R (2*R*,3*R*) 25.4 min, 96:4 er; **^{1}H NMR** (400 MHz, $CDCl_3$) (*selected*) δ_H: 2.28 (3H, s, CH_3), 3.78 (3H, s, OCH_3), 6.80 – 6.86 (2H, m, $Ar^3C(3,5)H$), 7.18 – 7.24 (2H, m, $Ar^3C(2,6)H$); **^{19}F{^{1}H} NMR** (470 MHz, $CDCl_3$) δ_F: -152.5 – 152.4 (m), -138.3 – -138.2 (m); **^{13}C{^{1}H} NMR** (126 MHz, $CDCl_3$) (*selected*) δ_C: 21.1 (*C*H_3), 28.2 (*C*(4)H), 47.4 (*C*(3)H), 52.3 (*C*(2)H), 55.2 (OCH_3), 111.1 (*C*N), 111.3 (*C*N), 114.4 (Ar^3C(3,5)H), 124.9 (Ar^3*C*(1)), 128.3 (Ar^2C(2,6)H), 129.6 (Ar^3*C*(2,6)H), 130.0 (Ar^2*C*(1)), 138.6 (Ar^2*C*(4)), 160.0 (Ar^3*C*(4)), 169.0 (*C*(1));

10 Isolation of CIDT products by filtration

A CIDT process was in operation for products **14**, **21**, and **28**. This allowed isolation of the respective products by simple filtration washing with cold Et_2O (4 mL). In each case the white solid precipitate was a single diastereoisomer of the product. The filtrate from each reaction contained a mixture of diastereoisomers which were subsequently purified by flash silica column chromatography.

14: Filtration afforded 65% yield, >95:5 dr, 99:1 er. The filtrate was purified to give a mixture of diastereoisomers (63:37 dr) both with 95:5 er in 12% combined yield.

21: Filtration afforded 19% yield, >95:5 dr, 99:1 er. The filtrate (76:24 dr) was purified to give **21** in 46% yield and 99:1 er.

28: Filtration afforded 57% yield, >95:5 dr, 99:1 er. The filtrate (80:20 dr) was purified to give **28** in 17% yield and 94:6 er.

11 Gram scale reaction

(2*R*,3*S*)-2,3,5,6-tetrafluorophenyl 4,4-dicyano-3-phenyl-2-(*p*-tolyl)butanoate (14)

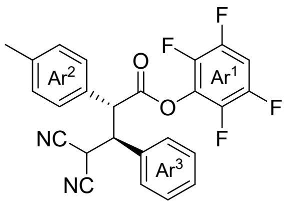

Following General Procedure E, 2,3,5,6-tetrafluorophenyl 2-(p-tolyl)acetate **9** (1.34 g, 4.5 mmol, 1.5 eq), 2-benzylidene malononitrile **2** (0.46 g, 3.0 mmol, 1.0 eq), and (4b*R*,11a*S*)-fused-BTM **19** (40 mg, 0.15 mmol, 5 mol%) in CPME (1.0 M) gave crude product that was purified directly by recrystallisation from hexane/CH_2Cl_2 to give the title compound as a colourless solid (1.10 g, 81%).

Chiral HPLC analysis, Chiralcel OD-H (99:1 *n*-hexane : IPA, flow rate 1.5 mLmin^{-1}, 211 nm, 30 °C) t_R (2*S*,3*R*) 24.1 min, t_R (2*R*,3*S*) 29.2 min, >99:1 er.

12 Product derivatisations

(2*R*,3*S*)-*N*-allyl-4,4-dicyano-3-phenyl-2-(*p*-tolyl)butanamide (37)

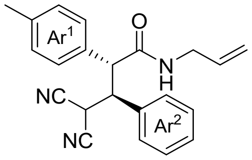

To a solution of TeFP ester **14** (181 mg, 0.4 mmol, 1.0 equiv.) in anhydrous EtOAc (0.2 M) was added allyl amine (60 μL, 0.8 mmol 2.0 equiv.) and the reaction mixture stirred at room temperature for 24 h. The mixture was then diluted with EtOAc (10 mL) and washed with 1 M NaOH (3 × 10 mL) then brine (10 mL). The organic layer was dried over $MgSO_4$, filtered, and concentrated under reduced pressure. The crude product was purified by flash silica column chromatography (hexane:EtOAc 80:20 to 70:30, R_F 0.31 at 70:30) to give the title compound (83 mg, 61%) as a colourless solid. **mp** 148–150 °C; $[\alpha]_D^{20}$ -62.1 (*c* 1.0 in $CHCl_3$); **Chiral HPLC analysis,** Chiralpak IB (90:10 *n*-hexane : IPA, flow rate 1.0 mLmin^{-1}, 211 nm, 30 °C) t_R (2*S*,3*R*) 8.8 min, t_R (2*R*,3*S*) 14.0 min, >99:1 er; **IR** ν_{max} (film) 3298 (N-H), 2891 (C-H), 1653 (C=O, amide), 1639, 1558, 1541, 1508, 1456, 1261, 991, 914, 760; **^{1}H NMR** (400 MHz, $CDCl_3$) δ_H: 2.41 (3H, s, C*H*$_3$), 3.41 – 3.53 (1H, m, NCH^AH^B), 3.62 – 3.73 (1H, m, NCH^AH^B), 3.76 (1H, d, *J* 3.6, C(4)*H*), 4.00 – 4.12 (2H, m, C(3)*H* and C(2)*H*), 4.58 – 4.67 (1H, m, CH=CH^AH^B), 4.81 – 4.89 (1H, m, CH=CH^AH^B), 5.36 – 5.50 (1H, m, C*H*=CH_2), 5.87 (1H, t, *J* 5.9, N*H*), 7.25 – 7.30 (2H, m, Ar^{1}C(3,5)*H*), 7.43 – 7.52 (5H, m, Ar^{1}C(2,6)*H* and Ar^{2}C(3,4,5)*H*), 7.57 – 7.63 (2H, m, Ar^{2}C(2,6)*H*); **^{13}C{^{1}H} NMR** (126 MHz, $CDCl_3$) δ_C: 21.2 (CH_3), 27.9 (*C*(4)H), 41.7 (NCH_2), 49.0 (*C*(3)H), 54.8 (*C*(2)H), 111.6 (*C*N), 111.9 (*C*N), 115.8 (CH=CH_2), 128.0 (Ar^1*C*(2,6)H), 128.5 (Ar^2*C*(2,6)H), 129.2 (Ar^{2}C(3,5)H), 129.3 (Ar^2*C*(4)H), 130.5 (Ar^1*C*(3,5)H), 132.3 (Ar^1*C*(1)), 133.3 (*C*H=CH_2), 135.1 (Ar^2*C*(1)), 139.2 (Ar^1*C*(4)), 169.5 (*C*(1)); **HRMS** (ESI^+) $C_{22}H_{21}ON_3Na$ $[M+Na]^+$ found 366.1576, requires 366.1577 (-0.3 ppm).

(2*R*,3*S*)-methyl 4,4-dicyano-3-phenyl-2-(*p*-tolyl)butanoate (38)

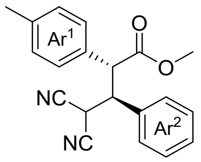

To a solution of TeFP ester **14** (181 mg, 0.4 mmol, 1.0 equiv.) in anhydrous EtOAc (0.25 M) was added anhydrous MeOH (0.41 mL, 10 mmol, 25.0 equiv.) and DMAP (9.8 mg, 0.08 mmol, 0.2 equiv.) and the reaction mixture stirred at RT for 24 h. The mixture was then diluted with EtOAc (10 mL) and washed with sat. Na_2CO_3 (2 × 10 mL) then brine (10 mL). The organic layer was dried over $MgSO_4$, filtered, and concentrated under reduced pressure. The crude product was purified by flash silica column chromatography (CH_2Cl_2:hexane 50:50 to 100:0, R_F 0.18 at 50:50) to give the title compound (85 mg, 67%) as a colourless solid. **mp** 130–132 °C; $[\alpha]_D^{20}$ -84.9 (*c* 1.0 in $CHCl_3$); **Chiral HPLC analysis,** Chiralcel OD-H (99:1 *n*-hexane : IPA, flow rate 1.0 mLmin^{-1}, 211 nm, 30 °C) t_R (2*R*,3*S*) 19.4 min, t_R (2*S*,3*R*) 23.7 min, >99:1 er; **IR** ν_{max} (film) 2953 (C-H), 1736 (C=O, ester), 1514, 1456, 1435, 1341, 1306, 1206, 1165, 760; **^{1}H NMR** (500 MHz, $CDCl_3$) δ_H: 2.41 (3H, s, C*H*$_3$), 3.47 (3H, s, OC*H*$_3$), 3.66 (1H, d, *J* 4.0, C(4)*H*), 3.91 (1H, dd, *J* 12.2, 4.0, C(3)*H*), 4.31 (1H, d, *J* 12.2, C(2)*H*), 7.26 – 7.33 (2H, m, Ar^{1}C(3,5)*H*), 7.41 – 7.51 (5H, m, Ar^{1}C(2,6)*H* and Ar^{2}C(3,4,5)*H*), 7.53 – 7.60 (2H, m, Ar^{2}C(2,6)*H*); **^{13}C{^{1}H} NMR** (126 MHz, $CDCl_3$) δ_C: 21.2 (CH_3), 27.8 (*C*(4)H), 49.1 (*C*(3)H), 52.5 (OCH_3), 53.2 (*C*(2)H), 110.9 (*C*N), 111.6 (*C*N), 128.0 (Ar^1*C*(2,6)H), 128.4 (Ar^2*C*(2,6)H), 129.3 (Ar^{2}C(3,5)H), 129.4 (Ar^2*C*(4)H), 130.6 (Ar^{1}C(3,5)H), 131.1 (Ar^1*C*(1)), 134.5 (Ar^2*C*(1)), 139.5 (Ar^1*C*(4)), 171.0 (*C*(1)); **HRMS** (ESI^+) $C_{20}H_{18}O_2N_2Na$ $[M+Na]^+$ found 341.1273, requires 341.1260 (+3.8 ppm).

Methyl (2*R*,3*S*,4*R*)-5-amino-4-cyano-5-oxo-3-phenyl-2-(*p*-tolyl)pentanoate (39) and Methyl (2*R*,3*S*,4*S*)-5-amino-4-cyano-5-oxo-3-phenyl-2-(*p*-tolyl)pentanoate (S28)

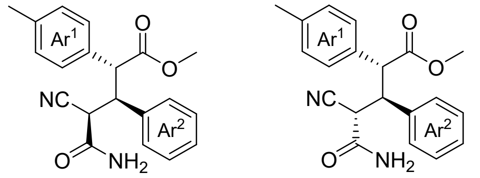

(2*R*,3*S*,4*R*)-**39** (2*R*,3*S*,4*S*)-**S28**

Adapting the procedure of Maffioli and co-workers,[12] methyl ester **38** (80 mg, 0.25 mmol, 1.0 equiv.), $PdCl_2$ (4.4 mg, 25 μmol, 10 mol%), and acetamide (62 mg, 1.05 mmol, 4.2 equiv.) were dissolved in THF/H_2O (1.5 mL, 3:1 v/v) and stirred at room temperature for 2 h until complete by TLC. The mixture was then diluted with CH_2Cl_2 and H_2O, and the organic layer separated. The aqueous phase was then extracted with CH_2Cl_2 (× 3) and the combined organics dried over Na_2SO_4, filtered, and concentrated under reduced pressure. The crude product was purified by flash silica column chromatography (CH_2Cl_2:Et_2O 90:10, R_F 0.29) to give the title compound (76 mg, 90%) as an inseparable mixture of diastereoisomers (86:14 dr) as a colourless solid. **mp** 71–73 °C; $[\alpha]_D^{20}$ -125 (*c* 1.0 in $CHCl_3$); **IR** ν_{max} (film) 3335 (N-H), 3192 (N-H), 2953 (C-H), 1736 (C=O, ester), 1682 (C=O, amide), 1605, 1512, 1342, 1161, 910, 824; **HRMS** (ESI^+) $C_{20}H_{21}O_3N_2$ $[M+H]^+$ found 337.1544, requires 337.1547 (-0.9 ppm).

Data for **39**: **Chiral HPLC analysis,** Chiralpak ID (93:7 *n*-hexane : IPA, flow rate 1.0 mLmin^{-1}, 211 nm, 40 °C) t_R (2*R*,3*S*,4*R*) 23.1 min, t_R (2*S*,3*R*,4*S*) 24.3 min, >99:1 er; **^{1}H NMR** (400 MHz, $CDCl_3$) δ_H: 2.39 (3H, s, C*H*$_3$), 3.28 (1H, d, *J* 4.0, C(4)*H*), 3.41 (3H, s, OC*H*$_3$), 4.18 (1H, dd, *J* 12.5, 4.0, C(3)*H*), 4.28 (1H, d, *J* 12.5, C(2)*H*), 5.75 (1H, br s, NH^AH^B), 5.84 (1H, br s, NH^AH^B), 7.24 – 7.28 (2H, m, Ar^{1}C(3,5)*H*), 7.31 – 7.42 (3H, m, Ar^{2}C(3,4,5)*H*), 7.43 – 7.53 (4H, m, Ar^{1}C(2,6)*H* and Ar^{2}C(2,6)*H*); **^{13}C{^{1}H} NMR** (126 MHz, $CDCl_3$) δ_C: 21.2 (*C*H$_3$), 42.3 (*C*(4)H), 47.8 (*C*(3)H), 52.2 (O*C*H$_3$), 54.1 (*C*(2)H), 116.8 (*C*N), 128.2 (Ar^1*C*(2,6)H), 128.4 (Ar^2*C*(4)H), 128.5 (Ar^2*C*(2,6)H), 128.8 (Ar^2*C*(3,5)H), 130.3 (Ar^1*C*(3,5)H), 132.1 (Ar^1*C*(1)), 136.2 (Ar^2*C*(1)), 138.7 (Ar^1*C*(4)), 165.6 (*C*(5)), 171.7 (*C*(1)).

Data for **S28**: **Chiral HPLC analysis,** Chiralpak ID (93:7 *n*-hexane : IPA, flow rate 1.0 mLmin^{-1}, 211 nm, 40 °C) t_R (2*S*,3*R*,4*R*) 21.6 min, t_R (2*R*,3*S*,4*S*) 33.7 min, >99:1 er; **^{1}H NMR** (400 MHz, $CDCl_3$) (*selected*) δ_H: 2.36 (3H, s, C*H*$_3$), 3.40 (3H, s, OC*H*$_3$), 3.46 (1H, d, *J* 2.4, C(4)*H*), 4.11 (1H, dd, *J* 12.3, 2.4, C(3)*H*), 4.66 (1H, d, *J* 12.3, C(2)*H*), 5.56 (1H, br s, NH^AH^B), 5.72 (1H, br s, NH^AH^B), 7.16 – 7.22 (2H, m, Ar^{1}C(3,5)*H*); **^{13}C{^{1}H} NMR** (126 MHz, $CDCl_3$) δ_C: 21.2 (*C*H$_3$), 41.4 (*C*(4)H), 49.1 (*C*(3)H), 52.1 (OCH$_3$), 52.8 (*C*(2)H), 117.8 (*C*N), 128.1 (Ar^1*C*(2,6)H), 128.3 (Ar^2*C*(4)H), 128.9 (Ar^2*C*(2,6)H), 129.4 (Ar^2*C*(3,5)H), 129.7 (Ar^1*C*(3,5)H), 132.2 (Ar^1*C*(1)), 137.7 (Ar^2*C*(1)), 138.5 (Ar^1*C*(4)), 165.4 (*C*(5)), 172.3 (*C*(1)).

13 Single crystal X-ray diffraction data

X-ray diffraction data for all compounds were collected using a Rigaku MM-007HF High Brilliance RA generator/confocal optics [Cu Kα radiation (λ = 1.54187 Å)]. Crystals of compounds **5** and **39** were run at 125 K on an XtaLAB P200 diffractometer, while those of compound **14** were run at 173 K on an XtaLAB P100 diffractometer. Intensity data were collected using either ω steps or both ω and φ steps, accumulating area detector images spanning at least a hemisphere of reciprocal space. Data for all compounds analysed were collected using CrystalClear[13] and processed (including correction for Lorentz, polarisation and absorption) using CrysAlisPro.[14] Structures were solved by either dual-space (SHELXT[15]) or direct (SIR2011[16]) methods and refined by full-matrix least-squares against F^2 (SHELXL-2018/3[17]). Non-hydrogen atoms were refined anisotropically, and carbon-bound hydrogen atoms were refined using a riding model. Hydrogens bound to nitrogen in compound **39** were located from the difference Fourier map and refined isotropically subject to a distance restraint. All calculations were performed using either the CrystalStructure[18] or the Olex2[19] interface. Selected crystallographic data are presented in Tables S7-9.

CCDC 2253984-2253986 contains the supplementary crystallographic data for this paper. These data can be obtained free of charge from The Cambridge Crystallographic Data Centre via www.ccdc.cam.ac.uk/structures.

Table S7: X-ray data for 5

	**(2*R*,3*R*)-5**
CCDC	2253984
empirical formula	$C_{25}H_{19}N_3O_4$
fw	425.44
crystal description	colourless prism
crystal size [mm]	0.20×0.10×0.05
space group	$P2_12_12_1$ (#19)
a [Å]	11.65320(10)
b [Å]	13.39570(11)
c [Å]	14.02840(12)
vol [Å]^3	2189.87(3)
Z	4
p (calc) [g/cm^3]	1.290
μ [mm^{-1}]	0.731
F(000)	888
reflections collected	24536
independent reflections ($\boldsymbol{R}_{int}$)	4447(0.0190)
parameters/restraints	291/0
GOF on F^2	1.029
R_1 [I > 2σ(I)]	0.0300
wR_2 (all data)	0.0815
largest diff. peak/hole [e/Å^3]	0.23, -0.26
Flack parameter	0.05(5)

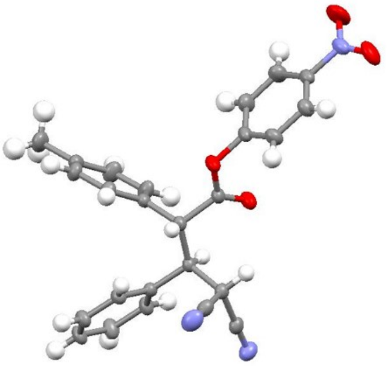

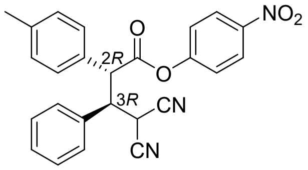

syn-**5**

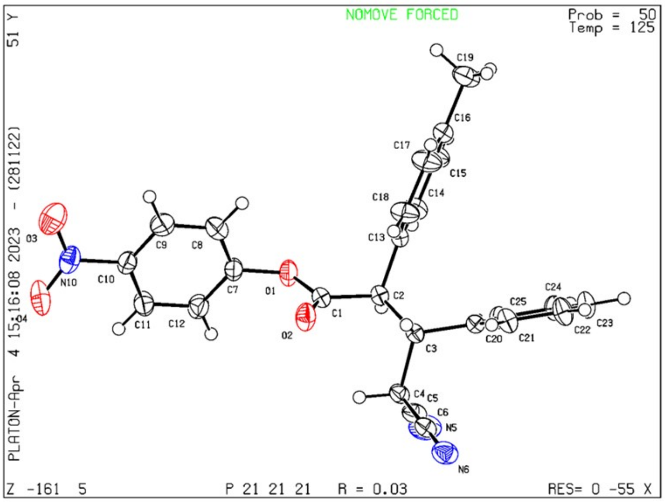

Table S8: X-ray data for 14

	**(2*R*,3S)-14**
CCDC	2253985
empirical formula	$C_{25}H_{16}F_4N_2O_2$
fw	452.41
crystal description	colourless prism
crystal size [mm]	0.18×0.10×0.03
space group	$P2_12_12_1$ (#19)
a [Å]	5.87366(8)
b [Å]	9.56529(13)
c [Å]	36.9186(4)
vol [Å]^3	2074.21(5)
Z	4
p (calc) [g/cm^3]	1.449
μ [mm^{-1}]	1.005
F(000)	928
reflections collected	20708
independent reflections ($\boldsymbol{R}_{int}$)	3758(0.0287)
parameters/restraints	300/0
GOF on F^2	1.230
R_1 [I > 2σ(l)]	0.0513
wR_2 (all data)	0.1079
largest diff. peak/hole [e/Å^3]	0.45, -0.54
Flack parameter	0.059(3)

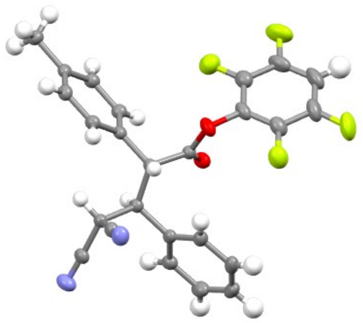

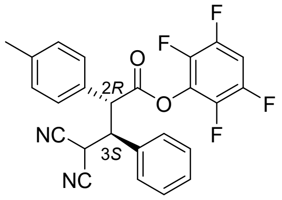

anti-**14**

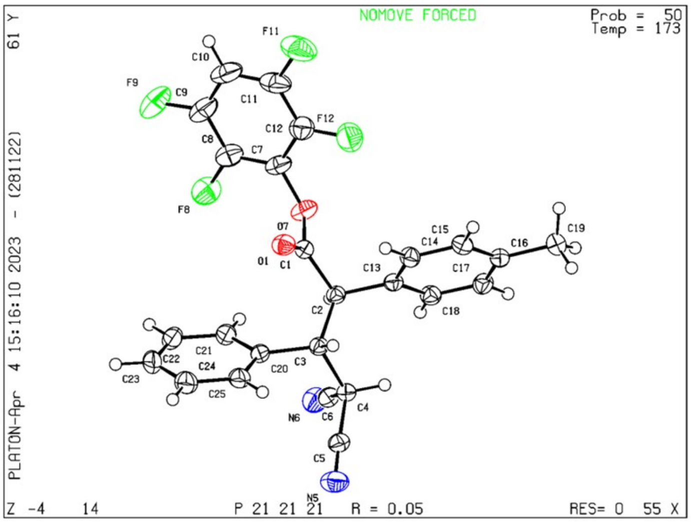

Table S9: X-ray data for 39

	(2*R*,3*S*,4*R*)-39
CCDC	2253986
empirical formula	$C_{20}H_{20}N_2O_3$
fw	336.38
crystal description	colourless prism
crystal size [mm]	0.21×0.06×0.02
space group	$P2_12_12_1$ (#19)
a [Å]	7.83550(10)
b [Å]	9.02620(10)
c [Å]	26.1596(4)
vol [Å]^3	1850.13(4)
Z	4
p (calc) [g/cm^3]	1.208
μ [mm^{-1}]	0.664
F(000)	712
reflections collected	21639
independent reflections (R_{int})	3772(0.0281)
parameters/restraints	236/2
GOF on F^2	1.049
R_1 [I > 2σ(I)]	0.0327
wR_2 (all data)	0.0906
largest diff. peak/hole [e/Å^3]	0.17, -0.16
Flack parameter	-0.03(8)

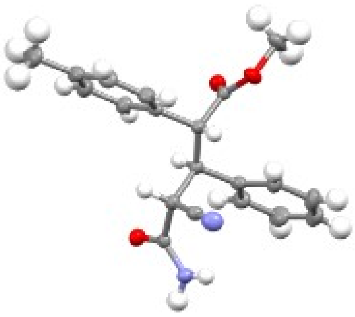

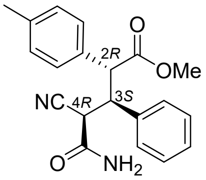

(2*R*,3*S*,4*R*)-**39**

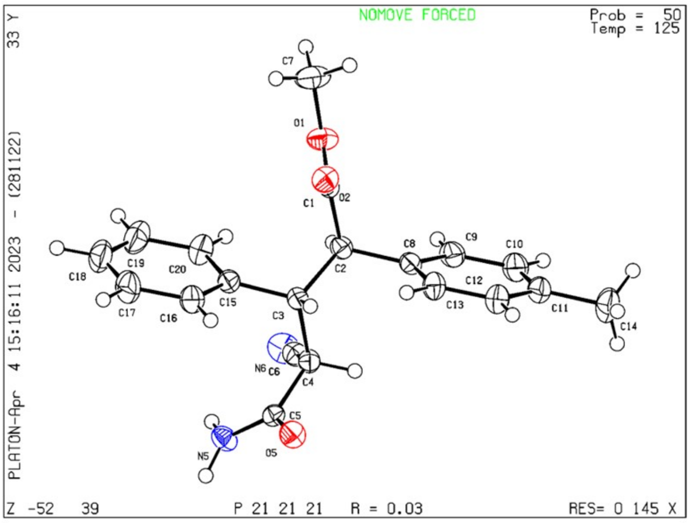

14 References

1. B. Yan, L. Zuo, X. Chang, T. Liu, M. Cui, Y. Liu, H. Sun, W. Chen and W. Guo, *Org. Lett.*, 2021, **23**, 351-357.
2. J. N. Arokianathar, A. B. Frost, A. M. Z. Slawin, D. Stead and A. D. Smith, *ACS Catal.*, 2018, **8**, 1153-1160.
3. C. McLaughlin, A. M. Z. Slawin and A. D. Smith, *Angew. Chem. Int. Ed.* , 2019, **58**, 15111-15119.
4. R. J. Mayer, M. Breugst, N. Hampel, A. R. Ofial and H. Mayr, *J. Org. Chem.*, 2019, **84**, 8837-8858.
5. M. Trilla, R. Pleixats, M. W. C. Man and C. Bied, *Green Chem.*, 2009, **11**, 1815-1820.
6. A. Karam, N. Villandier, M. Delample, C. K. Koerkamp, J.-P. Douliez, R. Granet, P. Krausz, J. Barrault and F. Jérôme, *Chem. Eur. J.*, 2008, **14**, 10196-10200.
7. Q. Wan, S. Li, Q. Kang, Y. Yuan and Y. Du, *J. Org. Chem.*, 2019, **84**, 15201-15211.
8. G. R. Krishnan and K. Sreekumar, *Eur. J. Org. Chem.*, 2008, **2008**, 4763-4768.
9. M. Zhang, A. Q. Zhang, H. H. Chen, J. Chen and H. Y. Chen, *Synth. Commun.*, 2006, **36**, 3441-3445.
10. S. Balalaie and N. Nemati, *Synth. Commun.*, 2000, **30**, 869-875.
11. A. R. Longstreet, B. S. Campbell, B. F. Gupton and D. T. McQuade, *Org. Lett.*, 2013, **15**, 5298-5301.
12. S. I. Maffioli, E. Marzorati and A. Marazzi, *Org. Lett.*, 2005, **7**, 5237-5239.
13. *CrystalClear-SM Expert* v2.1. Rigaku Americas, *The Woodlands, Texas, USA*, and Rigaku Corporation, *Tokyo, Japan,* 2015.
14. *CrysAlisPro* v1.171.38.46, v1.171.40.14a, v1.171.41.93a. Rigaku Oxford Diffraction, Rigaku Corporation, *Oxford, U.K.*, 2015-2020.
15. G. M. Sheldrick, *Acta Crystallogr., Sect. A: Found. Adv.* 2015, **71**, 3-8.
16. M. C. Burla, R. Caliandro, M. Camalli, B. Carrozzini, G. L. Cascarano, C. Giacovazzo, M. Mallamo, A. Mazzone, G. Polidori, and R. Spagna, *J. Appl. Crystallogr.* 2012, **45**, 357-361.
17. G. M. Sheldrick, *Acta Crystallogr., Sect. C: Struct. Chem.* 2015, **71**, 3-8.
18. *CrystalStructure* v4.3.0. Rigaku Americas, *The Woodlands, Texas, USA,* and Rigaku Corporation, *Tokyo, Japan,* 2018.
19. O. V. Dolomanov, L. J. Bourhis, R. J. Gildea, J. A. K. Howard, and H. Puschmann, *J. Appl. Crystallogr.* 2009, **42**, 339-341.

Appendix I: ^{1}H, $^{19}F\{^{1}H\}$, $^{13}C\{^{1}H\}$, 2D ^{1}H COSY, 2D ^{1}H–^{13}C HSQC and 2D ^{1}H–^{13}C HMBC NMR Spectra

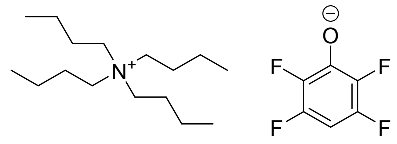

S1

^1H, MeOD, 400 MHz

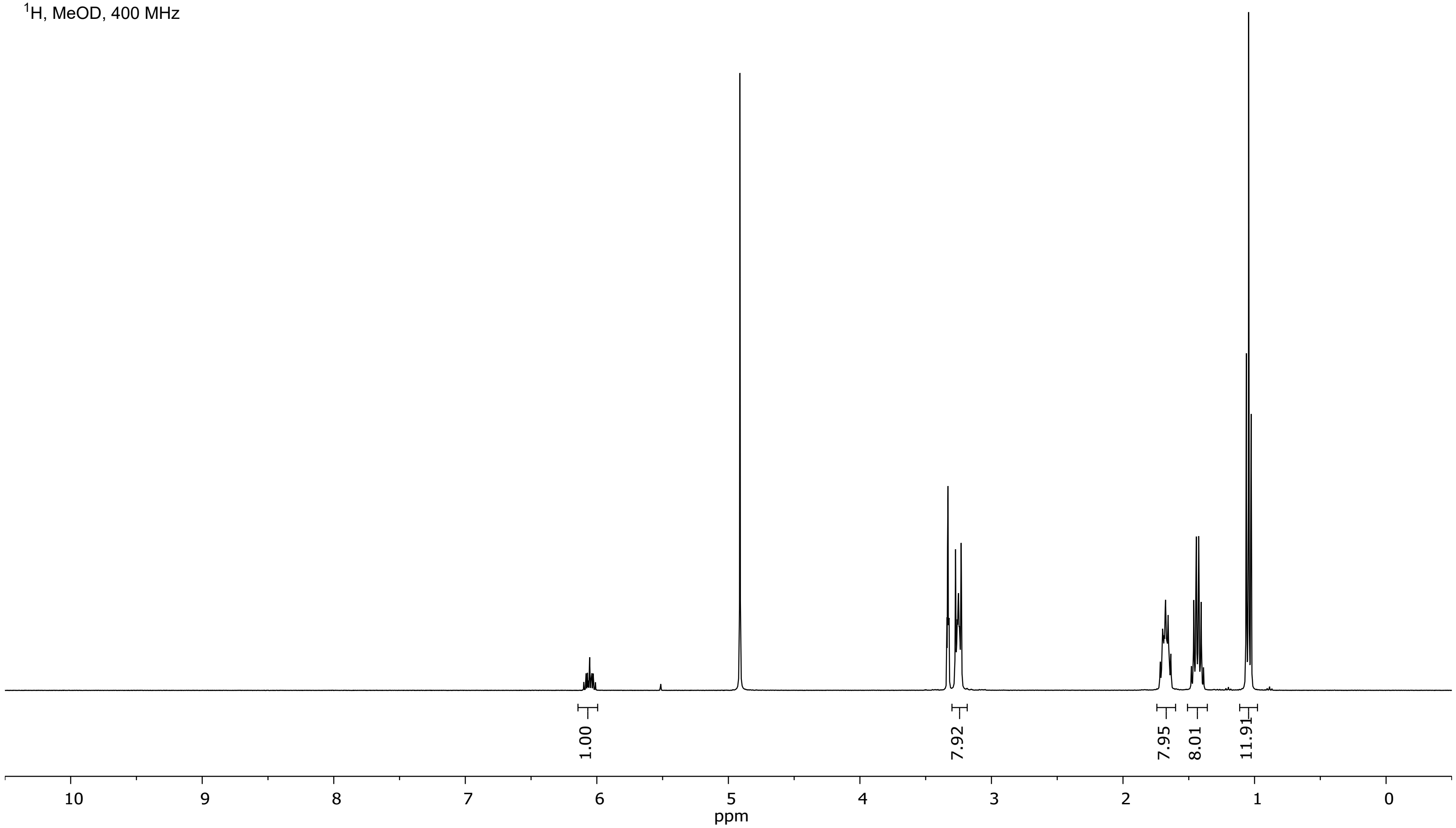

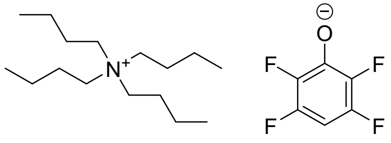

S1

$^{19}F\{^{1}H\}$, MeOD, 377 MHz

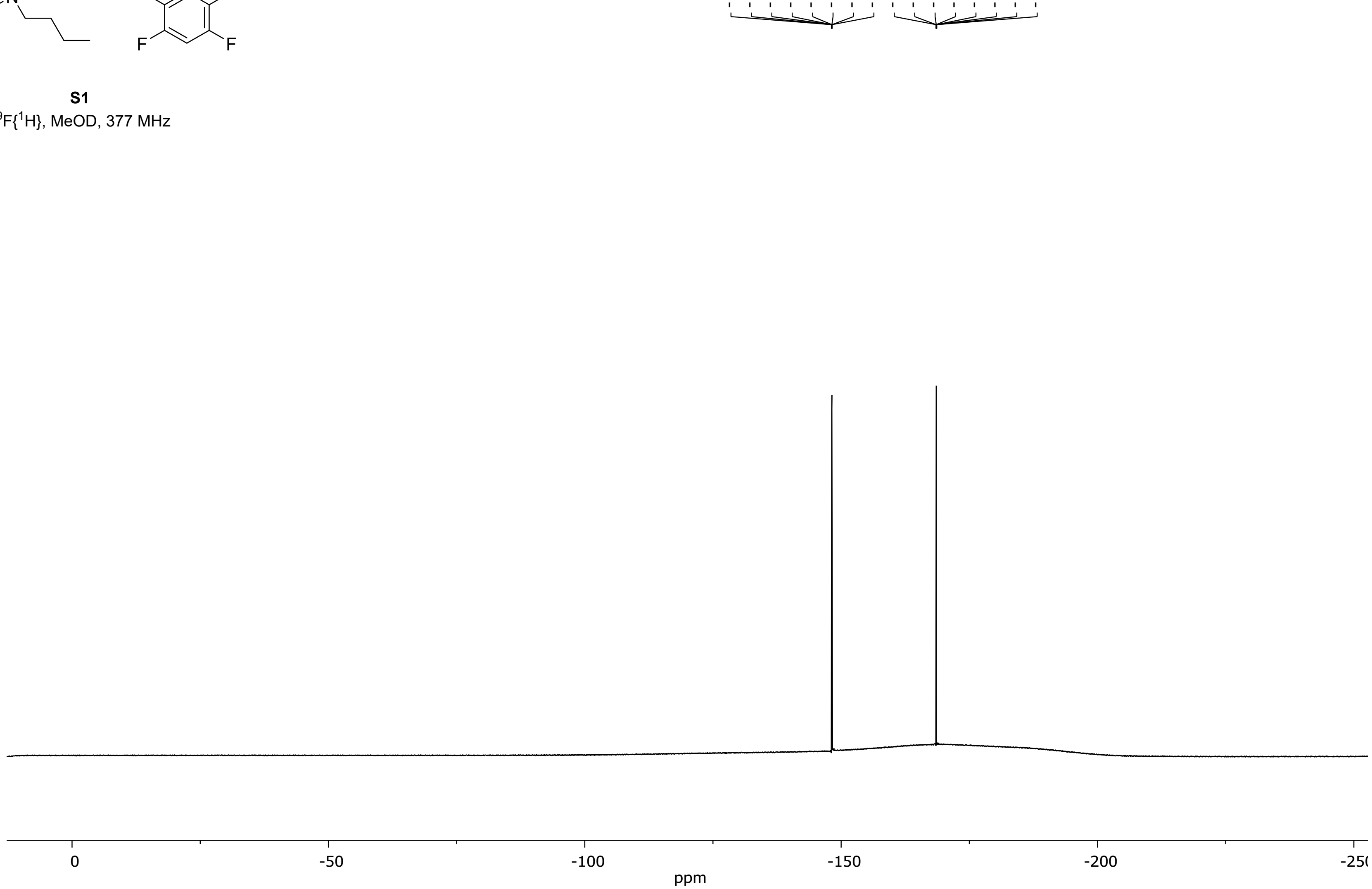

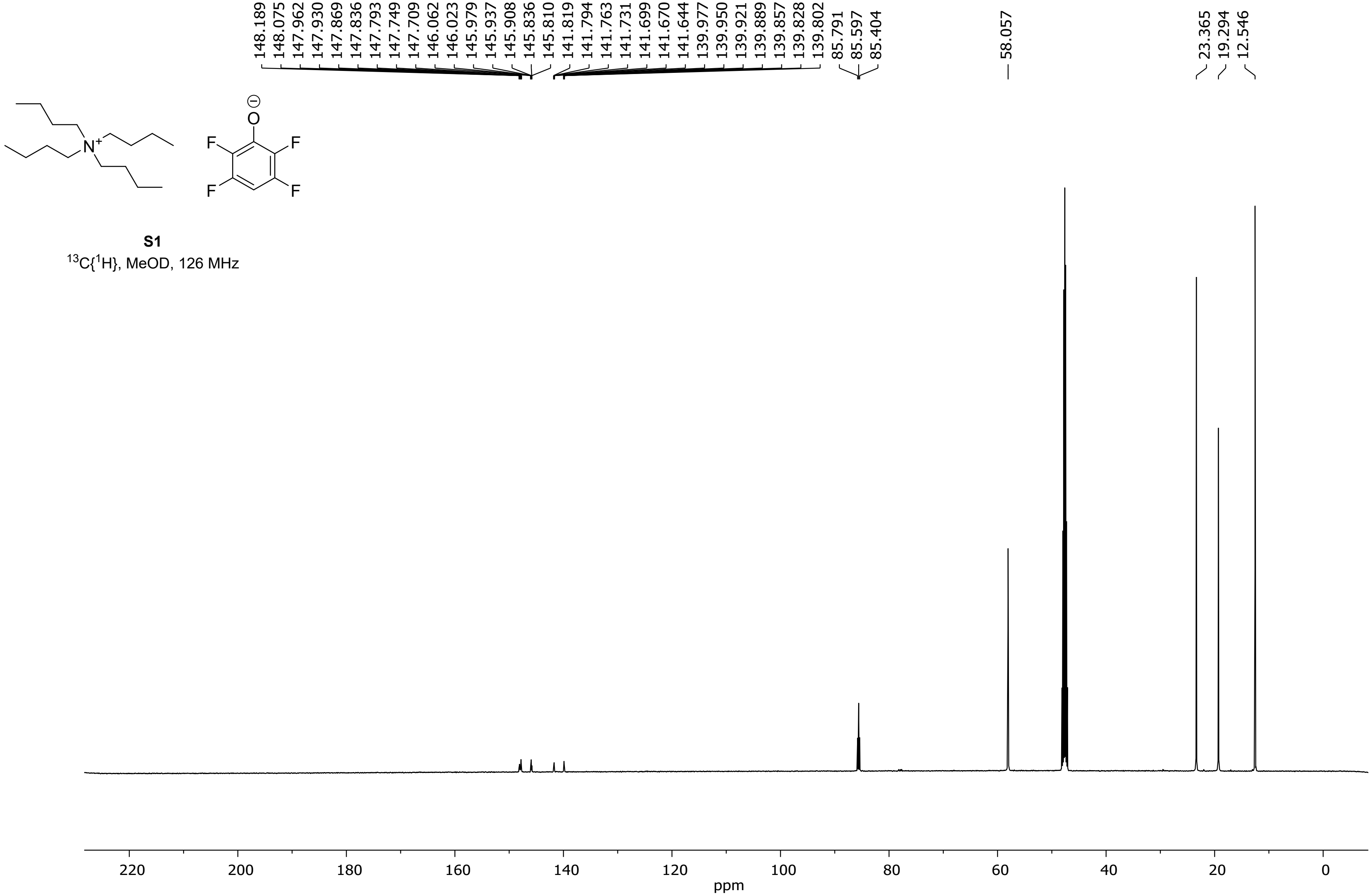
148.189
148.075
147.962
147.930
147.869
147.836
147.793
147.749
147.709
146.062
146.023
145.979
145.937
145.908
145.836
145.810
141.819
141.794
141.763
141.731
141.699
141.670
141.644
139.977
139.950
139.921
139.889
139.857
139.828
139.802
85.791
85.597
85.404
58.057
23.365
19.294
12.546
S1
$^{13}C\{^{1}H\}$, MeOD, 126 MHz
ppm

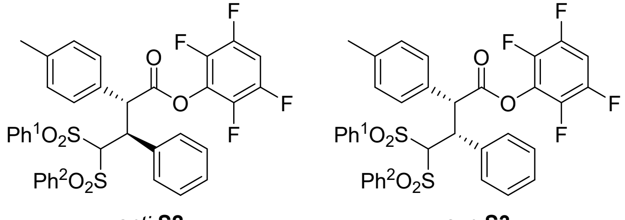

^{1}H NMR, $CDCl_3$, 500 MHz

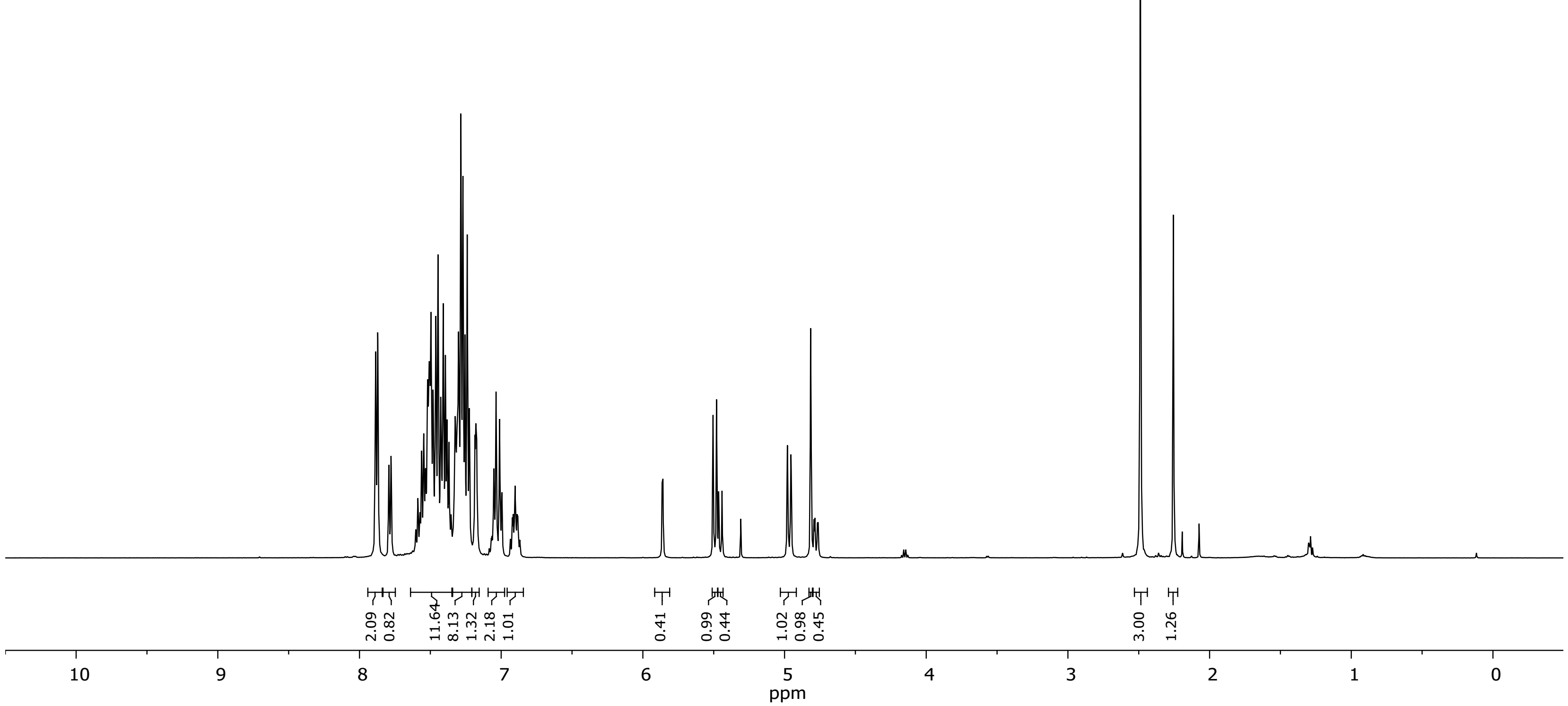

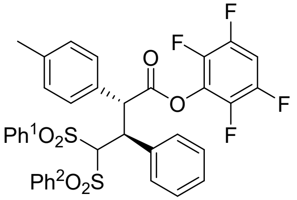

anti-**S2**

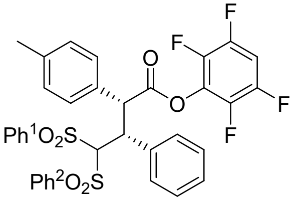

syn-**S3**

$^{19}F\{^1H\}$ NMR, $CDCl_3$, 470 MHz

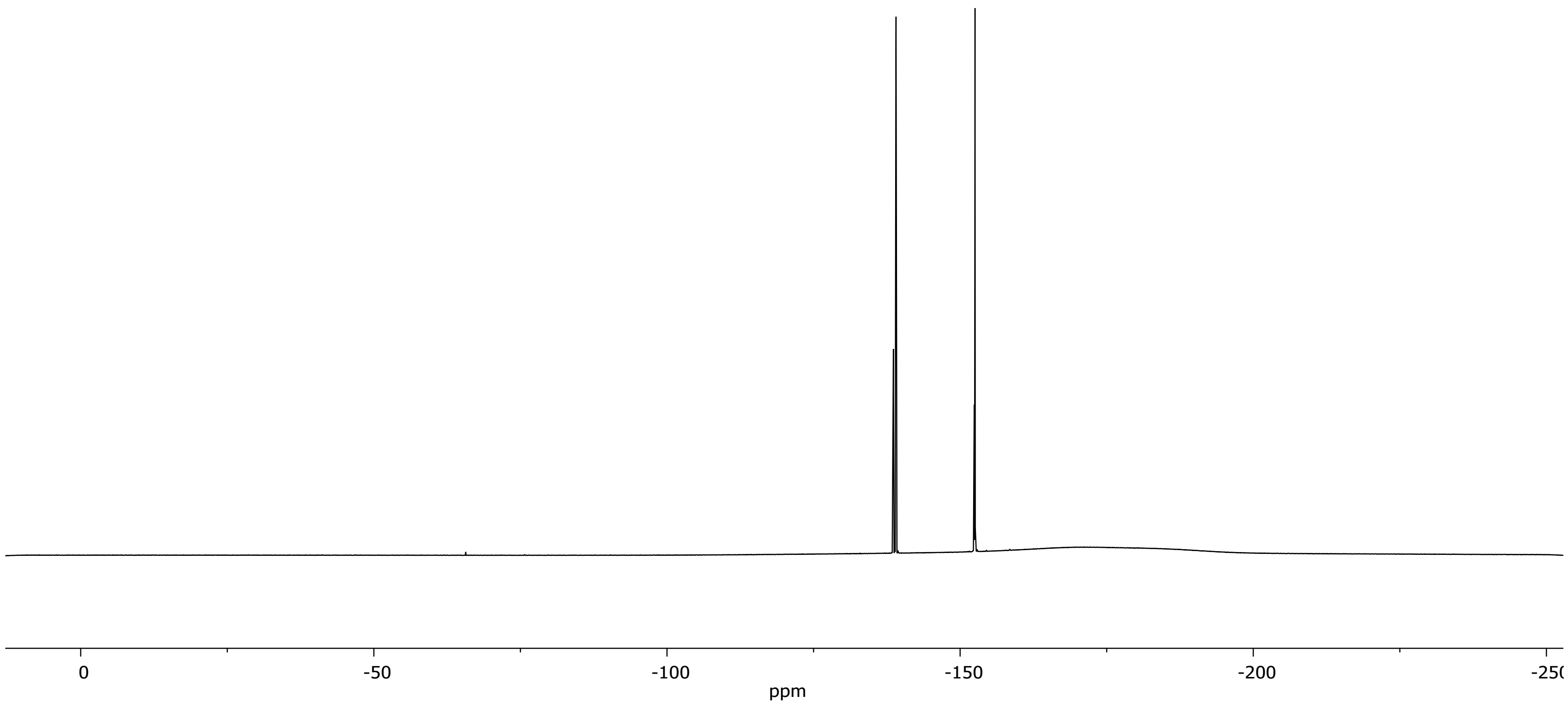

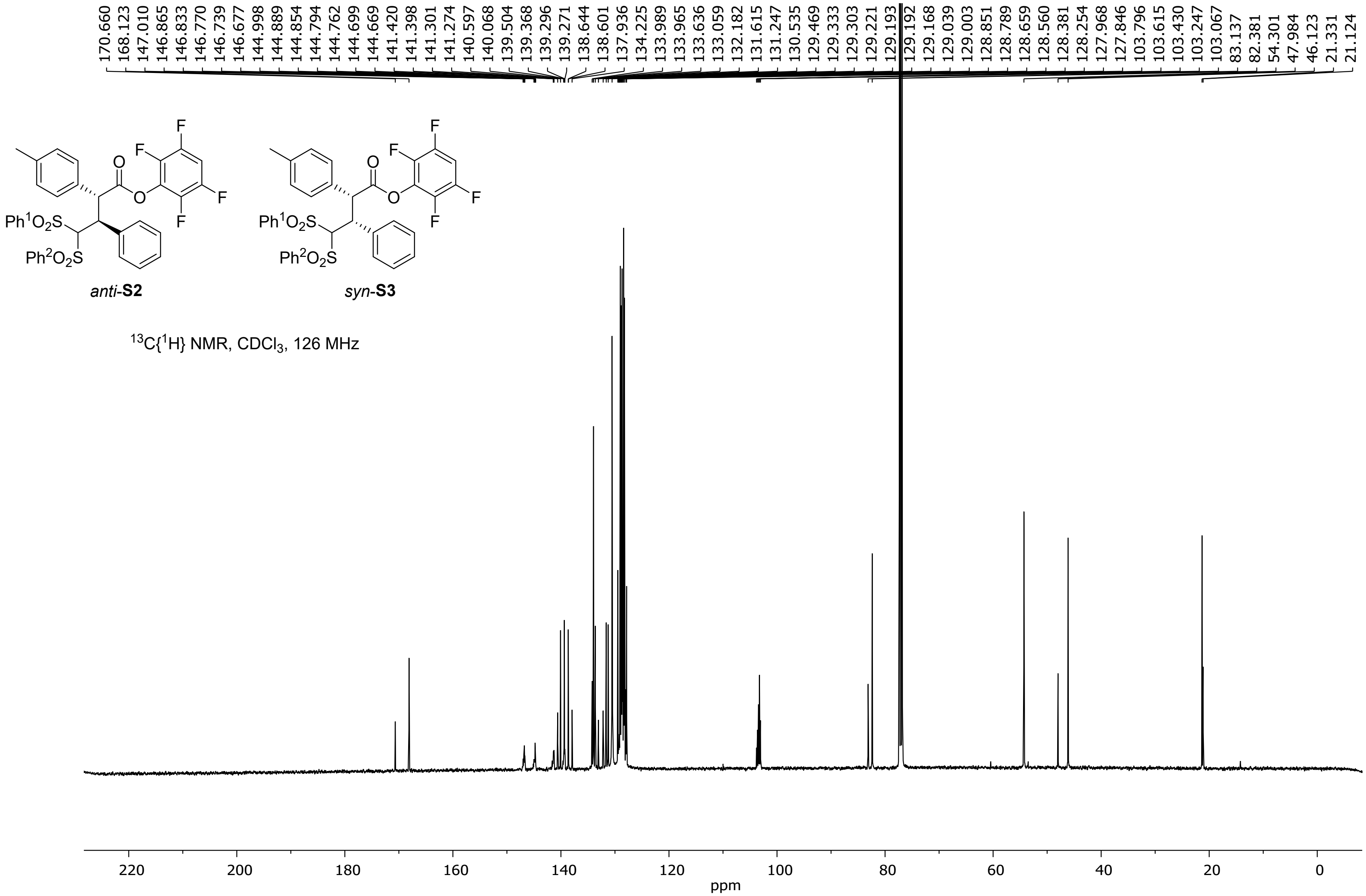
170.660
168.123
147.010
146.865
146.833
146.770
146.739
146.677
144.998
144.889
144.854
144.794
144.762
144.699
144.669
141.420
141.398
141.301
141.274
140.597
140.068
139.504
139.368
139.296
139.271
138.644
138.601
137.936
134.225
133.989
133.965
133.636
133.059
132.182
131.615
131.247
130.535
129.469
129.333
129.303
129.221
129.193
129.192
129.168
129.039
129.003
128.851
128.789
128.659
128.560
128.381
128.254
127.968
127.846
103.796
103.615
103.430
103.247
103.067
83.137
82.381
54.301
47.984
46.123
21.331
21.124
Ph1O2S
Ph2O2S
anti-S2
syn-S3
13C{1H} NMR, CDCl3, 126 MHz
ppm

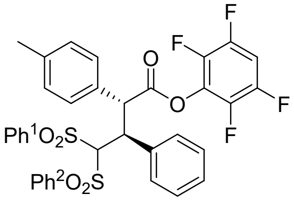

anti-**S2**

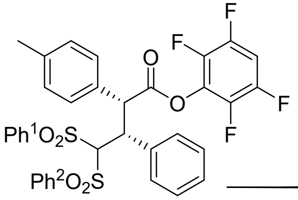

syn-**S3**

2D, ^{1}H-^{1}H COSY, $CDCl_3$

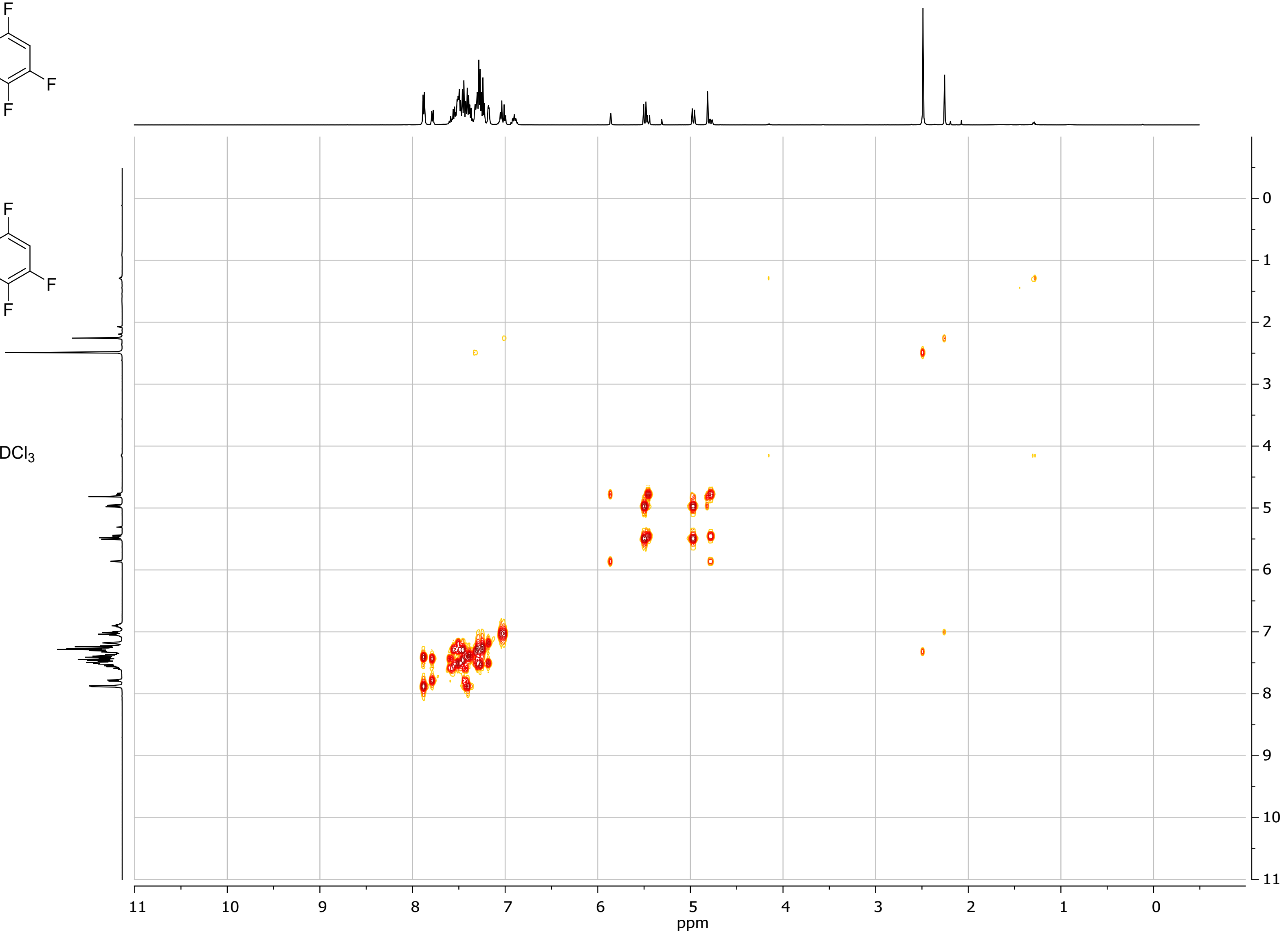

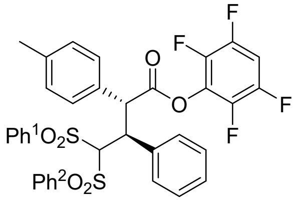

anti-**S2**

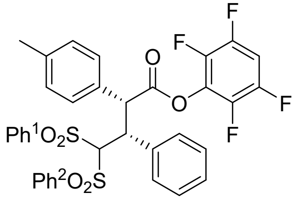

syn-**S3**

2D, ^{1}H-^{13}C HSQC, $CDCl_3$

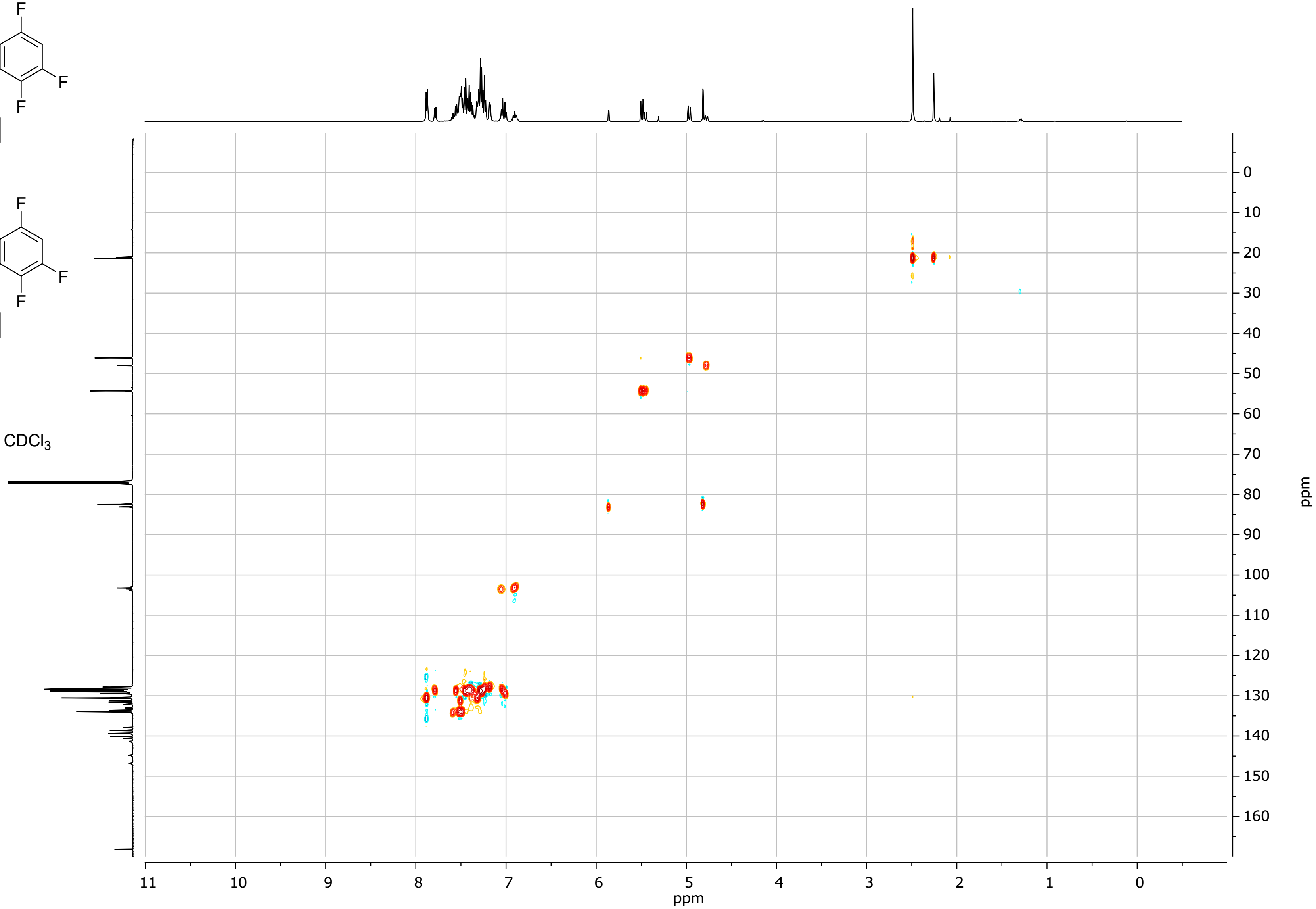

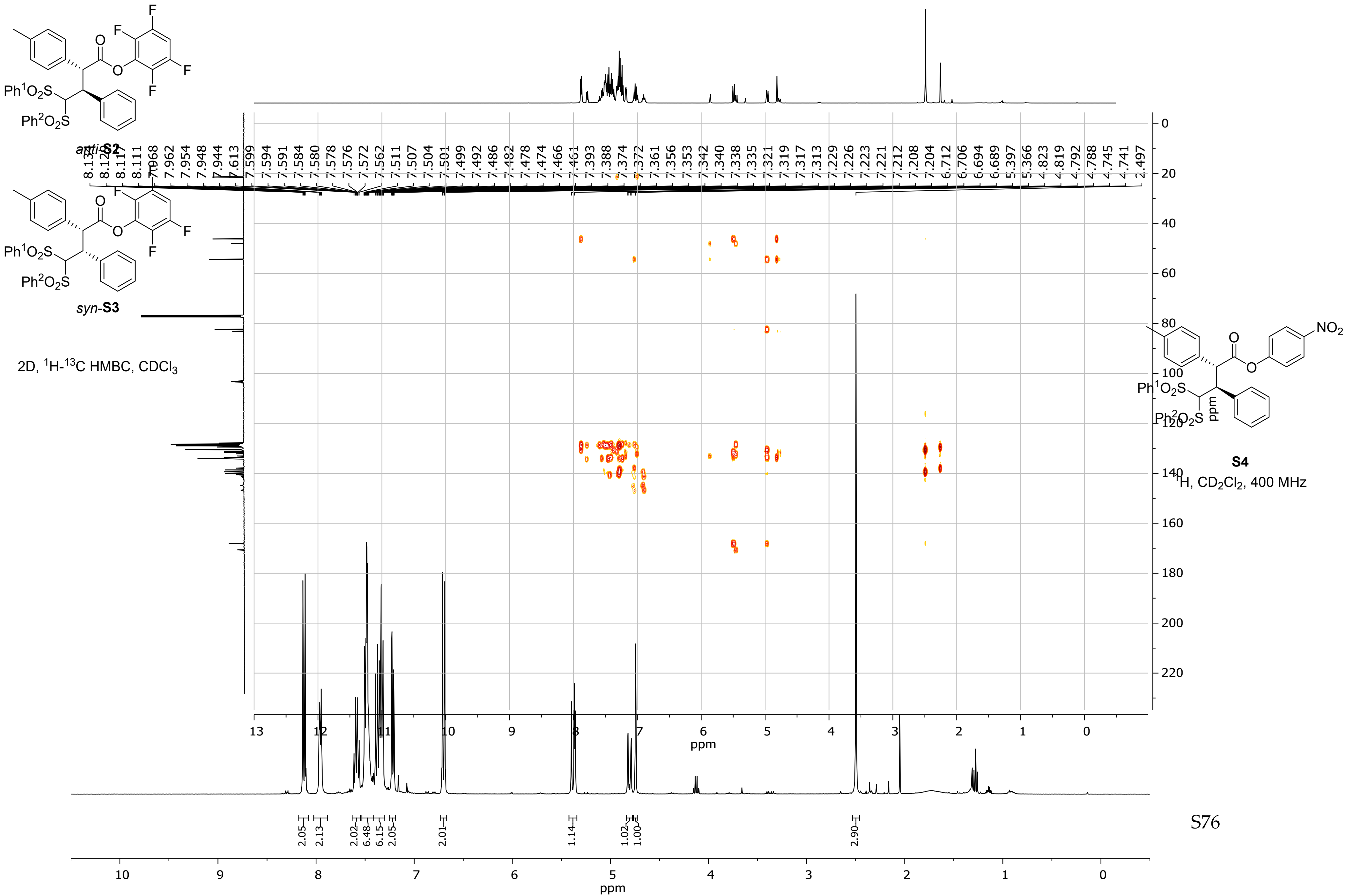
anti-S2
syn-S3
2D, 1H-13C HMBC, CDCl3
S4
1H, CD2Cl2, 400 MHz
ppm
8.131 8.121 8.117 8.111 7.968 7.962 7.954 7.948 7.944 7.613 7.599 7.594 7.591 7.584 7.580 7.578 7.576 7.572 7.562 7.511 7.507 7.504 7.501 7.499 7.492 7.486 7.482 7.478 7.474 7.466 7.461 7.393 7.388 7.374 7.372 7.361 7.356 7.353 7.342 7.340 7.338 7.335 7.321 7.319 7.317 7.313 7.229 7.226 7.223 7.221 7.212 7.208 7.204 6.712 6.706 6.694 6.689 5.397 5.366 4.823 4.819 4.792 4.788 4.745 4.741 2.497
2.05 2.13 2.02 6.48 6.15 2.05 2.01 1.14 1.02 1.00 2.90

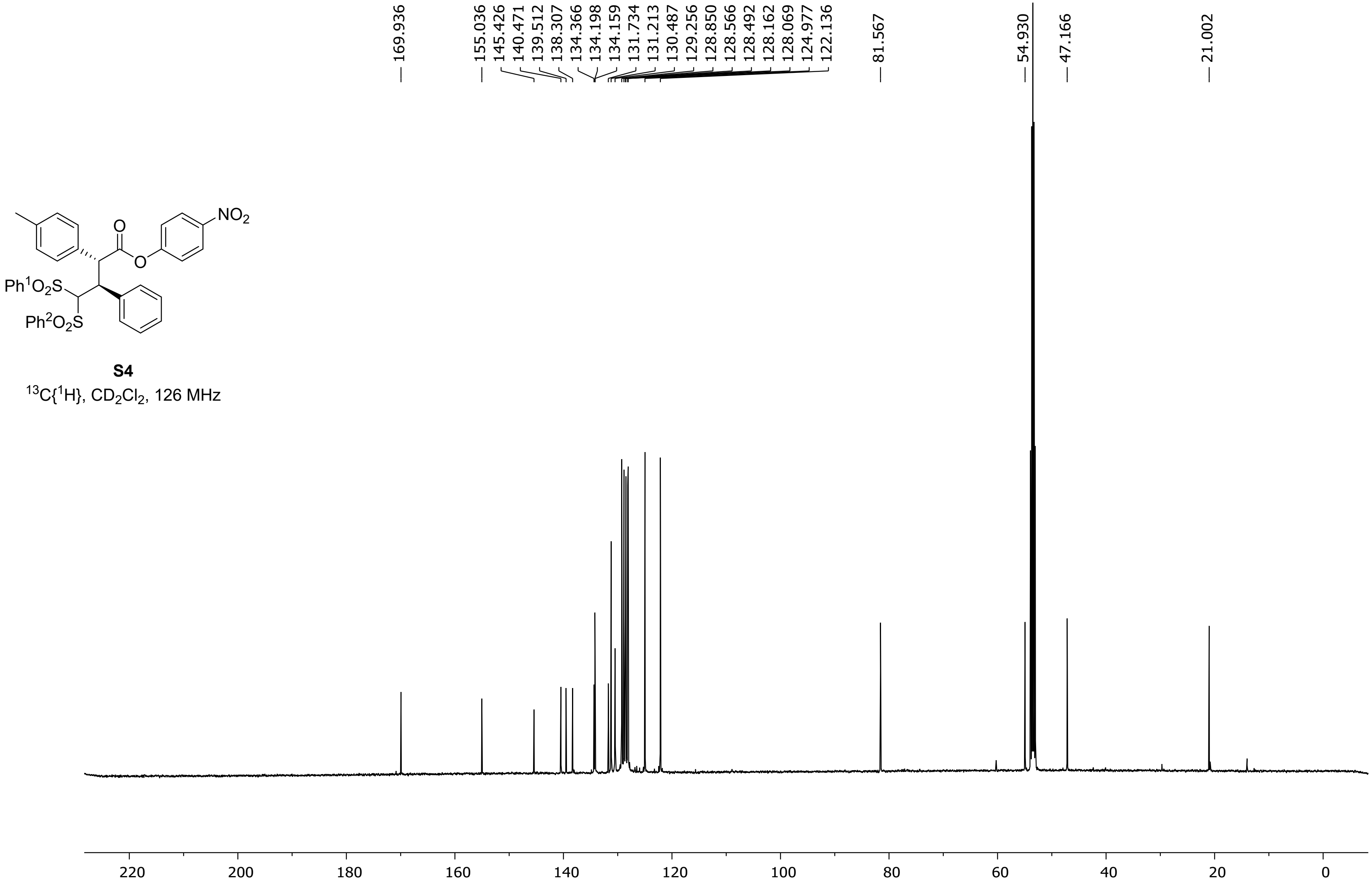

S4

$^{13}C\{^{1}H\}$, CD_2Cl_2, 126 MHz

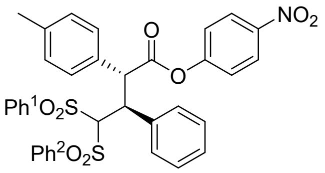

S4

2D ^1H-^1H COSY, CD_2Cl_2

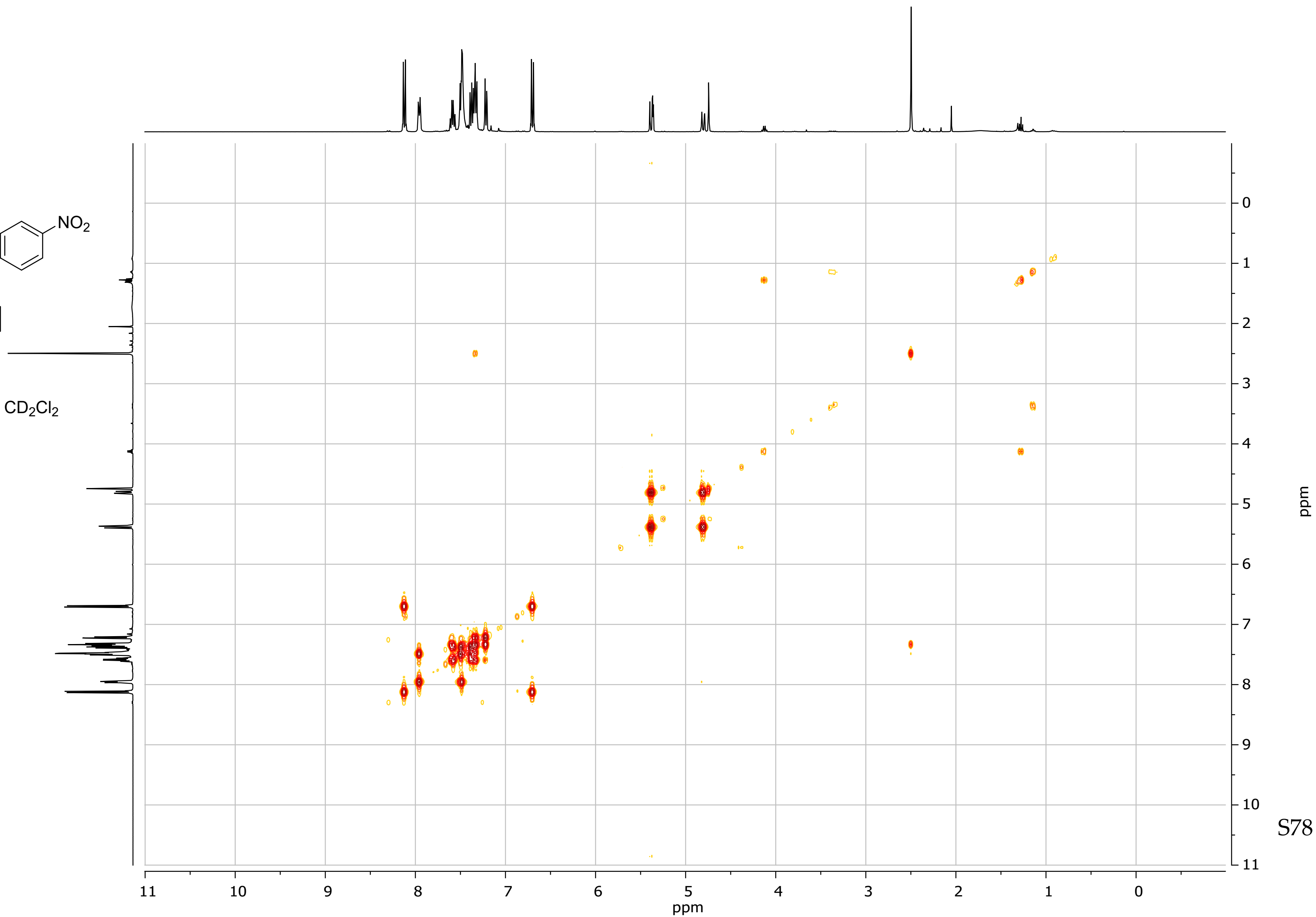

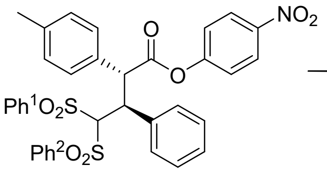

S4

2D ^{1}H-^{13}C HSQC, CD_2Cl_2

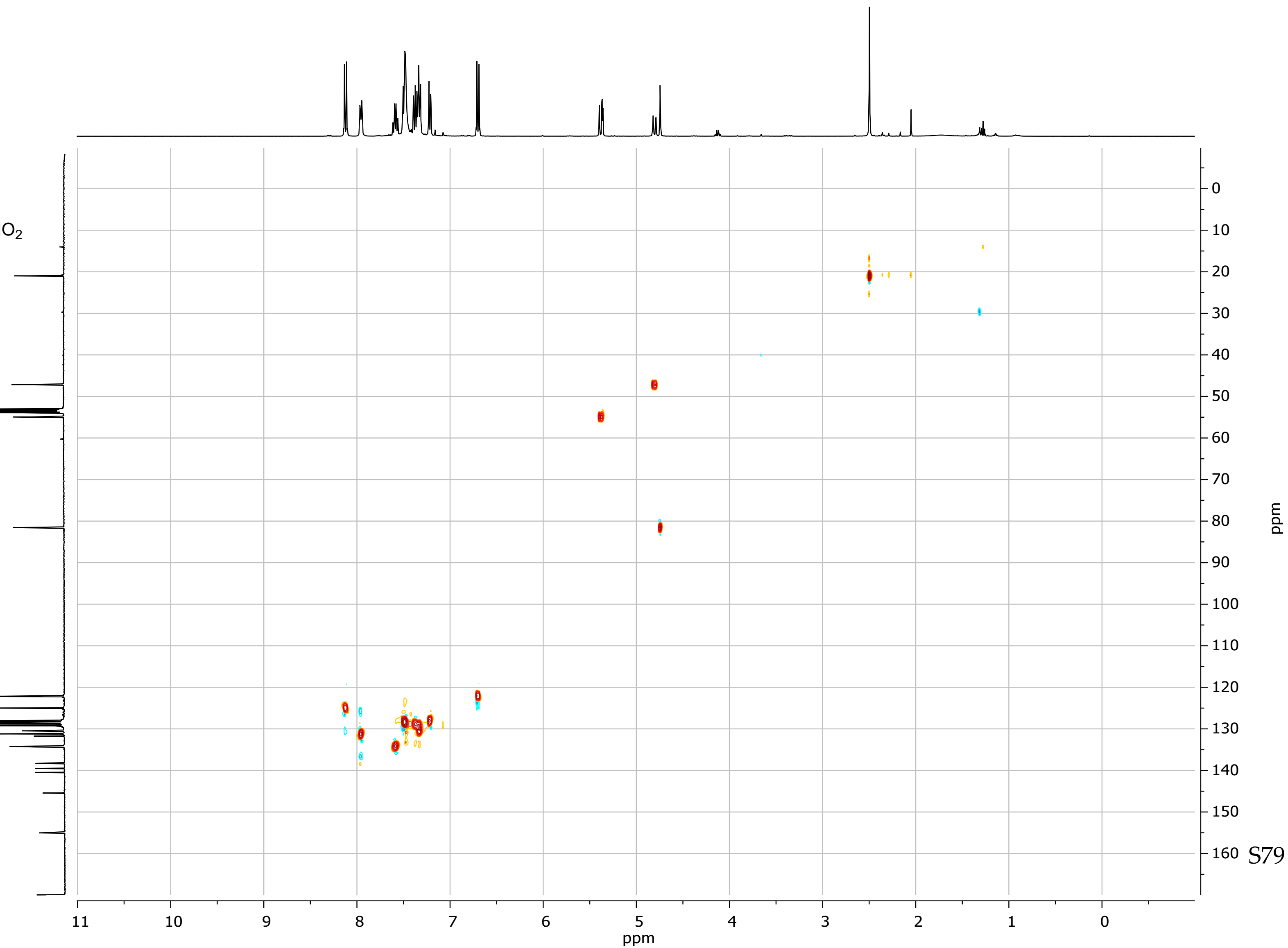

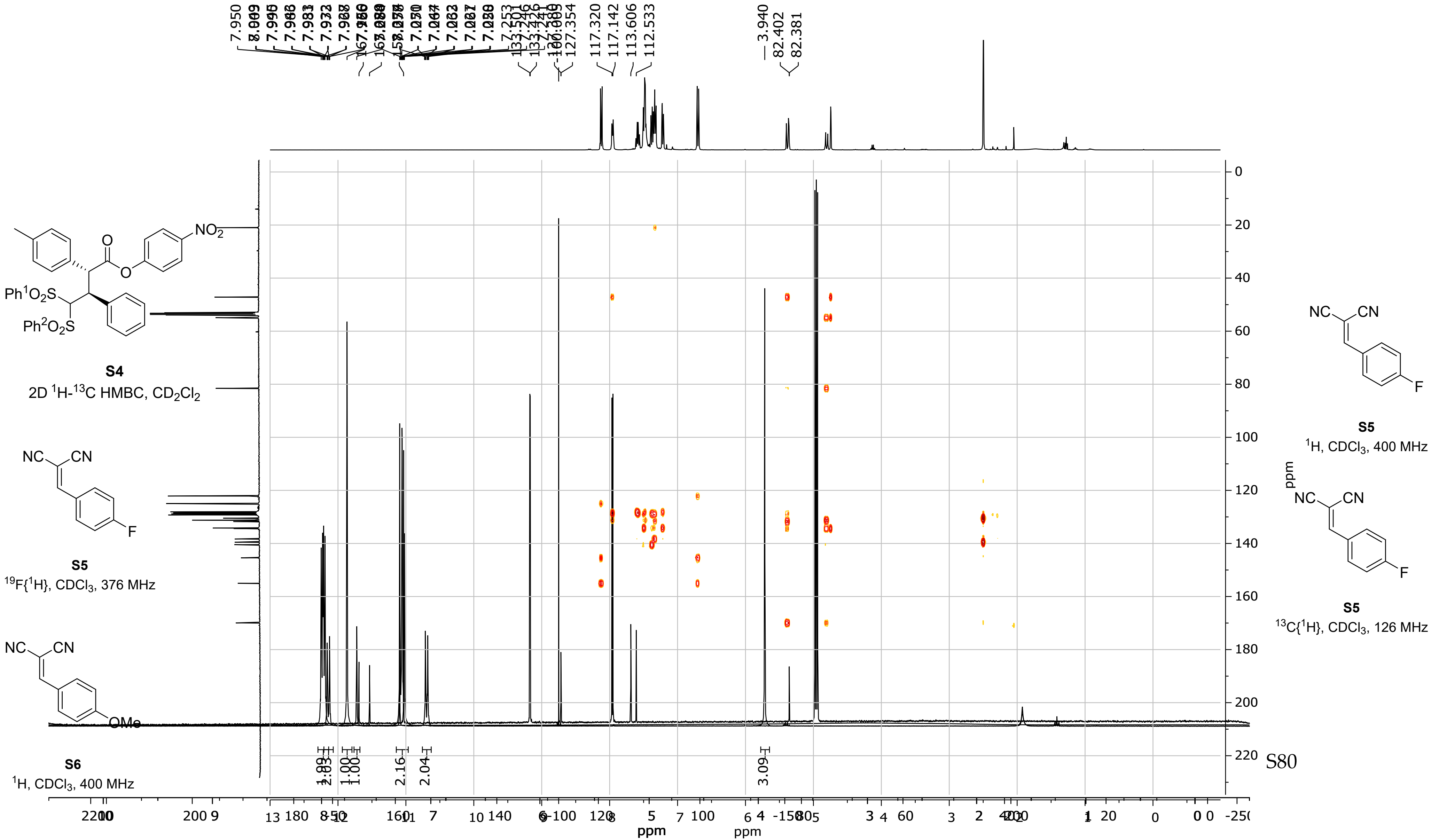
S4
2D ^{1}H-^{13}C HMBC, CD_2Cl_2
Ph^1O_2S
Ph^2O_2S
NO_2
S5
^{19}F{^{1}H}, $CDCl_3$, 376 MHz
S6
^{1}H, $CDCl_3$, 400 MHz
S5
^{1}H, $CDCl_3$, 400 MHz
S5
^{13}C{^{1}H}, $CDCl_3$, 126 MHz
NC
CN
F
OMe
ppm

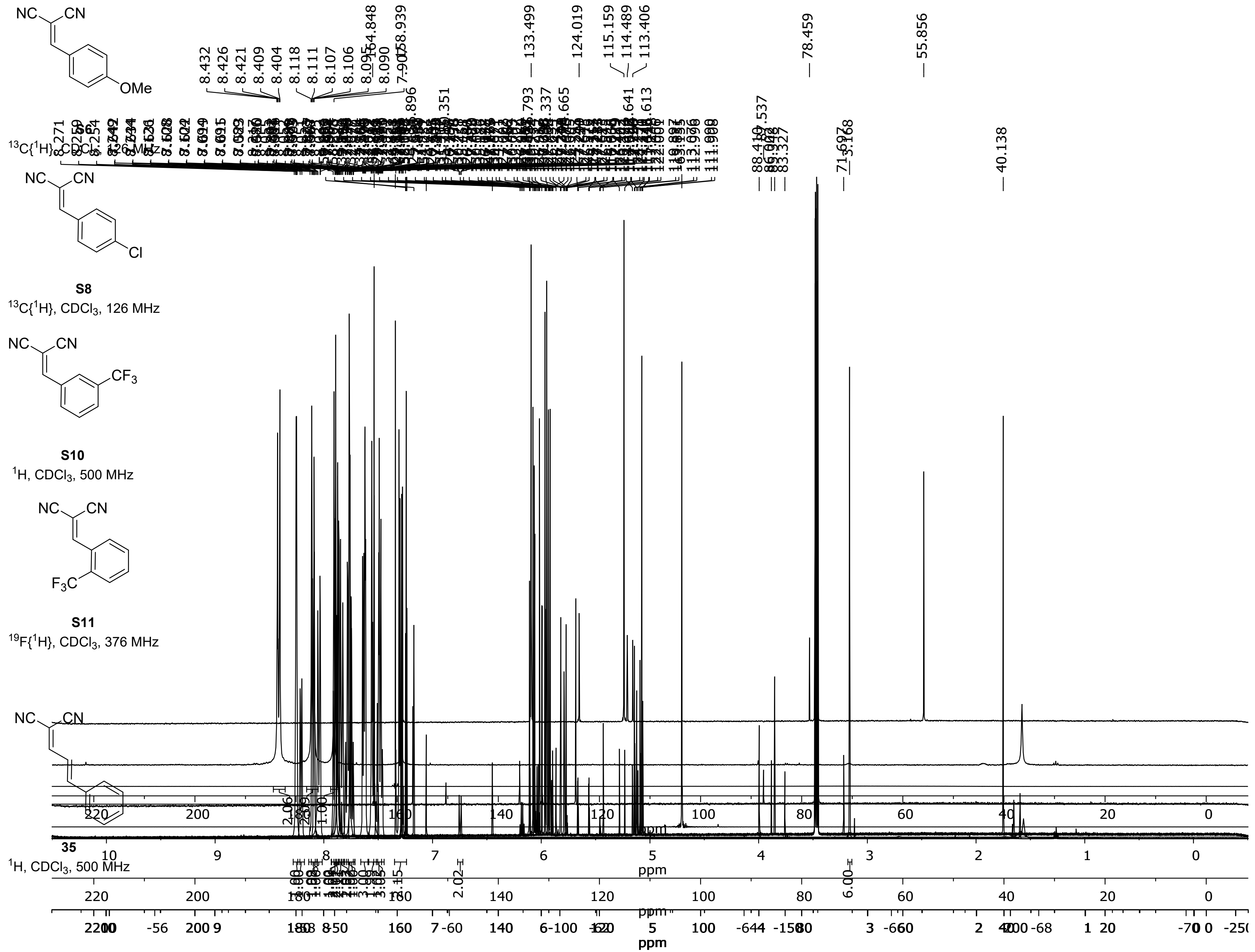
133.499
124.019
115.159
114.489
113.406
78.459
55.856
40.138
OMe
NC
CN
Cl
S8
13C{1H}, CDCl3, 126 MHz
CF3
S10
1H, CDCl3, 500 MHz
F3C
S11
19F{1H}, CDCl3, 376 MHz
35
1H, CDCl3, 500 MHz
ppm

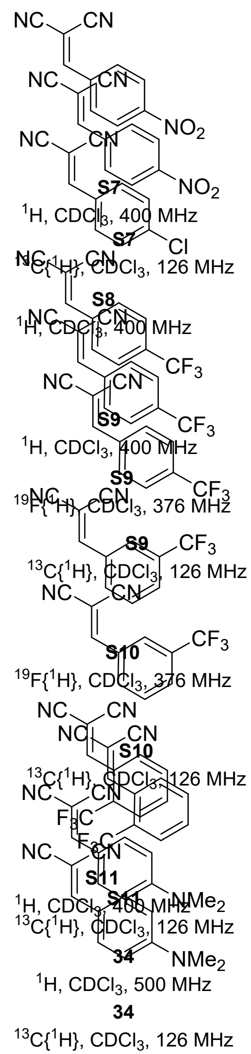
NO2
S7
1H, CDCl3, 400 MHz
CF3
S9
19F{1H}, CDCl3, 376 MHz
13C{1H}, CDCl3, 126 MHz
S10
NMe2
34
1H, CDCl3, 500 MHz
13C{1H}, CDCl3, 126 MHz

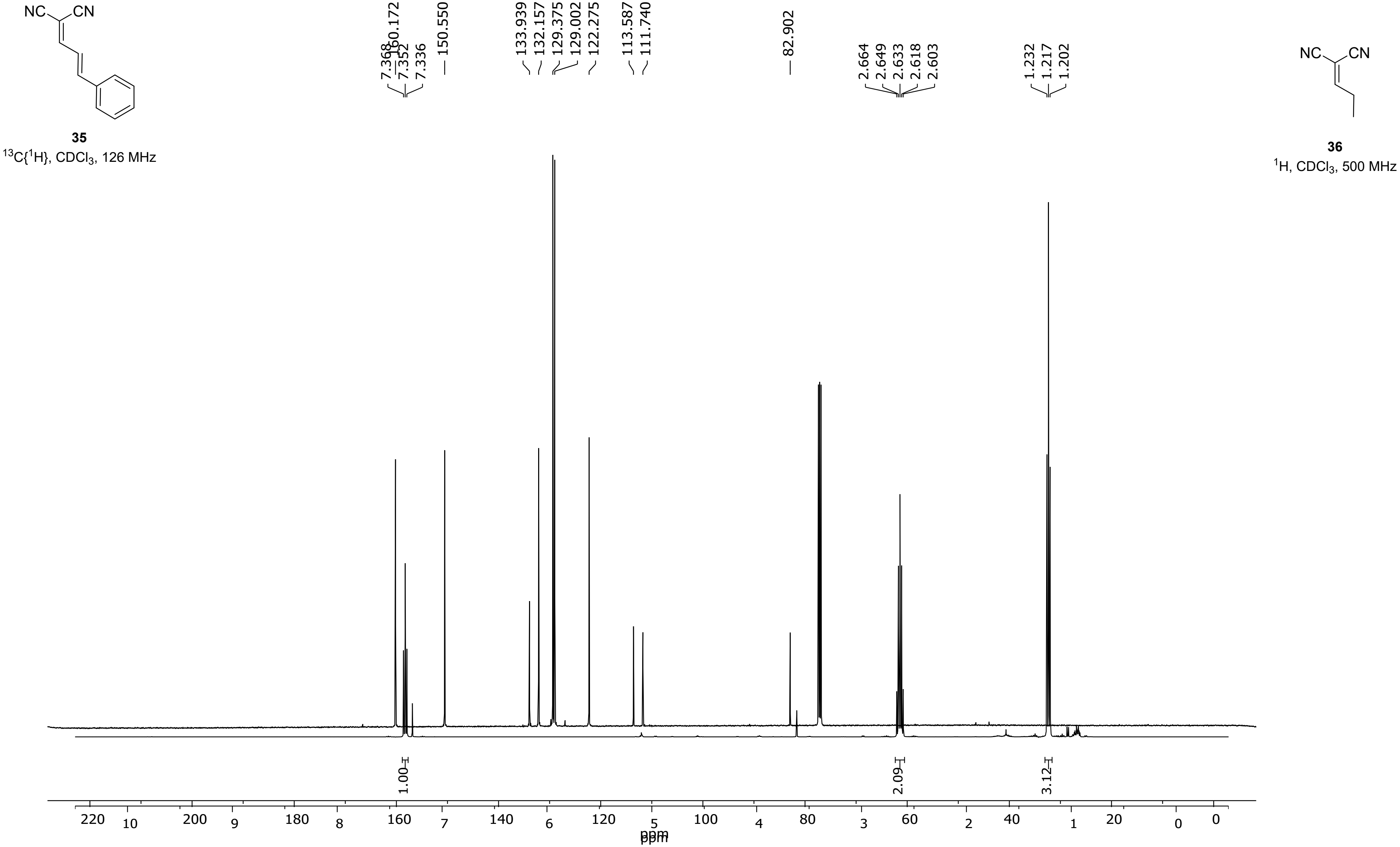
NC CN
35
$^{13}C\{^{1}H\}$, $CDCl_3$, 126 MHz
NC CN
36
^{1}H, $CDCl_3$, 500 MHz
7.368
160.172
7.352
7.336
150.550
133.939
132.157
129.375
129.002
122.275
113.587
111.740
82.902
2.664
2.649
2.633
2.618
2.603
1.232
1.217
1.202
1.00
2.09
3.12
220 200 180 160 140 120 100 80 60 40 20 0
10 9 8 7 6 5 4 3 2 1 0
ppm

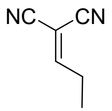

36

$^{13}C\{^{1}H\}$, $CDCl_3$, 126 MHz

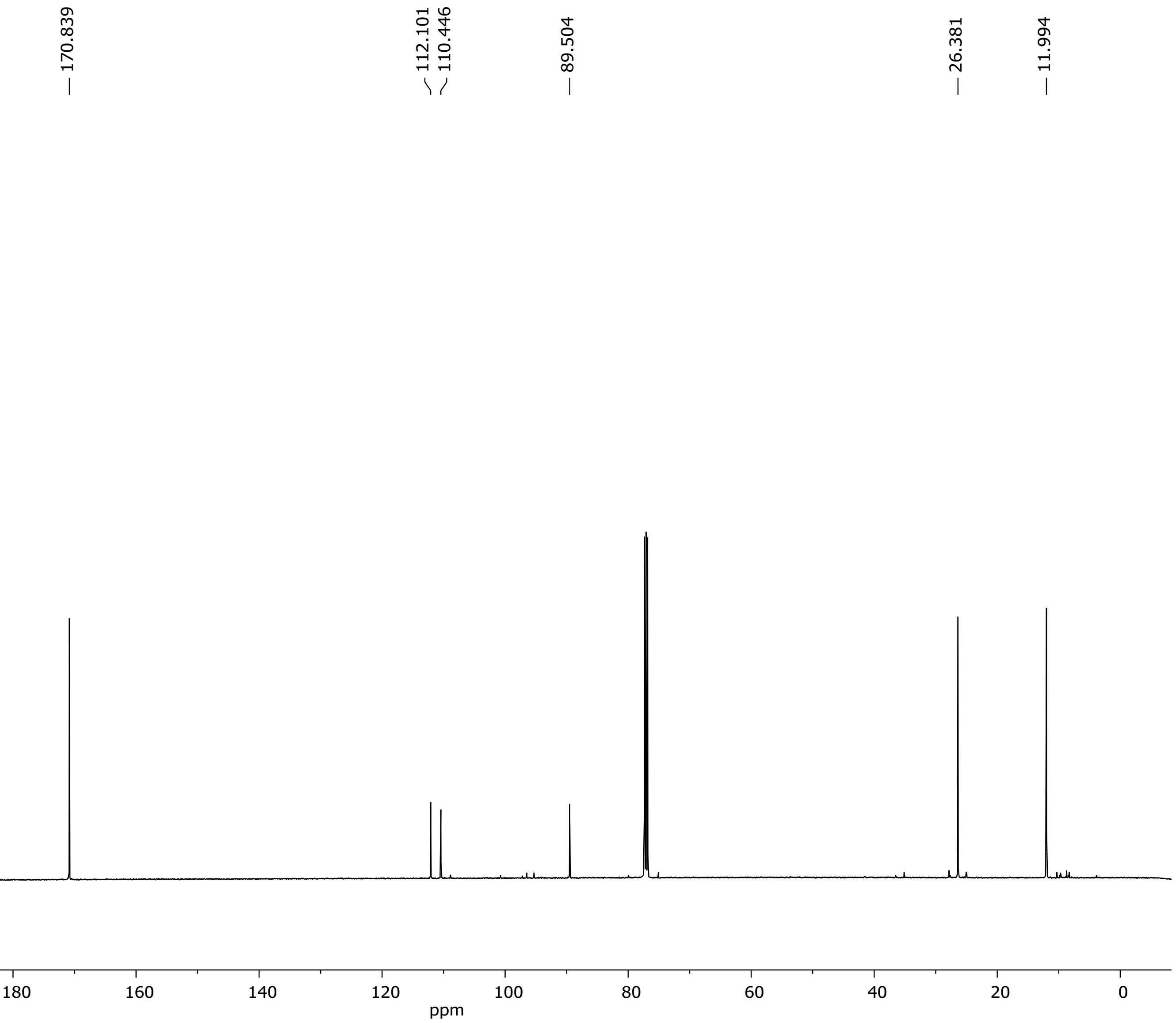

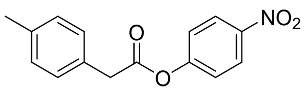

1

^{1}H, $CDCl_3$, 400 MHz

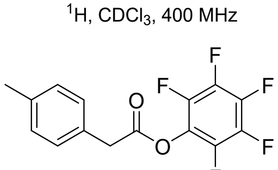

8

^{1}H, $CDCl_3$, 500 MHz

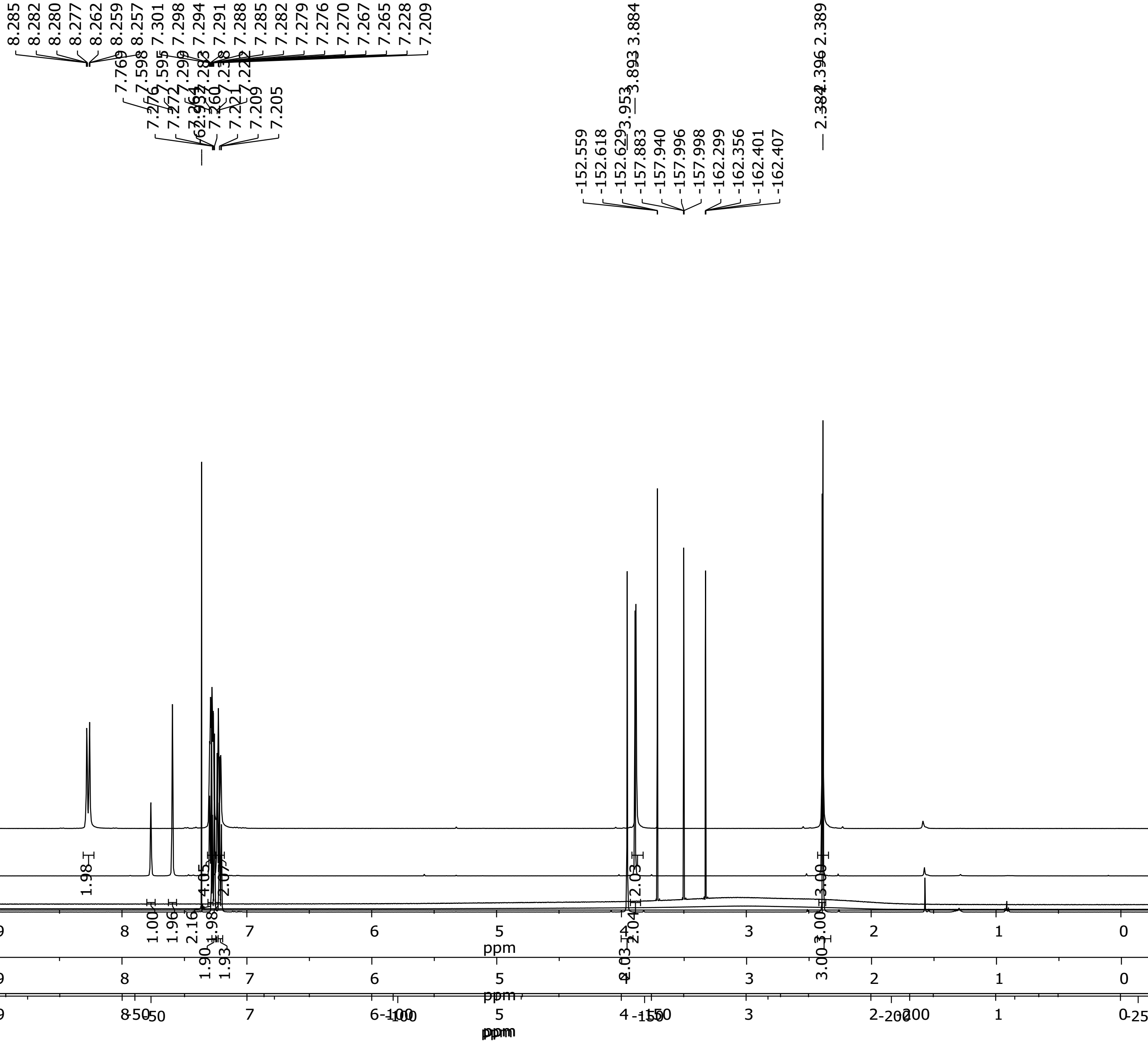

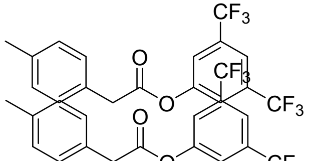

6

^{1}H, $CDCl_3$, 500 MHz

6

^{19}F{^{1}H}, $CDCl_3$, 470 MHz

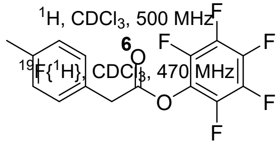

8

^{19}F{^{1}H}, $CDCl_3$, 376 MHz

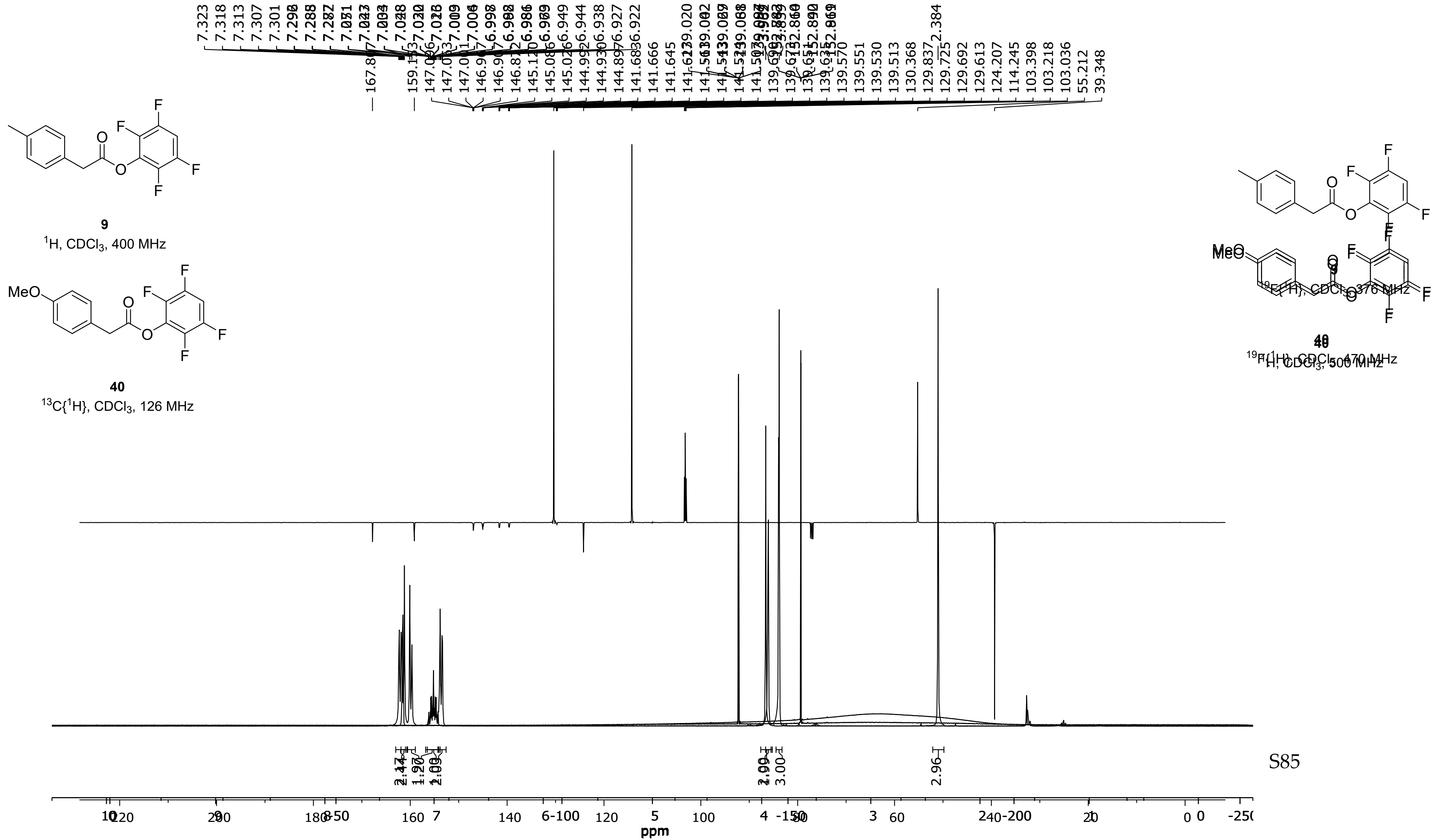
9
1H, CDCl3, 400 MHz
40
13C{1H}, CDCl3, 126 MHz
ppm

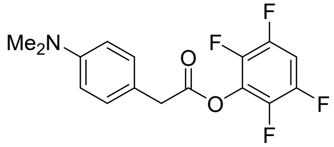

S12

^{1}H, $CDCl_3$, 400 MHz

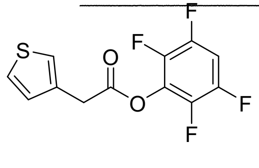

S13

$^{13}C\{^{1}H\}$, $CDCl_3$, 126 MHz

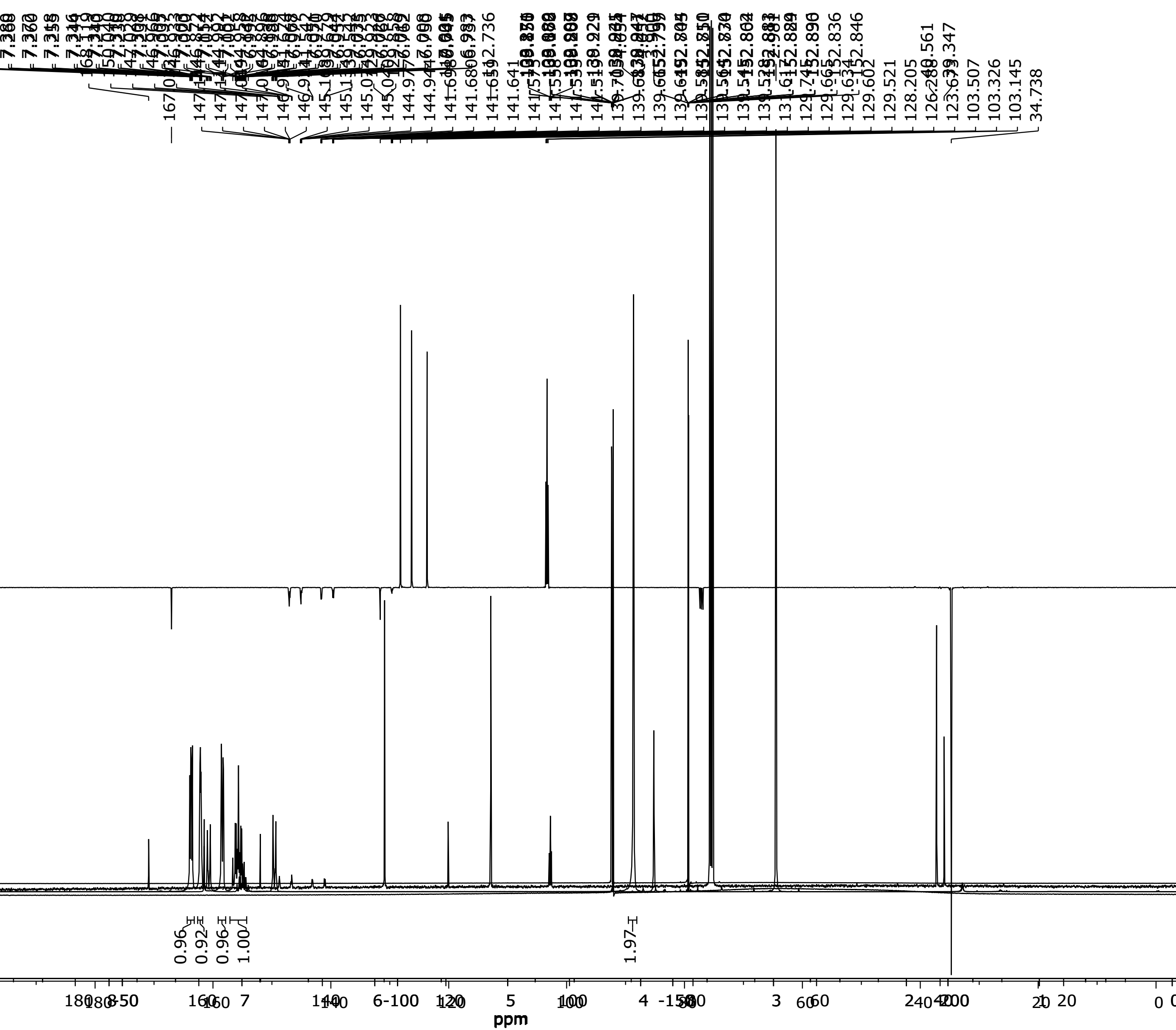

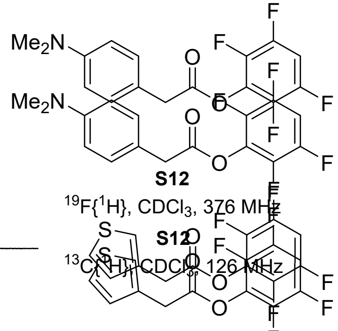

S12

$^{19}F\{^{1}H\}$, $CDCl_3$, 376 MHz

S12

$^{13}C\{^{1}H\}$, $CDCl_3$, 126 MHz

S13

S13

$^{19}F\{^{1}H\}$, $CDCl_3$, 376 MHz

^{1}H, $CDCl_3$, 400 MHz

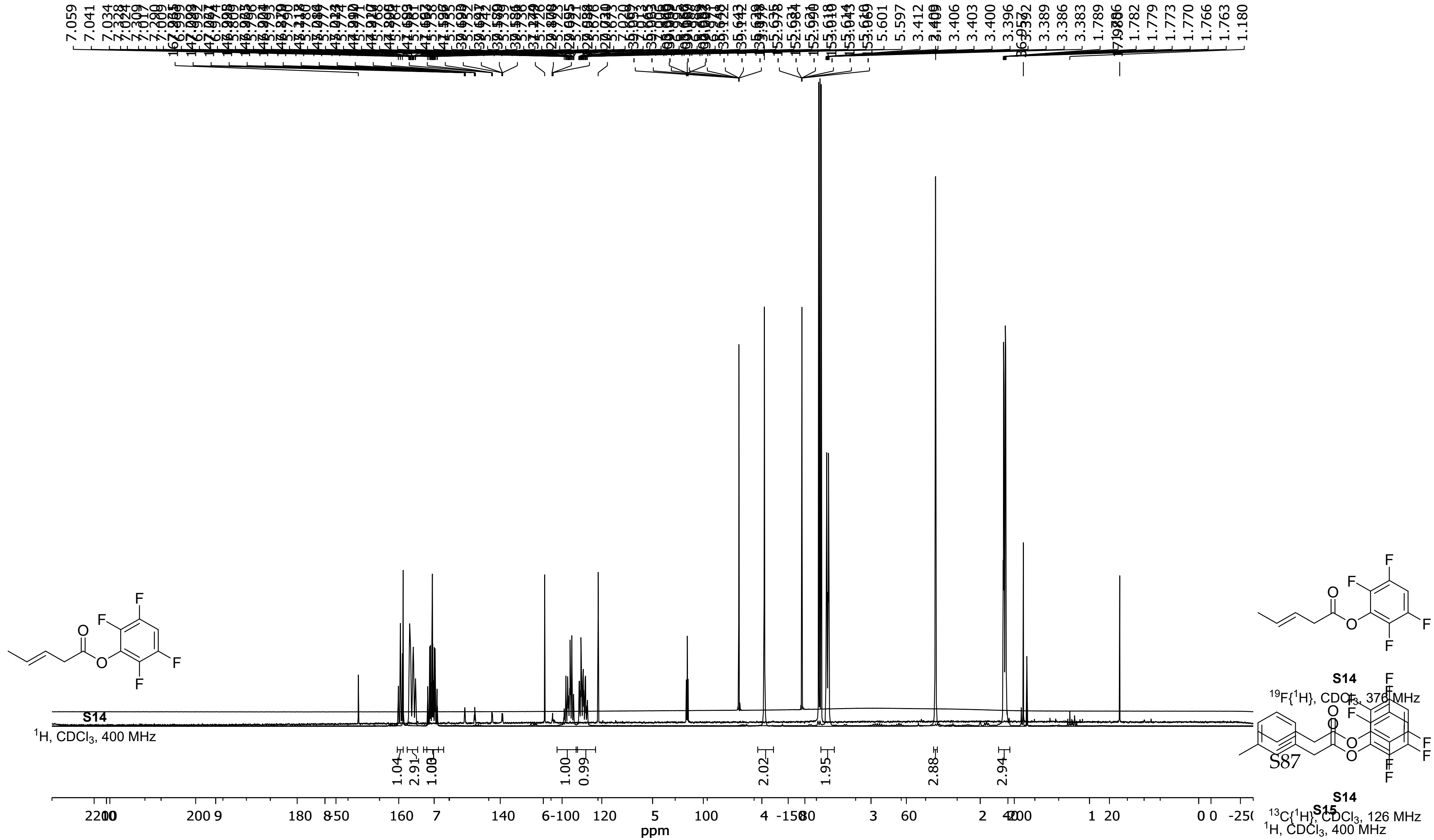
S14
^{1}H, CDCl$_3$, 400 MHz
S14
^{19}F{^{1}H}, CDCl$_3$, 376 MHz
S14
^{13}C{^{1}H}, CDCl$_3$, 126 MHz
S15
^{1}H, CDCl$_3$, 400 MHz
ppm

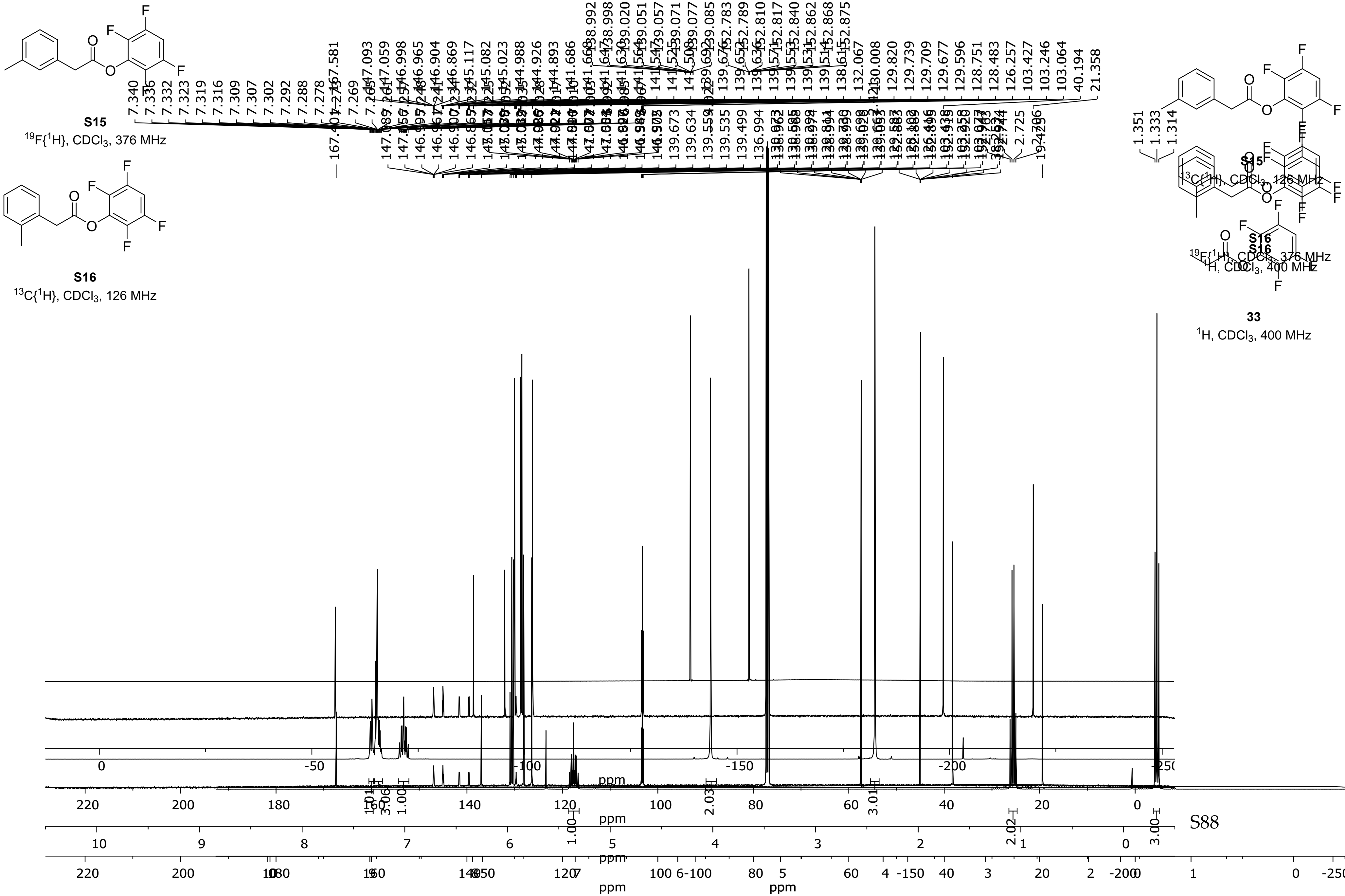

S15
^{19}F{^{1}H}, CDCl$_3$, 376 MHz
S16
^{13}C{^{1}H}, CDCl$_3$, 126 MHz
33
^{1}H, CDCl$_3$, 400 MHz
ppm

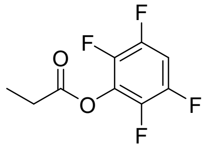

33

$^{19}F\{^{1}H\}$, $CDCl_3$, 376 MHz

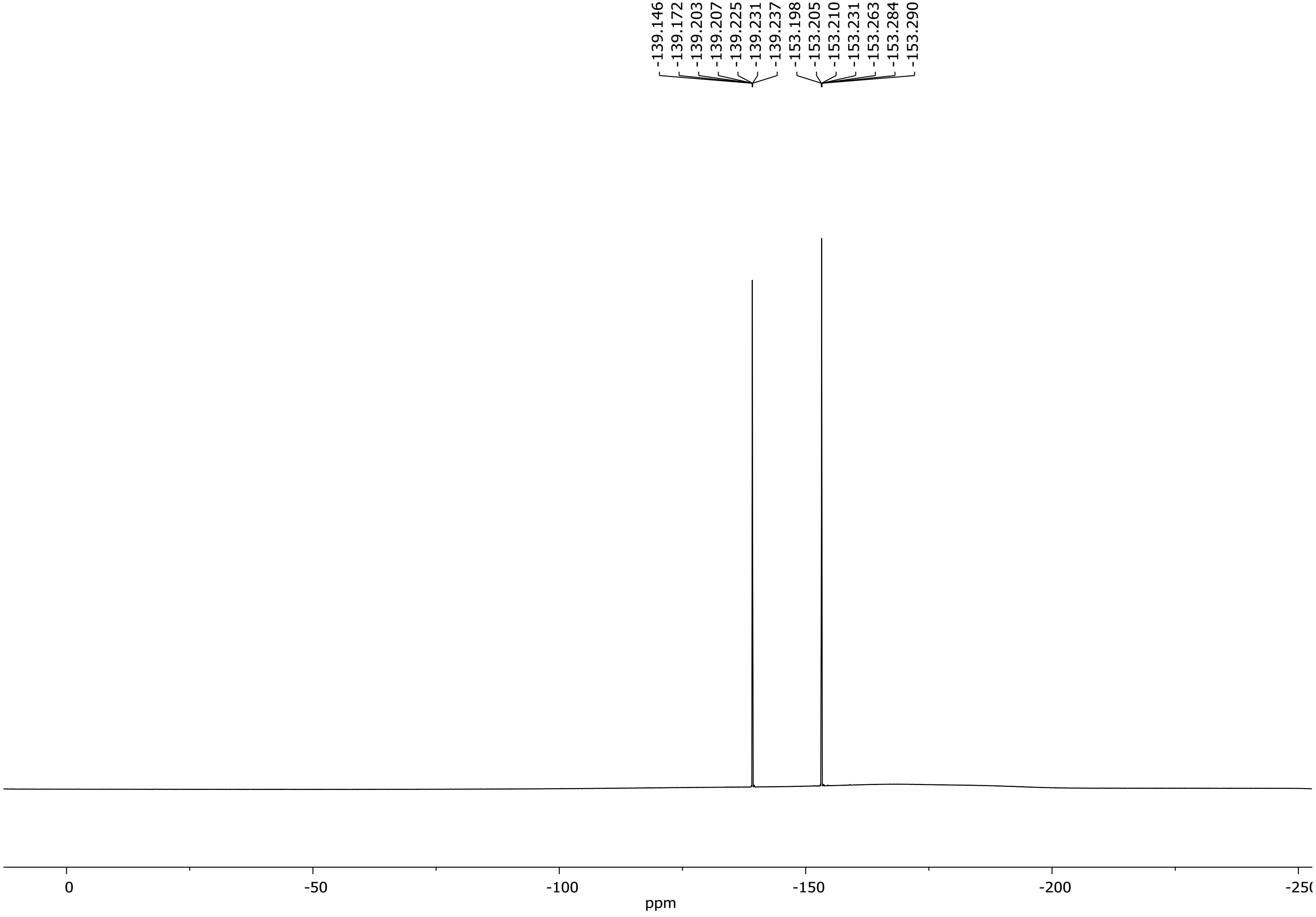

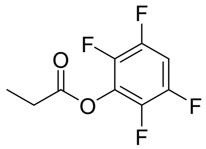

33

$^{13}C\{^1H\}$, $CDCl_3$, 126 MHz

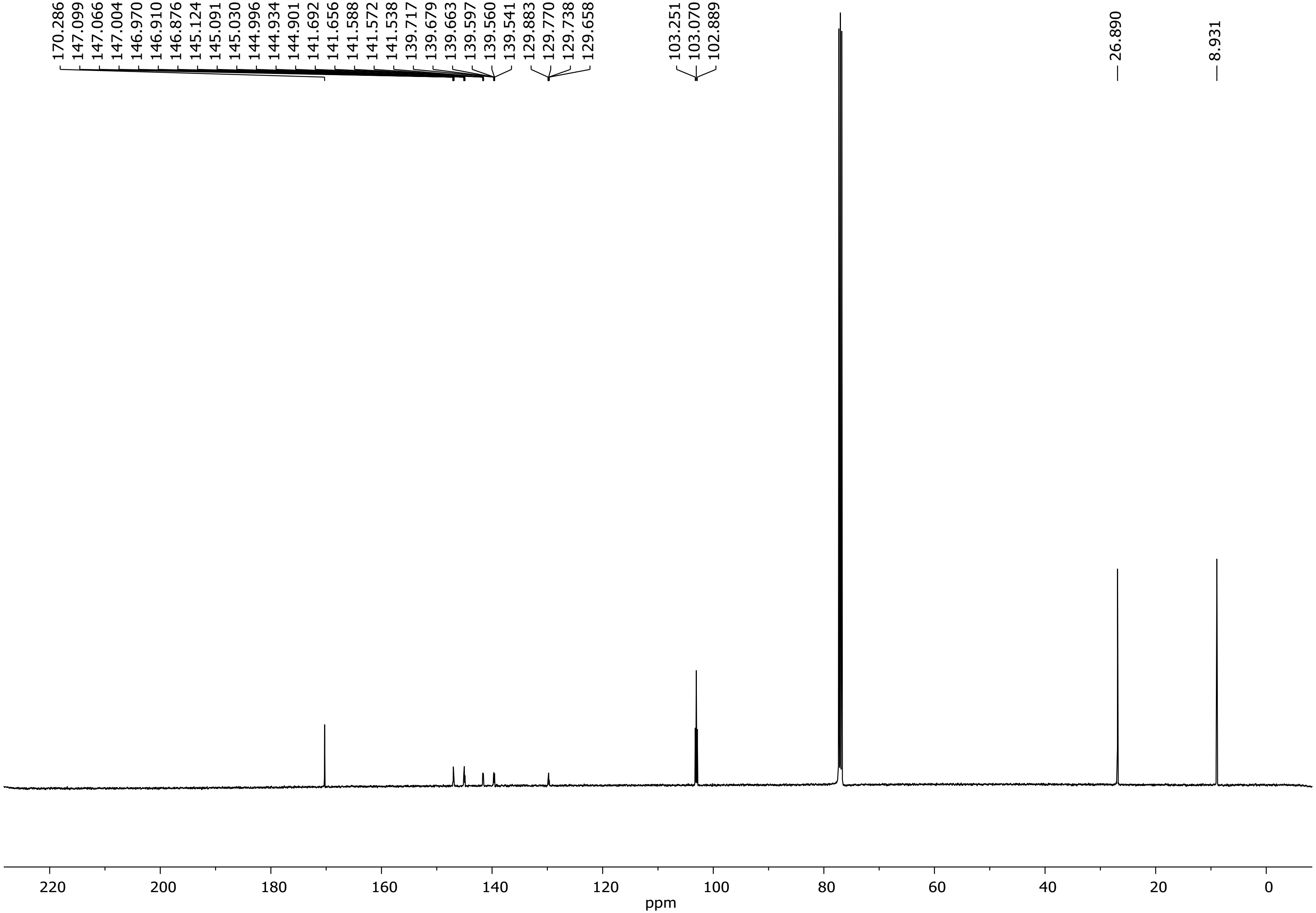

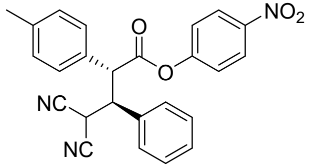

anti-**4**

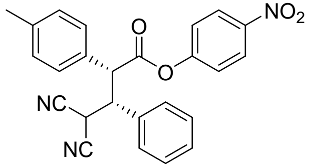

syn-**5**

^{1}H, $CDCl_3$, 400 MHz

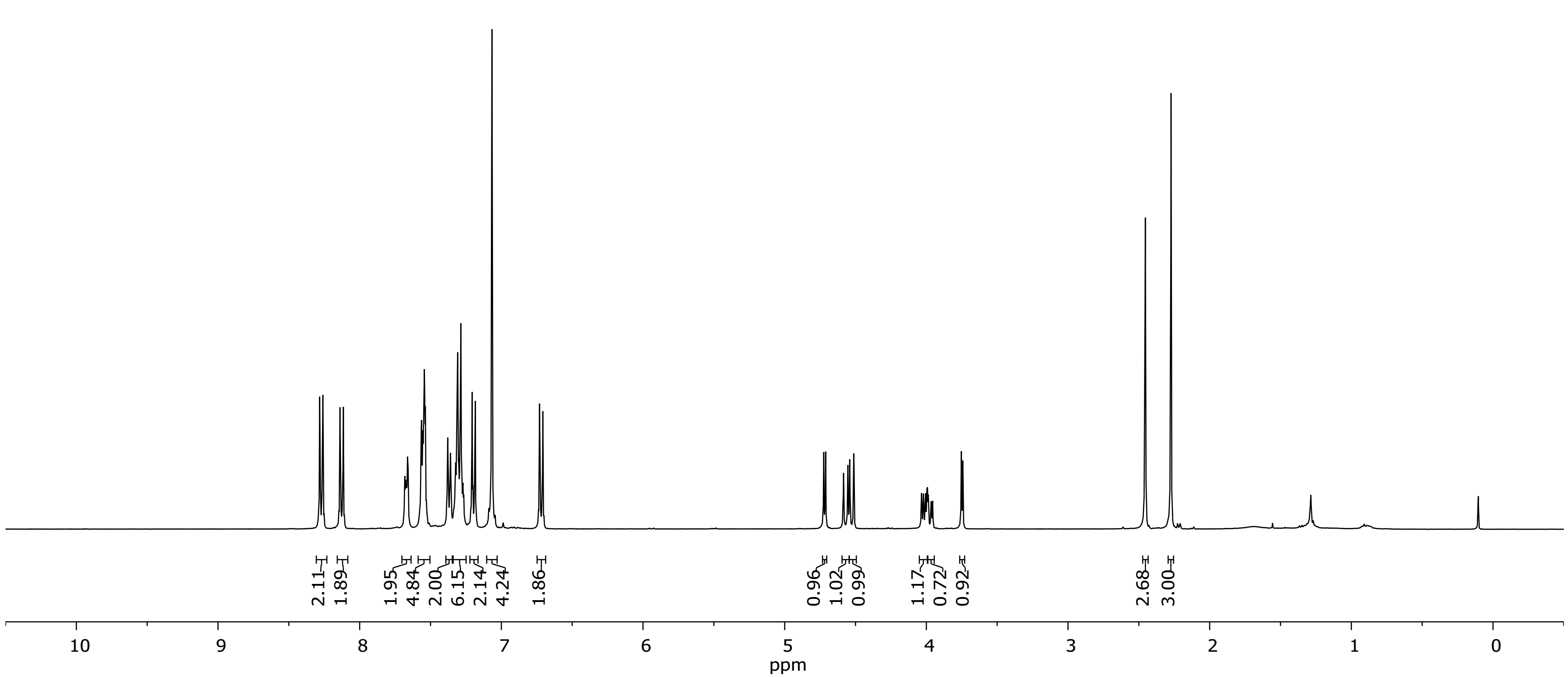

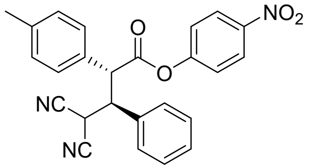

anti-**4**

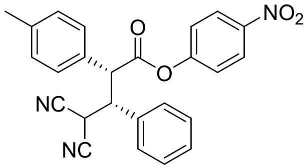

syn-**5**

$^{13}C\{^1H\}$, $CDCl_3$, 126 MHz

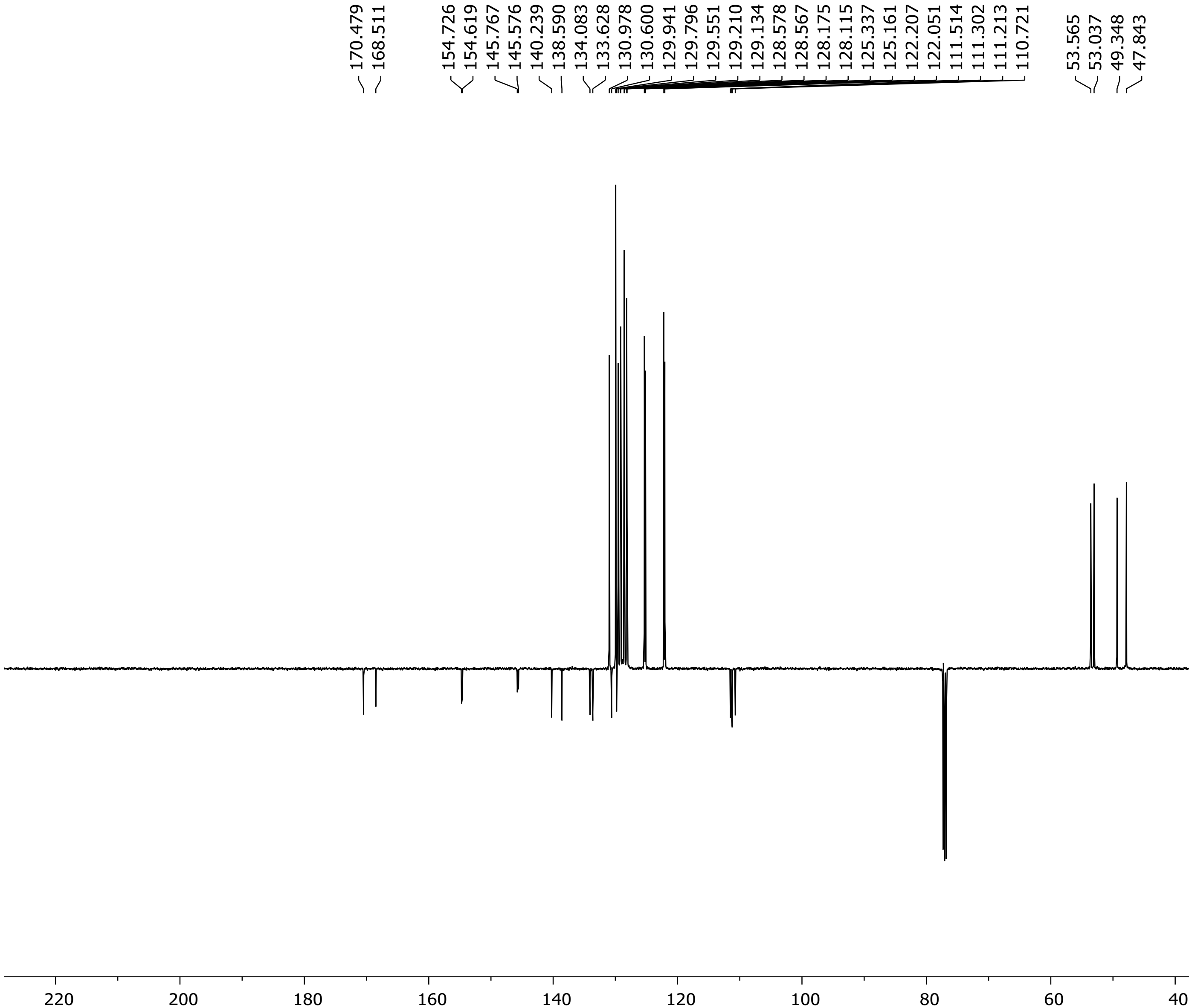

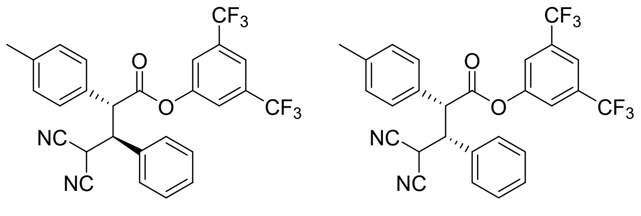

anti-**10** *syn*-**11**

^1H, $CDCl_3$, 500 MHz

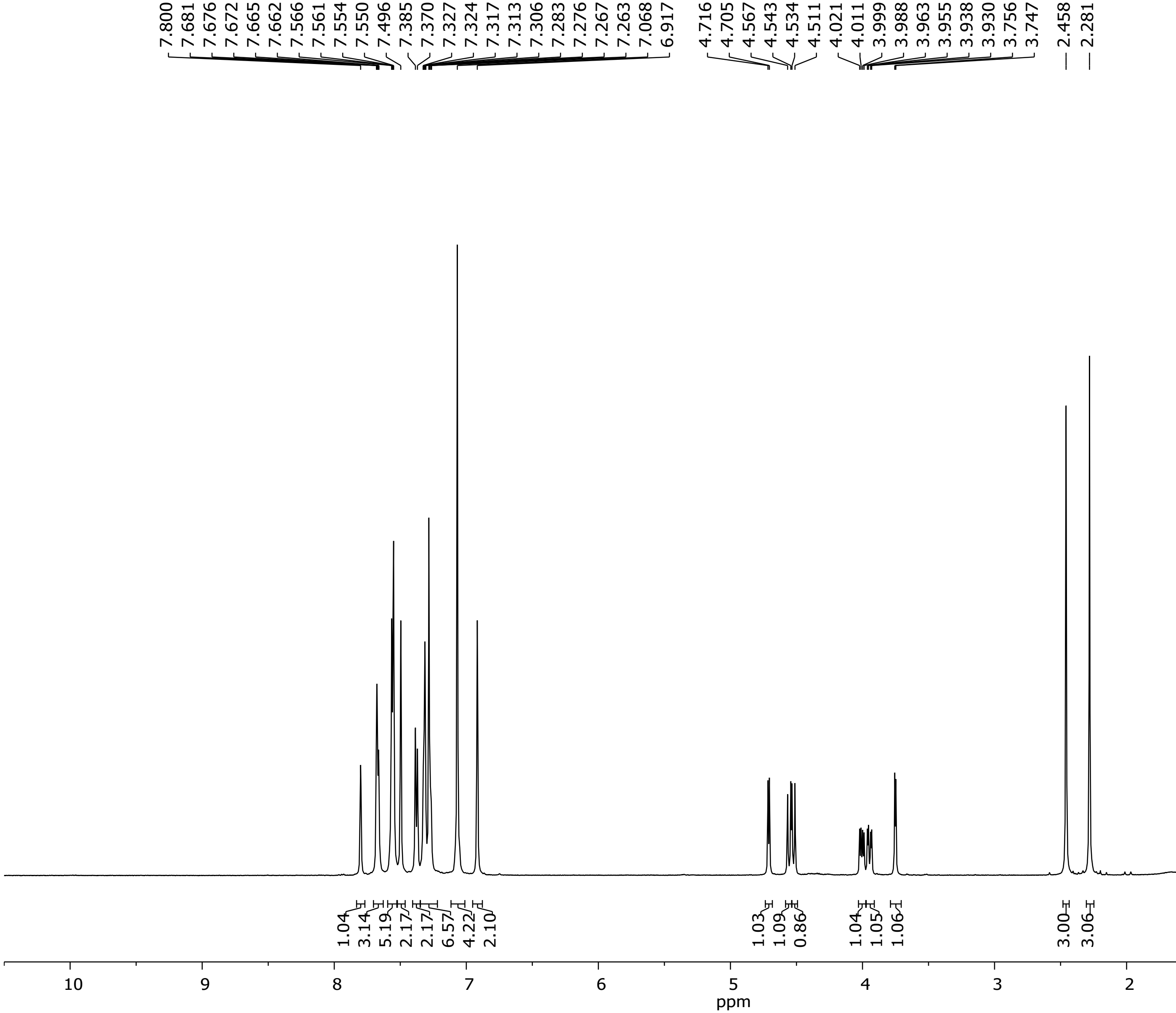

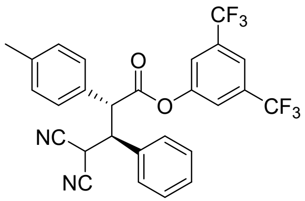

anti-**10**

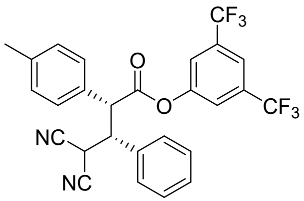

syn-**11**

$^{19}F\{^1H\}$, $CDCl_3$, 470 MHz

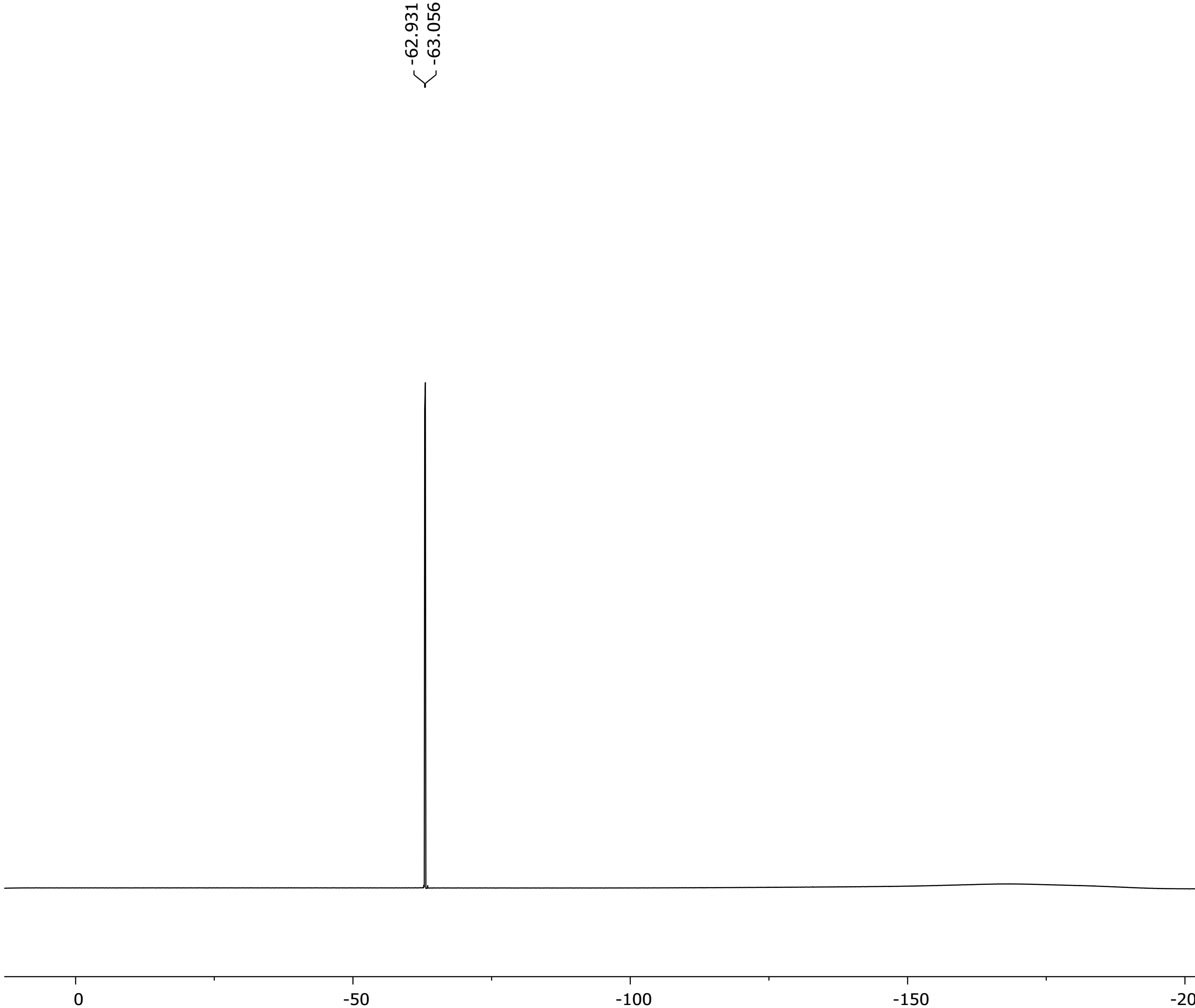

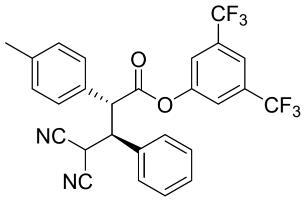

anti-**10**

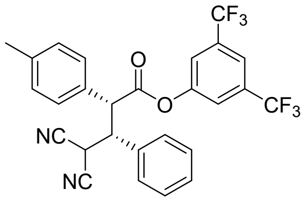

syn-**11**

$^{13}C\{^{1}H\}$, $CDCl_3$, 126 MHz

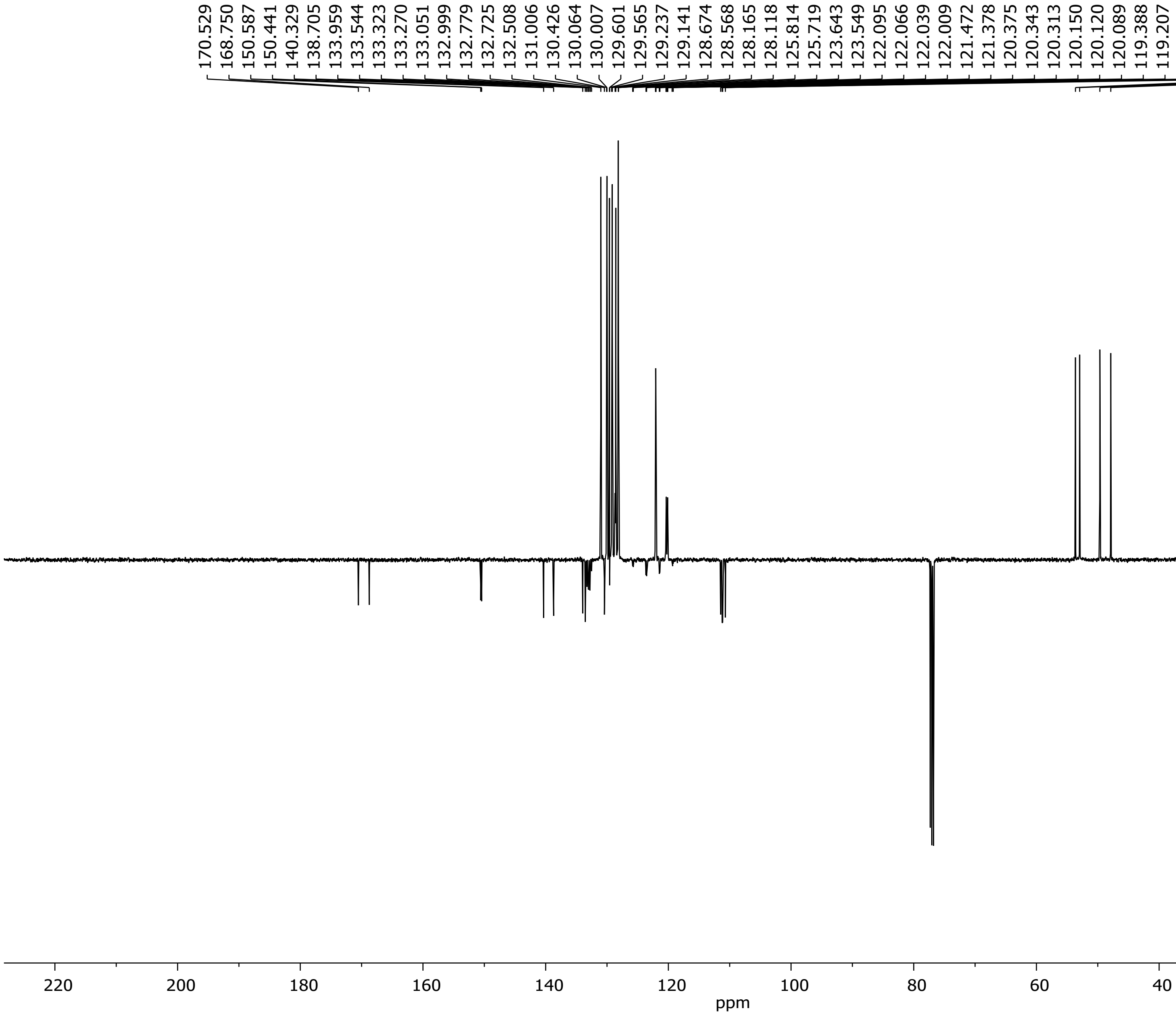

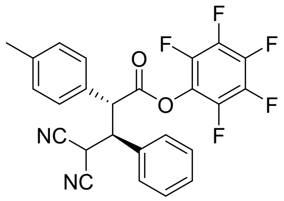

anti-**12**

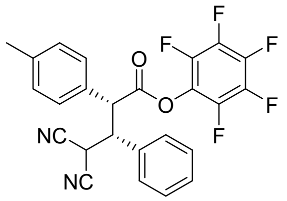

syn-**13**

^{1}H, $CDCl_3$, 500 MHz

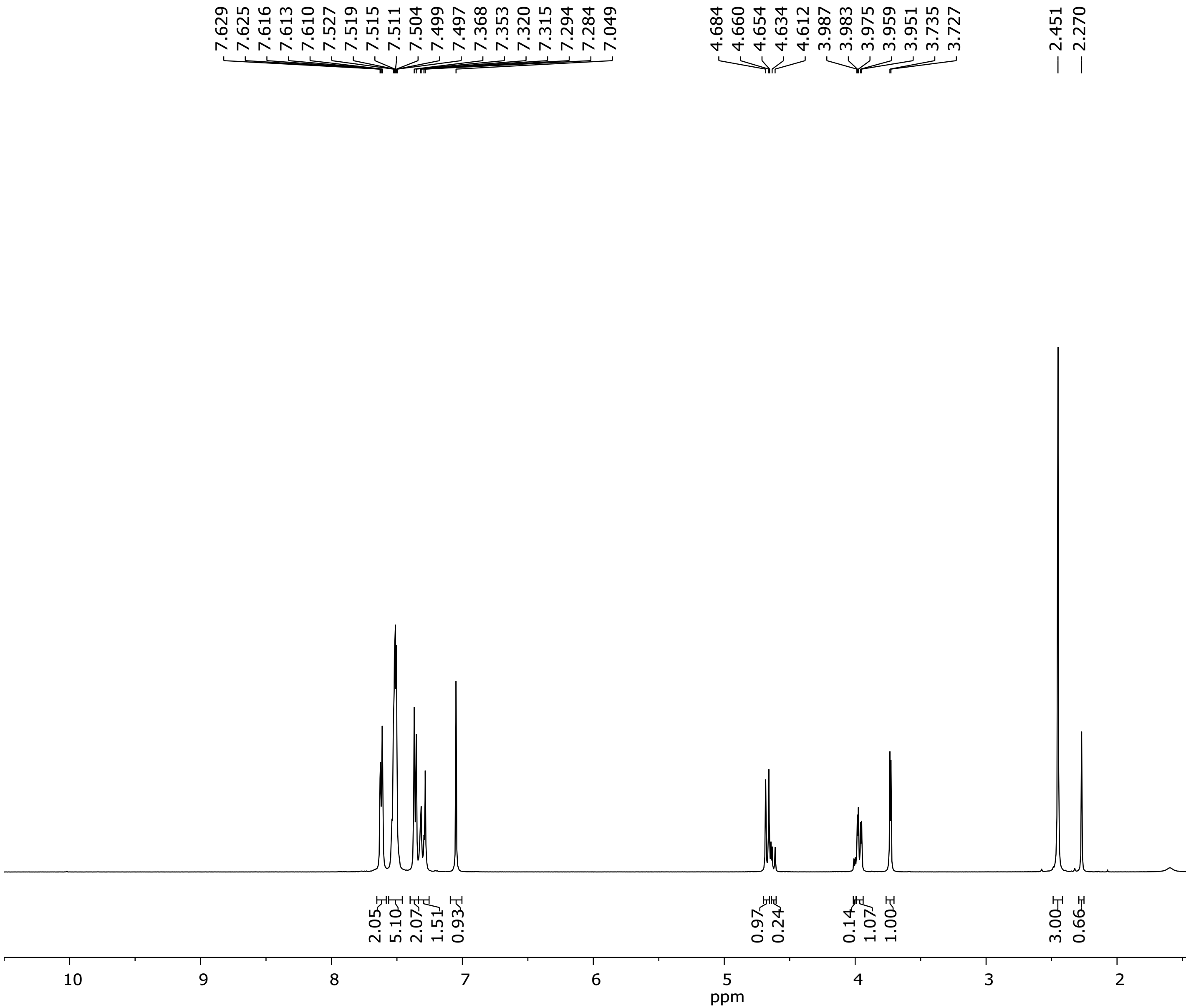

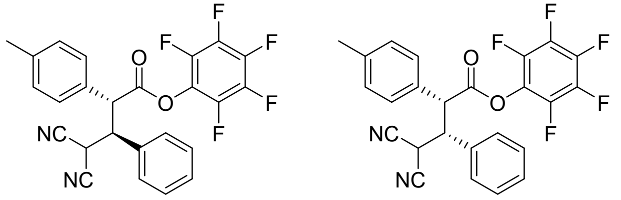

anti-**12**

syn-**13**

$^{19}F\{^{1}H\}$, $CDCl_3$, 470 MHz

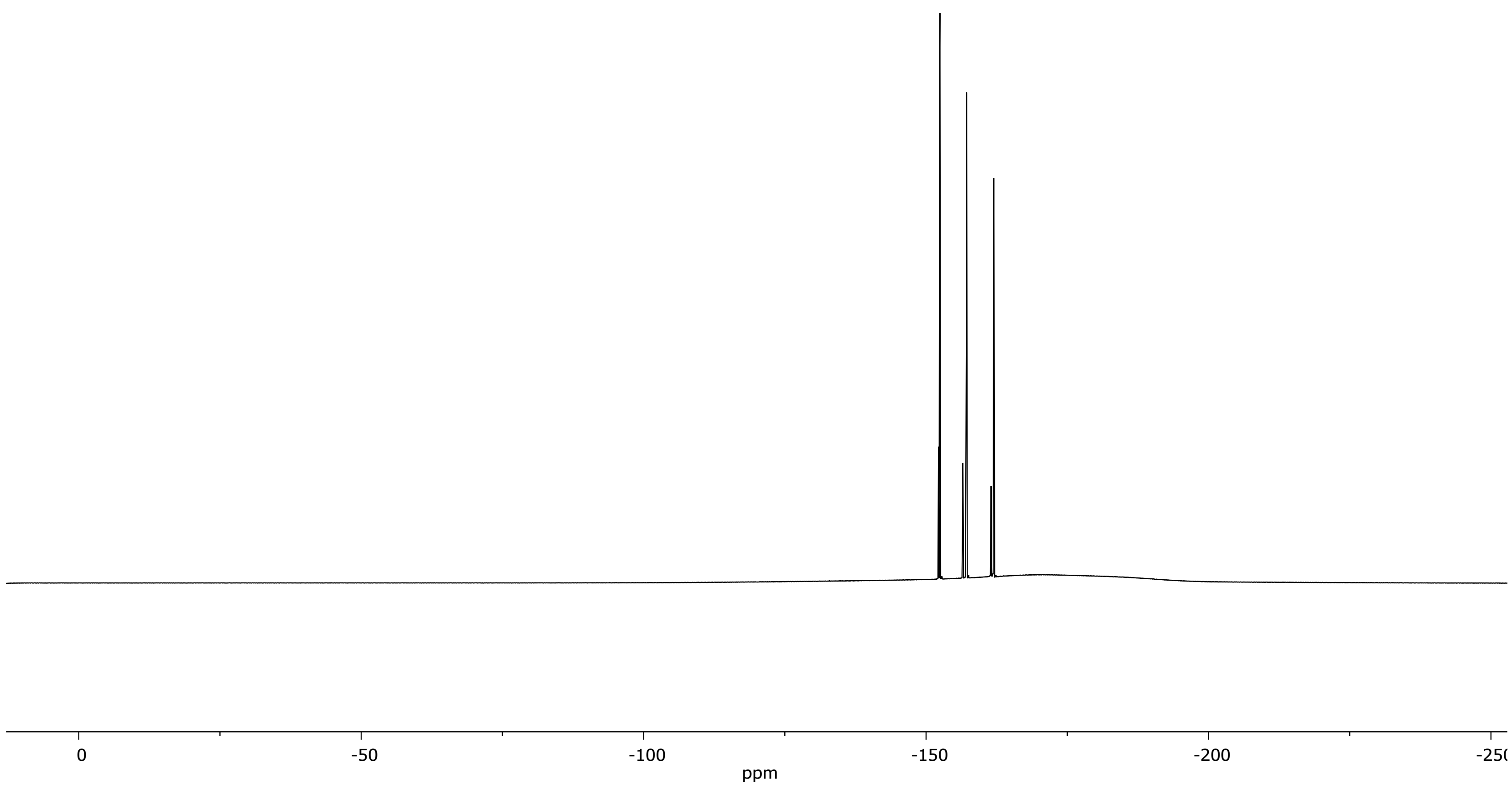

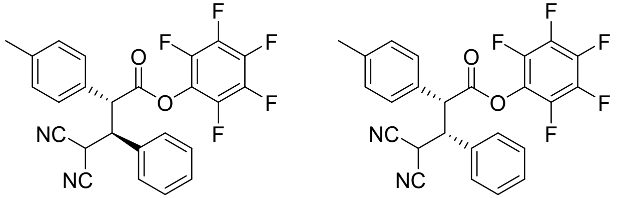

anti-**12**　　*syn*-**13**

$^{13}C\{^1H\}$, $CDCl_3$, 126 MHz

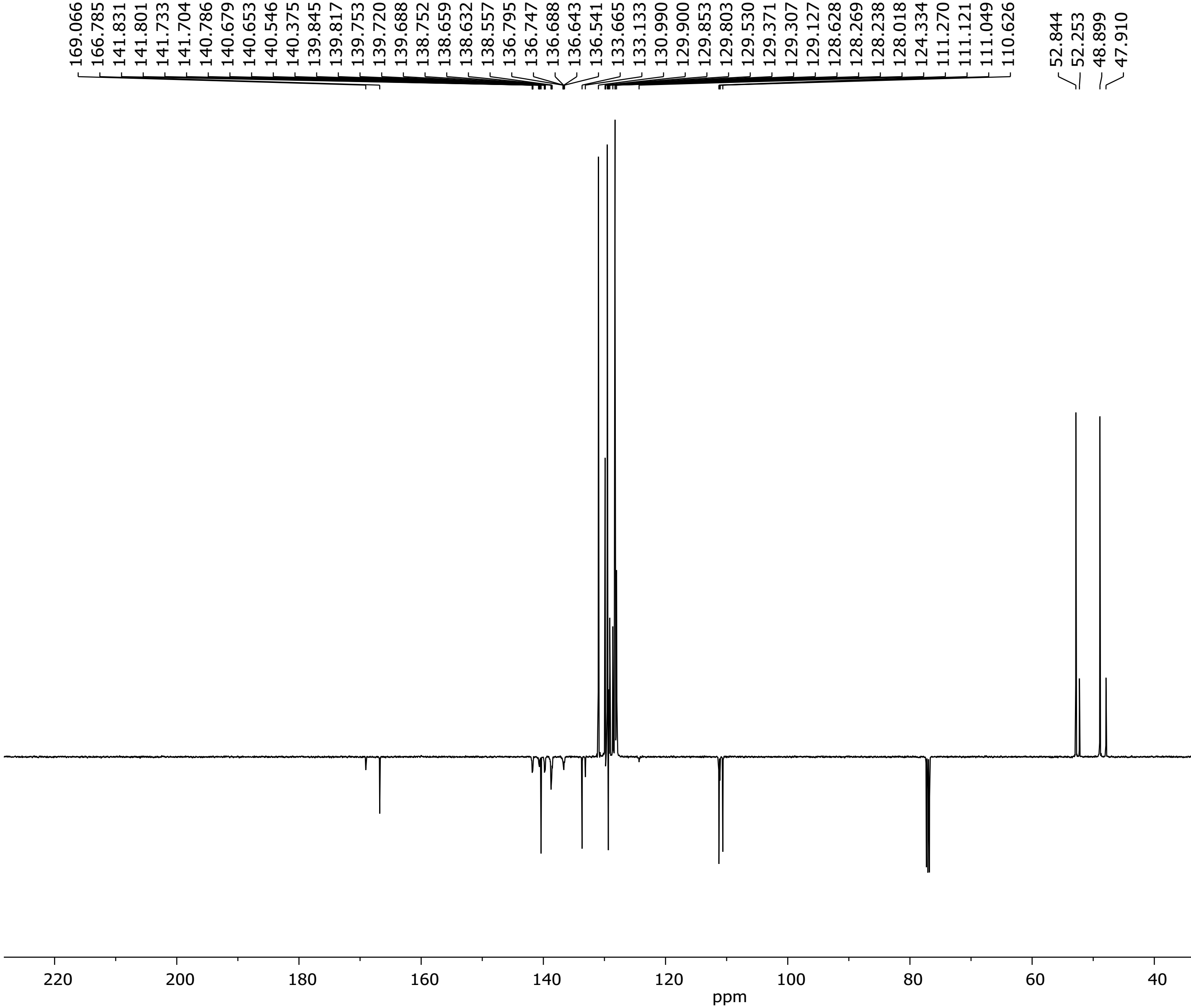

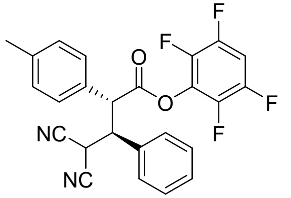

anti-**14**
^{1}H, $CDCl_3$, 400 MHz

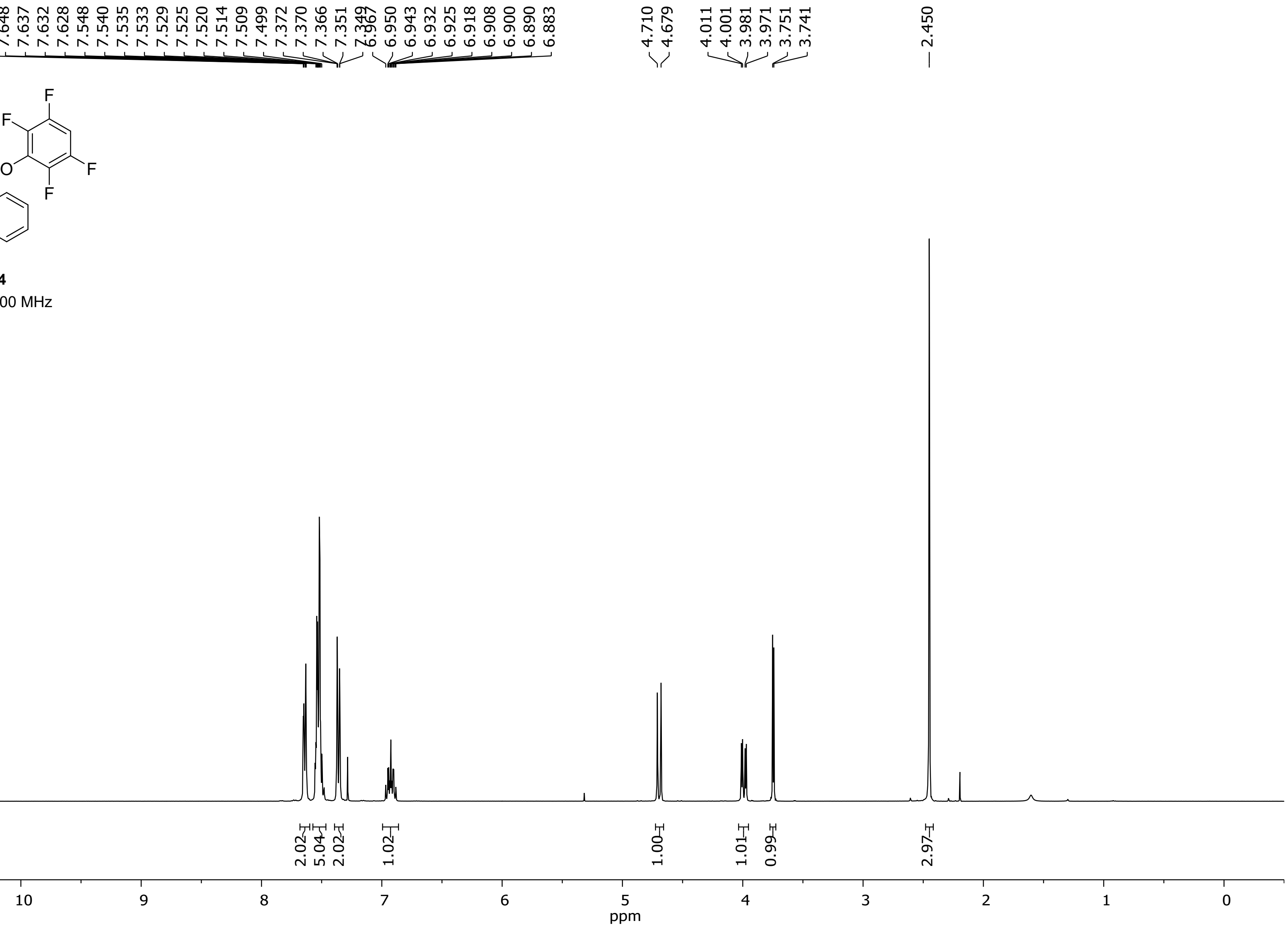

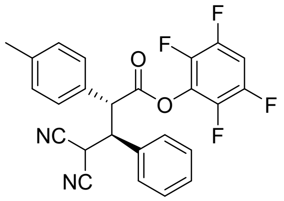

anti-**14**
$^{19}F\{^{1}H\}$, $CDCl_3$, 376 MHz

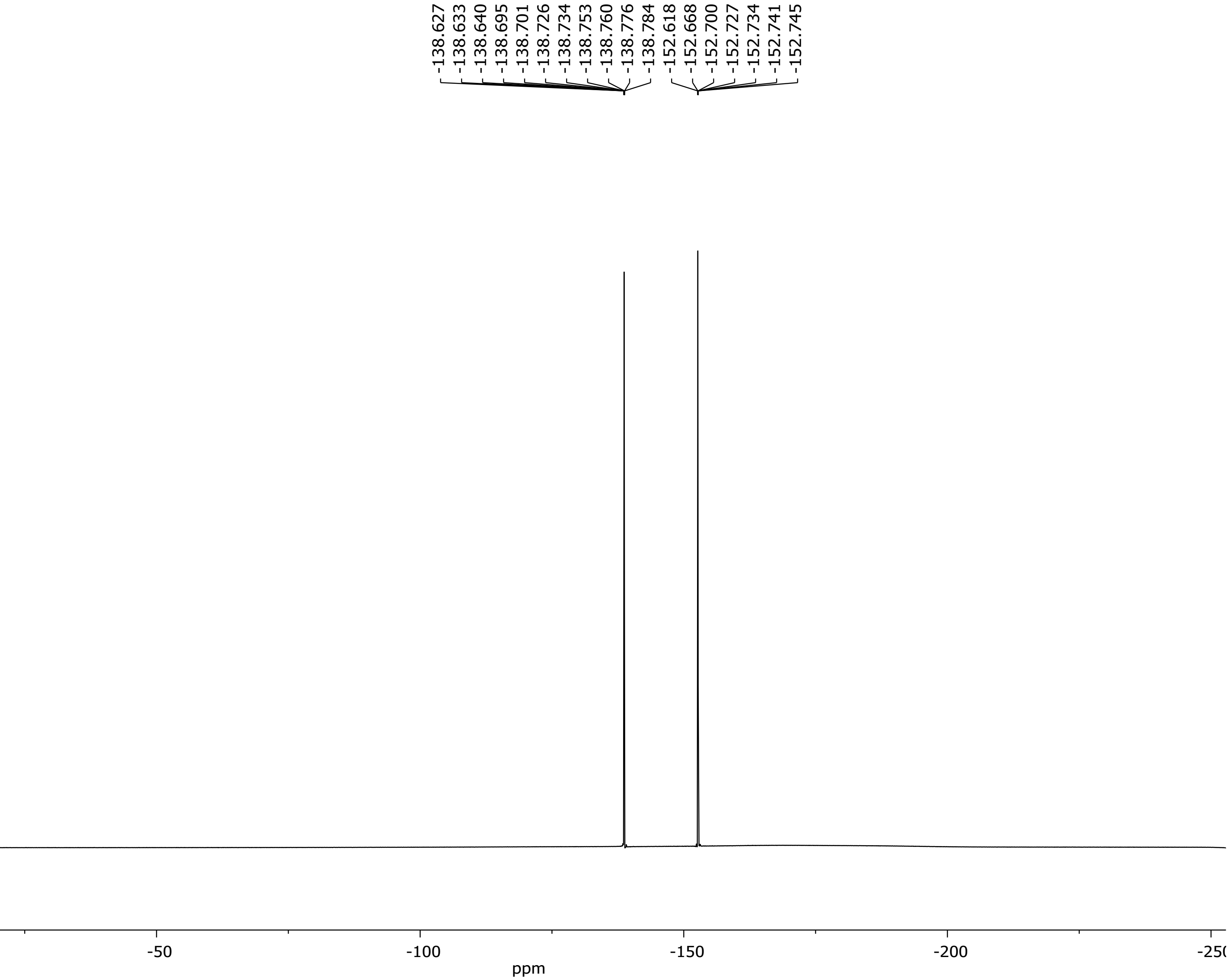

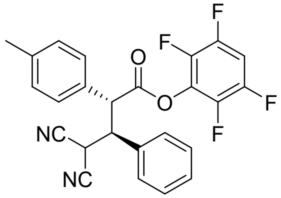

anti-**14**

$^{13}C\{^{1}H\}$, $CDCl_3$, 126 MHz

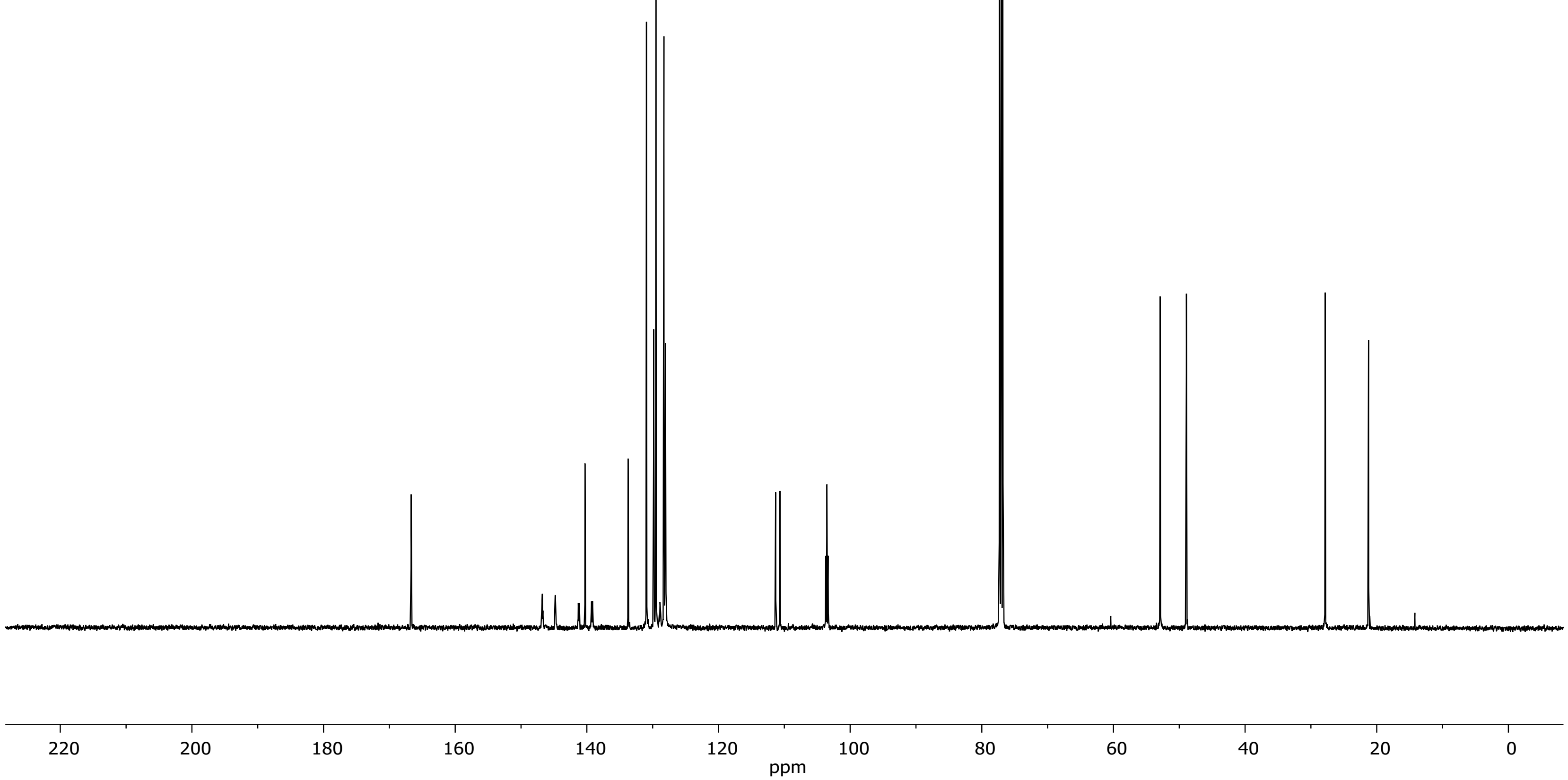

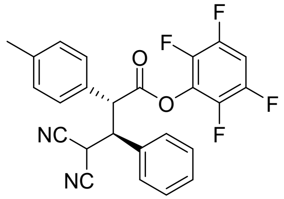

anti-**14**
2D ^1H-^1H COSY, $CDCl_3$

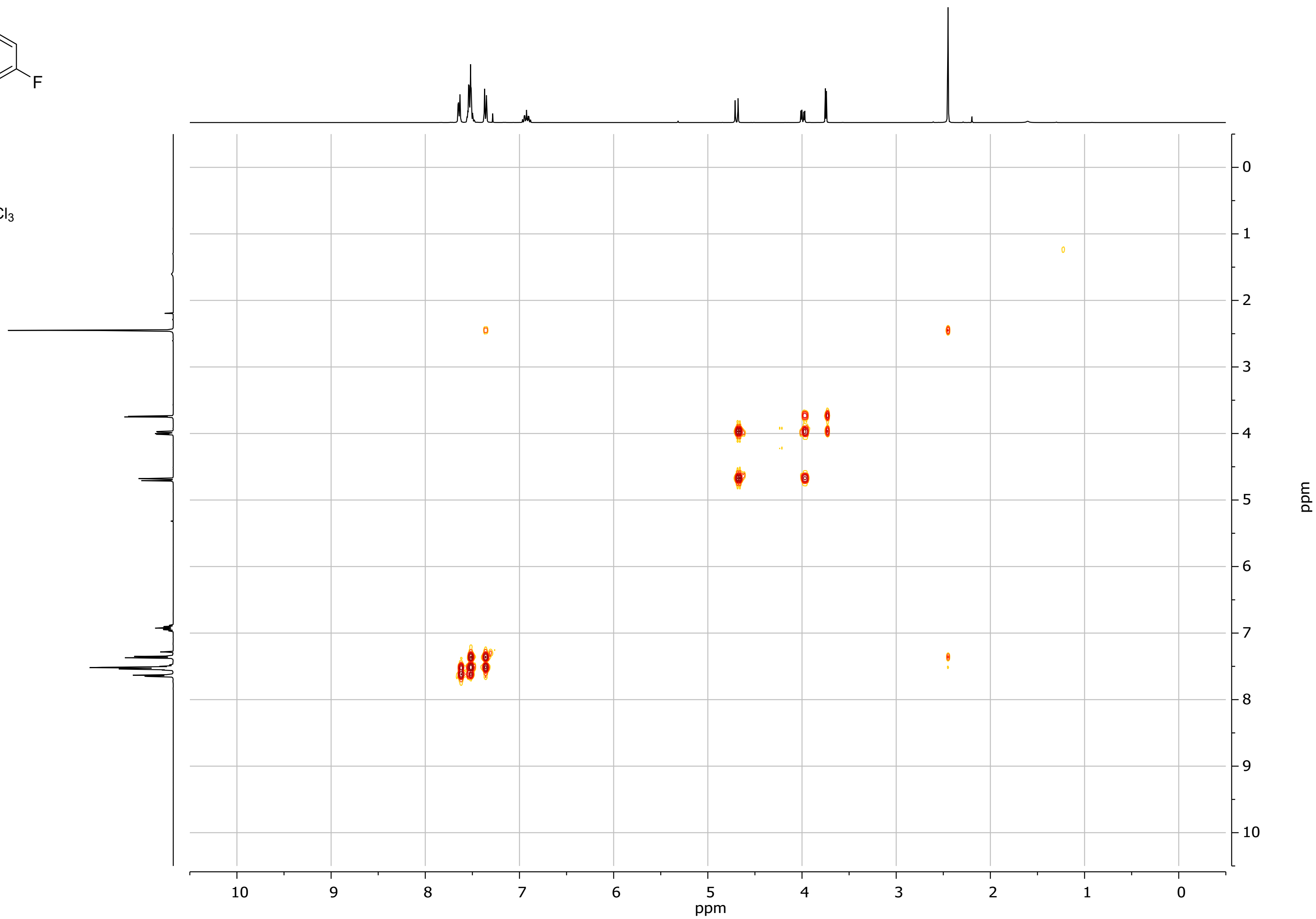

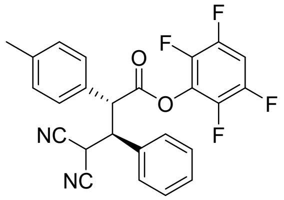

anti-**14**

2D ^{1}H-^{13}C HSQC, $CDCl_3$

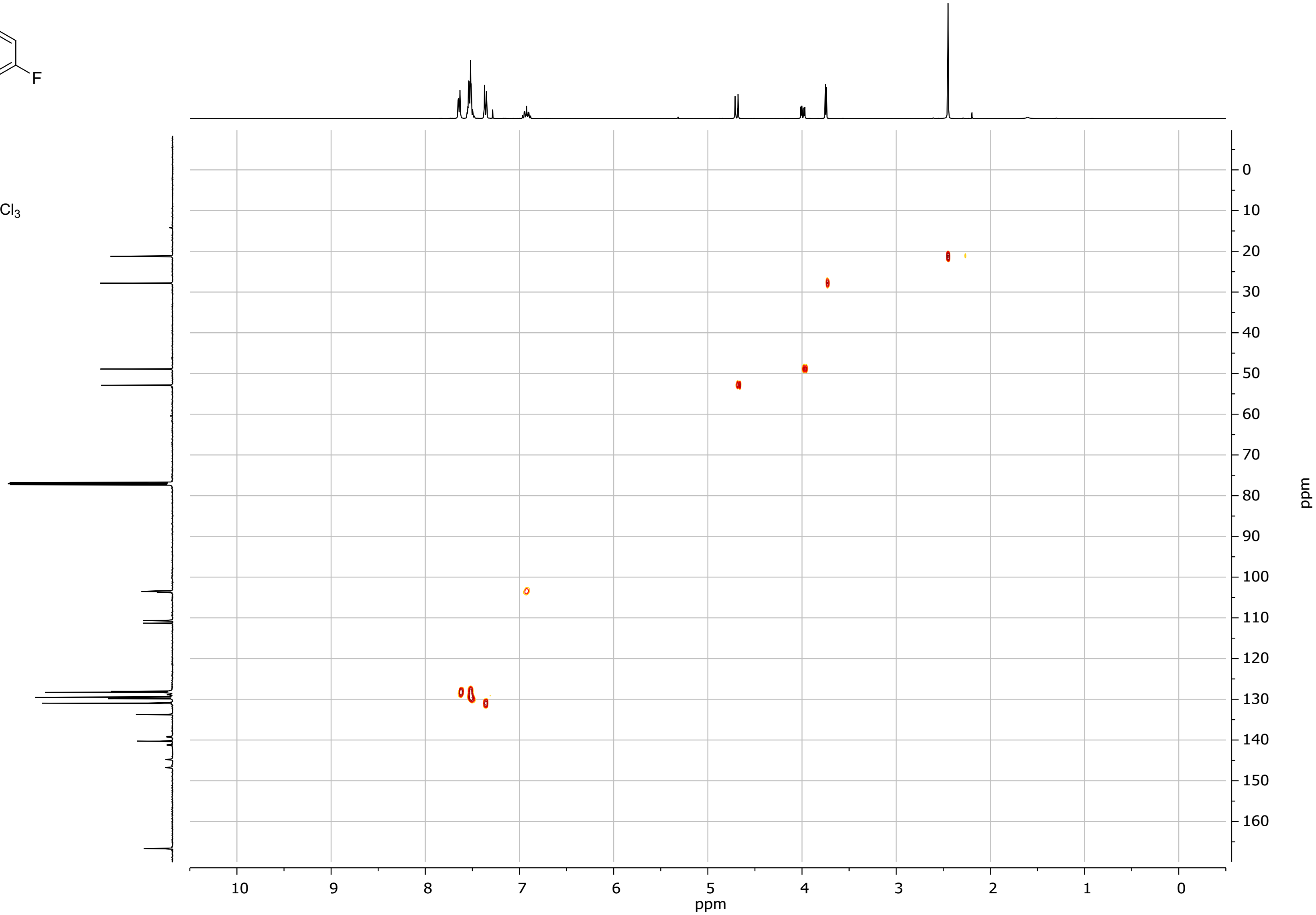

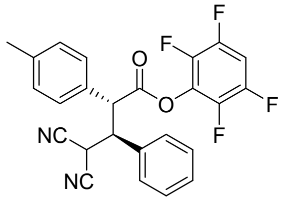

anti-**14**
2D ^{1}H-^{13}C HMBC, $CDCl_3$

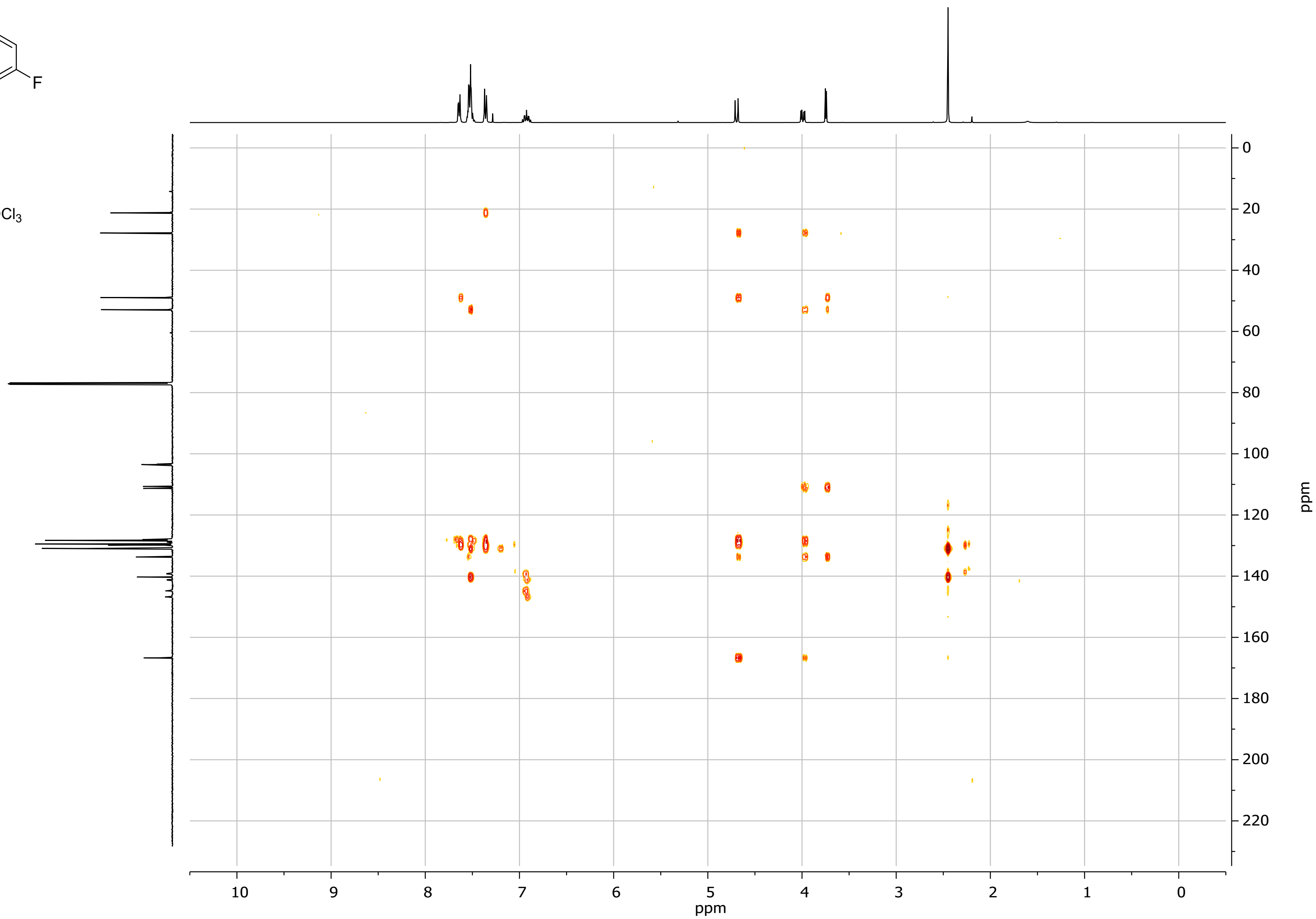

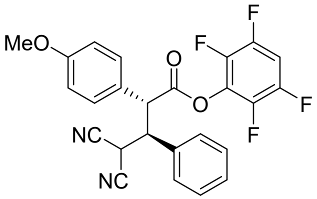

anti-**20**
^{1}H, $CDCl_3$, 400 MHz

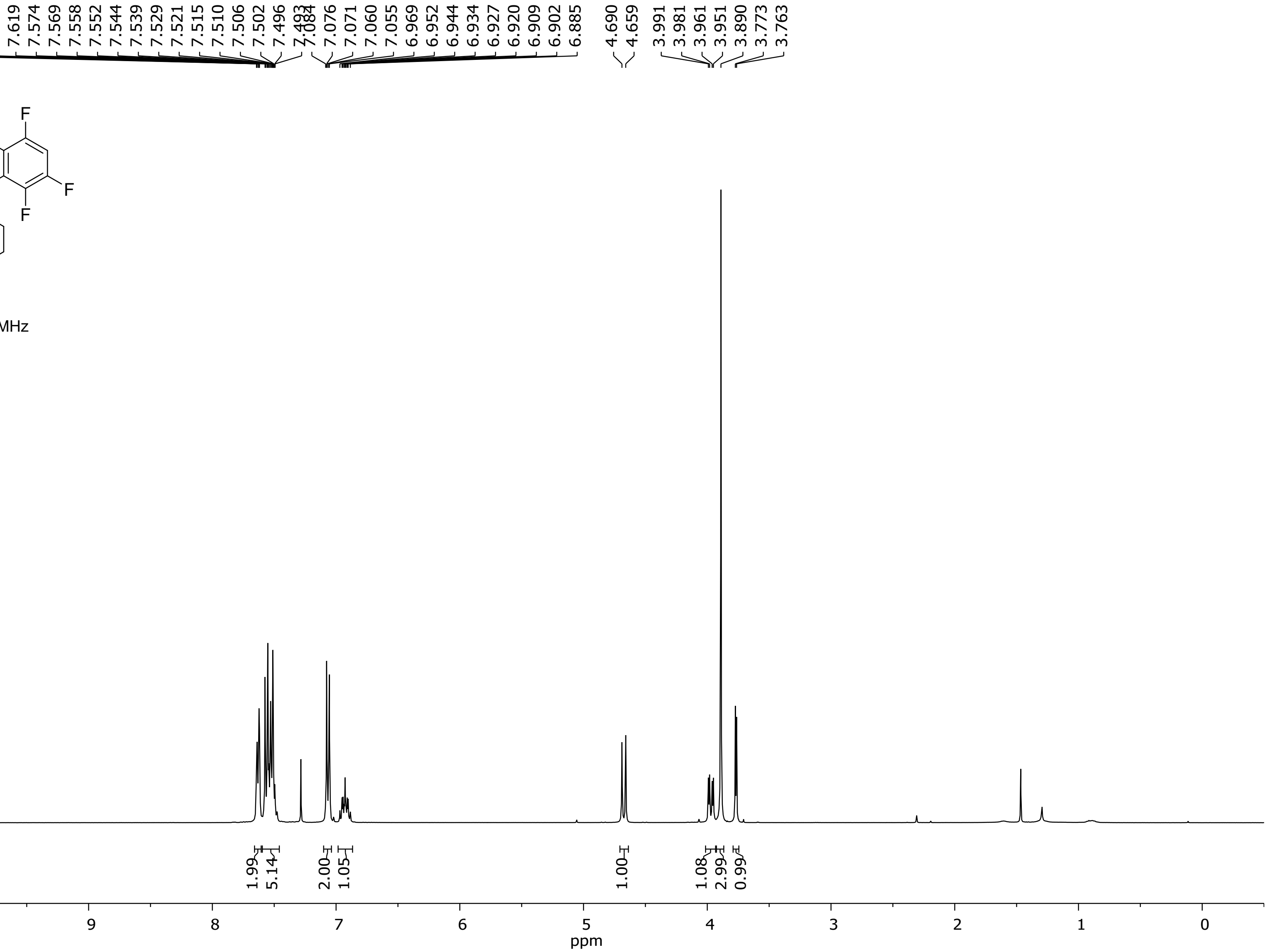

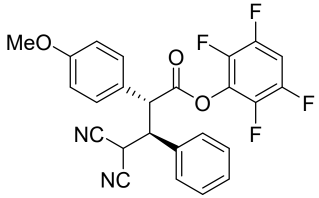

anti-**20**

$^{19}F\{^{1}H\}$, $CDCl_3$, 376 MHz

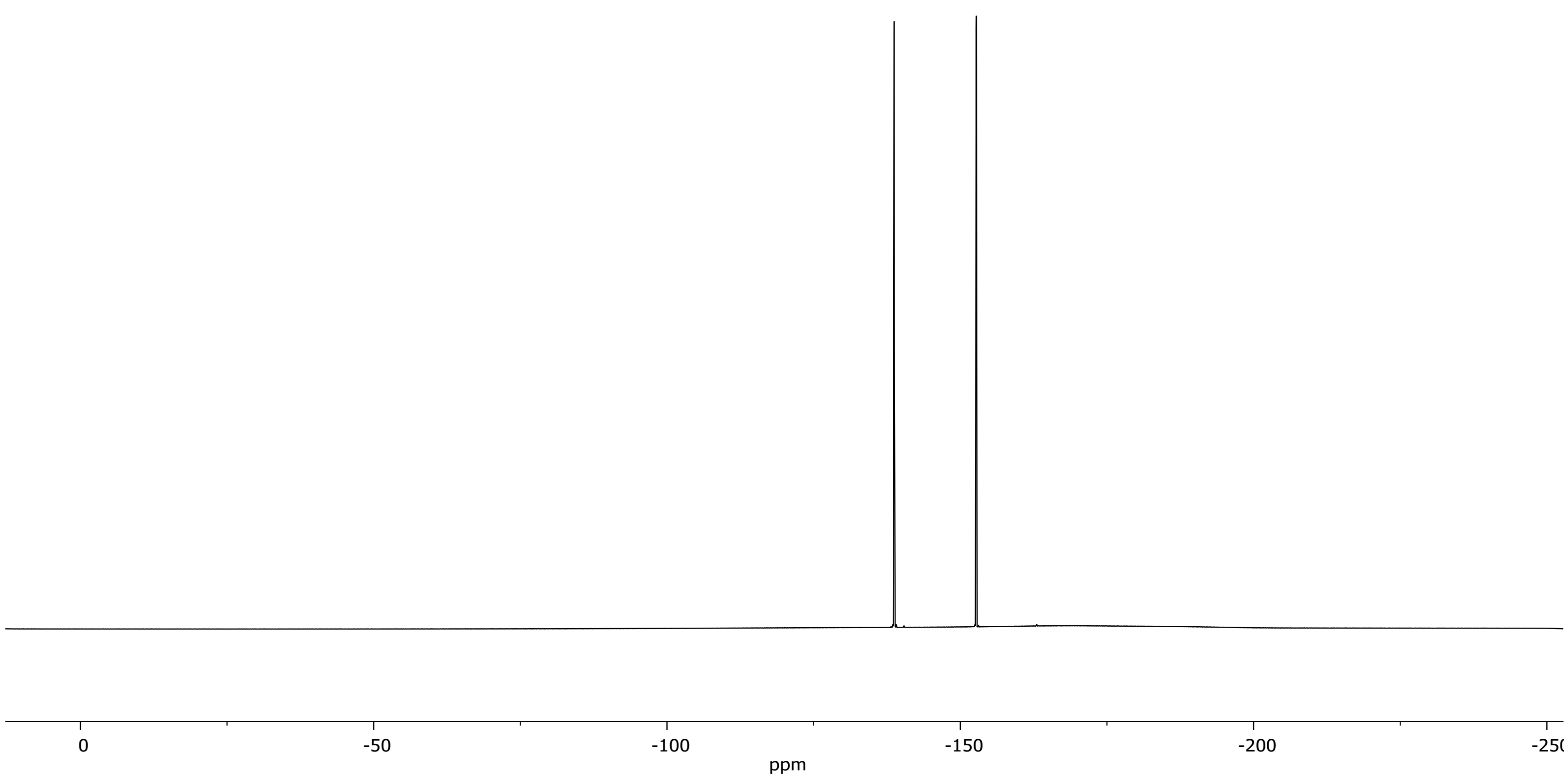

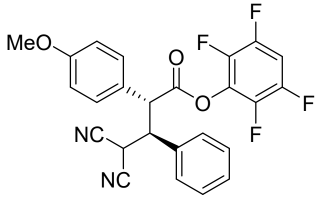

anti-**20**

$^{13}C\{^{1}H\}$, $CDCl_3$, 126 MHz

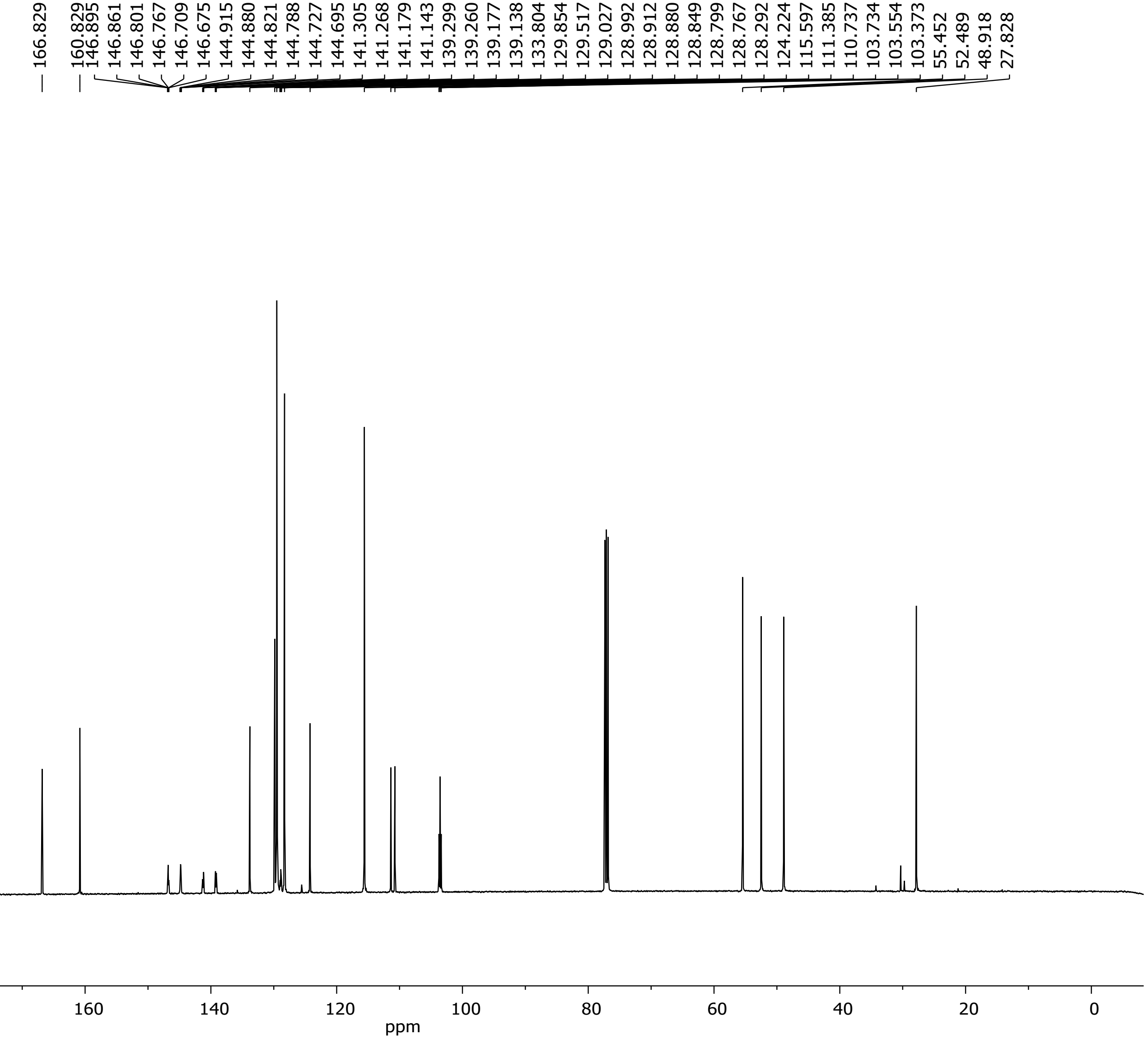

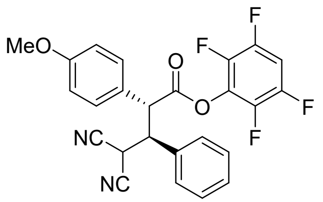

anti-**20**

2D ^{1}H-^{1}H COSY, $CDCl_3$

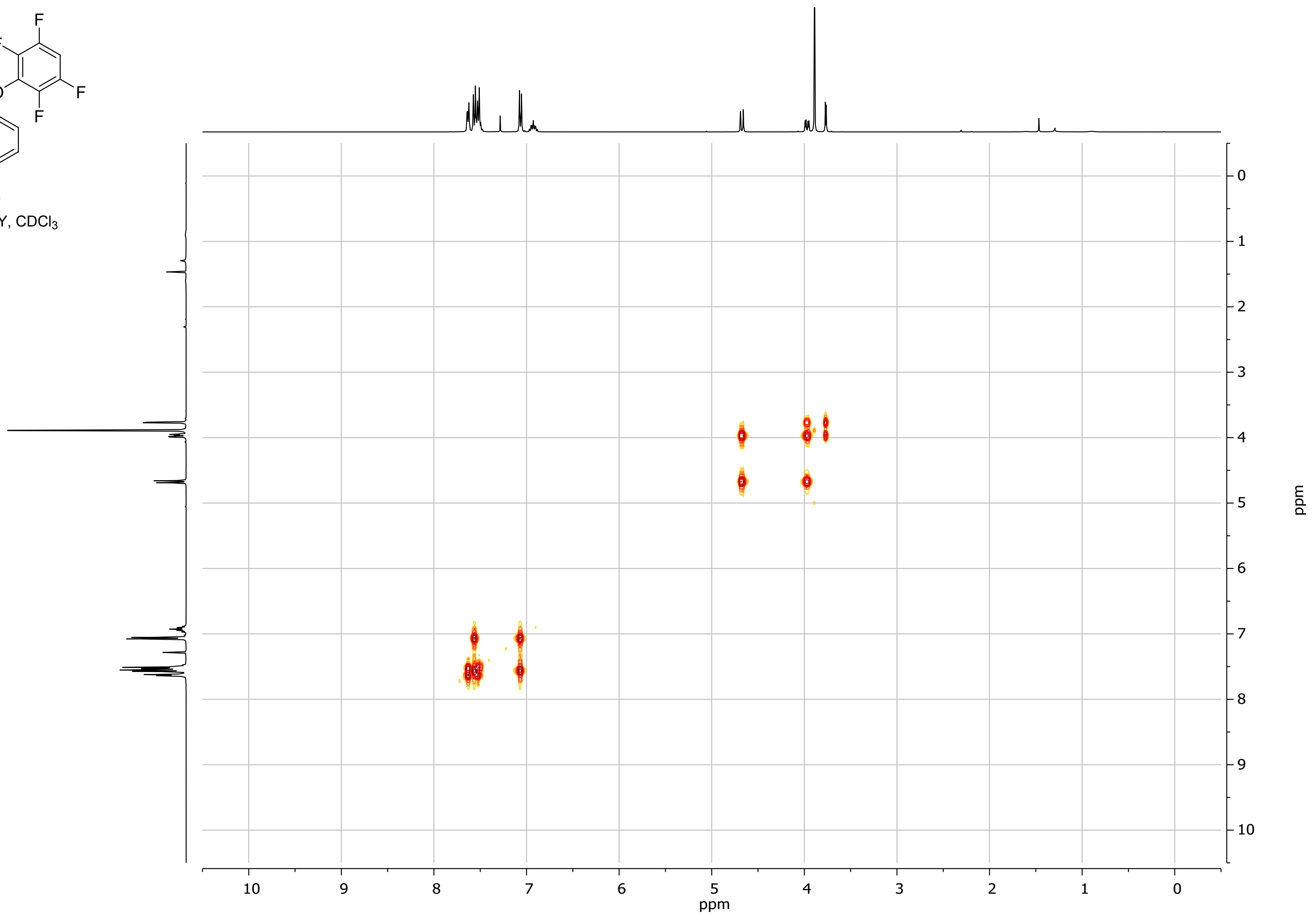

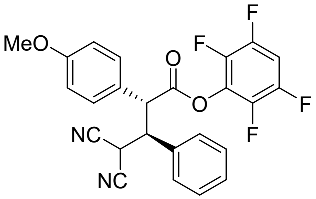

anti-**20**
2D ^{1}H-^{13}C HSQC, $CDCl_3$

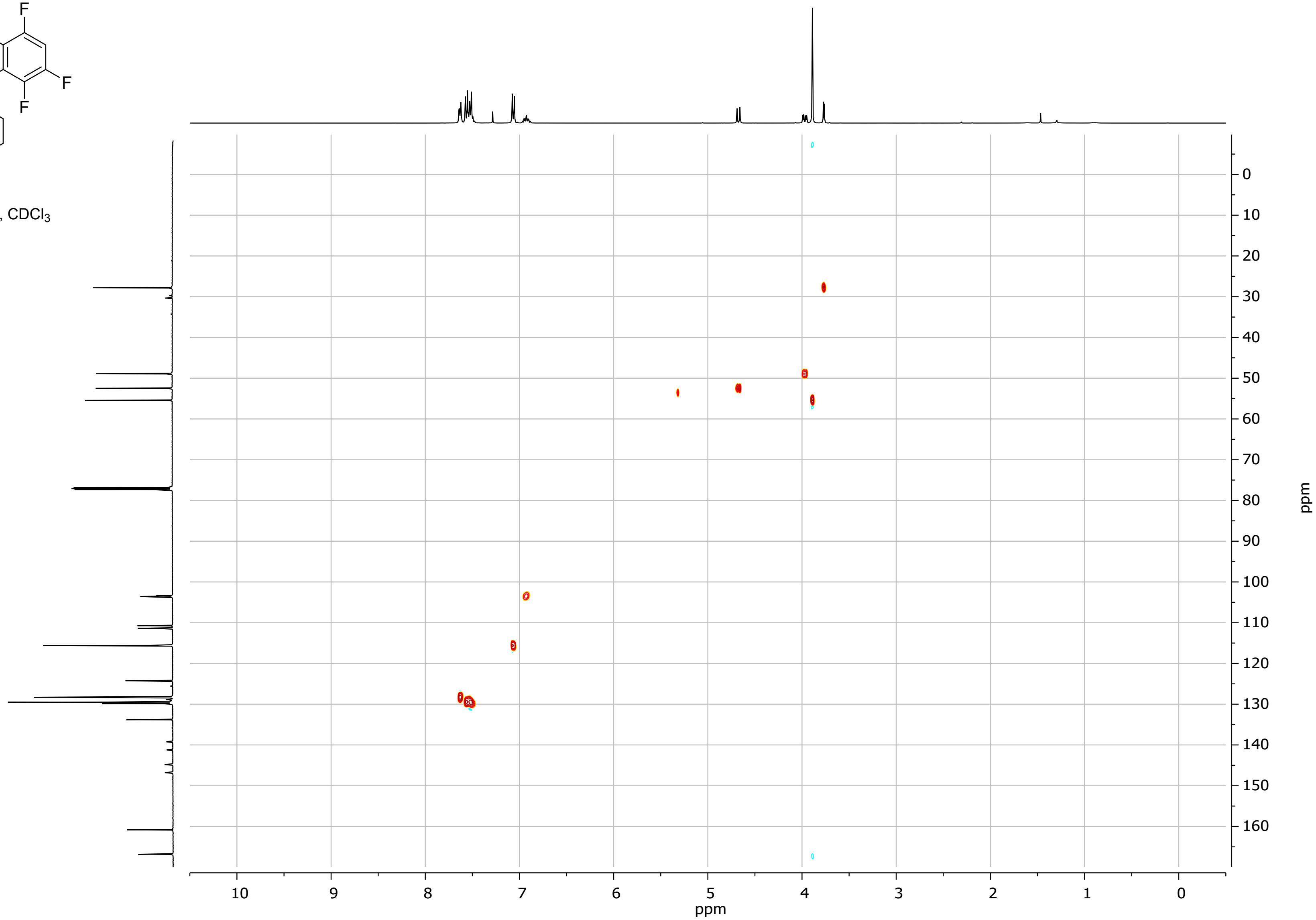

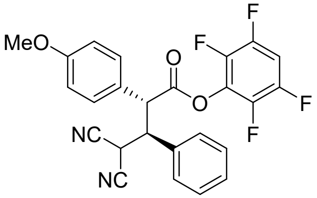

anti-**20**

2D ^{1}H-^{13}C HMBC, $CDCl_3$

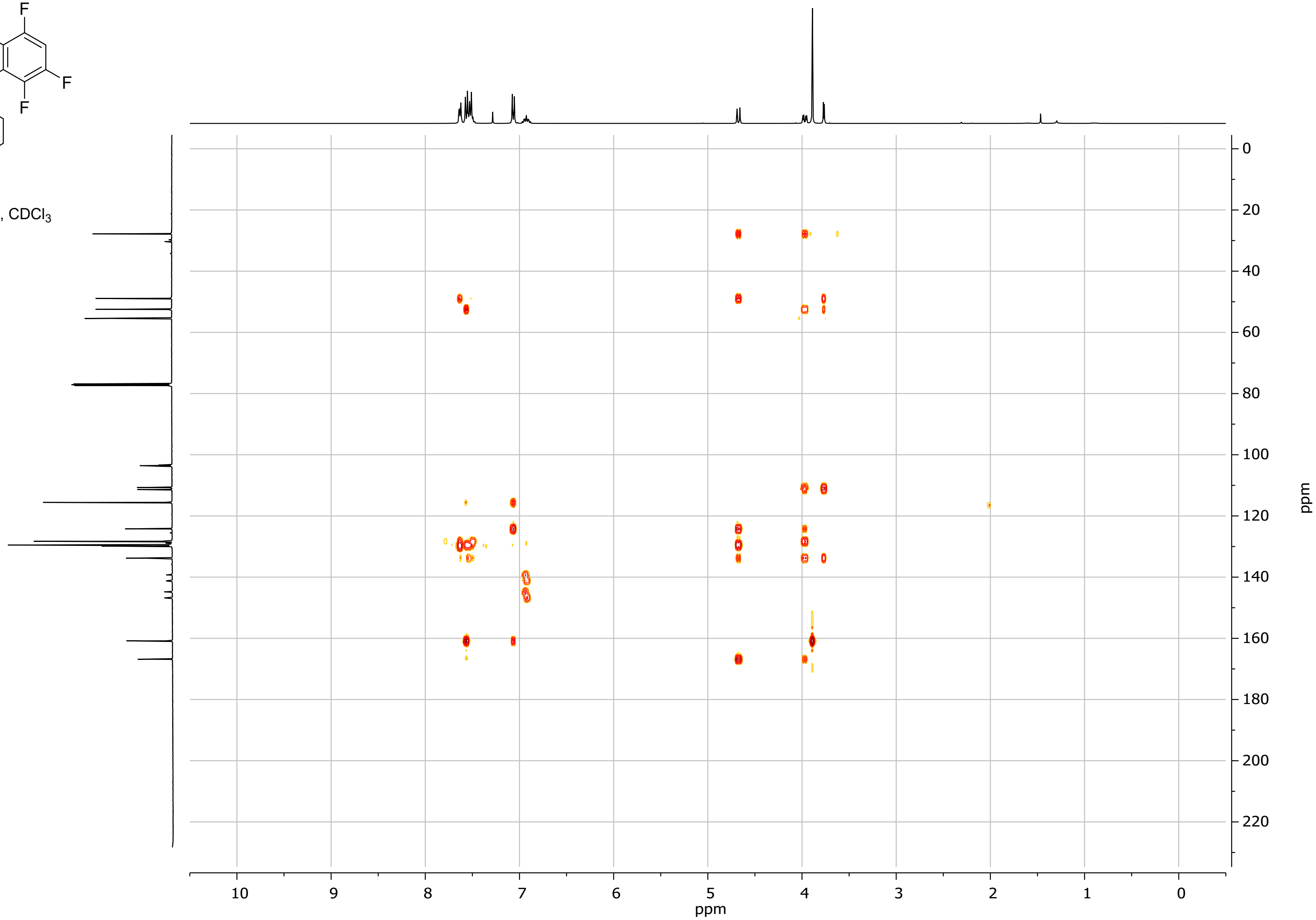

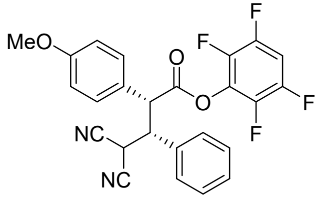

syn-**S17**
^{1}H, $CDCl_3$, 400 MHz

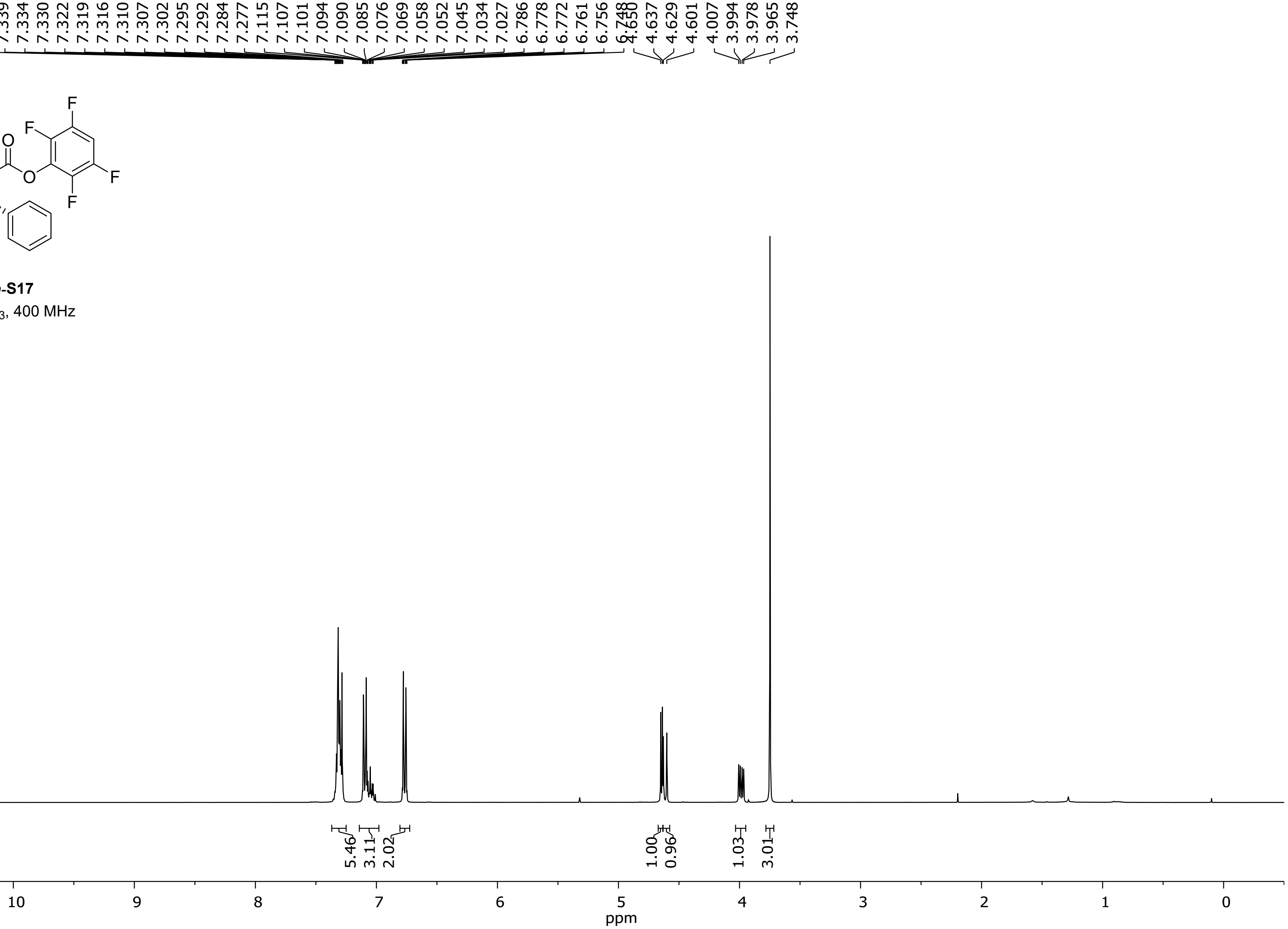

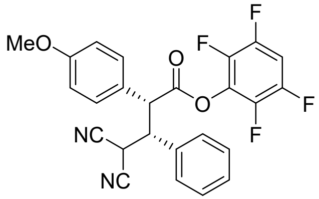

syn-**S17**

$^{19}F\{^{1}H\}$, $CDCl_3$, 376 MHz

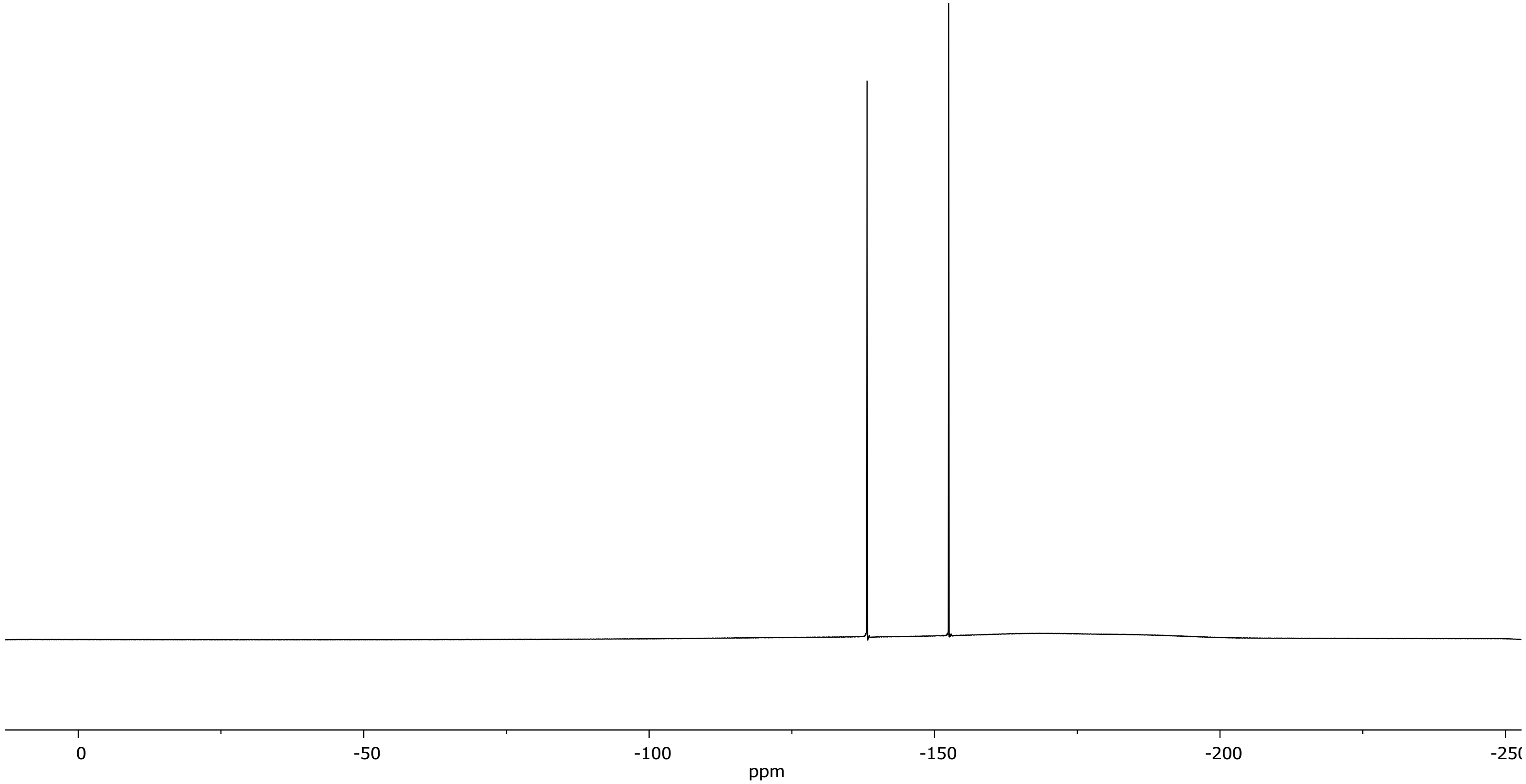

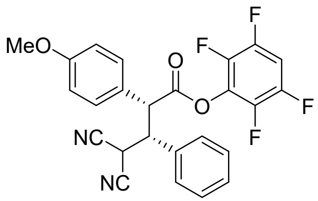

syn-**S17**

$^{13}C\{^1H\}$, $CDCl_3$, 126 MHz

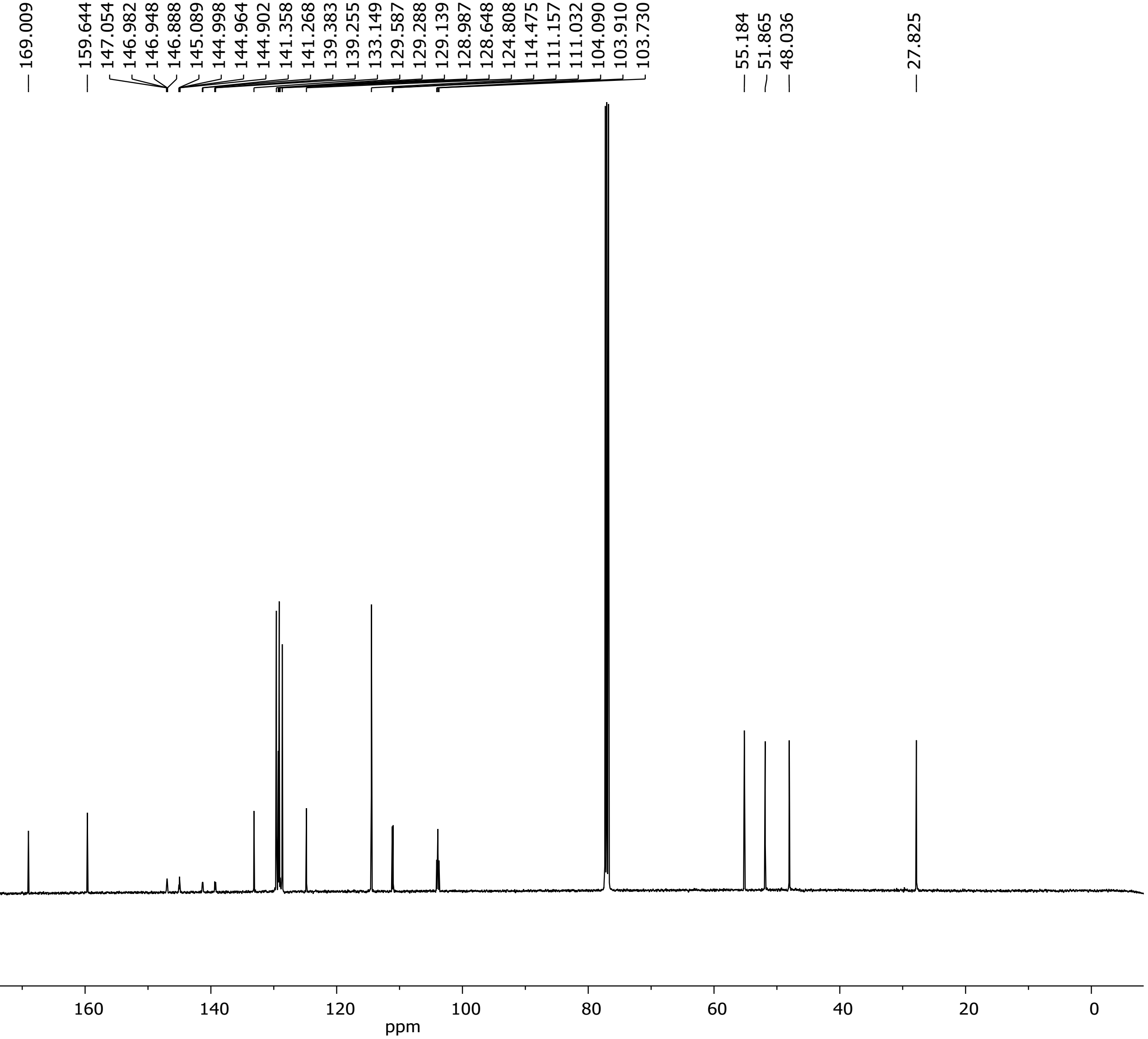

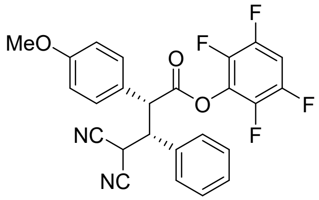

syn-**S17**
2D ^{1}H-^{1}H COSY, $CDCl_3$

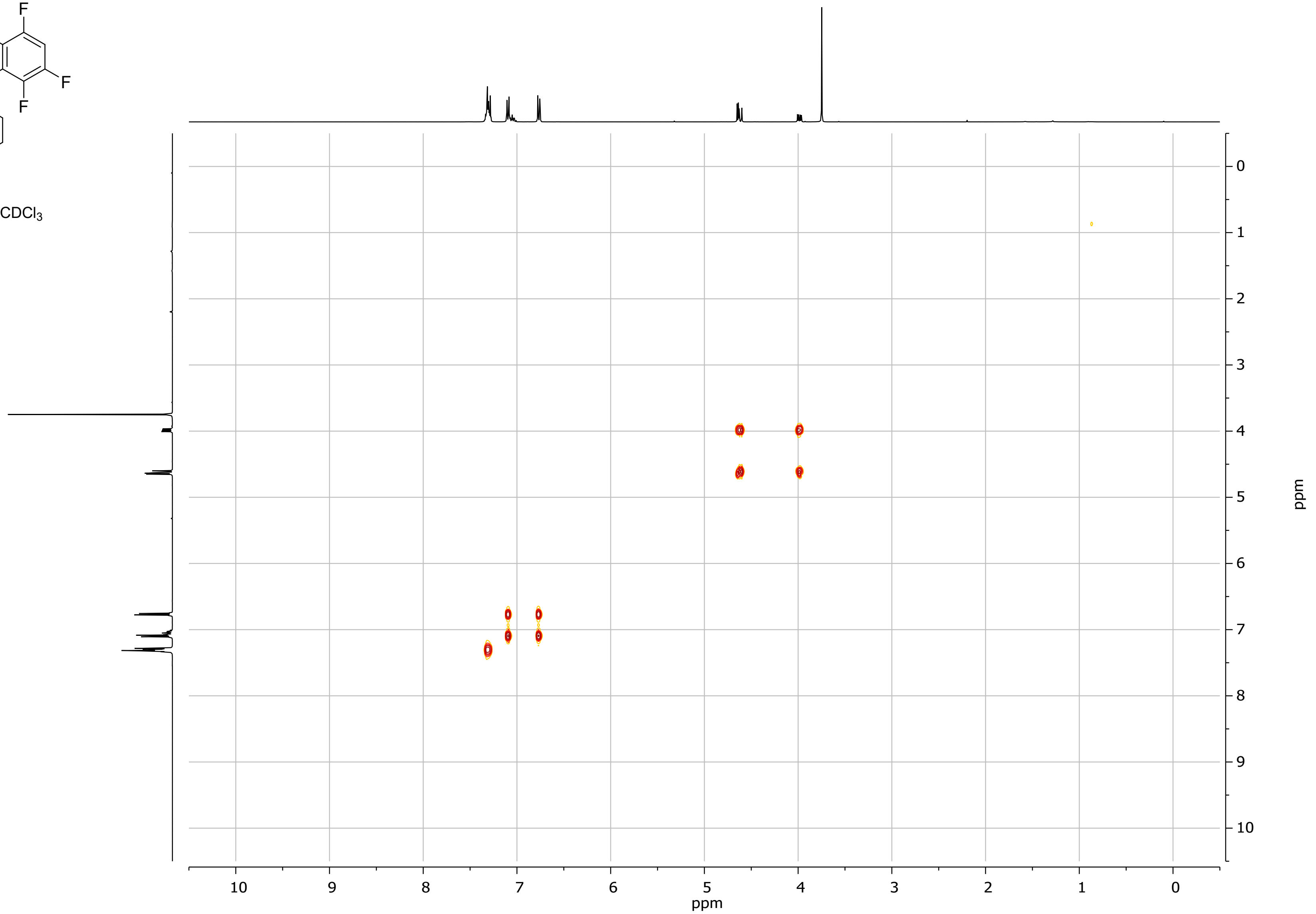

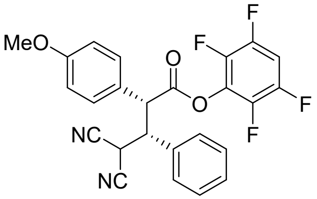

syn-**S17**

2D ^{1}H-^{13}C HSQC, $CDCl_3$

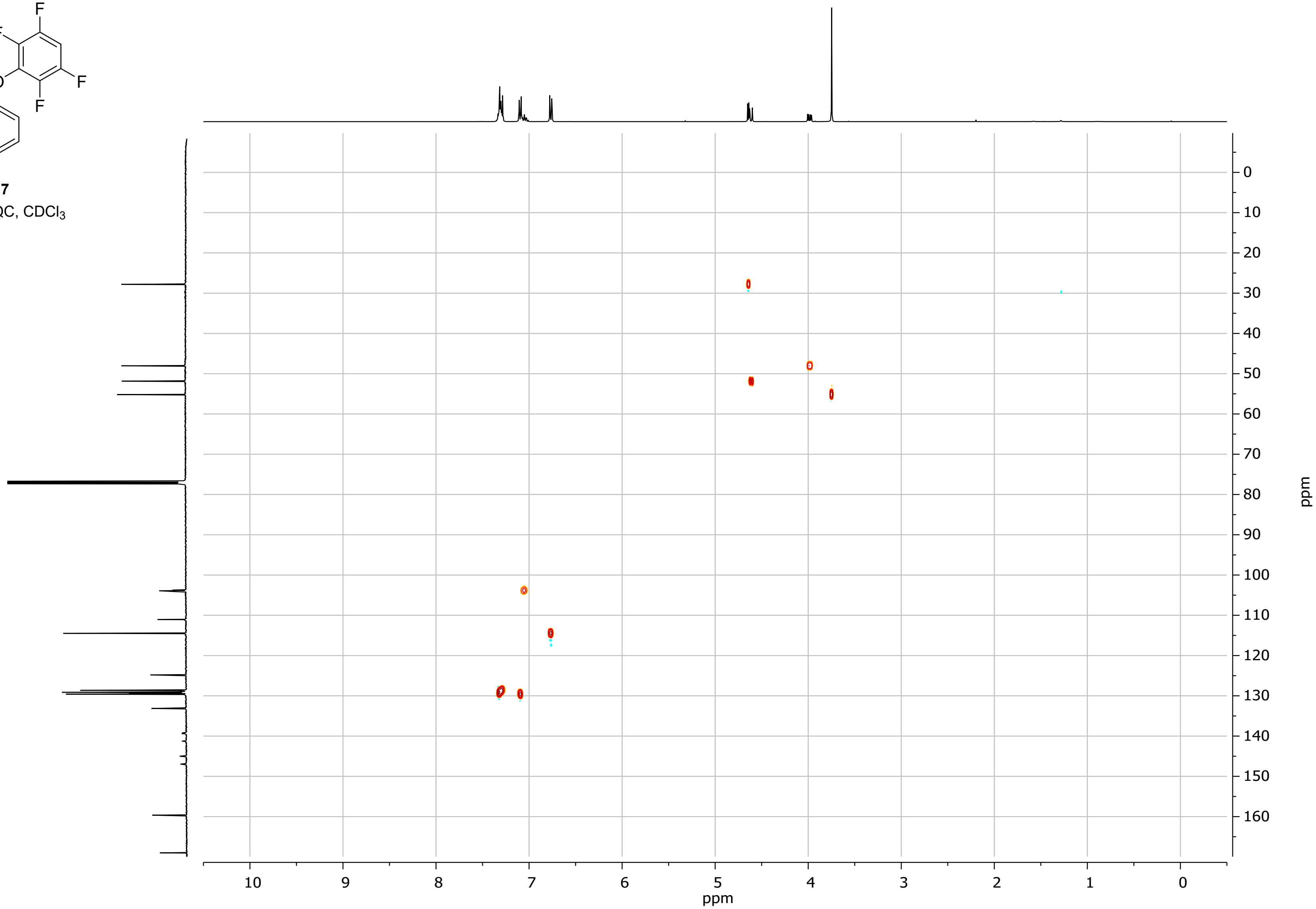

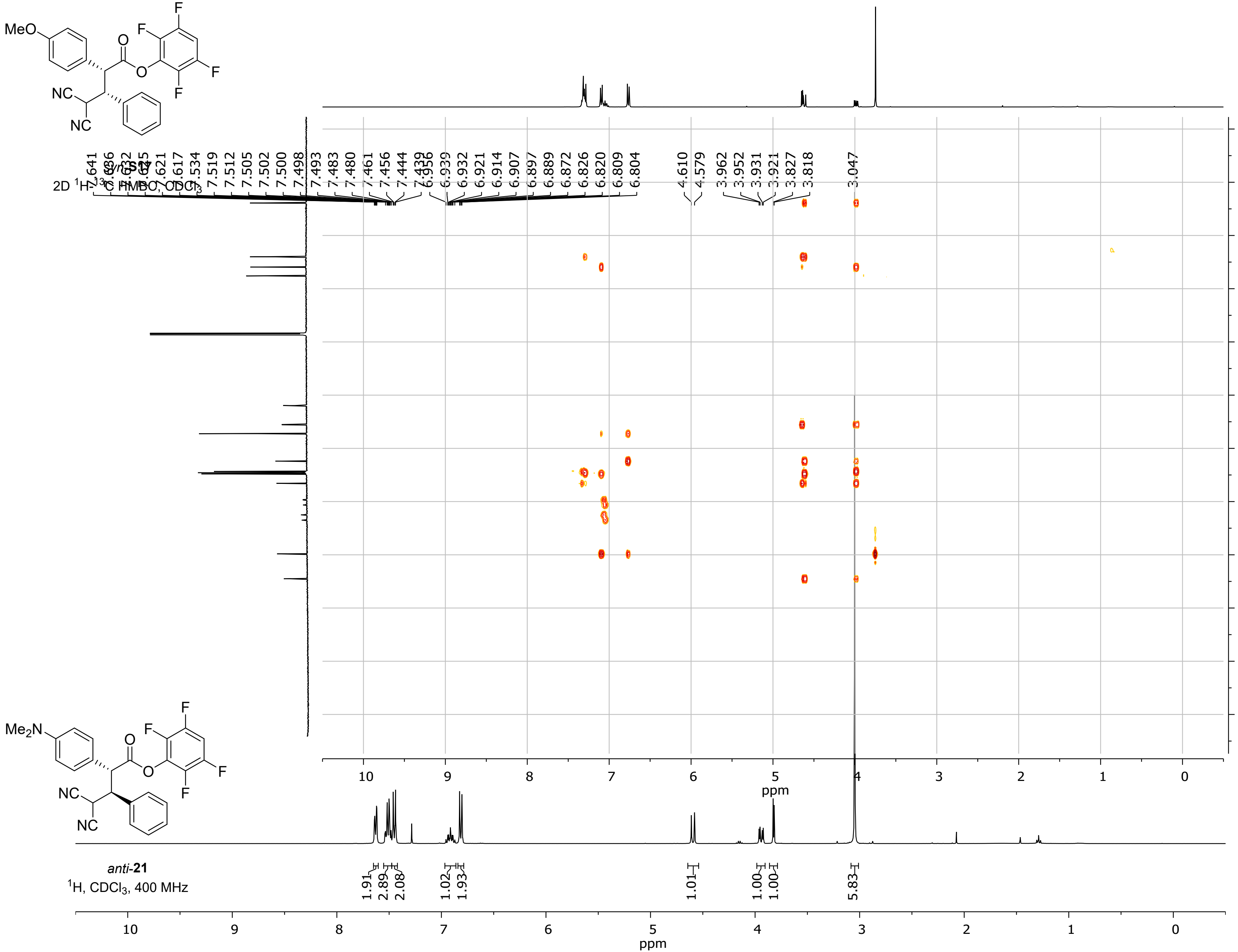

MeO
F
F
F
F
O
O
NC
NC
syn-S17
2D 1H-13C HMBC, CDCl3
7.641 7.636 7.632 7.625 7.621 7.617 7.534 7.519 7.512 7.505 7.502 7.500 7.498 7.493 7.483 7.480 7.461 7.456 7.444 7.439 6.956 6.939 6.932 6.921 6.914 6.907 6.897 6.889 6.872 6.826 6.820 6.809 6.804 4.610 4.579 3.962 3.952 3.931 3.921 3.827 3.818 3.047
ppm
Me2N
anti-21
1H, CDCl3, 400 MHz
1.91 2.89 2.08 1.02 1.93 1.01 1.00 1.00 5.83
ppm

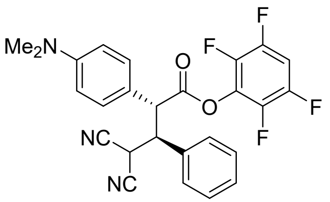

anti-**21**

$^{19}F\{^1H\}$, $CDCl_3$, 376 MHz

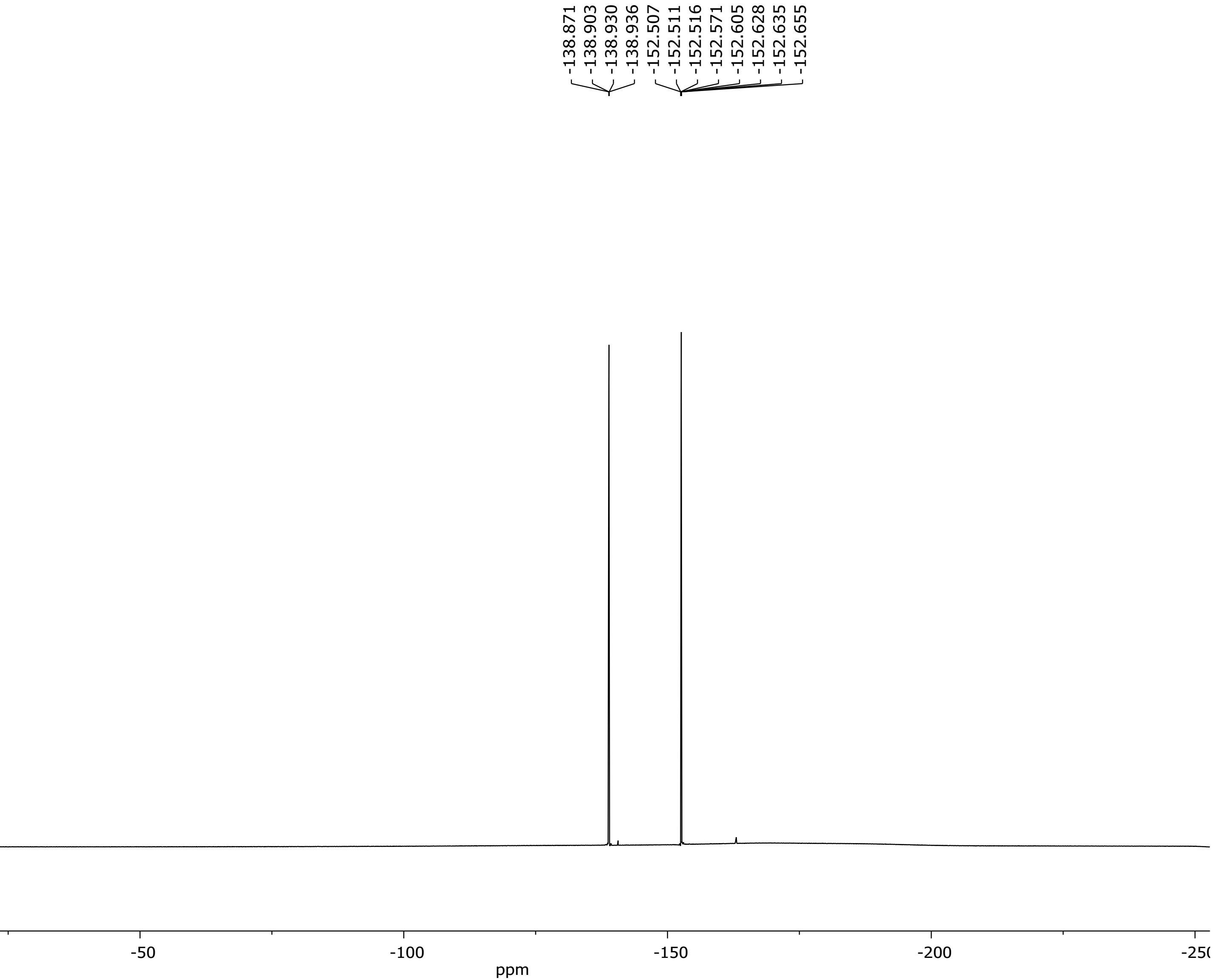

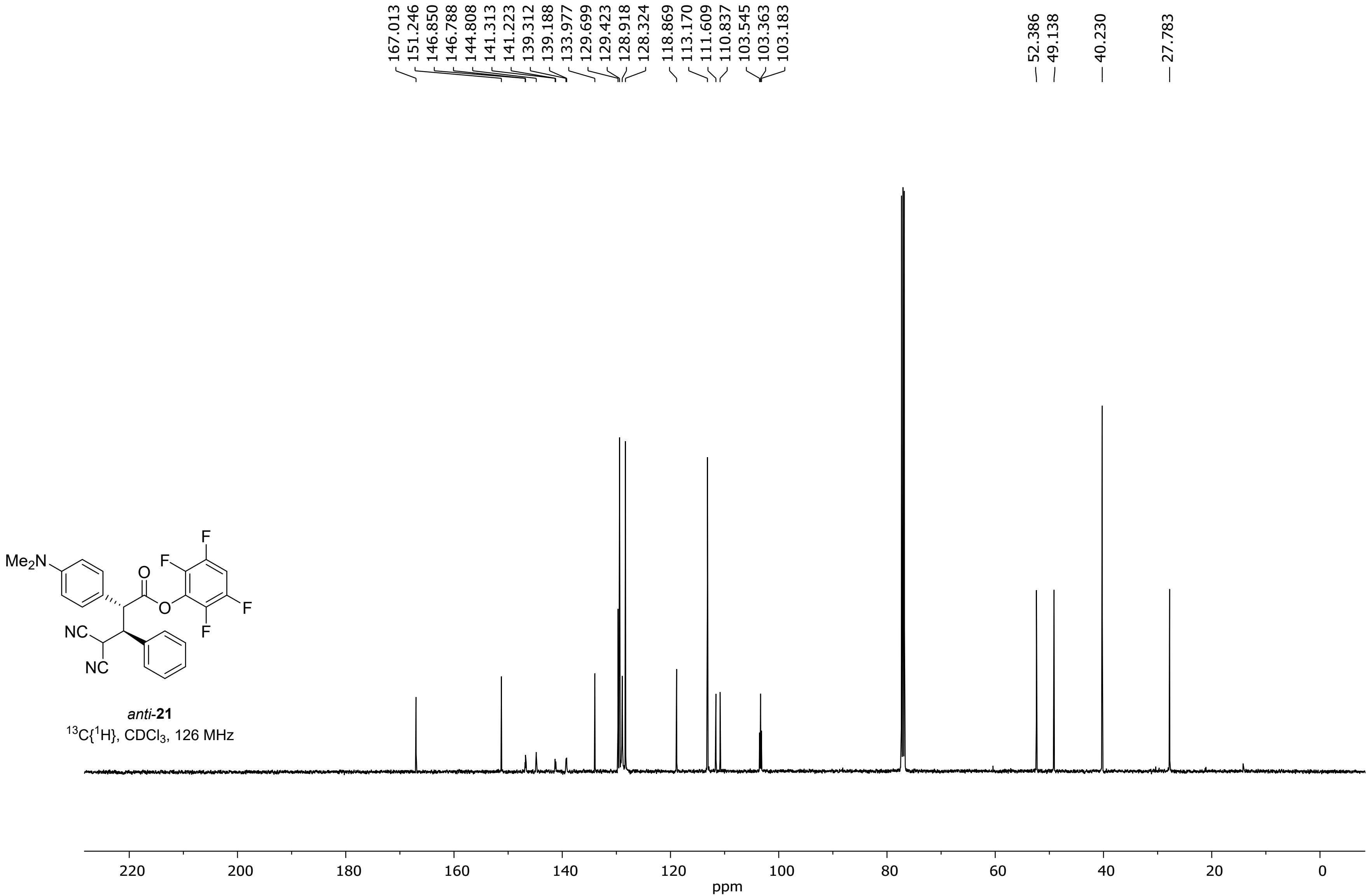

anti-**21**
$^{13}C\{^1H\}$, $CDCl_3$, 126 MHz

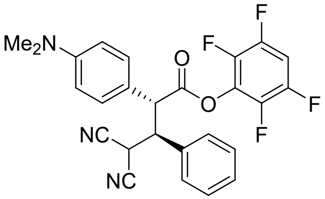

anti-**21**

2D ^{1}H-^{1}H COSY, $CDCl_3$

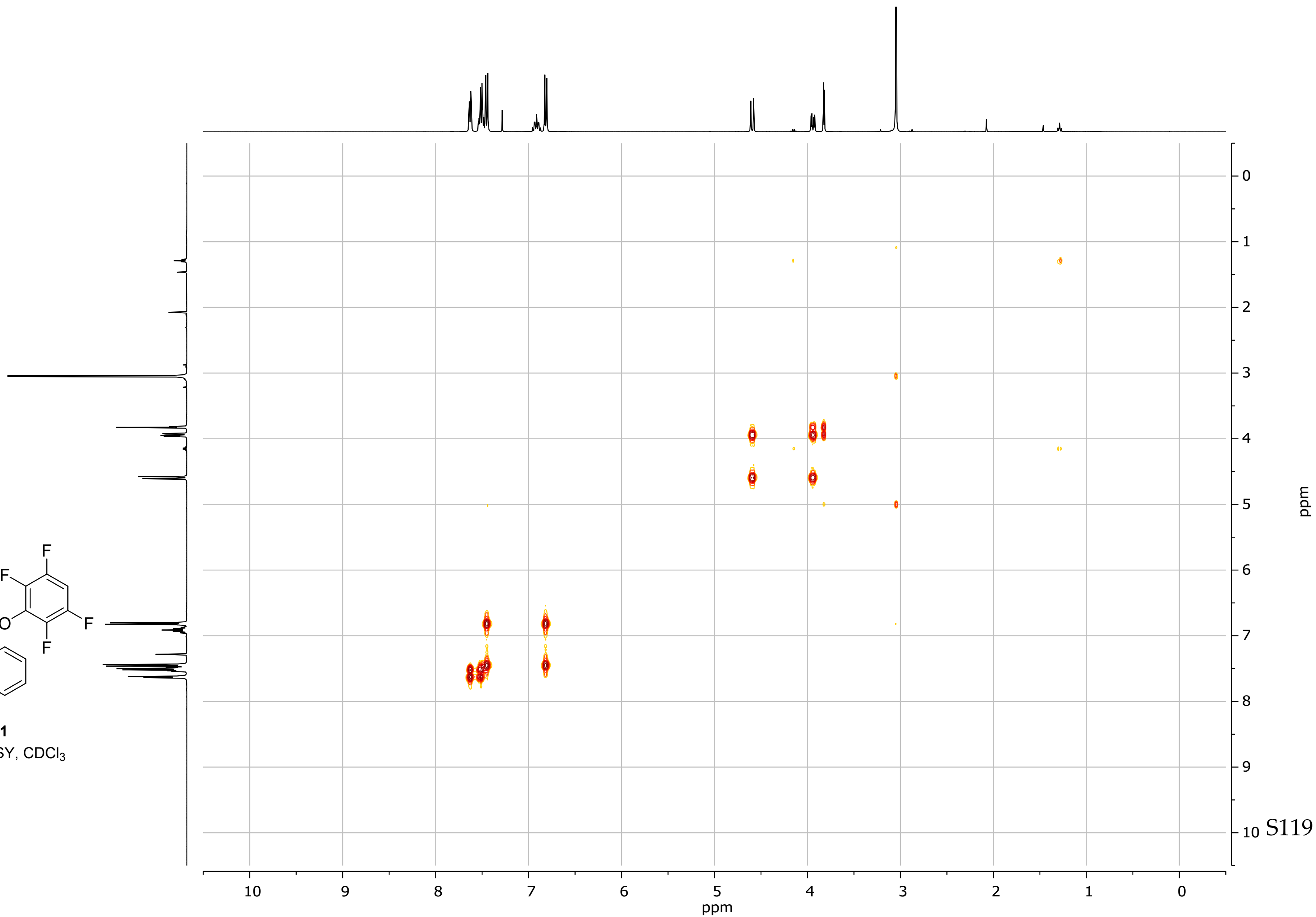

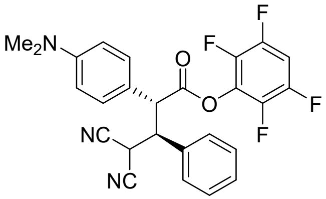

anti-**21**
2D ^{1}H-^{13}C HSQC, $CDCl_3$

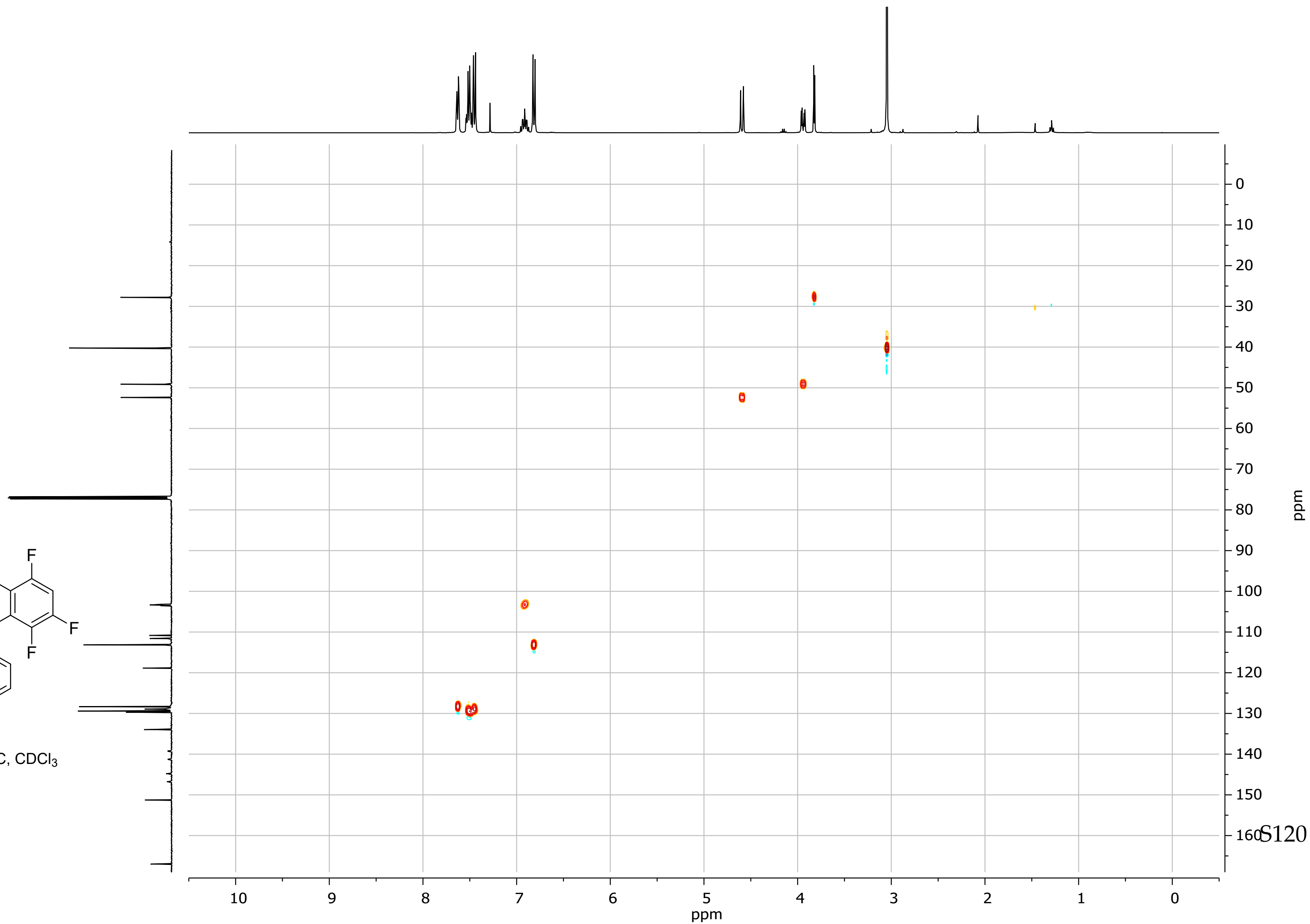

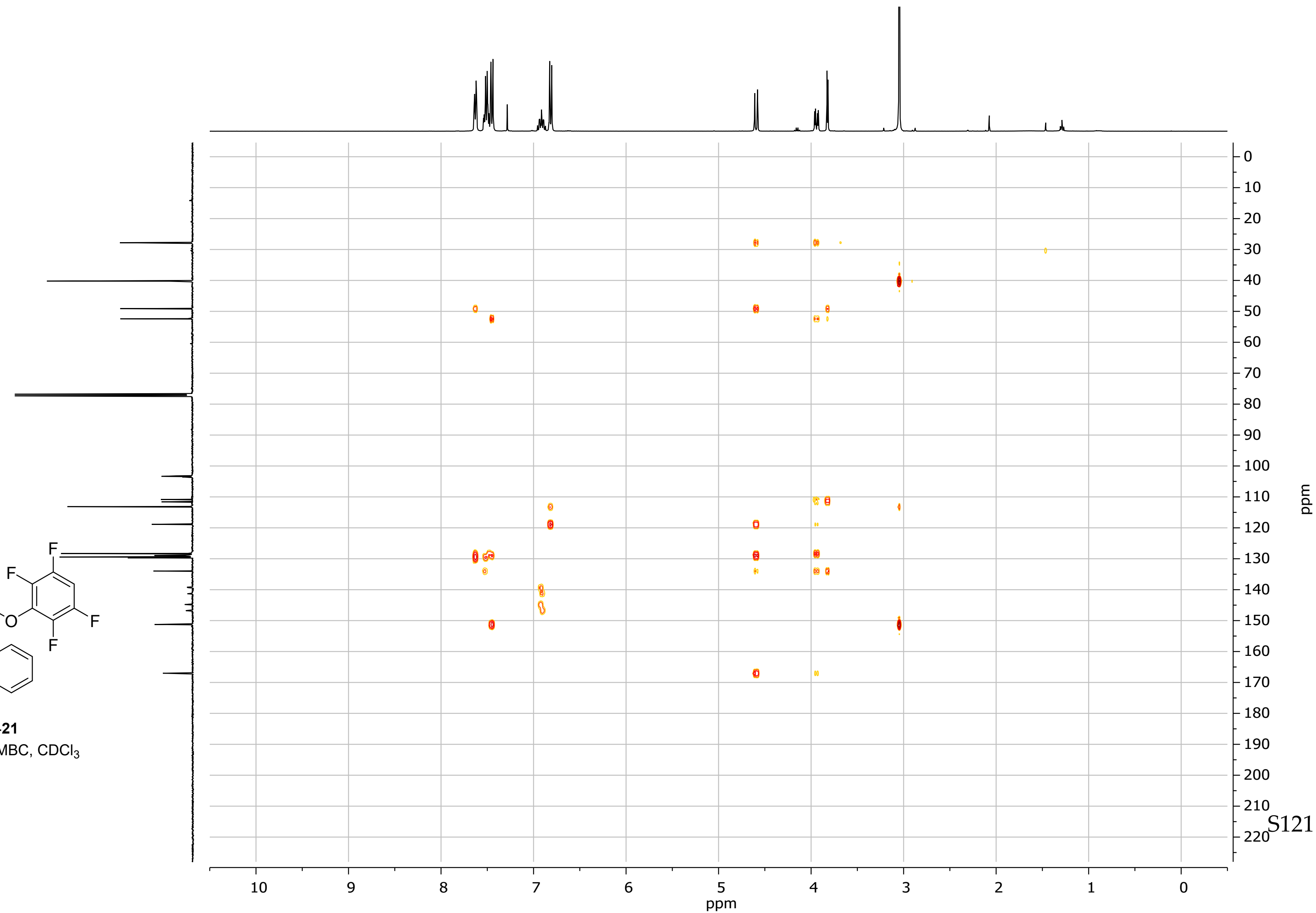

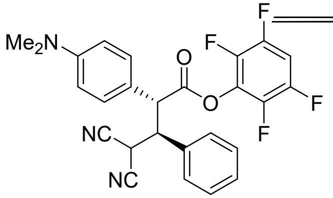

anti-**21**

2D ^{1}H-^{13}C HMBC, $CDCl_3$

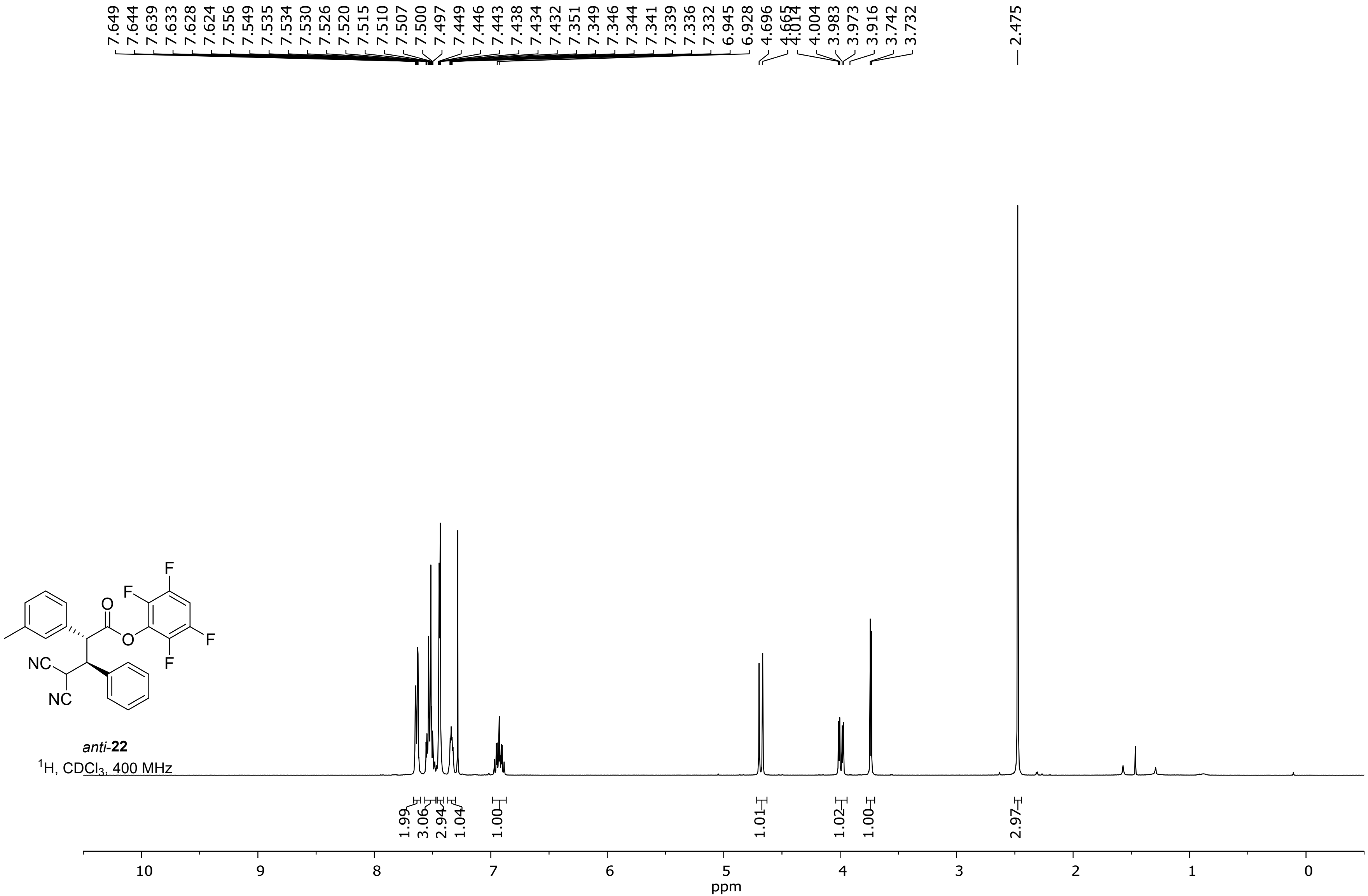
7.649
7.644
7.639
7.633
7.628
7.624
7.556
7.549
7.535
7.534
7.530
7.526
7.520
7.515
7.510
7.507
7.500
7.497
7.449
7.446
7.443
7.438
7.434
7.432
7.351
7.349
7.346
7.344
7.341
7.339
7.336
7.332
6.945
6.928
4.696
4.665
4.014
4.004
3.983
3.973
3.916
3.742
3.732
2.475
F
F
O
O
F
NC
F
NC
anti-22
1H, CDCl3, 400 MHz
1.99
3.06
2.94
1.04
1.00
1.01
1.02
1.00
2.97
10
9
8
7
6
5
4
3
2
1
0
ppm

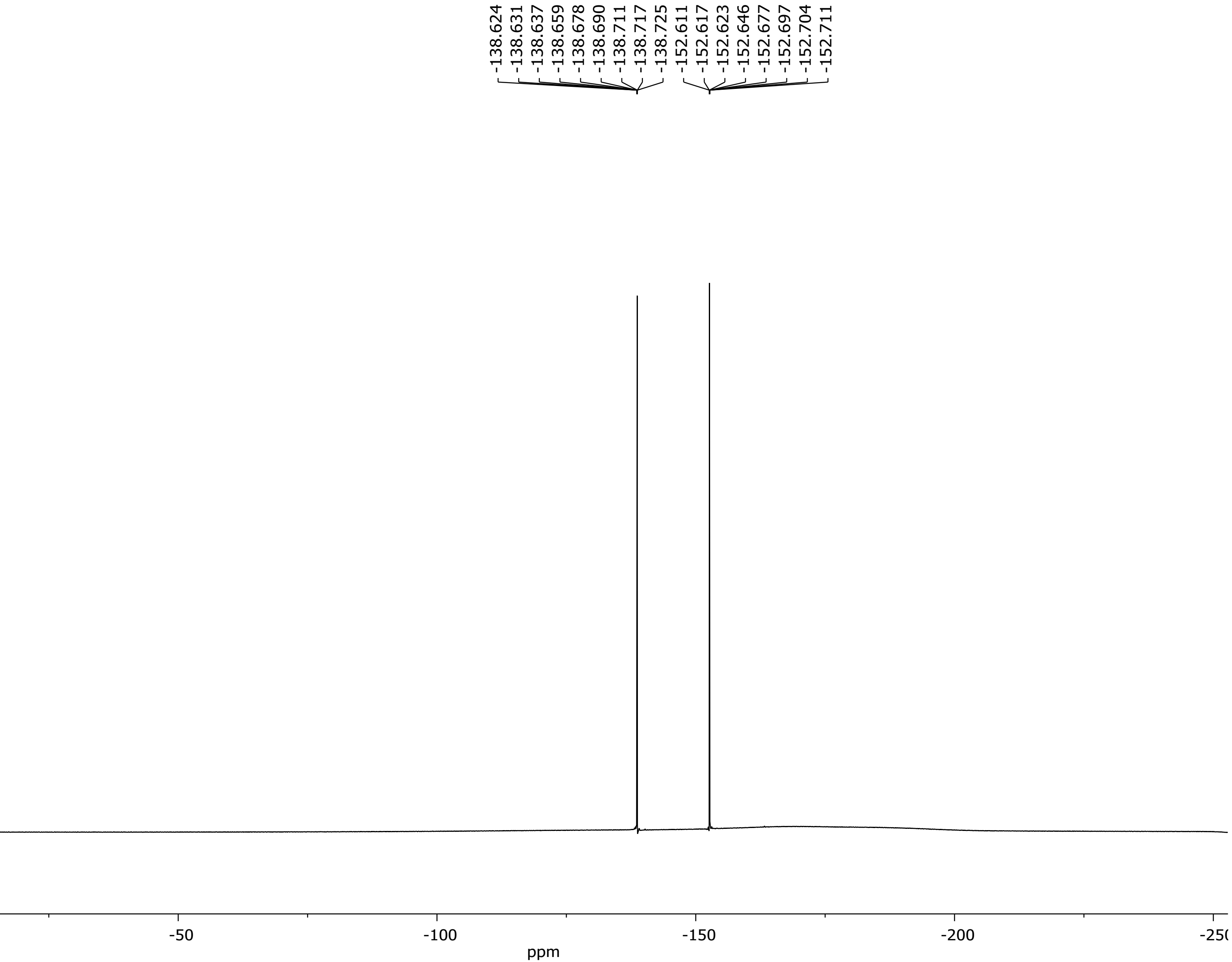

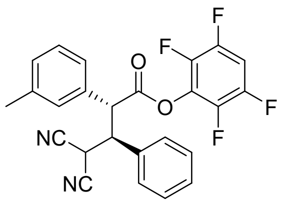

anti-**22**

$^{19}F\{^{1}H\}$, $CDCl_3$, 376 MHz

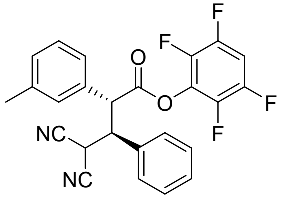

anti-**22**

$^{13}C\{^{1}H\}$, $CDCl_3$, 126 MHz

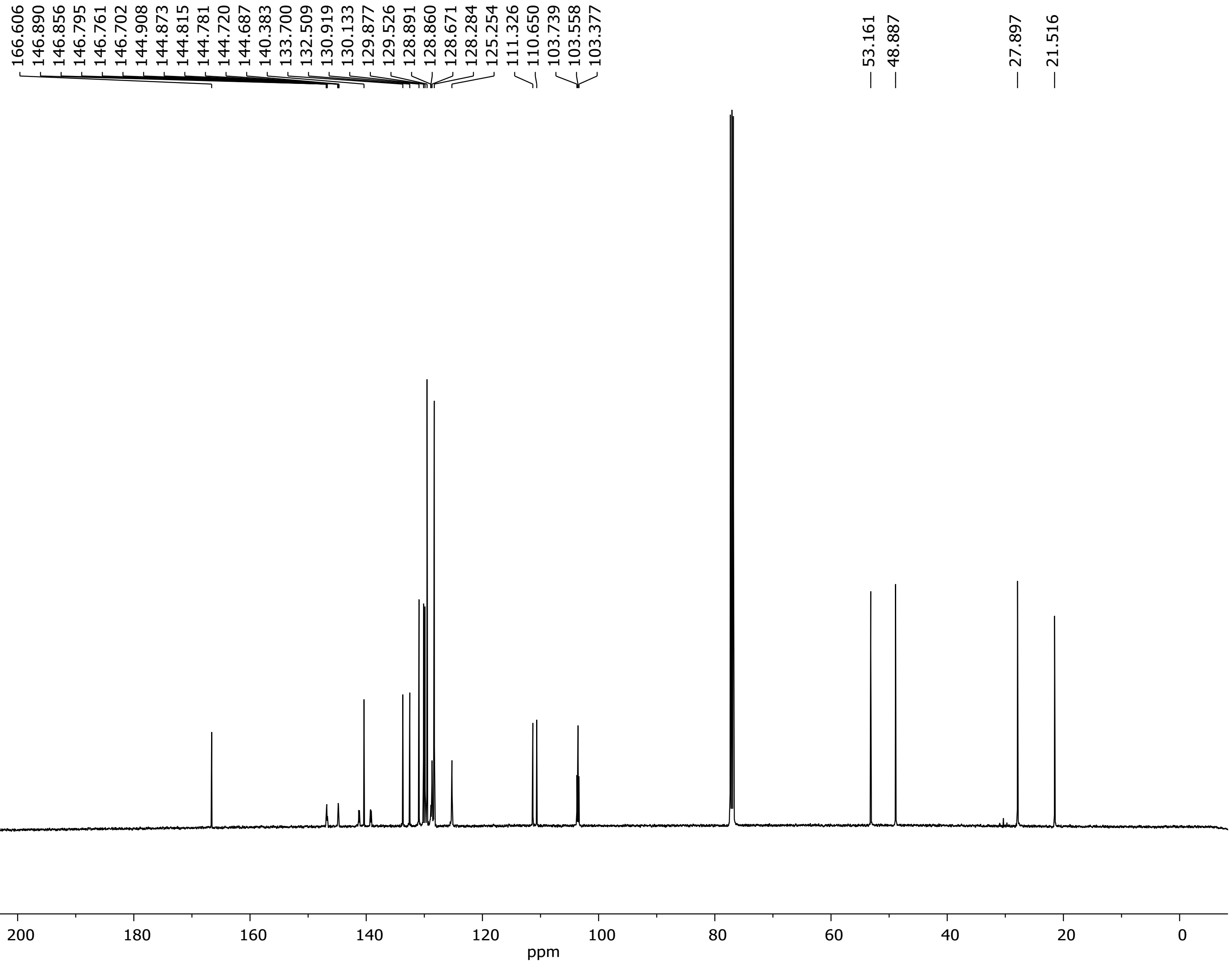

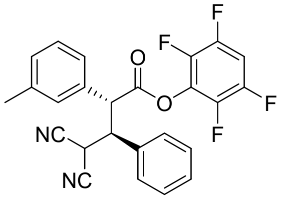

anti-**22**

2D ^{1}H-^{1}H COSY, $CDCl_3$

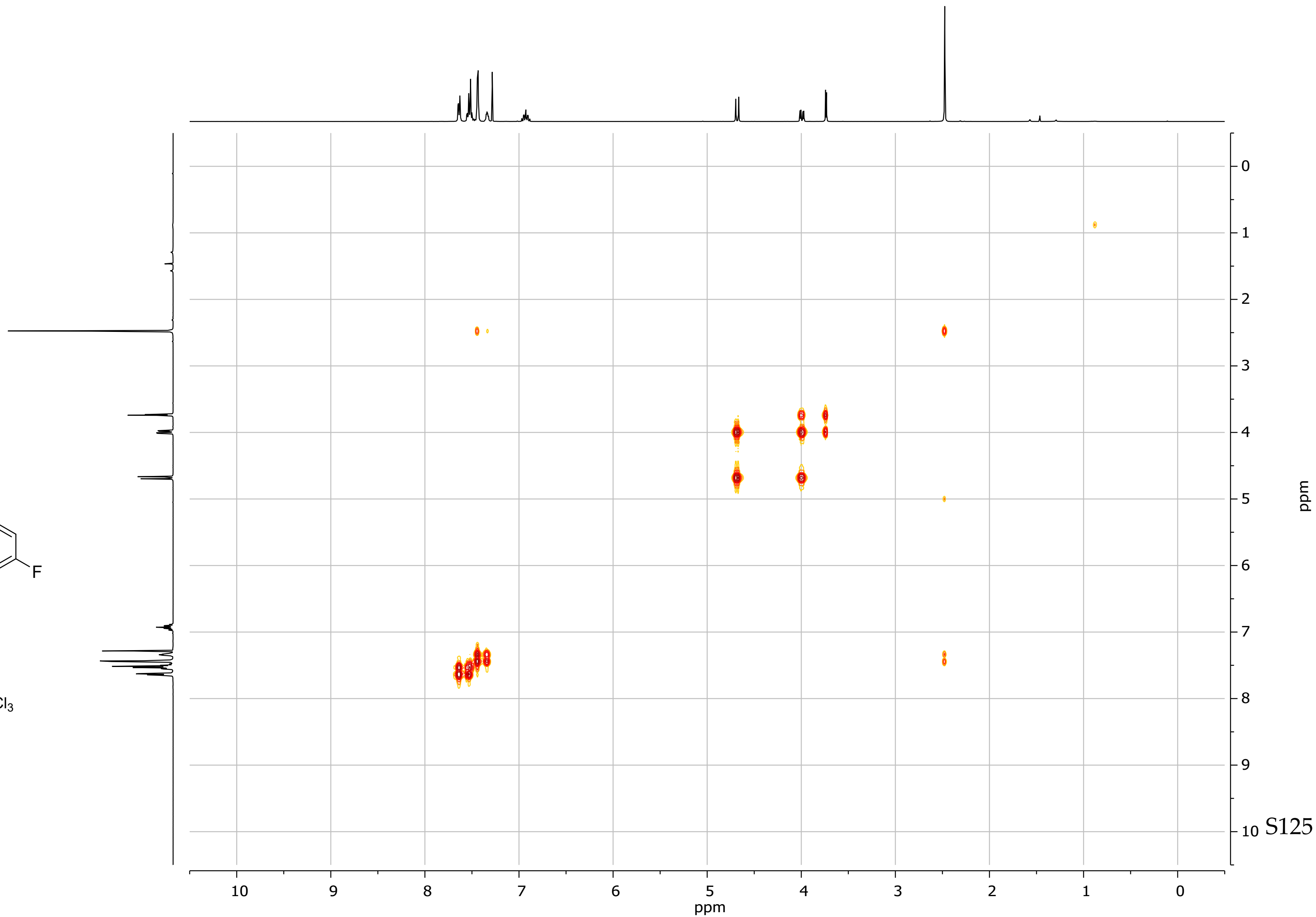

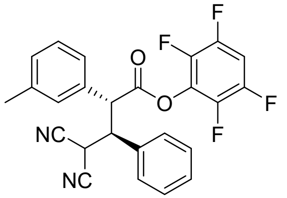

anti-**22**
2D ^{1}H-^{13}C HSQC, $CDCl_3$

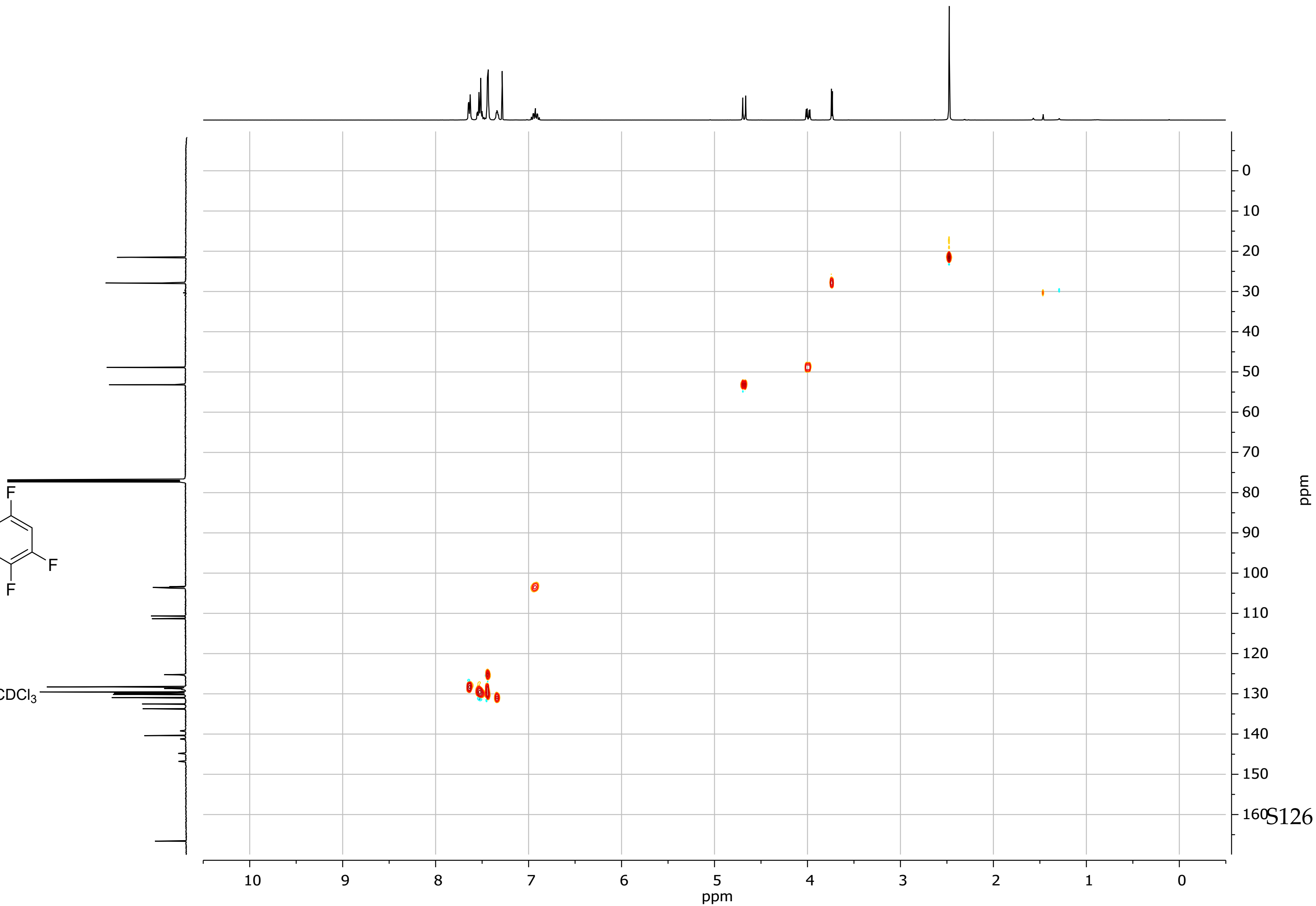

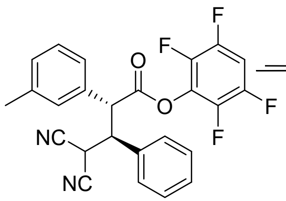

anti-**22**

2D ^{1}H-^{13}C HMBC, $CDCl_3$

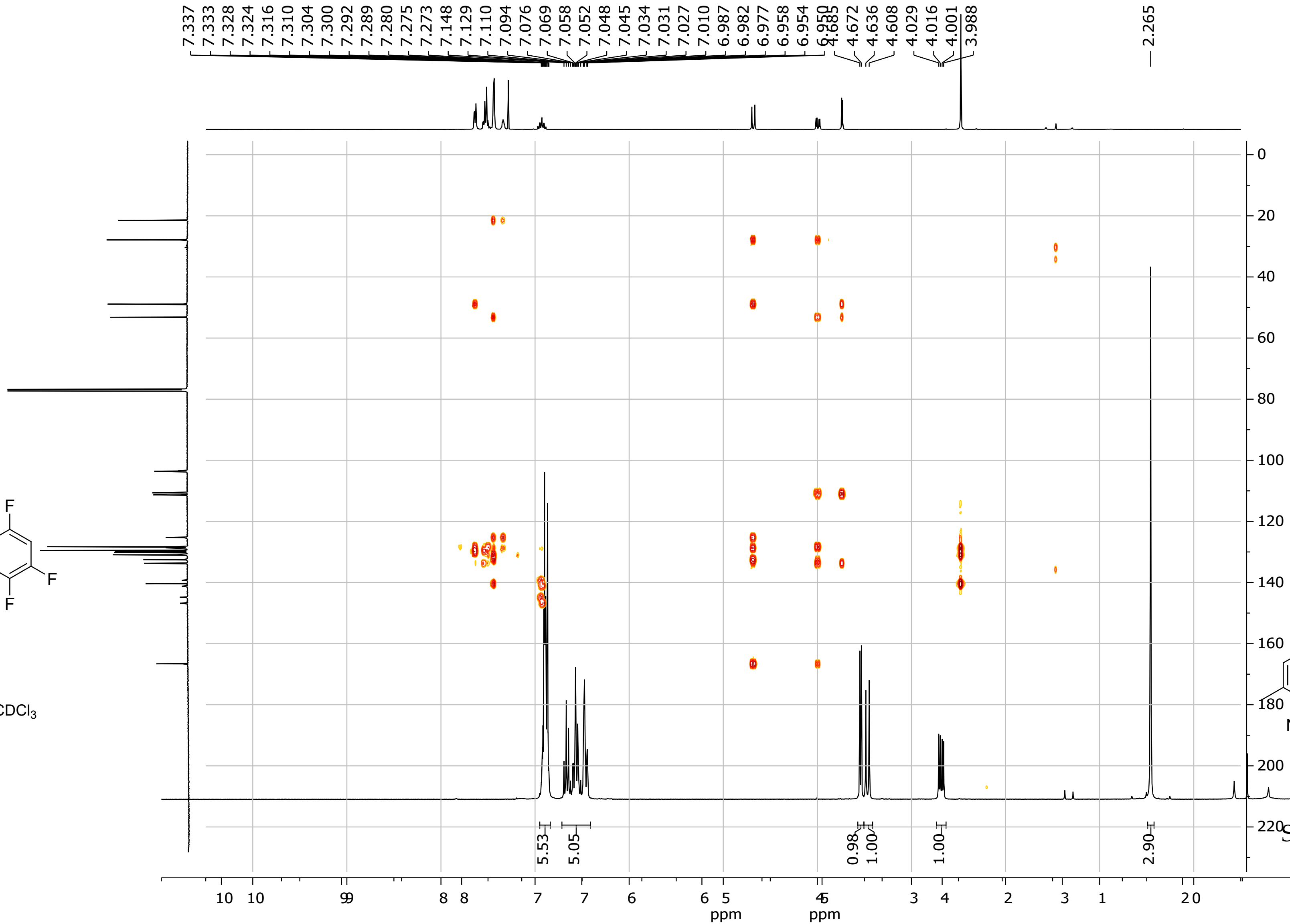

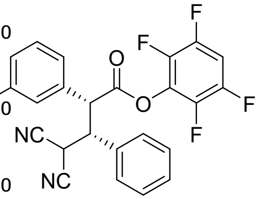

syn-**S18**

^{1}H, $CDCl_3$, 400 MHz

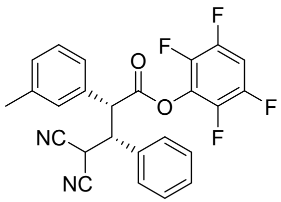

syn-**S18**

$^{19}F\{^1H\}$, $CDCl_3$, 376 MHz

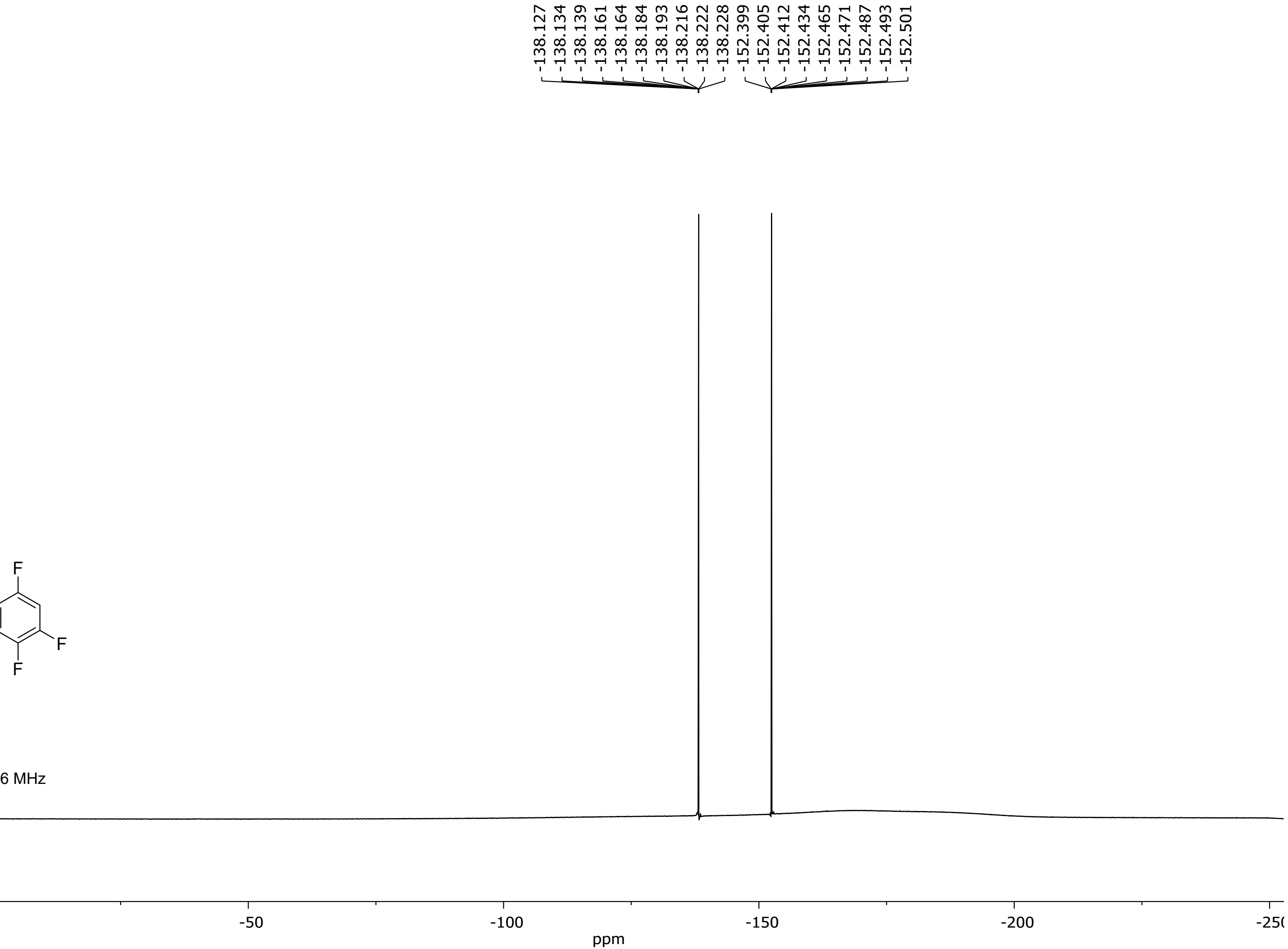

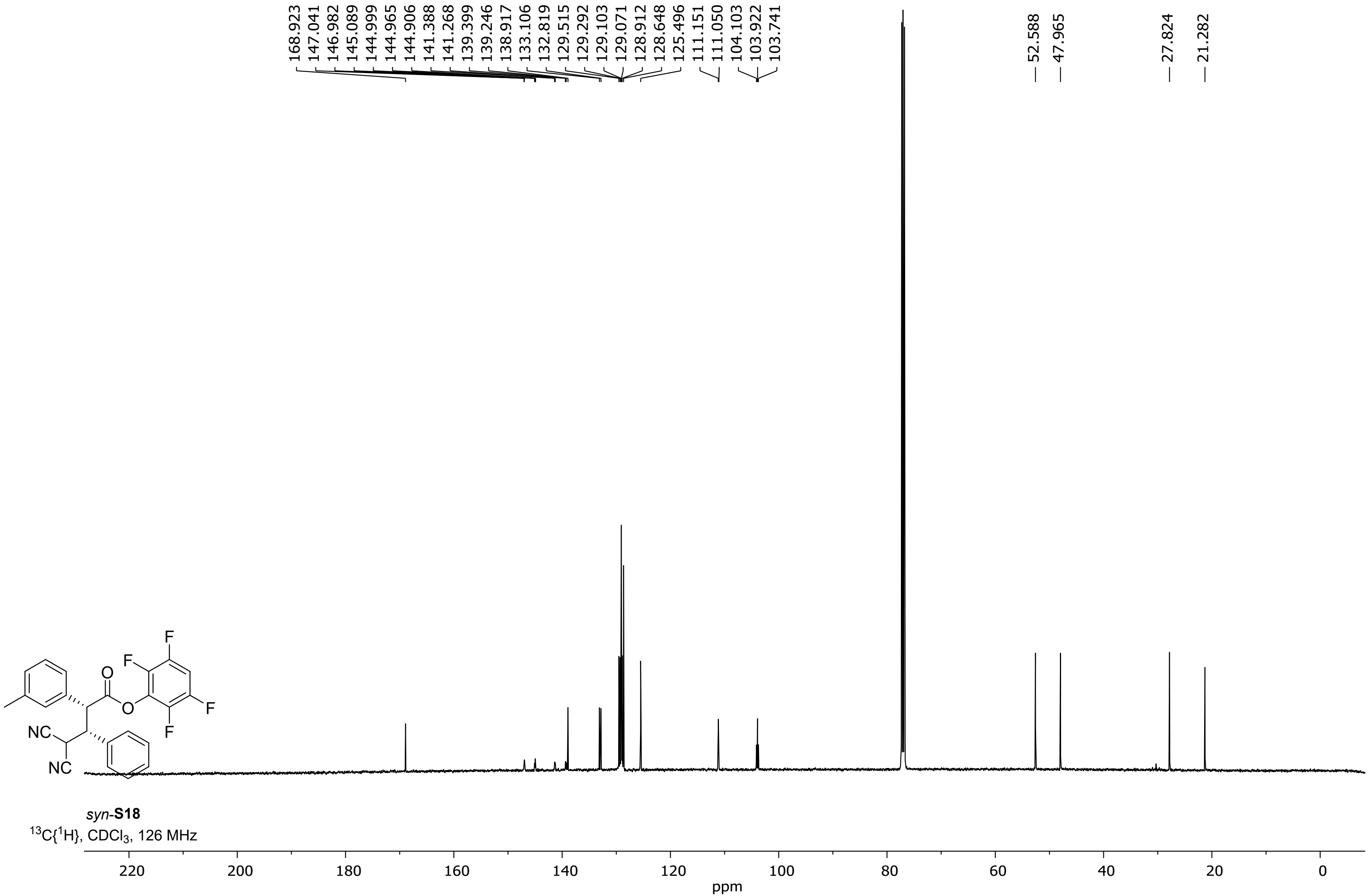

syn-**S18**
$^{13}C\{^1H\}$, $CDCl_3$, 126 MHz

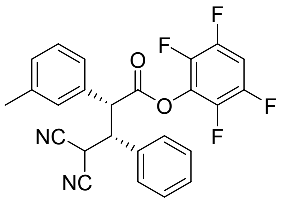

syn-**S18**

2D ^1H-^1H COSY, $CDCl_3$

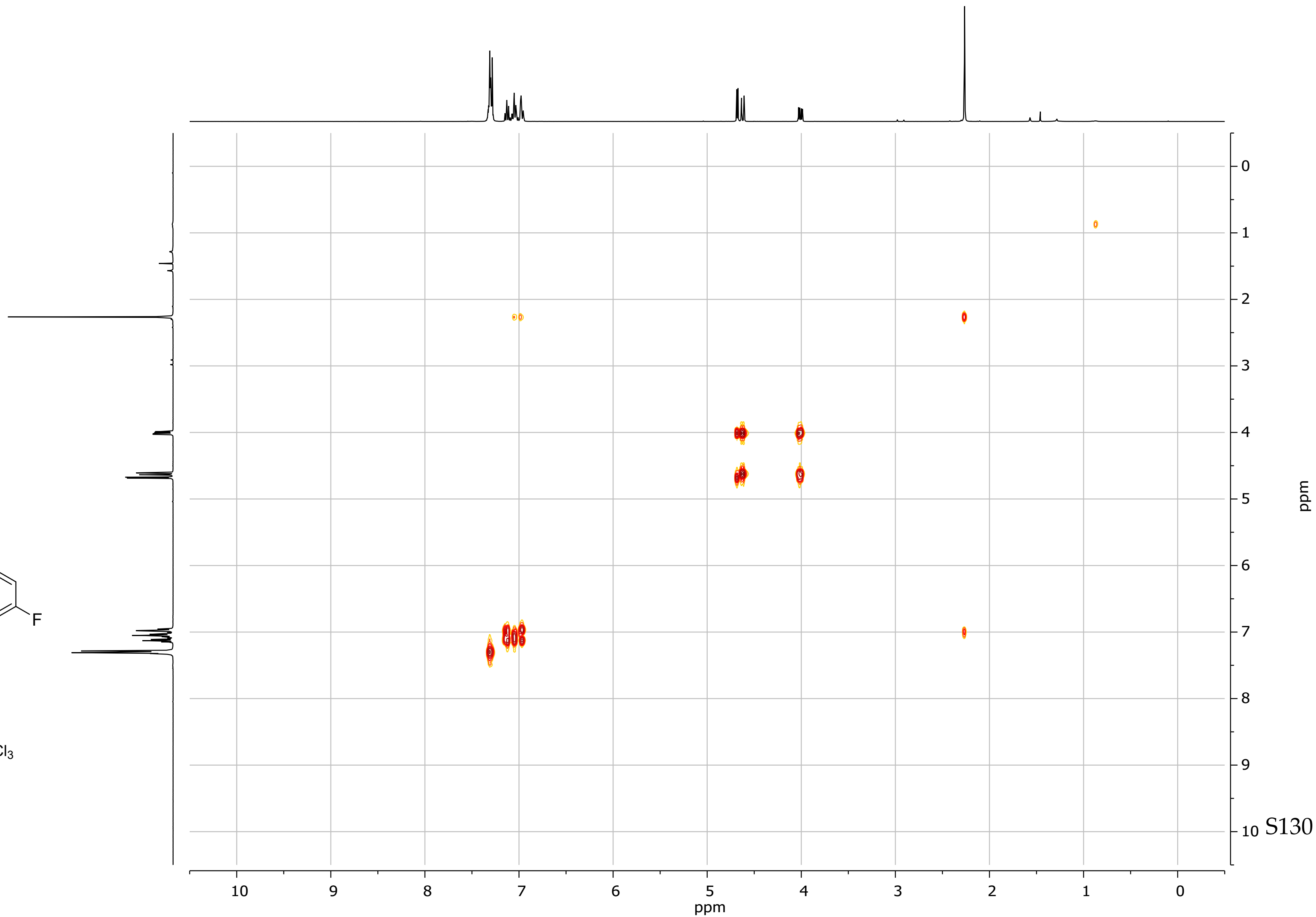

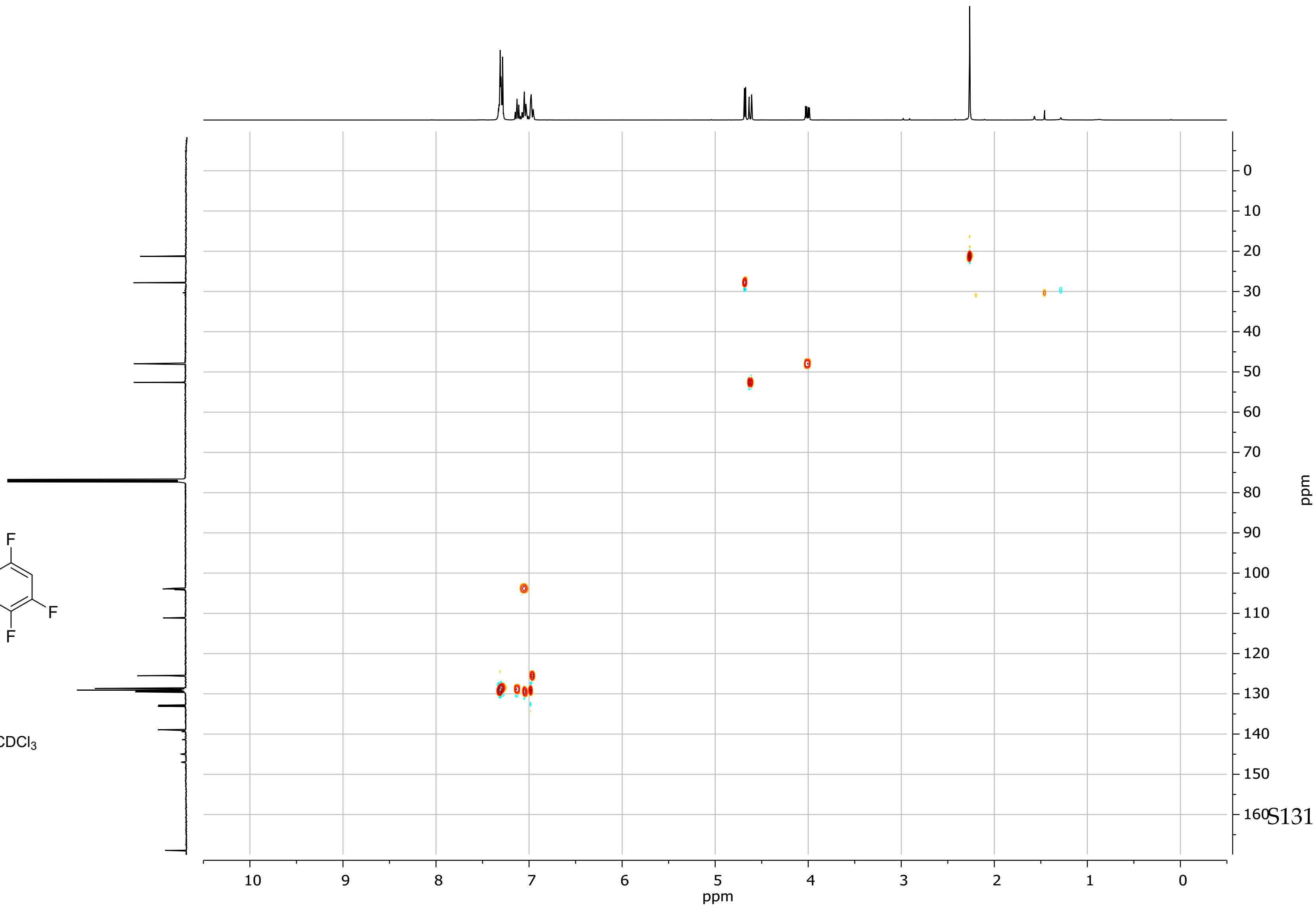

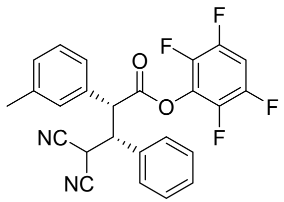

syn-**S18**
2D ^{1}H-^{13}C HSQC, $CDCl_3$

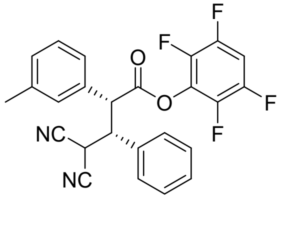

syn-**S18**
2D ^{1}H-^{13}C HMBC, $CDCl_3$

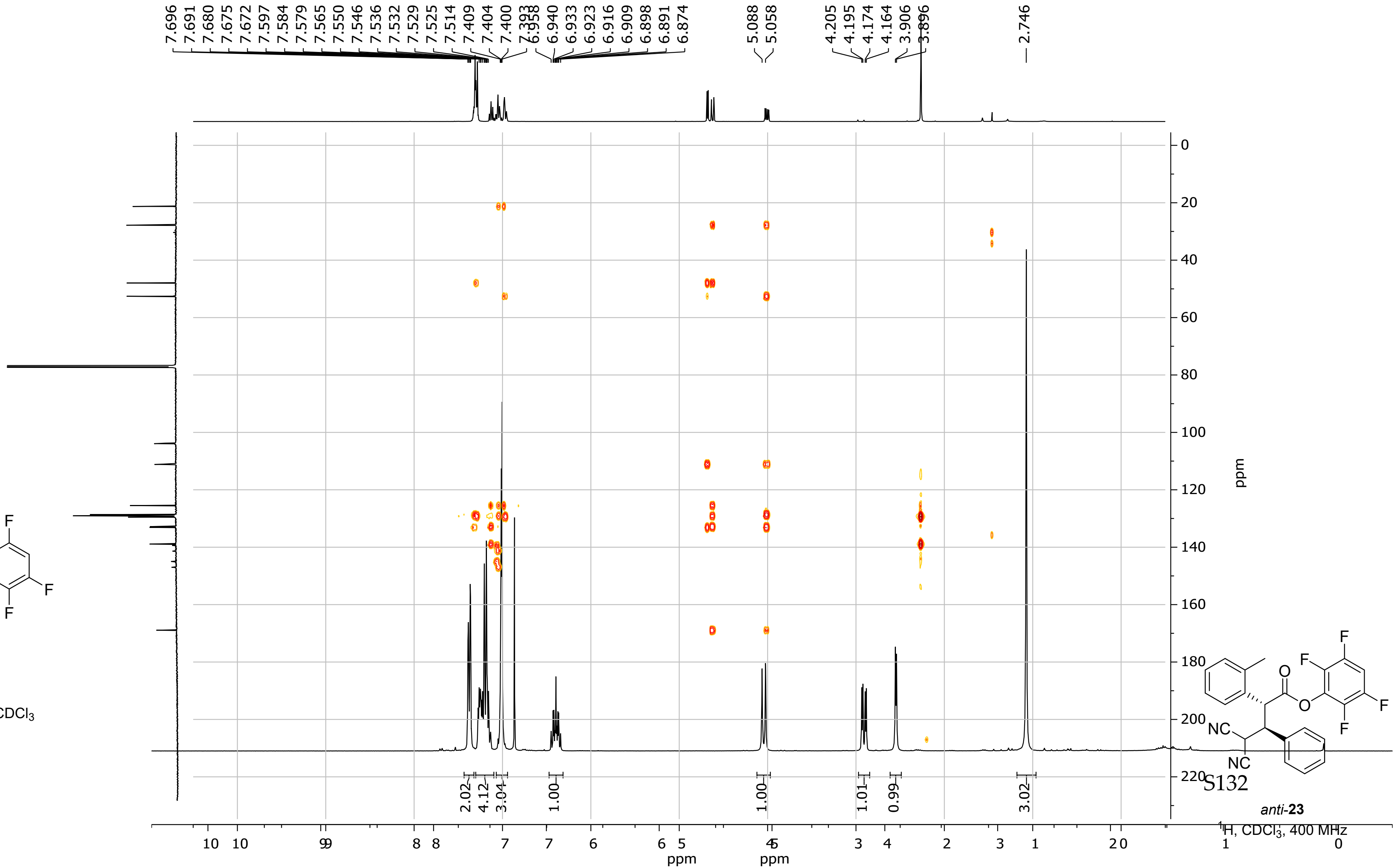

anti-**23**
^{1}H, $CDCl_3$, 400 MHz

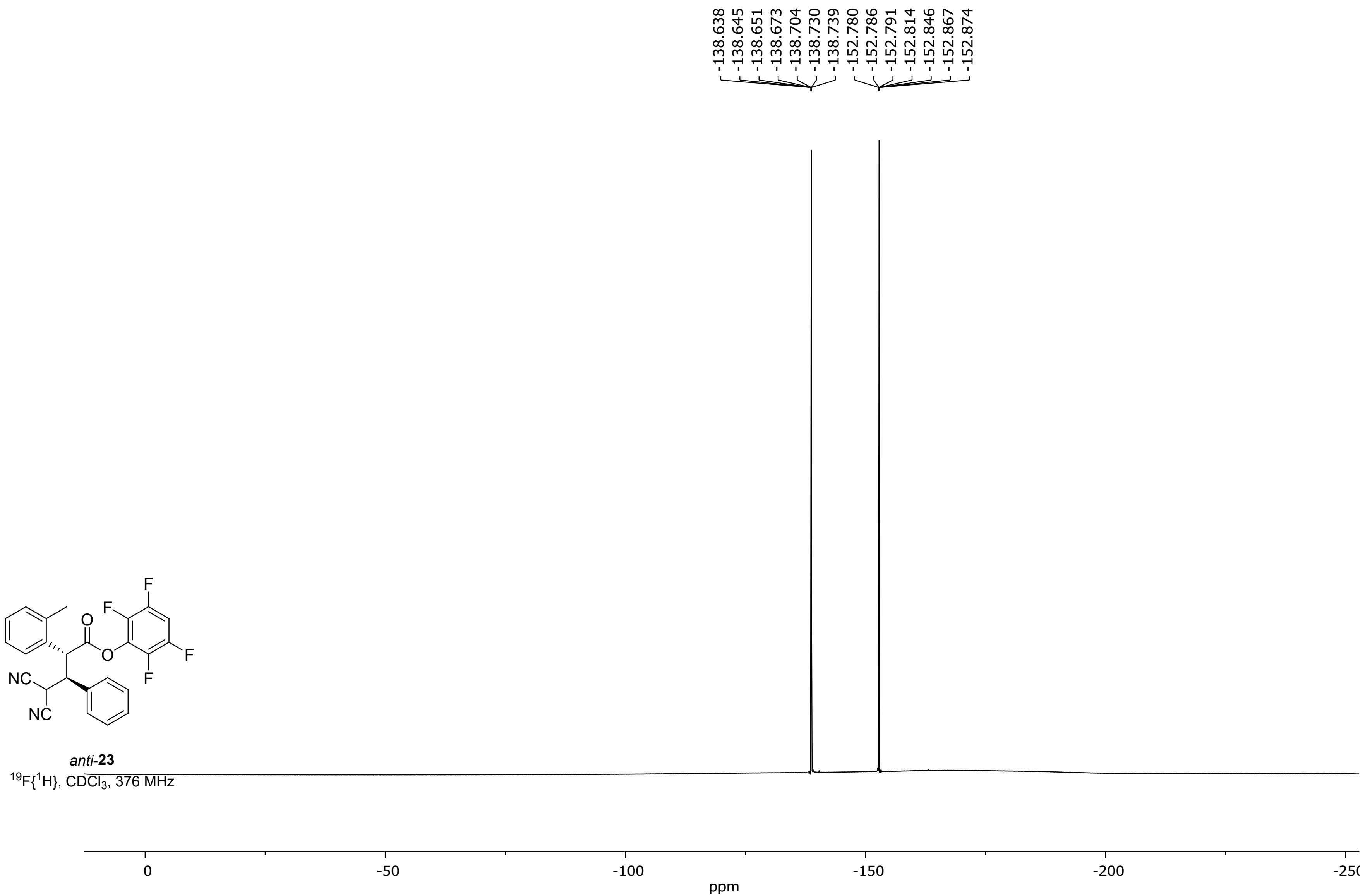
-138.638
-138.645
-138.651
-138.673
-138.704
-138.730
-138.739
-152.780
-152.786
-152.791
-152.814
-152.846
-152.867
-152.874
F
F
O
O
F
F
NC
NC
anti-**23**
$^{19}F\{^{1}H\}$, $CDCl_3$, 376 MHz
0
-50
-100
-150
-200
-25(
ppm

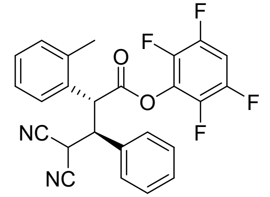

anti-**23**
$^{13}C\{^{1}H\}$, $CDCl_3$, 126 MHz

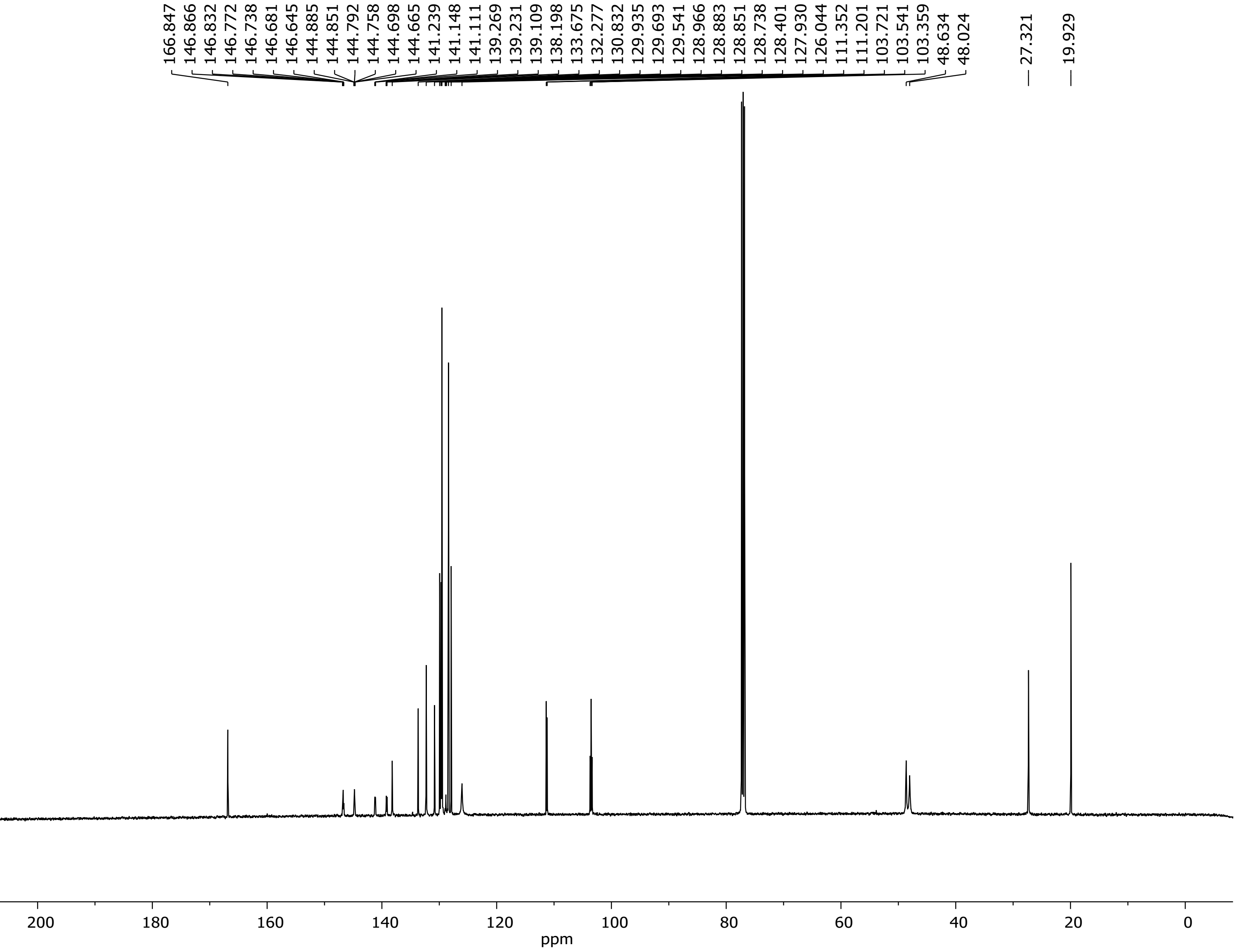

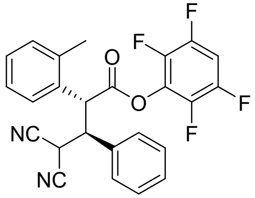

anti-**23**

2D ^{1}H-^{1}H COSY, $CDCl_3$

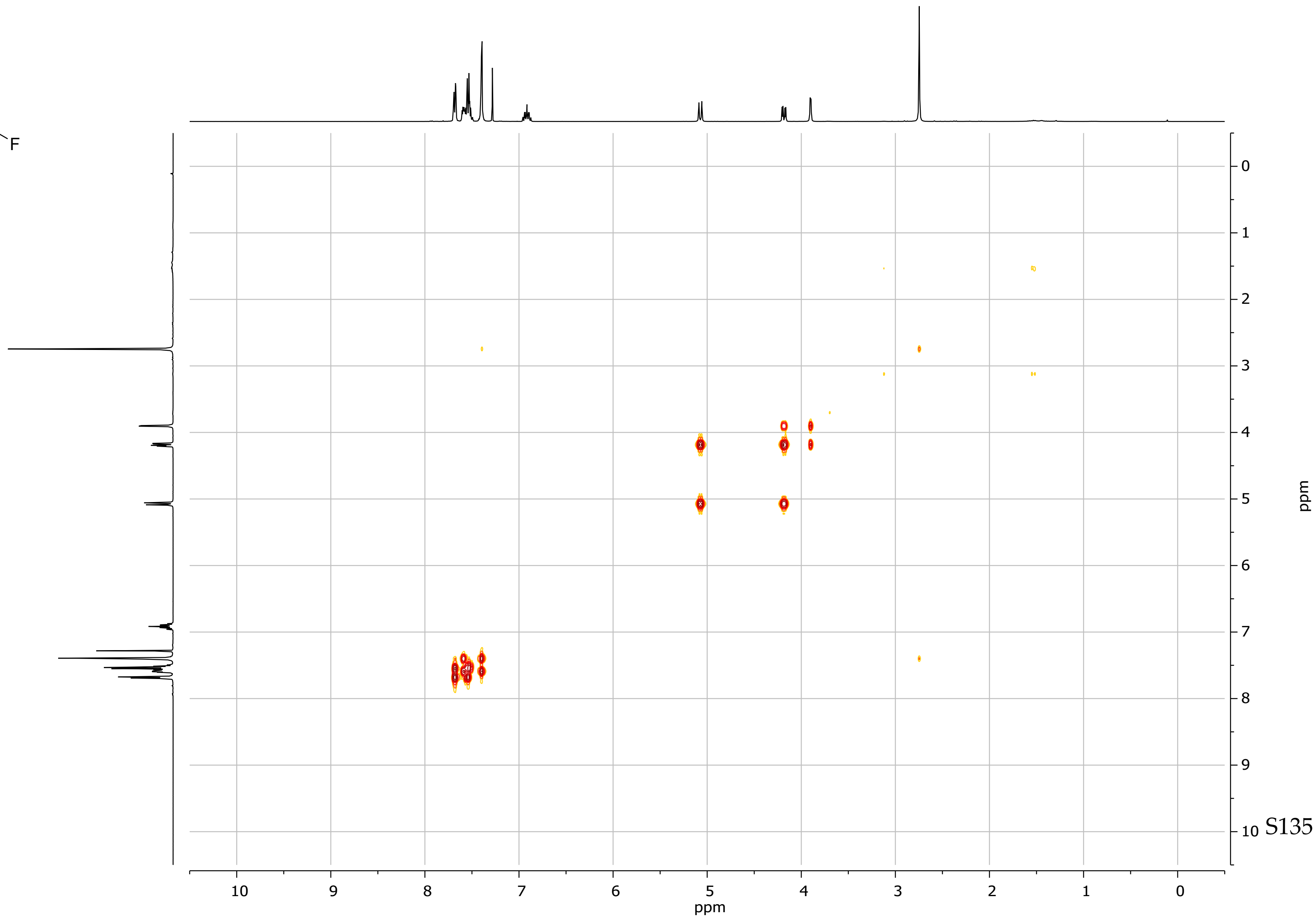

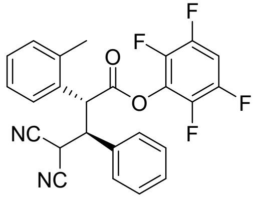

anti-**23**
2D ^{1}H-^{13}C HSQC, $CDCl_3$

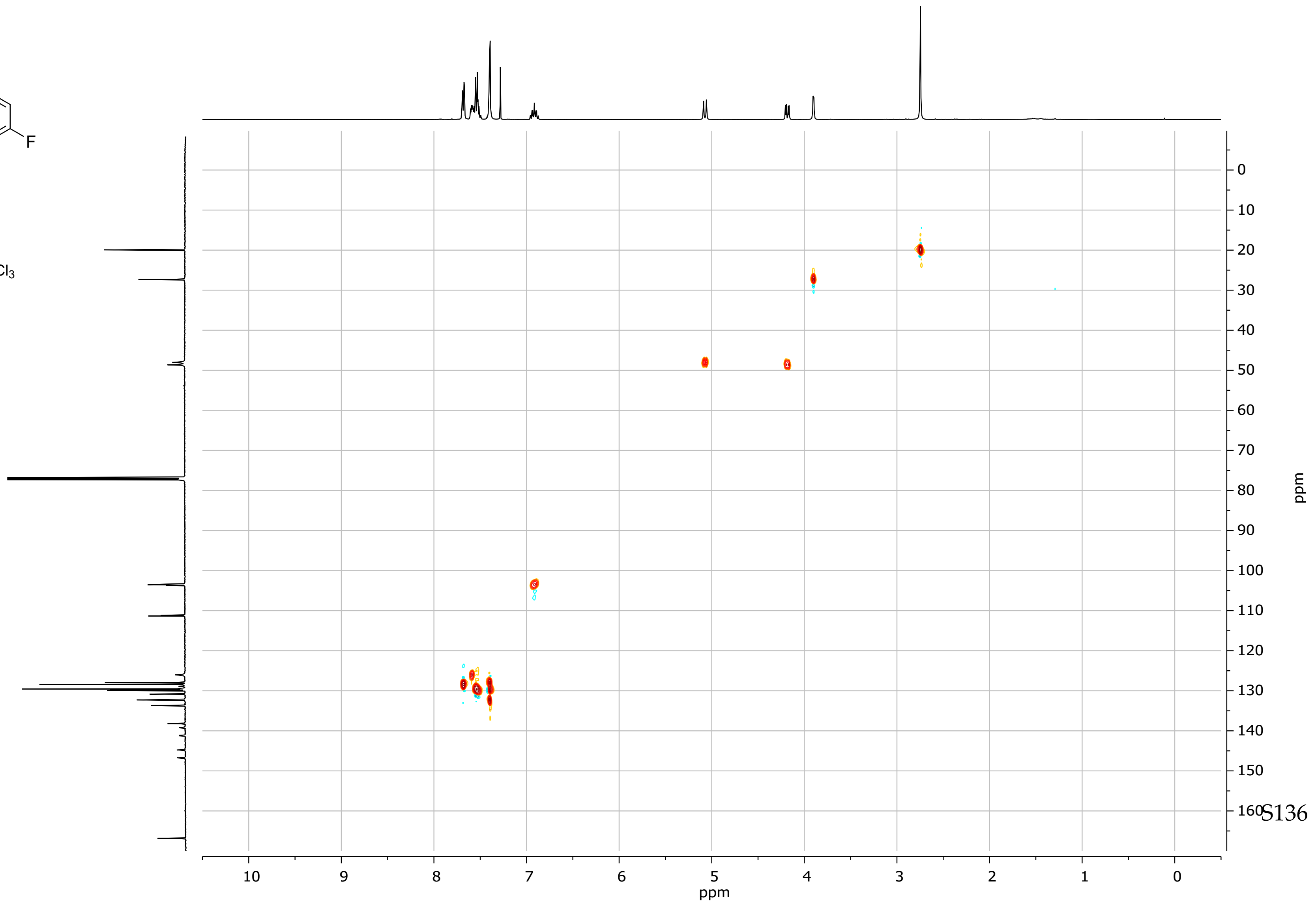

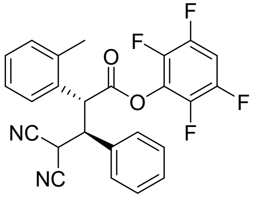

anti-**23**

2D ^{1}H-^{13}C HMBC, $CDCl_3$

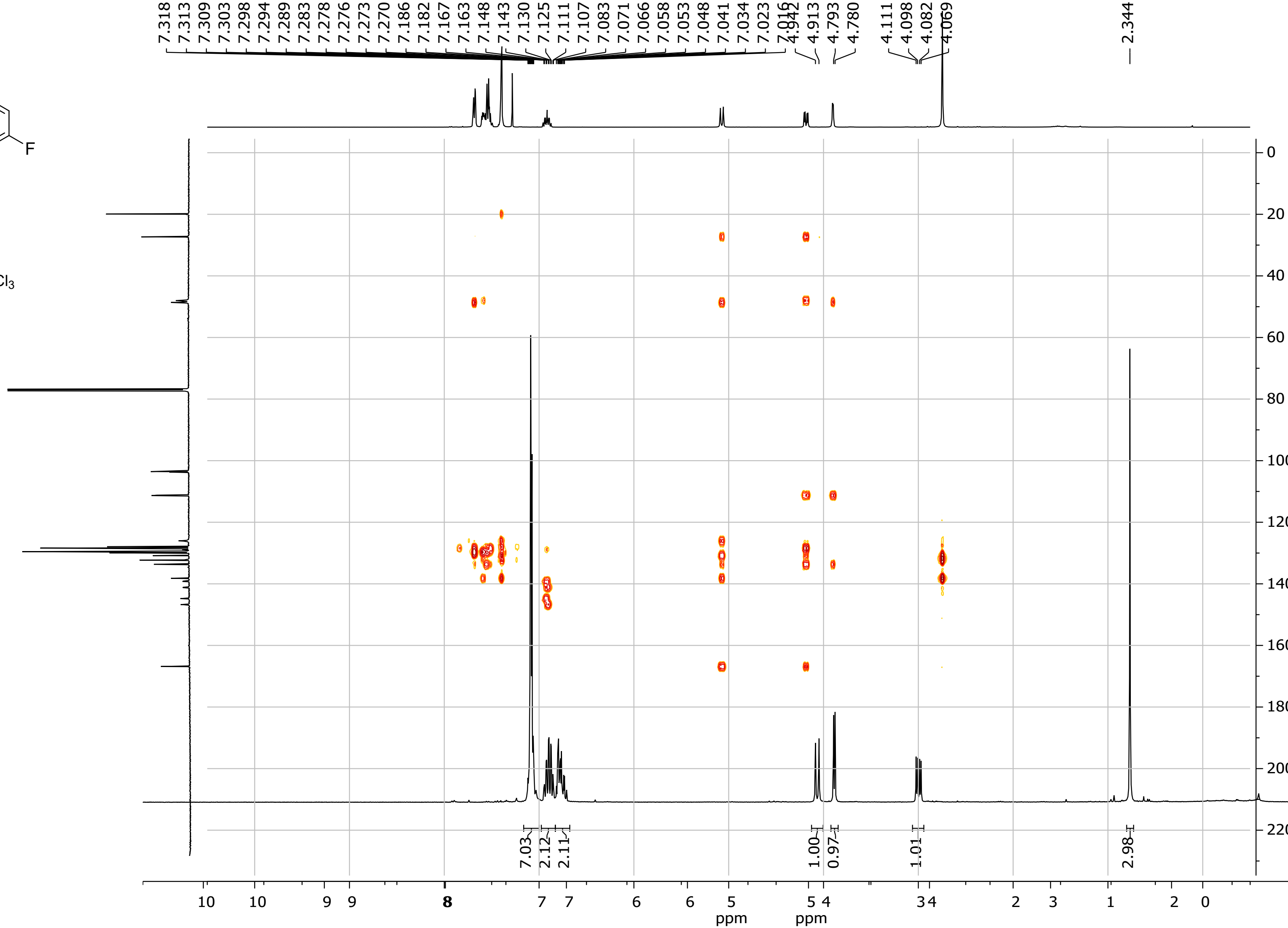

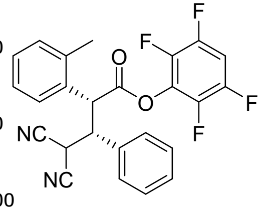

syn-**S19**

^{1}H, $CDCl_3$, 400 MHz

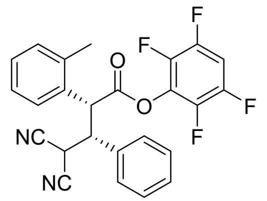

syn-**S19**
$^{19}F\{^{1}H\}$, $CDCl_3$, 376 MHz

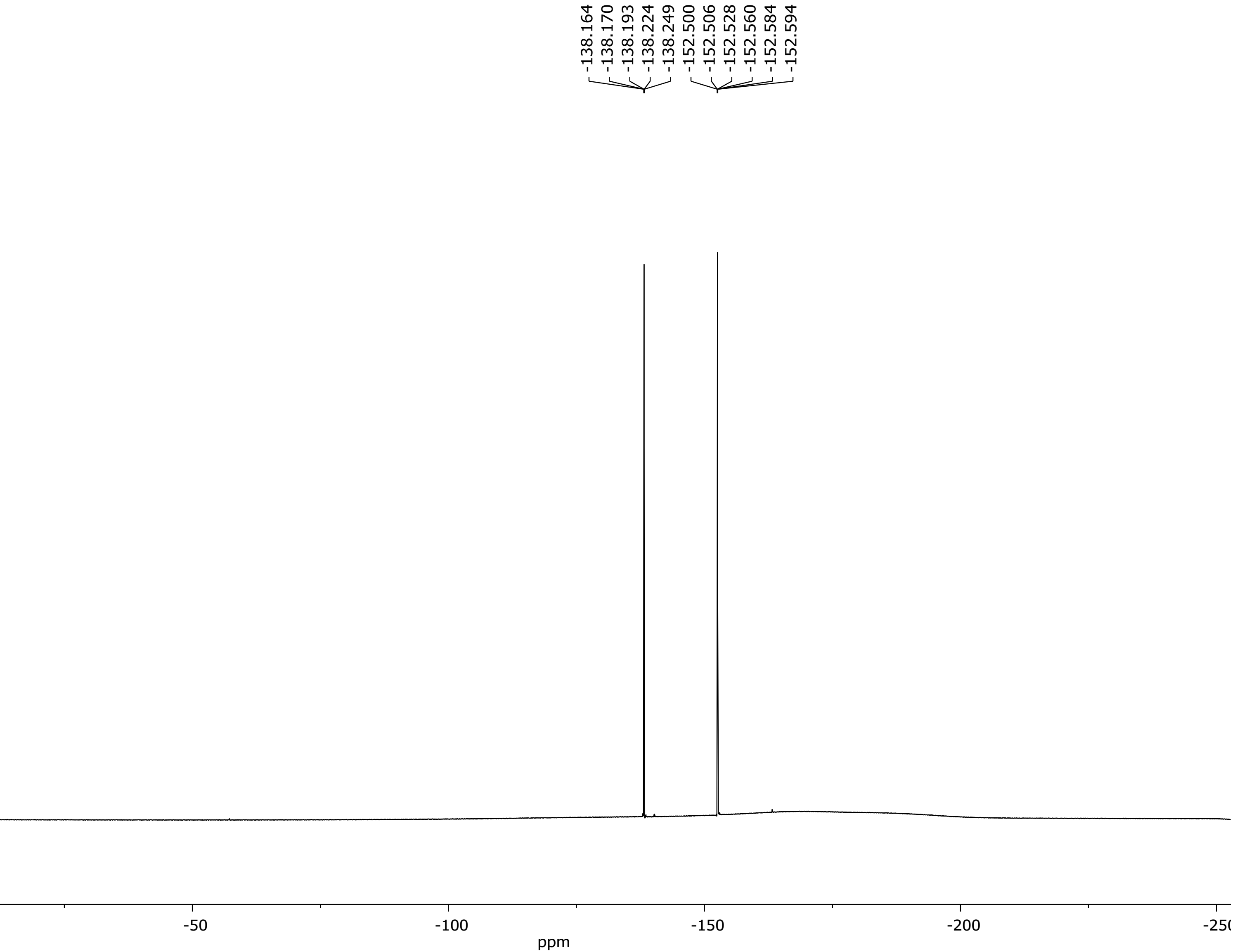

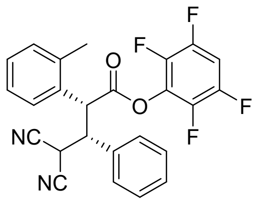

syn-**S19**

$^{13}C\{^{1}H\}$, $CDCl_3$, 126 MHz

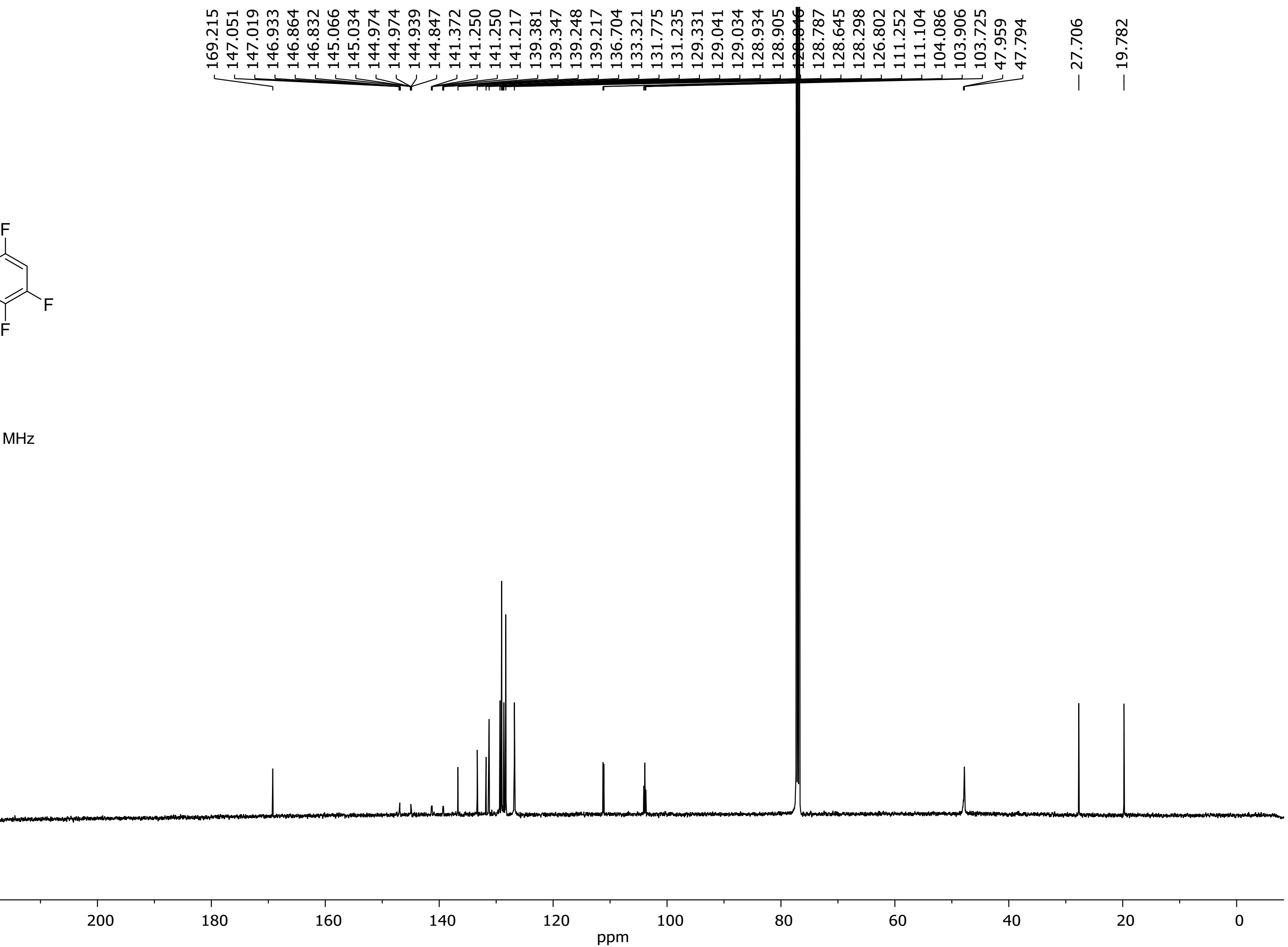

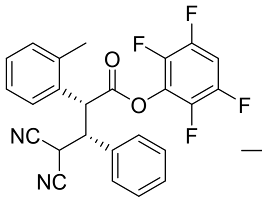

syn-**S19**
2D ^{1}H-^{1}H COSY, $CDCl_3$

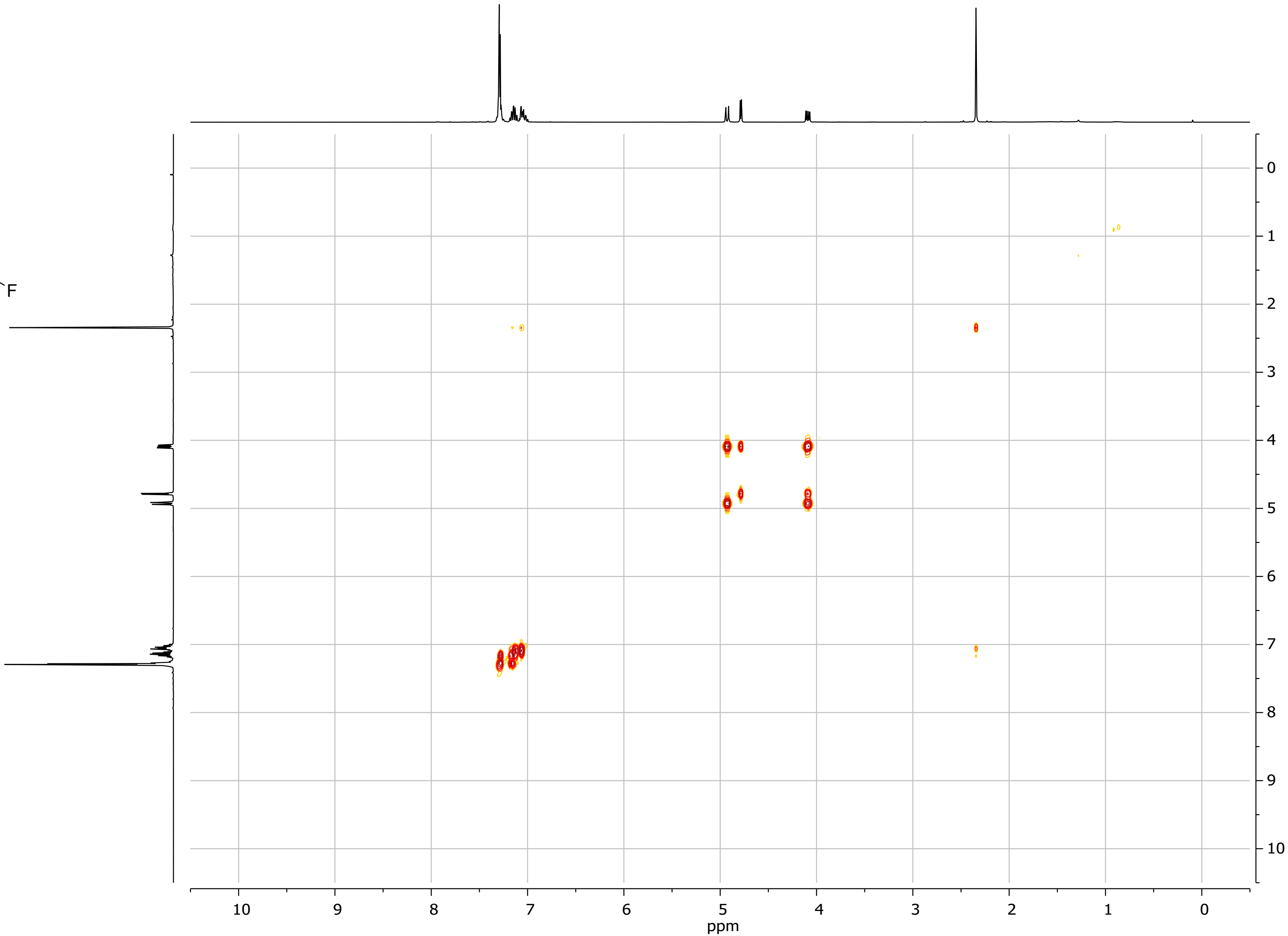

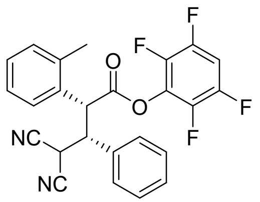

syn-**S19**
2D ^{1}H-^{13}C HSQC, $CDCl_3$

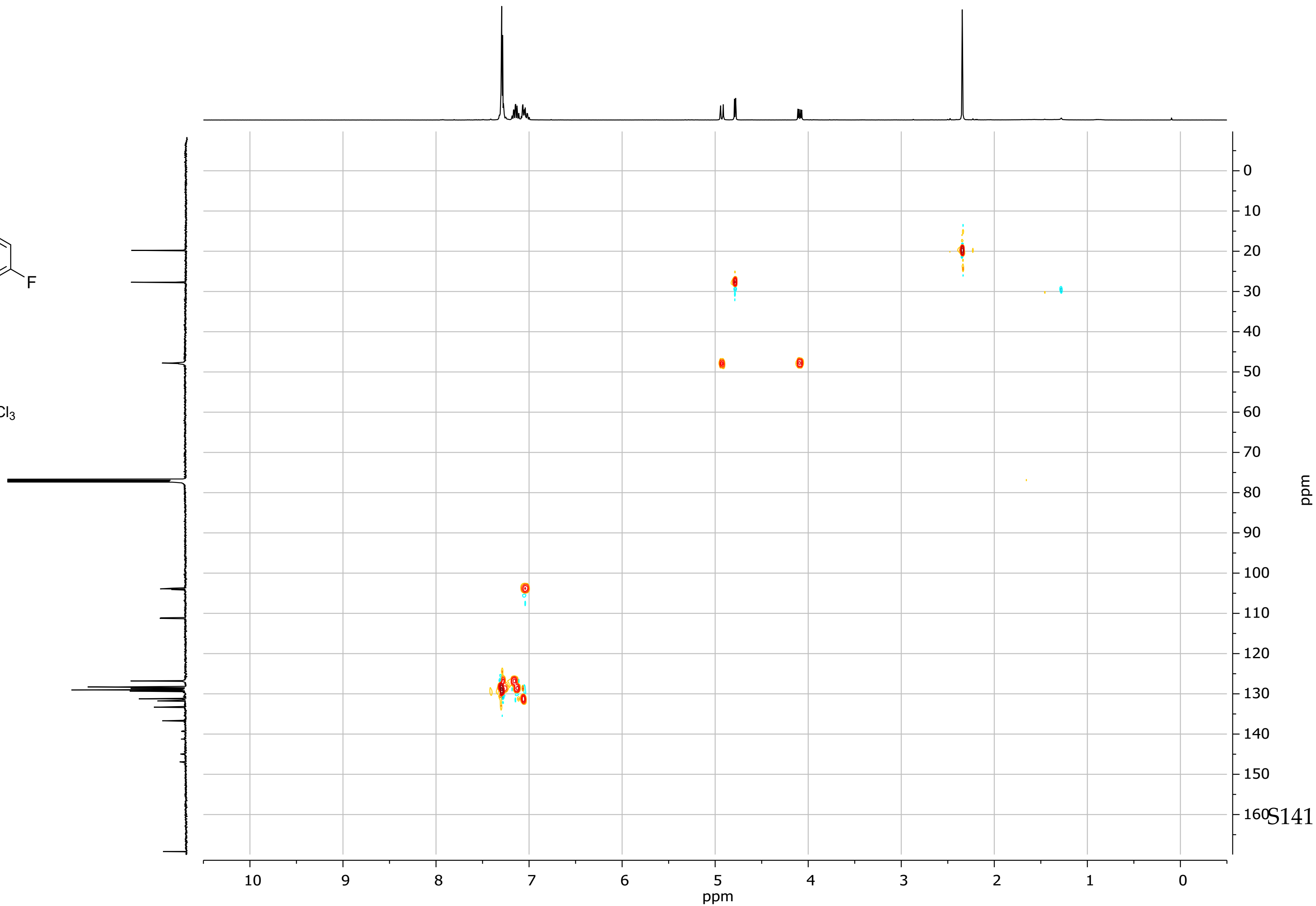

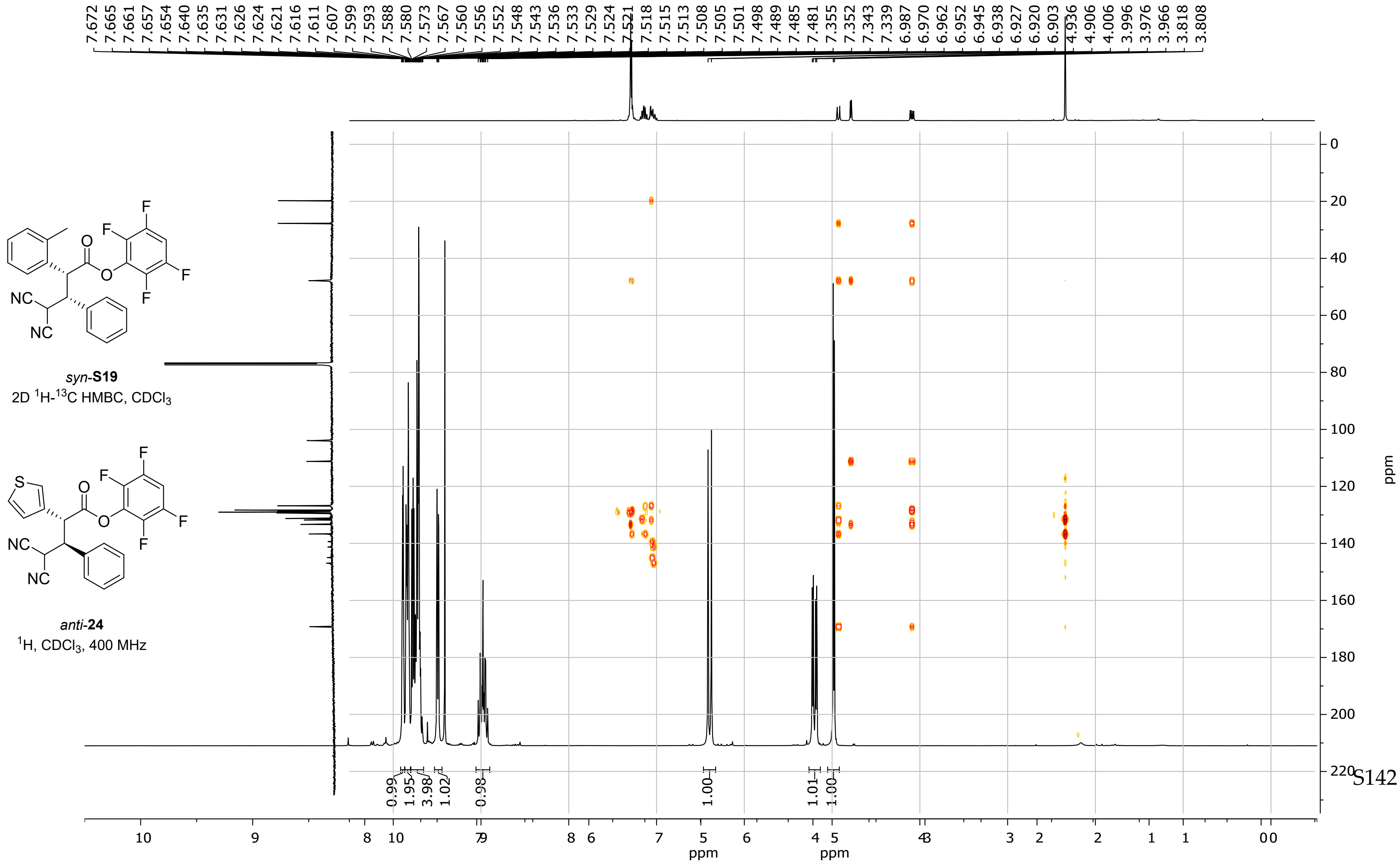

syn-S19
2D ¹H-¹³C HMBC, CDCl3
anti-24
¹H, CDCl3, 400 MHz
ppm

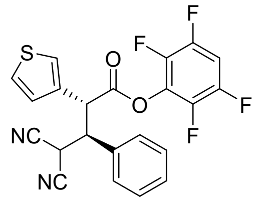

anti-**24**

$^{19}F\{^{1}H\}$, $CDCl_3$, 376 MHz

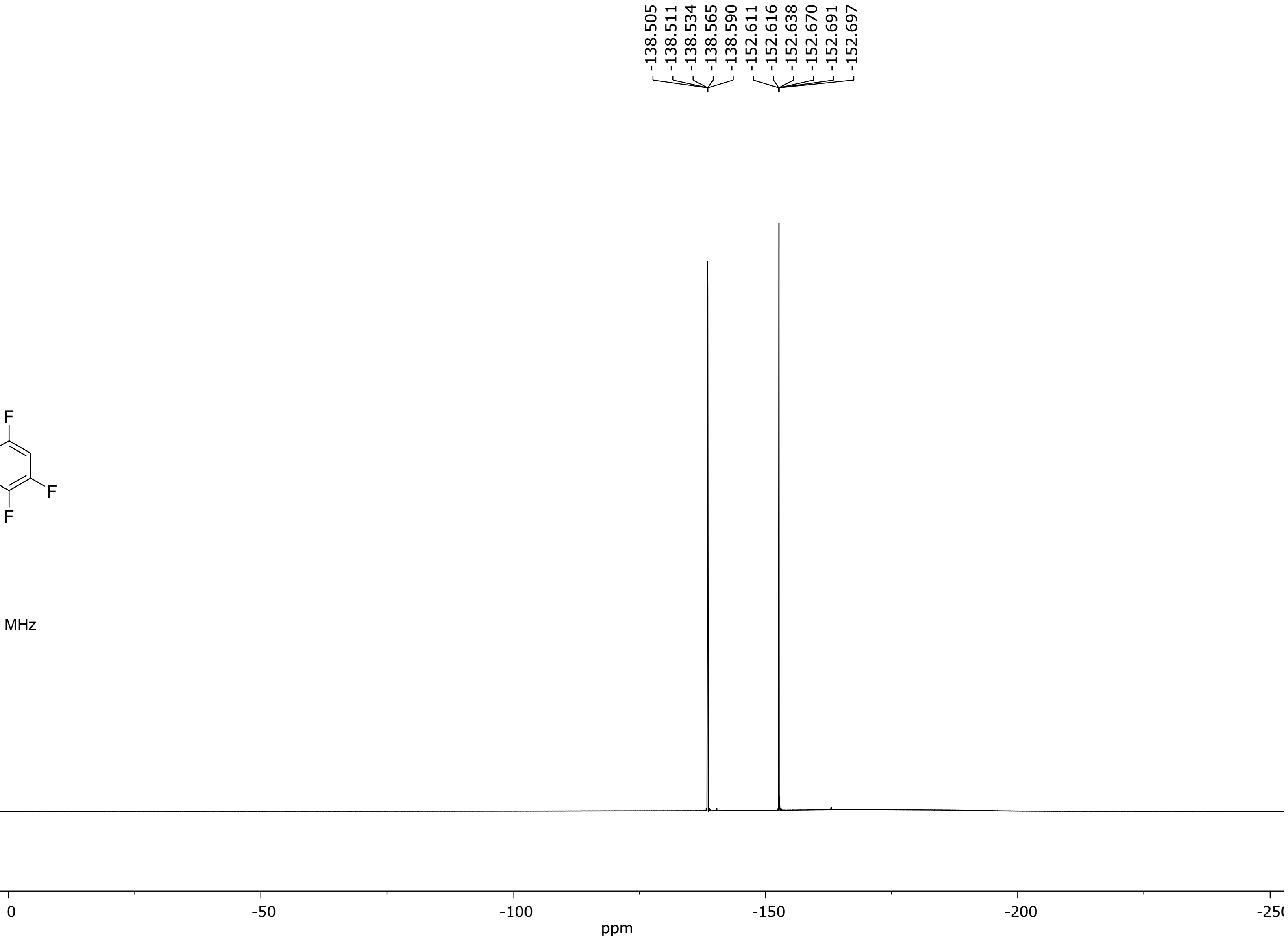

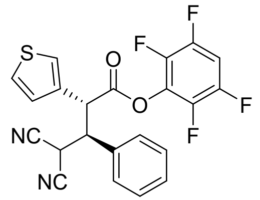

anti-**24**
$^{13}C\{^{1}H\}$, $CDCl_3$, 126 MHz

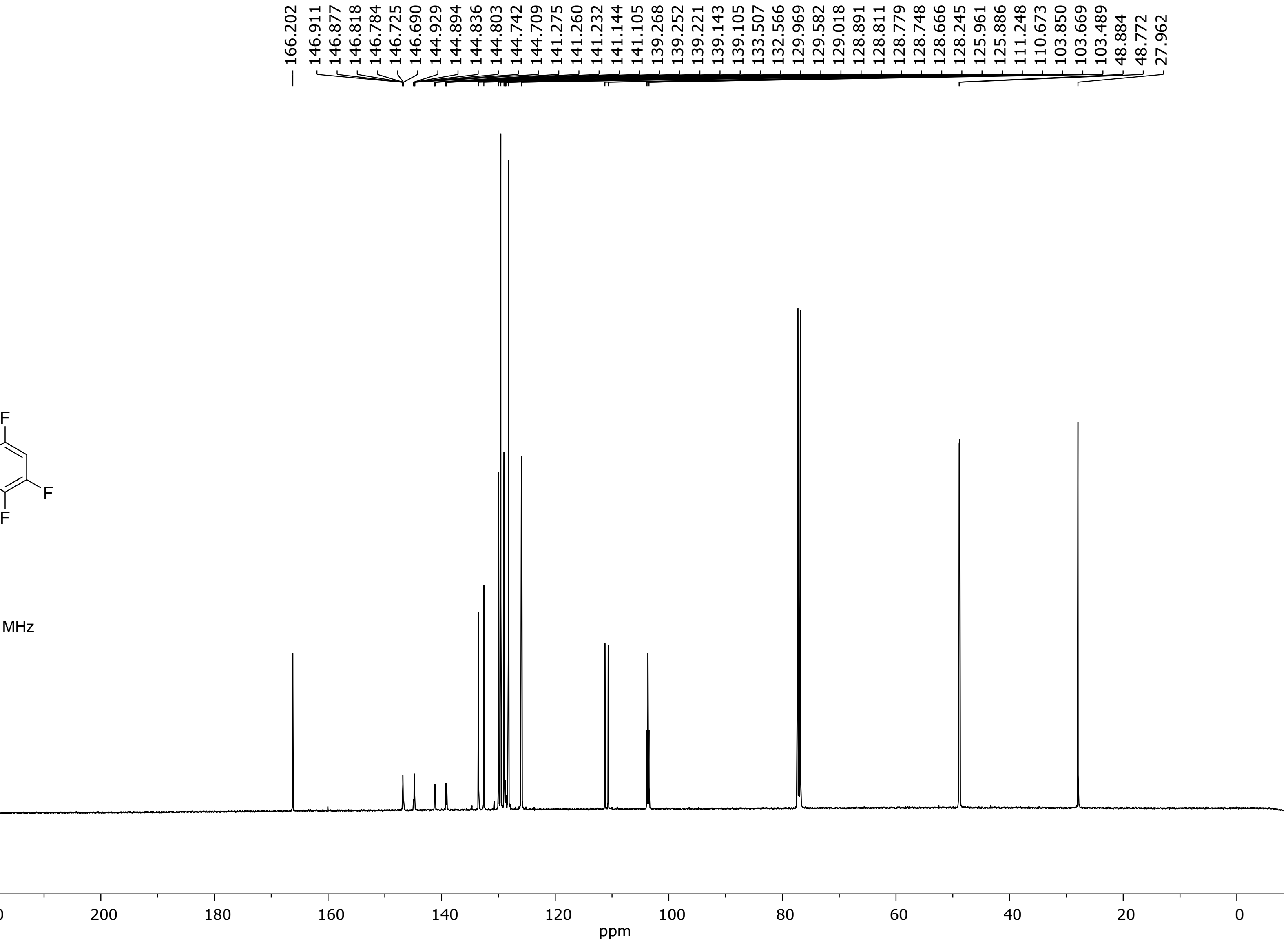

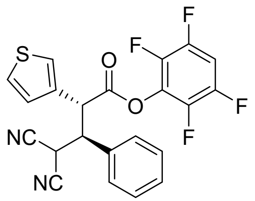

anti-**24**
2D ^{1}H-^{13}C HSQC, $CDCl_3$

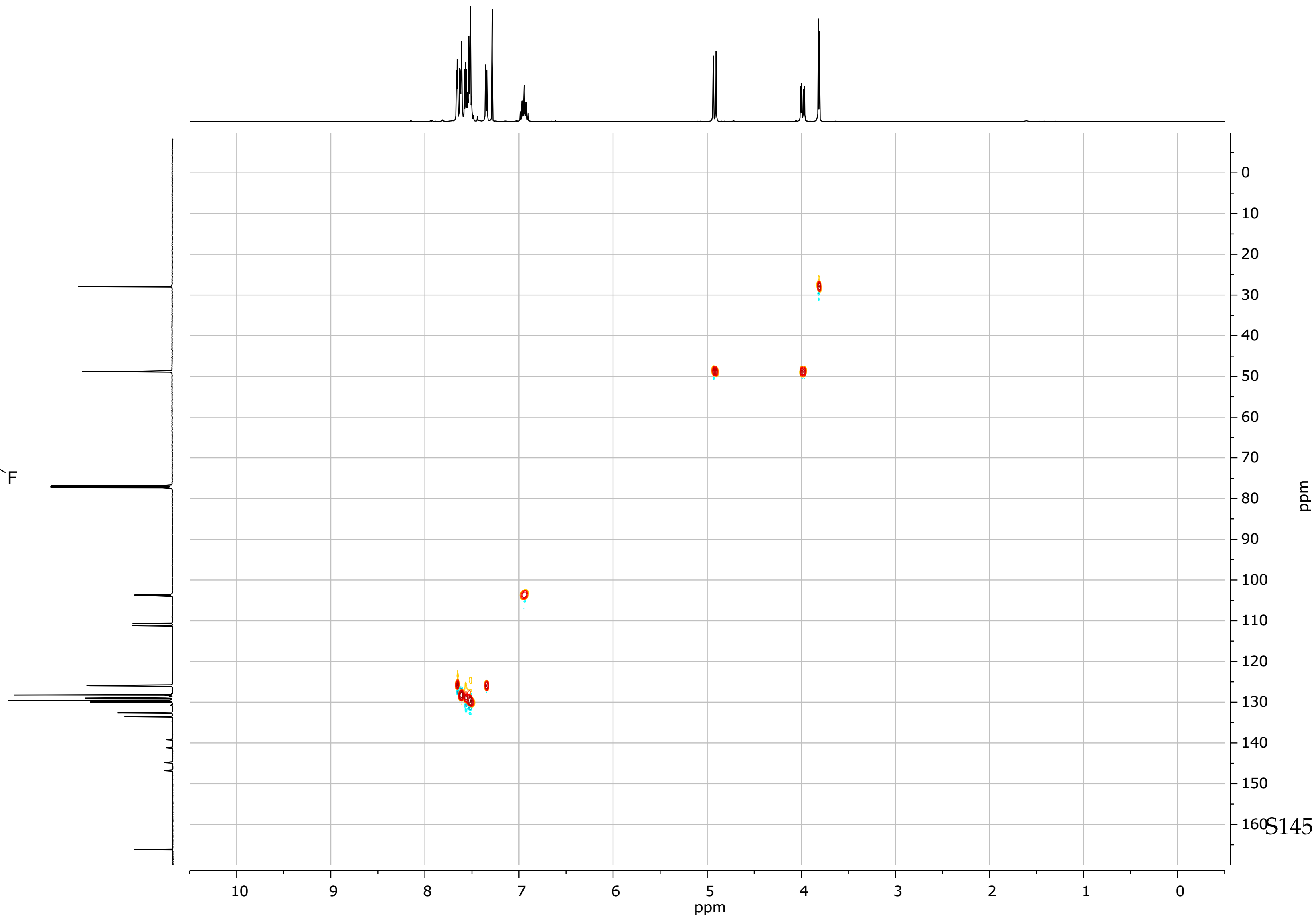

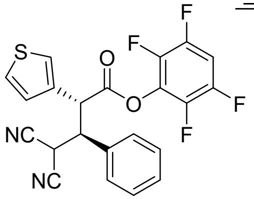

anti-**24**

2D ^{1}H-^{13}C HMBC, $CDCl_3$

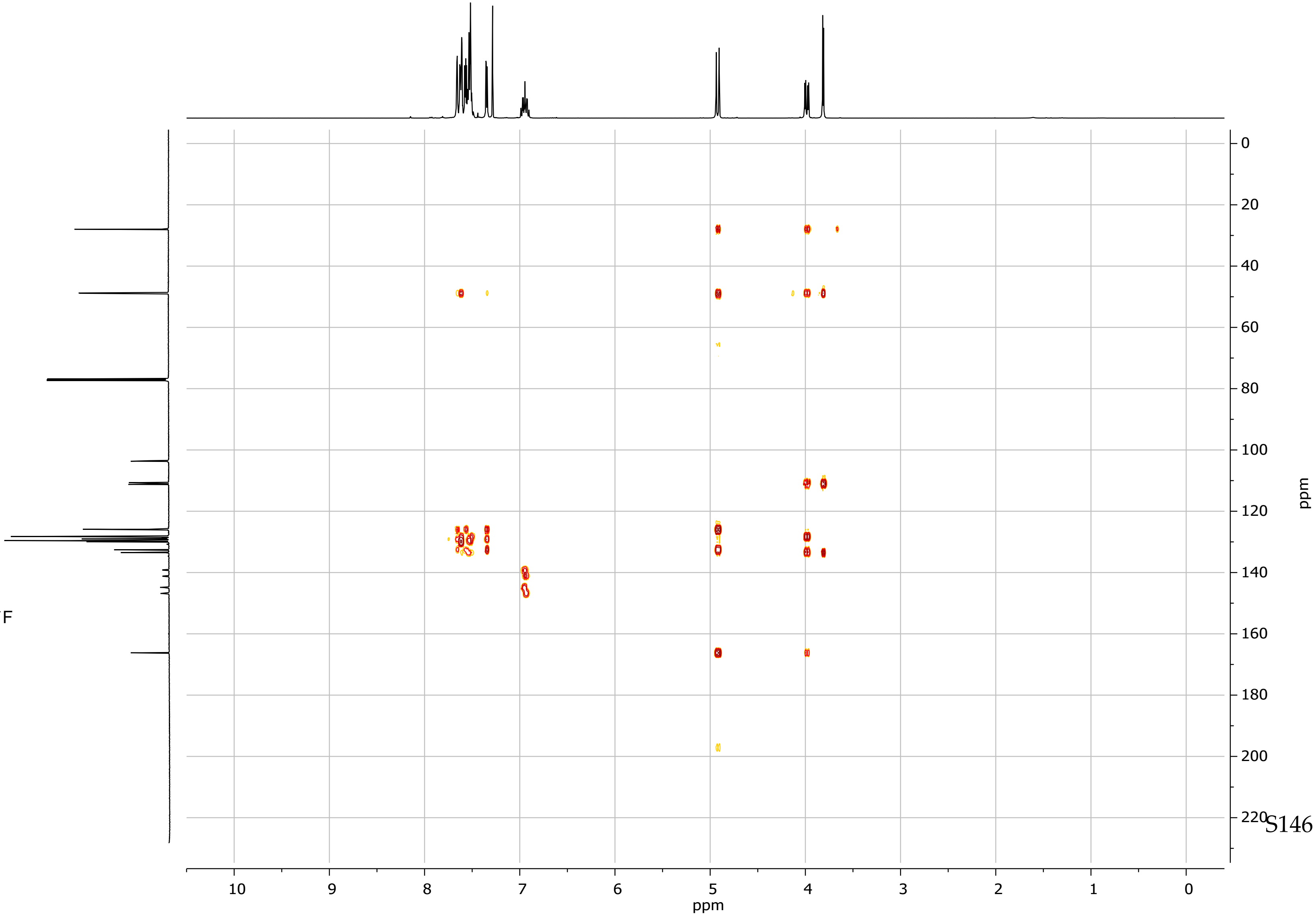

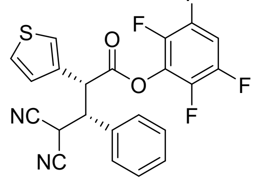

syn-**S20**

^1H, $CDCl_3$, 500 MHz

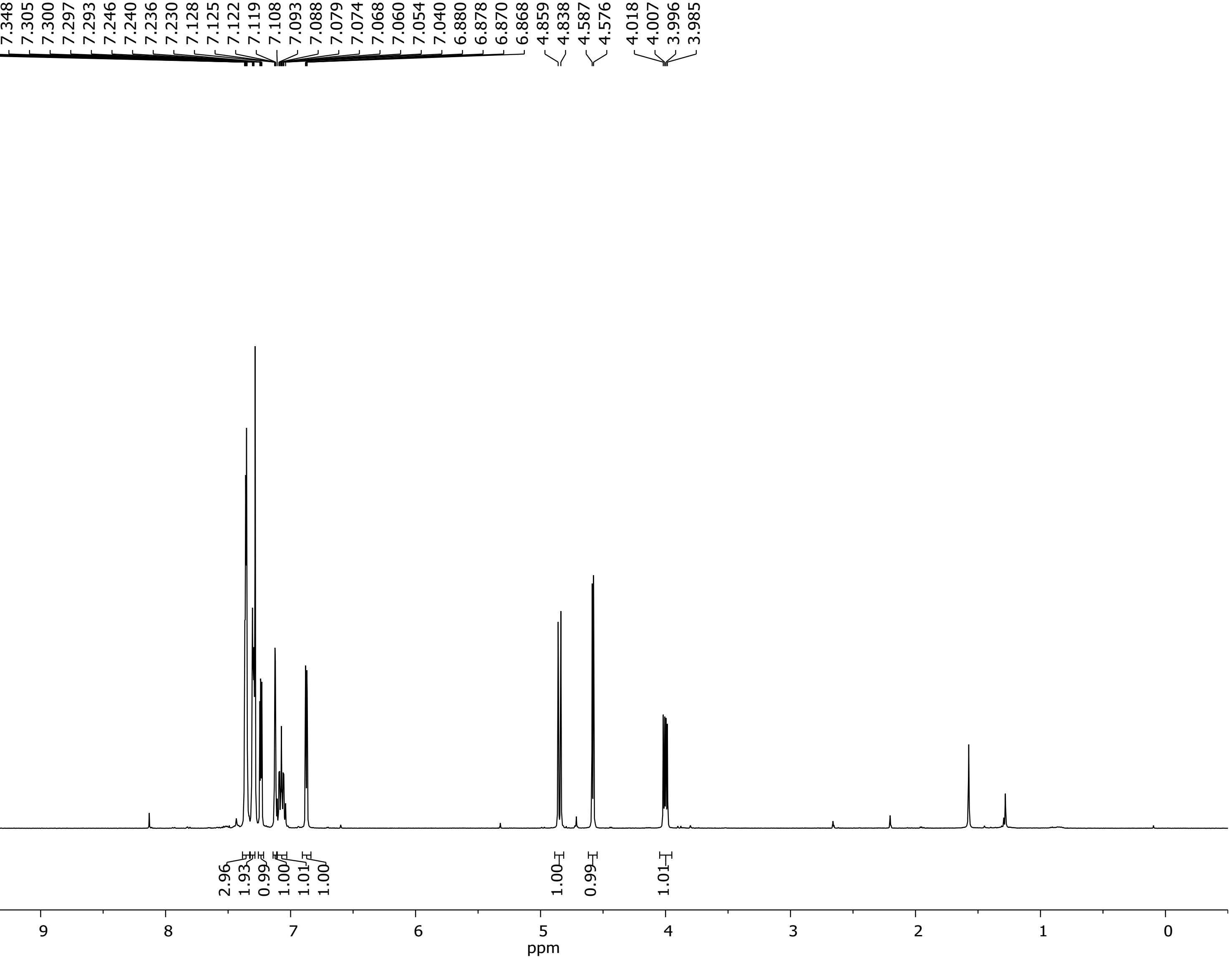

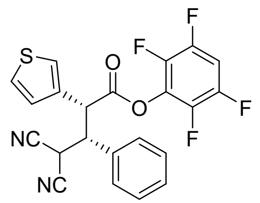

syn-**S20**

$^{19}F\{^{1}H\}$, $CDCl_3$, 376 MHz

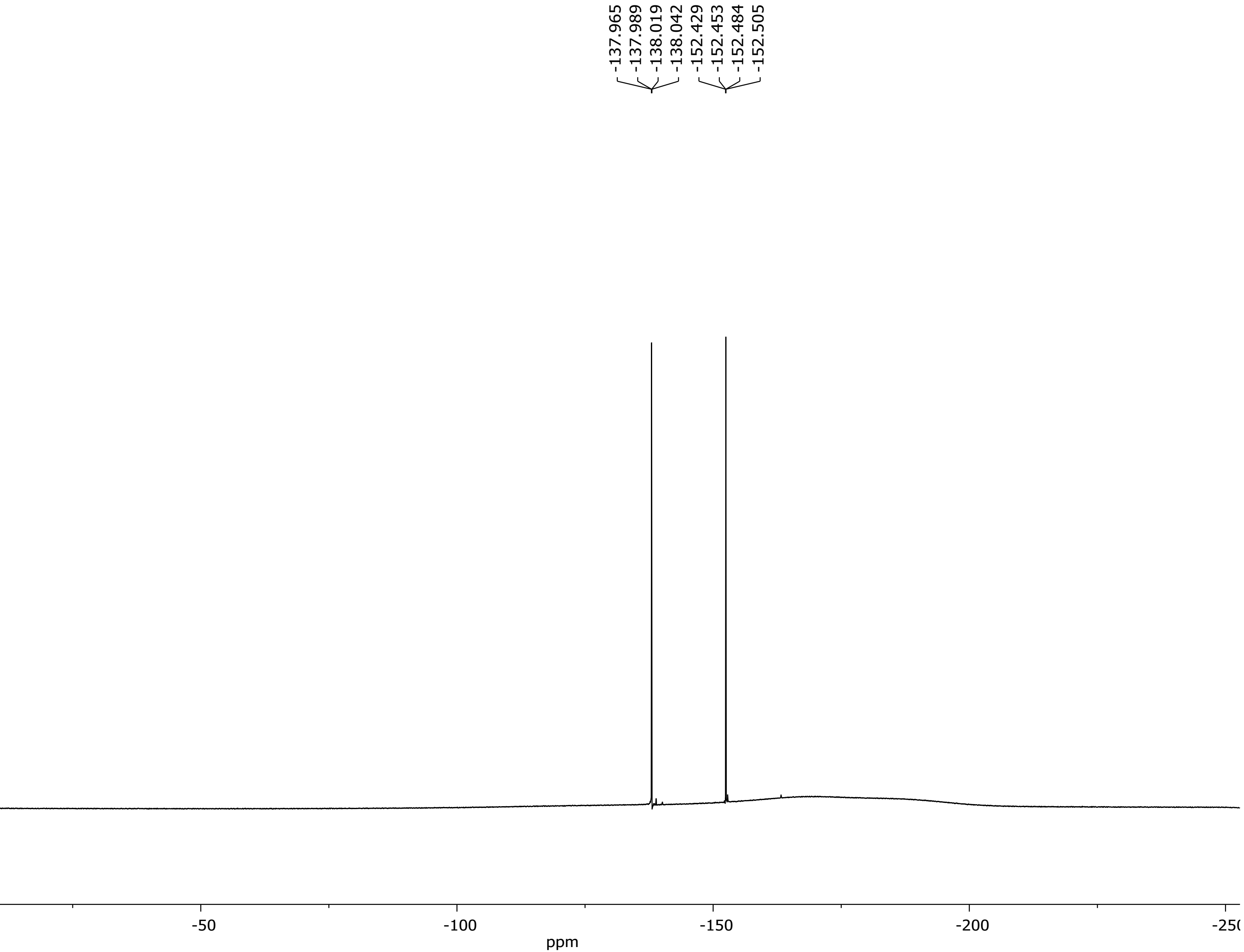

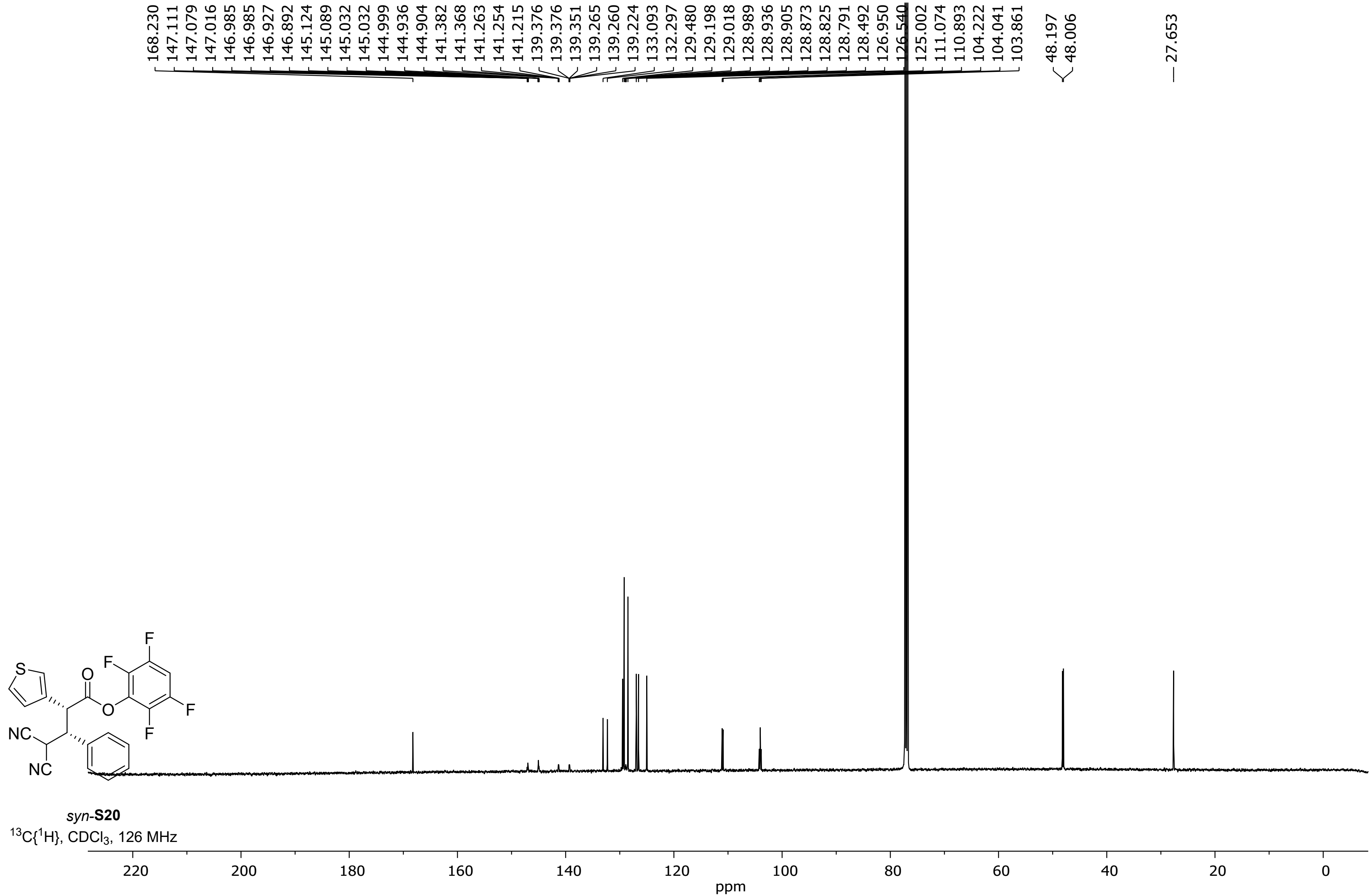

syn-**S20**

$^{13}C\{^{1}H\}$, $CDCl_3$, 126 MHz

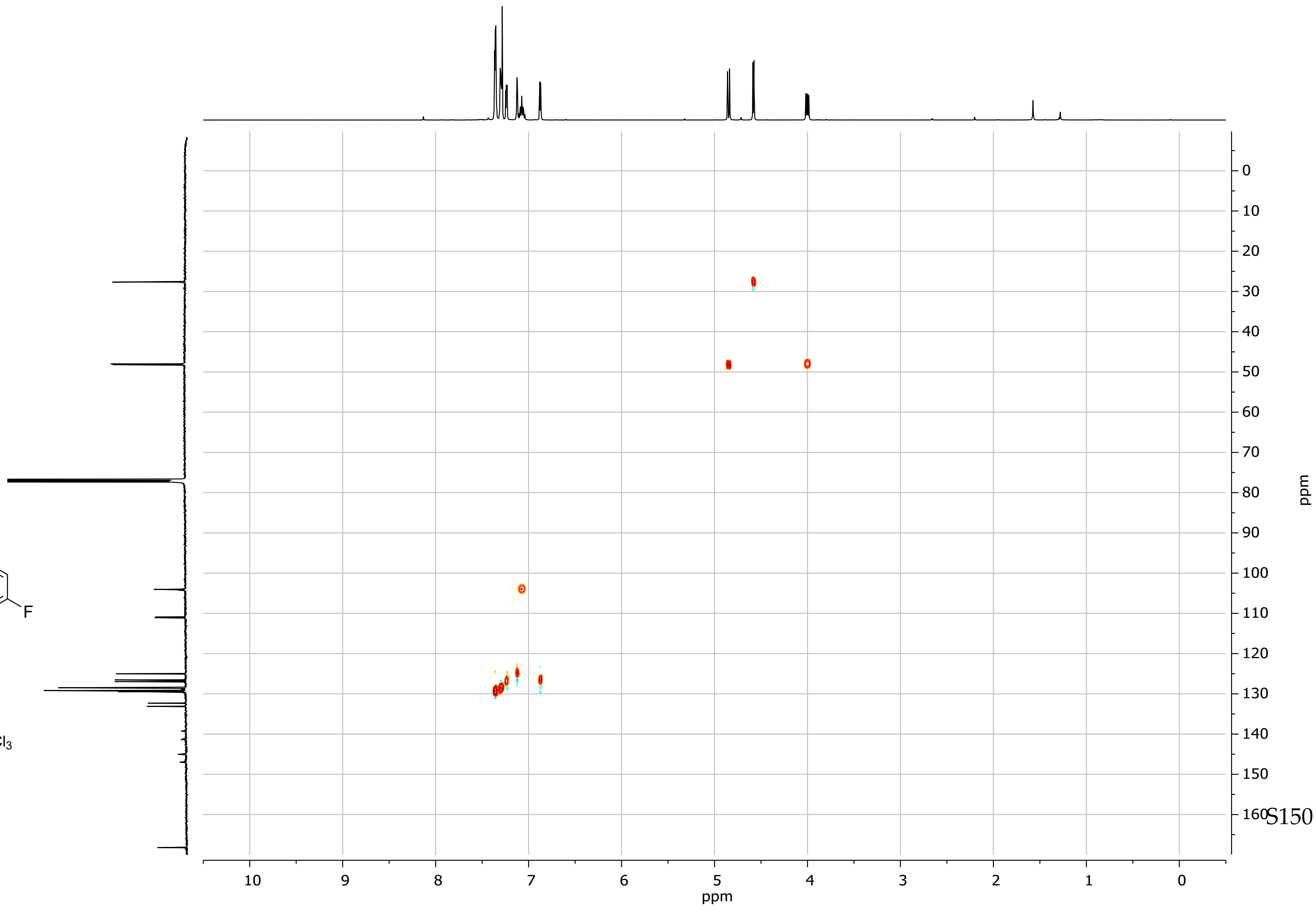

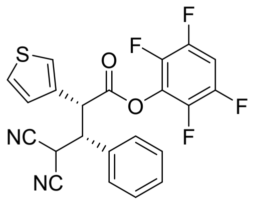

syn-**S20**
2D ^{1}H-^{13}C HSQC, $CDCl_3$

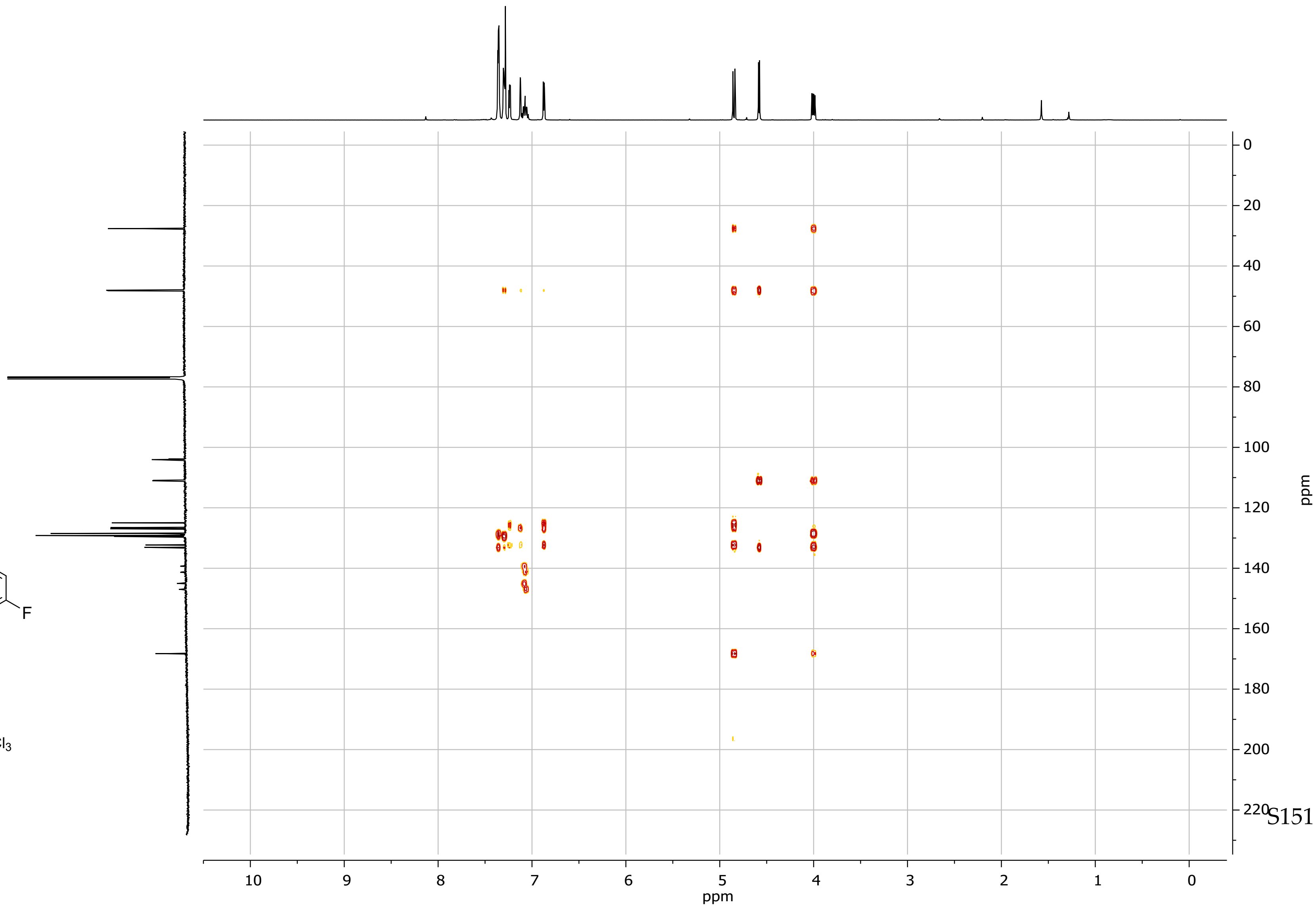

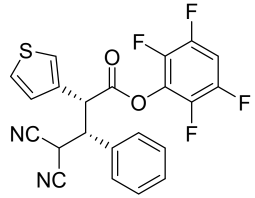

syn-**S20**
2D ^{1}H-^{13}C HMBC, $CDCl_3$

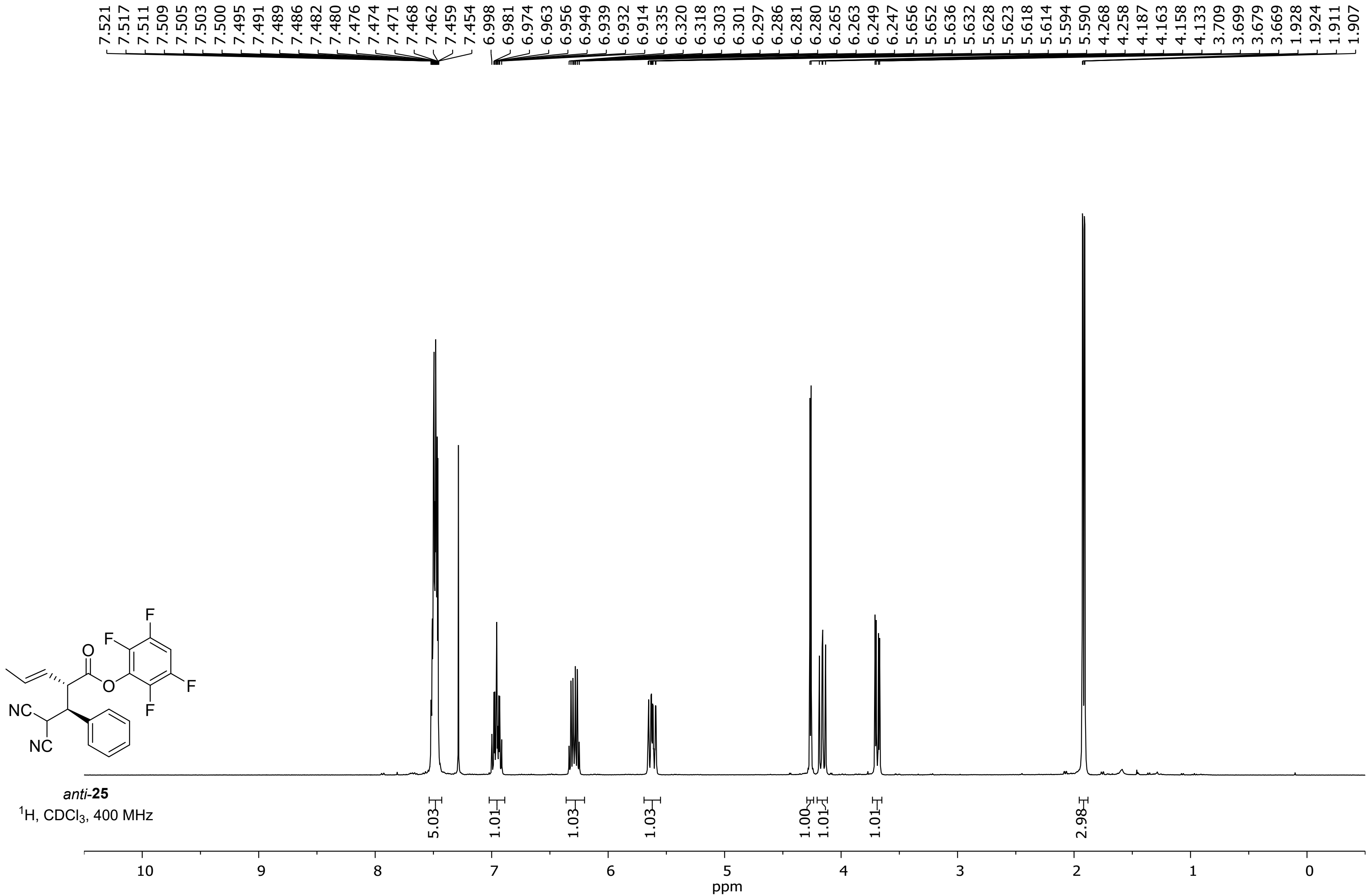

anti-**25**

^{1}H, $CDCl_3$, 400 MHz

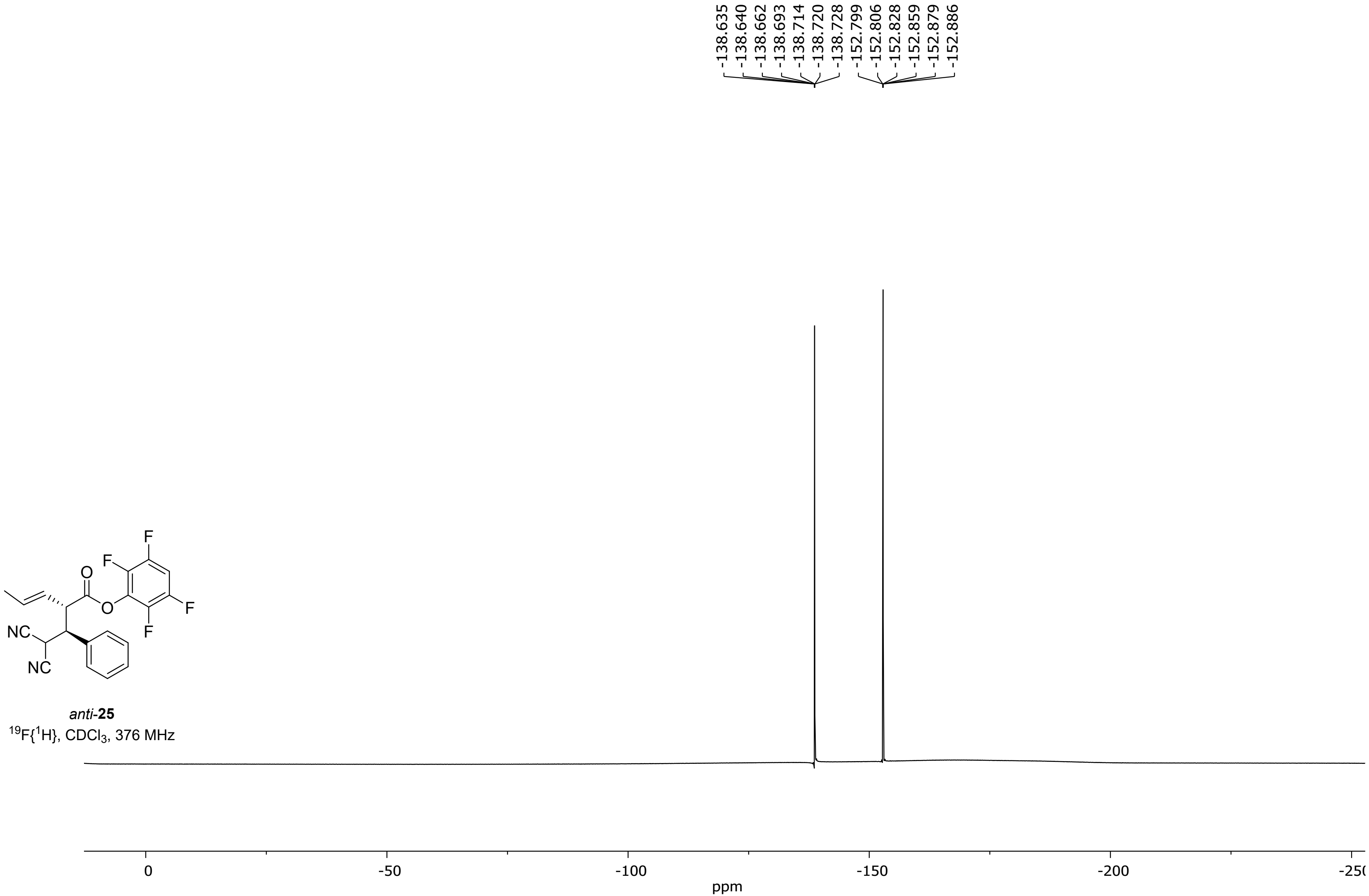

anti-**25**

$^{19}F\{^{1}H\}$, $CDCl_3$, 376 MHz

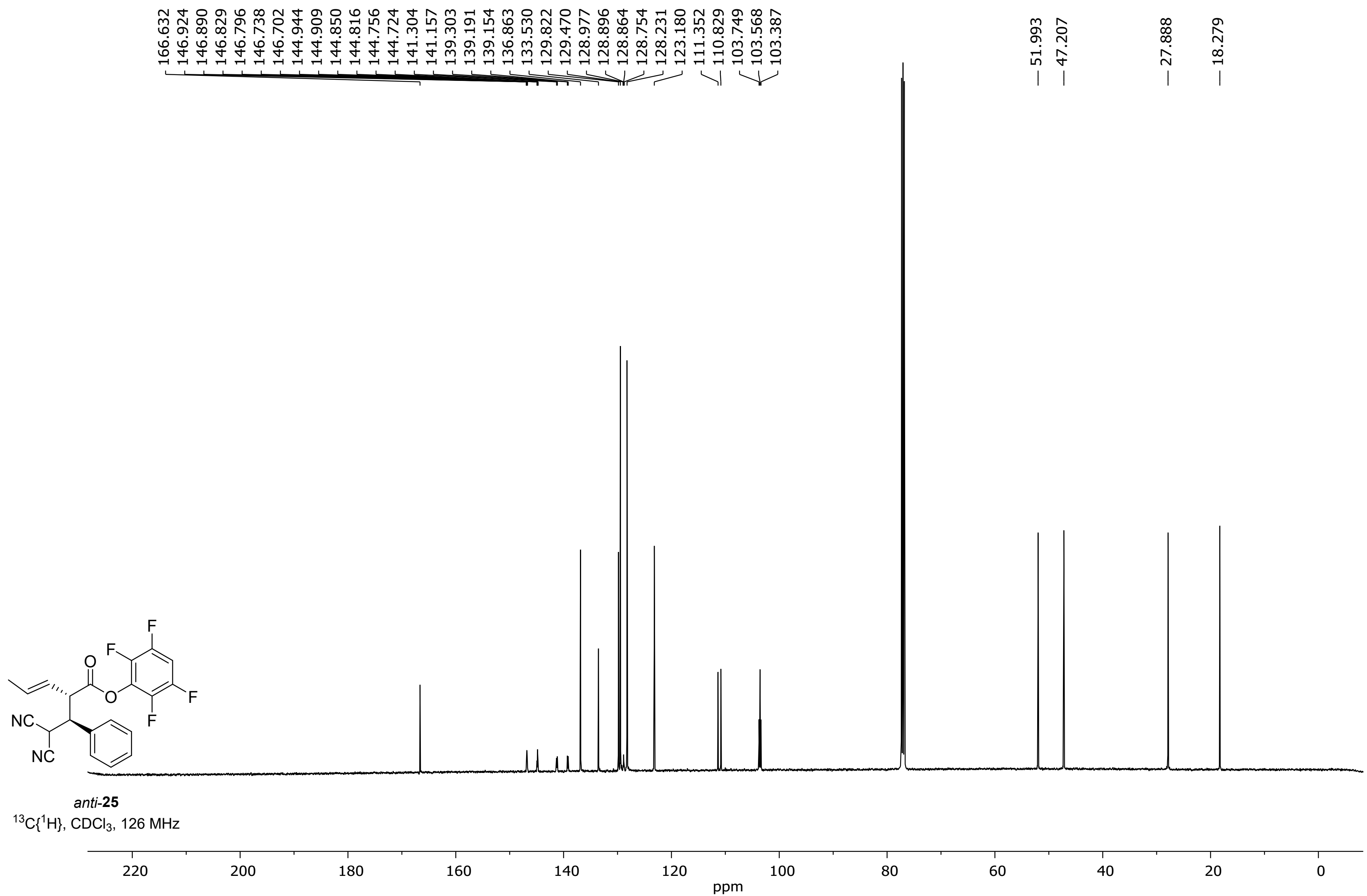

anti-**25**

$^{13}C\{^{1}H\}$, $CDCl_3$, 126 MHz

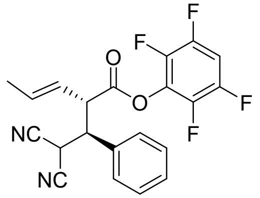

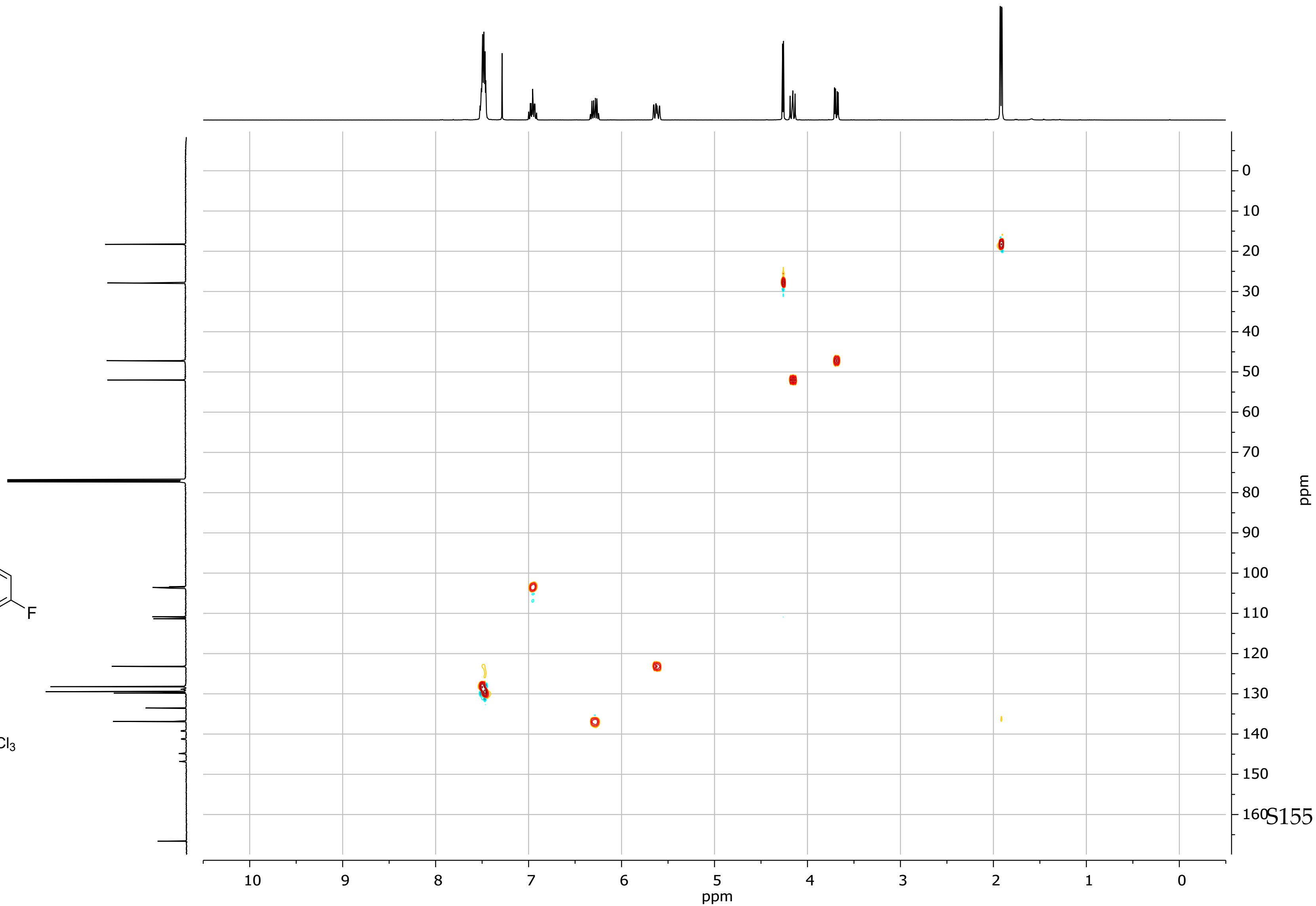

anti-**25**

2D ^{1}H-^{13}C HSQC, $CDCl_3$

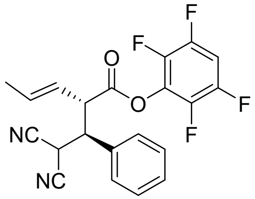

anti-**25**
2D ^{1}H-^{13}C HMBC, $CDCl_3$

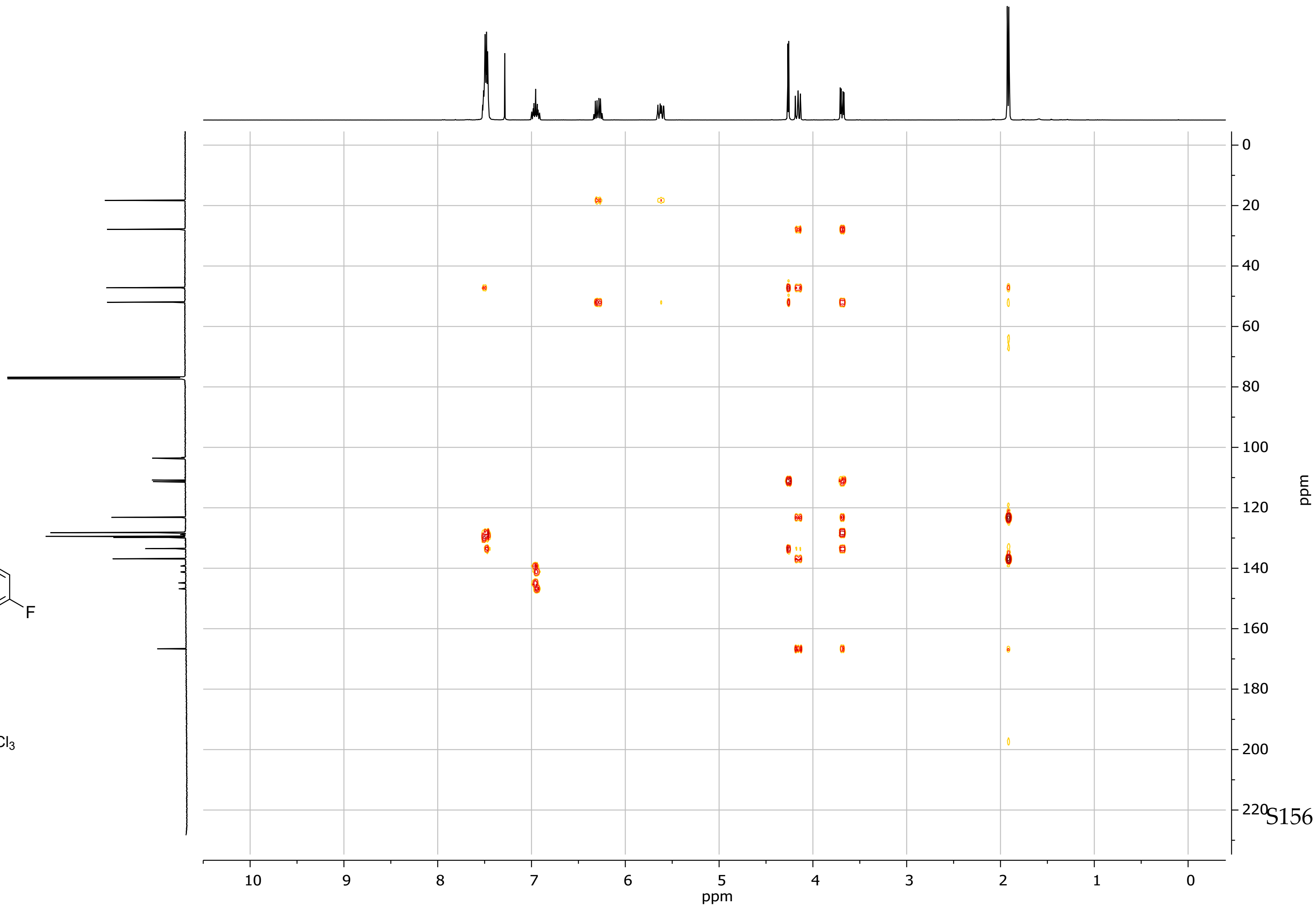

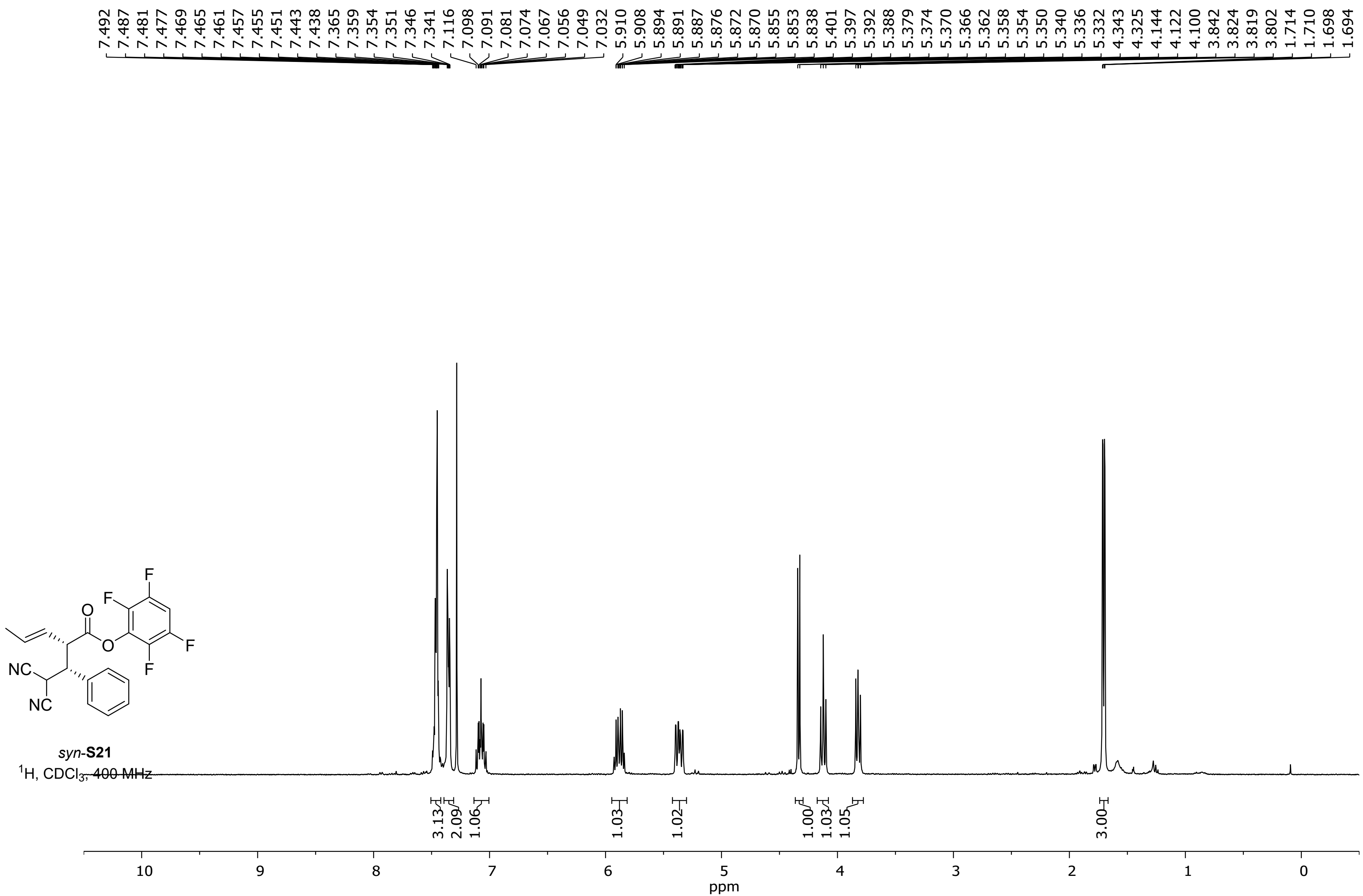

syn-**S21**

^{1}H, $CDCl_3$, 400 MHz

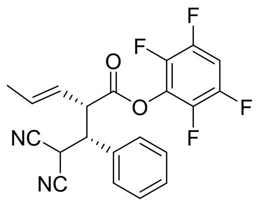

syn-**S21**

$^{19}F\{^{1}H\}$, $CDCl_3$, 376 MHz

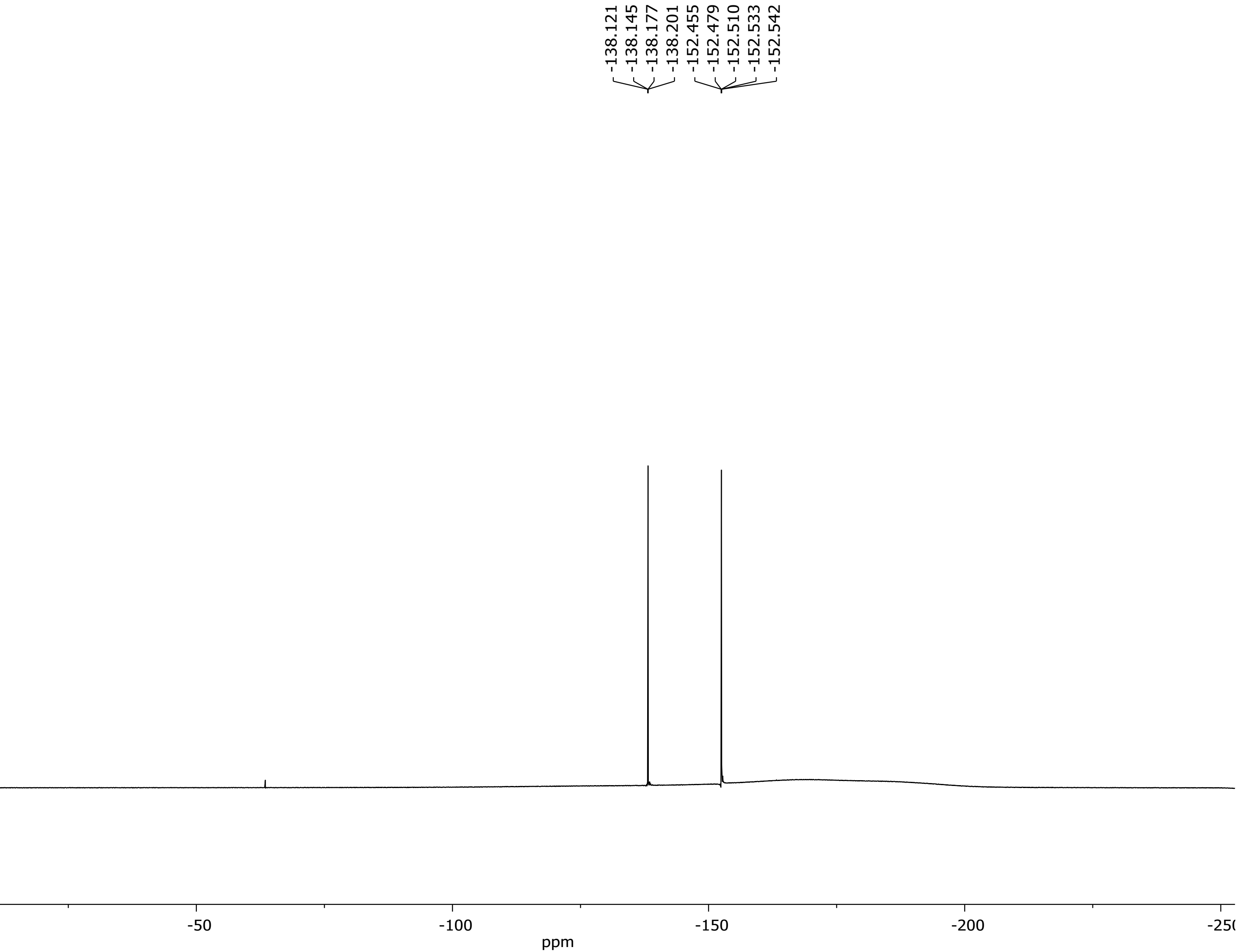

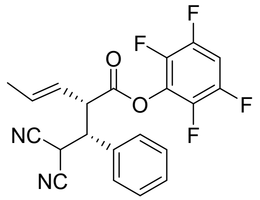

syn-**S21**

$^{13}C\{^{1}H\}$, $CDCl_3$, 126 MHz

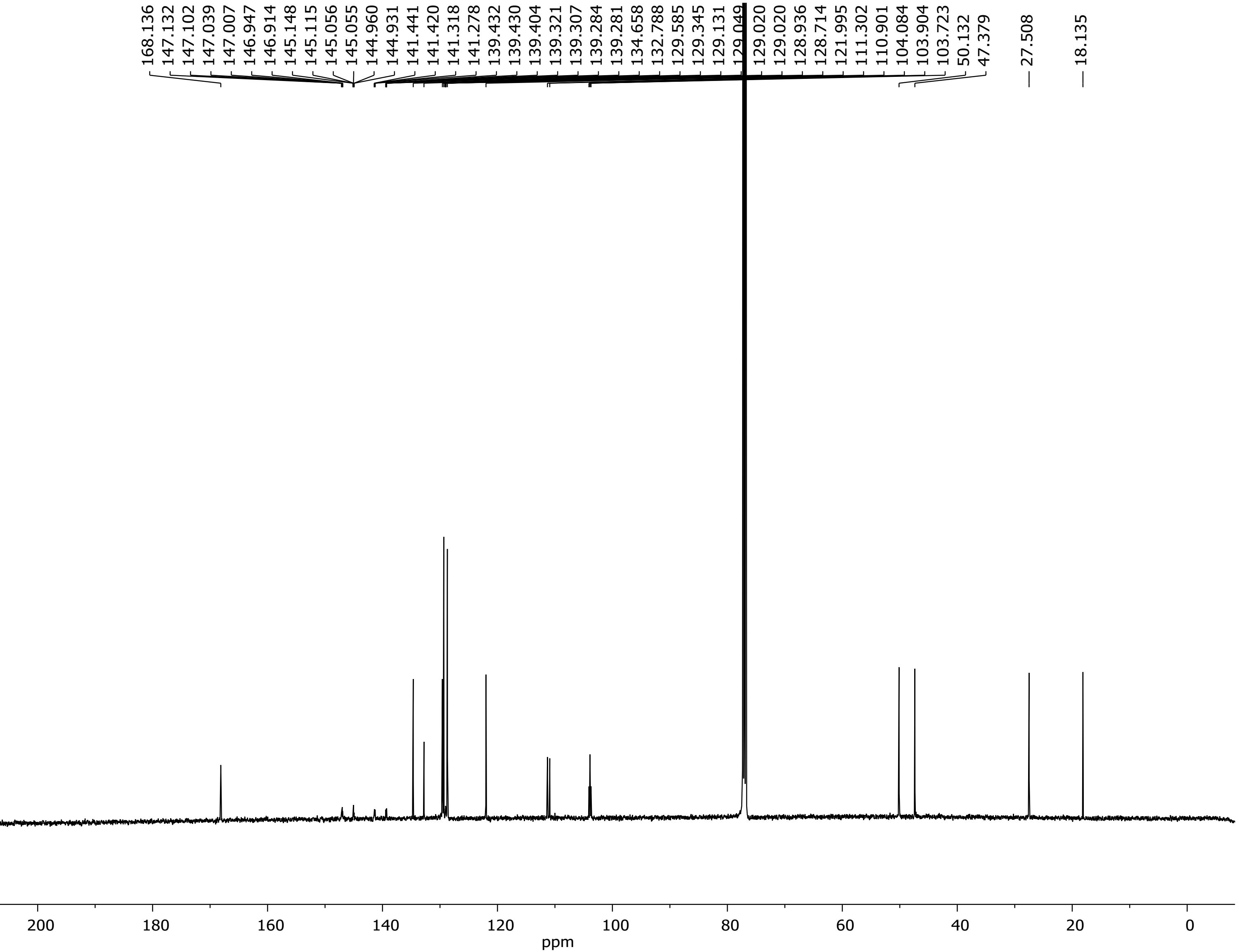

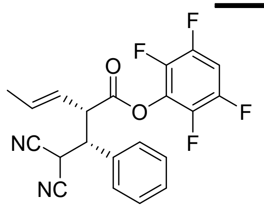

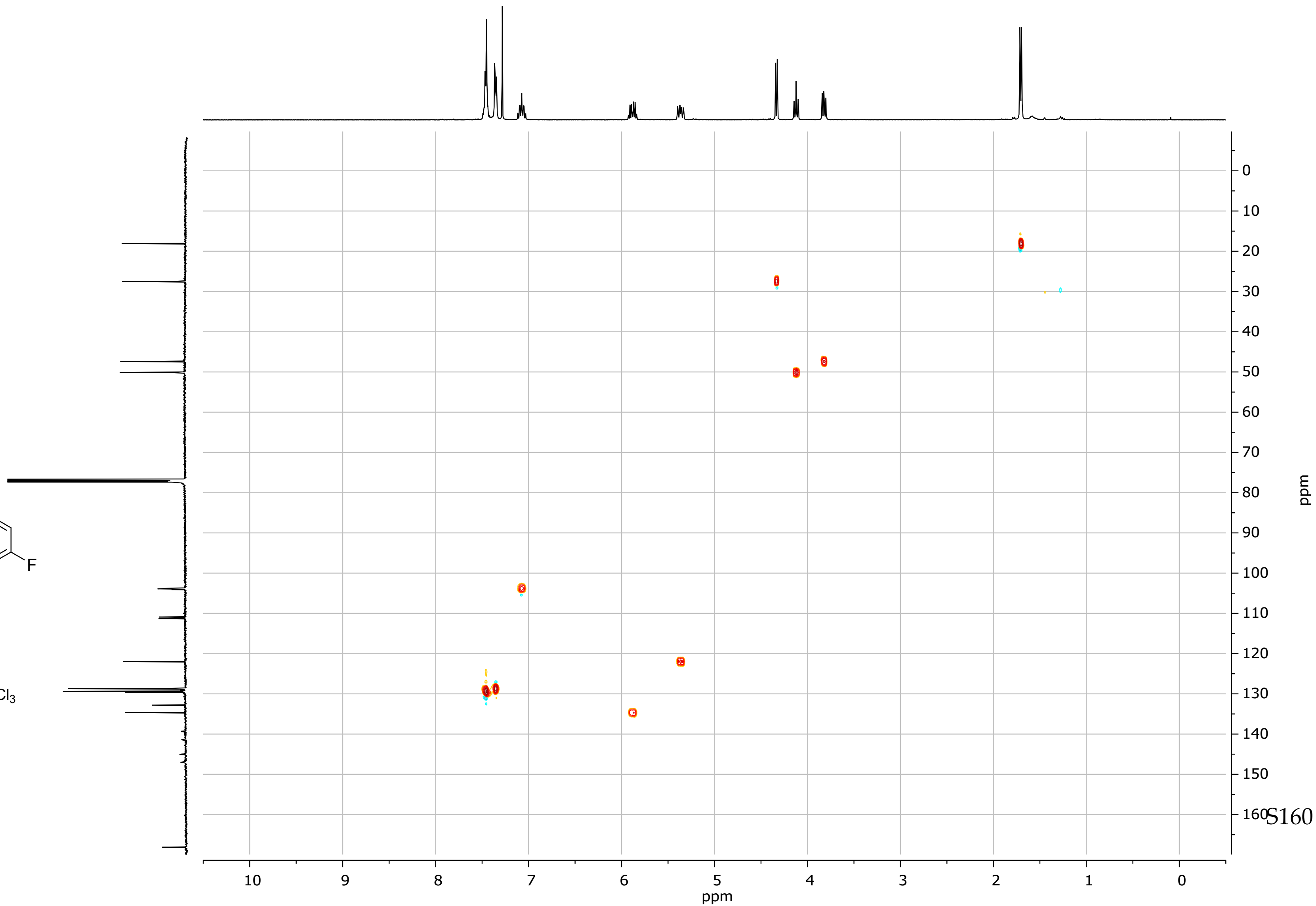

syn-**S21**
2D ^{1}H-^{13}C HSQC, $CDCl_3$

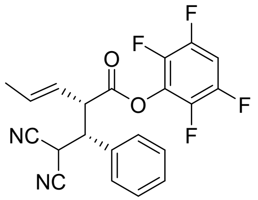

syn-**S21**

2D ^{1}H-^{13}C HMBC, $CDCl_3$

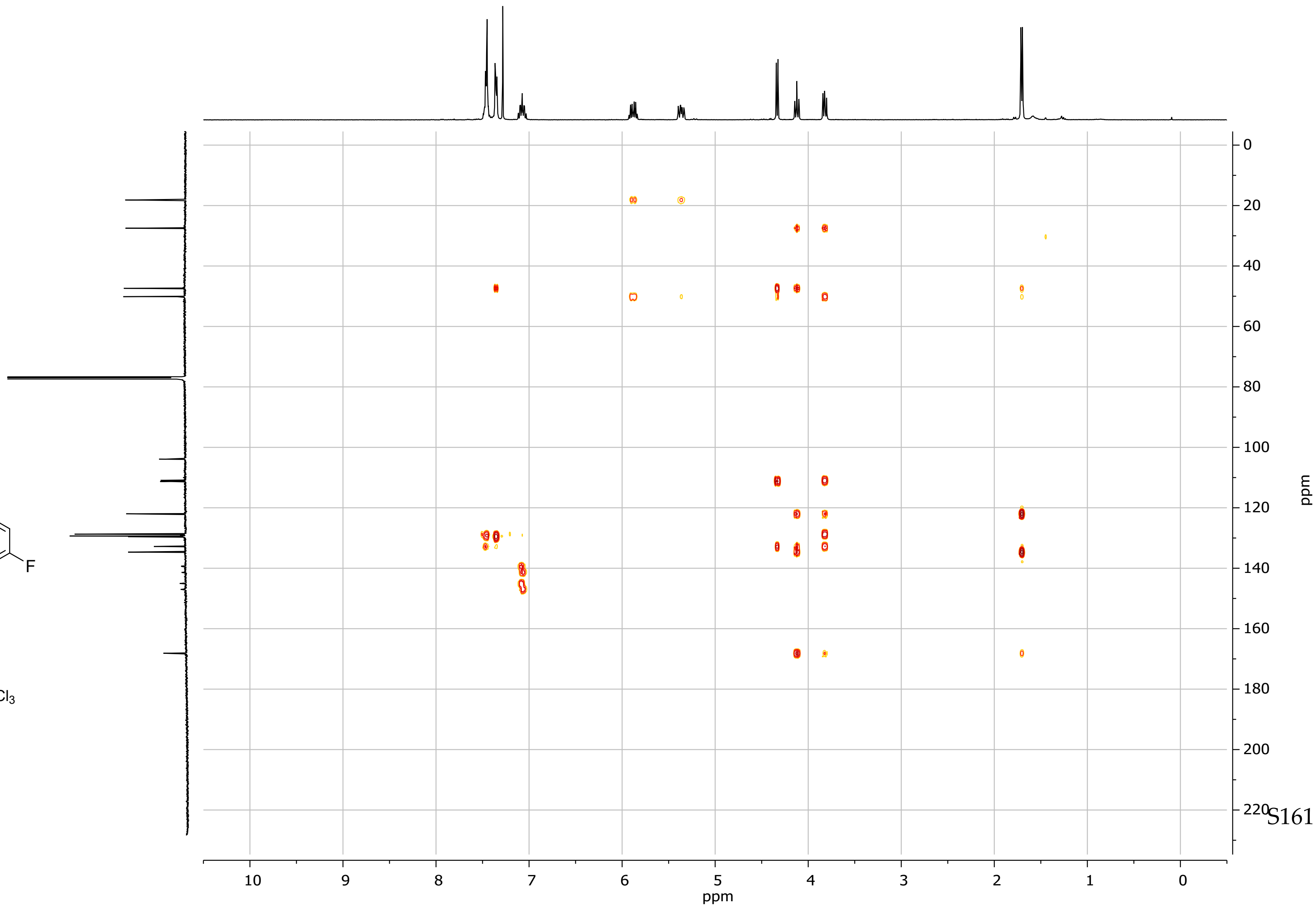

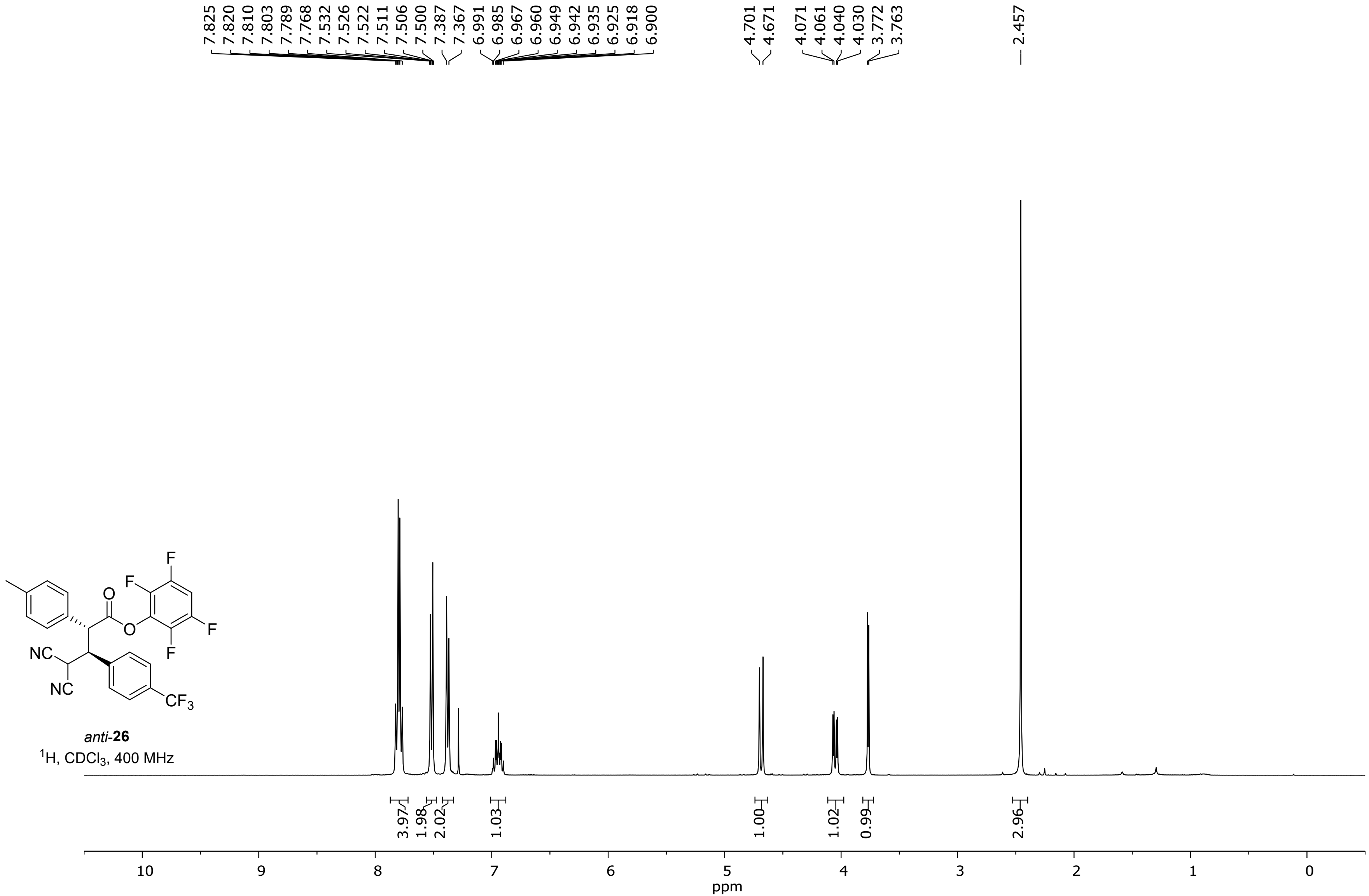

anti-**26**

^1H, $CDCl_3$, 400 MHz

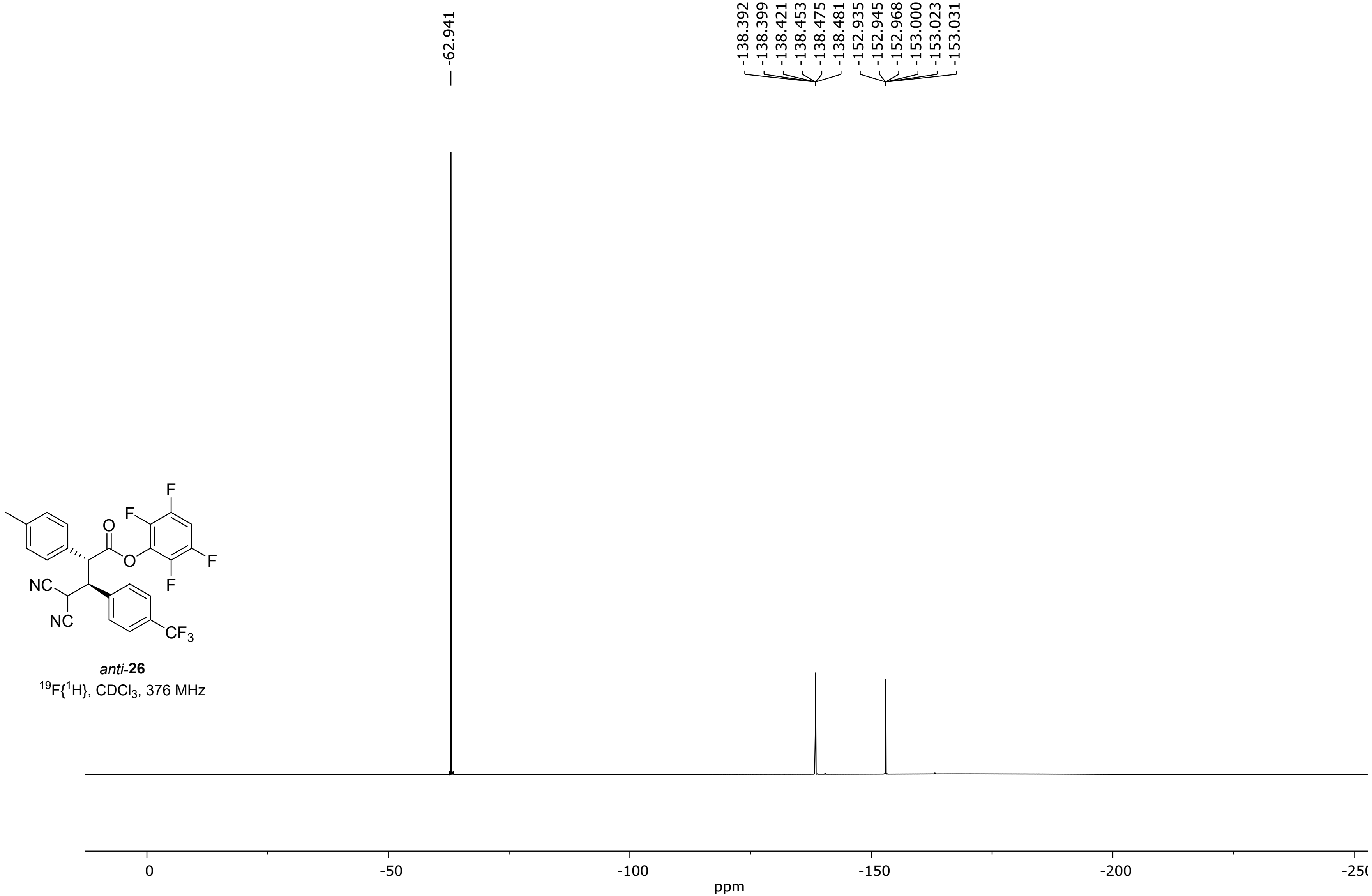

anti-**26**
$^{19}F\{^1H\}$, $CDCl_3$, 376 MHz

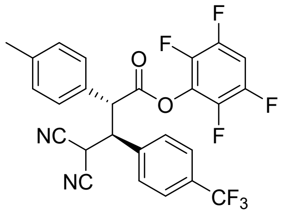

anti-**26**

$^{13}C\{^{1}H\}$, $CDCl_3$, 126 MHz

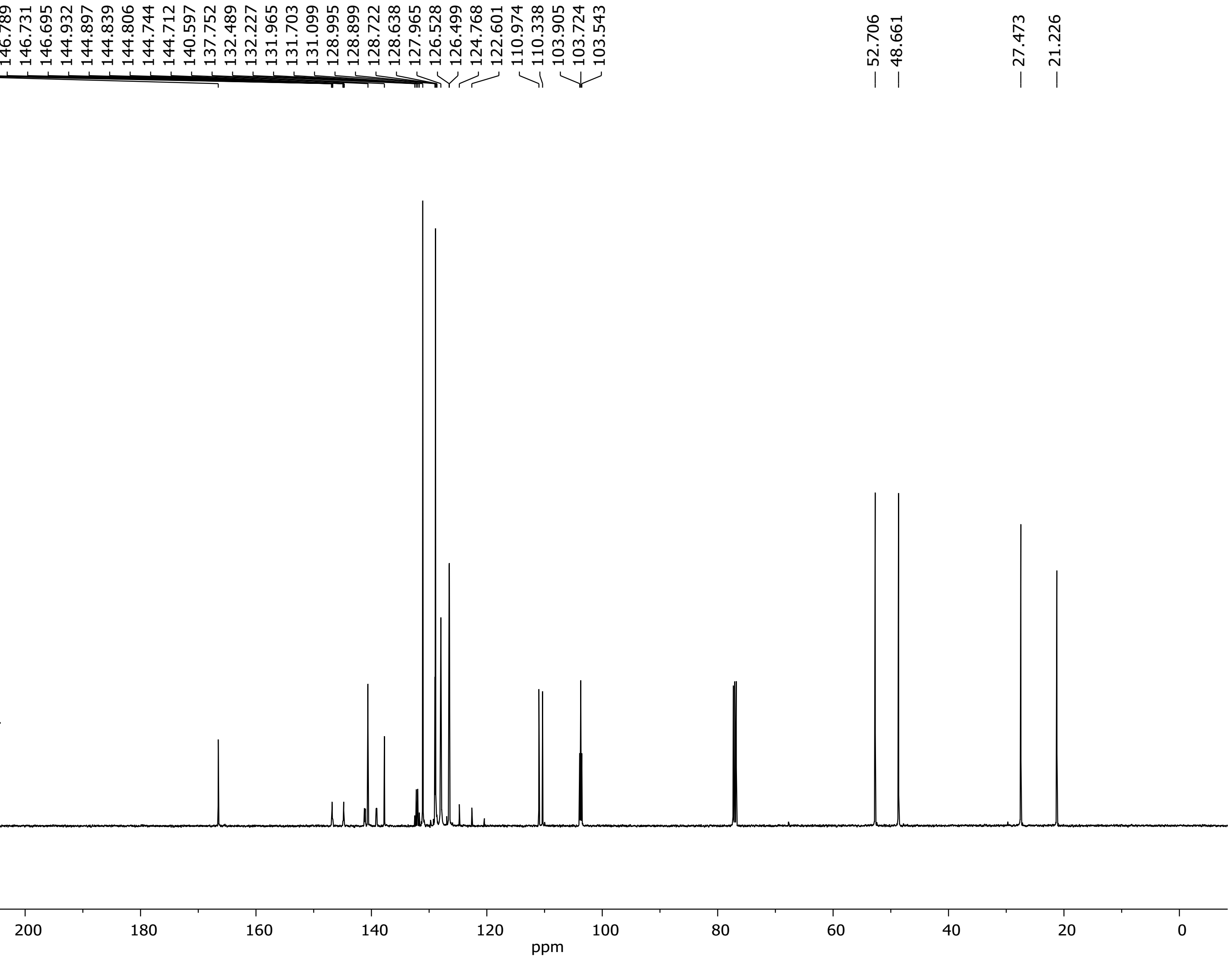

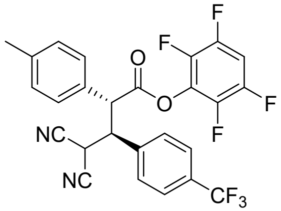

anti-**26**
2D ^{1}H-^{1}H COSY, $CDCl_3$

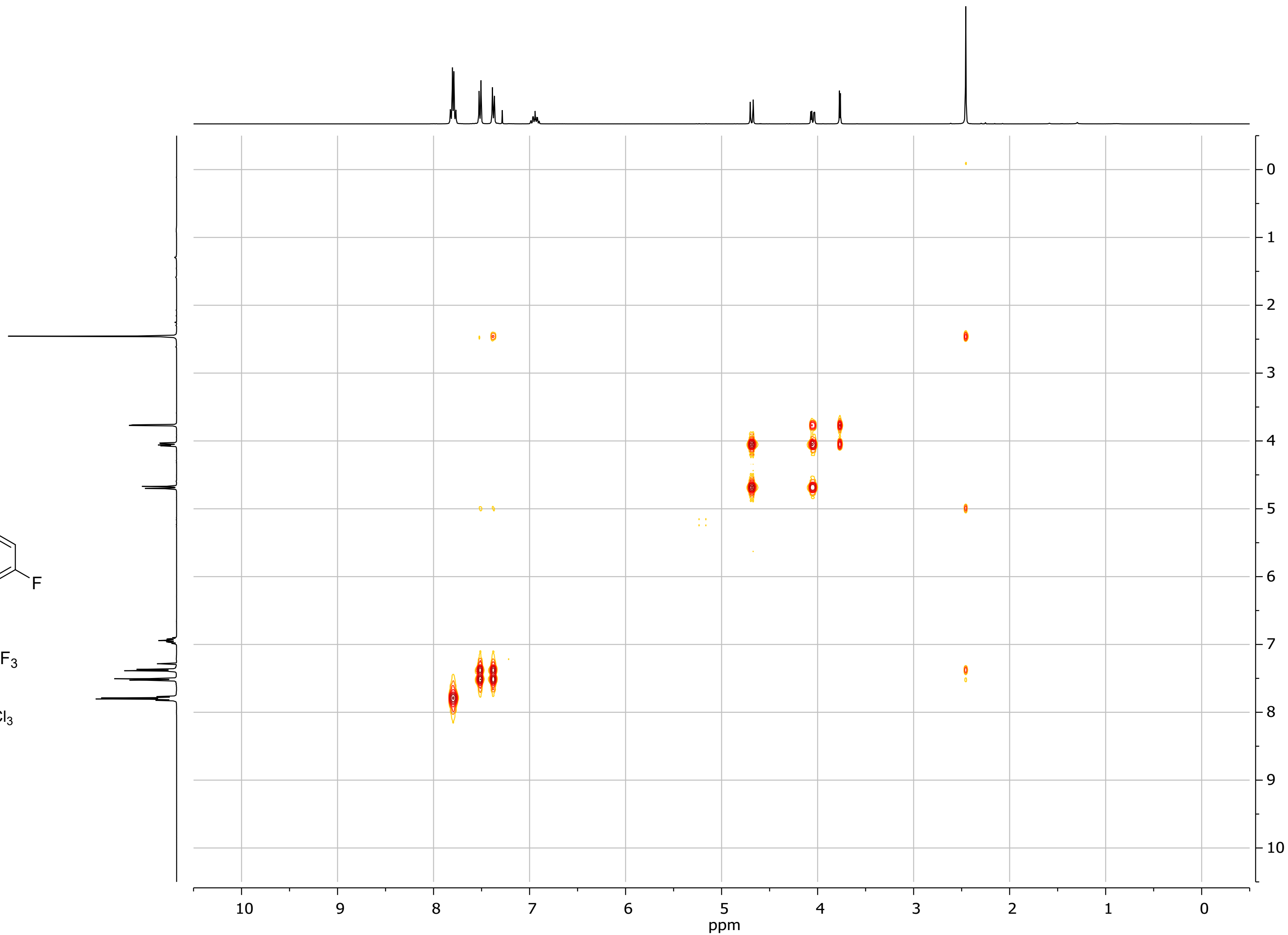

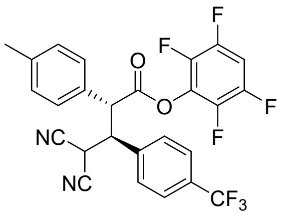

anti-**26**

2D ^{1}H-^{13}C HSQC, $CDCl_3$

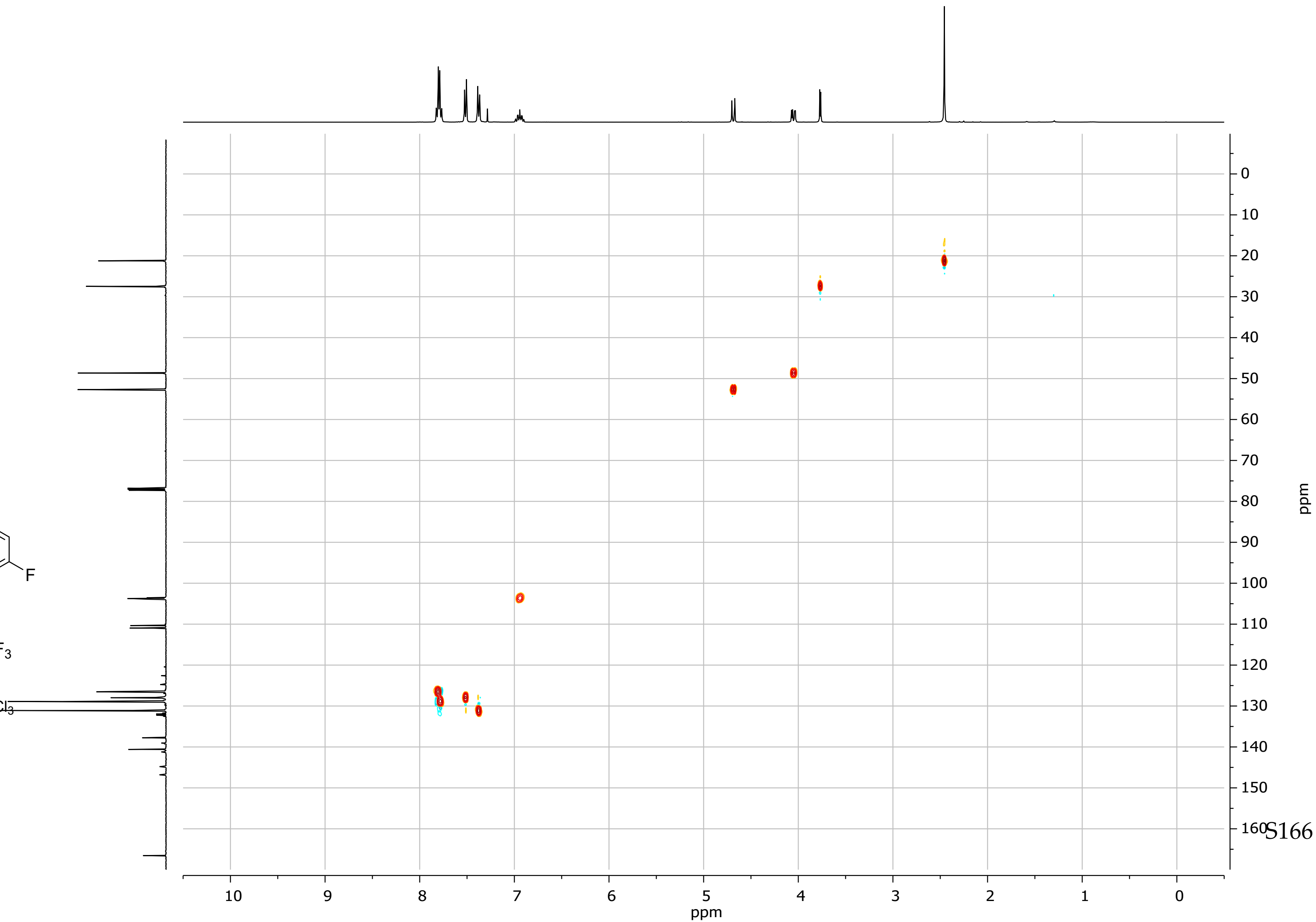

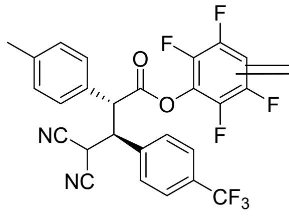

anti-**26**
2D ^{1}H-^{13}C HMBC, $CDCl_3$

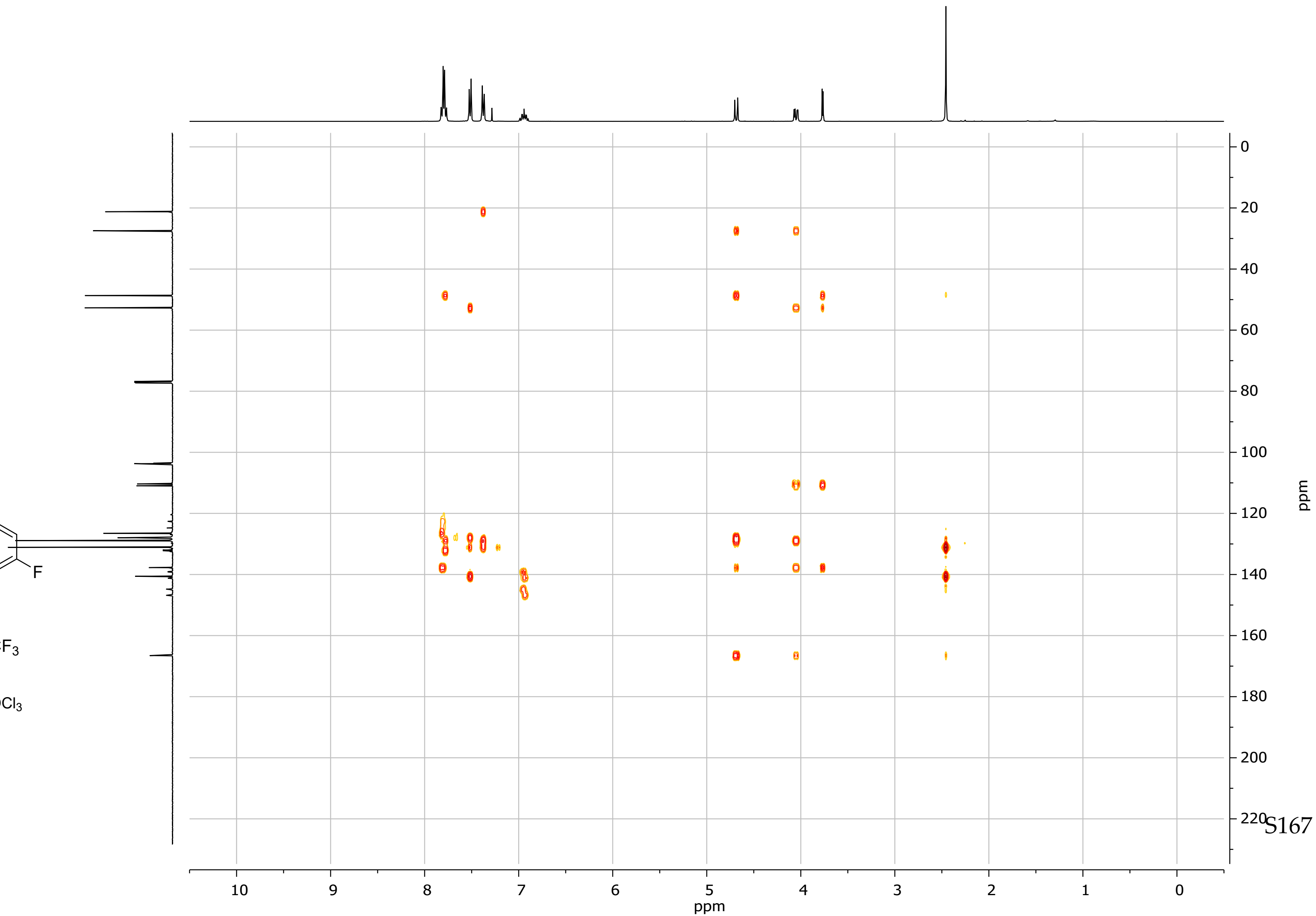

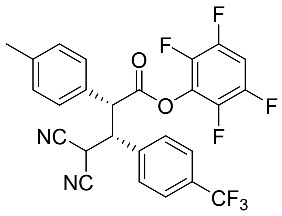

syn-**S22**

^{1}H, $CDCl_3$, 400 MHz

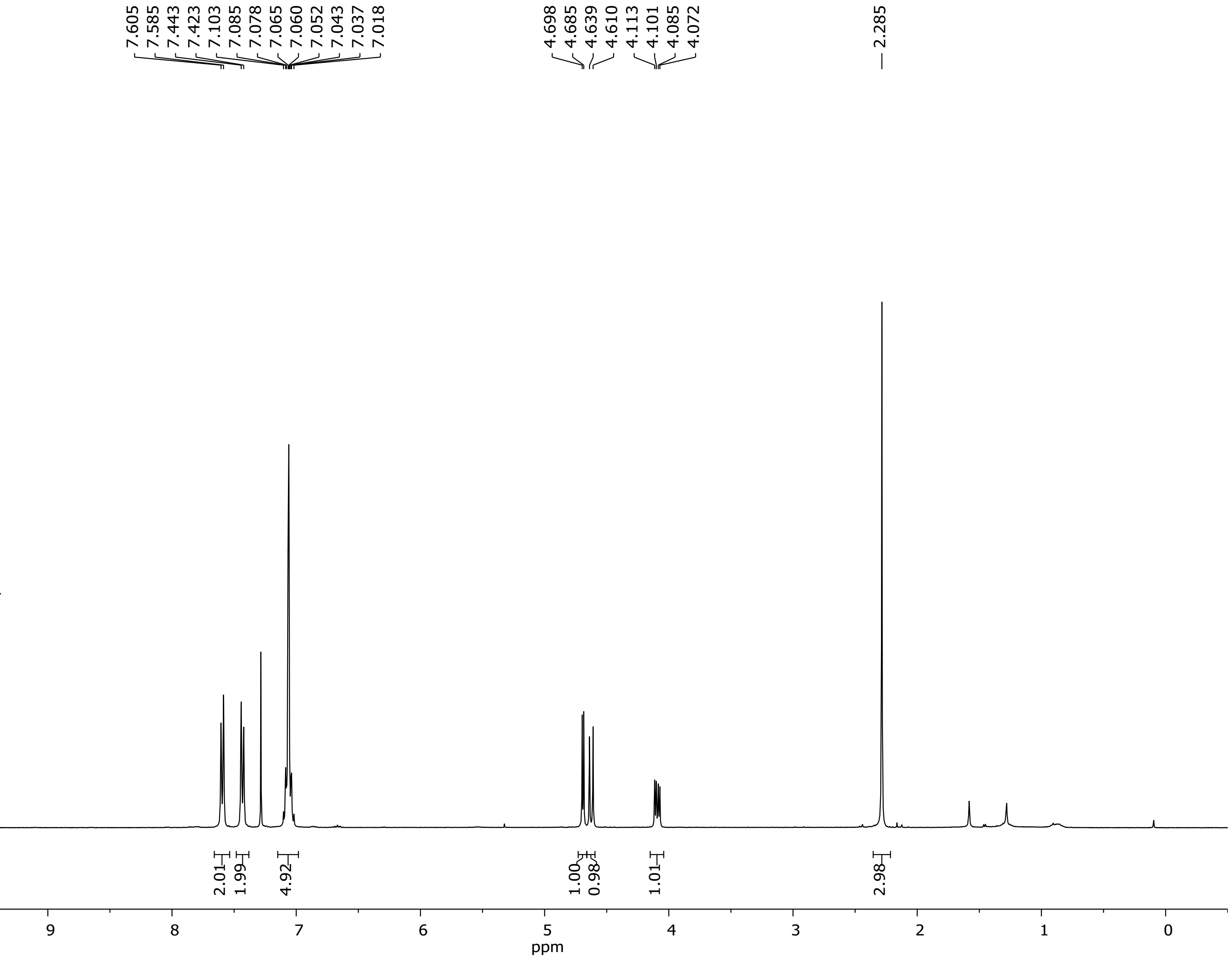

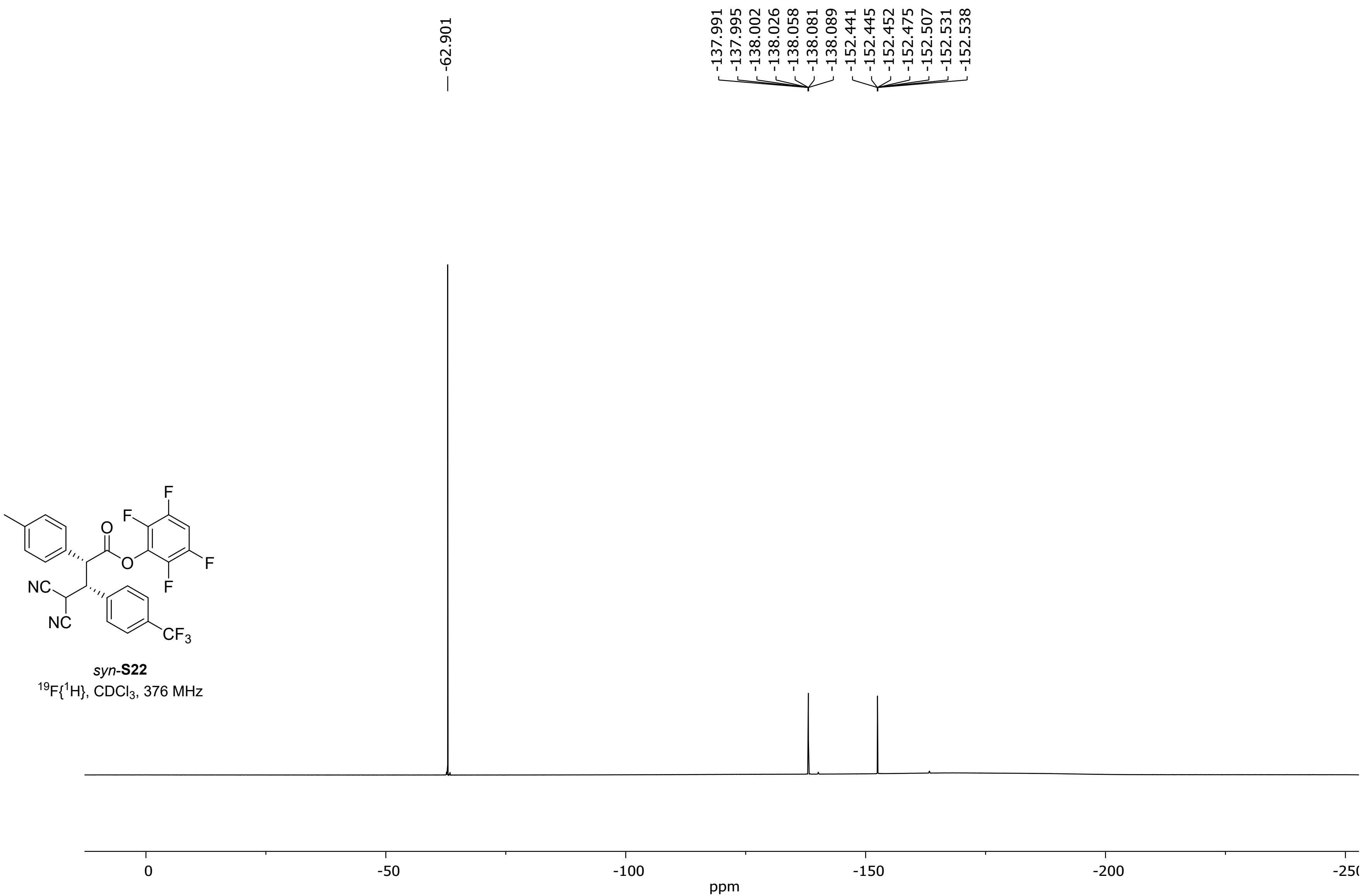

syn-**S22**

$^{19}F\{^{1}H\}$, $CDCl_3$, 376 MHz

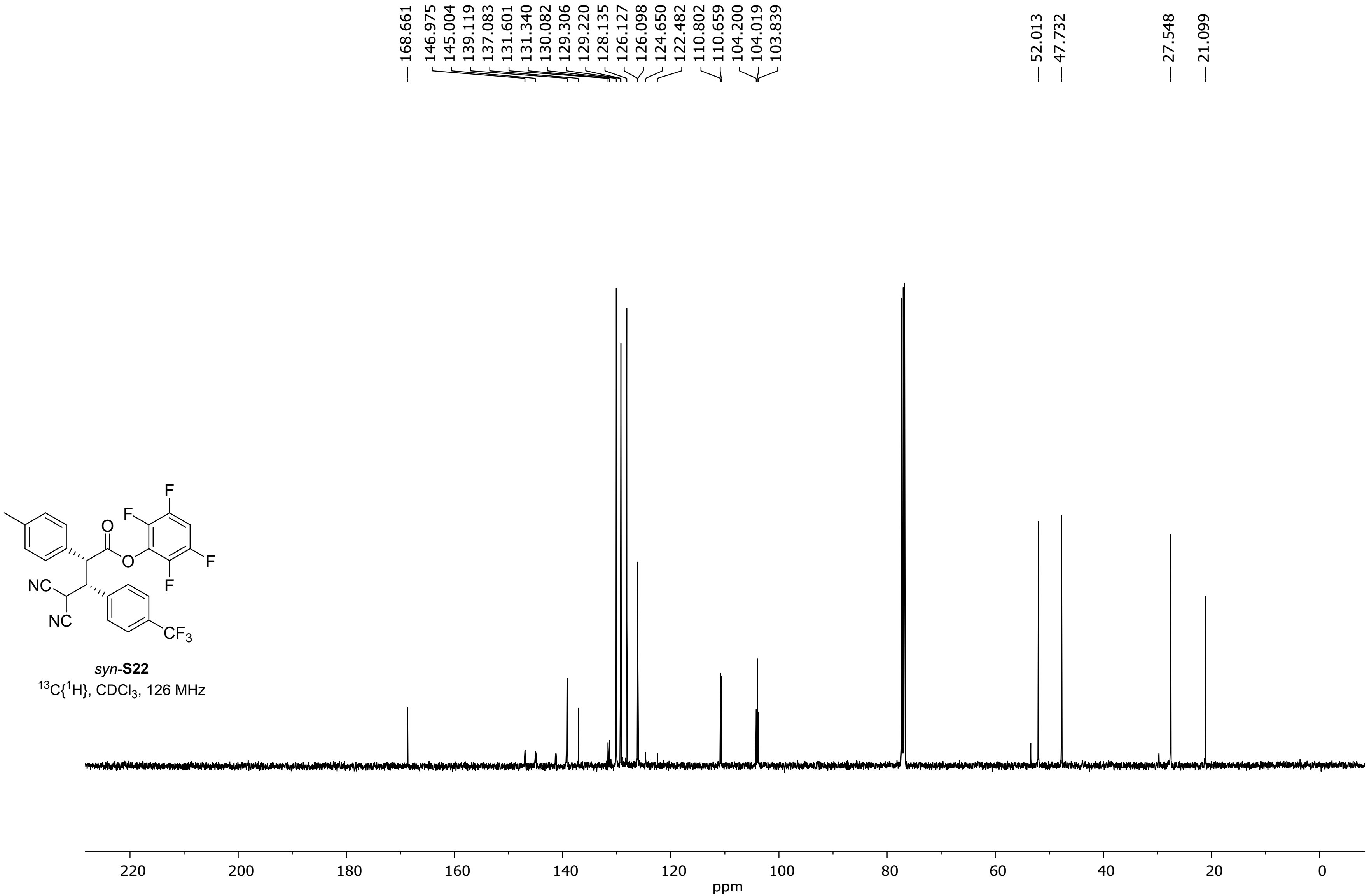

syn-**S22**

$^{13}C\{^{1}H\}$, $CDCl_3$, 126 MHz

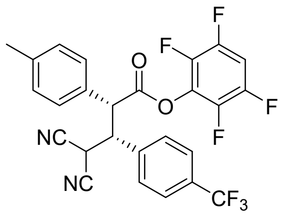

syn-**S22**
2D ^{1}H-^{1}H COSY, $CDCl_3$

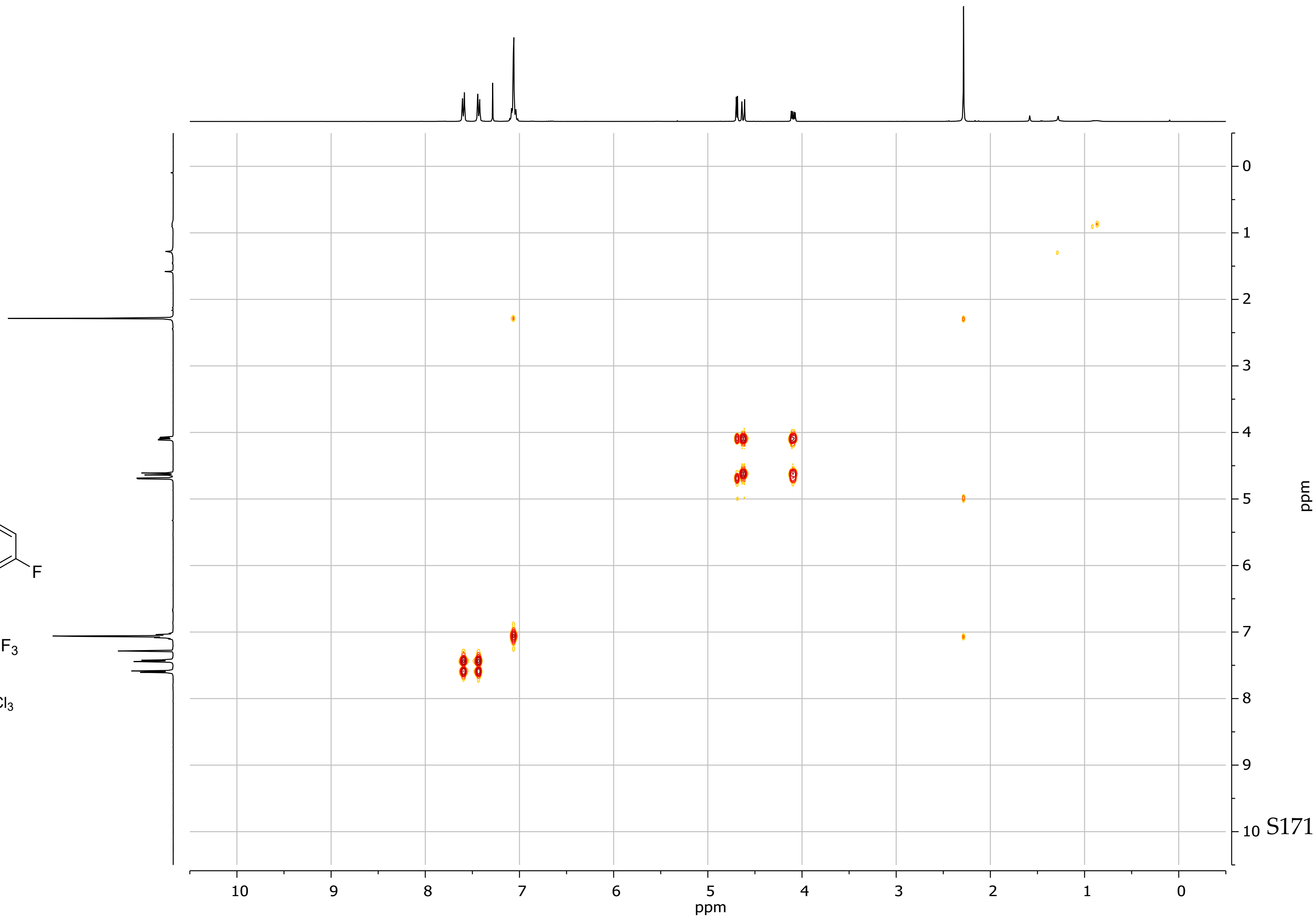

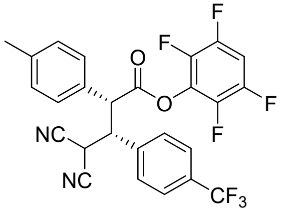

syn-**S22**

2D ^{1}H-^{13}C HSQC, $CDCl_3$

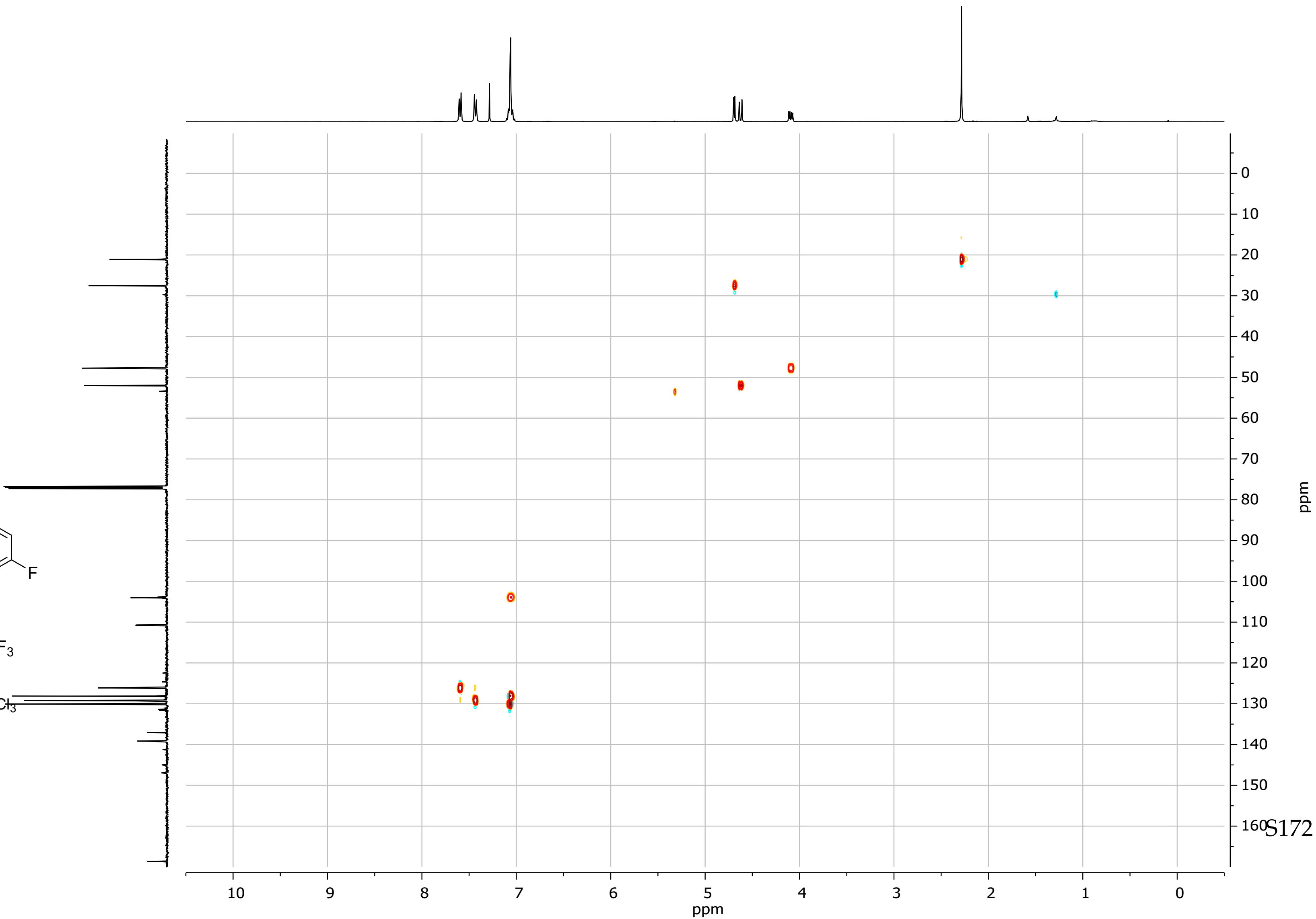

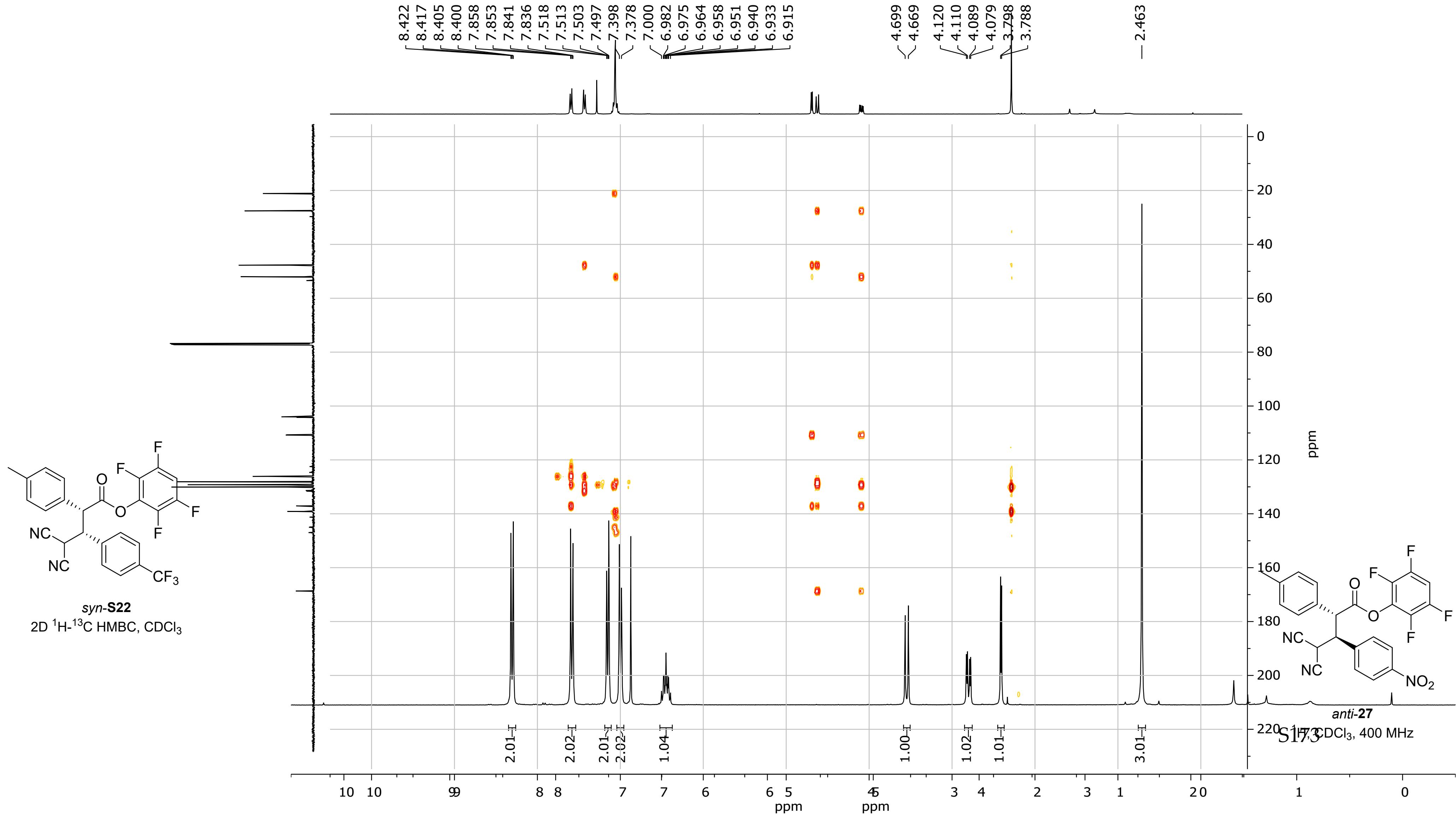
syn-S22
2D 1H-13C HMBC, CDCl3
anti-27
1H, CDCl3, 400 MHz
ppm

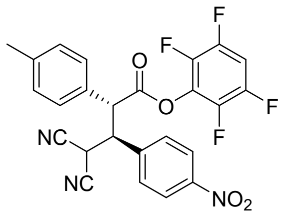

anti-**27**

$^{19}F\{^{1}H\}$, $CDCl_3$, 376 MHz

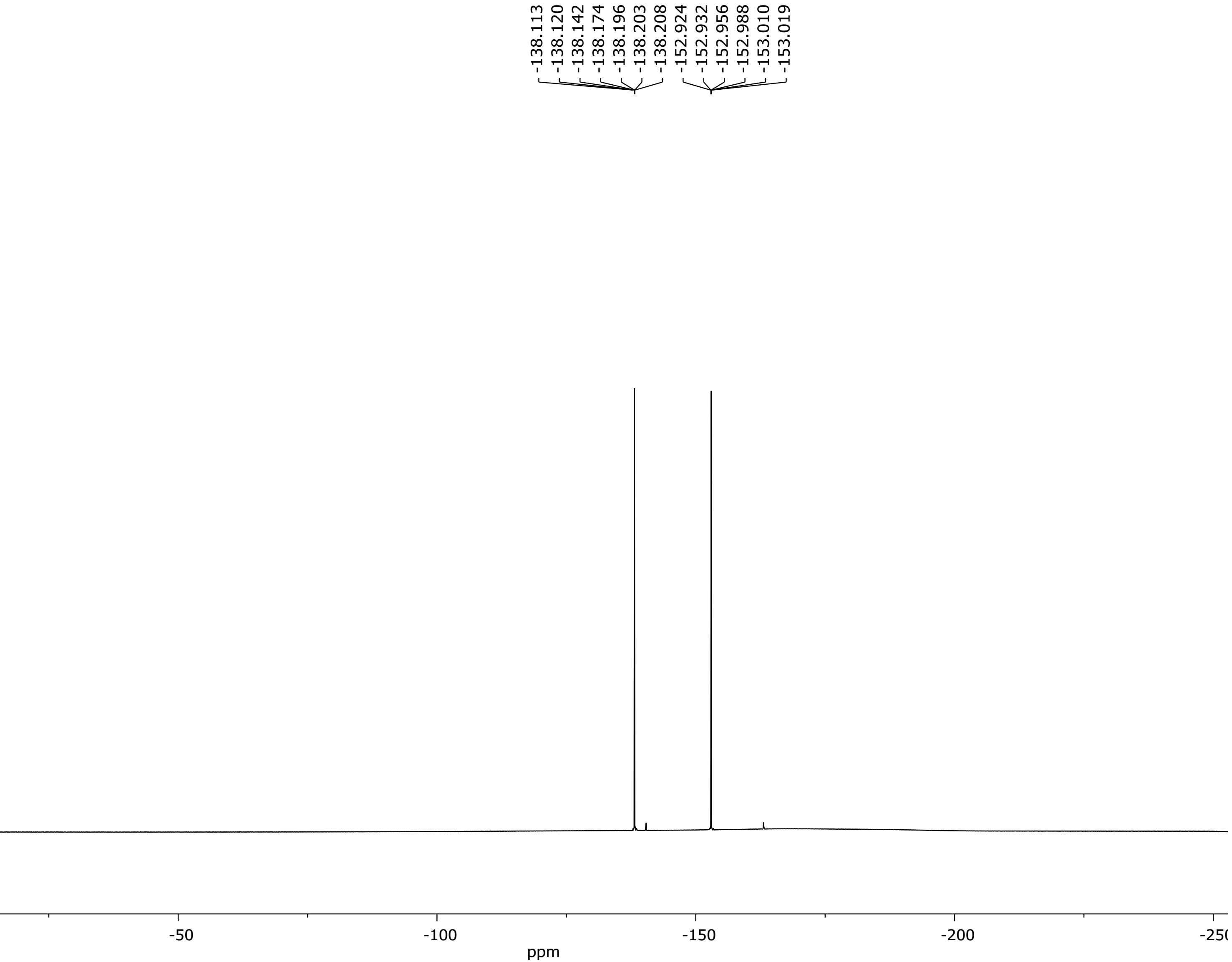

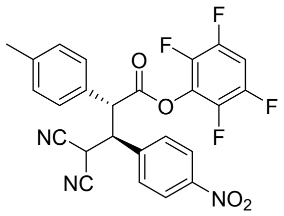

anti-**27**

$^{13}C\{^1H\}$, $CDCl_3$, 126 MHz

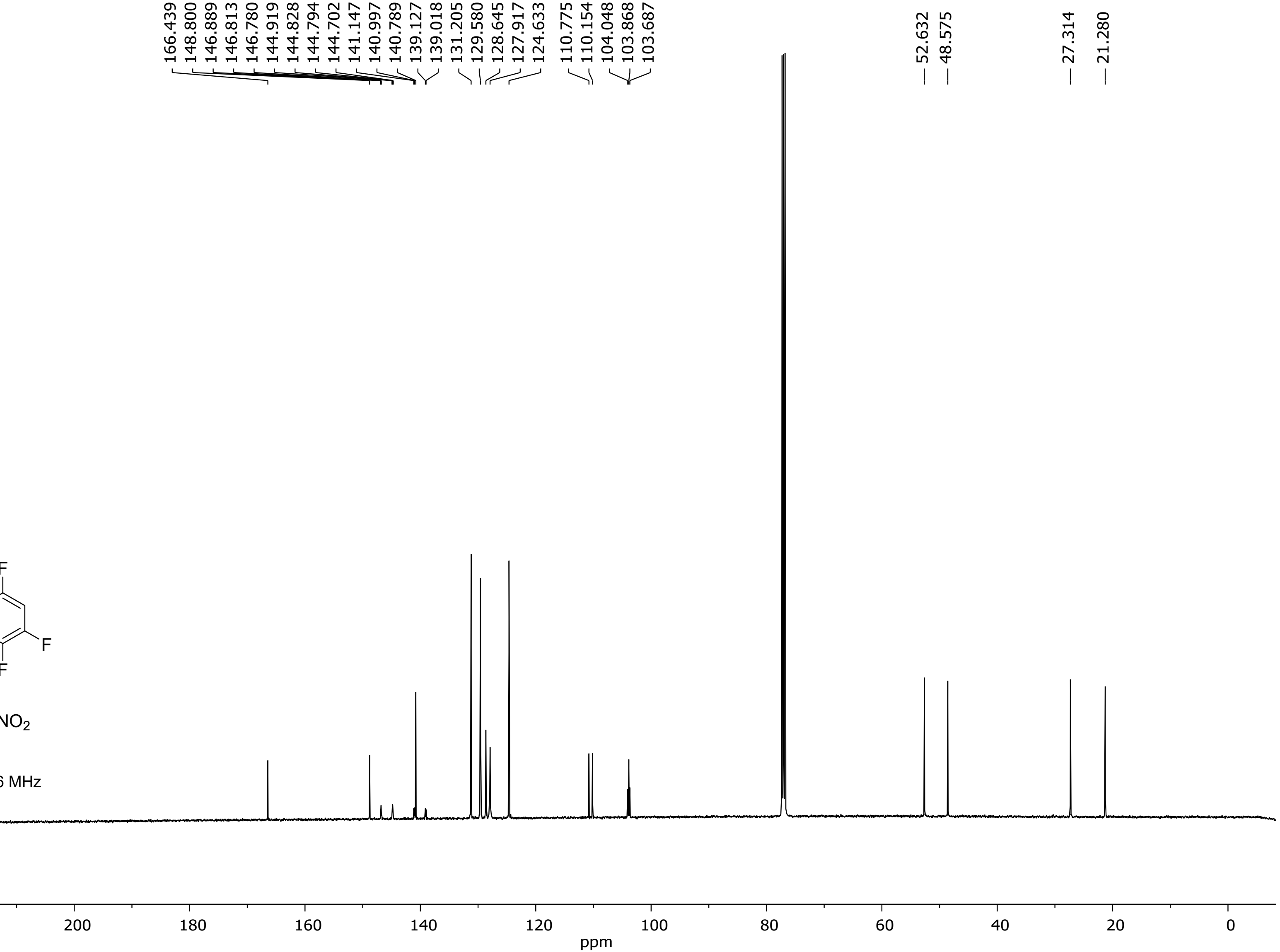

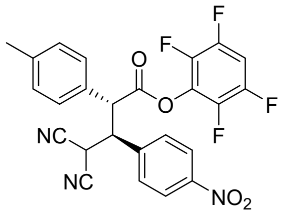

anti-**27**

2D ^{1}H-^{13}C HSQC, $CDCl_3$

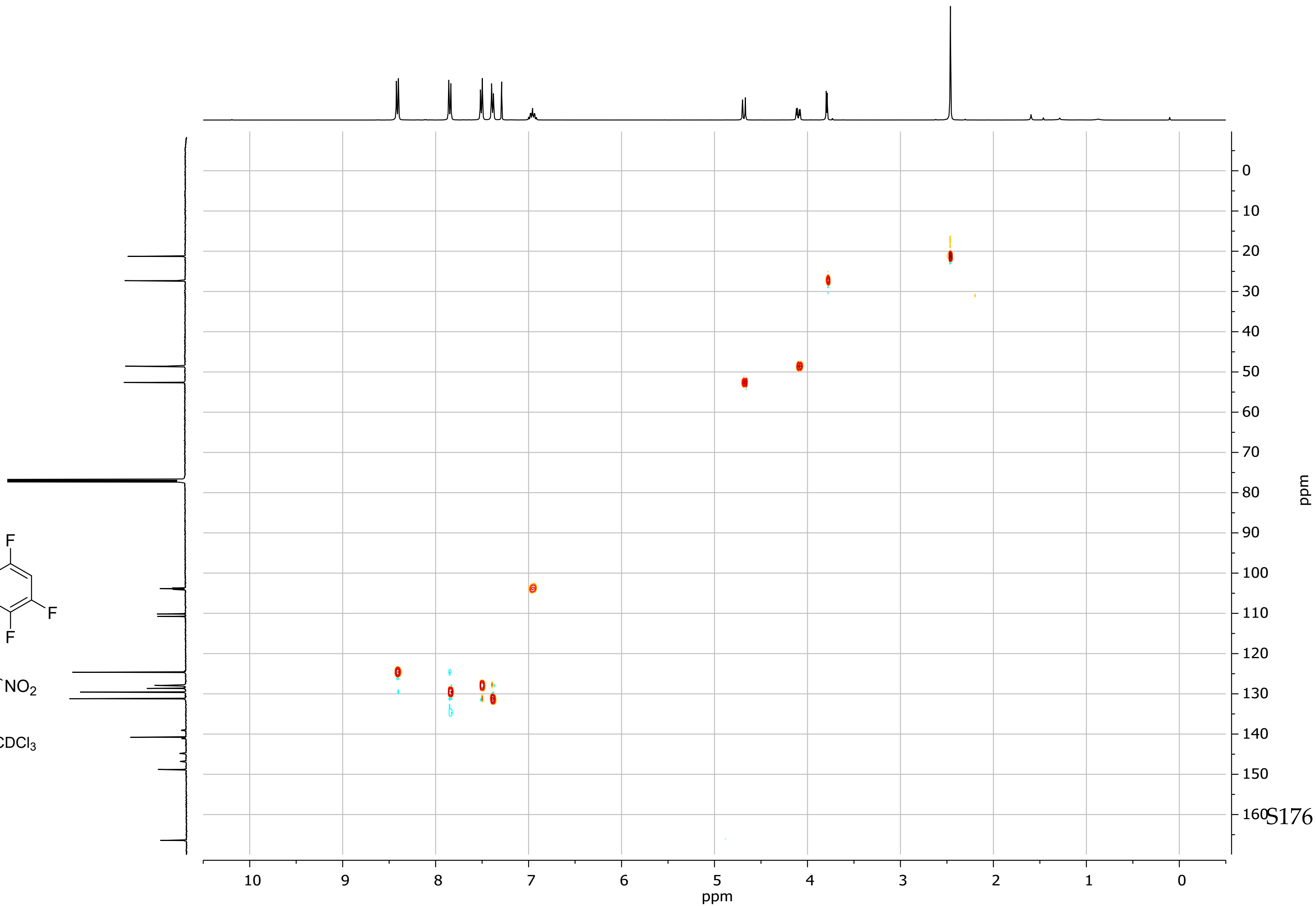

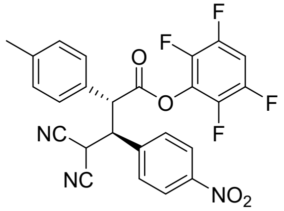

anti-**27**
2D ^1H-^{13}C HMBC, $CDCl_3$

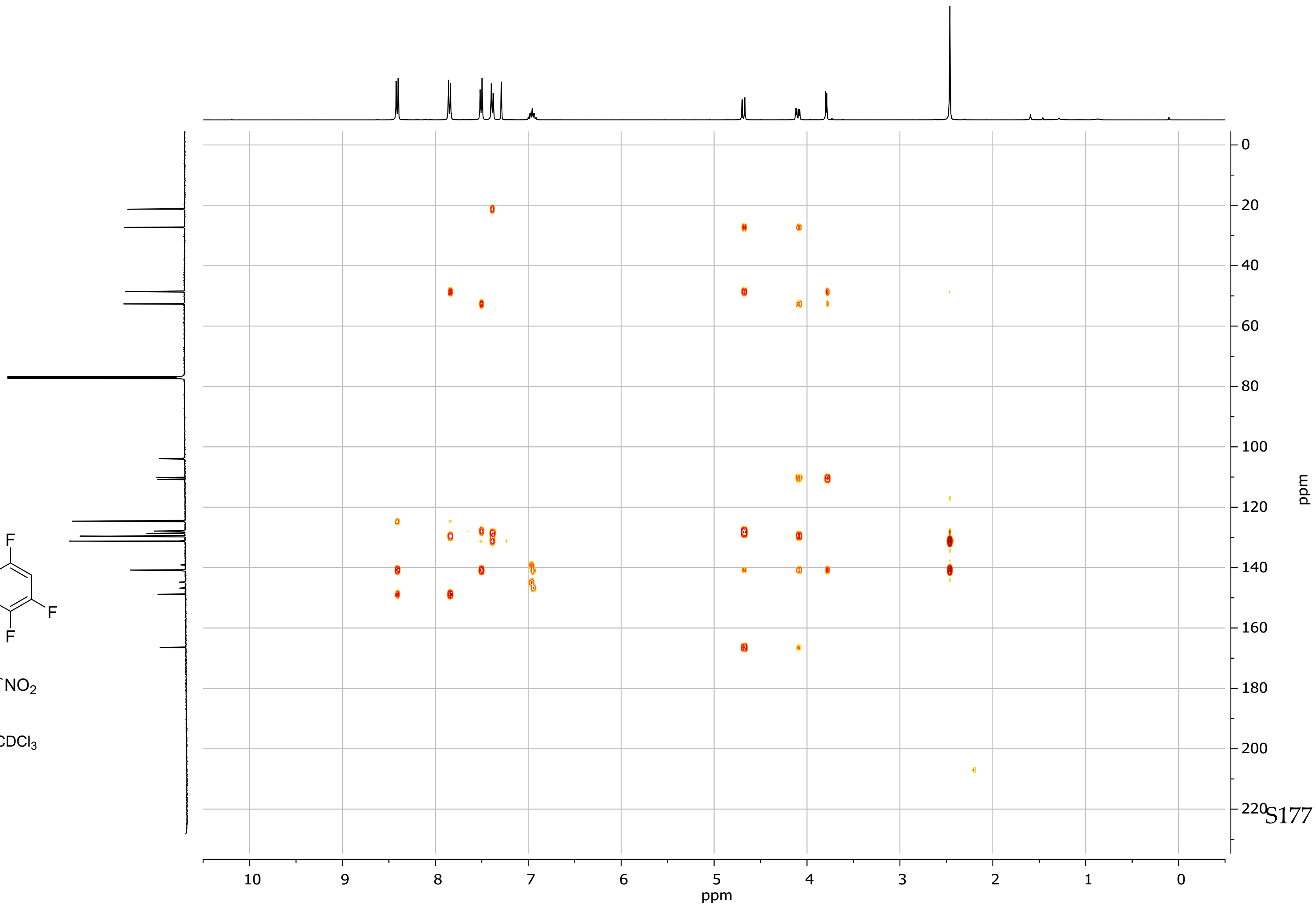

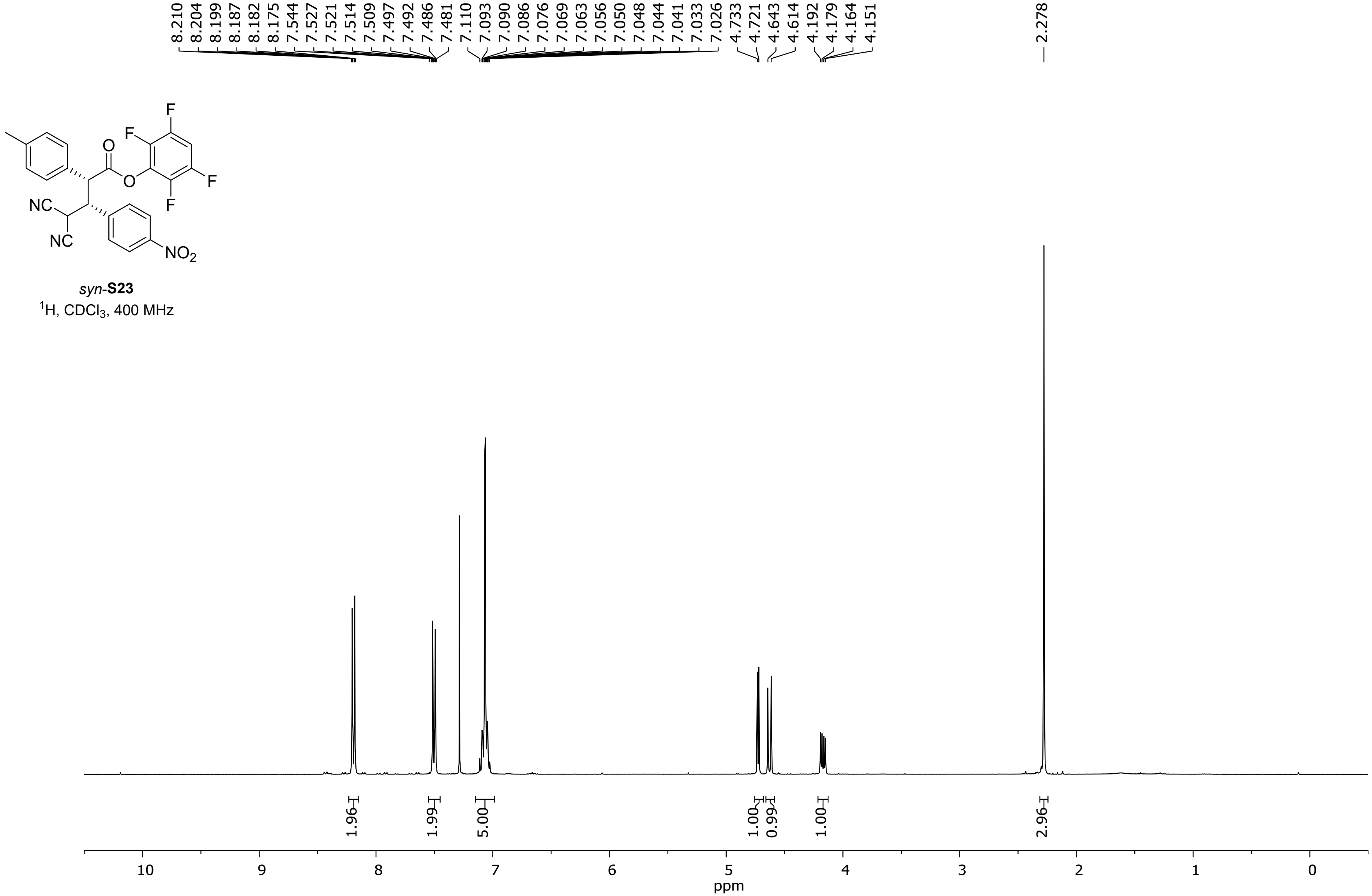

syn-**S23**
^1H, $CDCl_3$, 400 MHz

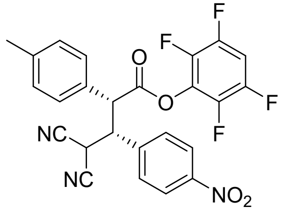

syn-**S23**
$^{19}F\{^{1}H\}$, $CDCl_3$, 376 MHz

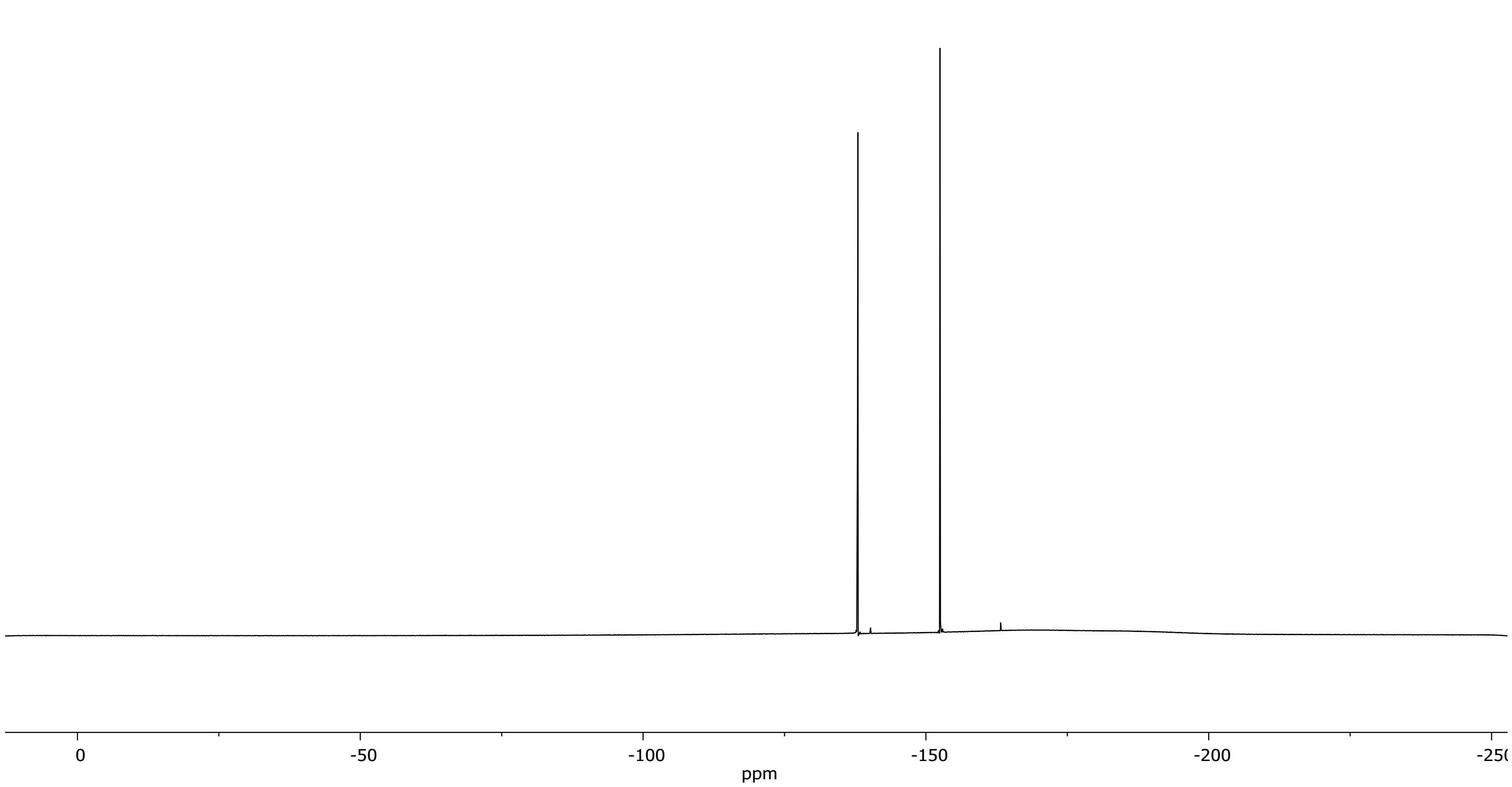

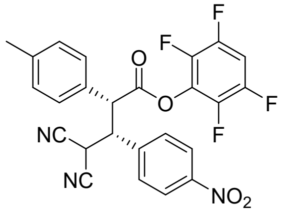

syn-**S23**

$^{13}C\{^{1}H\}$, $CDCl_3$, 126 MHz

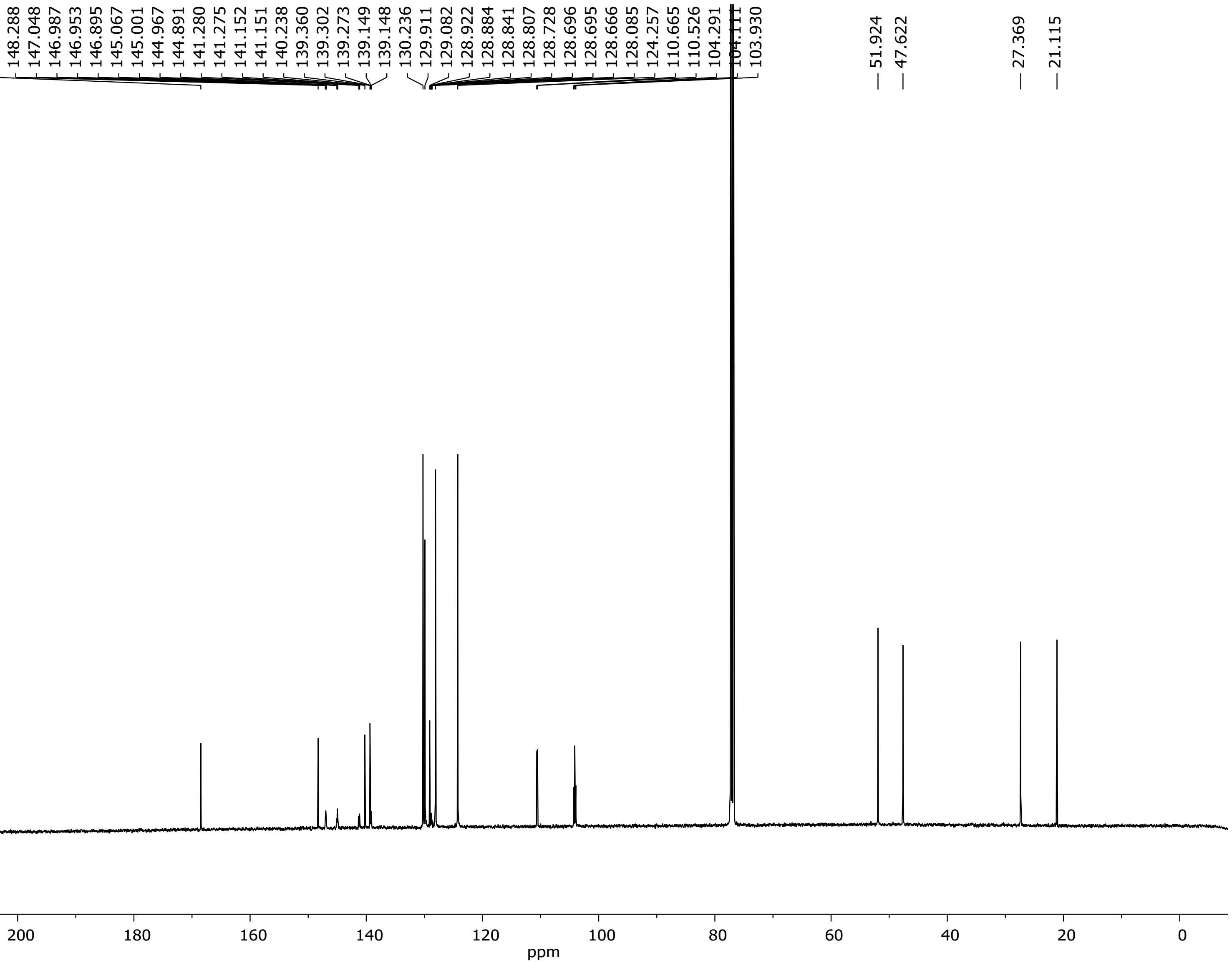

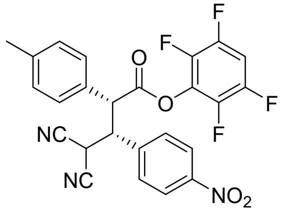

syn-**S23**
2D ^1H-^{13}C HSQC, $CDCl_3$

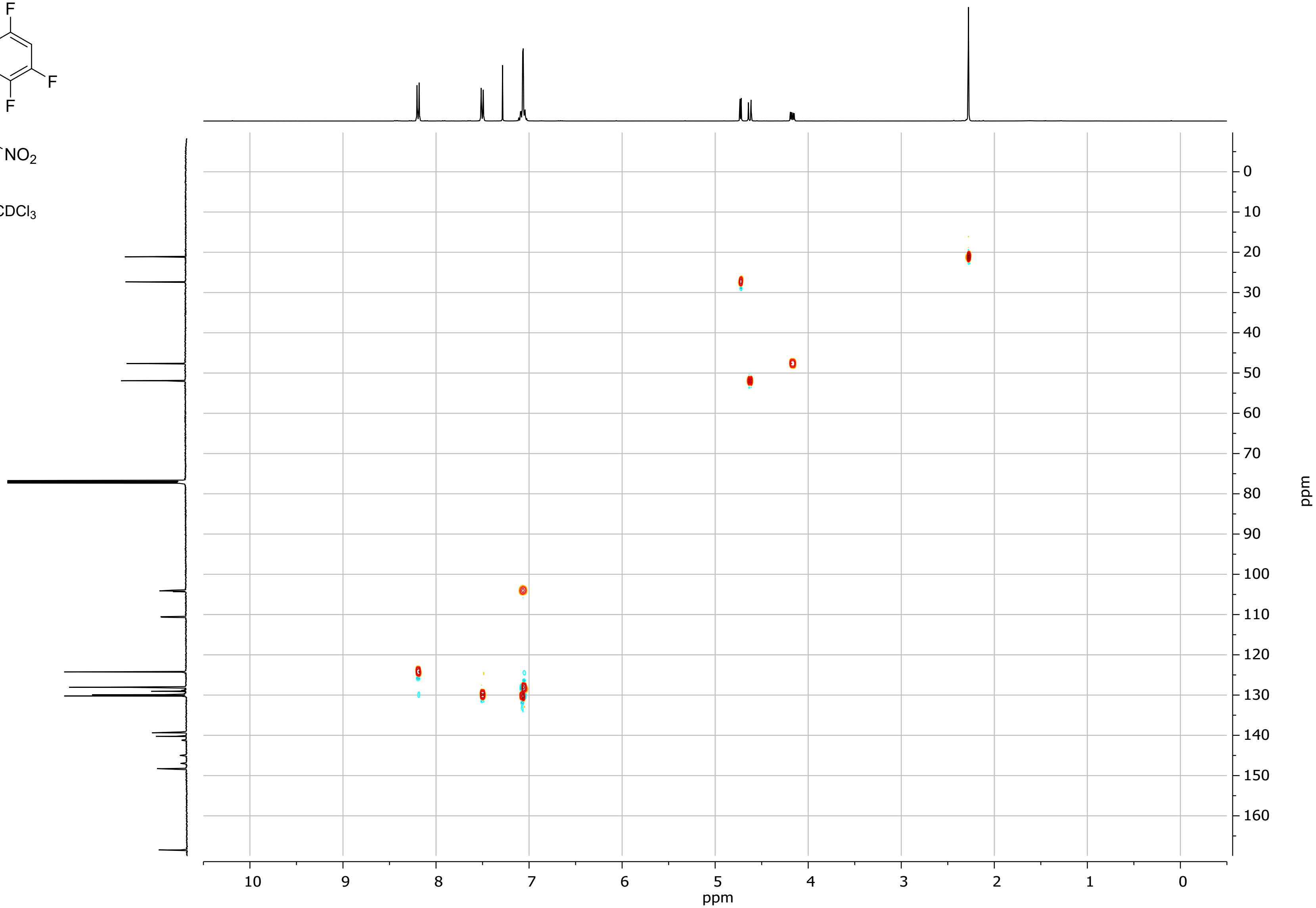

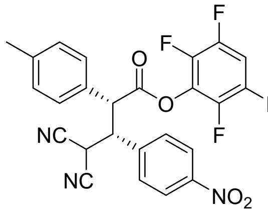

syn-**S23**
2D ^{1}H-^{13}C HMBC, $CDCl_3$

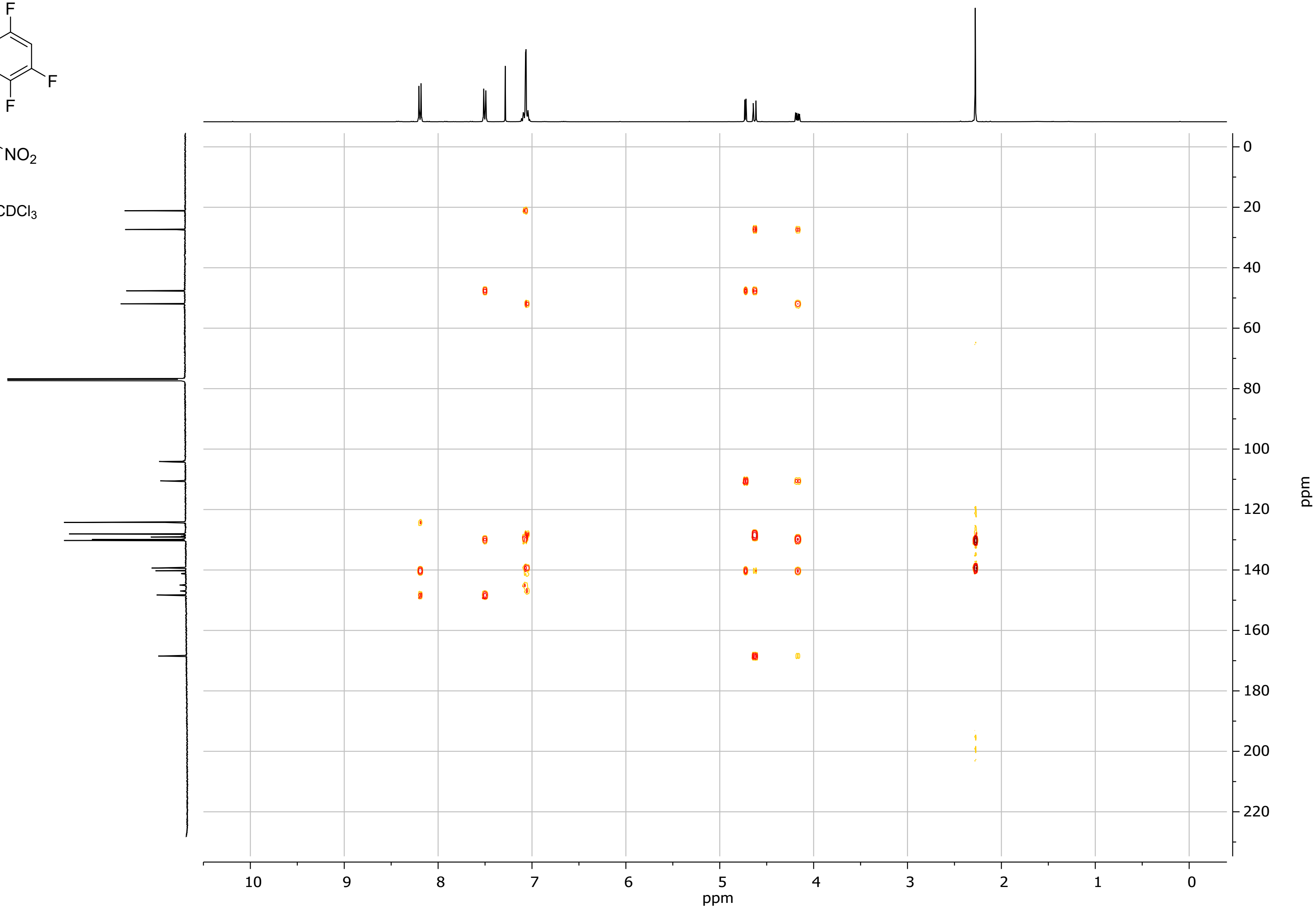

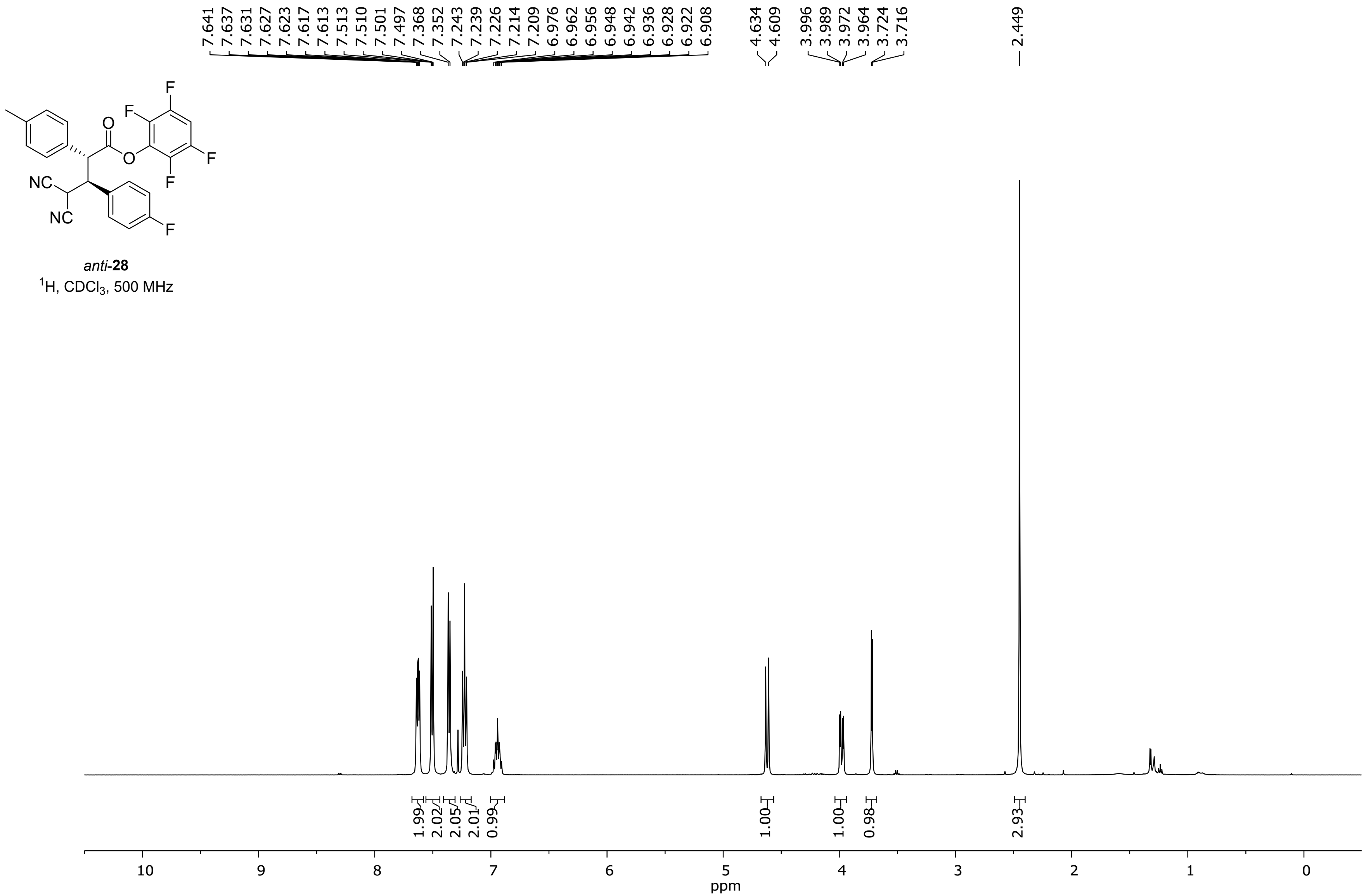

7.641
7.637
7.631
7.627
7.623
7.617
7.613
7.513
7.510
7.501
7.497
7.368
7.352
7.243
7.239
7.226
7.214
7.209
6.976
6.962
6.956
6.948
6.942
6.936
6.928
6.922
6.908
4.634
4.609
3.996
3.989
3.972
3.964
3.724
3.716
2.449
anti-**28**
^{1}H, $CDCl_3$, 500 MHz
1.99
2.02
2.05
2.01
0.99
1.00
1.00
0.98
2.93
10
9
8
7
6
5
4
3
2
1
0
ppm

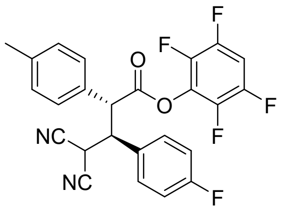

anti-**28**
$^{19}F\{^{1}H\}$, $CDCl_3$, 470 MHz

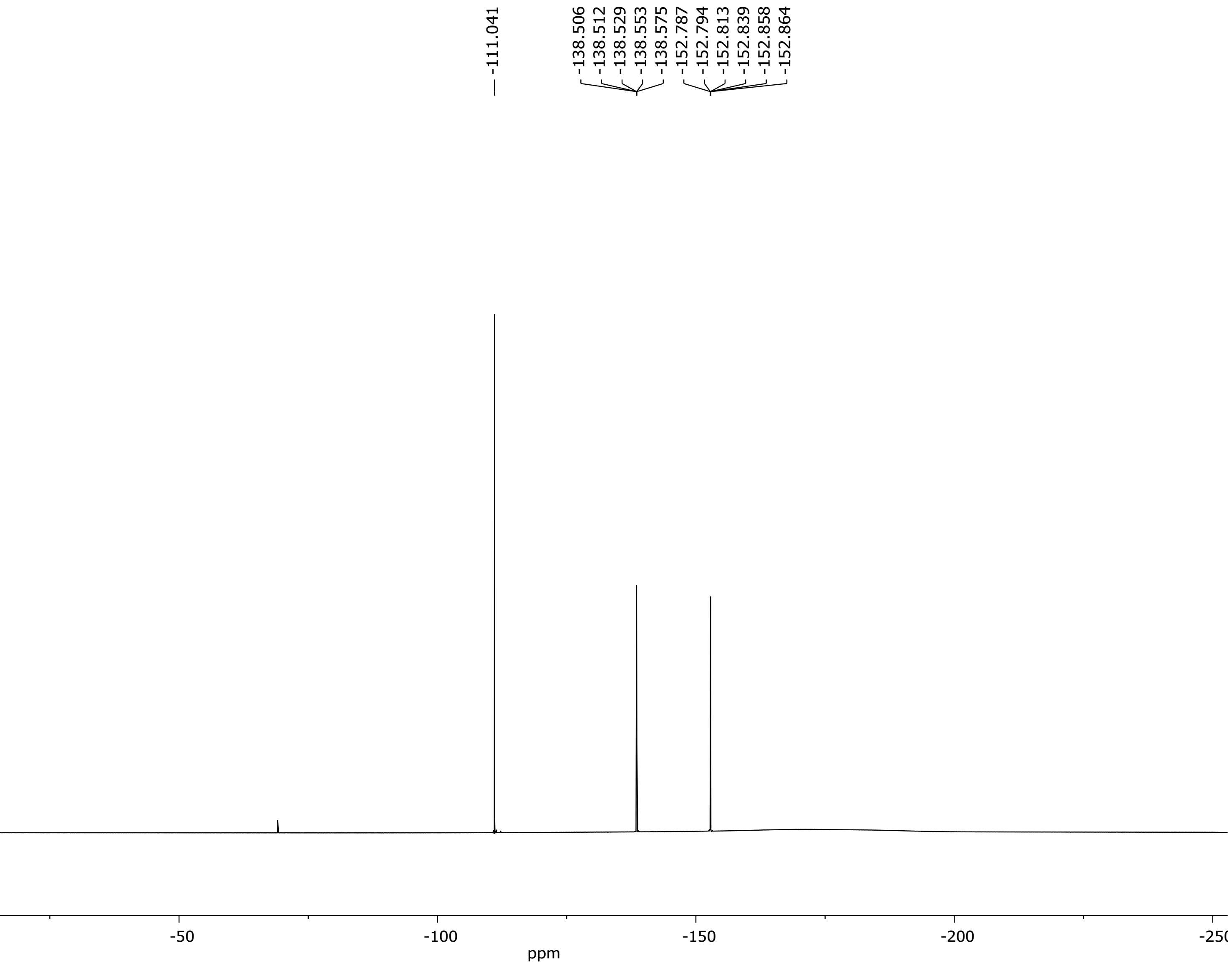

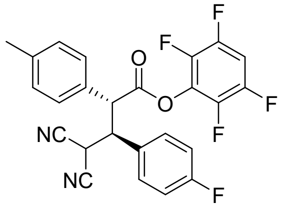

anti-**28**

$^{13}C\{^1H\}$, $CDCl_3$, 126 MHz

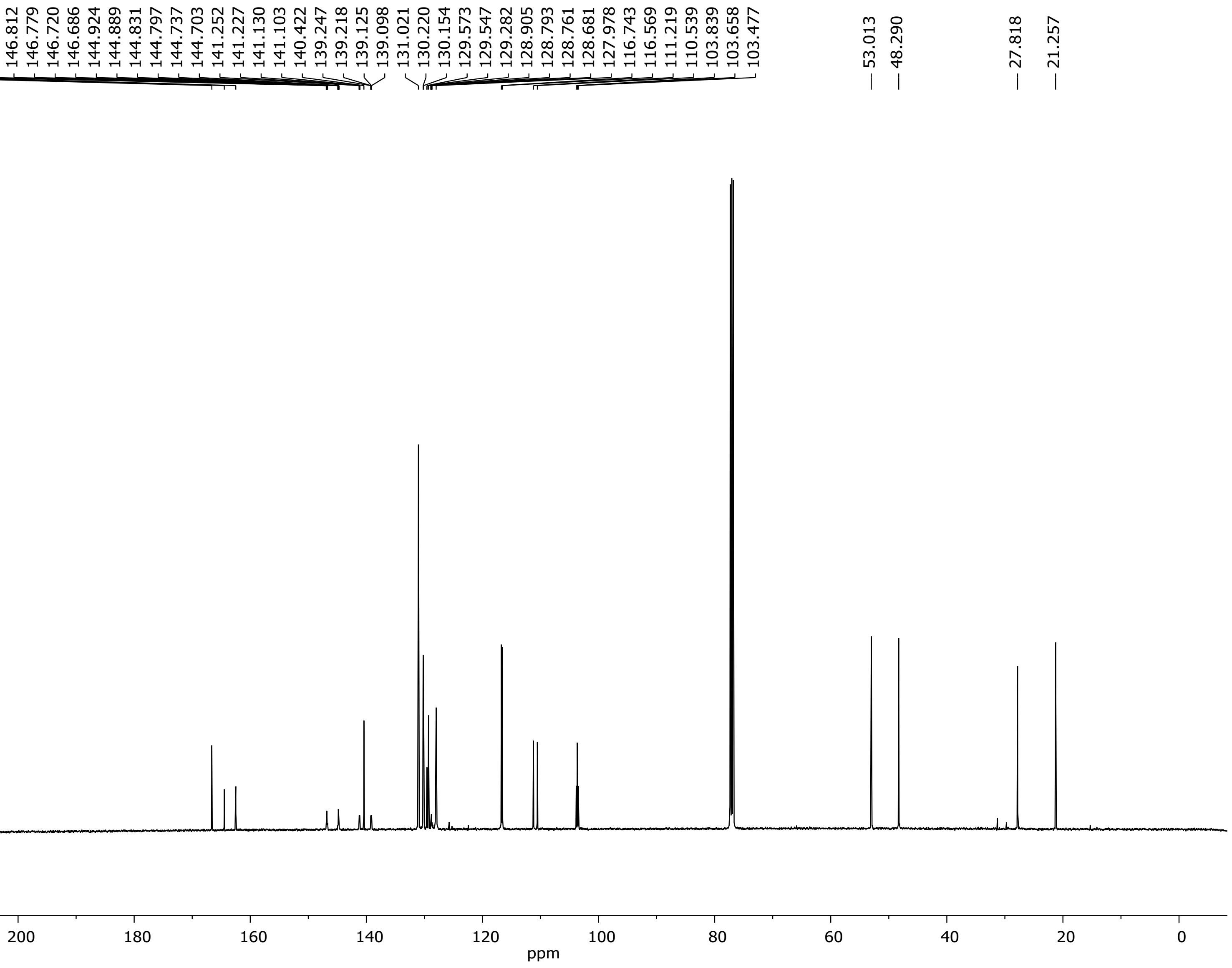

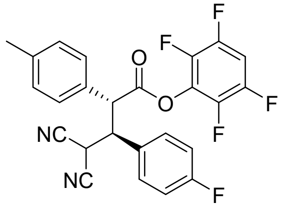

anti-**28**

2D ^{1}H-^{1}H COSY, $CDCl_3$

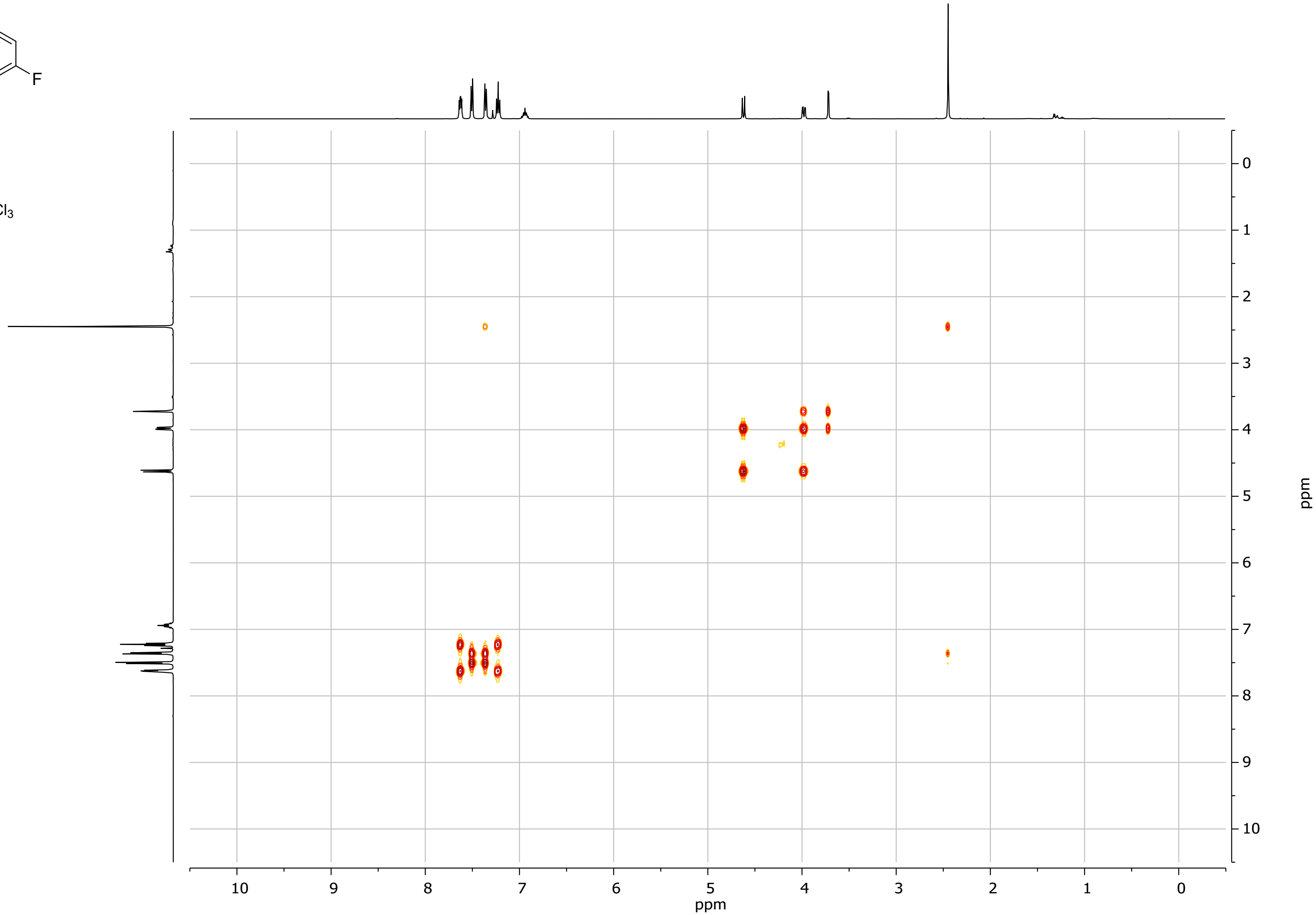

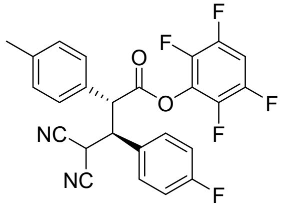

anti-**28**
2D ^1H-^{13}C HSQC, $CDCl_3$

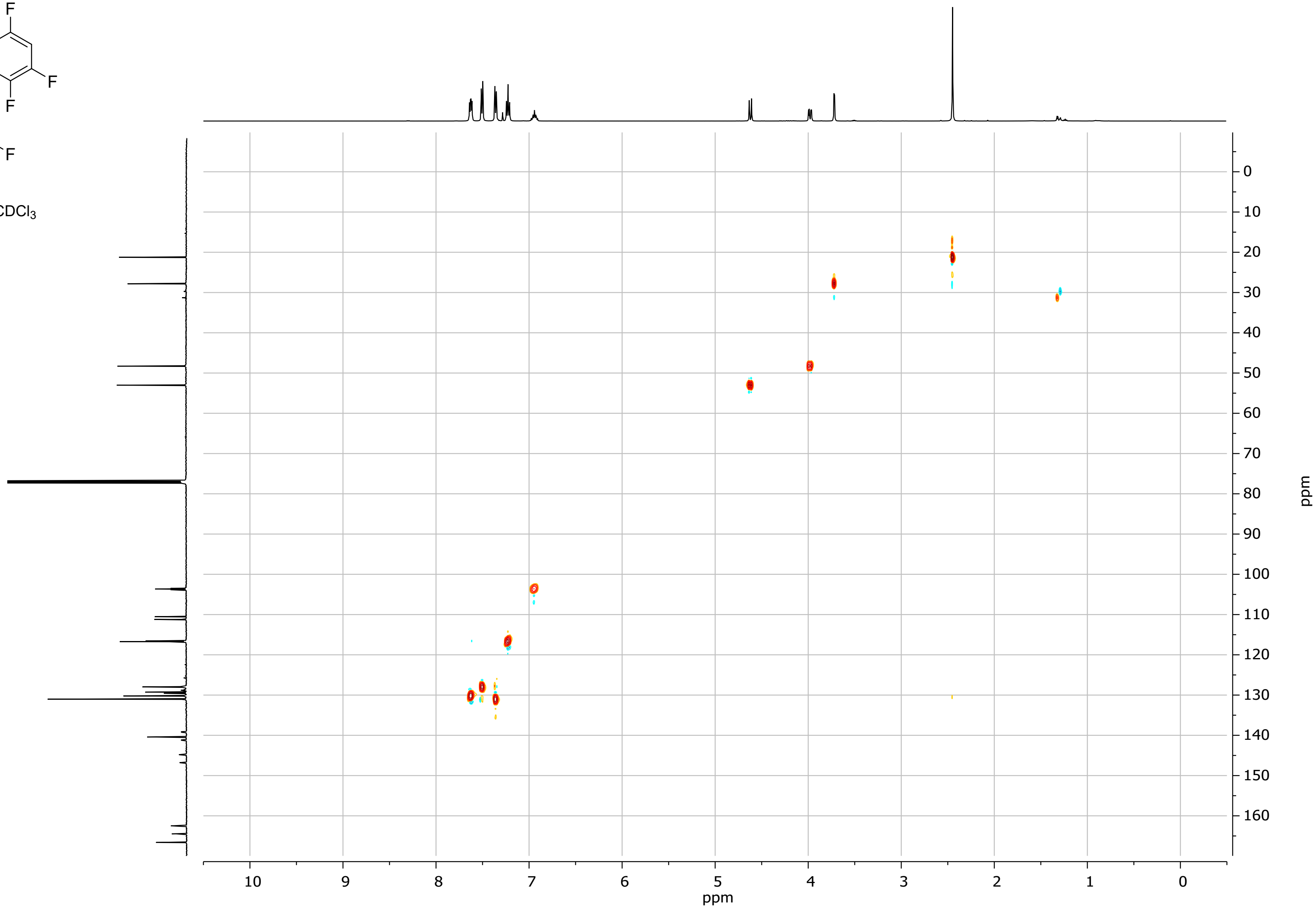

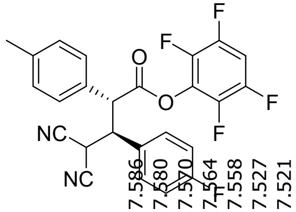

anti-**28**

2D ^{1}H-^{13}C HMBC, $CDCl_3$

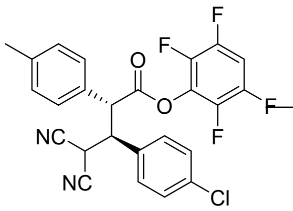

anti-**29**

^{1}H, $CDCl_3$, 400 MHz

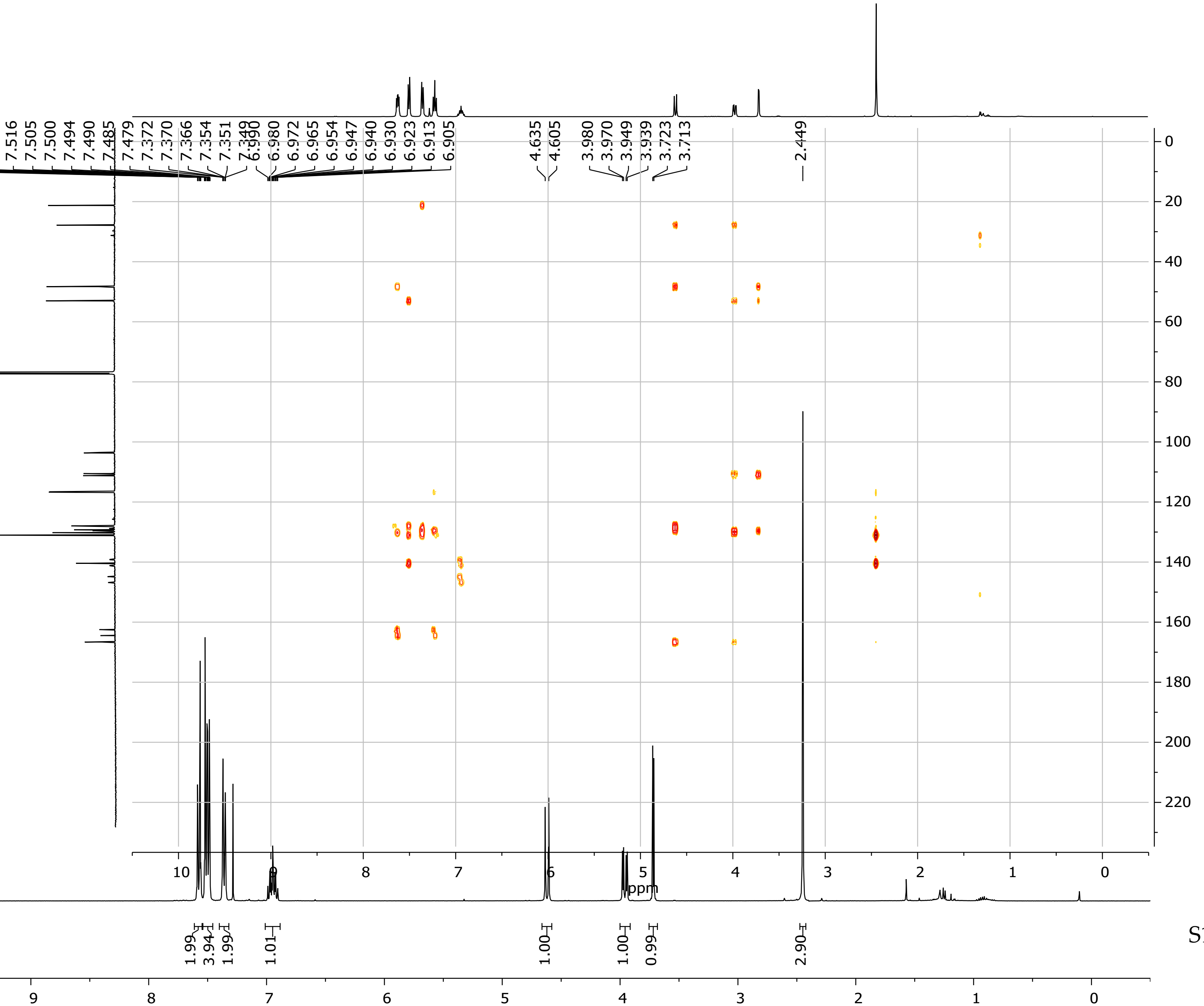

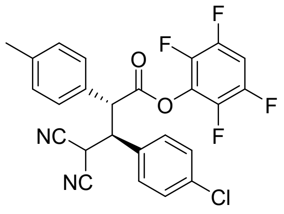

anti-**29**

$^{19}F\{^{1}H\}$, $CDCl_3$, 376 MHz

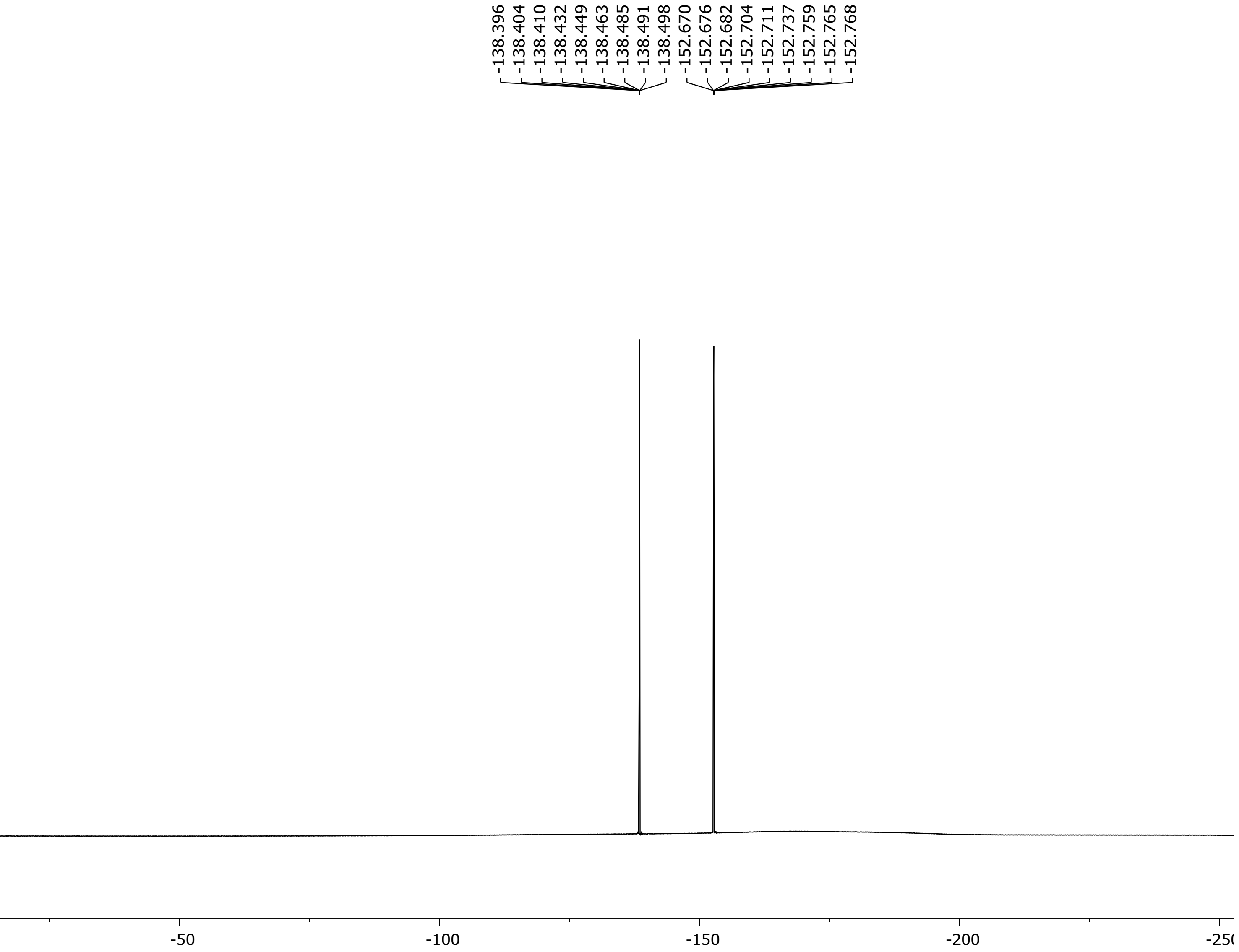

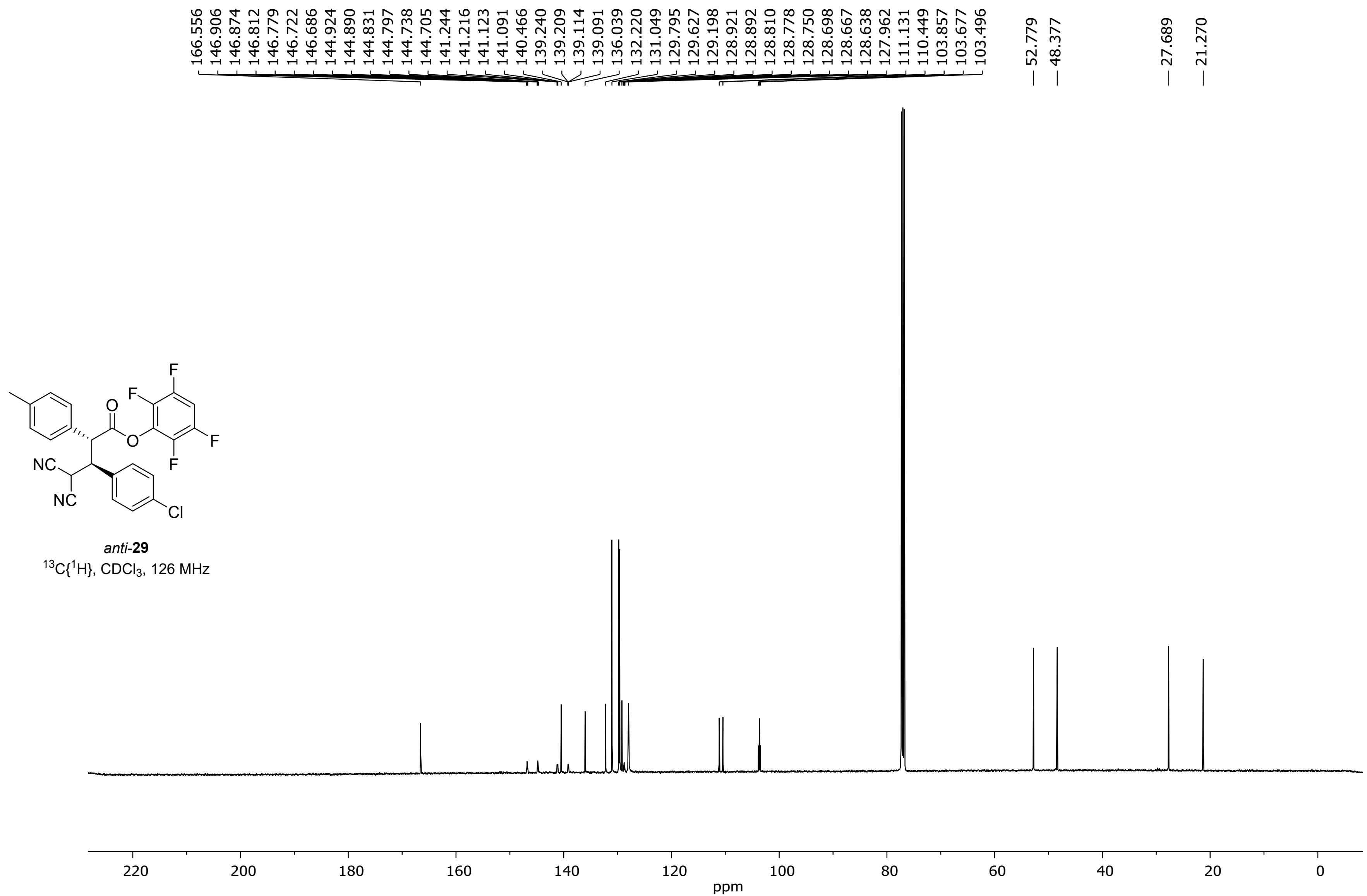

anti-**29**
$^{13}C\{^{1}H\}$, $CDCl_3$, 126 MHz

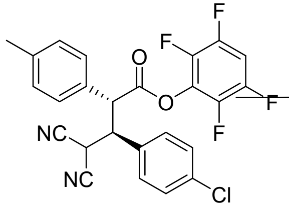

anti-**29**
2D ^{1}H-^{1}H COSY, $CDCl_3$

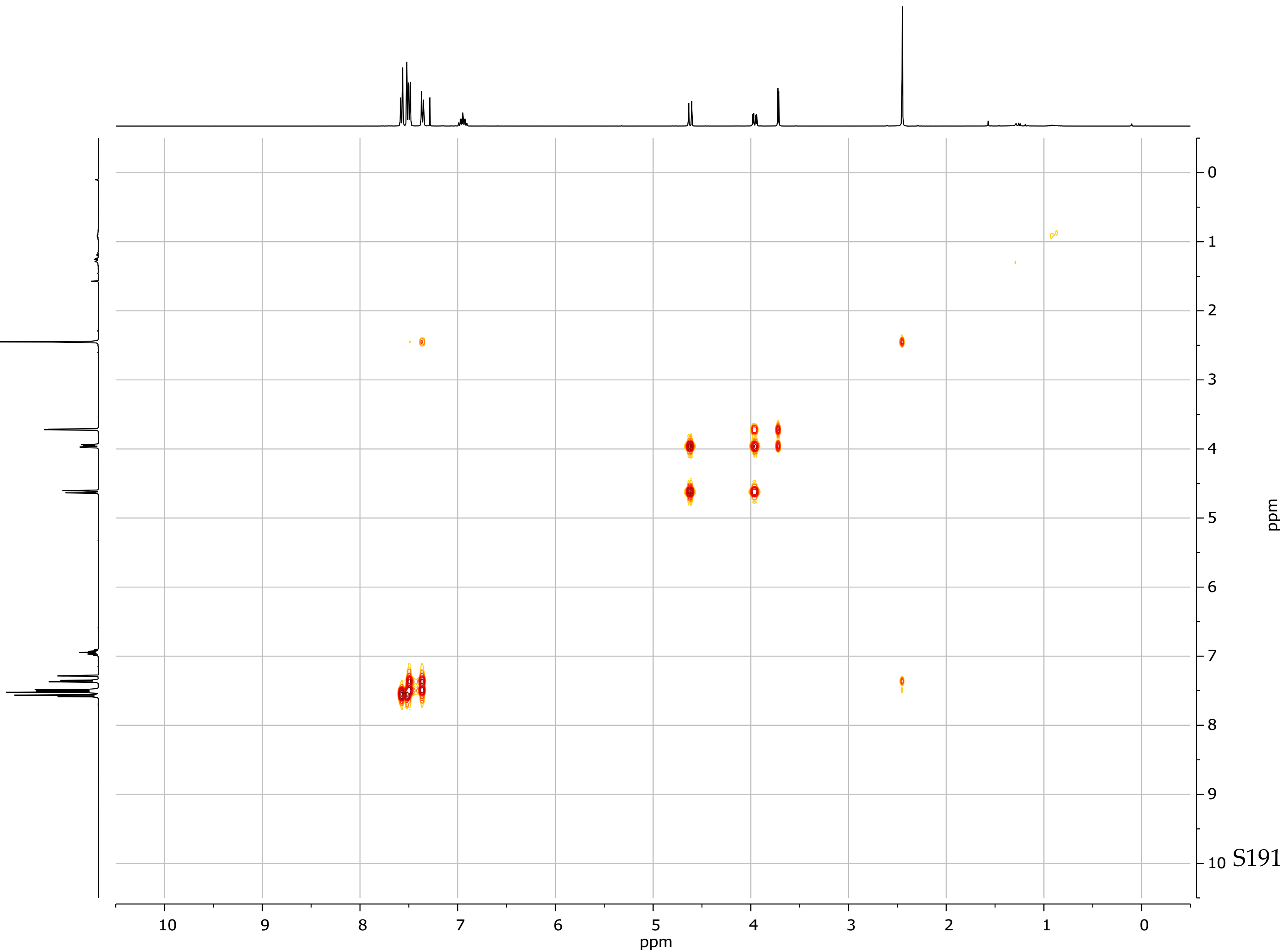

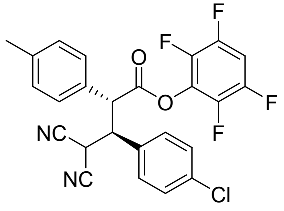

anti-**29**

2D ^{1}H-^{13}C HSQC, $CDCl_3$

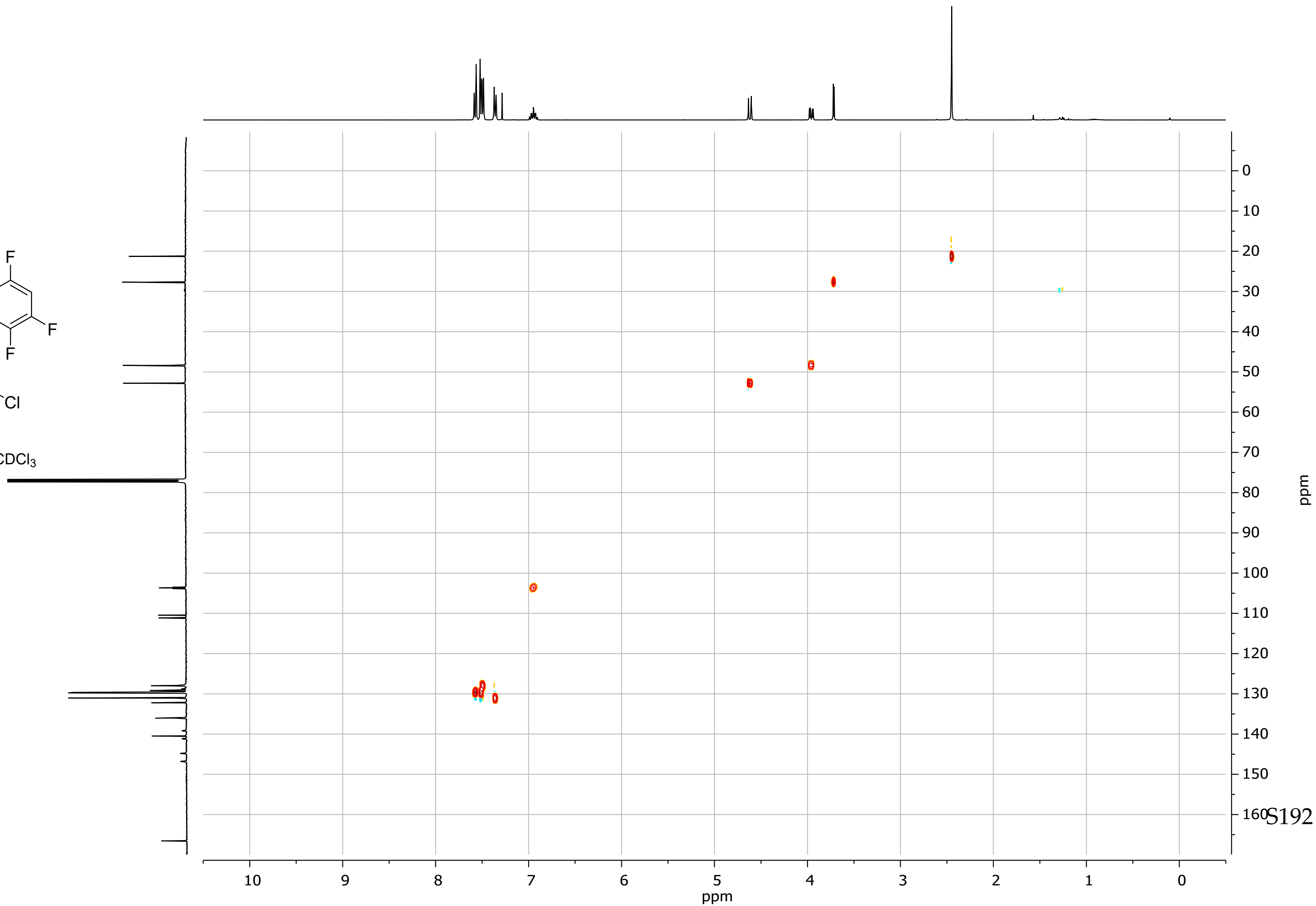

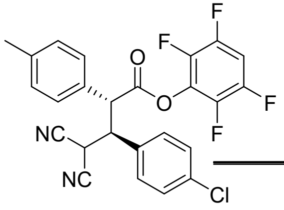

anti-**29**

2D ^{1}H-^{13}C HMBC, $CDCl_3$

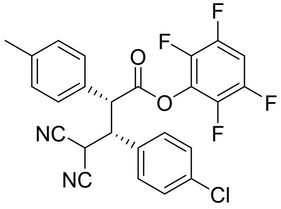

syn-**S24**

^{1}H, $CDCl_3$, 400 MHz

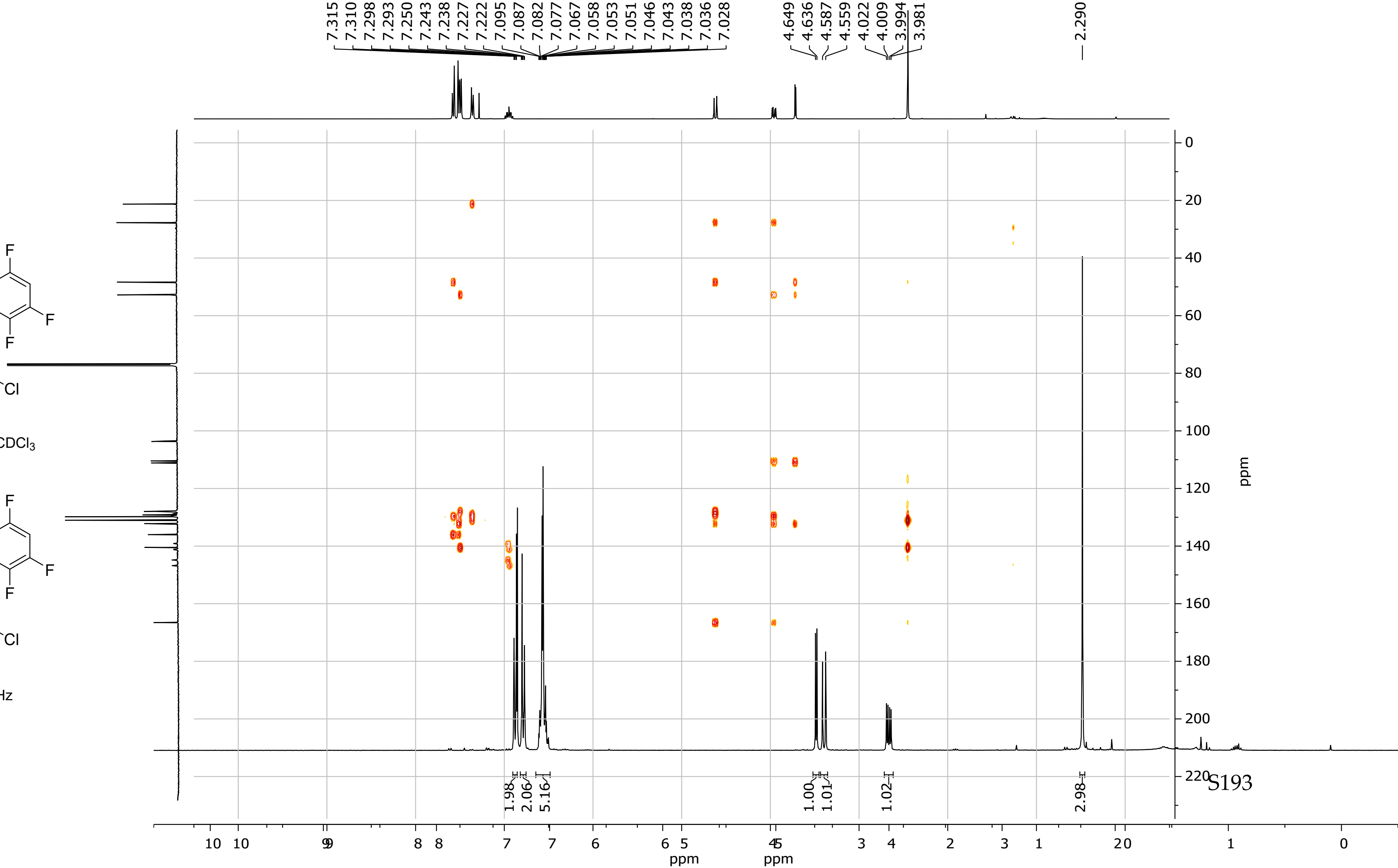

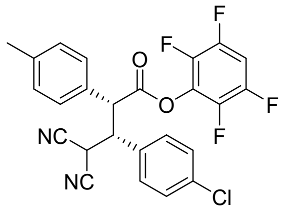

syn-**S24**
$^{19}F\{^{1}H\}$, $CDCl_3$, 376 MHz

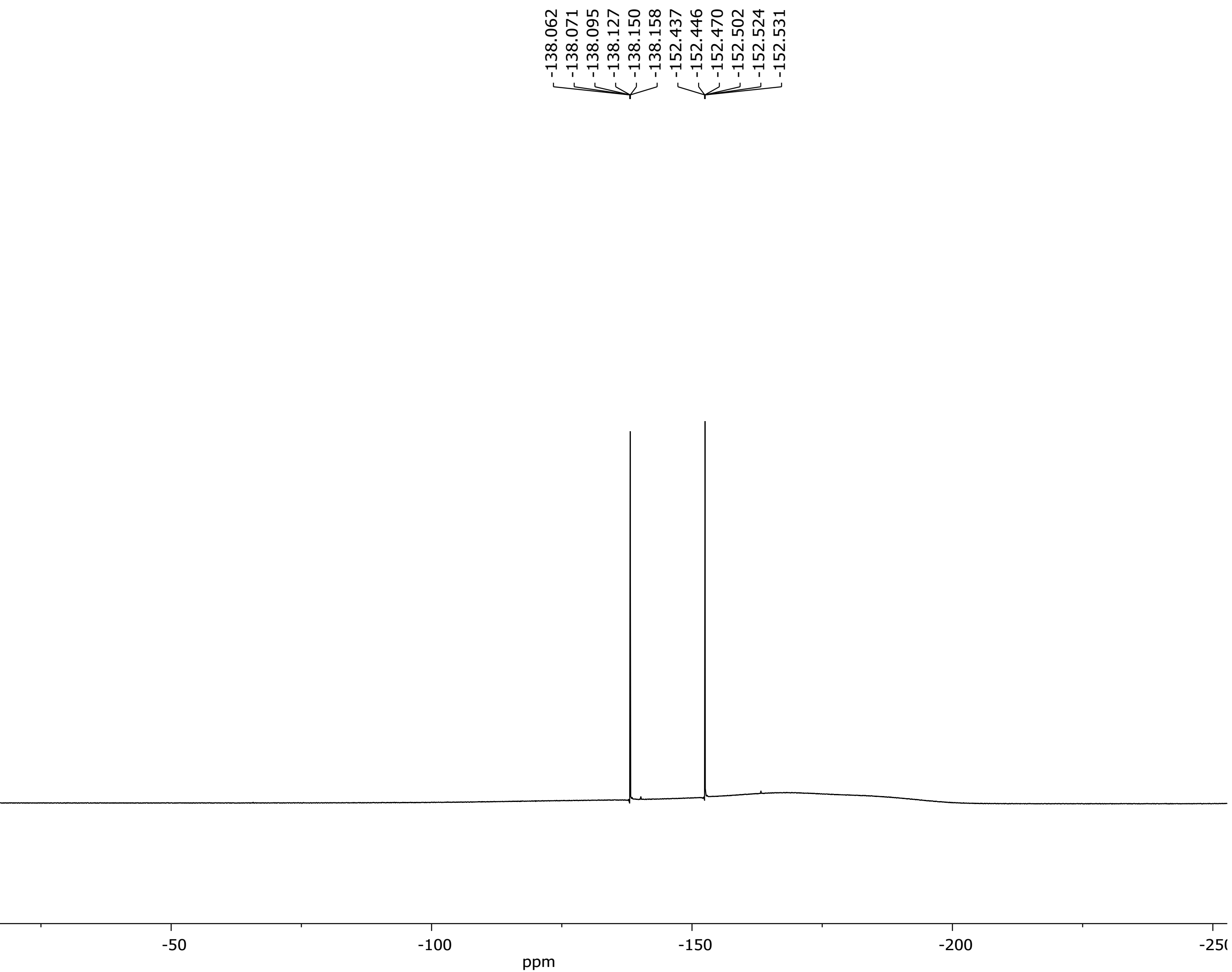

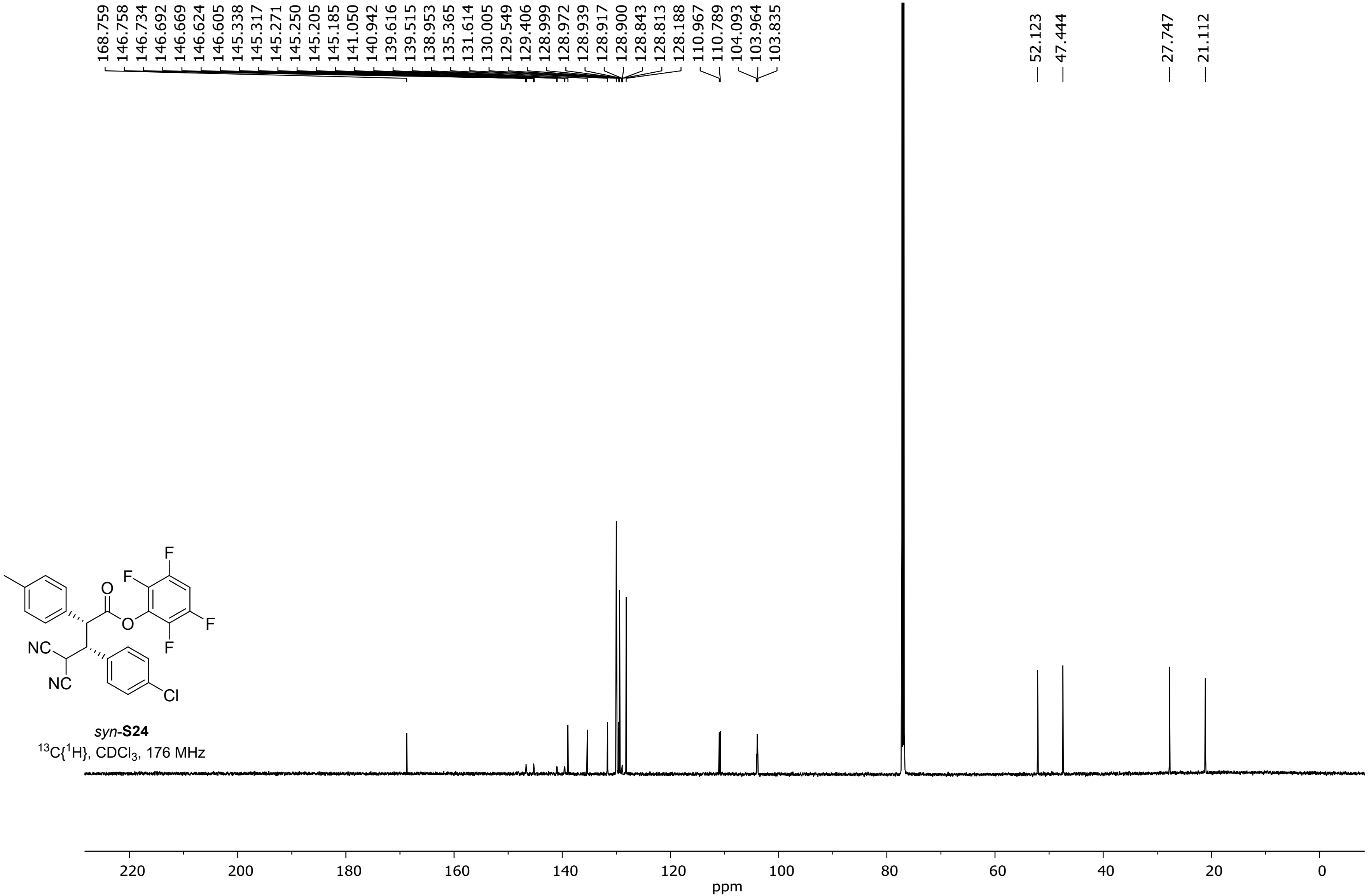

syn-**S24**
$^{13}C\{^{1}H\}$, $CDCl_3$, 176 MHz

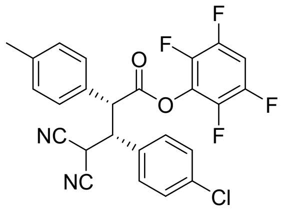

syn-**S24**
2D ^{1}H-^{1}H COSY, $CDCl_3$

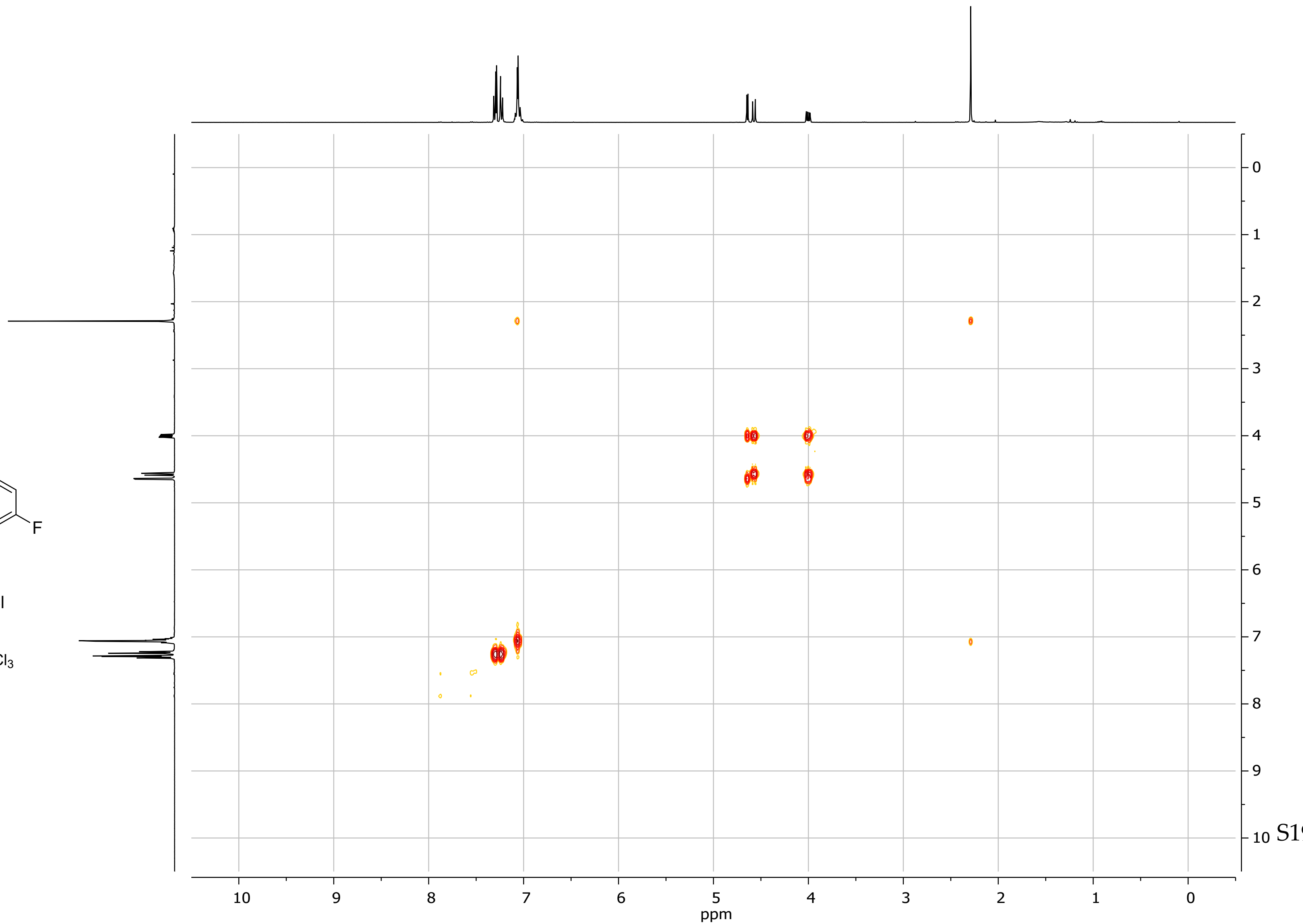

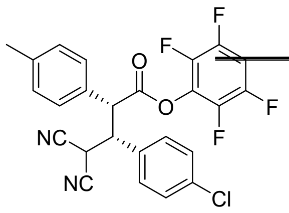

syn-**S24**
2D ^{1}H-^{13}C HSQC, $CDCl_3$

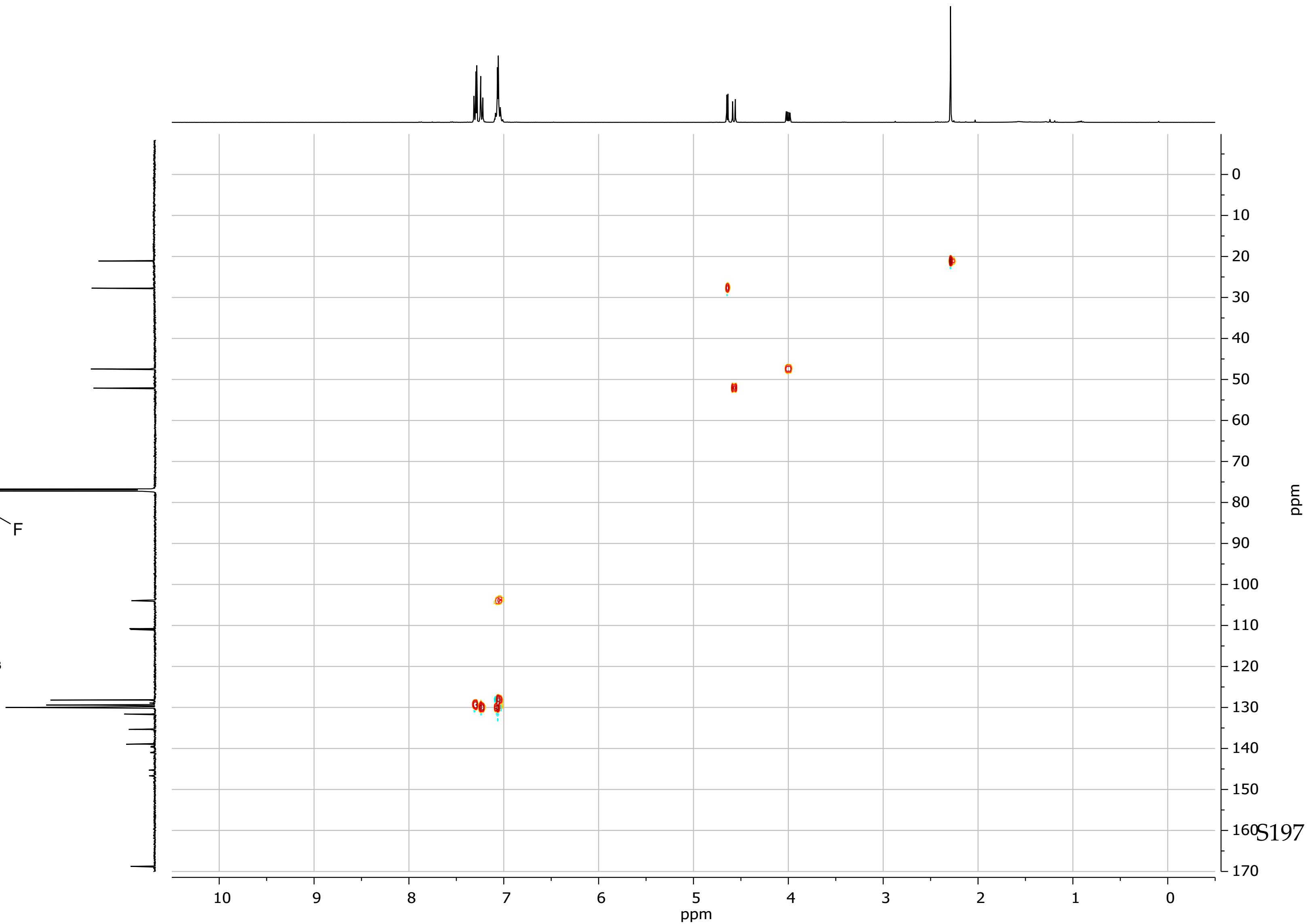

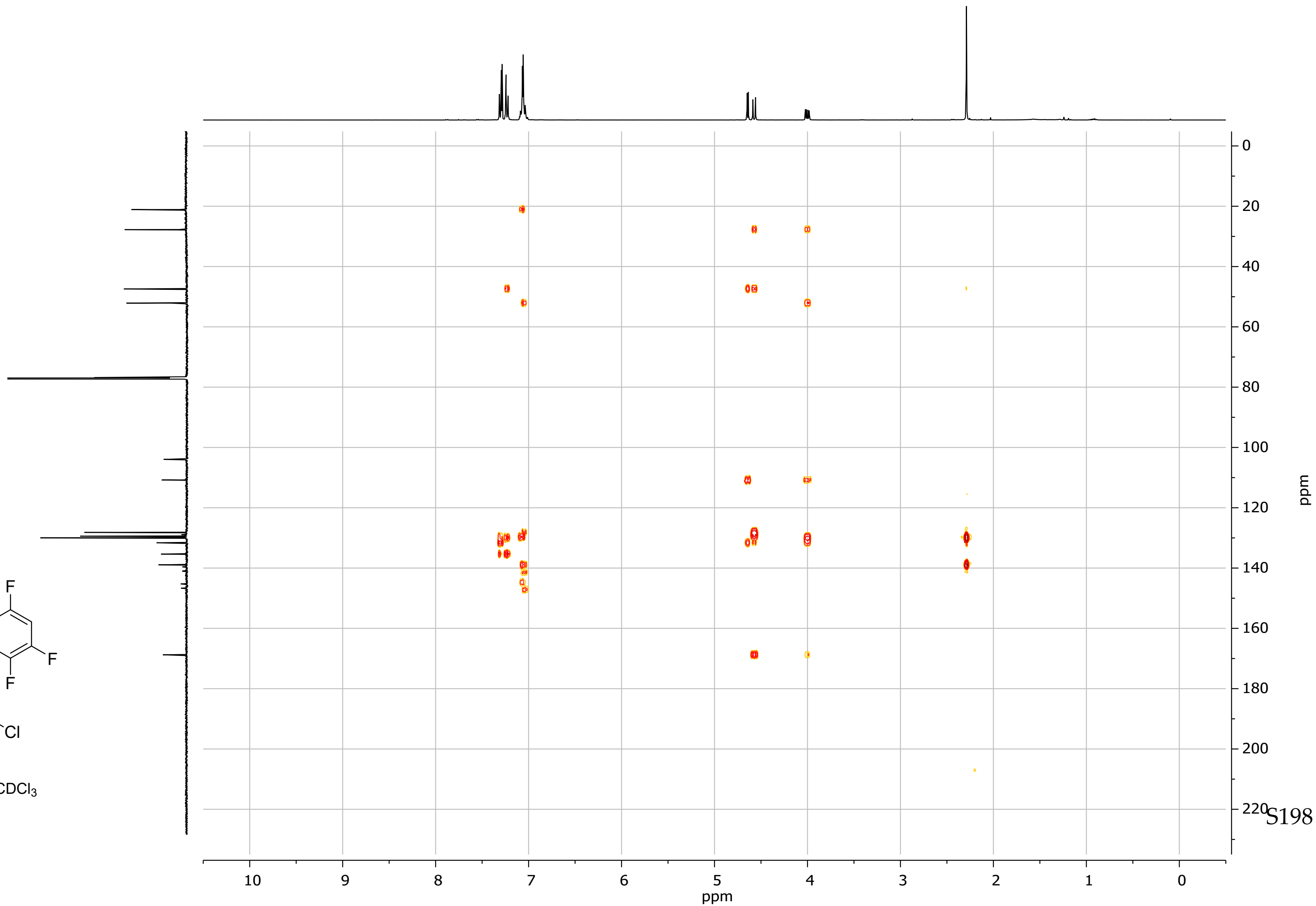

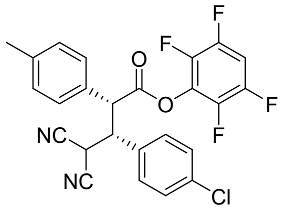

syn-**S24**
2D ^1H-^{13}C HMBC, $CDCl_3$

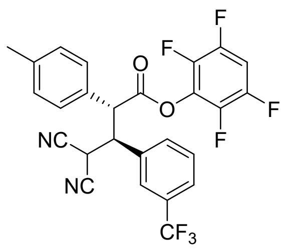

anti-**30**

^{1}H, $CDCl_3$, 400 MHz

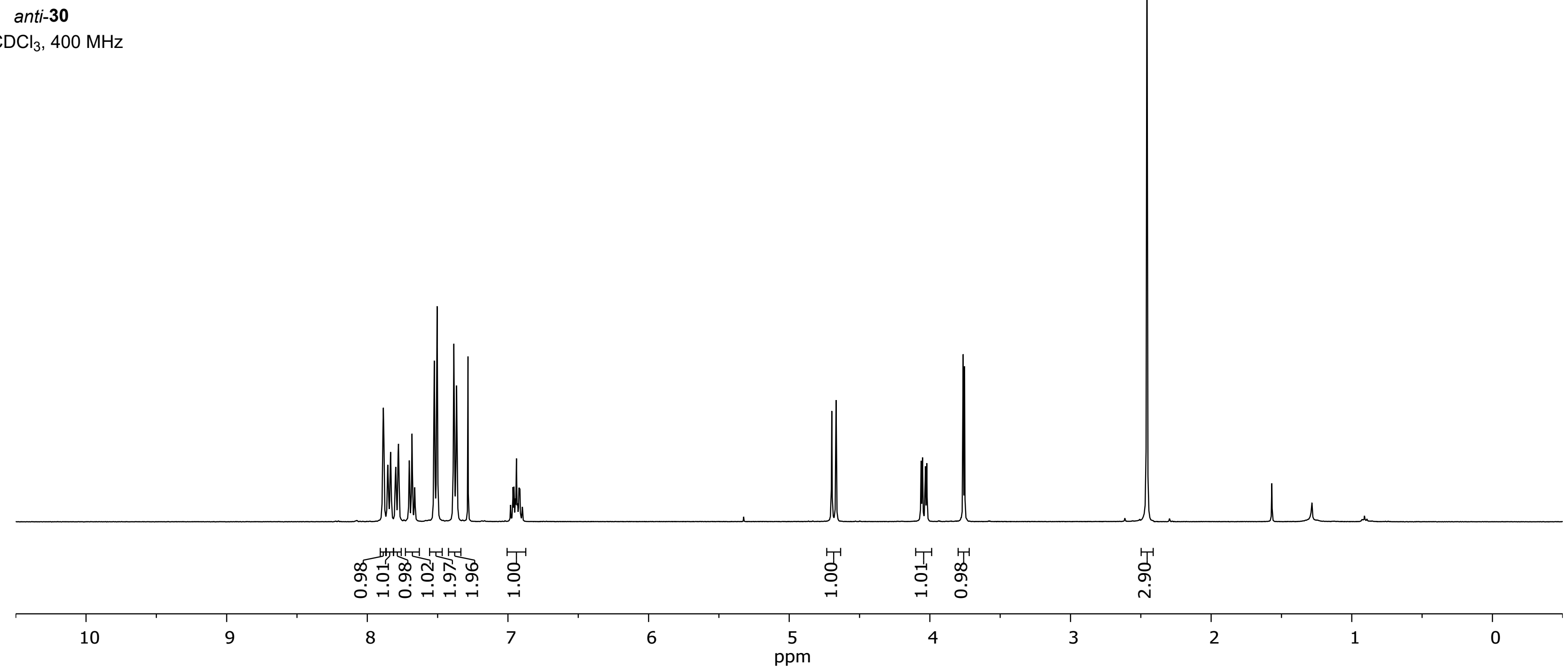

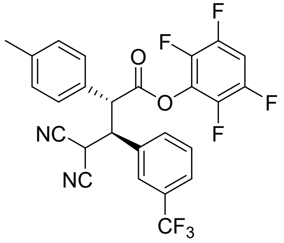

anti-**30**

$^{19}F\{^1H\}$, $CDCl_3$, 376 MHz

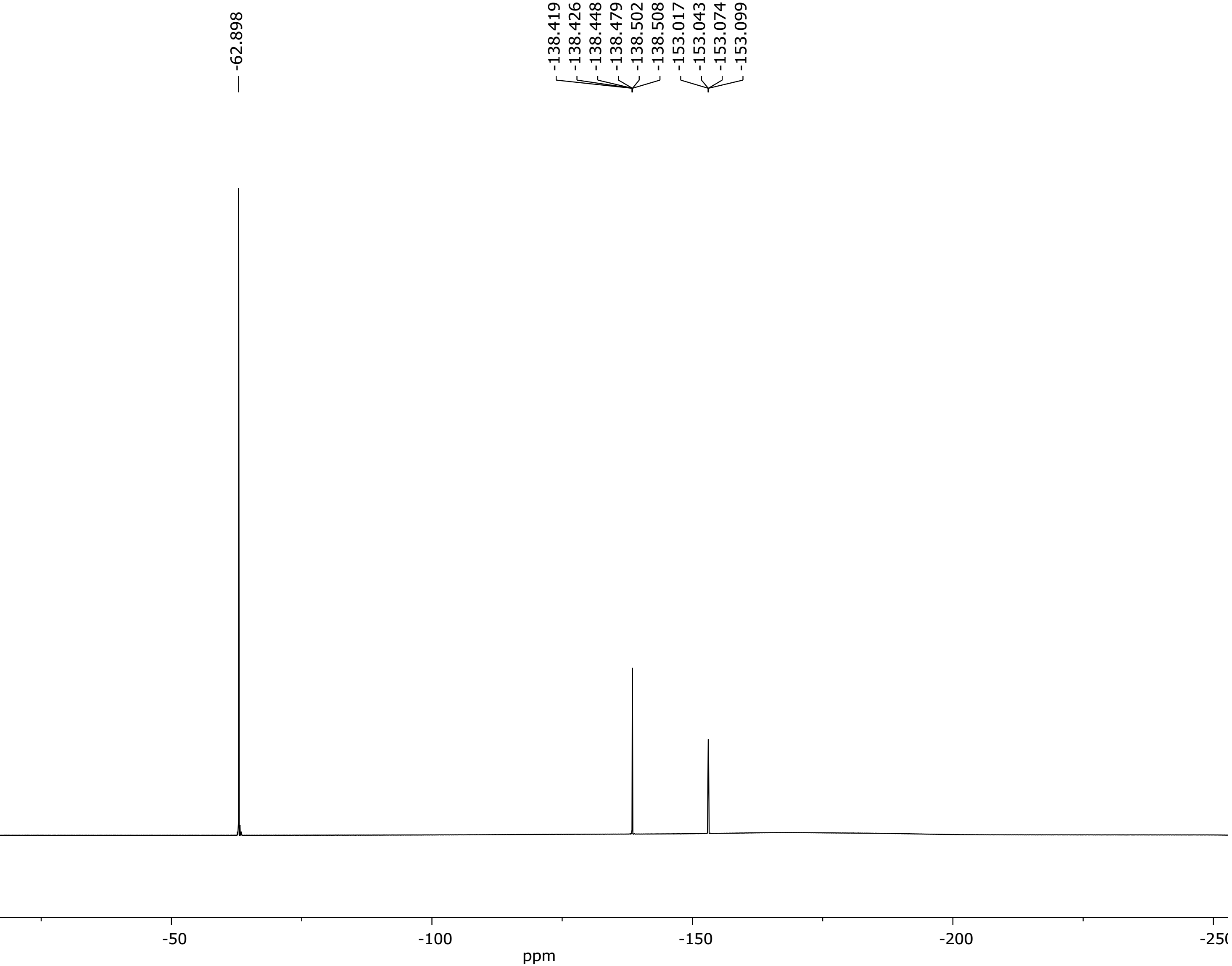

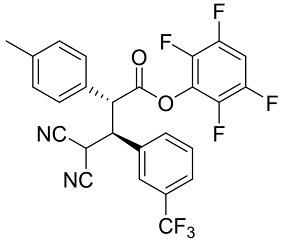

anti-**30**

$^{13}C\{^{1}H\}$, $CDCl_3$, 126 MHz

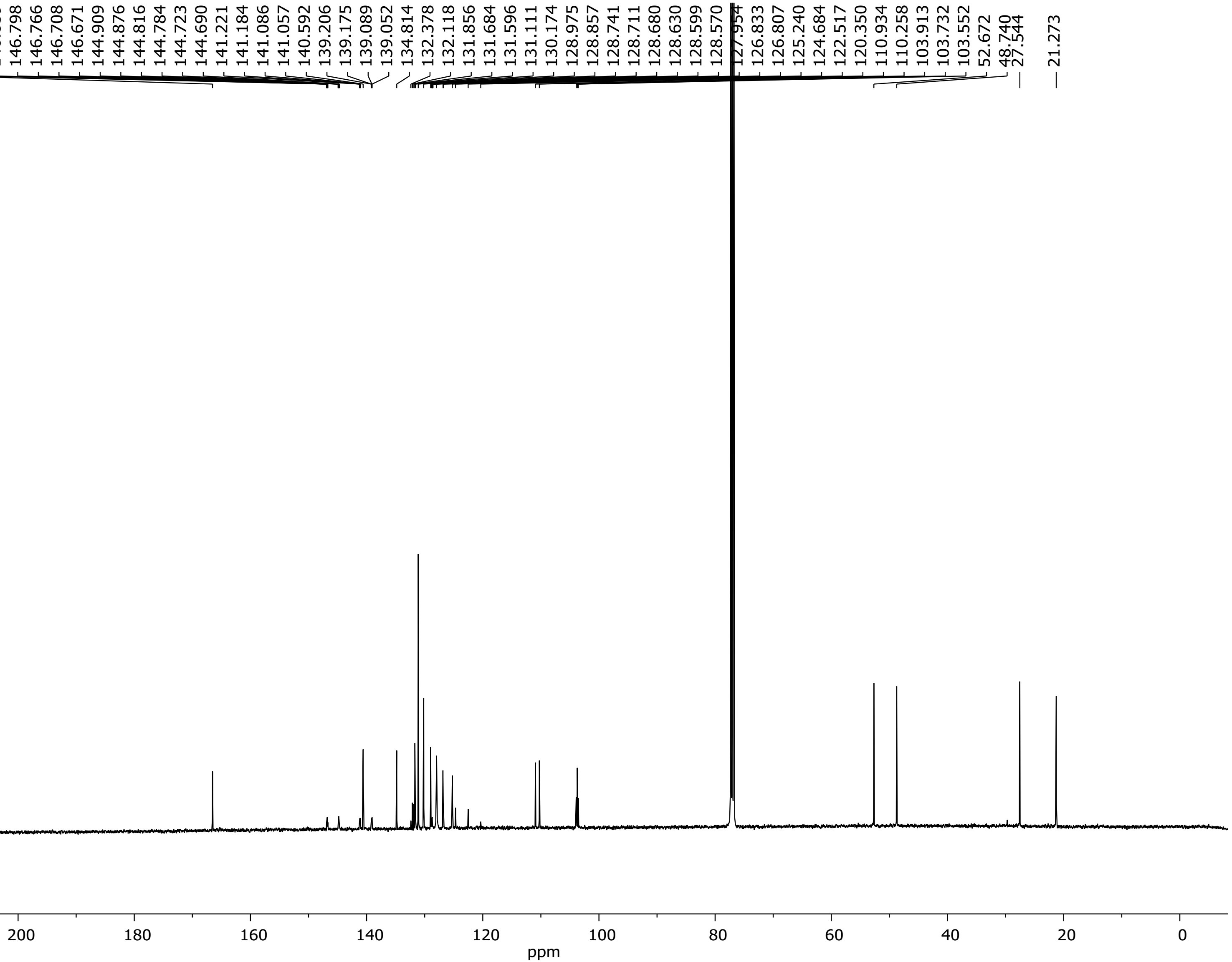

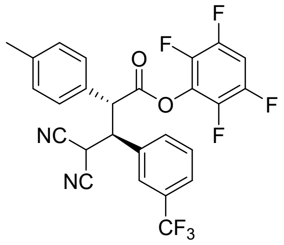

anti-**30**

2D ^{1}H-^{1}H COSY, $CDCl_3$

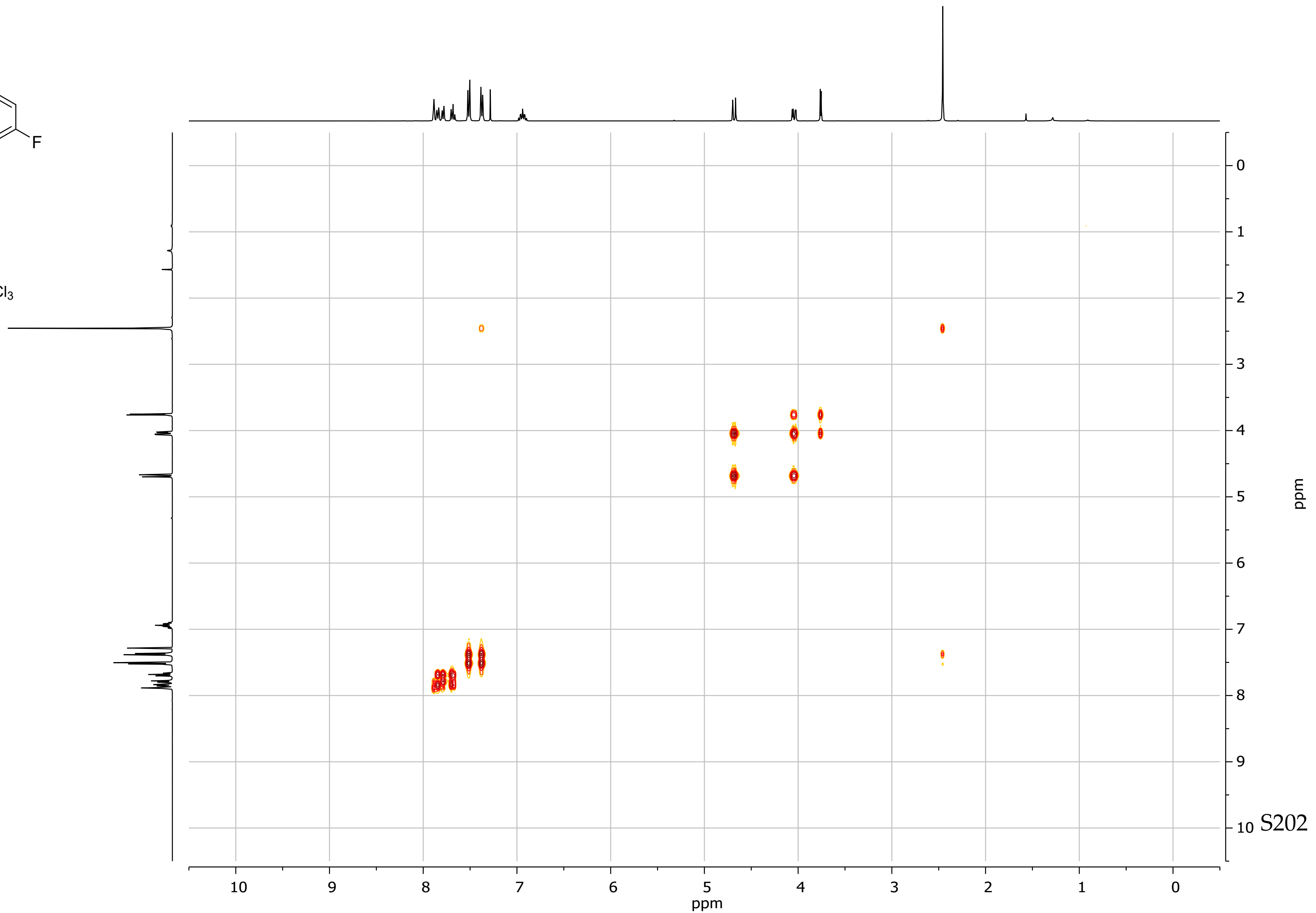

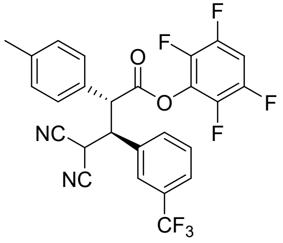

anti-**30**

2D ^{1}H-^{13}C HSQC, $CDCl_3$

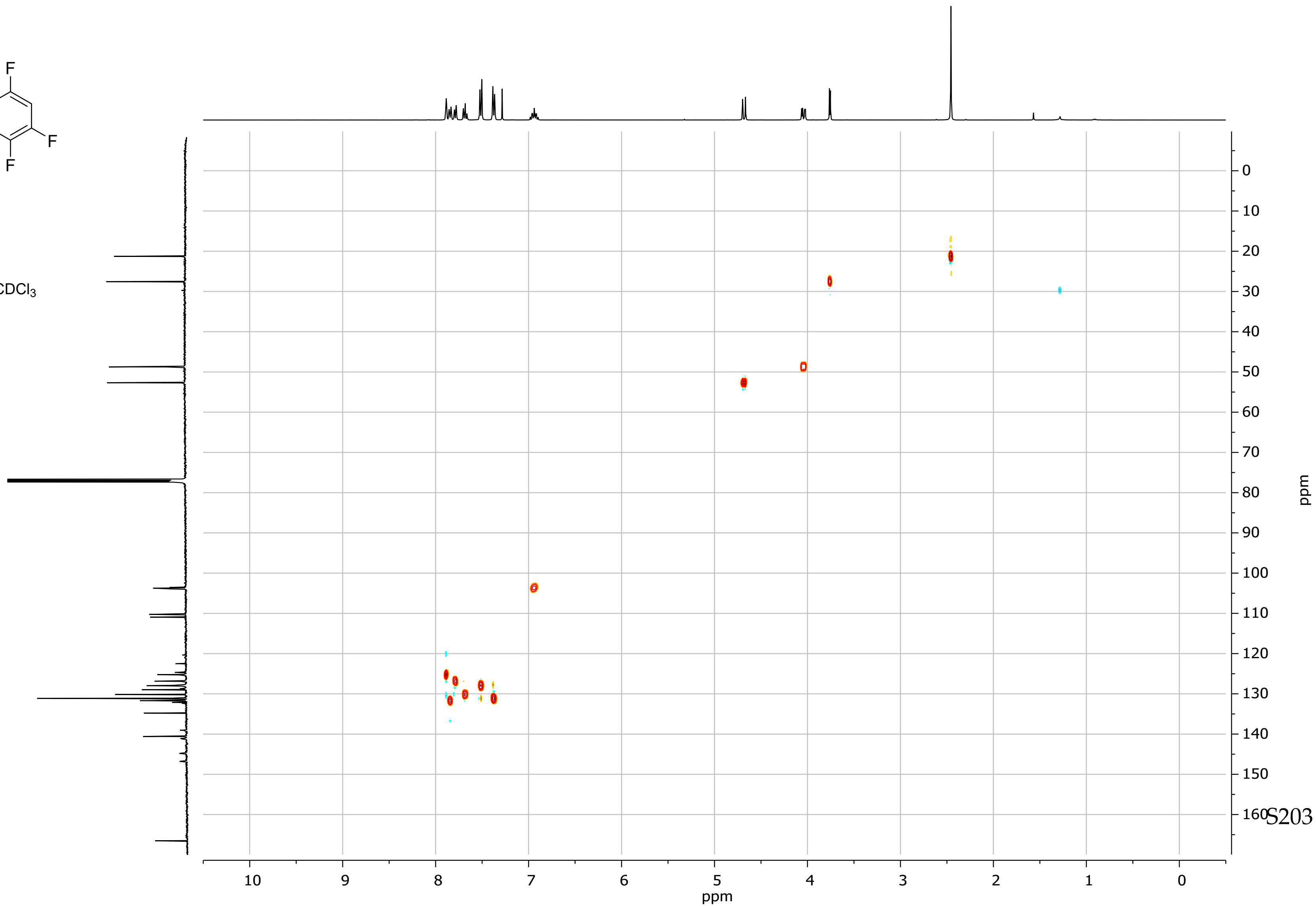

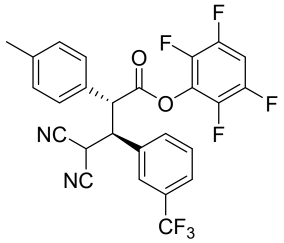

anti-**30**

2D ^{1}H-^{13}C HMBC, $CDCl_3$

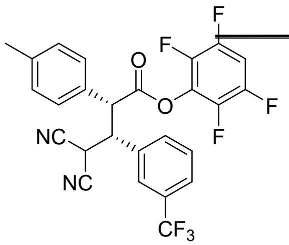

syn-**S25**

^{1}H, $CDCl_3$, 500 MHz

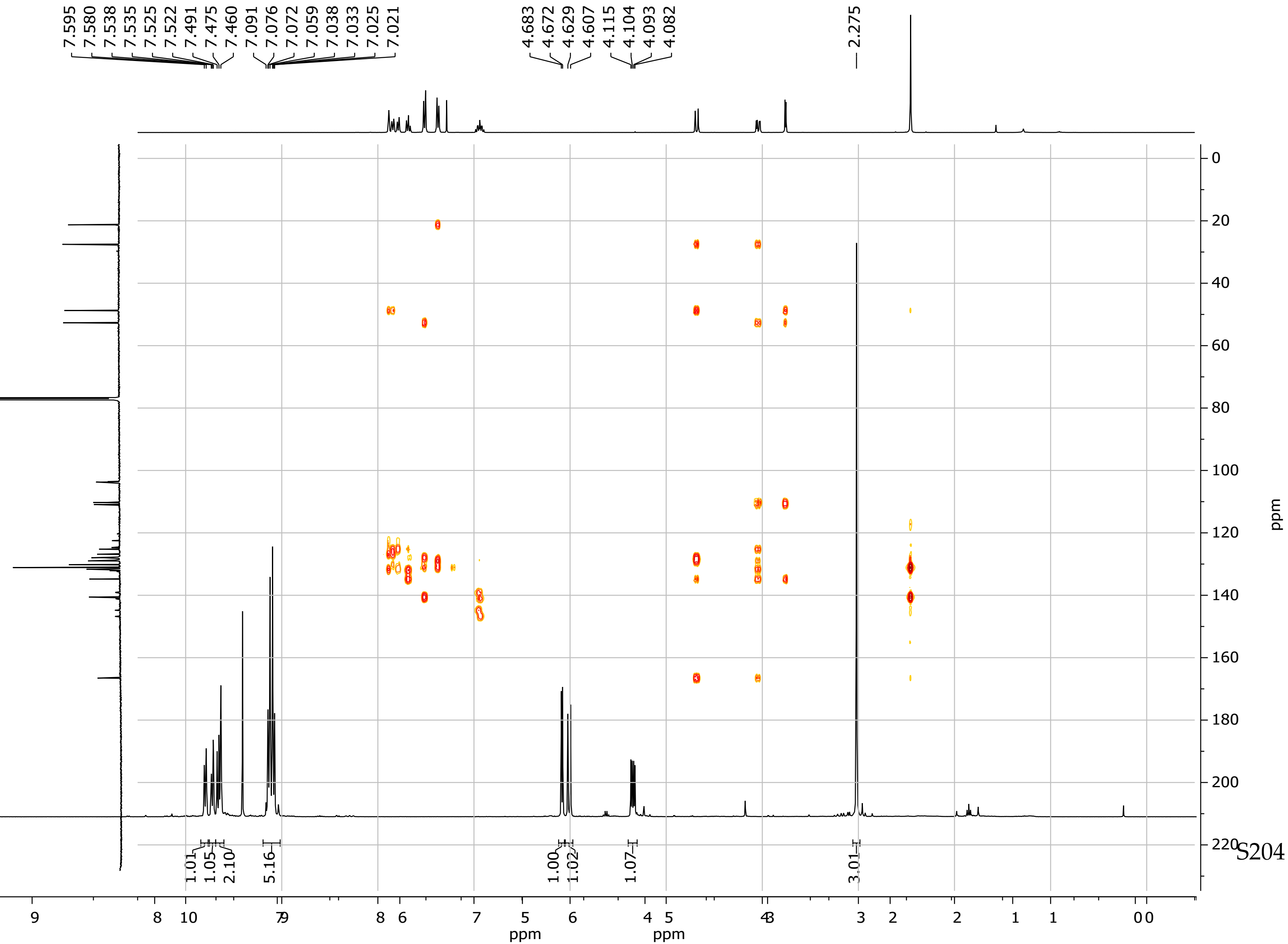

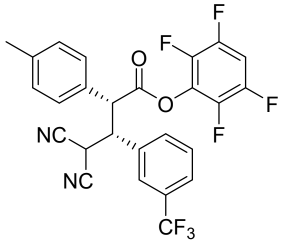

syn-**S25**

$^{19}F\{^{1}H\}$, $CDCl_3$, 376 MHz

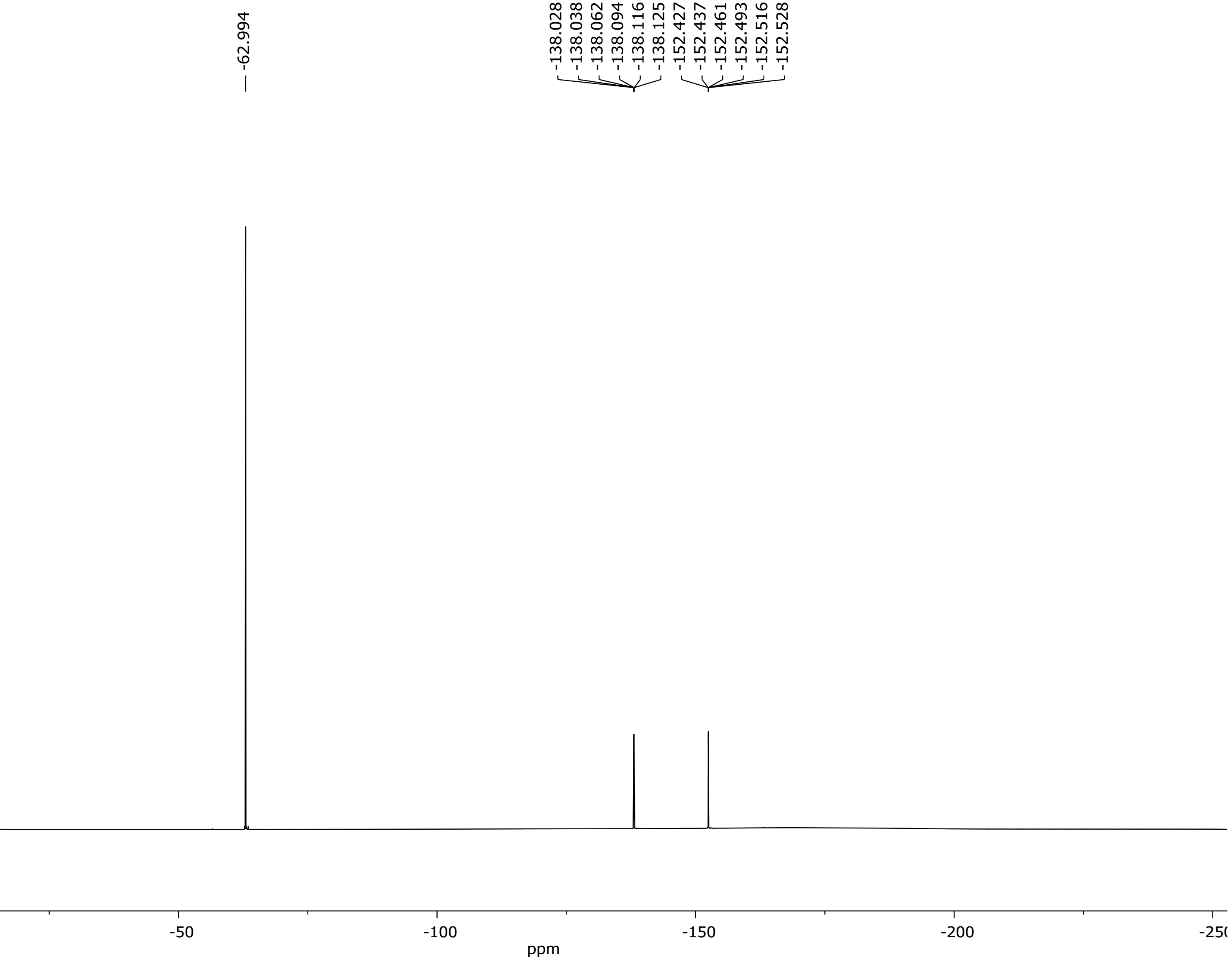

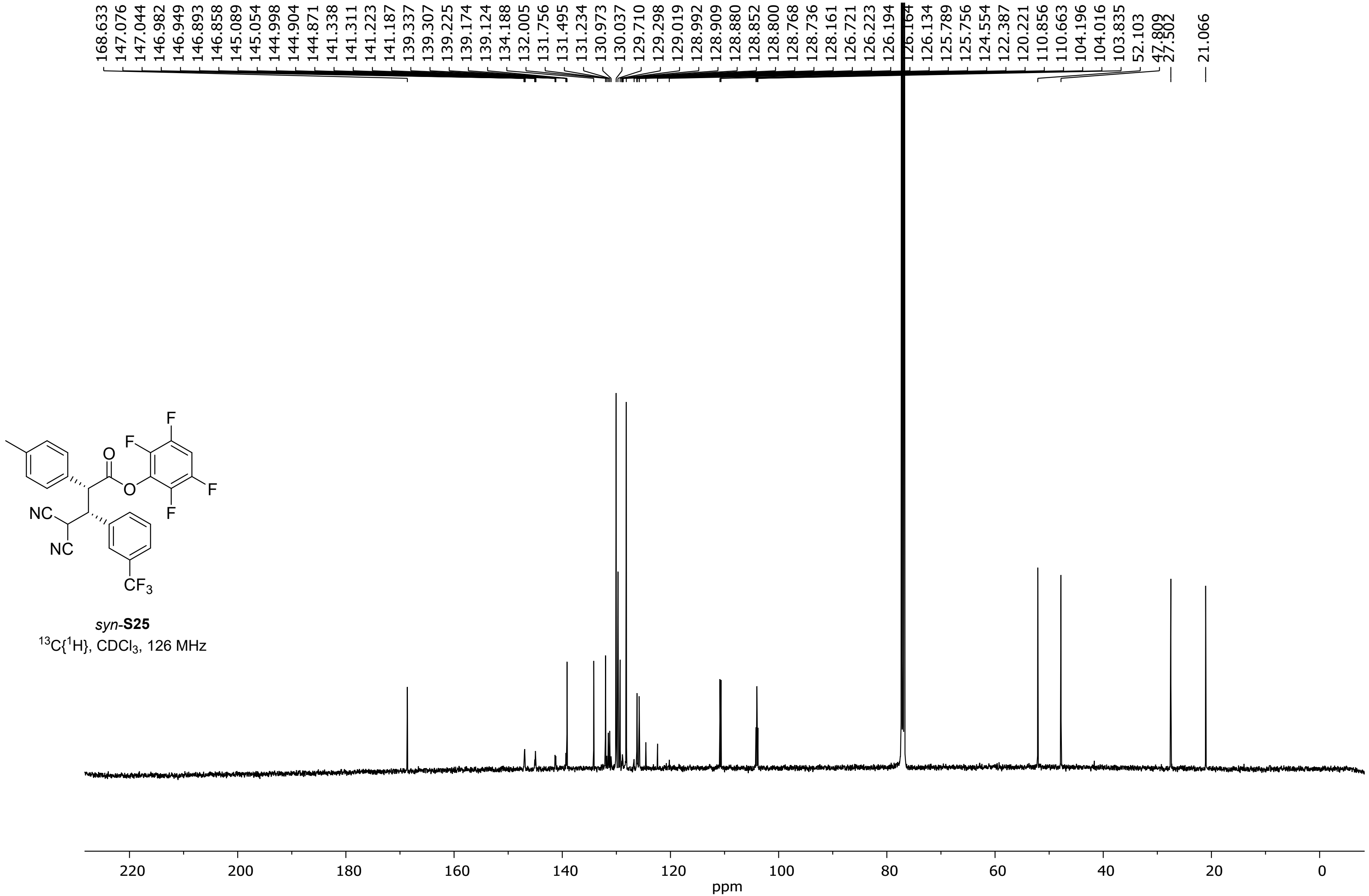

syn-**S25**

$^{13}C\{^{1}H\}$, $CDCl_3$, 126 MHz

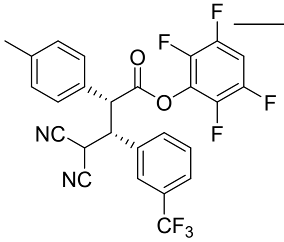

syn-**S25**

2D ^1H-^1H COSY, $CDCl_3$

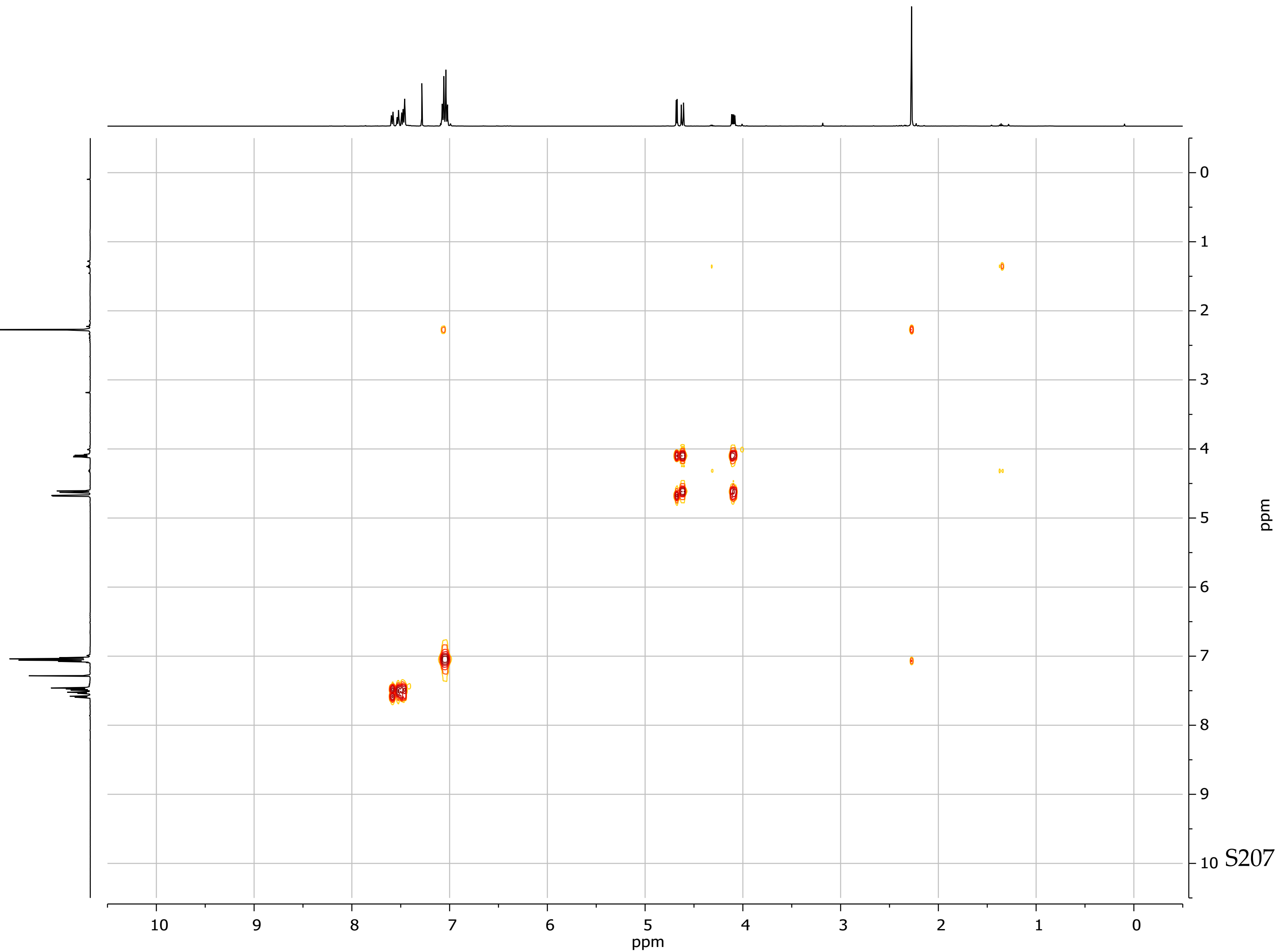

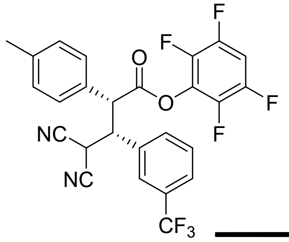

syn-**S25**

2D ^{1}H-^{13}C HSQC, $CDCl_3$

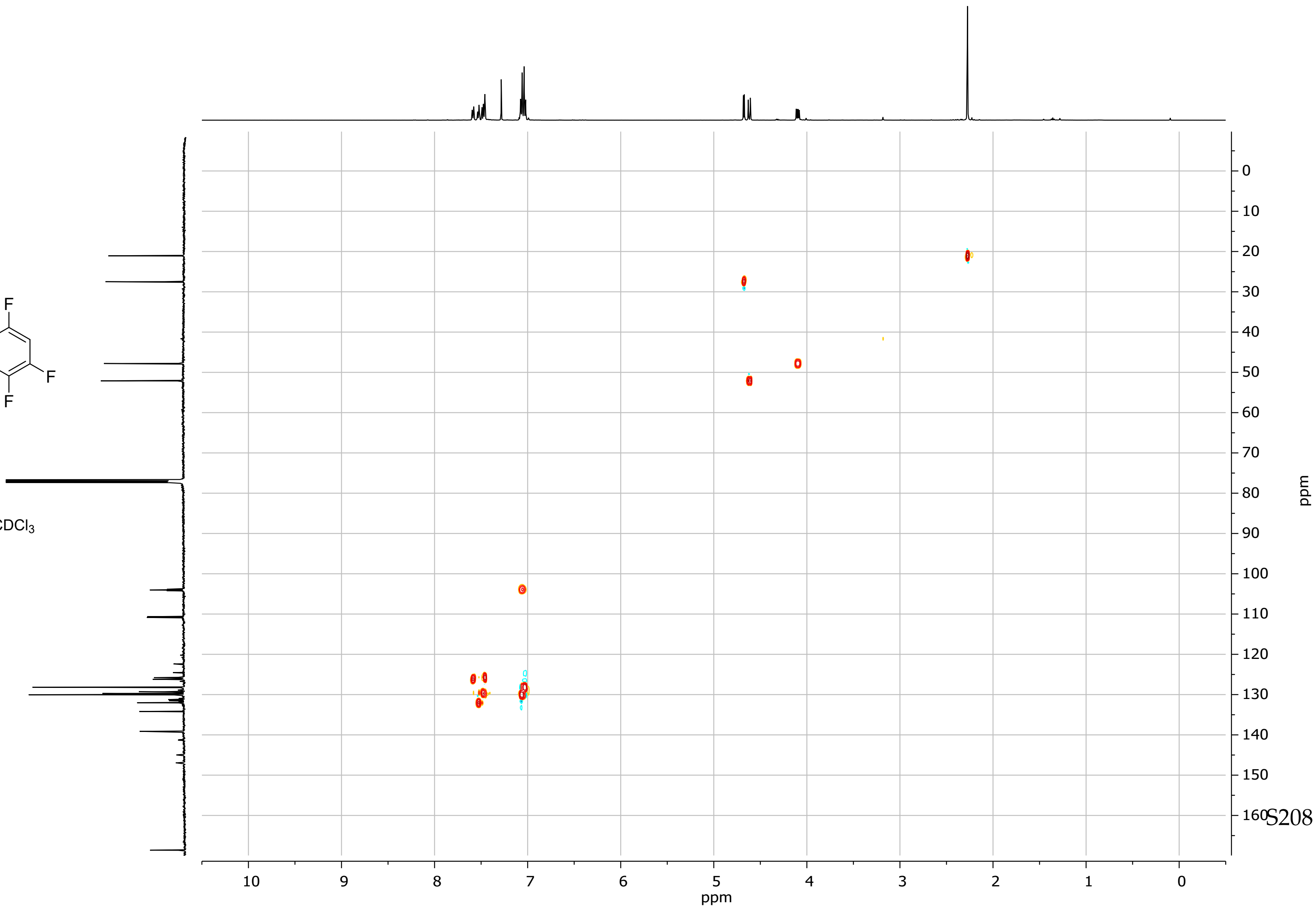

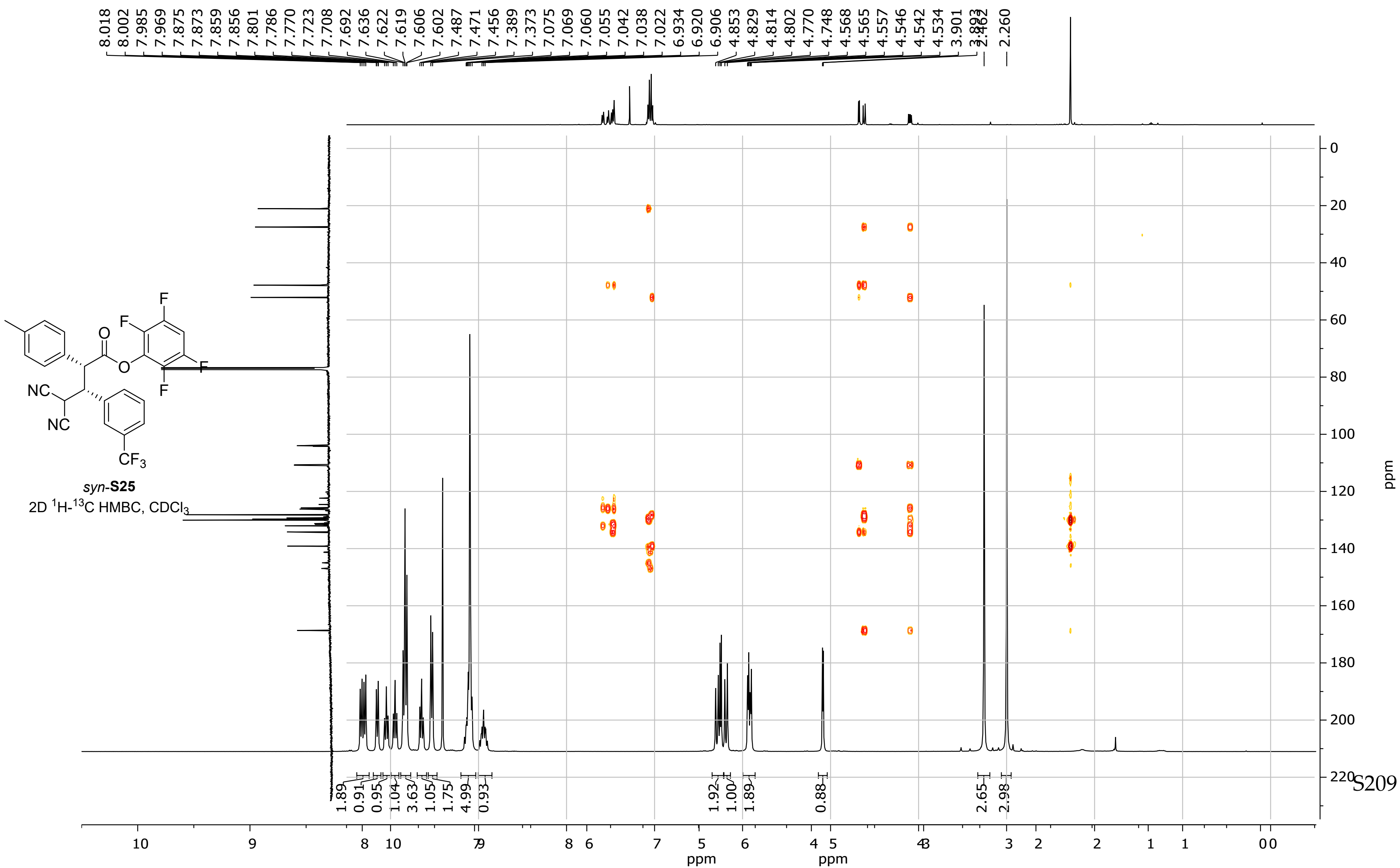

syn-**S25**

2D ^{1}H-^{13}C HMBC, $CDCl_3$

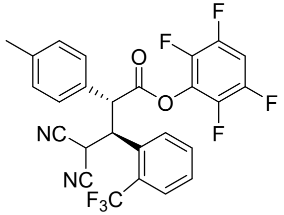

anti-**31**

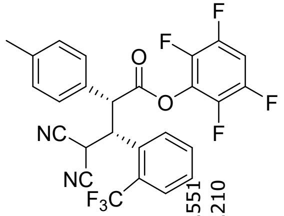

syn-**S26**

^{1}H, CDCl$_3$, 500 MHz

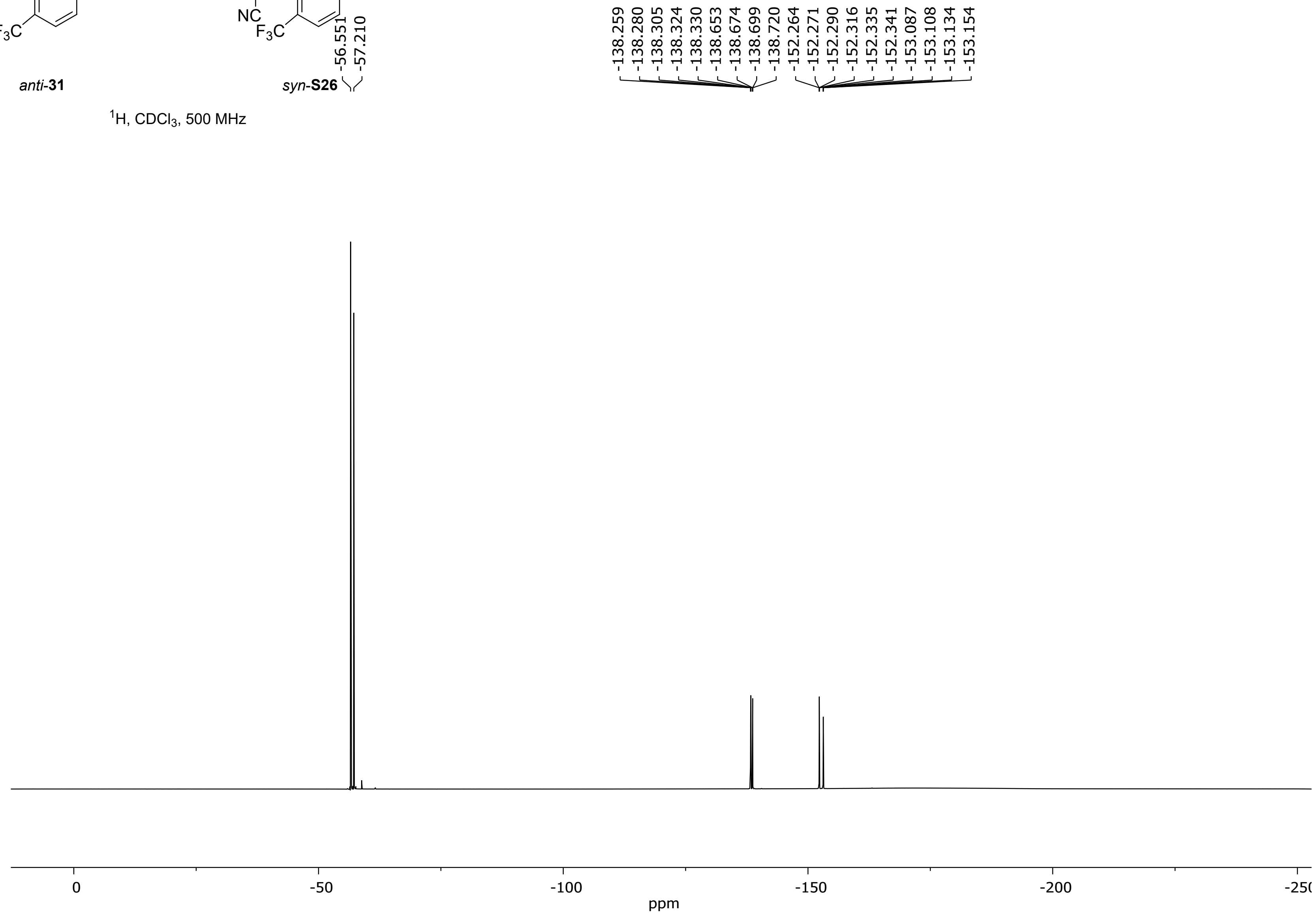

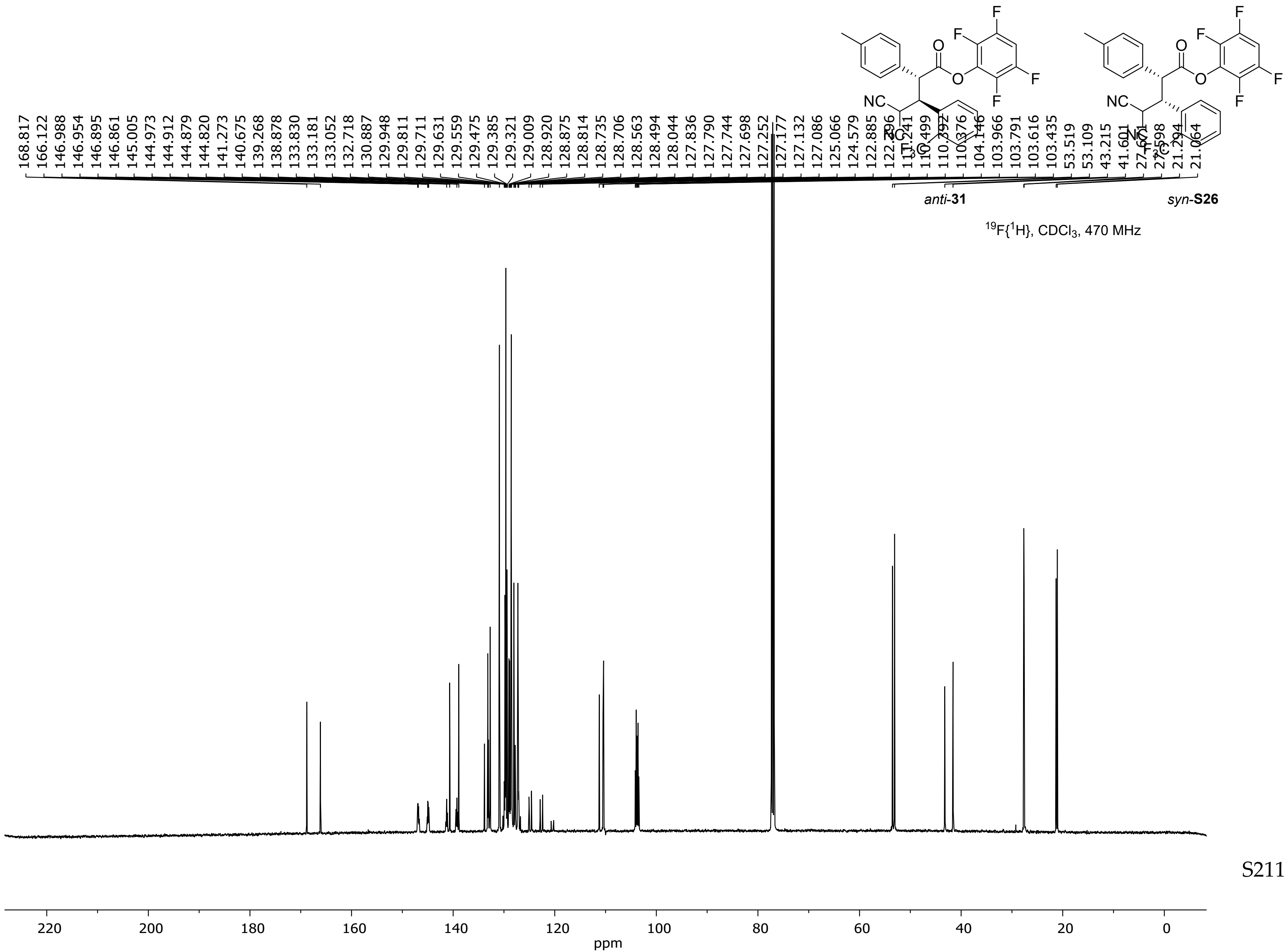

168.817
166.122
146.988
146.954
146.895
146.861
145.005
144.973
144.912
144.879
144.820
141.273
140.675
139.268
138.878
133.830
133.181
133.052
132.718
130.887
129.948
129.811
129.711
129.631
129.559
129.475
129.385
129.321
129.009
128.920
128.875
128.814
128.735
128.706
128.563
128.494
128.044
127.836
127.790
127.744
127.698
127.252
127.177
127.132
127.086
125.066
124.579
122.885
110.499
110.376
104.146
103.966
103.791
103.616
103.435
53.519
53.109
43.215
41.601
21.294
21.064
NC
O
O
F
F
F
F
anti-31
syn-S26
$^{19}F\{^{1}H\}$, $CDCl_3$, 470 MHz
220
200
180
160
140
120
100
80
60
40
20
0
ppm

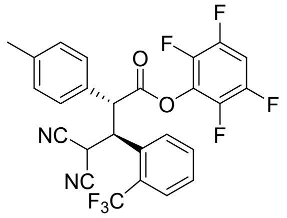

anti-**31**

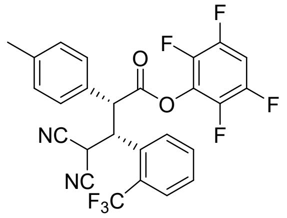

syn-**S26**

$^{13}C\{^{1}H\}$, $CDCl_3$, 126 MHz

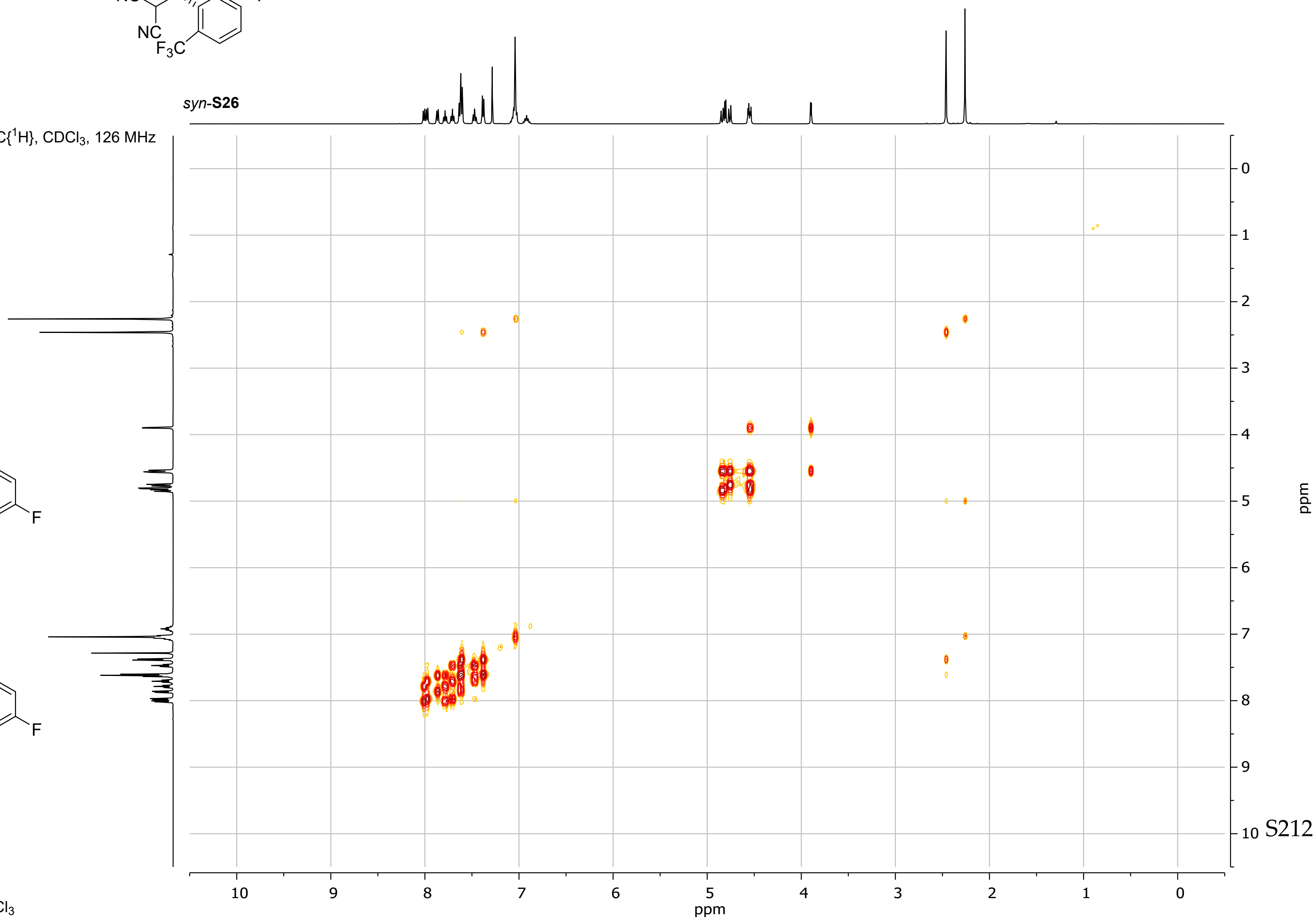

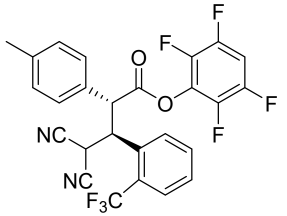

anti-**31**

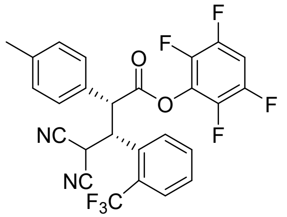

syn-**S26**

2D ^{1}H-^{1}H COSY, $CDCl_3$

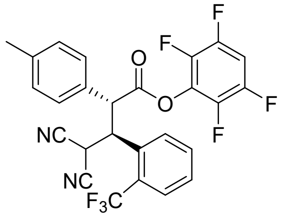

anti-**31**

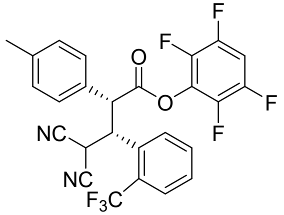

syn-**S26**

2D ^1H-^{13}C HSQC, $CDCl_3$

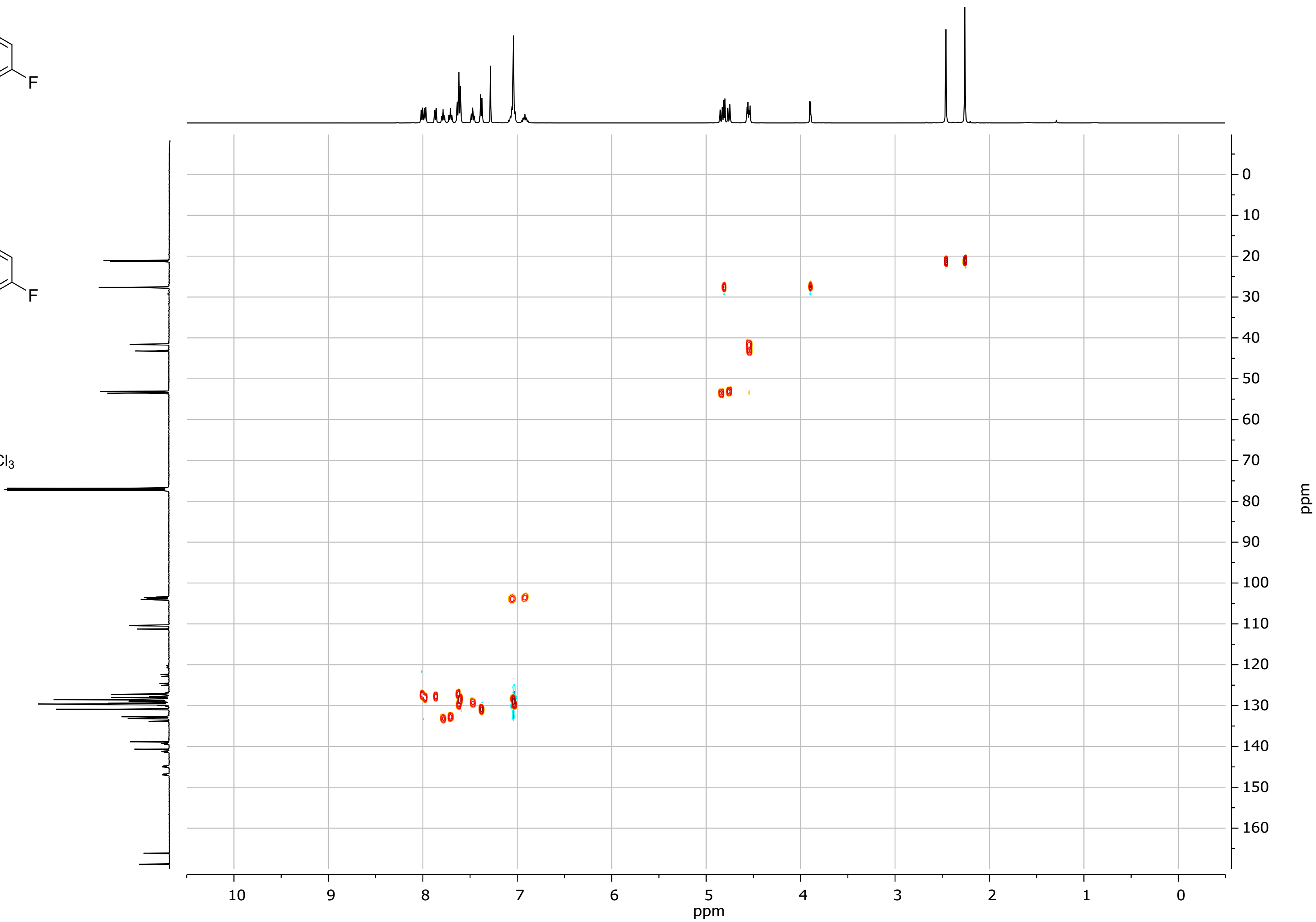

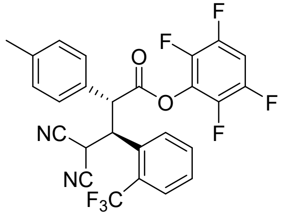

anti-**S1**

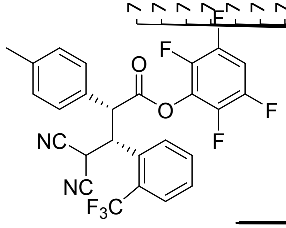

syn-**S26**

2D ^{1}H-^{13}C HMBC, $CDCl_3$

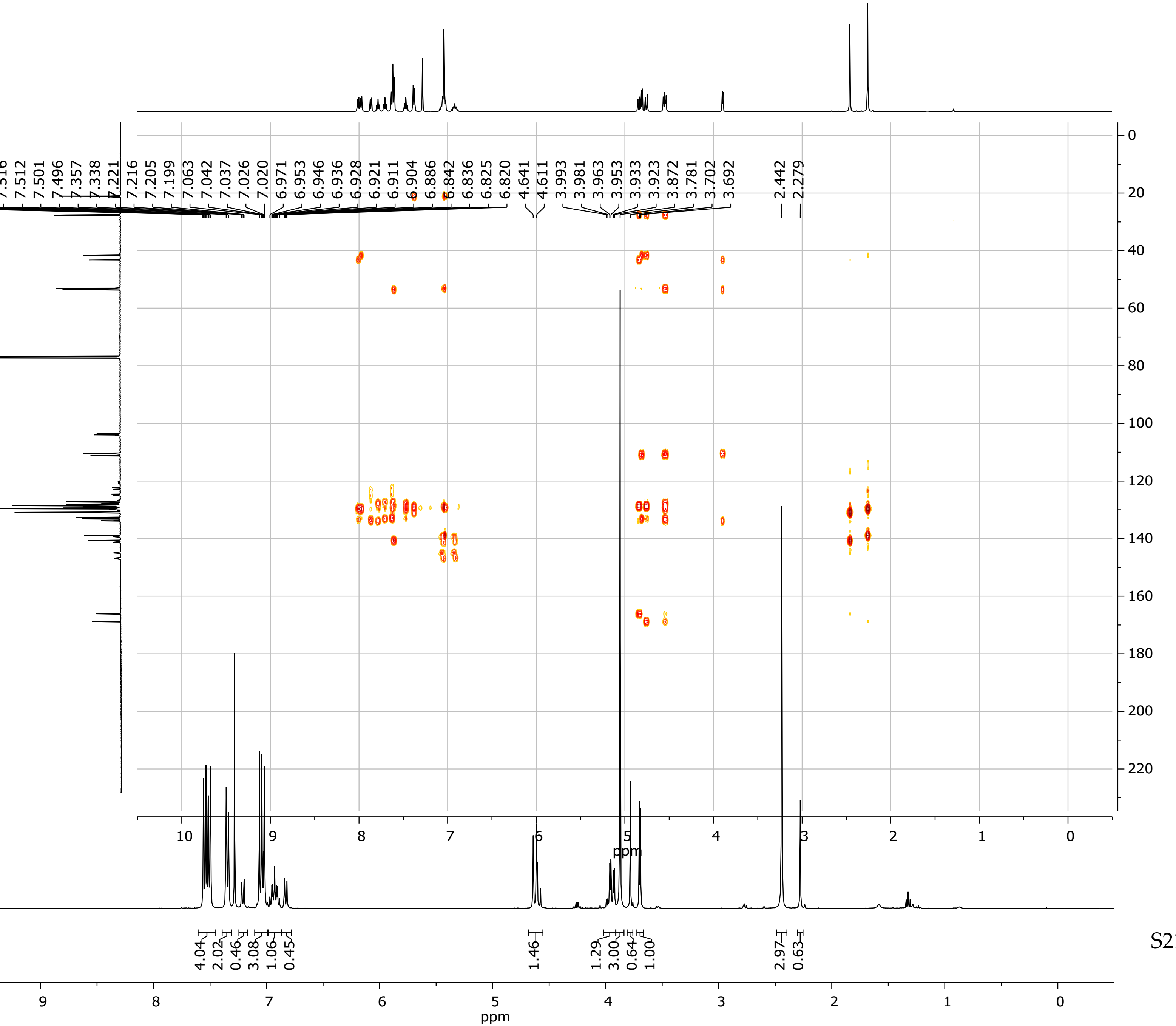

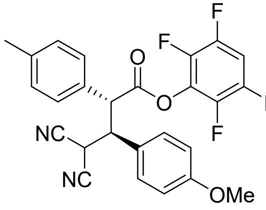

anti-**32**

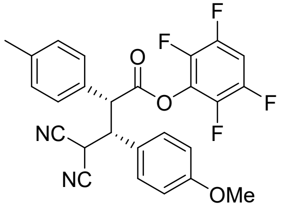

syn-**S27**

^{1}H, $CDCl_3$, 400 MHz

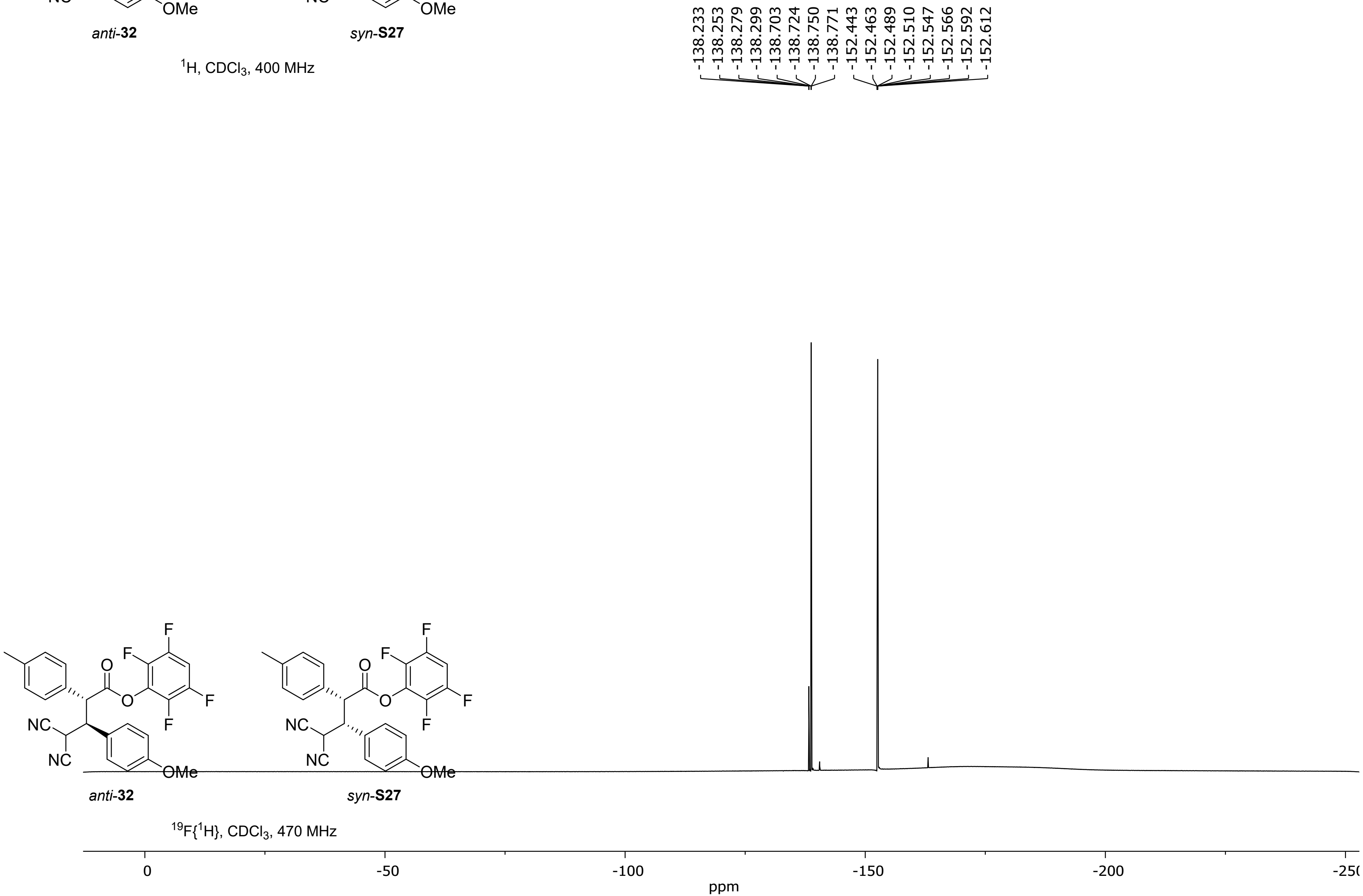

$^{19}F\{^{1}H\}$, $CDCl_3$, 470 MHz

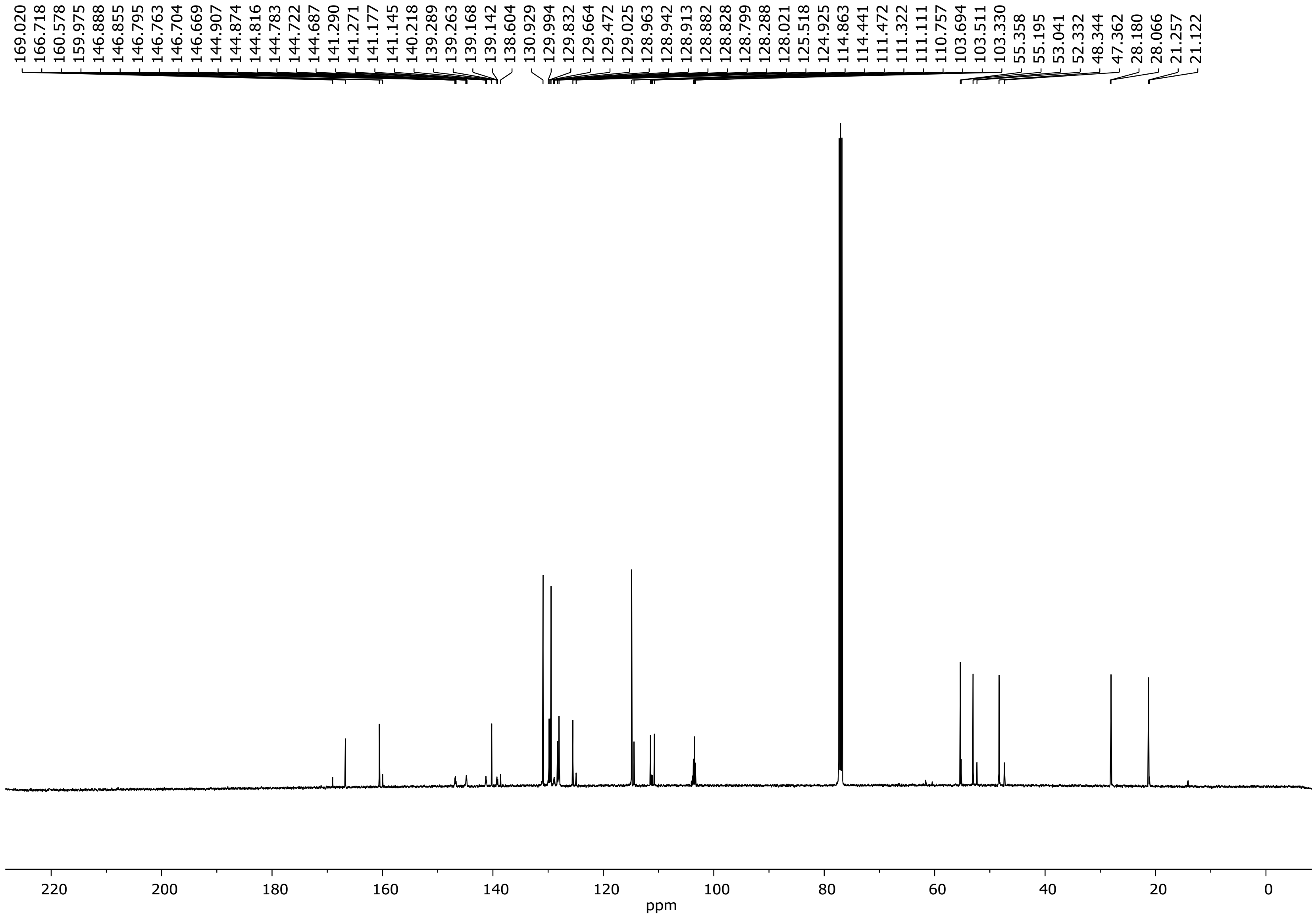
169.020
166.718
160.578
159.975
146.888
146.855
146.795
146.763
146.704
146.669
144.907
144.874
144.816
144.783
144.722
144.687
141.290
141.271
141.177
141.145
140.218
139.289
139.263
139.168
139.142
138.604
130.929
129.994
129.832
129.664
129.472
129.025
128.963
128.942
128.913
128.882
128.828
128.799
128.288
128.021
125.518
124.925
114.863
114.441
111.472
111.322
111.111
110.757
103.694
103.511
103.330
55.358
55.195
53.041
52.332
48.344
47.362
28.180
28.066
21.257
21.122
220
200
180
160
140
120
100
80
60
40
20
0
ppm

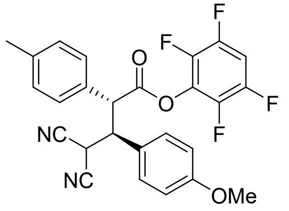

anti-**32**

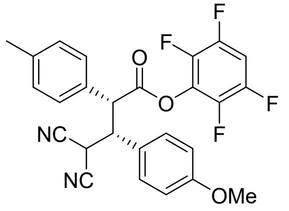

syn-**S27**

$^{13}C\{^{1}H\}$, $CDCl_3$, 126 MHz

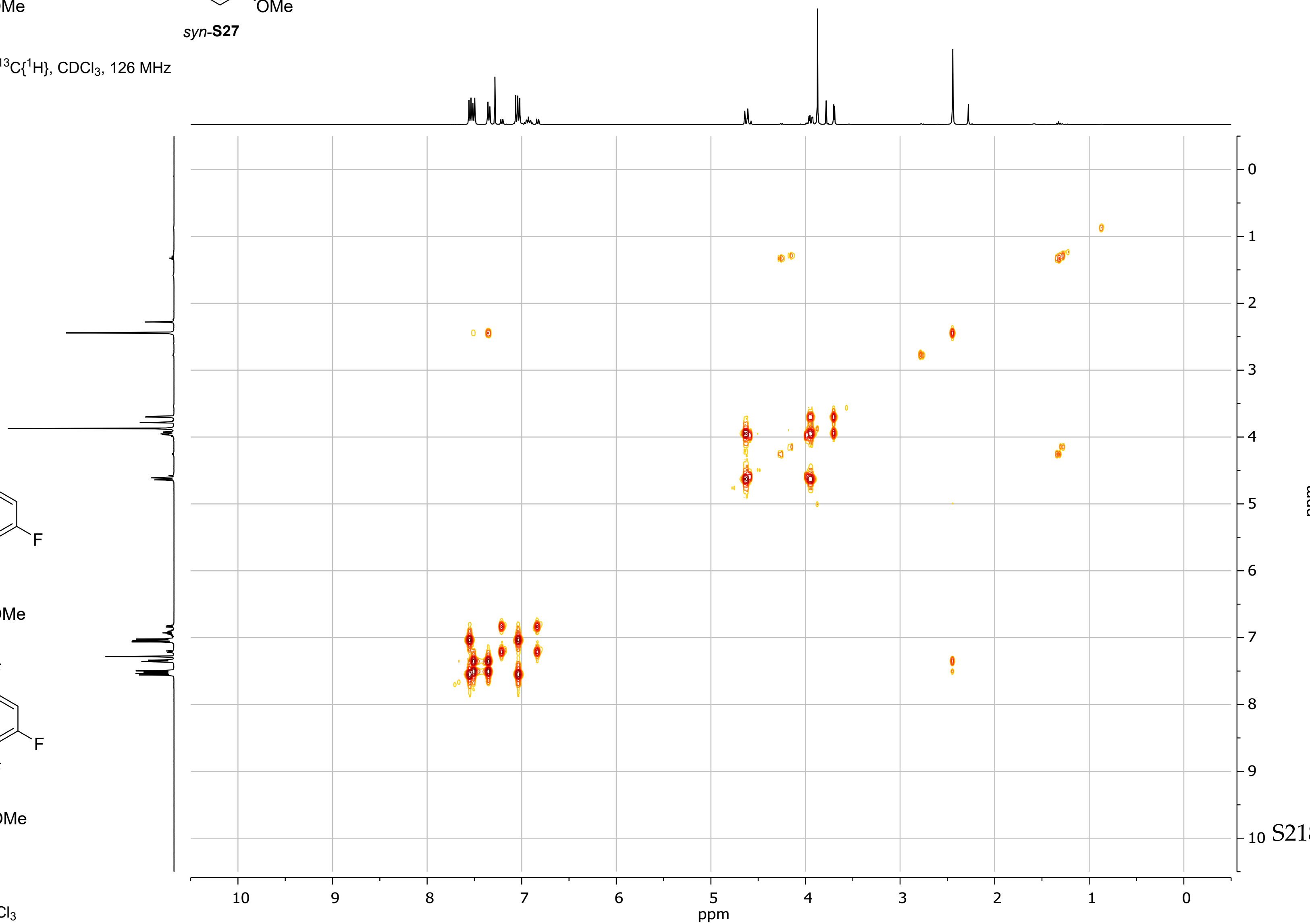

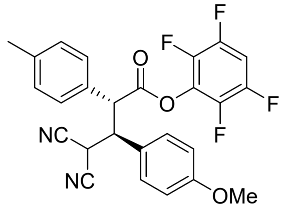

anti-**32**

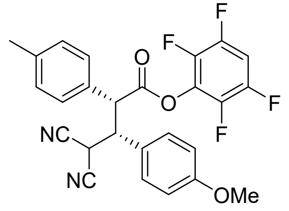

syn-**S27**

2D ^{1}H-^{1}H COSY, $CDCl_3$

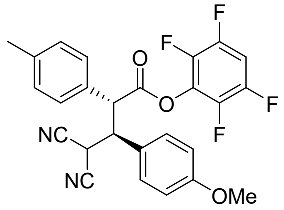

anti-**32**

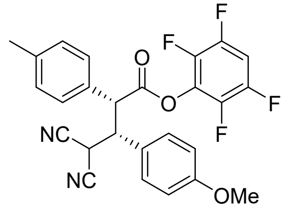

syn-**S27**

2D ^{1}H-^{13}C HSQC, $CDCl_3$

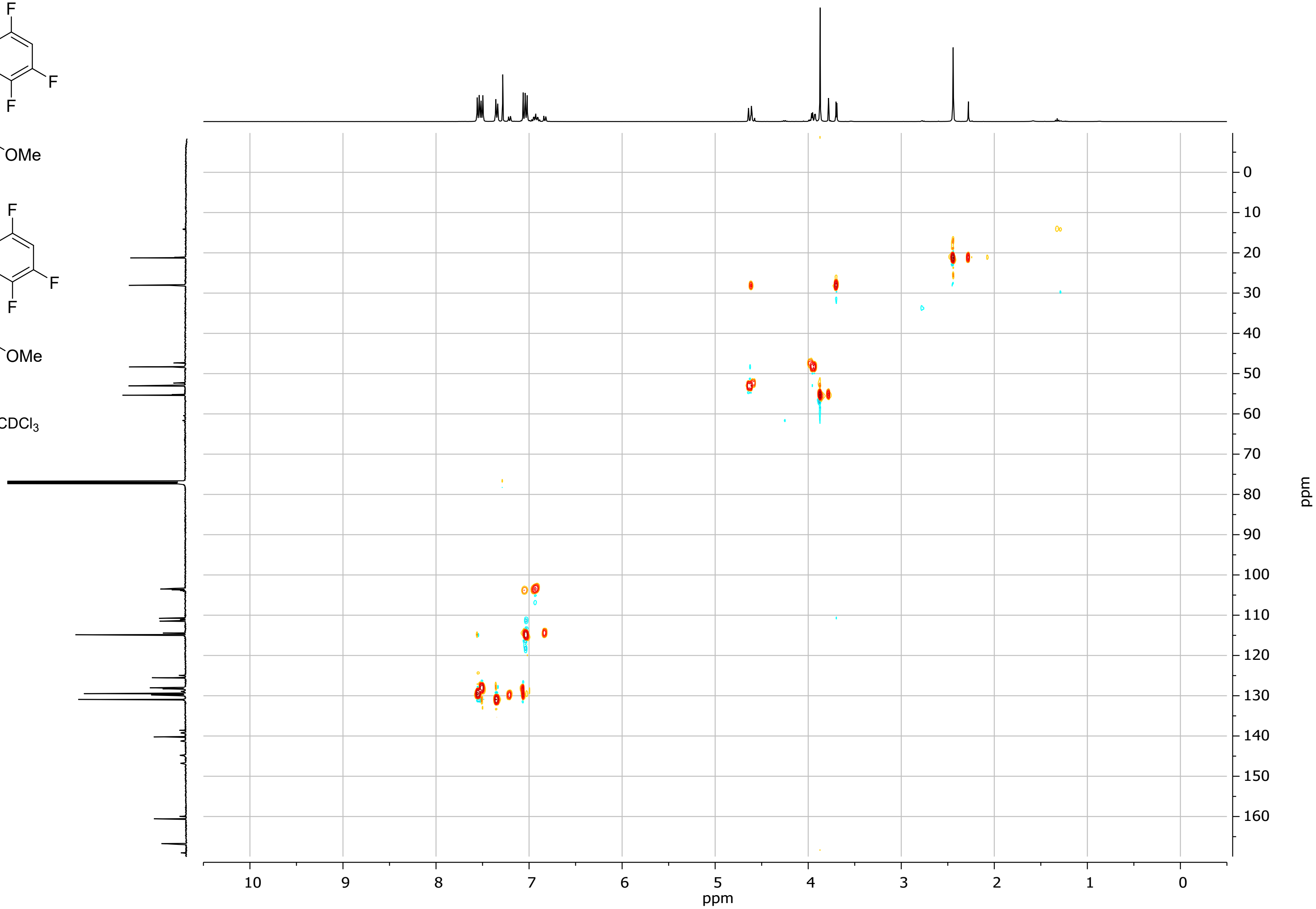

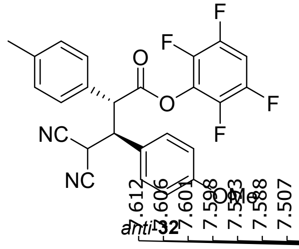

anti-**32**

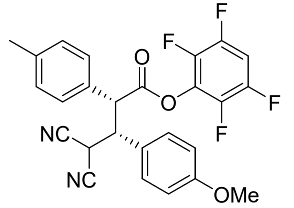

syn-**S27**

2D ^{1}H-^{13}C HMBC, $CDCl_3$

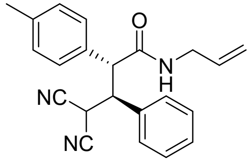

37

^{1}H, $CDCl_3$, 400 MHz

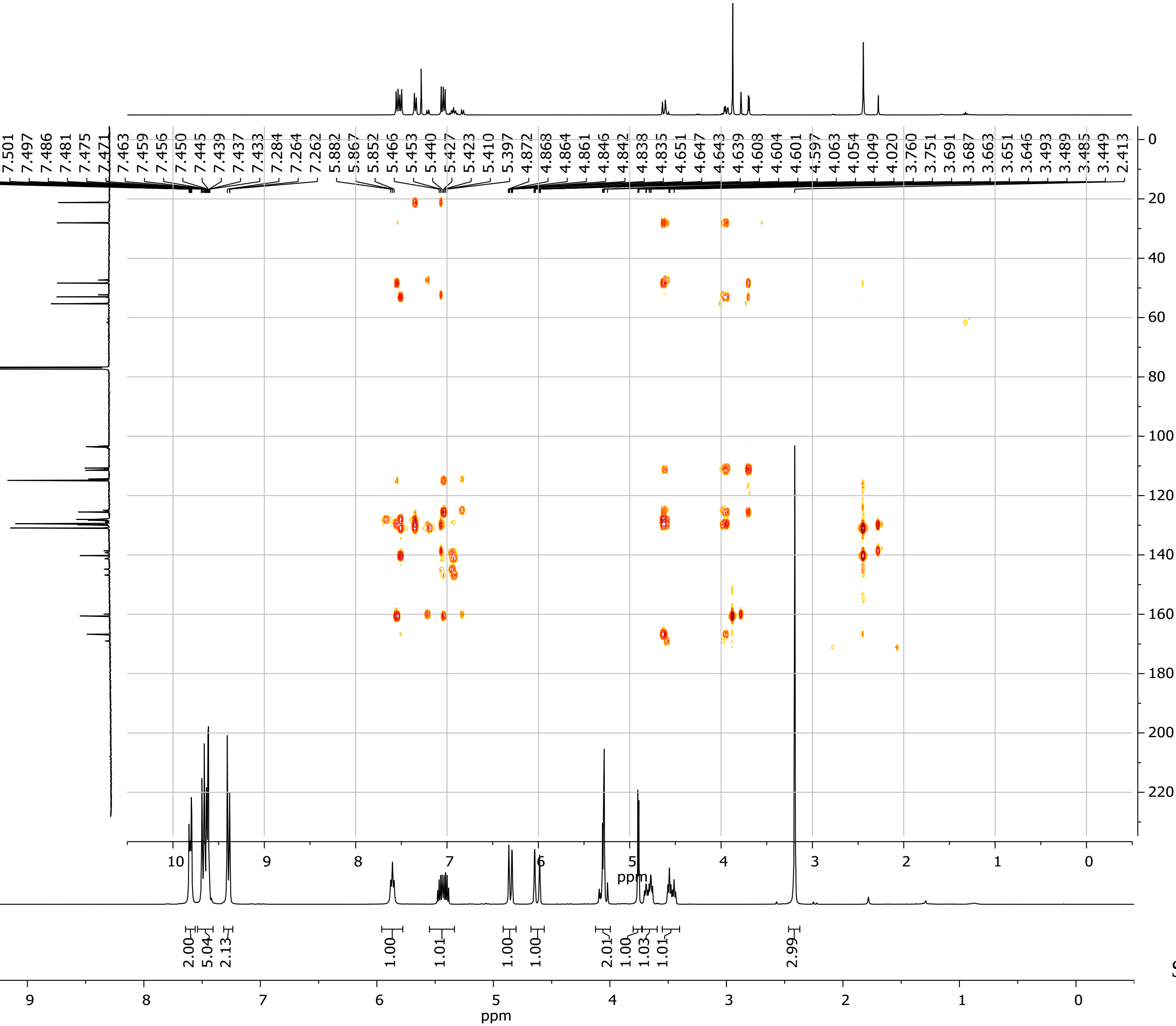

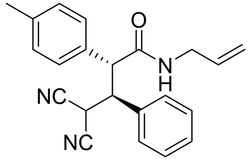

37

$^{13}C\{^{1}H\}$, $CDCl_3$, 126 MHz

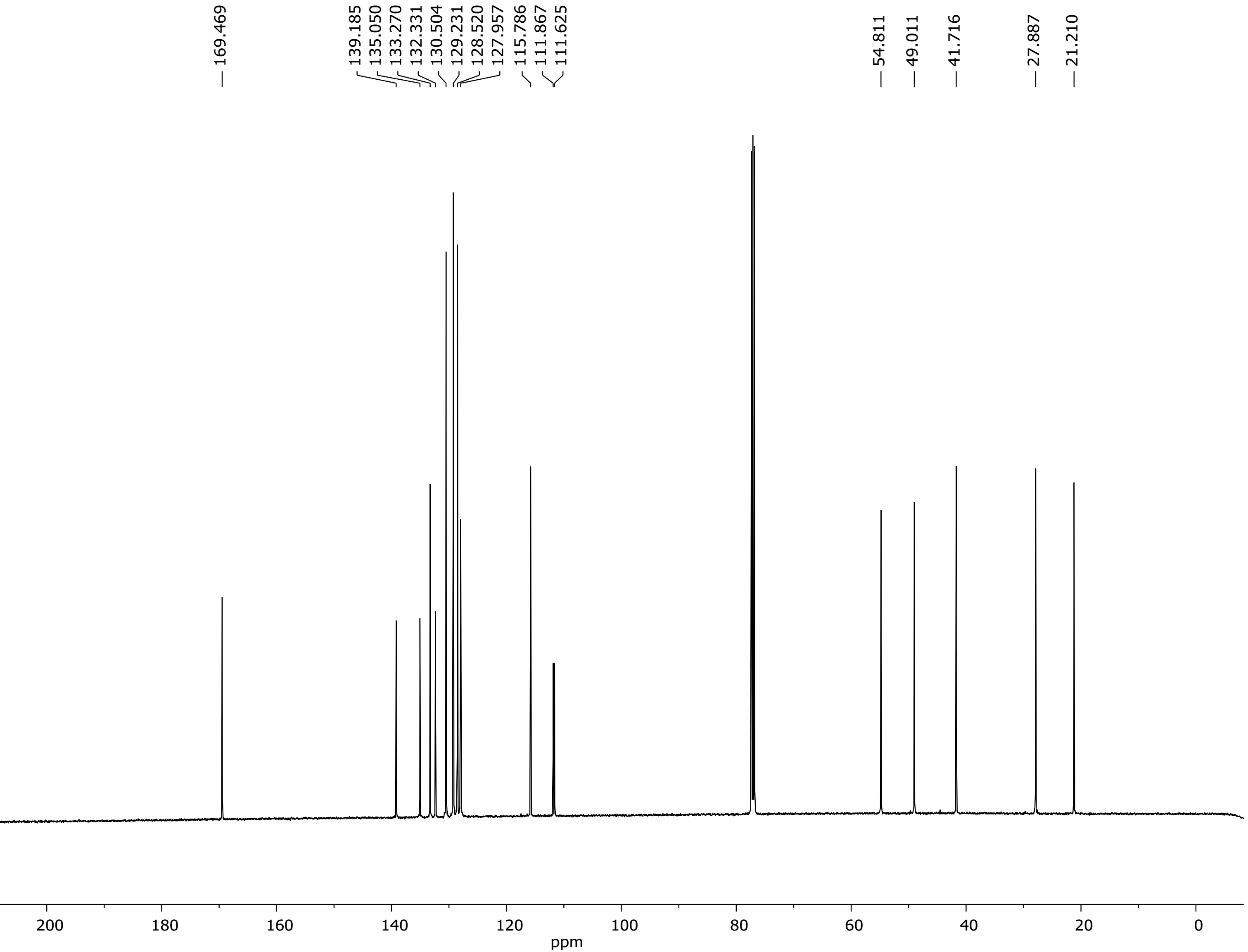

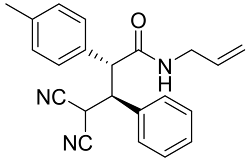

37

2D ^{1}H-^{1}H COSY, $CDCl_3$

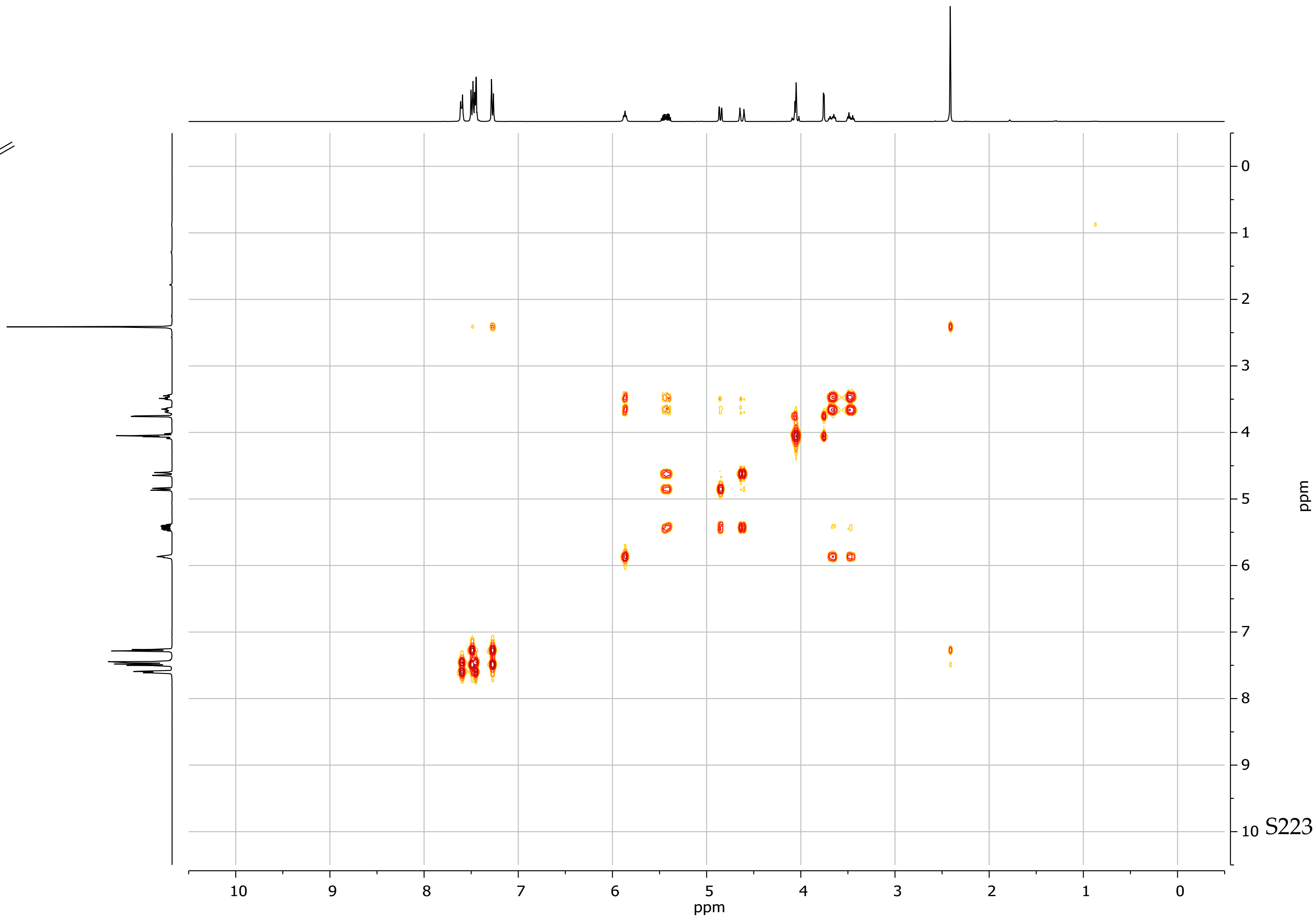

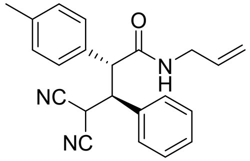

37

2D ^{1}H-^{13}C HSQC, $CDCl_3$

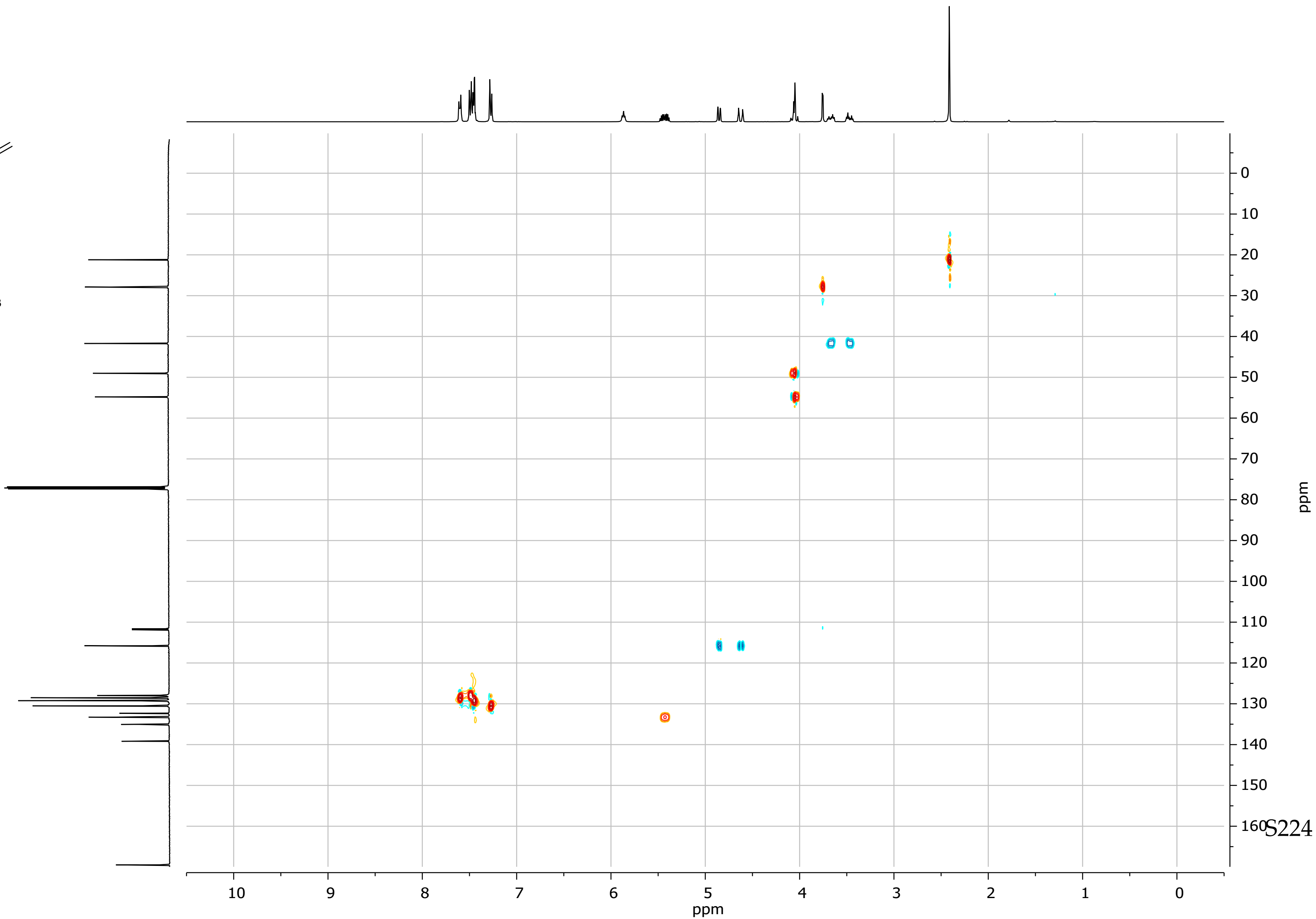

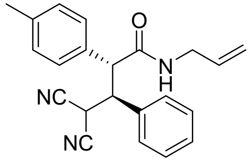

37
2D ^{1}H-^{13}C HMBC, $CDCl_3$

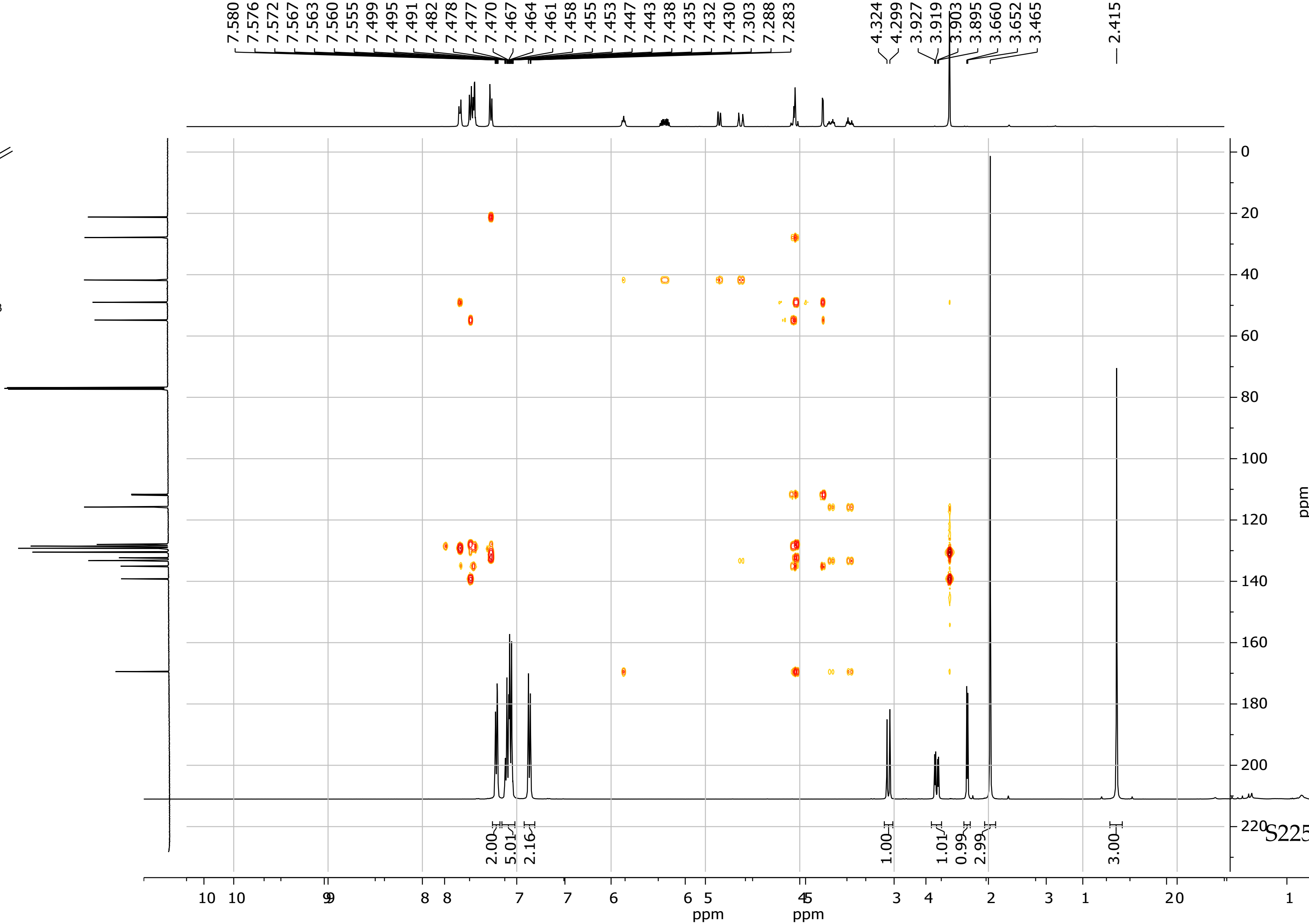

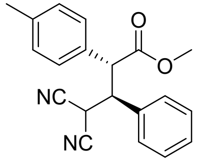

38
^{1}H, $CDCl_3$, 500 MHz

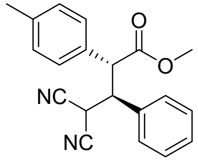

38

$^{13}C\{^1H\}$, $CDCl_3$, 126 MHz

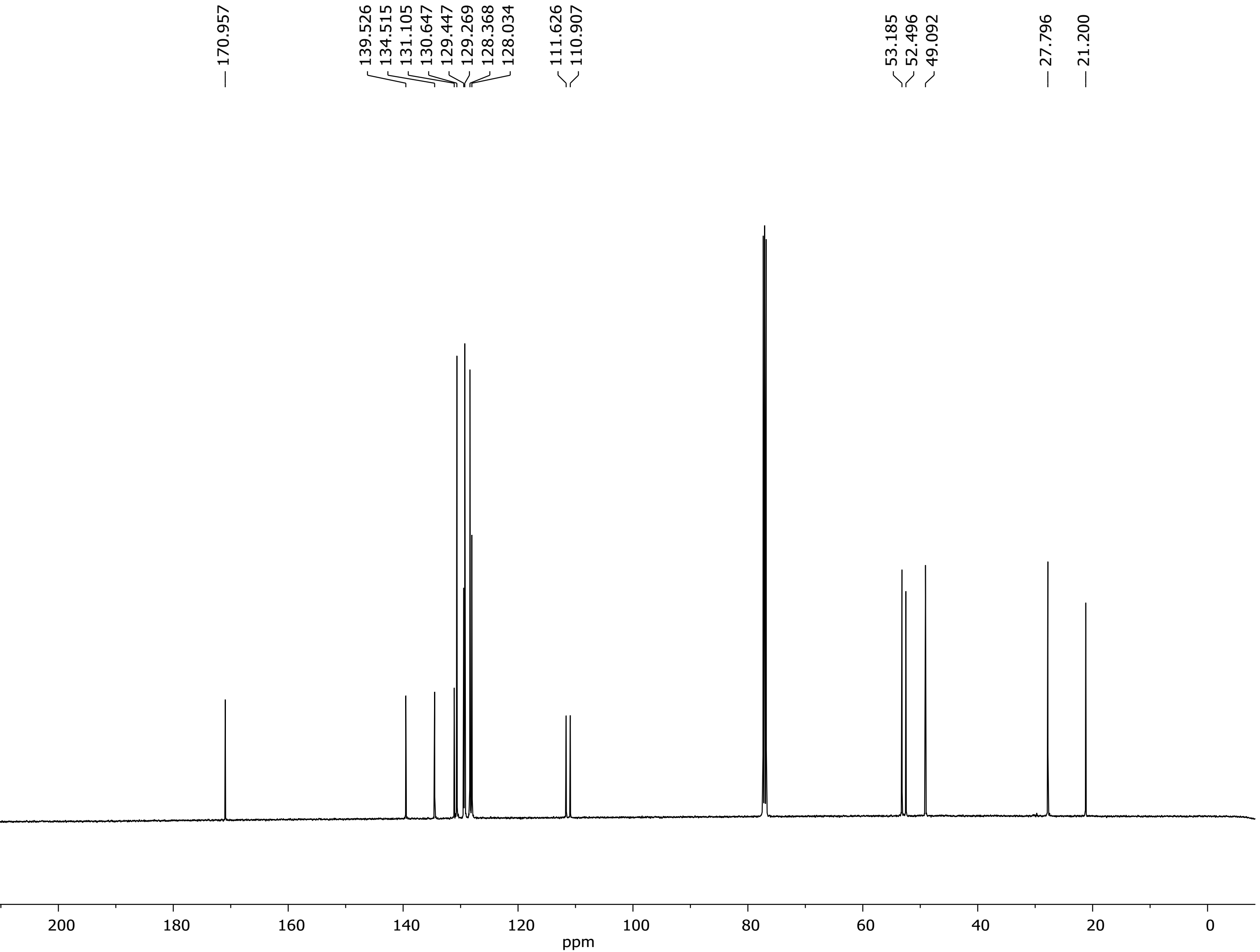

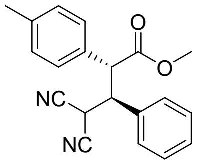

38
2D ^{1}H-^{13}C HSQC, $CDCl_3$

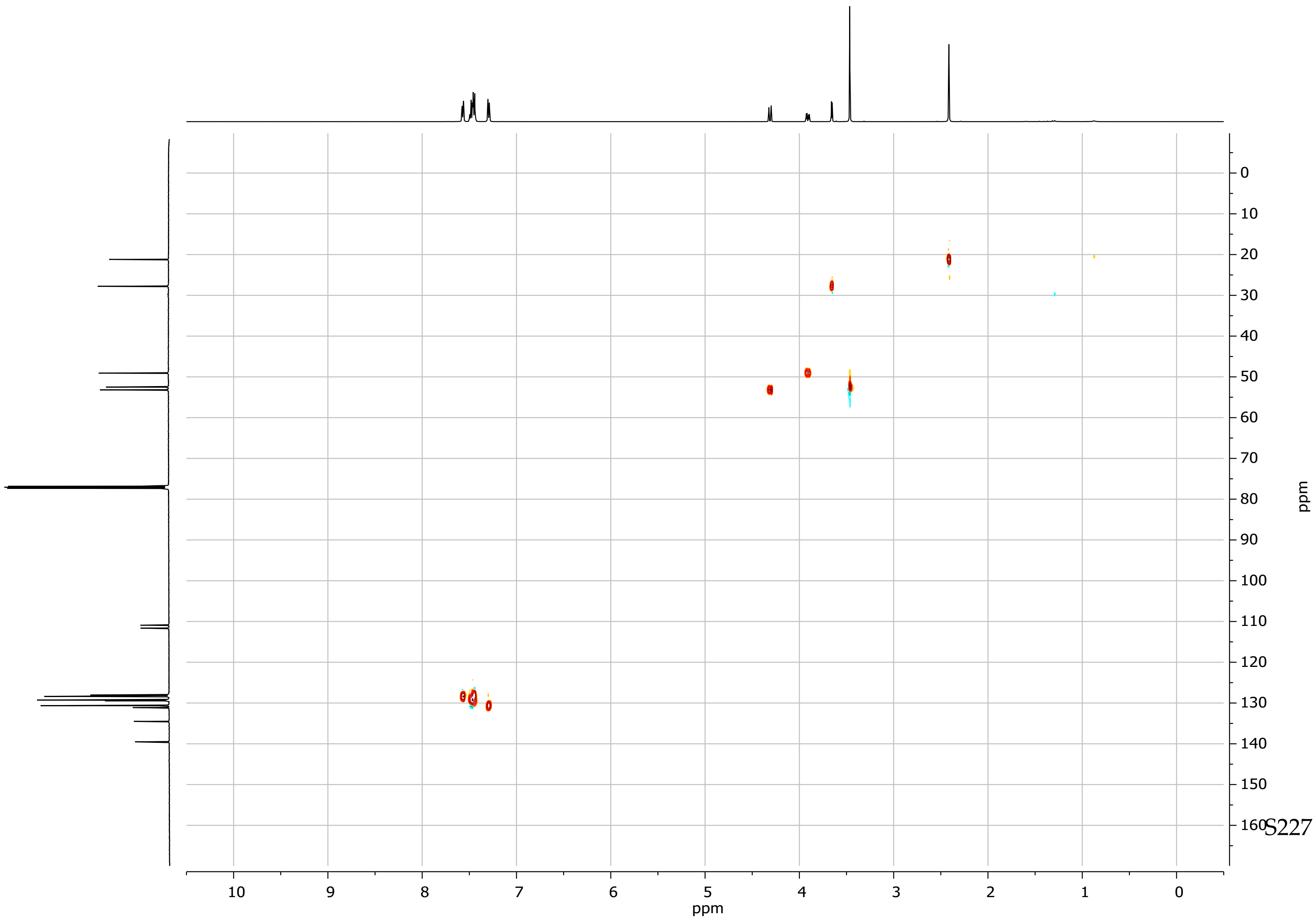

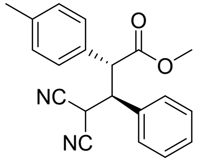

38

2D ^{1}H-^{13}C HMBC, $CDCl_3$

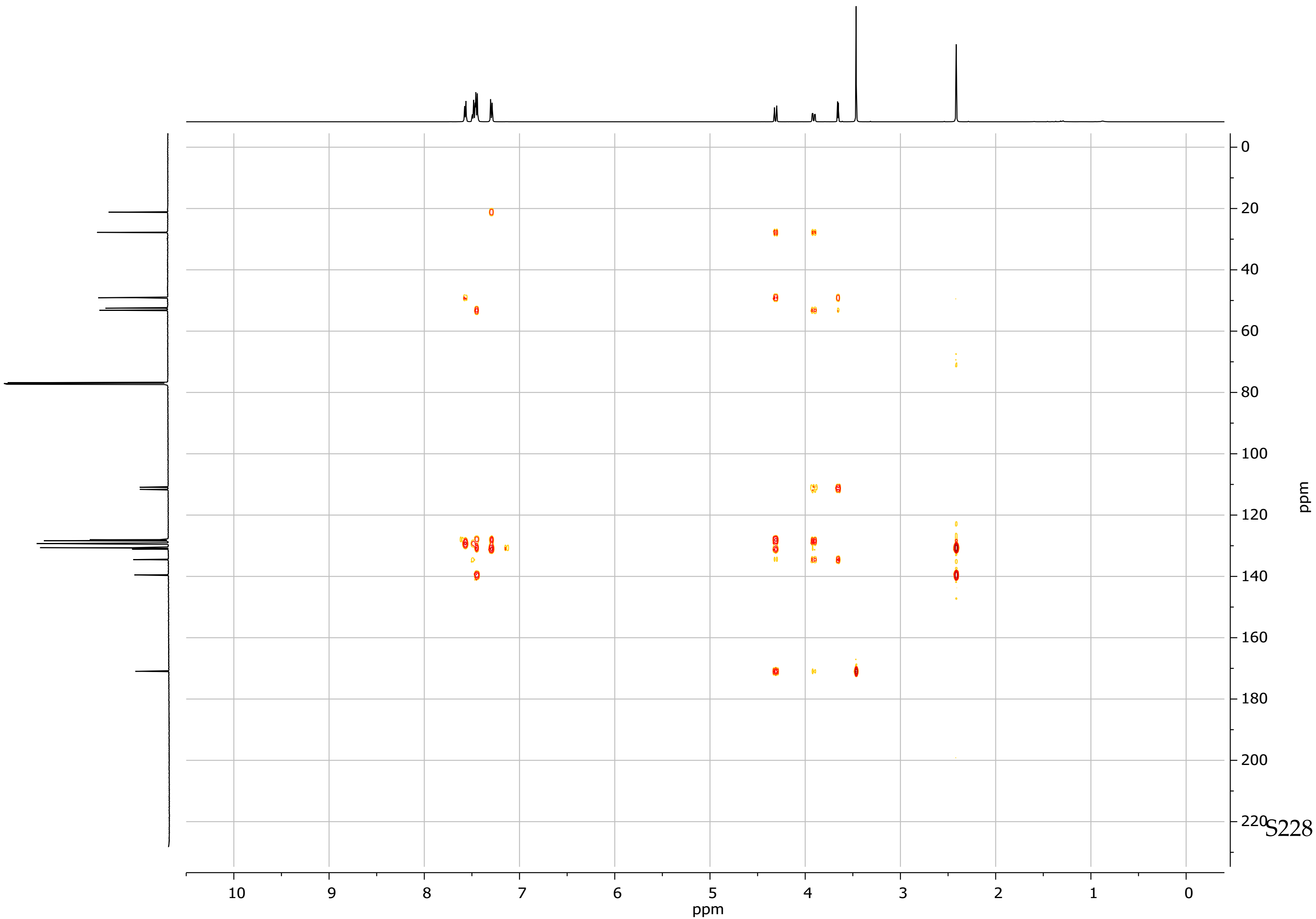

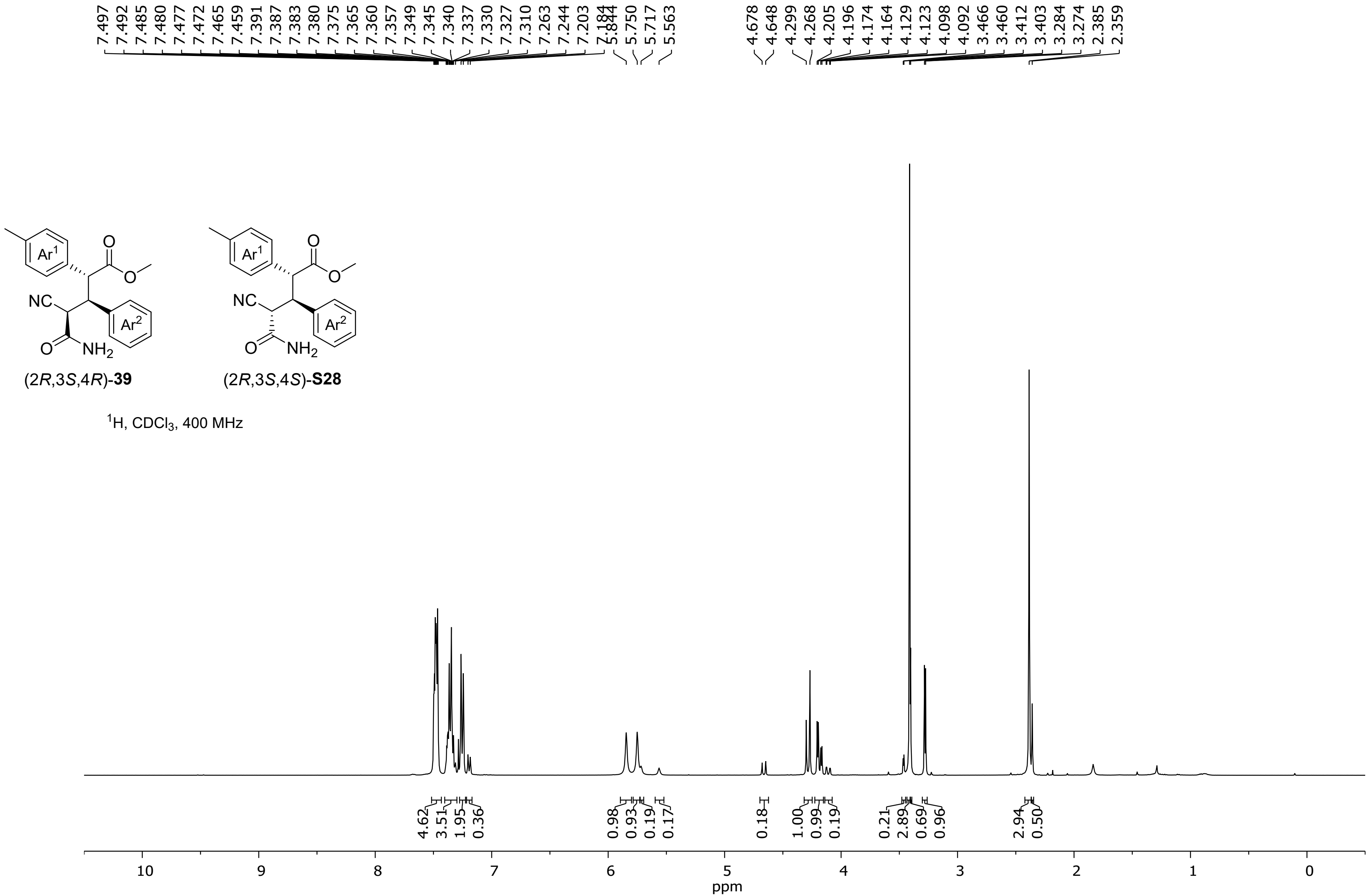
7.497 7.492 7.485 7.480 7.477 7.472 7.465 7.459 7.391 7.387 7.383 7.380 7.375 7.365 7.360 7.357 7.349 7.345 7.340 7.337 7.330 7.327 7.310 7.263 7.244 7.203 7.184 5.844 5.750 5.717 5.563 4.678 4.648 4.299 4.268 4.205 4.196 4.174 4.164 4.129 4.123 4.098 4.092 3.466 3.460 3.412 3.403 3.284 3.274 2.385 2.359
Ar1
Ar2
NC
O
NH2
(2R,3S,4R)-39
(2R,3S,4S)-S28
1H, CDCl3, 400 MHz
4.62 3.51 1.95 0.36 0.98 0.93 0.19 0.17 0.18 1.00 0.99 0.19 0.21 2.89 0.69 0.96 2.94 0.50
10 9 8 7 6 5 4 3 2 1 0
ppm

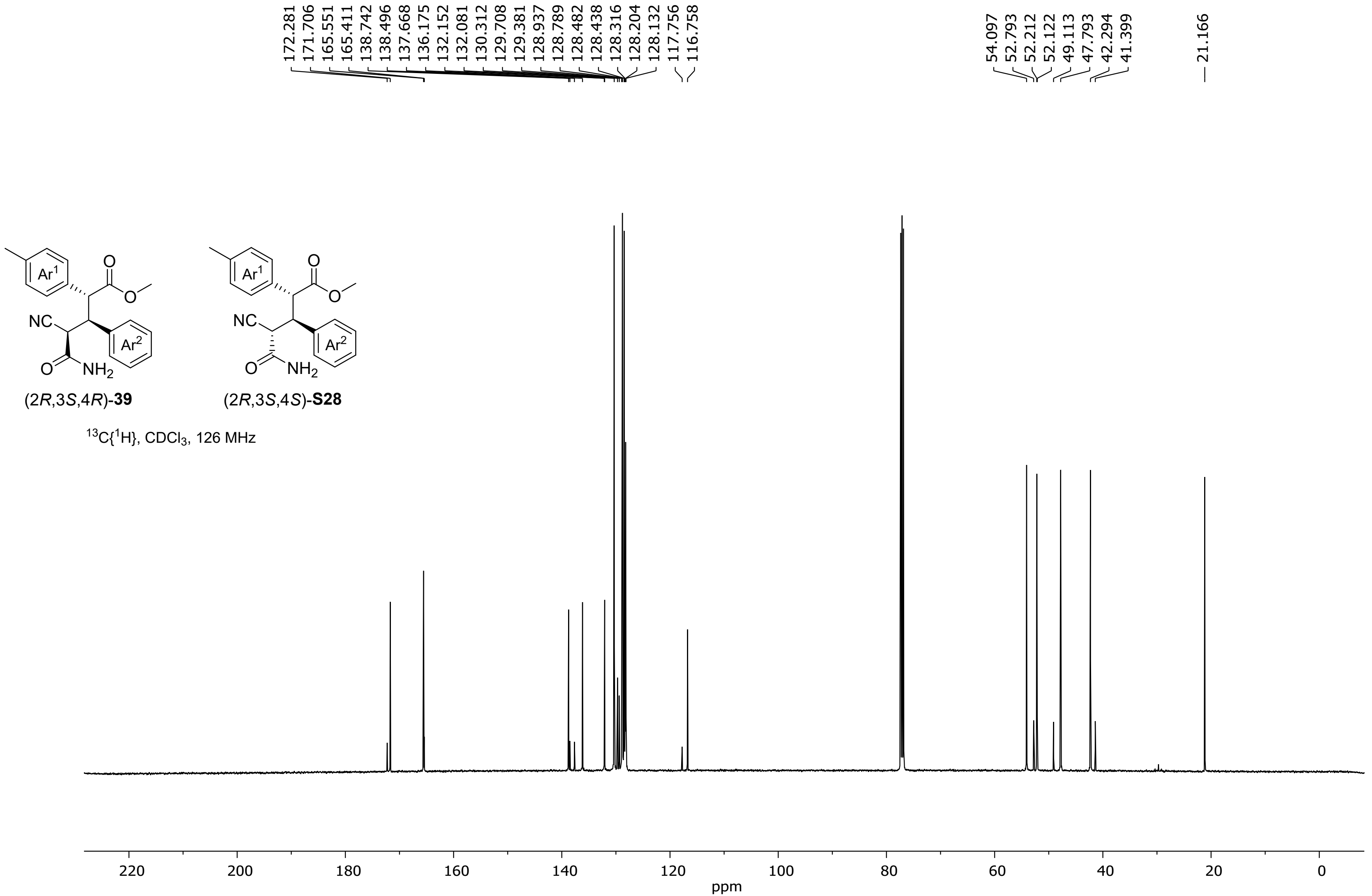
172.281
171.706
165.551
165.411
138.742
138.496
137.668
136.175
132.152
132.081
130.312
129.708
129.381
128.937
128.789
128.482
128.438
128.316
128.204
128.132
117.756
116.758
54.097
52.793
52.212
52.122
49.113
47.793
42.294
41.399
21.166
Ar1
Ar2
NC
NH2
(2R,3S,4R)-39
(2R,3S,4S)-S28
13C{1H}, CDCl3, 126 MHz
220
200
180
160
140
120
100
80
60
40
20
0
ppm

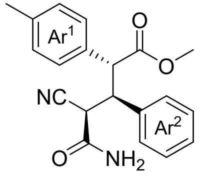

(2*R*,3*S*,4*R*)-**39**

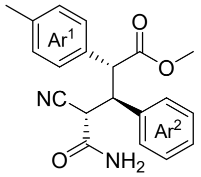

(2*R*,3*S*,4*S*)-**S28**

2D ^{1}H-^{1}H COSY, $CDCl_3$

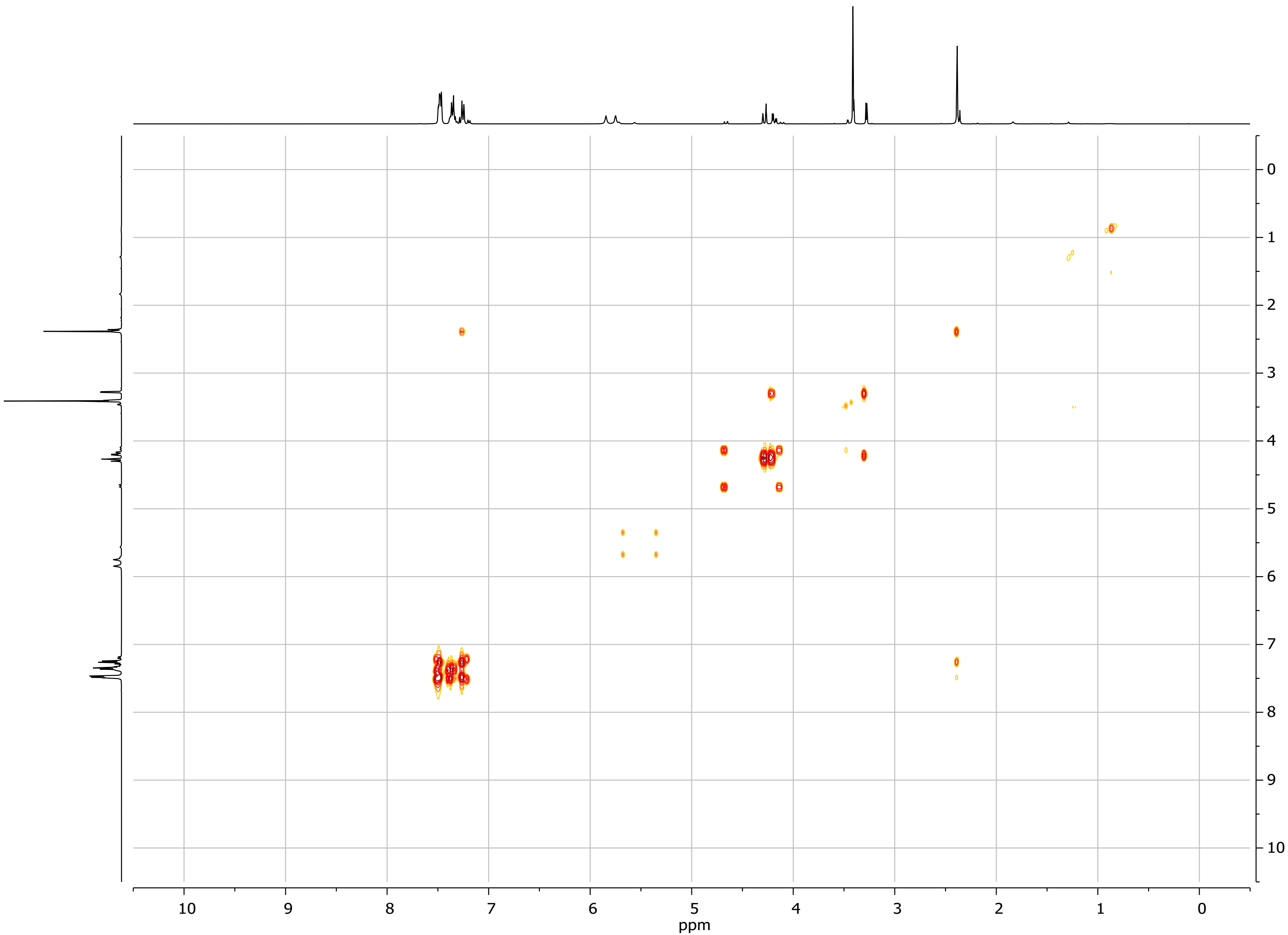

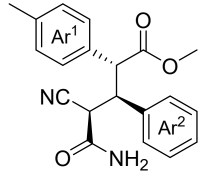

(2*R*,3*S*,4*R*)-**39**

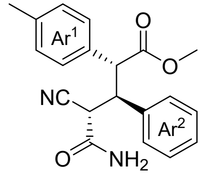

(2*R*,3*S*,4*S*)-**S28**

2D ^{1}H-^{13}C HSQC, $CDCl_3$

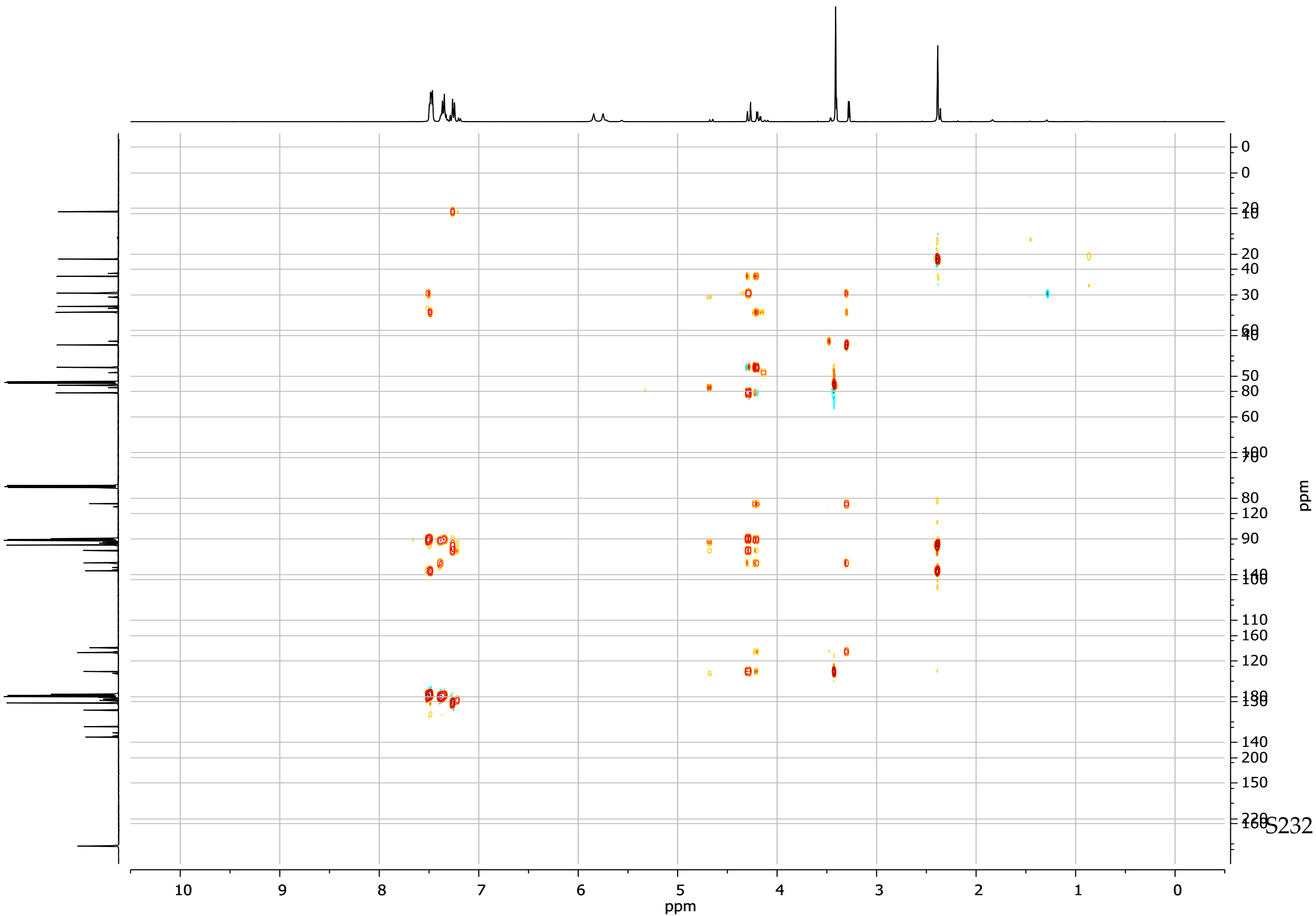

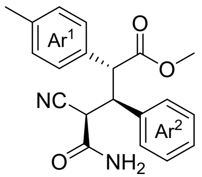

(2*R*,3*S*,4*R*)-**39**

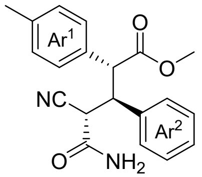

(2*R*,3*S*,4*S*)-**S28**

2D ^1H-^{13}C HMBC, $CDCl_3$

Appendix II: HPLC traces of novel compounds

HPLC data for *anti*-**14**: Chiralcel OD-H (99:1 *n*-hexane : IPA, flow rate 1.5 mLmin^{-1}, 211 nm, 30 °C) t_R (2*S*,3*R*) 24.4 min, t_R (2*R*,3*S*) 29.4 min, >99:1 er.

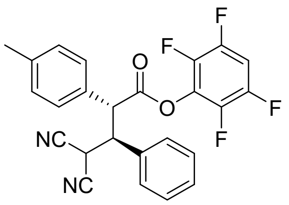

anti-**14**

PDA Ch1 211nm

Peak#	Ret. Time	Area%
1	23.999	50.221
2	33.034	49.779
Total		100.000

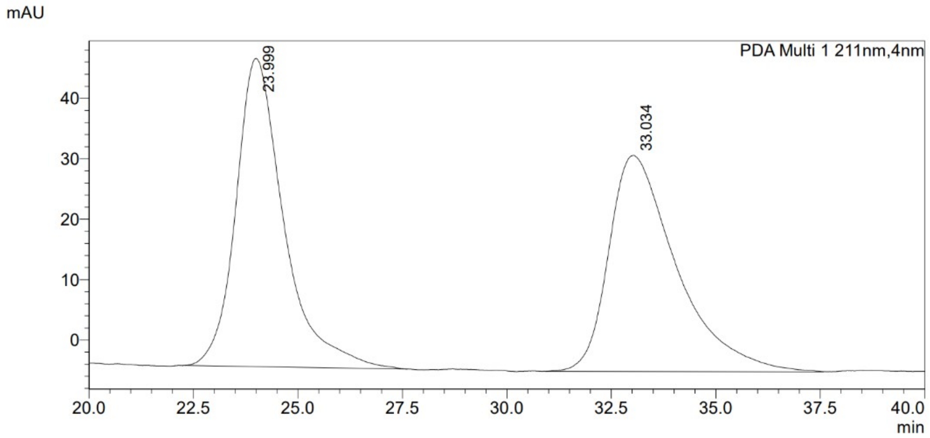

PDA Ch1 211nm

Peak#	Ret. Time	Area%
1	24.371	0.330
2	29.361	99.670
Total		100.000

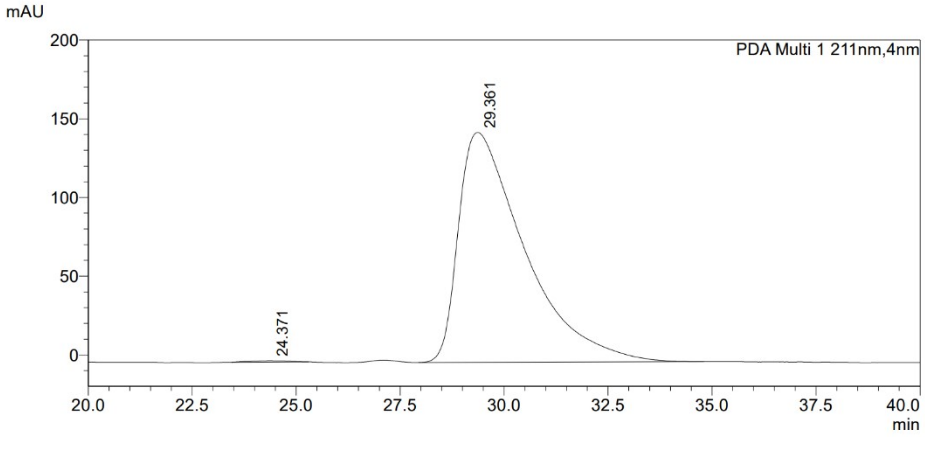

HPLC data for *anti*-**20**: Chiralcel OD-H (90:10 *n*-hexane : IPA, flow rate 1.0 mLmin^{-1}, 211 nm, 30 °C) t_R (2*S*,3*R*) 19.3 min, t_R (2*R*,3*S*) 22.7 min, 99:1 er.

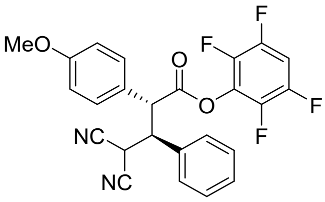

anti-**20**

PDA Ch1 211nm

Peak#	Ret. Time	Area%
1	19.133	50.248
2	22.995	49.752
Total		100.000

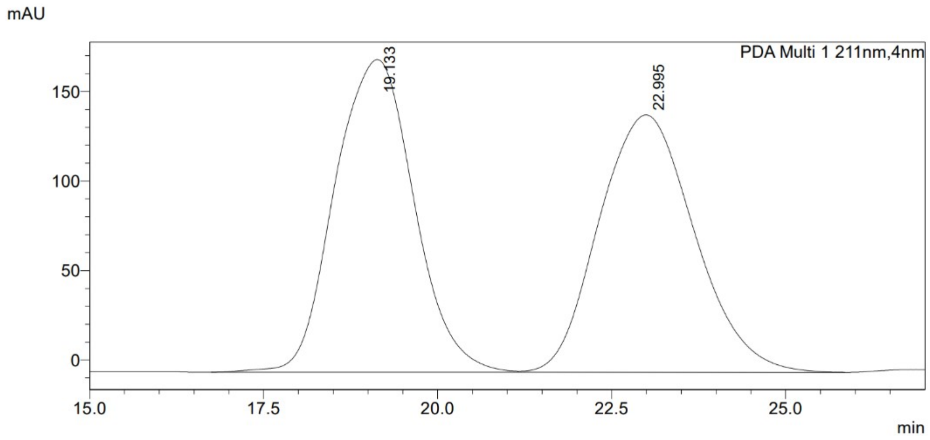

PDA Ch1 211nm

Peak#	Ret. Time	Area%
1	19.319	0.767
2	22.714	99.233
Total		100.000

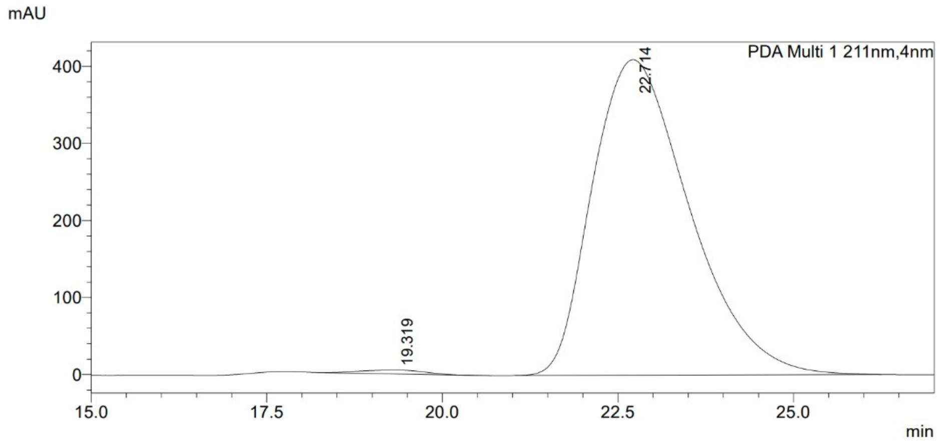

HPLC data for *syn*-**S17**: Chiralcel OD-H (90:10 *n*-hexane : IPA, flow rate 1.0 mLmin^{-1}, 211 nm, 30 °C) t_R (2*S*,3*S*) 11.7 min, t_R (2*R*,3*R*) 16.1 min, 96:4 er.

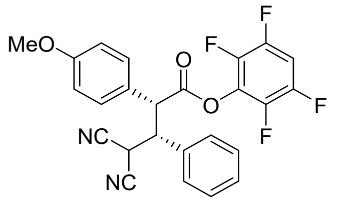

syn-**S17**

PDA Ch1 211nm

Peak#	Ret. Time	Area%
1	11.566	49.852
2	16.213	50.148
Total		100.000

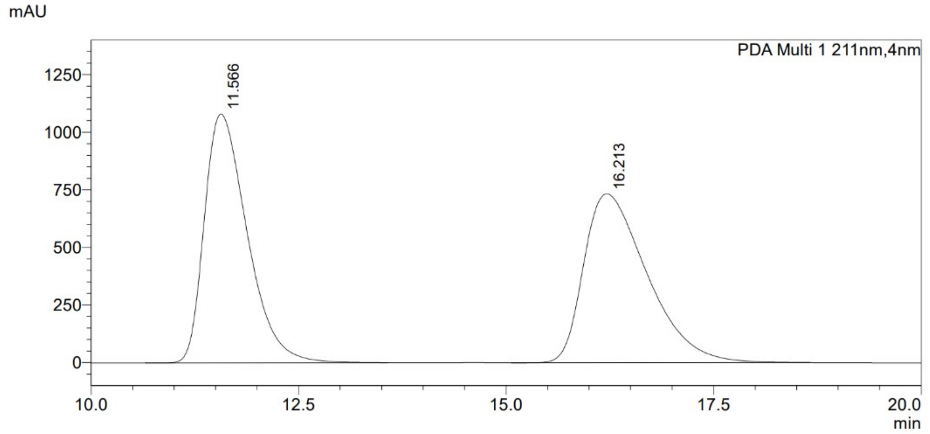

PDA Ch1 211nm

Peak#	Ret. Time	Area%
1	11.673	3.847
2	16.092	96.153
Total		100.000

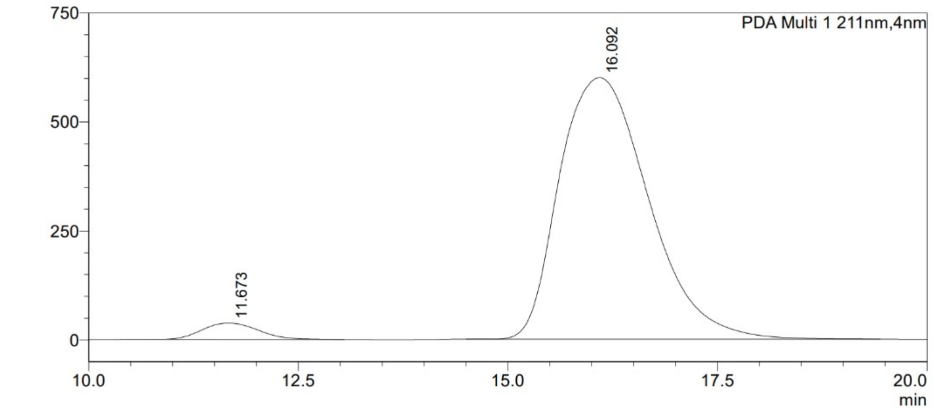

HPLC data for *anti*-**21**: Chiralcel AD-H (90:10 *n*-hexane : IPA, flow rate 1.0 mLmin^{-1}, 211 nm, 30 °C) t_R (2*R*,3*S*) 11.3 min, t_R (2*S*,3*R*) 14.7 min, 99:1 er.

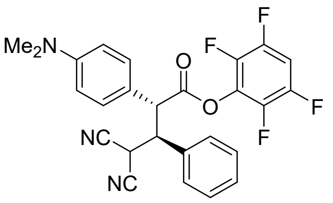

anti-**21**

PDA Ch1 211nm

Peak#	Ret. Time	Area%
1	11.092	49.724
2	14.715	50.276
Total		100.000

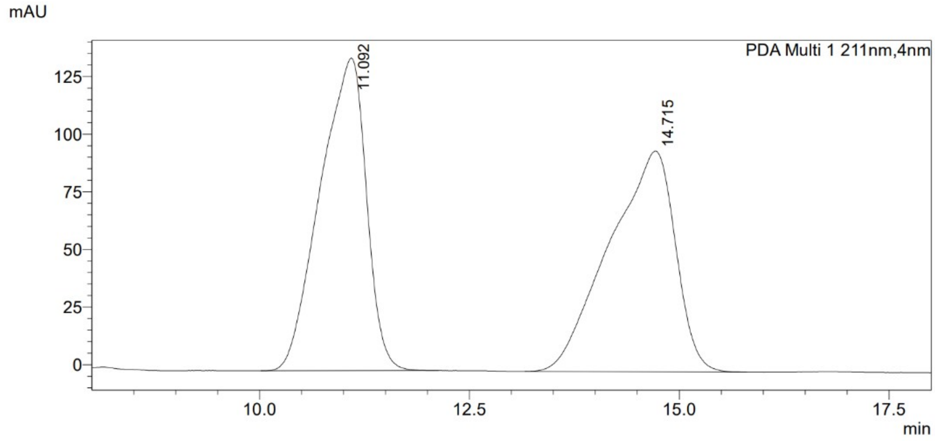

PDA Ch1 211nm

Peak#	Ret. Time	Area%
1	11.260	99.284
2	14.710	0.716
Total		100.000

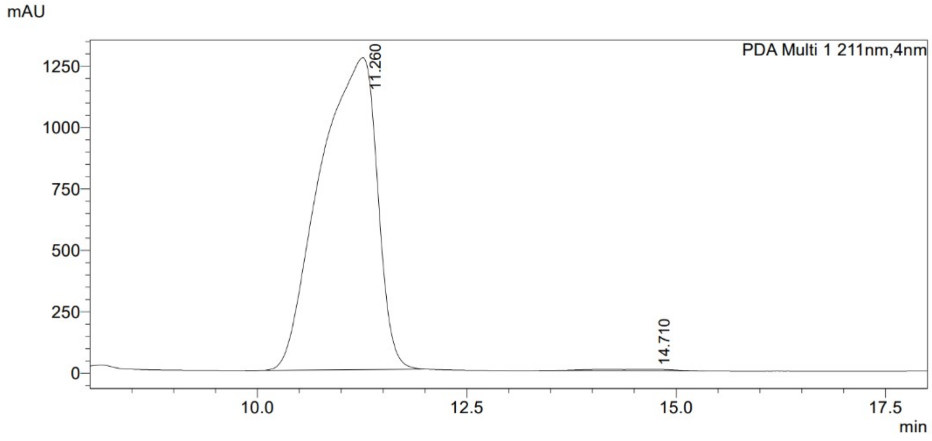

HPLC data for *anti*-**22**: Chiralpak IA (97:3 *n*-hexane : IPA, flow rate 1.0 mLmin^{-1}, 211 nm, 30 °C) t_R (2*R*,3*S*) 10.1 min, t_R (2*S*,3*R*) 14.6 min, 99:1 er.

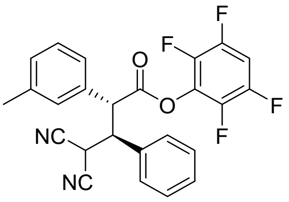

anti-**22**

PDA Ch1 211nm

Peak#	Ret. Time	Area%
1	10.124	50.049
2	14.733	49.951
Total		100.000

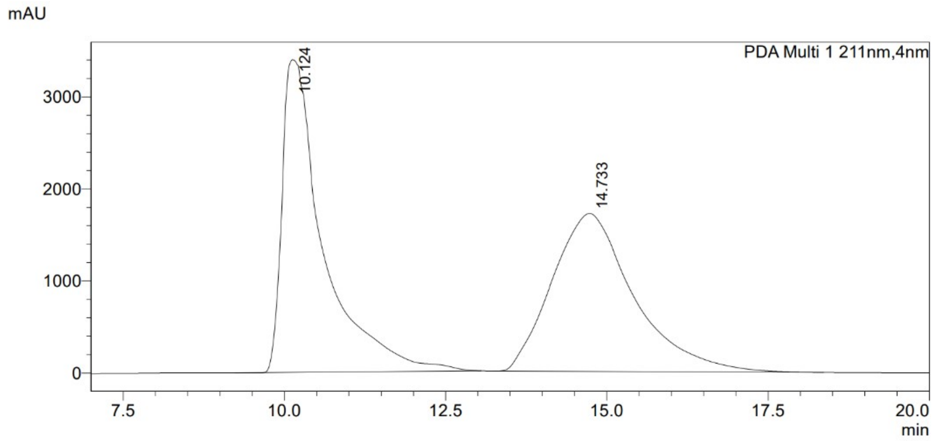

PDA Ch1 211nm

Peak#	Ret. Time	Area%
1	10.124	99.401
2	14.613	0.599
Total		100.000

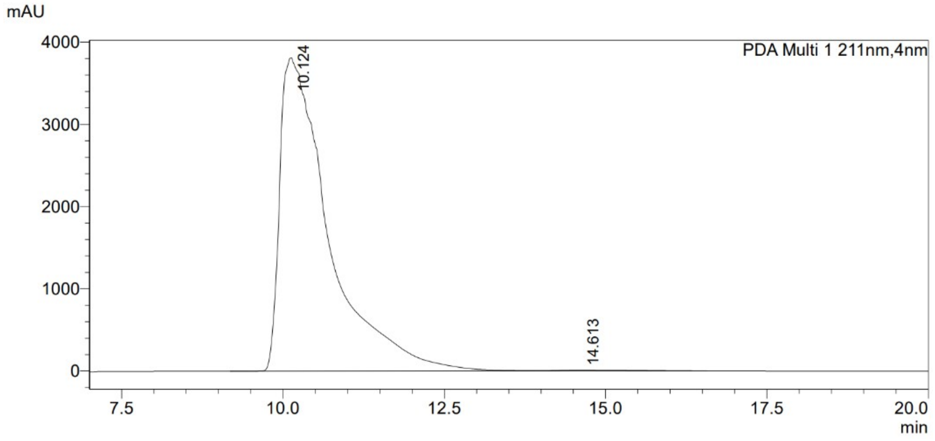

HPLC data for *syn*-**S18**: Chiralpak AD-H (97:3 *n*-hexane : IPA, flow rate 1.0 mLmin^{-1}, 211 nm, 30 °C) t_R (2*R*,3*R*) 10.8 min, t_R (2*S*,3*S*) 15.8 min, 91:9 er.

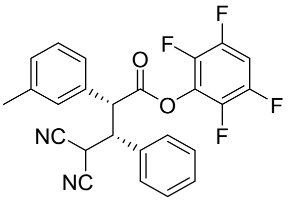

syn-**S18**

PDA Ch1 211nm

Peak#	Ret. Time	Area%
1	10.729	49.730
2	15.694	50.270
Total		100.000

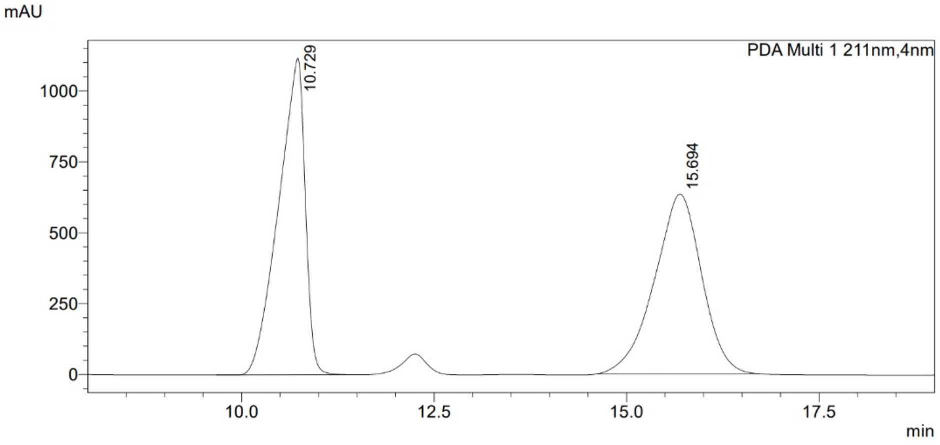

PDA Ch1 211nm

Peak#	Ret. Time	Area%
1	10.791	90.714
2	15.808	9.286
Total		100.000

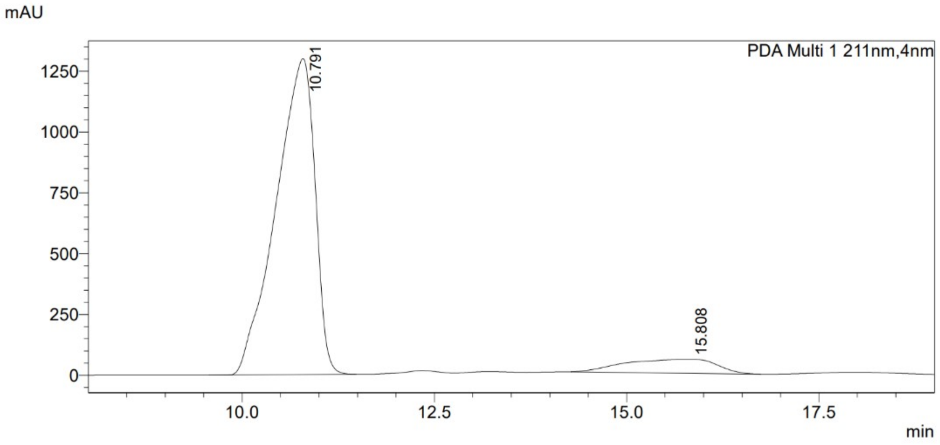

HPLC data for *anti*-**23**: Chiralcel OD-H (99.5:0.5 *n*-hexane : IPA, flow rate 1.0 mLmin^{-1}, 211 nm, 30 °C) t_R (2*S*,3*R*) 40.6 min, t_R (2*R*,3*S*) 47.8 min, 91:9 er.

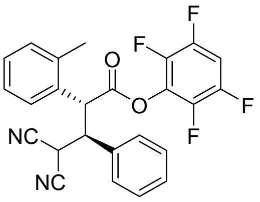

anti-**23**

PDA Ch1 211nm

Peak#	Ret. Time	Area%
1	40.364	49.702
2	49.144	50.298
Total		100.000

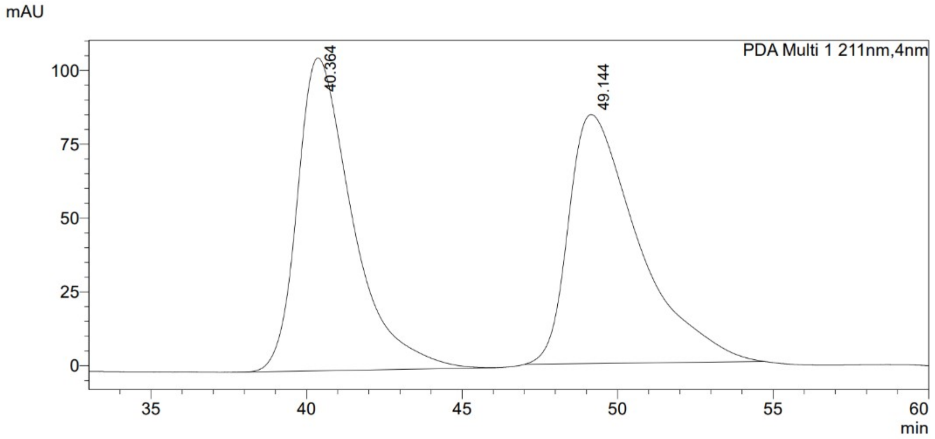

PDA Ch1 211nm

Peak#	Ret. Time	Area%
1	40.557	9.201
2	47.579	90.799
Total		100.000

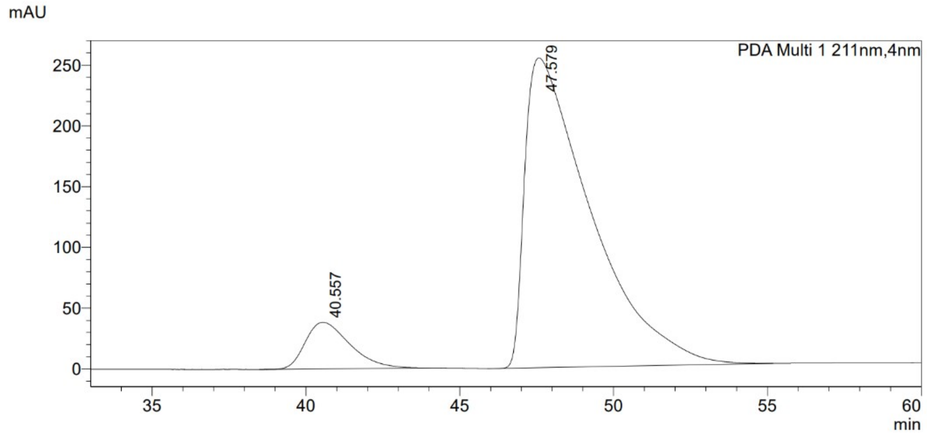

HPLC data for *syn*-**S19**: Chiralcel OD-H (93:7 *n*-hexane : IPA, flow rate 1.5 mLmin^{-1}, 211 nm, 30 °C) t_R (2*S*,3*S*) 7.3 min, t_R (2*R*,3*R*) 11.4 min, 83:17 er.

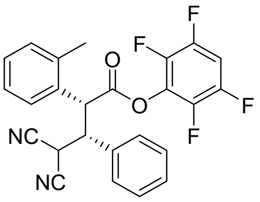

syn-**S19**

PDA Ch1 211nm

Peak#	Ret. Time	Area%
1	7.267	49.797
2	11.453	50.203
Total		100.000

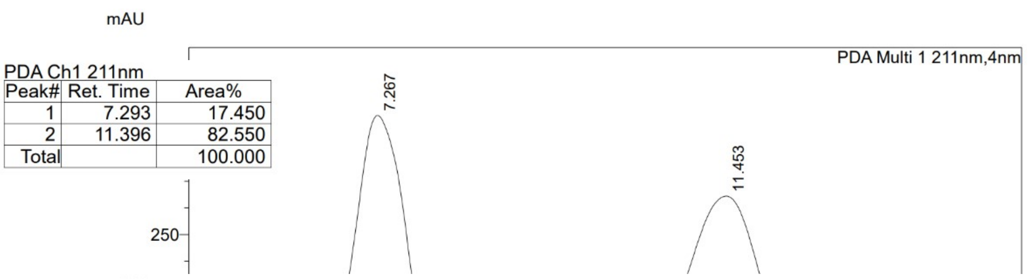

PDA Ch1 211nm

Peak#	Ret. Time	Area%
1	7.293	17.450
2	11.396	82.550
Total		100.000

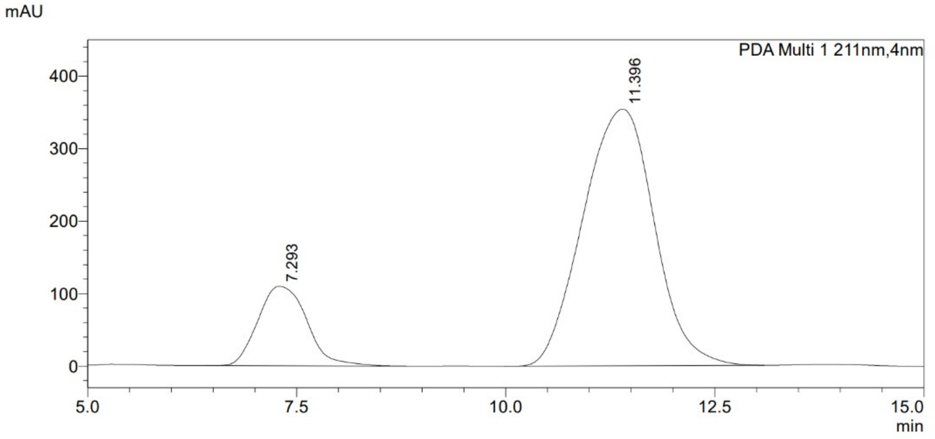

HPLC data for *anti*-**24**: Chiralcel OD-H (97:3 *n*-hexane : IPA, flow rate 1.0 mLmin^{-1}, 211 nm, 30 °C) t_R (2*R*,3*S*) 27.6 min, t_R (2*S*,3*R*) 31.2 min, 97:3 er.

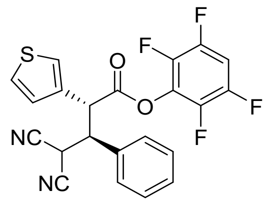

anti-**24**

PDA Ch1 211nm

Peak#	Ret. Time	Area%
1	28.309	49.908
2	31.401	50.092
Total		100.000

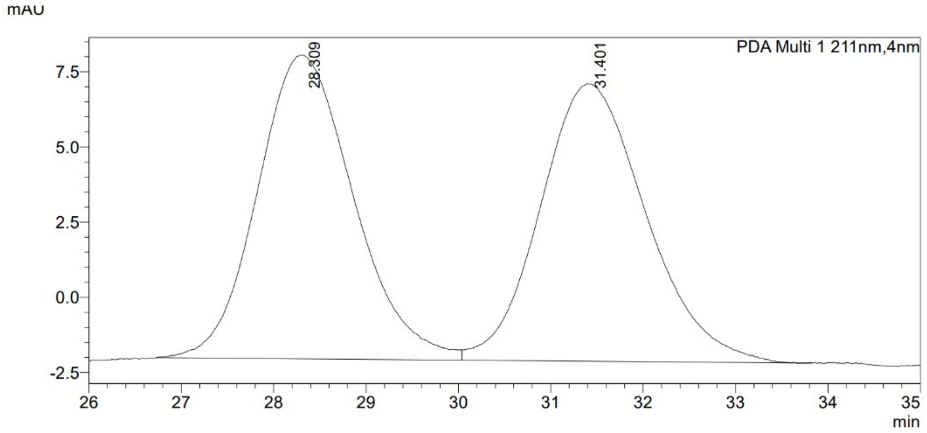

PDA Ch1 211nm

Peak#	Ret. Time	Area%
1	27.557	97.161
2	31.243	2.839
Total		100.000

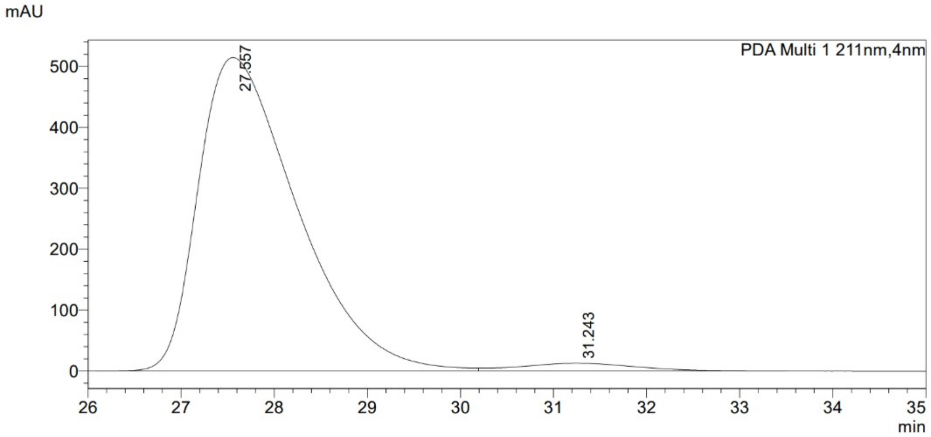

HPLC data for *syn*-**S20**: Chiralpak IB (98:2 *n*-hexane : IPA, flow rate 1.0 mLmin^{-1}, 211 nm, 30 °C) t_R (2*S*,3*S*) 22.6 min, t_R (2*R*,3*R*) 29.1 min, 87:13 er.

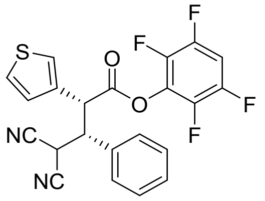

syn-**S20**

PDA Ch1 211nm

Peak#	Ret. Time	Area%
1	22.483	49.935
2	28.828	50.065
Total		100.000

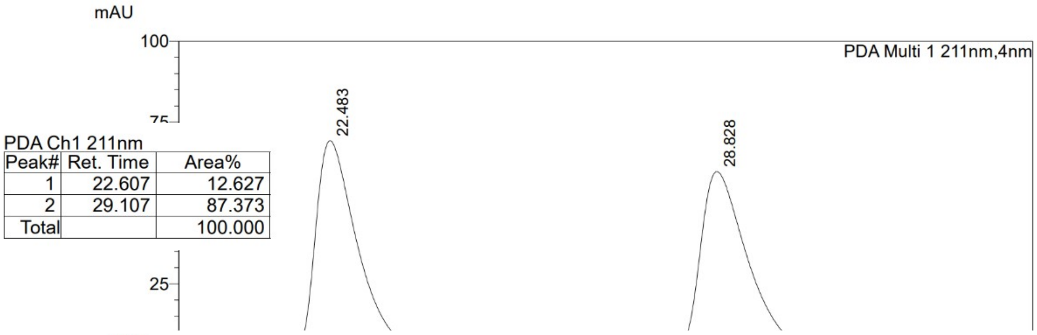

PDA Ch1 211nm

Peak#	Ret. Time	Area%
1	22.607	12.627
2	29.107	87.373
Total		100.000

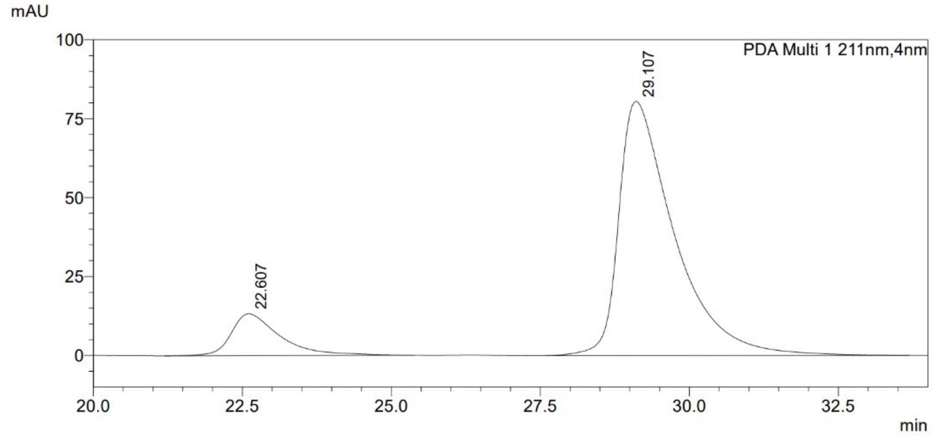

HPLC data for *anti*-**25**: Chiralcel OD-H (95:5 *n*-hexane : IPA, flow rate 1.0 mLmin^{-1}, 211 nm, 30 °C) t_R (2*S*,3*S*) 12.3 min, t_R (2*R*,3*R*) 19.9 min, 99:1 er.

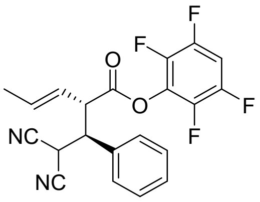

anti-**25**

PDA Ch1 211nm

Peak#	Ret. Time	Area%
1	12.074	49.991
2	19.178	50.009
Total		100.000

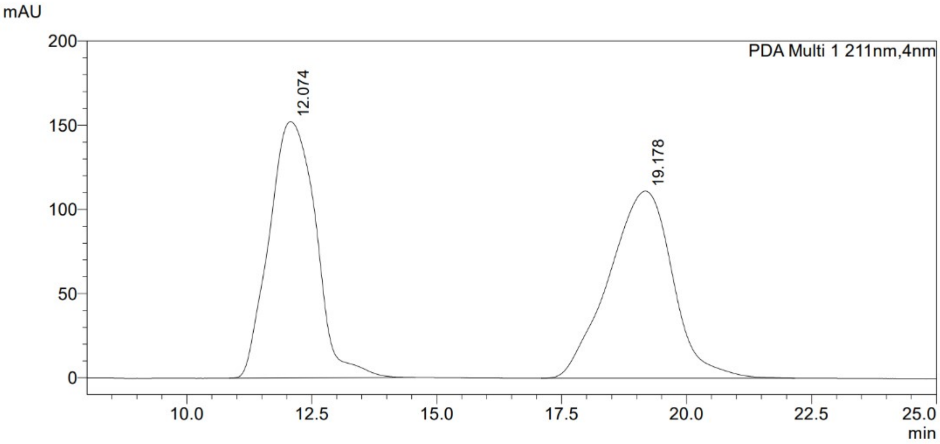

PDA Ch1 211nm

Peak#	Ret. Time	Area%
1	12.300	98.807
2	19.853	1.193
Total		100.000

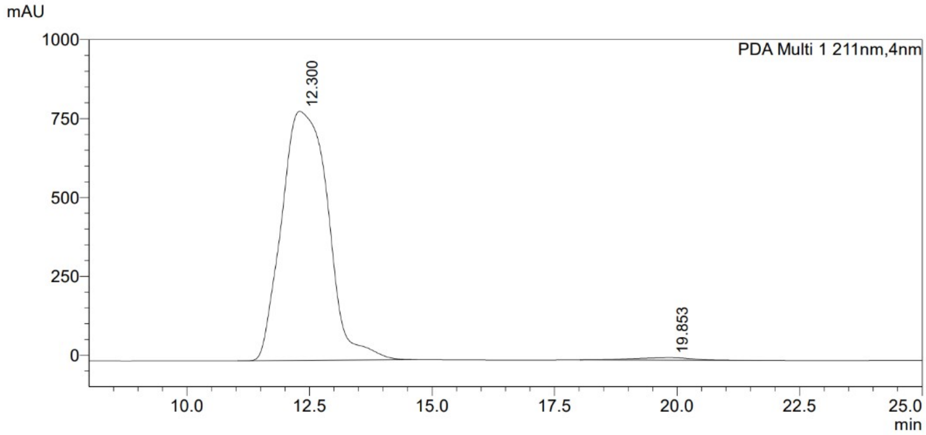

HPLC data for *syn*-**S21**: Chiralcel OD-H (99:1 *n*-hexane : IPA, flow rate 1.5 mLmin^{-1}, 211 nm, 40 °C) t_R (2*R*,3*S*) 16.4 min, t_R (2*S*,3*R*) 23.3 min, 97:3 er.

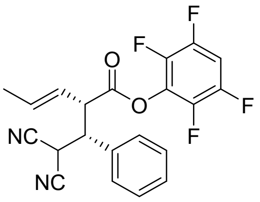

syn-**S21**

PDA Ch1 211nm

Peak#	Ret. Time	Area%
1	16.517	50.262
2	23.940	49.738
Total		100.000

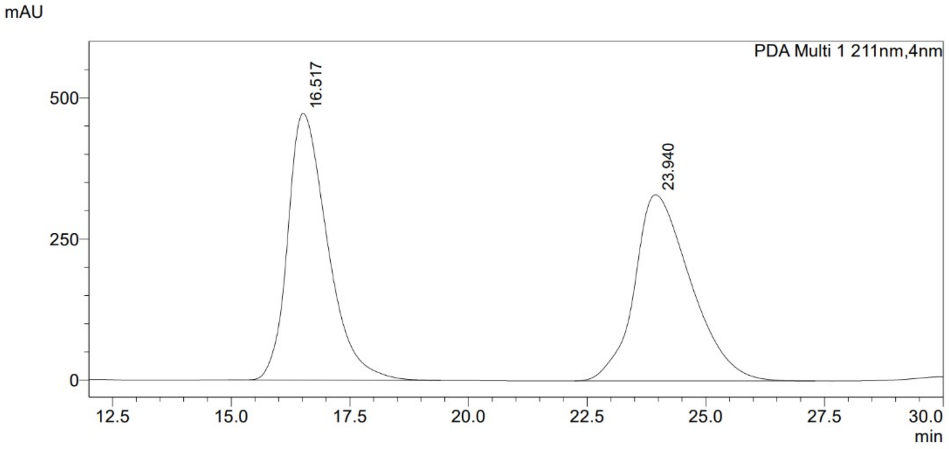

PDA Ch1 211nm

Peak#	Ret. Time	Area%
1	16.359	3.170
2	23.281	96.830
Total		100.000

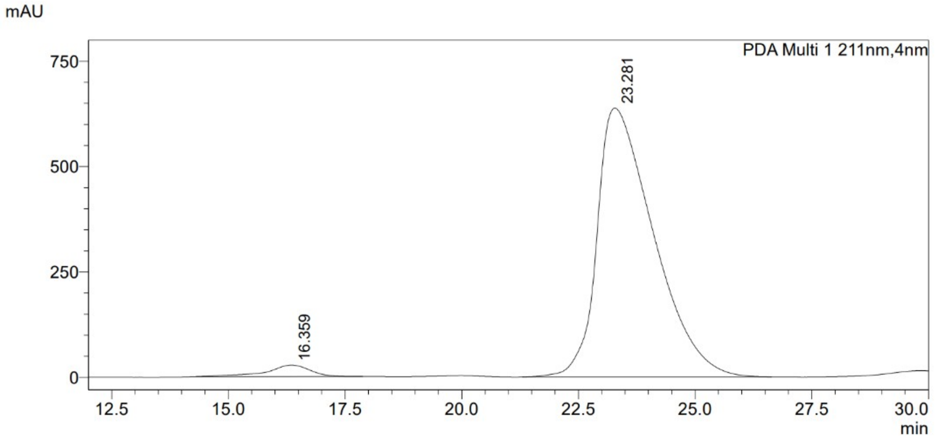

HPLC data for *anti*-**26**: Chiralcel OD-H (93:7 *n*-hexane : IPA, flow rate 2.0 mLmin^{-1}, 211 nm, 30 °C) t_R (2*S*,3*R*) 7.0 min, t_R (2*R*,3*S*) 12.6 min, 98:2 er.

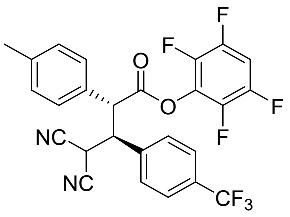

anti-**26**

PDA Ch1 211nm

Peak#	Ret. Time	Area%
1	6.907	50.194
2	12.564	49.806
Total		100.000

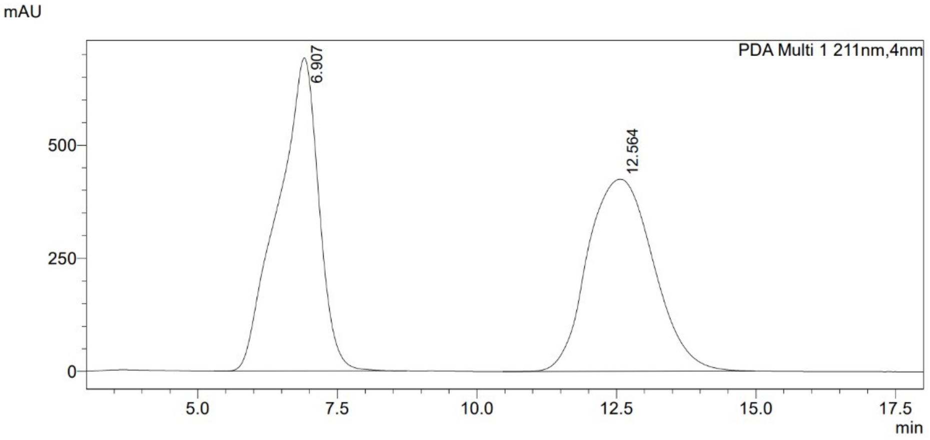

PDA Ch1 211nm

Peak#	Ret. Time	Area%
1	6.990	2.312
2	12.621	97.688
Total		100.000

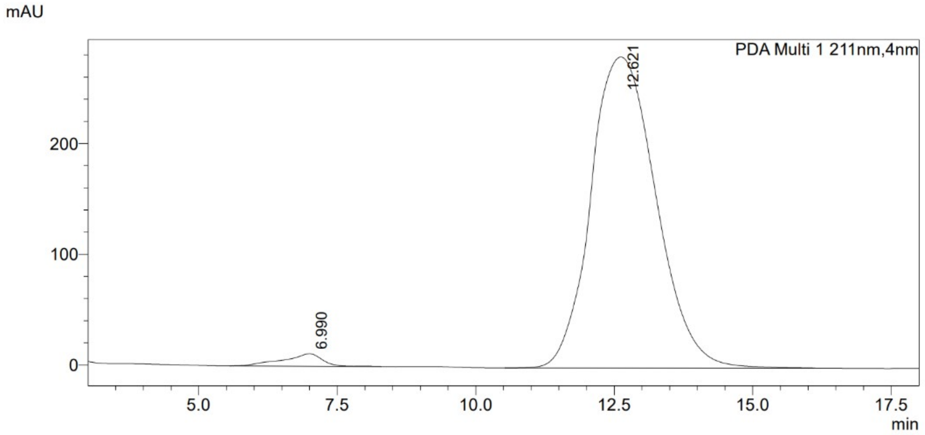

HPLC data for *syn*-**S22**: Chiralcel OD-H (95:5 *n*-hexane : IPA, flow rate 1.0 mLmin^{-1}, 211 nm, 30 °C) t_R (2*S*,3*S*) 10.7 min, t_R (2*R*,3*R*) 24.6 min, 87:13 er.

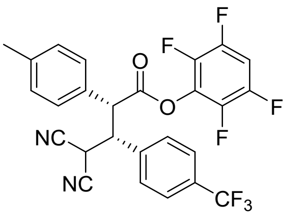

syn-**S22**

PDA Ch1 211nm

Peak#	Ret. Time	Area%
1	10.514	49.885
2	23.920	50.115
Total		100.000

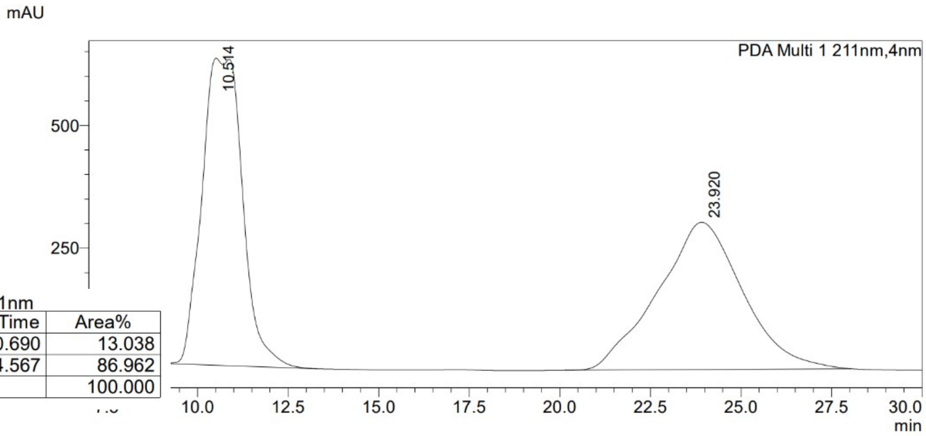

PDA Ch1 211nm

Peak#	Ret. Time	Area%
1	10.690	13.038
2	24.567	86.962
Total		100.000

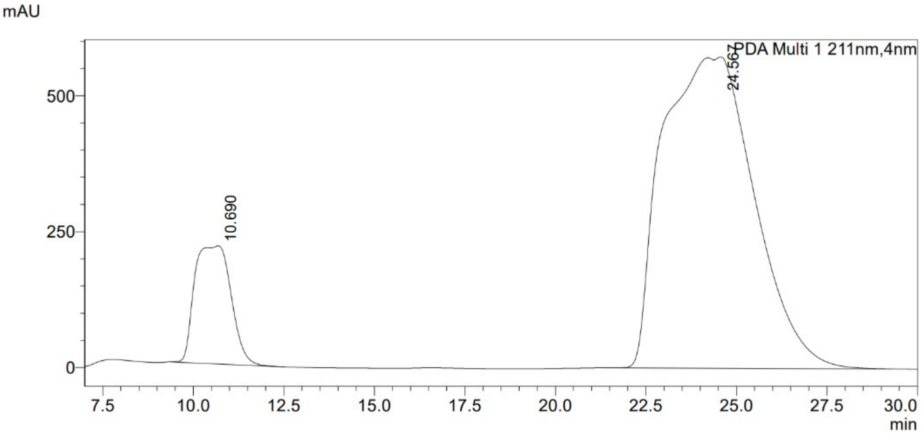

HPLC data for *anti*-**27**: Chiralcel OD-H (93:7 *n*-hexane : IPA, flow rate 1.0 mLmin^{-1}, 211 nm, 40 °C) t_R (2*S*,3*R*) 25.3 min, t_R (2*R*,3*S*) 46.2 min, 99:1 er.

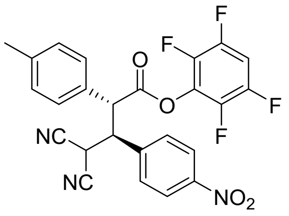

anti-**27**

PDA Ch1 211nm

Peak#	Ret. Time	Area%
1	25.230	50.037
2	46.448	49.963
Total		100.000

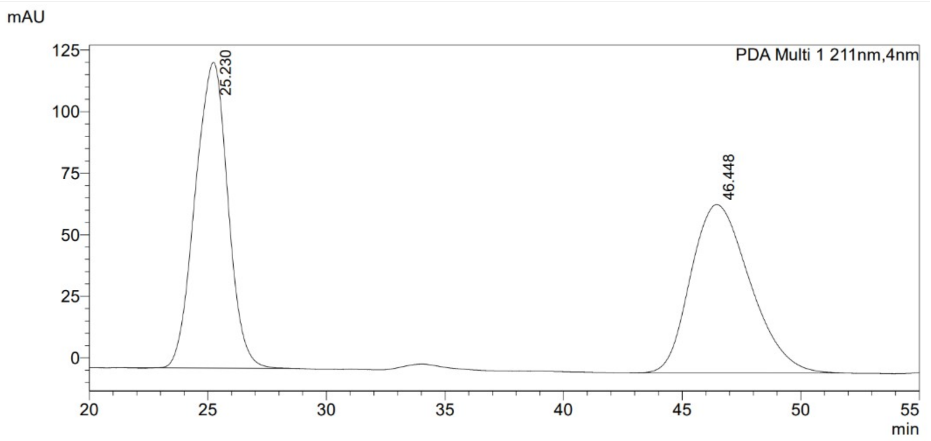

PDA Ch1 211nm

Peak#	Ret. Time	Area%
1	25.347	0.781
2	46.167	99.219
Total		100.000

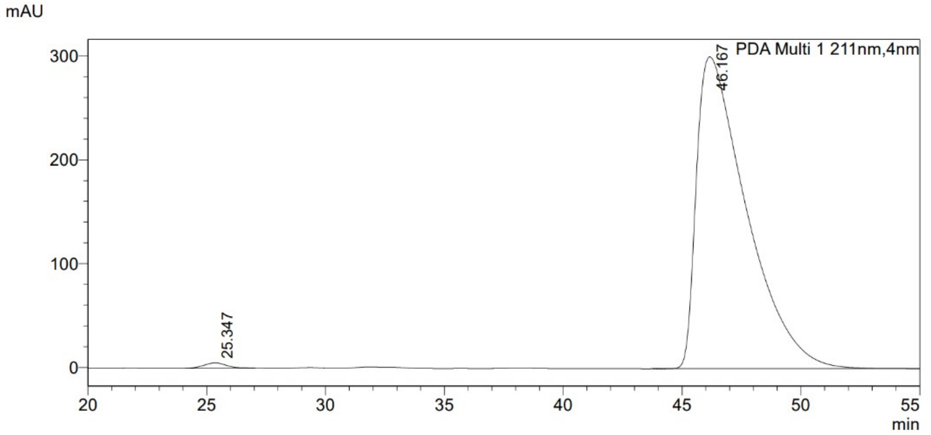

HPLC data for *syn*-**S23**: Chiralcel OD-H (93:7 *n*-hexane : IPA, flow rate 1.0 mLmin^{-1}, 211 nm, 40 °C) t_R (2*S*,3*S*) 16.9 min, t_R (2*R*,3*R*) 34.7 min, 99:1 er.

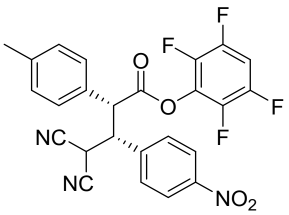

syn-**S23**

PDA Ch1 211nm

Peak#	Ret. Time	Area%
1	16.844	50.233
2	36.873	49.767
Total		100.000

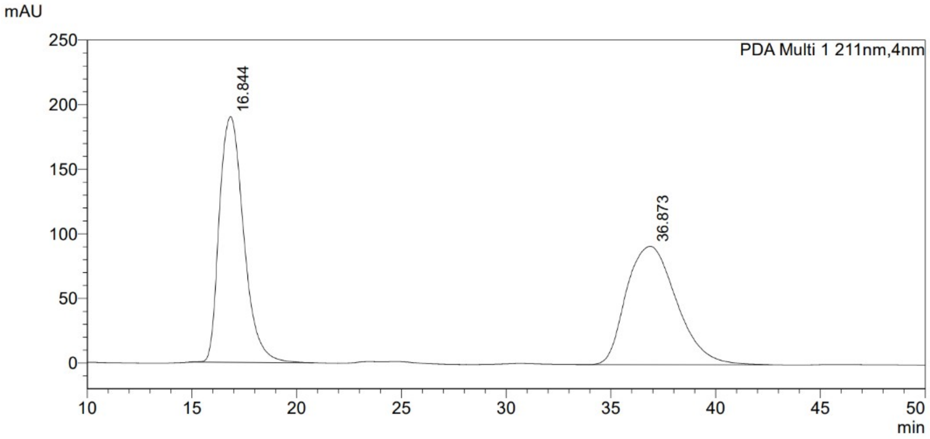

PDA Ch1 211nm

Peak#	Ret. Time	Area%
1	16.944	1.183
2	34.659	98.817
Total		100.000

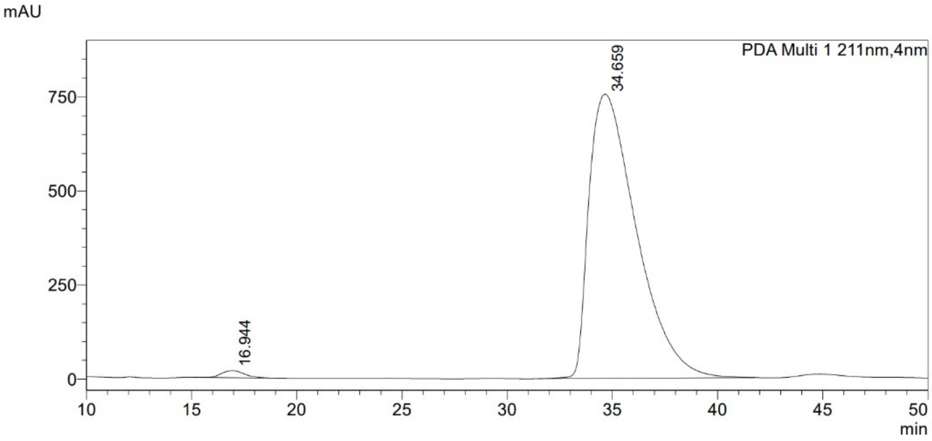

HPLC data for *anti*-**28**: Chiralcel OD-H (95:5 *n*-hexane : IPA, flow rate 1.0 mLmin^{-1}, 211 nm, 30 °C) t_R (2*S*,3*R*) 16.4 min, t_R (2*R*,3*S*) 23.4 min, 99:1 er.

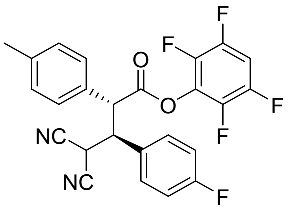

anti-**28**

PDA Ch1 211nm

Peak#	Ret. Time	Area%
1	16.020	49.928
2	22.924	50.072
Total		100.000

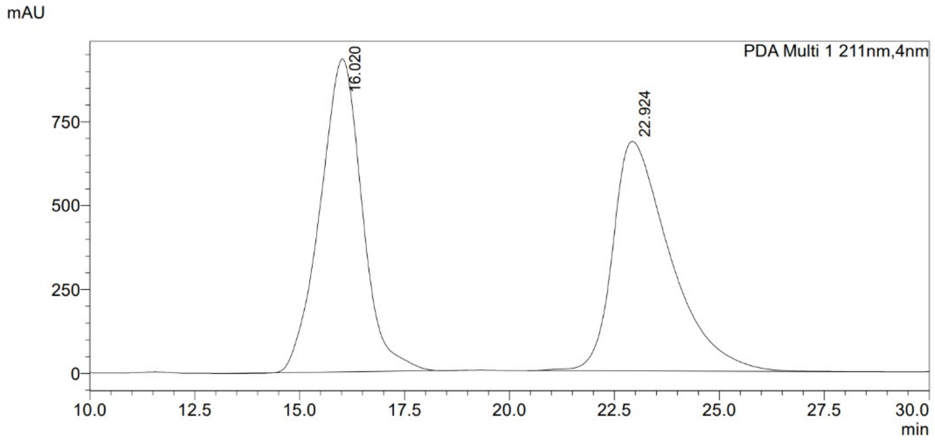

PDA Ch1 211nm

Peak#	Ret. Time	Area%
1	16.428	1.419
2	23.384	98.581
Total		100.000

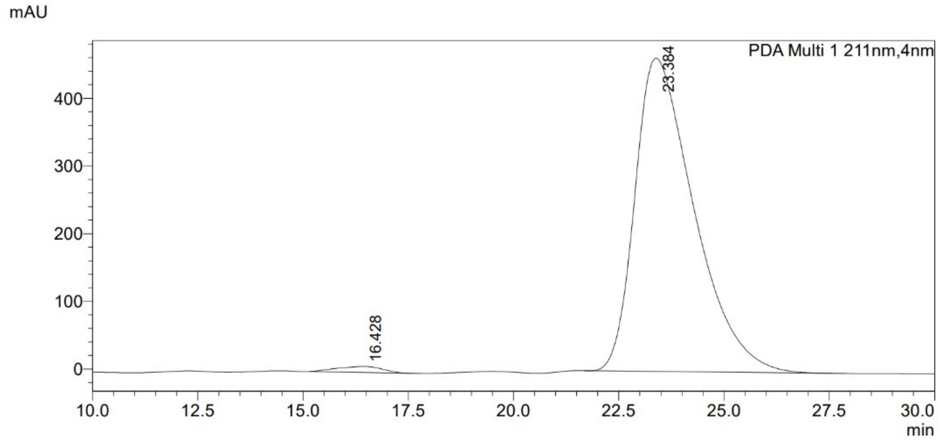

HPLC data for *anti*-**29**: Chiralcel OD-H (95:5 *n*-hexane : IPA, flow rate 1.0 mLmin^{-1}, 211 nm, 30 °C) t_R (2*S*,3*R*) 16.8 min, t_R (2*R*,3*S*) 27.9 min, 99:1 er.

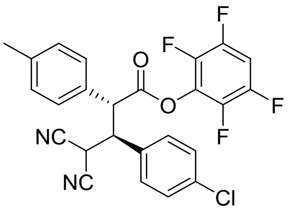

anti-**29**

PDA Ch1 211nm

Peak#	Ret. Time	Area%
1	16.876	49.951
2	28.173	50.049
Total		100.000

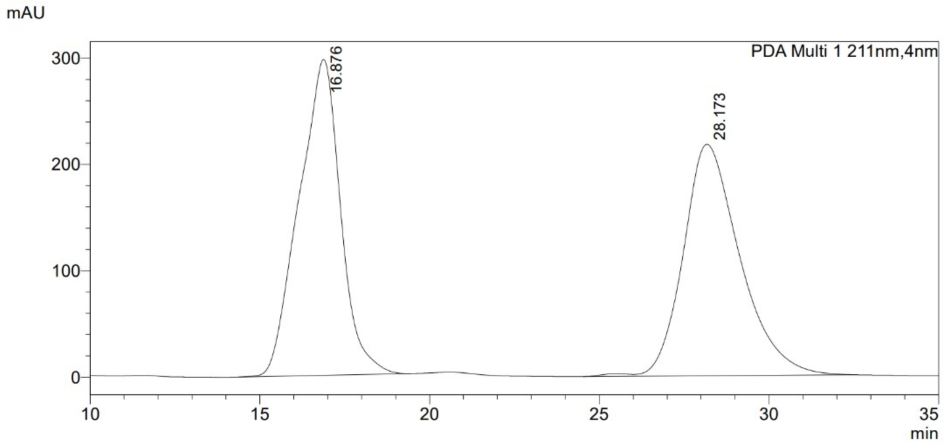

PDA Ch1 211nm

Peak#	Ret. Time	Area%
1	16.768	1.437
2	27.894	98.563
Total		100.000

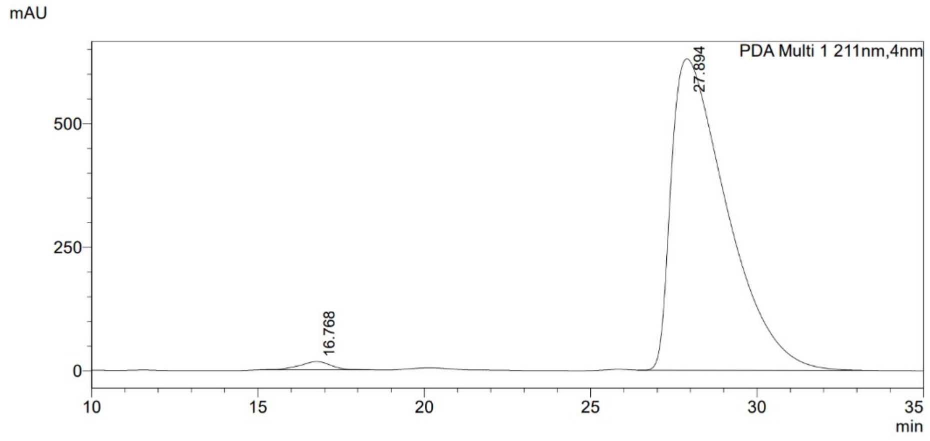

HPLC data for *syn*-**S24**: Chiralcel OD-H (93:7 *n*-hexane : IPA, flow rate 1.5 mLmin^{-1}, 211 nm, 30 °C) t_R (2*S*,3*S*) 6.5 min, t_R (2*R*,3*R*) 11.6 min, 96:4 er.

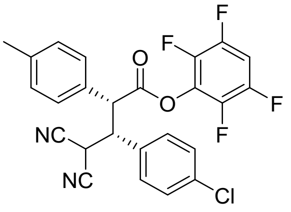

syn-**S24**

PDA Ch1 211nm

Peak#	Ret. Time	Area%
1	6.571	49.530
2	12.039	50.470
Total		100.000

PDA Ch1 211nm

Peak#	Ret. Time	Area%
1	6.516	4.194
2	11.615	95.806
Total		100.000

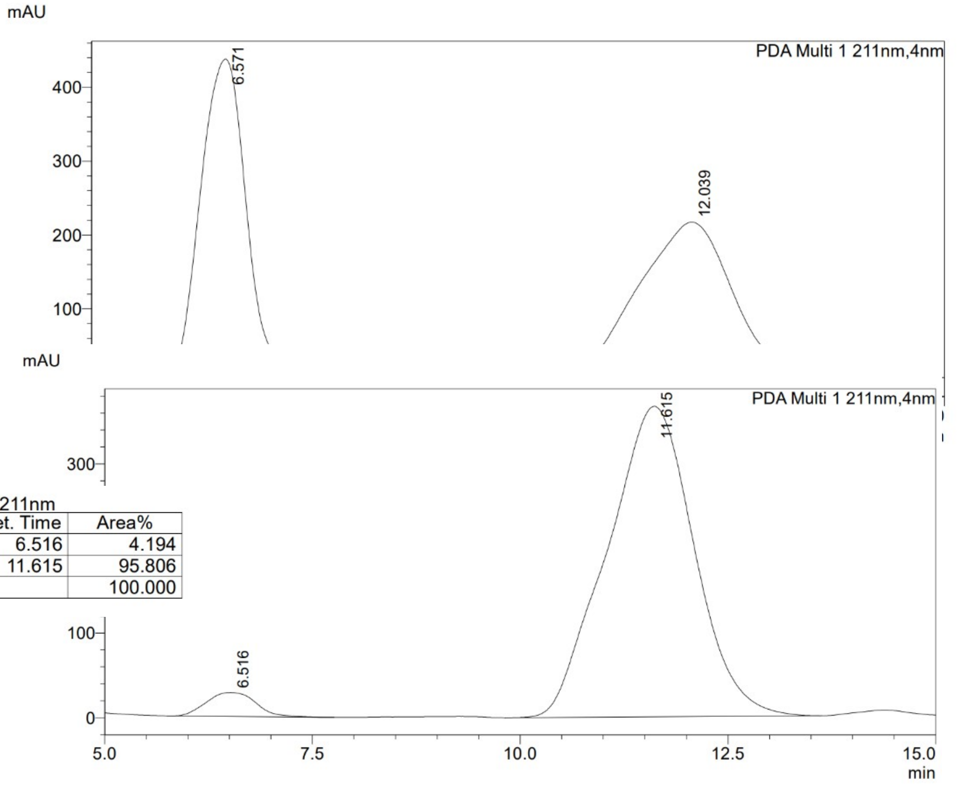

HPLC data for *anti*-**30**: Chiralcel OD-H (97:3 *n*-hexane : IPA, flow rate 1.0 mLmin^{-1}, 211 nm, 30 °C) t_R (2*S*,3*R*) 25.8 min, t_R (2*R*,3*S*) 31.2 min, 97:3 er.

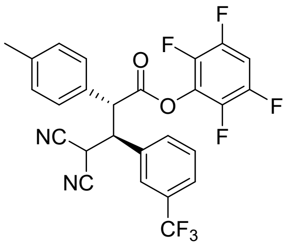

anti-**30**

PDA Ch1 211nm

Peak#	Ret. Time	Area%
1	24.838	50.416
2	32.031	49.584
Total		100.000

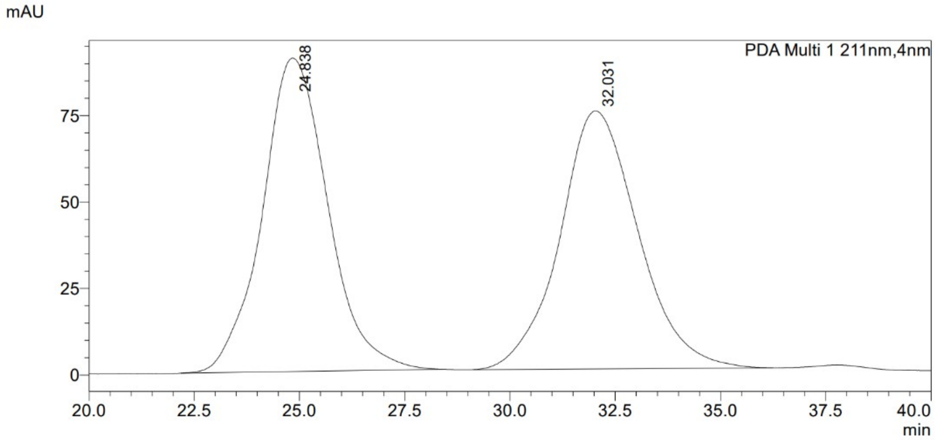

PDA Ch1 211nm

Peak#	Ret. Time	Area%
1	25.816	2.727
2	31.215	97.273
Total		100.000

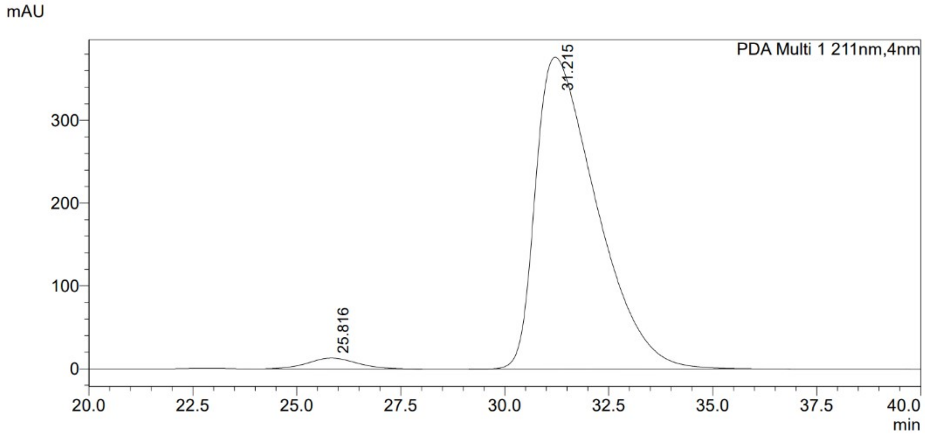

HPLC data for *syn*-**S25**: Chiralcel OD-H (97:3 *n*-hexane : IPA, flow rate 1.5 mLmin^{-1}, 211 nm, 30 °C) t_R (2*S*,3*S*) 12.3 min, t_R (2*R*,3*R*) 21.0 min, 89:11 er.

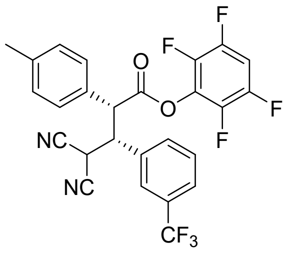

syn-**S25**

PDA Ch1 211nm

Peak#	Ret. Time	Area%
1	12.405	49.726
2	22.268	50.274
Total		100.000

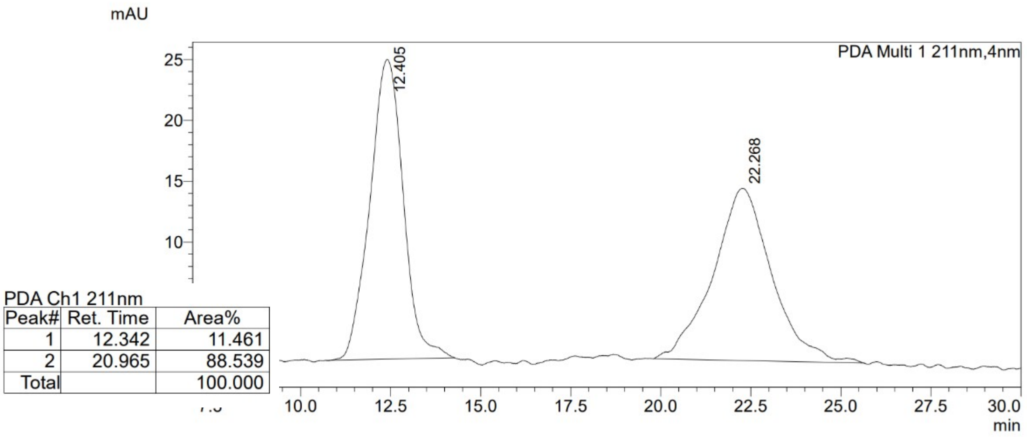

PDA Ch1 211nm

Peak#	Ret. Time	Area%
1	12.342	11.461
2	20.965	88.539
Total		100.000

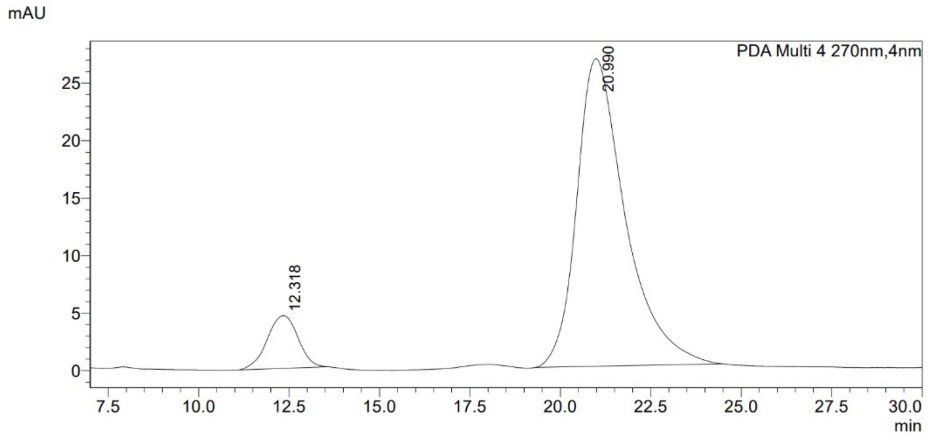

HPLC data for *anti*-**31**: Chiralpak AD-H (99:1 *n*-hexane : IPA, flow rate 1.0 mLmin^{-1}, 211 nm, 30 °C) t_R (2*S*,3*R*) 16.2 min, t_R (2*R*,3*S*) 18.3 min, 99:1 er.

HPLC data for *syn*-**S26**: enantiomeric ratio could not be determined.

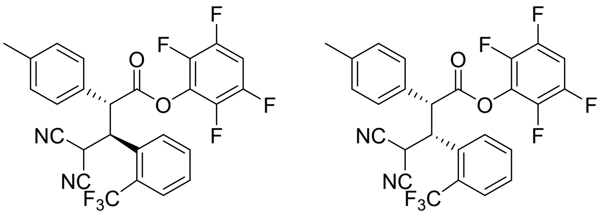

anti-**31** *syn*-**S26**

PDA Ch1 211nm

Peak#	Ret. Time	Area%
1	16.268	38.009
2	18.497	37.202
3	28.319	24.789
Total		100.000

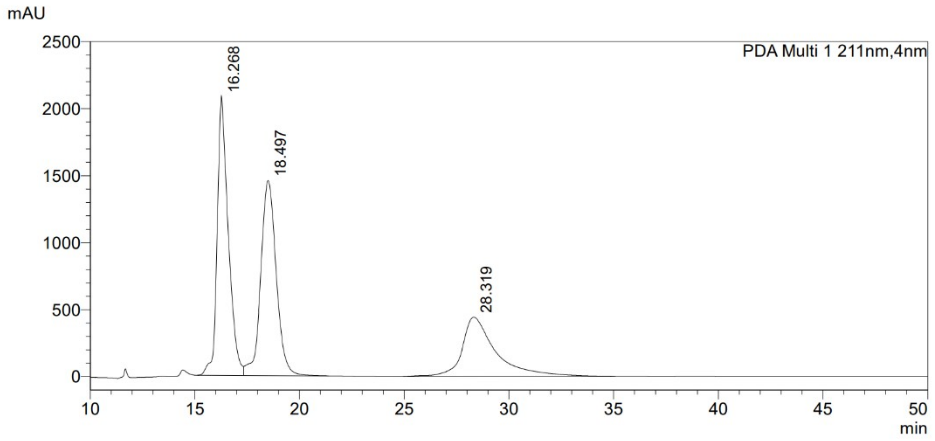

PDA Ch1 211nm

Peak#	Ret. Time	Area%
1	16.184	0.399
2	18.327	52.834
3	27.891	46.766
Total		100.000

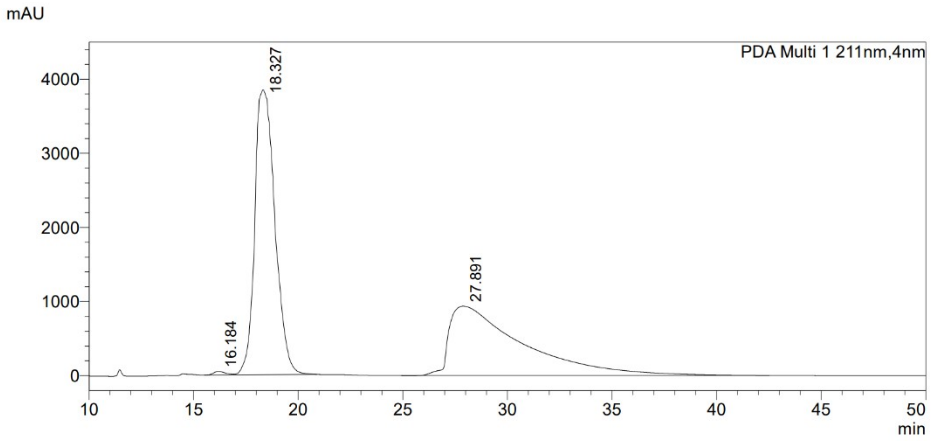

HPLC data for *anti*-**32**: Chiralcel OD-H (98:2 *n*-hexane : IPA, flow rate 1.5 mLmin^{-1}, 211 nm, 30 °C) t_R (2*S*,3*R*) 15.6 min, t_R (2*R*,3*S*) 19.0 min, 99:1 er.

HPLC data for *syn*-**S27**: Chiralcel OD-H (98:2 *n*-hexane : IPA, flow rate 1.5 mLmin^{-1}, 211 nm, 30 °C) t_R (2*S*,3*S*) 12.2 min, t_R (2*R*,3*R*) 25.4 min, 96:4 er.

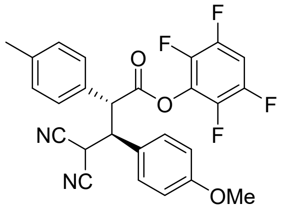

anti-**32**

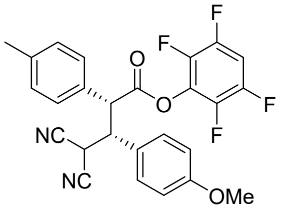

syn-**S27**

PDA Ch1 211nm

Peak#	Ret. Time	Area%
1	12.261	12.118
2	15.605	38.038
3	19.963	37.688
4	26.156	12.156
Total		100.000

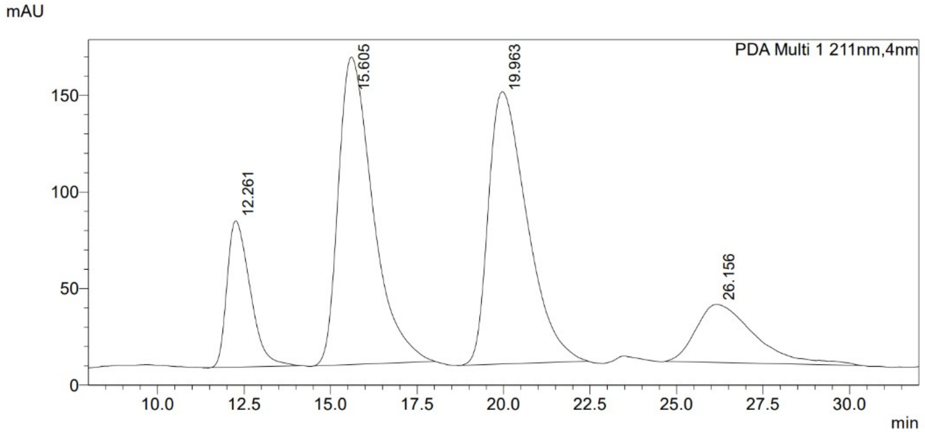

PDA Ch1 211nm

Peak#	Ret. Time	Area%
1	12.157	0.709
2	15.568	0.852
3	18.971	82.166
4	25.430	16.272
Total		100.000

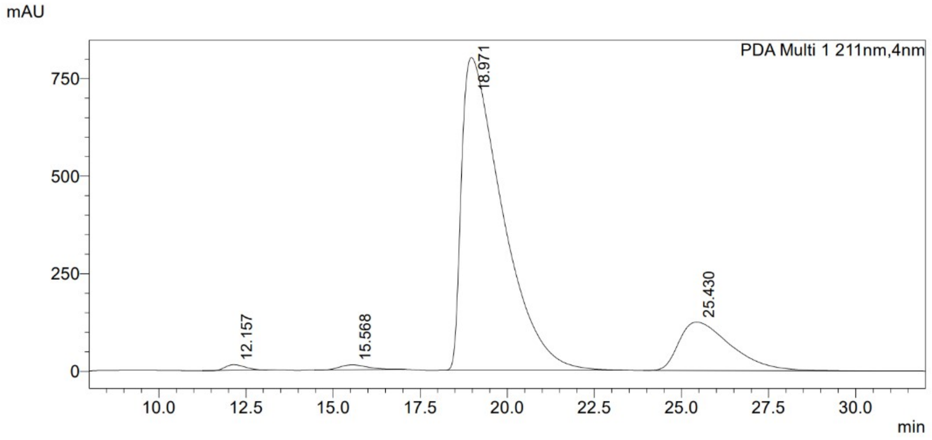

HPLC data for **37**: Chiralpak IB (90:10 *n*-hexane : IPA, flow rate 1.0 mLmin^{-1}, 211 nm, 30 °C) t_R (2*S*,3*R*) 8.8 min, t_R (2*R*,3*S*) 14.0 min, >99:1 er.

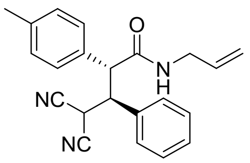

37

PDA Ch1 211nm

Peak#	Ret. Time	Area%
1	8.750	50.069
2	14.086	49.931
Total		100.000

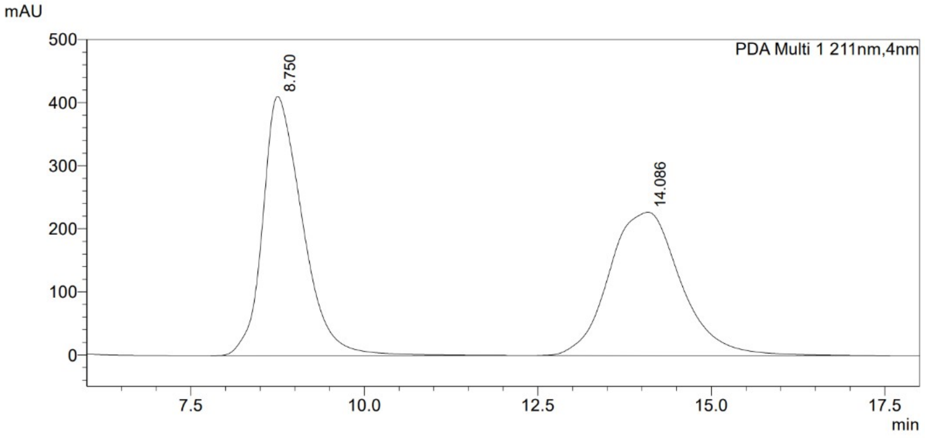

PDA Ch1 211nm

Peak#	Ret. Time	Area%
1	14.027	100.000
Total		100.000

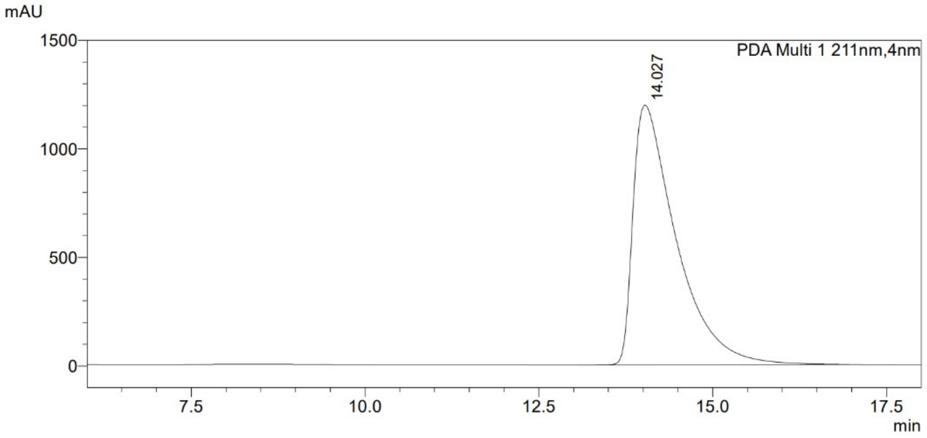

HPLC data for **38**: Chiralcel OD-H (99:1 *n*-hexane : IPA, flow rate 1.0 mLmin^{-1}, 211 nm, 30 °C) t_R (2*R*,3*S*) 19.4 min, t_R (2*S*,3*R*) 23.7 min, >99:1 er.

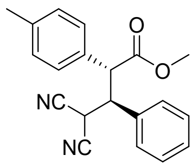

38

Detector A Channel 1 211nm

Peak#	Ret. Time	Area%
1	19.025	50.477
2	23.724	49.523
Total		100.000

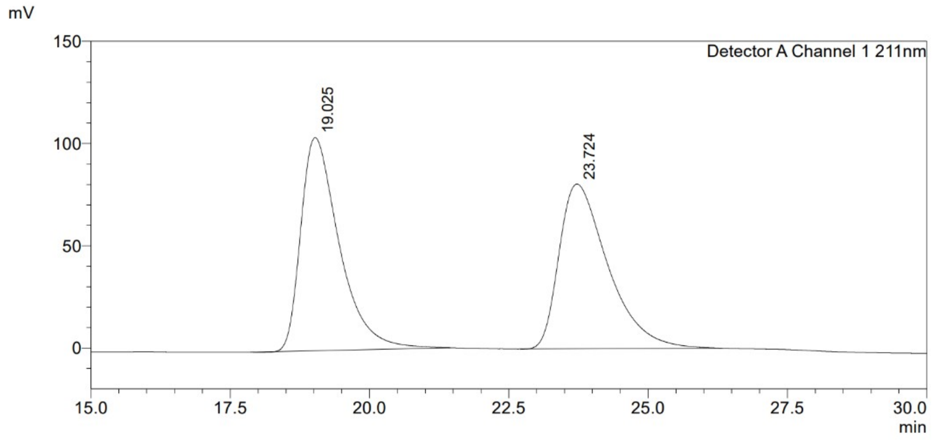

Detector A Channel 1 211nm

Peak#	Ret. Time	Area%
1	19.423	100.000
Total		100.000

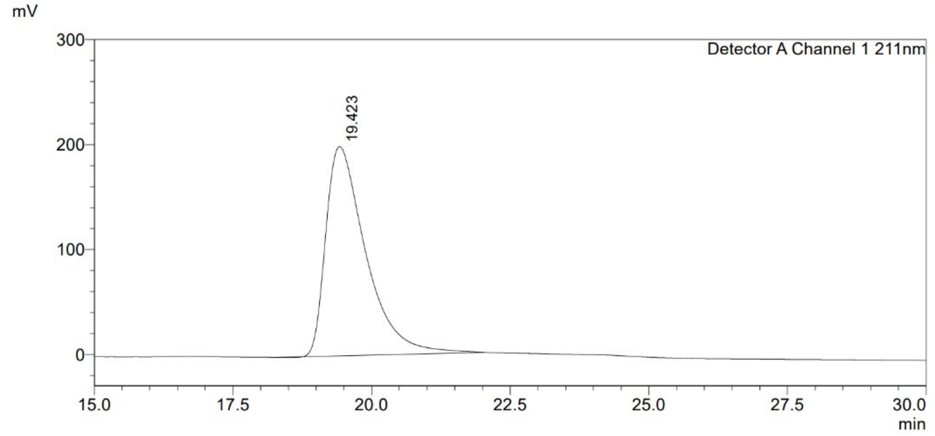

HPLC data for (2*R*,3*S*,4*R*)-**39**: Chiralpak ID (93:7 *n*-hexane : IPA, flow rate 1.0 mLmin^{-1}, 211 nm, 40 °C) t_R (2*R*,3*S*,4*R*) 23.1 min, t_R (2*S*,3*R*,4*S*) 24.3 min, >99:1 er.

HPLC data for (2*R*,3*S*,4*S*)-**S28**: Chiralpak ID (93:7 *n*-hexane : IPA, flow rate 1.0 mLmin^{-1}, 211 nm, 40 °C) t_R (2*S*,3*R*,4*R*) 21.6 min, t_R (2*R*,3*S*,4*S*) 33.7 min, >99:1 er.

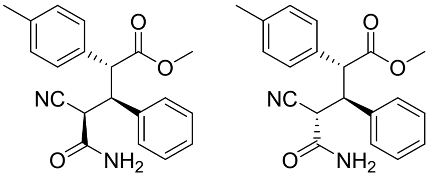

(2*R*,3*S*,4*R*)-**39** (2*R*,3*S*,4*S*)-**S28**

Detector A Channel 1 211nm

Peak#	Ret. Time	Area%
1	21.586	5.269
2	23.092	42.848
3	24.315	47.554
4	33.998	4.329
Total		100.000

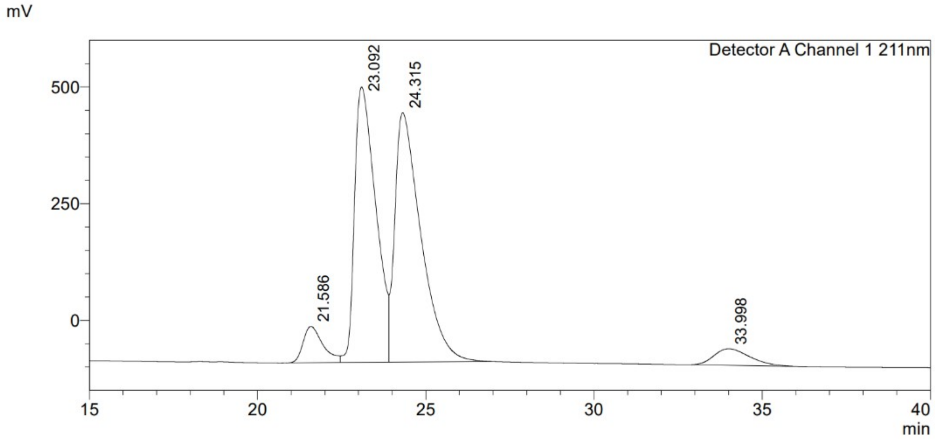

Detector A Channel 1 211nm

Peak#	Ret. Time	Area%
1	23.116	90.582
2	33.703	9.418
Total		100.000

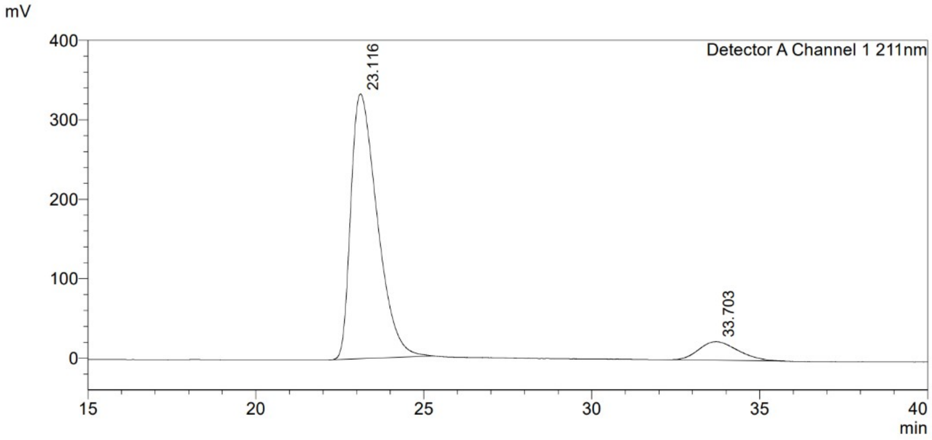

Supplement: SC-014-D3SC02101G-s001 [file SC-014-D3SC02101G-s001.pdf]
